# IL-17 Induces an Expanded Range of Downstream Genes in Reconstituted Human Epidermis Model

**DOI:** 10.1371/journal.pone.0090284

**Published:** 2014-02-28

**Authors:** Andrea Chiricozzi, Kristine E. Nograles, Leanne M. Johnson-Huang, Judilyn Fuentes-Duculan, Irma Cardinale, Kathleen M. Bonifacio, Nicholas Gulati, Hiroshi Mitsui, Emma Guttman-Yassky, Mayte Suárez-Fariñas, James G. Krueger

**Affiliations:** 1 Laboratory for Investigative Dermatology, The Rockefeller University, New York City, New York, United States of America; 2 Center for Clinical and Translational Science, The Rockefeller University, New York City, New York, United States of America; 3 Department of Dermatology, University of Rome “Tor Vergata”, Rome, Italy; 4 Department of Dermatology, Mount Sinai School of Medicine, New York City, New York, United States of America; Keio University School of Medicine, Japan

## Abstract

**Background:**

IL-17 is the defining cytokine of the Th17, Tc17, and γδ T cell populations that plays a critical role in mediating inflammation and autoimmunity. Psoriasis vulgaris is an inflammatory skin disease mediated by Th1 and Th17 cytokines with relevant contributions of IFN-γ, TNF-α, and IL-17. Despite the pivotal role IL-17 plays in psoriasis, and in contrast to the other key mediators involved in the psoriasis cytokine cascade that are capable of inducing broad effects on keratinocytes, IL-17 was demonstrated to regulate the expression of a limited number of genes in monolayer keratinocytes cultured in vitro.

**Methodology/Principal Findings:**

Given the clinical efficacy of anti-IL-17 agents is associated with an impressive reduction in a large set of inflammatory genes, we sought a full-thickness skin model that more closely resemble in vivo epidermal architecture. Using a reconstructed human epidermis (RHE), IL-17 was able to upregulate 419 gene probes and downregulate 216 gene probes. As possible explanation for the increased gene induction in the RHE model is that C/CAAT-enhancer-binding proteins (C/EBP) -β, the transcription factor regulating IL-17-responsive genes, is expressed preferentially in differentiated keratinocytes.

**Conclusions/Significance:**

The genes identified in IL-17-treated RHE are likely relevant to the IL-17 effects in psoriasis, since ixekizumab (anti-IL-17A agent) strongly suppressed the “RHE” genes in psoriasis patients treated in vivo with this IL-17 antagonist.

## Introduction

Psoriasis is a chronic inflammatory skin disorder characterized by a dense dermal inflammatory infiltrate and altered keratinocyte (KC) differentiation [1]. Leukocytes that infiltrate the dermis produce many pro-inflammatory mediators that set up the cycle of pathogenic inflammation. Interleukin (IL)-17 has emerged as one of the most crucial players in the current model of psoriasis pathogenesis. IL-17 was thought to be produced mainly by Th17 cells, a subset of CD4+ T helper cells that is distinct from the Th1 and Th2 lineages [2], [3], but it is becoming increasingly appreciated that it is also produced by CD8+ T cells (Tc1) and γδ T cells [4], [5], and potentially by some non-T cells, including mast cells and neutrophils [6]. IL-17 signaling activates the Nuclear factor kappa-light-chain-enhancer of activated B cells (NF-κB) pathway and the C/CAAT-enhancer-binding proteins (C/EBP) family, particularly C/EBPβ and C/EBPδ [7], [8] to enhance expression of pro-inflammatory cytokines and chemokines, intercellular adhesion molecules, and anti-microbial peptides (AMPs) by numerous cell types, including granulocytes, chondroblasts, fibroblasts, and epithelial cells (keratinocytes, endothelial cells, and mucosal epithelial cells) [9]–[16].

A crucial role of IL23/Th17 axis in the pathogenesis of psoriasis was proposed based on several recent studies: (i) dermal IL-17-producing CD4+ T cell and γδ T cell infiltrate as well as (ii) IL-17-producing CD8+ T cells within psoriatic epidermis; (iii) high expression levels of IL-23, IL-17, and IL-22 in psoriatic lesional skin; (iv) high serum levels of IL-22 and IL-17 that correlated with disease severity score [4], [5], [17]–[21]. Moreover, some of the Th17 pathway-related genes, IL-23A subunit, IL-23R, IL23B subunit, have been identified as psoriasis susceptibility genes [22]–[24]. Responses to tumor necrosis factor (TNF)α-blocking therapy and narrow-band ultraviolet B light therapy are correlated with the suppression of Th17 pathway [20], [25]–[27]. More recently, therapeutic approaches suppressing the IL-23/Th17 axis have proved highly effective in the treatment of psoriasis [28]–[33].

Keratinocytes are the key-responding cells to the psoriatic pro-inflammatory and pro-proliferative microenvironment since they bear receptors for key inflammatory cytokines, including IL-17 [12], [34], [35]. Surprisingly, although anti-IL-17 therapies showed astonishing clinical efficacy in improving psoriasis, in vitro studies of cultured monolayer keratinocytes identified a restricted number of genes induced by IL-17 [10], [12]. This discrepancy between the biological effects of neutralizing IL-17 and the IL-17–induced gene expression is even more pronounced considering other key-cytokines, such as TNFα and interferon (IFN)-γ, are capable of broadly regulating genomic expression in keratinocytes (502 and 3549 gene transcripts induced by TNFα and IFN-γ, respectively) [12]. Hence, in order to investigate this discrepancy further, we analyzed expression of C/EBPβ, a downstream signaling molecule of IL-17, and found that it localizes to the uppermost layers of the human epidermis in non-lesional and lesional psoriatic skin, indicating that perhaps only mature keratinocytes fully respond to IL-17 stimulation. We, therefore, sought to create in vitro conditions that could more closely mimic the in vivo epidermal architecture. Using Reconstructed Human Epidermis (RHE), a 3D epidermal skin model composed of a keratinocyte multi-layer supported by connective tissue, we evaluated the genomic response to IL-17.

Our results suggest IL-17 acts as a regulator of inflammatory gene expression preferentially in differentiated keratinocytes and induced genes include many psoriasis-related transcripts.

## Results

### Increased expression of the IL-17-specific transcription factor, C/EBPβ, by terminally differentiated keratinocytes

Normal human keratinocytes were found to constitutively bear the IL-17 receptor (IL-17R) and they are able to produce several IL-17-induced inflammatory and immune-related mediators implicated in psoriasis pathogenesis (e.g. IL-8, CCL20, S100A12, CXCL1, and CXCL2).

**Figure 1:**
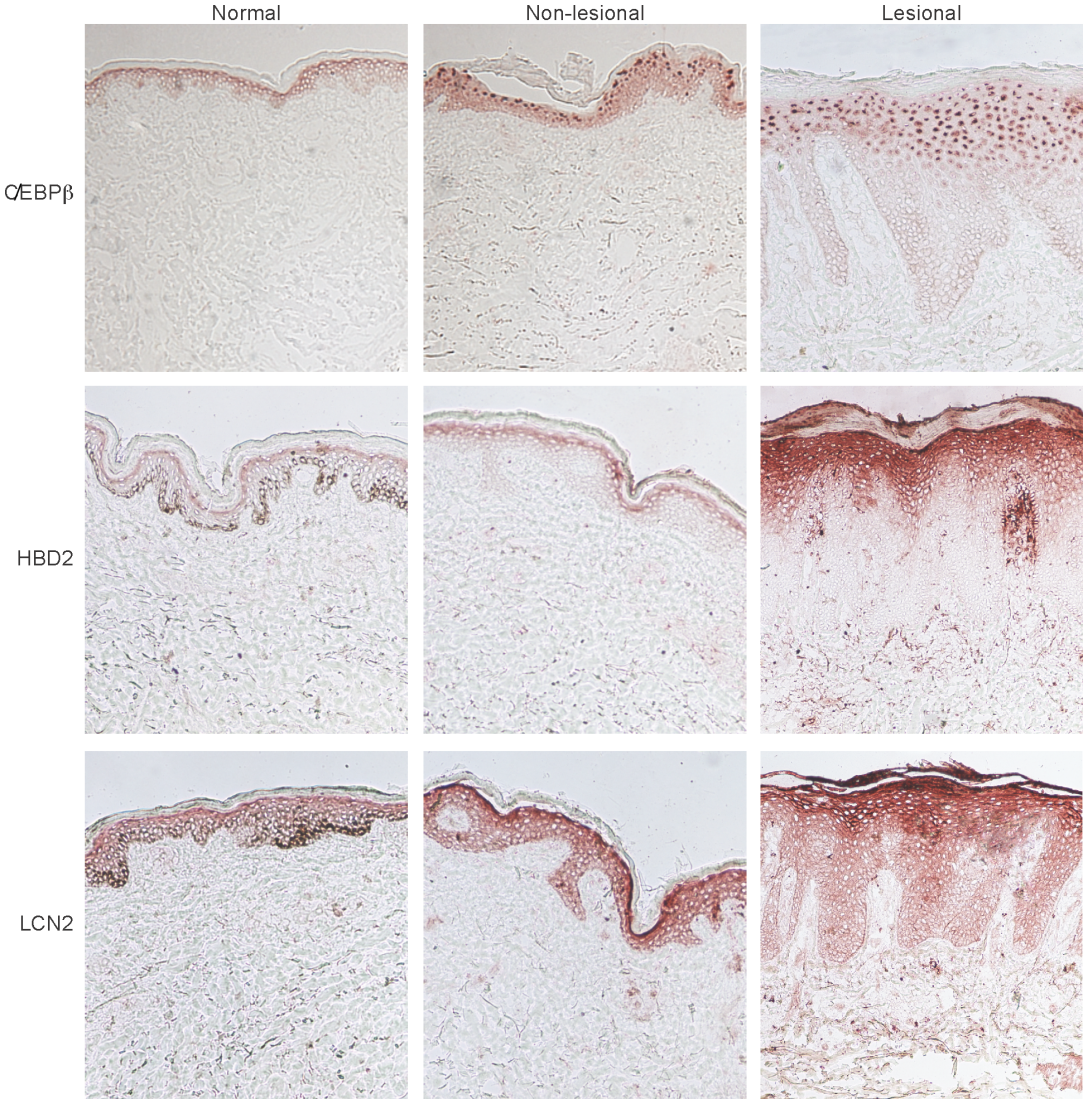
IL-17-regulated C/EBPβ, human β-defensin 2, and lipocalin are expressed by terminally differentiated keratinocytes. Immunohistochemistry for C/EBPβ (top), human β-defensin 2 (HBD2, middle), and lipocalin (LCN-2, bottom) in normal, non-lesional or lesional psoriatic skin showing predominant expression in the spinous-granular layer of the epidermis.

Unlike the constitutively expressed IL-17R, C/EBPβ, a critical transcriptional factor in the IL-17 signaling cascade, was detected only within more mature keratinocytes localized to the upper spinous-granular layers of the epidermis (Figure 1). C/EBPβ staining showed a nuclear pattern that was slightly detectable in the uppermost layers of the epidermis in normal skin, while it was very evident in the epidermis of non-lesional and lesional psoriatic skin. In addition to C/EBPβ, IL-17-regulated proteins, such as human β-defensin 2 (HBD2) and lipocalin 2 (LCN2), also localized to the outermost spinous-granular layers (Figure 1). Immunofluorescence staining further illustrated the co-localization of C/EBPβ and HBD2 in psoriatic skin (Figure S1).

In order to verify whether this staining pattern was specific for C/EBPβ, we examined the expression patterns of additional epidermal transcriptional factors: RFX5, which is not usually overexpressed in lesional psoriatic skin, and STAT (Signal Transducers and Activators of Transcription) −1, a crucial mediator of IFN-γ signaling that has also been implicated in psoriasis pathogenesis. In contrast to the localization C/EBPβ to the spinous-granular layers of the epidermis, RFX5 was only localized to the basal layer of the epidermis (Figure S2). On the other hand, STAT1 was expressed by all viable keratinocytes as shown by pan-epidermal staining (Figure S2). These distinct staining patterns suggest that transcription factors may be activated in different types of KCs (e.g. basal KCs versus granular KCs).

As further confirmation of increased expression of C/EBPβ in differentiated keratinocytes, we accessed data from a new study that measured mRNAs in human dermis, basal epidermis, and suprabasal epidermis after laser-capture microdissection [36] C/EBPβ mRNA was increased about 4–fold in suprabasal epidermis compared to the basal layer (Figure S3).

Furthermore, attempting to fully differentiate monolayer *in vitro* normal human epidermal keratinocytes (NHEKs) using different calcium concentrations, we obtained a significantly higher C/EBPβ expression in high-calcium-treated NHEKs compared to low-calcium condition. Together with C/EBPβ, we tested the expression of some keratinocyte-terminal differentiation genes such as involucrin (IVL), transglutaminase-1 (TGM1), and filaggrin-2 (FLG2). Keratinocytes differentiated after culturing in high Ca^++^ plus 2% fetal bovine serum (FBS) medium, showed enhanced expression of C/EBPβ (p = 0.03) as well as high expression of FLG2, TGM1, and IVL mRNAs (p<0.002 for all) (Figure S4).

### IL-17 induces a large number of genes in RHE

The fact that C/EBPβ and some IL-17-regulated proteins are expressed mainly by terminally differentiated KCs may explain why IL-17 induces such a limited number of genes in primary KCs cultured in vitro, as these KCs maintain a more undifferentiated, basal phenotype.

**Figure 2:**
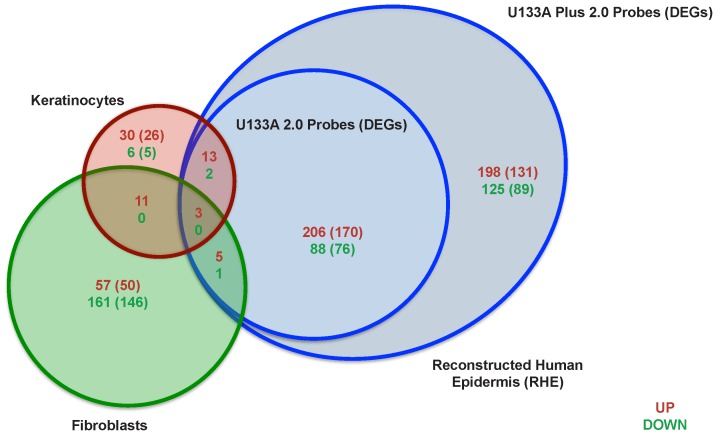
IL-17 induces a large number of genes in RHE. Venn diagram illustrates the number of up-regulated (red) and down-regulated (green) probe-sets with the number of unique DEGs in parentheses of IL-17-treated keratinocytes, fibroblasts or RHE compared to the respective untreated conditions. U133A 2.0 arrays were used for KC and fibroblasts, while U133A Plus 2.0 arrays were used for RHE (FCH >1.5 and FDR<0.1 were used for all arrays). The additional semi-circle of RHE genes represents the probe-sets (DEGs) that were not present in the U133A 2.0 arrays.

Therefore, in order to fully characterize the genes induced by IL-17 in KCs, we used a Reconstructed Human Epidermis (RHE) model, a full thickness epidermal skin structure, consisting of normal human-derived epidermal KCs organized into basal, spinous, granular, and cornified layers, analogously to those found in vivo. This epidermal structure is supported by connective tissue including fibroblasts. RHE was incubated with IL-17 (200 ng mL−1), IL-22 (200 ng mL−1), or IFN-γ (20 ng mL−1) for 48 hours and the cytokine-induced gene expression levels were measured with AffymetrixU133A Plus 2.0 arrays. In order to align the gene array analysis with the previously published data [12], we compared gene expression levels in IL-17-treated RHE versus untreated RHE using the selection criteria of fold change (FCH) >1.5 and false discovery rate (FDR)<0.1 that were used for the monolayer KC gene expression analysis [12]. In contrast to monolayer KCs [12] in which IL-17 altered the expression of only 65 probe-sets (60 unique differentially expressed gene, DEGs, using ENTREZ identifiers), in RHE, IL-17 induced the expression of many more genes (641 probe-sets, representing 490 DEGs) (Table S1), of which 425 probe-sets (322 DEGs) resulted upregulated and 216 probe-sets (168 DEGs) downregulated (Figure 2).

To demonstrate that the IL-17 response in RHE did not merely represent the sum of the genes induced by IL-17 in fibroblasts and KCs, the RHE gene set was compared to gene sets obtained from in vitro cultured KCs or fibroblasts treated with IL-17. Differential expression induced by IL-17 in KCs or fibroblasts was compared to the respective untreated conditions. A subset of transcripts (323 probe-sets) was only detected in RHE with U133A plus 2.0 arrays, as these probe-sets were not present on the U133A 2.0 arrays, which were used for treated fibroblasts and KCs (Figure 2, semi-circle). Even when the analysis was restrictedly performed with U133A 2.0 arrays, the number of upregulated RHE gene transcripts (227 probe-sets) resulted about 3-fold and 4-fold higher than the number of upregulated gene detected in fibroblasts and KCs, respectively. There was very little overlap in the IL-17-regulated genes between the three conditions (9 DEGs were detected in both RHE and fibroblasts and 18 DEGs in both KCs and RHE). While there were some KC or fibroblast-specific genes, a large number of genes were uniquely expressed in RHE (Figure 2 and Table S1).

### IL-17 induces the expression of C/EBPβ and many inflammatory genes in RHE

We also verified the expression of C/EBPβ in RHE model, eventually reflecting the *in vivo* condition. In the IL-17-treated RHE compared with untreated RHE, we detected an upregulation of C/EBPβ gene transcripts by polymerase chain reaction (PCR), and also, protein expression of C/EBPβ within the granular layer cells by immunohistochemistry (Figure S5). The expression of C/EBPβ induced by IL-17 in RHE correlates with the upregulation of Th17 pathway genes, such as IL23A, STAT3, and DEFB4 (Table 1 and Table S1). IL-17 also induced a number of anti-microbial peptides, including S100A12, S100A7A, SERPINB4, SERBINB3, which are highly expressed in psoriasis (Table 1) [37], as has been described previously [12]. Some other characteristic IL-17-regulated genes, such as IL8, IL6, CCL20, CXCL2, CXCL3, CXCL5, and LCN2 were up-regulated to a lesser extent (1.3–2.3 fold induction), but these elevations did not pass significance thresholds (Table S1). Interestingly, IL-1 family members, IL1A, IL1B, IL1F8, and IL1F9 were significantly activated by IL-17 in this model (Table 1). Signaling through these cytokines activates NF-κB, which may synergize with IL-17-induced C/EBPs to enhance transcription of many IL-17-regulated genes, further amplifying the inflammatory loops in psoriasis. In addition, there was up-regulation of cytokines, which limit NF-κB activation, such as IL1F5 and IL11, suggesting that IL-17 may also induce control mechanisms to prevent excessive inflammation [38], [39].

**Table 1:** Selected immune-related genes expressed in IL-17-treated RHE.

Symbol	Description	FCH^1^	FDR^2^
**DEFB4A**	defensin, beta 4A	24.37	0.005
**IL1F9**	interleukin 1 family, member 9	16.37	0.001
**IL19**	interleukin 19	12.82	0.007
**S100A7A**	s100 calcium binding protein A7A	12.70	0.011
**IL23A**	interleukin 23, alpha subunit, p19	11.53	0.001
**SERPINB4**	serpin peptidase inhibitor, clade B, member 4	7.03	0
**TGFA**	transforming growth factor, alpha	5.04	0.035
**S100A12**	s100 calcium binding protein A12	4.85	0.013
**IL1F8**	interleukin 1 family, member 8 (eta)	4.34	0.086
**C/EBPA**	CCAAT/enhancer binding protein (C/EBP), alpha	4.28	0.006
**IL1B**	interleukin 1, beta	3.94	0.013
**IL1B**	interleukin 1, beta	3.89	0.038
**RNASE7**	ribonuclease, RNase A family, 7	3.48	0.005
**IL11**	interleukin 11	3.42	0.045
**RNASE7**	ribonuclease, RNase A family, 7	3.23	0.013
**SERPINA1**	serpin peptidase inhibitor, clade A, member 1	3.08	0.058
**SERPINA1**	serpin peptidase inhibitor, clade A, member 1	2.88	0.089
**TGFA**	transforming growth factor, alpha	3.08	0.046
**IL1A**	interleukin 1, alpha	2.85	0.038
**IL1F5**	interleukin 1 family, member 5 (delta)	2.68	0.01
**MAP3K9**	mitogen-activated protein kinase kinasekinase 9	2.58	0.044
**RNASE7**	ribonuclease, RNase A family, 7	2.30	0.031
**STAT3**	signal transducer and activator of transcription 3	2.18	0.021
**MAP2K3**	mitogen-activated protein kinase kinase 3	1.97	0.03
**SERPINB3**	serpin peptidase inhibitor, clade B, member 3	1.88	0.01

1 FCH, fold change;

2 FDR, false discovery rate.

### IL-17 modulates keratinocyte mitogens and cell-cycle genes

Intriguingly, we observed an induction of cytokines that induce epidermal hyperplasia, including IL19 and heparin-binding EGF-like growth factor (HBEGF). Thus, IL-17 might indirectly stimulate KC proliferation and epidermal hyperplasia via paracrine cytokine secretion. There was also over-expression of cell cycle-related genes, such as cyclin E1 (CCNE1), cell division cycle associated 5 (CDCA5), and cell division cycle 25 homolog A (CDC25A). IL-17 likely stimulates epidermal keratinocyte differentiation, modulating the expression of KC differentiation genes including transglutaminase (TGM)-1, TGM3, small proline-rich protein 2C and 4 (SPRR2C, SPRR4), kallikrein-related peptidase 6, 10, and 13 (KLK6, KLK10, KLK13), and cornifelin (CNFN). Furthermore, IL-17 also modulates the expression of genes, including sphingomyelin phosphodiesterase 1, acid lysosomal (SMPD1) and serine palmitoyltransferase, long chain base subunit 2(SPTLC2), which are related to lamellar bodies and epidermal lipid barrier formation (Table 2).

**Table 2:** Selected genes involved in keratinocyte proliferation and differentiation.

Symbol	Description	FCH^1^	FDR^2^	Biological Function
**IL19**	interleukin 19	12.82	0.01	Epidermal hyperplasia inducers
**HBEGF**	herparin-binding EGF-like growth factor	3.29	0.03	
**SPRR2C**	smallproline-rich protein 2C (pseudogene)	27.07	0.00	
**TGM3**	transglutaminase 3	7.15	0.01	
**SPRR4**	smallproline-rich protein 4	5.21	0.01	
**KLK13**	kallikrein-related peptidase 13	4.75	0.10	
**KLK13**	kallikrein-related peptidase 13	4.57	0.08	KC differentiation-related genes
**TGM1**	transglutaminase 1	3.01	0.02	
**KLK6**	kallikrein-related peptidase 6	2.89	0.04	
**CNFN**	cornifelin	2.80	0.05	
**KLK10**	kallikrein-related peptidase 10	2.13	0.01	
**SMPD1**	sphingomyelinphosphodiesterase 1, acid lysosomal	2.61	0.01	Laminar bodies/extra-cellular lipids formation
**SPTLC2**	serinepalmitoyltransferease, long chain base subunit 2	2.54	0.08	
**CCNE1**	cyclin E1	2.50	0.02	
**CDCA5**	cell division cycle associated 5	1.88	0.04	Cell cycle-related genes
**CDC25A**	cell division cycle 25 homolog A (S. pombe)	1.68	0.01	

1 FCH, fold change;

2 FDR, false discovery rate.

### RHE showed a specific genomic response to IL-17 stimulation

To verify whether the RHE genomic response to IL-17 stimulation was specific to IL-17, we stimulated RHE with IFN-γ or IL-22, cytokines thought to be involved in the psoriasis inflammatory cascade. The IL-22 response in KCs or RHE was minimal with only 35 probe-sets induced in RHE and 23 in KCs (data not shown). The response to IFN-γ was strong, with upregulation of 294 transcripts (Figure S6). As illustrated in Figure S6A, there was minimal overlap in the genes induced by IL-17 or IFN-γ. Furthermore, scatter plots comparing the genes induced in RHE versus *in vitro* cultured KCs showed that IL-17 induced a large number of genes only in RHE, while on the contrary, IFN-γ, induced a larger number of genes in monolayer KCs compared to RHE (Figure S6B), consistent with the constitutive expression of STAT1 by all KCs at all stages of differentiation (Figure S2).

**Figure 3:**
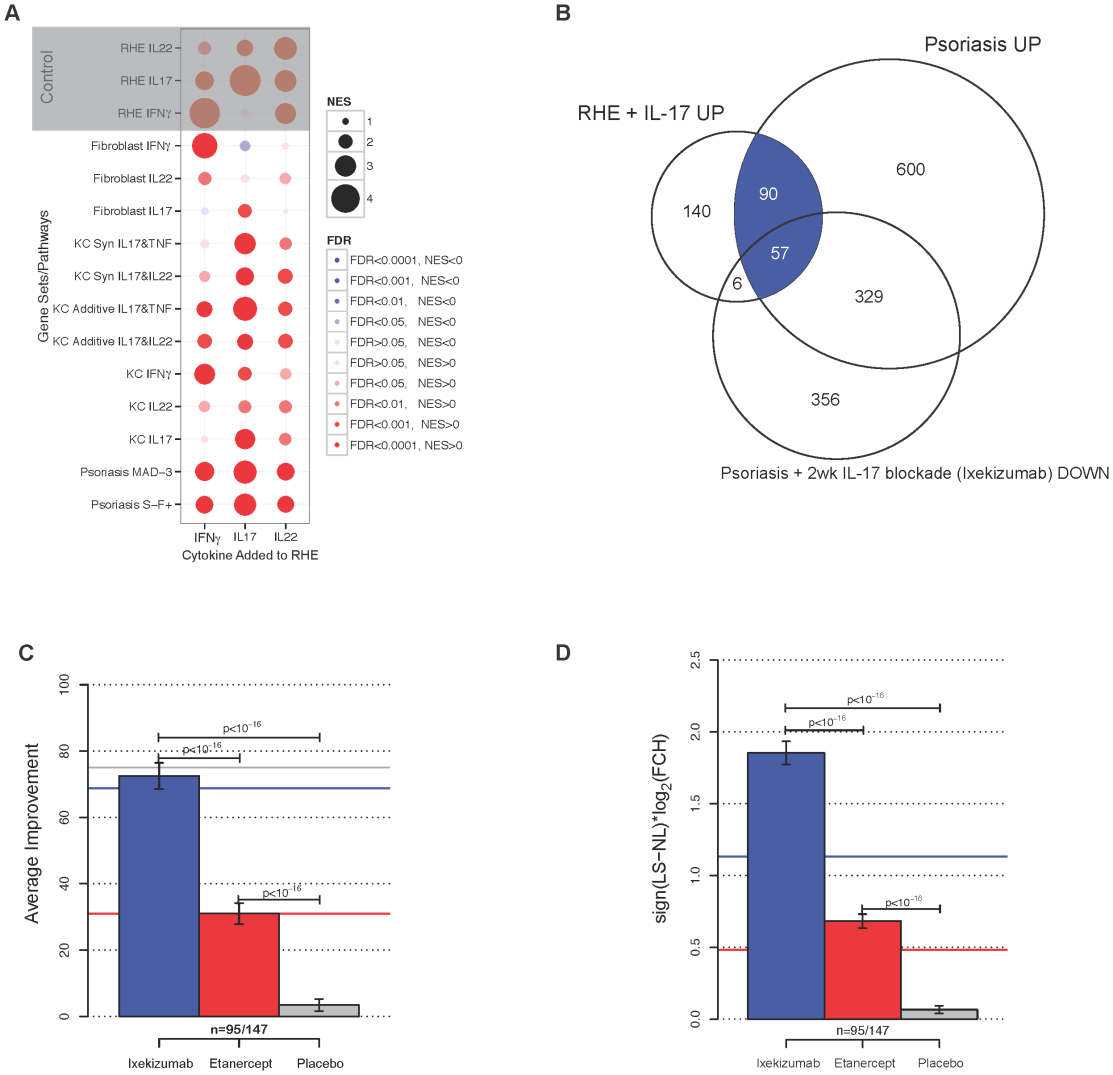
Improvement of psoriasis with IL-17 blockade is associated with reduced expression of IL-17-induced RHE genes. (**A**) Correlation between various gene sets and RHE gene profile response to cytokine stimulation (IL-17, IFN-γ, or IL-22) using GSEA. NES: normalized enrichment score; FDR: false discovery rate. (**B**) Venn diagram summarizing the number of DEGs among those in the psoriasis transcriptome or IL-17-treated RHE with improvement of at least 75% at two weeks post-ixekizumab. (**C**) Proportion of genes in IL-17-treated RHE that were differentially regulated in psoriasis (blue shaded area of (**A**)) and on the U133A 2.0 arrays (n = 95 out of 147 total DEGs which included additional genes only seen on the U133A Plus 2.0 arrays) that responded to treatment with IL-17 blockade (Ixekizumab, blue), TNF blockade (etanercept, red) or placebo (gray) at 2 weeks. Colored lines are changes in all MAD-3 psoriasis genes after both treatments. (**D**) The average change in expression (log_2_FCH) of RHE+IL-17 genes toward recovery with ixekizumab, etanercept, or placebo at 2 weeks.

As confirmation of the specific RHE response to cytokine stimulation, we performed Reverse transcriptase-PCR (RT-PCR) (Figure S6C) testing some genes including IL23A, S100A7A, IL19, and IL1F8, which appeared to be upregulated by IL-17 in RHE, as listed in Table S1. We also correlated RHE gene expression induced by IL-17, IL-22, or IFN-γ with various gene sets using gene set enrichment analysis (GSEA) (Figure 3A). The phenotype induced by IL-17 on RHE (as defined by the FCH between IL17 treated vs. control), was strongly enriched of genes in the psoriasis transcriptomes, and, to a lesser strength, with additive and synergistic TNF-α/IL-17 KC genes. RHE profile response to IL-22 or IFN-γ showed lower normalized enrichment scores (NESs), compared to IL-17 stimulation.

In order to determine if this IL-17 response is a reasonable model for the in vivo role of IL-17 in psoriasis, we compared the transcriptome of RHE treated with IL-17 to a previously published MAD-3 psoriasis transcriptome [40]. As shown in Figure 3B, half of the genes induced by IL-17 in RHE are also included in the psoriasis transcriptome, indicating that the response of RHE to IL-17 may reflect gene activation common in psoriasis lesion.

We next examined to what extent IL-17 blockade suppressed the genes highly induced by IL-17 in RHE. We hypothesized that if the genes regulated by IL-17 in the RHE model are relevant to psoriasis, then there should be a corresponding reduction in the same genes after therapeutic blockade of IL-17. Therefore, we compared the transcriptome of IL-17-treated RHE with the transcriptome of lesional skin two weeks after treatment with ixekizumab, an anti-IL-17 antibody [29] (Figure 3B). We have previously defined a “residual disease genomic profile,” which included the genes that do not improve by at least 75% compared to NL levels by the end of successful treatment [40]. In the recent ixekizumab study, 72.5% of the 95 genes in the IL-17 treated RHE model that were also part of the MAD-3 psoriasis transcriptome improved by over 75% at two weeks post-treatment compared with 68% of all psoriasis genes (Figure 3C) [41]. For comparison, only 31% of either the RHE genes or the psoriasis genes had recovered with etanercept at the same time point. There was a 3.45 FCH (1,79 log2) average expression of these RHE genes towards recovery with ixekizumab at two weeks, compared with 1.62 FCH (0.70 log2) for etanercept and no change (1.01 FCH, 0.01 log2) with placebo (Figure 3D). Overall, these results suggest that IL-17-induced genes in the RHE model coincide with genes that are suppressed *in vivo* by blocking IL-17 signaling with a neutralizing antibody to IL-17A.

## Discussion

Previous experiments have shown a limited number of genes induced by IL-17 in KCs despite ubiquitous epidermal expression of the IL-17 receptor [10], [12], [42]. In this study, we found that the distinct expression pattern of C/EBPβ, a crucial IL-17-related transcriptional factor, in the upper spinous-granular layers composed of more differentiated keratinocytes, may explain why undifferentiated, monolayer KCs respond less to IL-17 stimulation. Thus, in order to more fully investigate the genomic effects of IL-17 on KCs, we used a full-thickness skin model that more closely mimicked epidermal architecture and KC differentiation process. Using RHE, we identified a much larger number of genes induced by IL-17.

IL-17 elicits its pro-inflammatory effects in RHE, stimulating the expression of several genes including IL23A, IL1β, and IL36B (IL1F8) that have been implicated in psoriasis pathogenesis and found to be over-expressed in psoriatic lesional skin. The production of inflammatory cytokines by epidermal KCs likely perpetuates and sustains skin inflammation driven by T cells, especially given the essential roles of IL-23 and IL-1β in stimulating IL-17 production. Moreover, as key-regulator of innate immunity, IL-17 was shown to modulate antimicrobial peptides (AMPs) such as S100A7A, DEFB4, RNASE7, and Serpins A1, B3, and B4. Johnston *et al.* [43], demonstrated that IL-1F8 significantly induced several AMPs in an RHE model, including LCN2, defensins, HBD-2 and HBD-3, CAMP, elafin, serpinB1, and IL-8, and thus, the IL-1F8 expression induced by IL-17 stimulation may lead to a feed-forward loop amplifying AMP expression, which represents a distinct feature of lesional psoriatic skin.

Many abnormalities in keratinocyte differentiation are highly evident in psoriasis, including the expansion of the spinous-granular layer of the epidermis and alterations in terminal differentiation of KCs leading to a defective epidermal barrier with increased transepidermal water loss. Expression of the IL-17-specific transcription factor, C/EBPβ, in the spinous-granular layer may implicate IL-17 in these processes. Along these lines, Rizzo *et al.* [44], showed that IL-17A, like IL-22, was a downstream mediator of the changes induced by IL-23 injection in murine skin, and that both of these Th17 cytokines are necessary to produce IL-23–mediated psoriasis-like skin pathology. Indeed, the blockade of IL-17 or IL-22 in this model inhibits epidermal hyperplasia, indicating that either IL-17 or IL-22 can increase keratinocyte proliferation. Accordingly, in RHE, IL-17 induced IL19, a pro-proliferative cytokine belonging to IL-10 family, that is overexpressed in lesional psoriatic skin and has been implicated in epidermal hyperplasia [45]. Moreover, IL-17 induced several genes involved in terminal differentiation, including S100 proteins, S100A12 and S100A7A, and transglutaminases, TGM1 and TGM3, suggesting a role for IL-17 in this process. Additionally, the up-regulation of cell cycle-related genes such as CCNE1, CDCA5, and CDCA25A, suggests a direct contribution of IL-17 to epidermal KC proliferation. Therefore, the results of the current study suggest that IL-17 may have a much broader role than previously thought in stimulating the epidermal changes seen in psoriasis.

We defined the *in vivo* correlations of the novel gene set induced by IL-17 in RHE by analyzing the results of a recent clinical trial in psoriasis patients with a potent IL-17A antagonist, ixekizumab. IL-17 blockade is highly effective in reversing psoriasis, impressively resolving clinical, histological, and genomic facets of the disease [29]. Our results suggest that the genes regulated by IL-17 in RHE are likely relevant to the effects of IL-17 in psoriasis, since ixekizumab strongly suppressed these “RHE” genes in psoriasis patients treated in vivo with this IL-17 antagonist.

Psoriasis is thought to develop and be maintained as a result of cooperative efforts of several T cell cytokines in addition to IL-17, namely IFN-γ and IL-22, which augments cellular recruitment through chemokine induction and stimulates epidermal hyperplasia, respectively. However, the complete reversal of the psoriasis phenotype by ixekizumab and other IL-17 antagonists [29], suggests that this model may need to be revised to account for the centrality of IL-17 in driving the inflammatory circuits in psoriasis. While epidermal acanthosis is not highly evident in histological sections of IL-17 treated RHE, many cyclins associated with increased cell proliferation are elevated in IL-17 treated cultures and growth factors associated with keratinocyte proliferation, e.g. IL-19, TGFα, HBEGF, are increased. Thus, IL-17 might contribute to epidermal hyperplasia in vivo through indirect effects on keratinocytes. In addition, leukocytes that are recruited by cytokines induced in keratinocytes by IL-17 could also be important in producing epidermal hyperplasia in vivo, either through elaborated interleukins or by migration through the epithelium [38], [43], [45]–[47]. Furthermore, the induction of genes such as CCL20 (a chemoattractant for CCR6-bearing cells, such as Th17 cells), IL1β, and IL23 (both involved in the Th17 differentiation process) suggests that IL-17 may create feed-forward loops that perpetuate Th17-polarized inflammatory processes. Along these lines, the induction of anti-microbial peptides may also sustain inflammation as IL-17-induced cathelicidin (LL-37) has been shown to complex with nucleic acids to activate DC stimulation of broader and more non-specific T cell activation [48], [49]. Overall, these data strongly implicate IL-17 as a central player in the pathogenic mechanism underlying the pathogenesis of psoriasis. Additionally, this study provides insight into the IL-17-induced expression of inflammatory genes belonging to damage-associated molecular pattern molecules (DAMP) or inflammasome that could be potentially identified as novel therapeutic targets. Indeed, the blockade of these IL-17-downstream genes may represent a further step in the therapeutic strategy to be more selective in inhibiting the inflammatory cascade.

IL-17 is most closely associated with the pathogenesis of psoriasis, but it could also contribute to other inflammatory skin disease, e.g., atopic dermatitis (AD). Significant IL-17 expression is seen in skin lesions of intrinsic AD (low IgE sub-type) and increased expression of several S100A genes (S100A7, S100A8, S100A9, S100A12), that are synergistically regulated by IL-17 and IL-22 is also detected [50].

Additionally, relative to normal skin, AD lesions have increased expression of AMPs (LCN, β-defensins, etc.) that are IL-17-regulated, although the measured levels are much lower than seen in psoriasis. Since the range of products regulated by IL-17 in AD are largely overexpressed in the upper spinous and granular layers of AD epidermis, the selective expression of C/EBPβ in more differentiated KCs is also likely to be relevant to AD pathogenesis.

## Materials and Methods

### Skin samples

Skin punch biopsies (6 mm diameter) were obtained from normal volunteers and patients with moderate-to-severe chronic plaque psoriasis under a Rockefeller University Institutional Review Board-approved protocol. Written, informed consent was obtained from all subjects, and adhered to the Declaration of Helsinki Principles.

The biopsy specimens were frozen in OTC (Sakura, Torrance, CA, U.S.A.) and stored at −80°C for immunohistochemistry and immunofluorescence.

### Immunohistochemistry and Immunofluorescence

Frozen tissue sections of psoriatic lesional, non-lesional, and normal skin were stained using standard procedures for both IHC and IF as previously described [51].

#### Immunohistochemistry

Staining was performed with antibody targeting C/EBPβ, LCN2, HBD2, STAT1, RFX5 (Table S2). According to the primary antibody species, either biotin-labeled horse anti-mouse antibodies (Vector Laboratories, Burlingame, CA, U.S.A.) or biotin-labeled rabbit anti-goat antibodies (Vector Laboratories, Burlingame, CA, U.S.A.) were amplified with avidin-biotin complex (Vector Laboratories) and developed using chromogen 3-amino-9-ethylcarbazole (Sigma Aldrich, St Louis, MO, U.S.A.). For the staining in the RHE, a black line denotes the dermoepidermal junction. Appropriate negative controls were used.

#### Immunofluorescence

Frozen skin sections from non-lesional and lesional psoriasis patients were fixed with acetone and blocked in 10% normal chicken serum (Vector Laboratories) for 30 minutes. Primary antibodies for C/EBPβ and HBD2 (Table S2) were incubated overnight at 4°C and amplified with the appropriate secondary antibody goat anti-mouse IgG1 conjugated to Alexa Fluor 488 and chicken anti-goat Alexa Flour 594 (Invitrogen, Eugene, OR) respectively, for 30 minutes.

IF images were acquired using the appropriate filters of a Zeiss Axioplan 2 wide-field fluorescence microscope (Thornwood, NY) with a Plan Neofluar 20×0.7 numerical aperture lens and a Hamamatsu Orca Er-cooled charge-coupled device camera (Bridgewater, NJ), controlled by the METAVUE software (MDS Analytical Technologies, Downington, PA). Images in each figure are presented both as single-color stains (green and red) located above the merged image, so that localization of two markers on similar or different cells can be appreciated. Cells that co-express the two markers in a similar location are yellow in color. A white line denotes the dermoepidermal junction. Dermal collagen fibers gave green autofluorescence, and antibodies conjugated with a fluorochrome often gave background epidermal fluorescence.

### Cell cultures

We cultivated primary human skin fibroblast lines (HF40 and HFF-1) (n = 2 each) that were obtained from the American Type Culture Collection (ATCC, Manassas, VA) and cultivated in Dulbecco's minimum essential medium supplemented with 10% fetal calf serum and, when confluent, medium was supplemented with or without recombinant human (rh)-IL-17 (R&D System, Minneapolis, MN) of 200 ng ml^−1^ (same IL-17 source and concentration used in prior experiments with human keratinocytes) [10], [12]. After 24-hour incubation, fibroblasts were harvested for further analyses.

We also cultivated NHEKs obtained from PromoCell, in the Keratinocyte Growth Medium 2 supplemented with 0.004 ml/ml BPE, 0.125 ng/ml EGF, 5 ug/ml Insulin, 0.33 ug/ml Hydrocortisone, 0.39 ug/ml Epinephrine, 10 ug/ml Transferrin, and 0.06 mM Ca++ (all items purchased from PromoCell GmbH, Heidelberg, Germany). The experiment was performed in triplicate.

Once 70–80% confluent, the medium was changed with full media containing 0.06 mM Ca++, 1.2 mM Ca++, or 1.2 mM Ca++ plus 2.0% FBS, for 24 and 48 hours before harvesting for other analyses.

### Human full-thickness skin model (RHE)

Full-thickness human skin models (MatTek Corp., Ashland, MA, U.S.A.) (n = 4) were incubated in assay media (MatTek Corp.) supplemented with or without rh-IL-17 (R&D Systems, Minneapolis, MN, U.S.A.) 200 ng mL^−1^, rh-IL-22 (Peprotech Inc., Rocky Hill, NJ, U.S.A.) 200 ng mL^−1^ 200, or rh-IFN-γ (R&D Systems, Minneapolis, MN, U.S.A.) 20 ng mL^−1^, for 2 days. On day 2, the skin models were harvested for microarray analyses. The same concentrations used for treating *in vitro* monolayer keratinocytes were applied for RHE, as they were proved effective in gene modulation as previously described by our group [12].

### Gene array

RNA was extracted from RHE using the RNeasy Mini Kit (Qiagen, Valencia, CA, U.S.A.) and on-column DNAse digestion (RNAse-free DNAse Set, Qiagen), for either gene array or RT-PCR procedures.

For each Affymetrix genechip, 4 µg total RNA was reverse transcribed, amplified, and labeled as described previously using BioArray High Yield RNA Transcription Labeling Kit (Enzo Biochem Inc., Farmingdale, NY, U.S.A.) [52]. Fifteen micrograms of the biotinylated cRNA were then hybridized to Affymetrix Human Genome U133A Plus 2.0 Array (Affymetrix, Santa Clara, CA, U.S.A.). The chips were washed, stained with streptavidin-phycoerythin, and scanned with a Hewlett-Packard HP GeneArray Scanner (Hewlett-Packard, Palo Alto, CA, U.S.A.).

### Reverse transcriptase–polymerase chain reaction

To perform RT-PCR, the RNA extracted from fibroblasts and RHE model was processed using EZ PCR core reagents, primers, and probes (Applied Biosystems, Foster City, CA) as previously published [53], whilst total RNA was extracted from NHEKs using RNeasy micro kit (QIAGEN Inc, Valencia, CA).

The following sequences of primers and probes were used in this study: IL-19 (Hs00604655_m1), C/EBPβ (Hs 00270923_s1), S100A7A (Hs00752780_s1), IL-1F8 (Hs00758166_m1), IL-23A (Hs00372324_m1). The data were analyzed by the Applied Biosystems PRISM 7700 software (Sequence Detection Systems, ver. 1.7) and normalized to human acidic ribosomal protein (hARP) housekeeping gene (primer sequences Forward: CGCTGCTGAACATGCTCAA, Reverse: TGTCGAACACCTGCTGGATG, Probe: 6-FAM-TCCCCCTTCTCCTTTGGGCTGG- TAMRA).

### Statistical analysis

Preprocessing and statistical analysis was conducted in R (http://www.rproject.org/).

Microarray GeneChip CEL data files were scanned for spatial artifacts using Harshlight package (http://asterion.rockefeller.edu/Harshlight/index2.html) [54]. Expression values were pre-processed using GCRMA algorithm [55]. ArrayQualityControl was used for standard QC.

Probes with at least one sample showing expression values greater than 3 and SD >0.1 were selected for further analyses.

Significance of cytokine induction in RHE gene expression was assessed by using a moderated paired t-test, comparing untreated RHE with cytokine-treated RHE. Subsequently, p-values were adjusted using Benjamini-Hochberg correction, which controls the FDR.

Genes were considered DEGs if FDR>0.1 and FCH>1.5, accordingly to the same cut-offs used for gene array data derived from previous IL-17/keratinocyte experiments [12]. IL-17 effects on gene expression was evaluated in RHE and compared with keratinocyte [12] and fibroblast responses to IL-17 exposure. These data are available in the Gene Expression Omnibus (GEO) repository under accession No. GSE52361.

To assess the biological meaning of IL-17-induced RHE genes in psoriasis, a comparison with the MAD-3 psoriasis transcriptome (defined by a meta-analysis of 3 published transcriptomes) [40] was performed. To evaluate the effect of antipsoriatic therapies on RHE genes induced by IL-17, previously published genomic responses to different therapeutic agents, namely ixekizumab [29] and etanercept [27], were analyzed. Comparisons included only IL17-induced RHE probe sets in hgu133a2 chips, since the gene array data for both treatments were performed using the same kind of chips. For those probes, whose expression differed after 2-week treatment compared to baseline, the mean variation was calculated. Similarly, the improvement at 2 weeks of treatment under both treatments was summarized, meaning as improvement the treatment effect divided by the level of disregulation at baseline, which was measured as LS vs NL differences estimated through the MAD-3 transcriptome.

Gene Set Enrichment Analysis (GSEA) was used to evaluate the enrichment of various gene sets in the gene response profile of the RHE treated with IL-17, IL-22, or IFN-γ [56].

## Supporting Information

Figure S1 **Co-localization of C/EBPβ and HBD2 in psoriatic skin.** Immunofluorescence staining for IL-17 transcription factor, C/EBPβ (green), and downstream target, human β-defensin 2 (HBD2, red), in non-lesional (left) or lesional (right) psoriatic skin. Both proteins are localized to the spinous-granular layer, which is especially evident in non-lesional skin.
(TIFF)

Figure S2 **Distinct staining patterns of epidermal transcription factors.** Immunohistochemistry for transcription factors, RFX-5 and STAT1, in normal, non-lesional, and lesional psoriatic skin. RFX5 stains basal keratinocytes, while STAT1 has pan-epidermal expression.
(TIFF)

Figure S3 **C/EBPβ gene expression in normal skin.** C/EBPβ gene expression in reticular dermis, basal epidermis, and suprabasal epidermis, obtained by laser capture microdissection of normal human skin (Gulati et al., 2013).
(TIFF)

Figure S4 **Expression levels of terminal differentiation genes in monolayer** ***in vitro*** **NHEKs.** Increased expression of terminal differentiation genes was detected in high-calcium-treated NHEKs: (A) C/EBPβ, (B) FLG2, (C) TGM1, (D) IVL. Differences with low-calcium condition were statistically significant. Gene expression was normalized by hARP.
(TIFF)

Figure S5 **C/EBPβ expression in RHE model.** Differential C/EBPβ expression in untreated RHE (**A**) versus IL-17-treated RHE (**B**). Black line shapes the dermoepidermal junction, while arrows mark the light staining displayed in differentiated keratinocytes localized in the upper layers of the epidermis. (**C**) Detection of C/EBPβ gene expression in untreated and IL-17-treated-RHE by PCR.
(TIFF)

Figure S6 **IL-17, IL-22, and IFN-γ induce unique RHE gene signatures.** (**A**) Venn diagram illustrates the number of probe-sets regulated in RHE by IL-17, IL-22, or IFN-γ treatment. (**B**) Scatter plots comparing the genes induced in RHE versus *in vitro* cultured KCs showing that IL-17 induced a large number of genes only in RHE, while IFN-γ induced a larger number of genes in monolayer KCs compared to RHE. (**C**) Gene expression levels of some IL-17 signature genes detected by RT-PCR, confirmatory of the gene array results.
(TIFF)

Table S1 **Differentially expressed genes in IL-17-treated keratinocytes and/or RHE and/or fibroblasts.**
(PDF)

Table S2 **Antibodies used for immunohistochemistry and immunofluorescence.**
(DOCX)
