# Supplementary figures and images for: IL-17 Induces an Expanded Range of Downstream Genes in Reconstituted Human Epidermis Model
Source: PLoS One. 2014 Feb 28;9(2):e90284. doi: 10.1371/journal.pone.0090284 (PMC3938679; doi:10.1371/journal.pone.0090284)

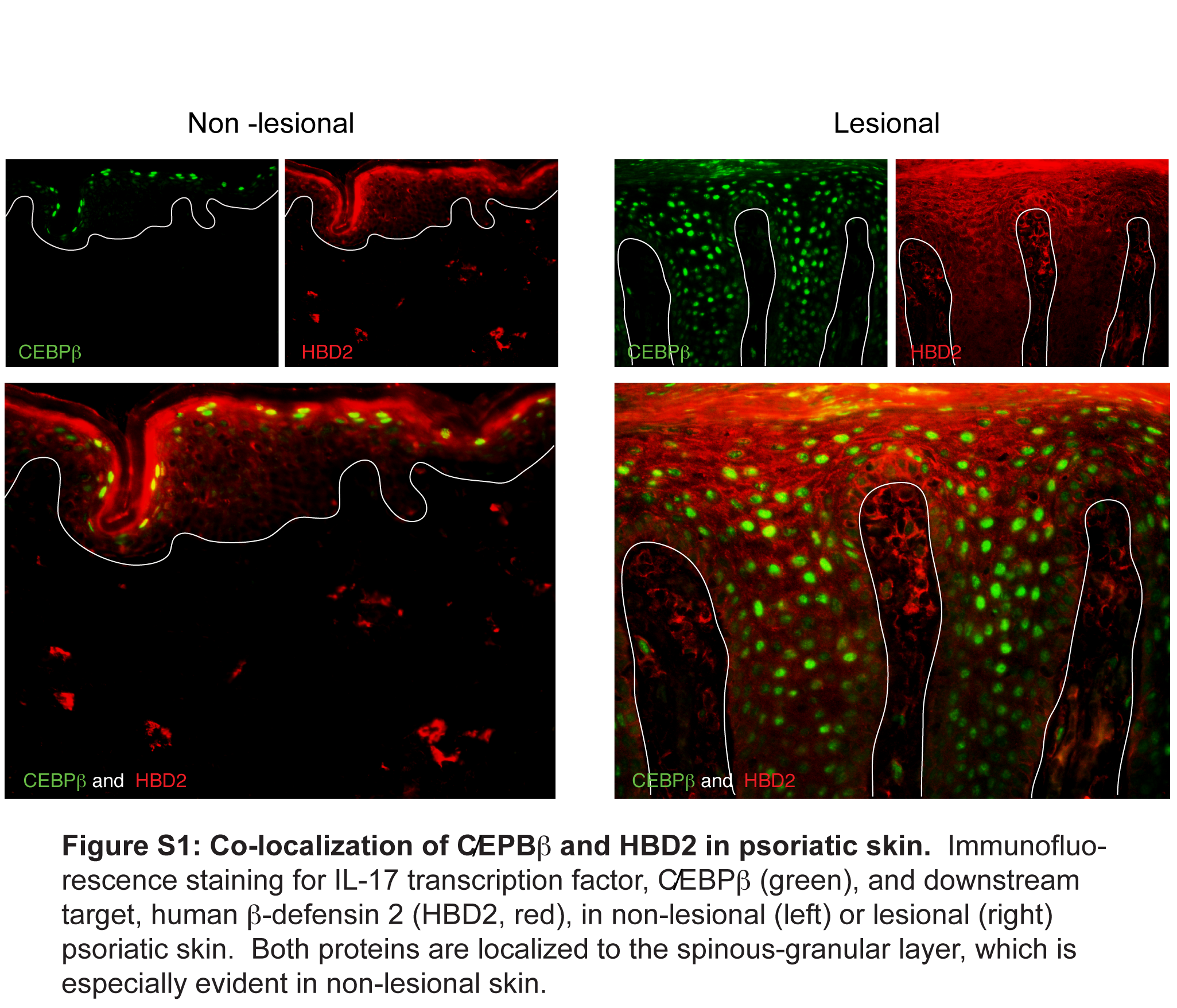

Supplement: Figure S1 — Co-localization of C/EBPβ and HBD2 in psoriatic skin. Immunofluorescence staining for IL-17 transcription factor, C/EBPβ (green), and downstream target, human β-defensin 2 (HBD2, red), in non-lesional (left) or lesional (right) psoriatic skin. Both proteins are localized to the spinous-granular layer, which is especially evident in non-lesional skin. (TIFF) [file pone.0090284.s001.tif]

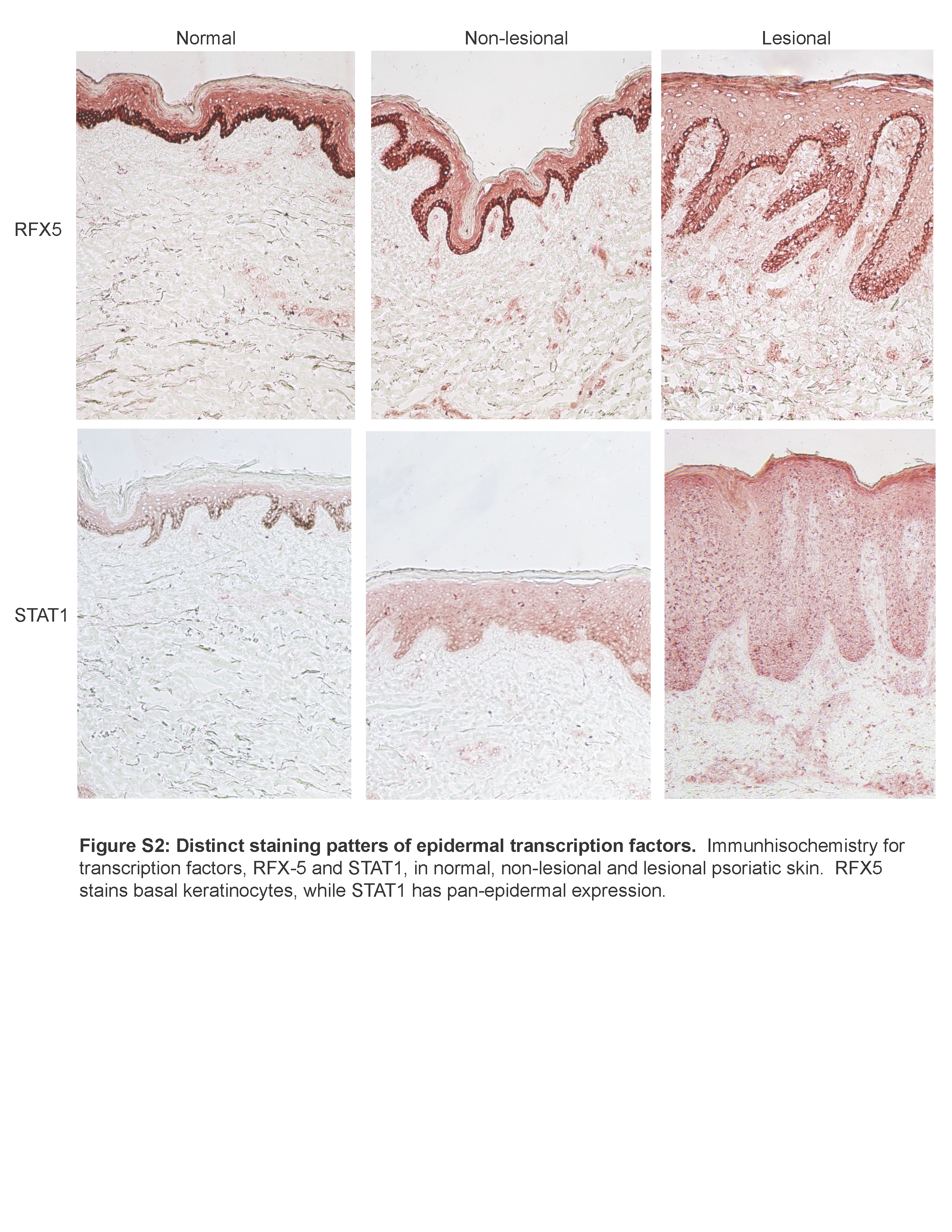

Supplement: Figure S2 — Distinct staining patterns of epidermal transcription factors. Immunohistochemistry for transcription factors, RFX-5 and STAT1, in normal, non-lesional, and lesional psoriatic skin. RFX5 stains basal keratinocytes, while STAT1 has pan-epidermal expression. (TIFF) [file pone.0090284.s002.tif]

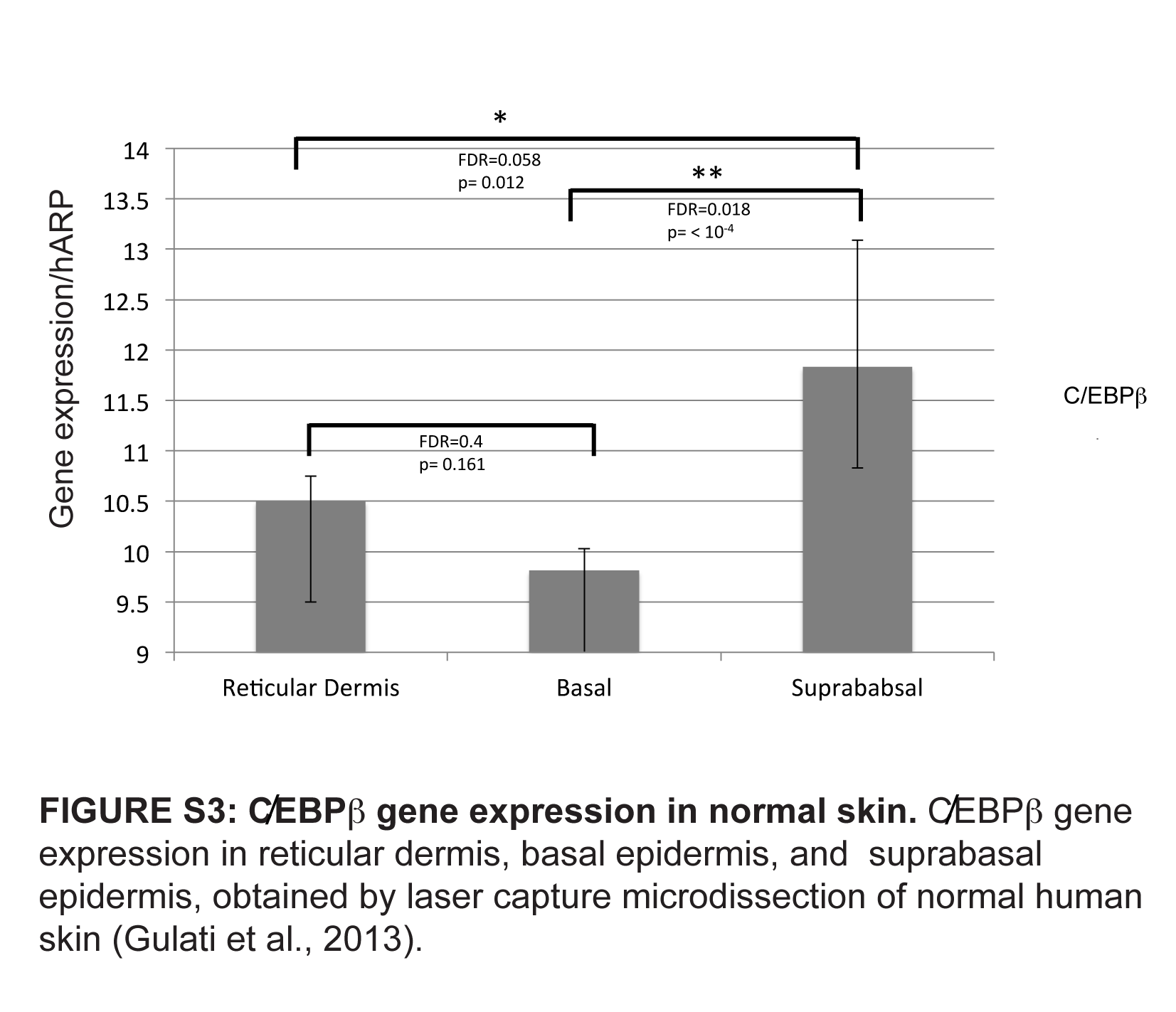

Supplement: Figure S3 — C/EBPβ gene expression in normal skin. C/EBPβ gene expression in reticular dermis, basal epidermis, and suprabasal epidermis, obtained by laser capture microdissection of normal human skin (Gulati et al., 2013). (TIFF) [file pone.0090284.s003.tif]

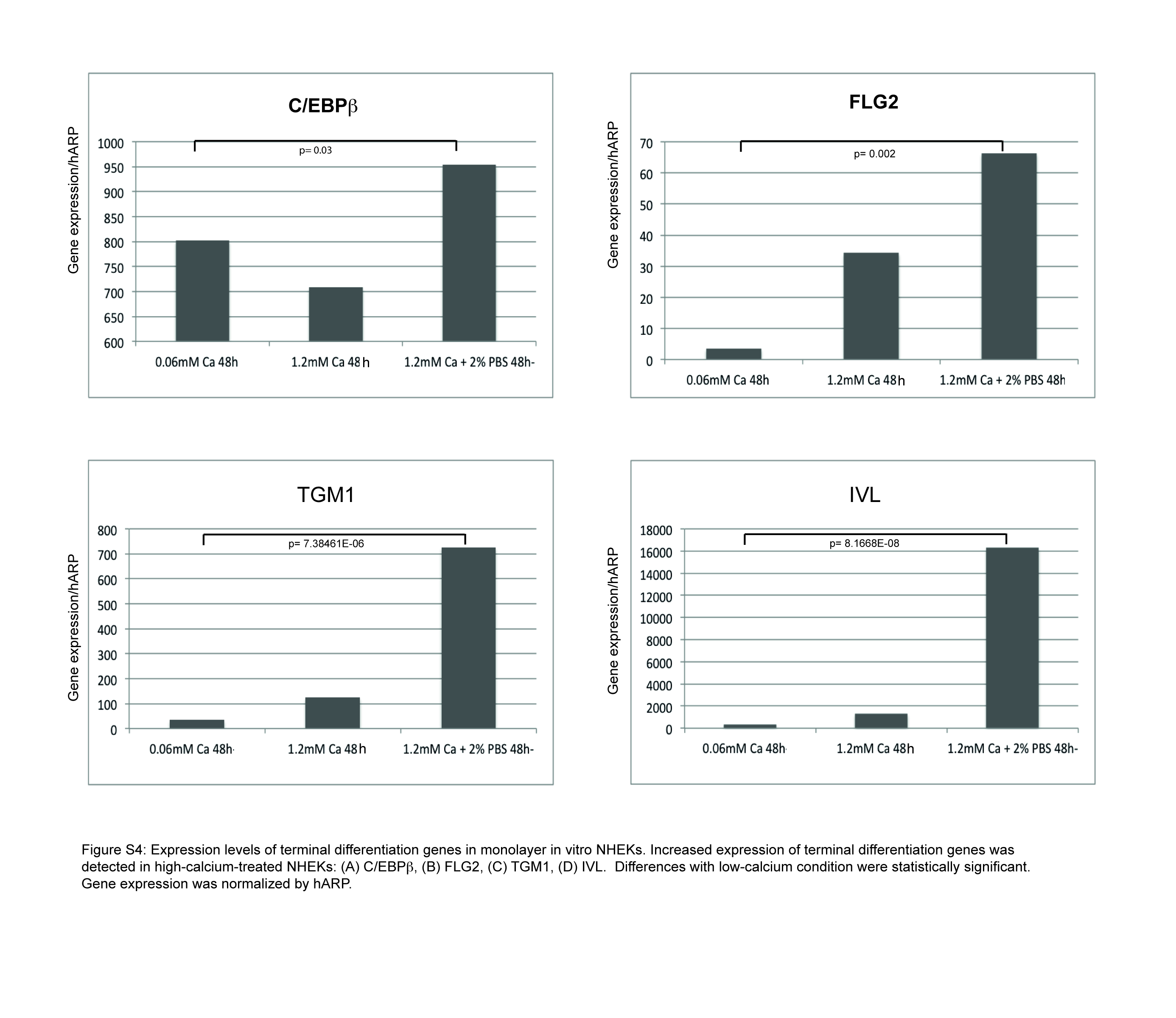

Supplement: Figure S4 — Expression levels of terminal differentiation genes in monolayer in vitro NHEKs. Increased expression of terminal differentiation genes was detected in high-calcium-treated NHEKs: (A) C/EBPβ, (B) FLG2, (C) TGM1, (D) IVL. Differences with low-calcium condition were statistically significant. Gene expression was normalized by hARP. (TIFF) [file pone.0090284.s004.tif]

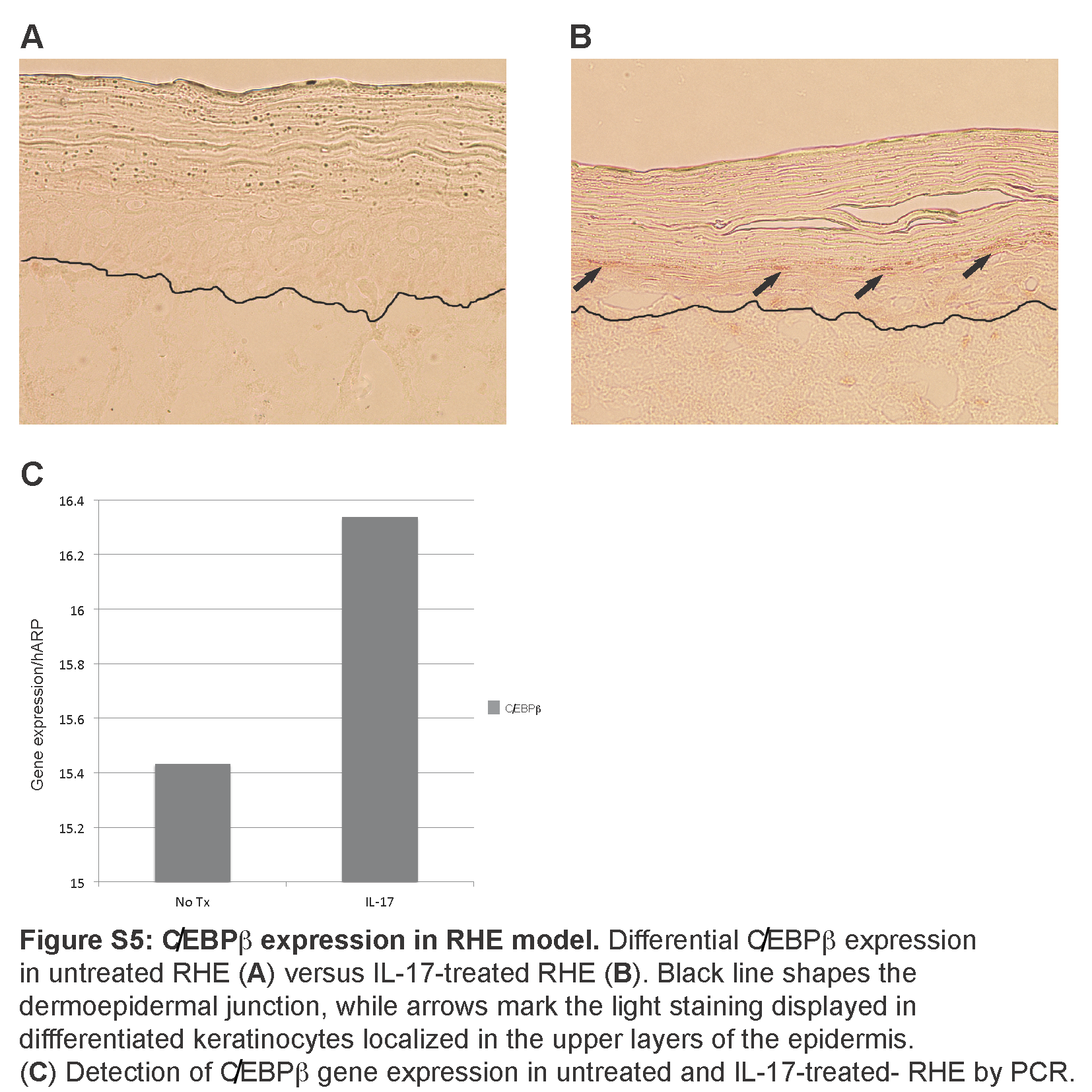

Supplement: Figure S5 — C/EBPβ expression in RHE model. Differential C/EBPβ expression in untreated RHE (A) versus IL-17-treated RHE (B). Black line shapes the dermoepidermal junction, while arrows mark the light staining displayed in differentiated keratinocytes localized in the upper layers of the epidermis. (C) Detection of C/EBPβ gene expression in untreated and IL-17-treated-RHE by PCR. (TIFF) [file pone.0090284.s005.tif]

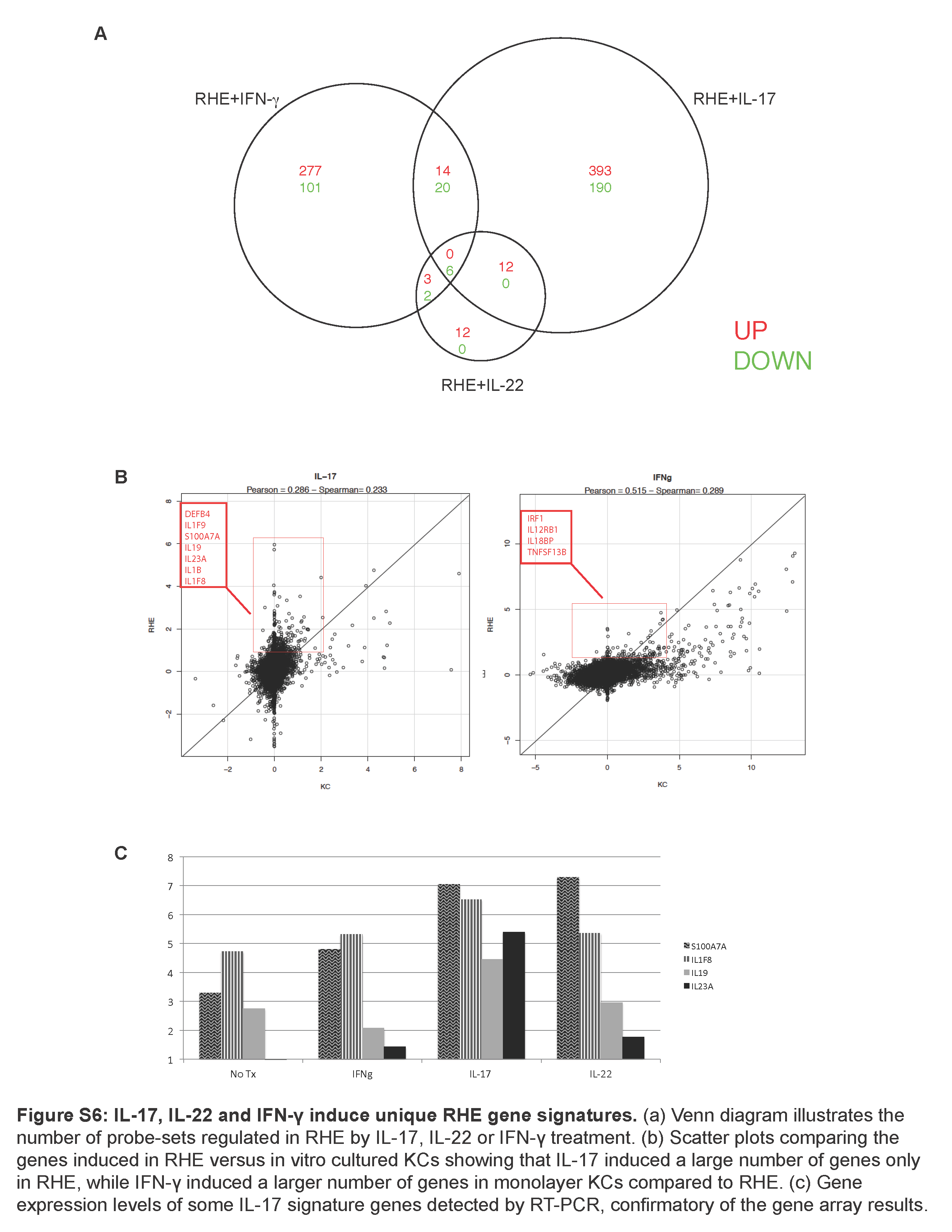

Supplement: Figure S6 — IL-17, IL-22, and IFN-γ induce unique RHE gene signatures. (A) Venn diagram illustrates the number of probe-sets regulated in RHE by IL-17, IL-22, or IFN-γ treatment. (B) Scatter plots comparing the genes induced in RHE versus in vitro cultured KCs showing that IL-17 induced a large number of genes only in RHE, while IFN-γ induced a larger number of genes in monolayer KCs compared to RHE. (C) Gene expression levels of some IL-17 signature genes detected by RT-PCR, confirmatory of the gene array results. (TIFF) [file pone.0090284.s006.tif]
