# Supplementary material for: IL-17 Induces an Expanded Range of Downstream Genes in Reconstituted Human Epidermis Model
Source: PLoS One. 2014 Feb 28;9(2):e90284. doi: 10.1371/journal.pone.0090284 (PMC3938679; doi:10.1371/journal.pone.0090284)
Supplement: Table S1 — Differentially expressed genes in IL-17-treated keratinocytes and/or RHE and/or fibroblasts. (PDF) [file pone.0090284.s007.pdf]

Table S1: Differentially expressed genes in IL-17-treated keratinocytes and/or RHE and/or Fibroblasts

SYMBOL	Description	FCH_IL17_RHE	lgFCH_IL17_RHE	p_lgFCH_IL17_RHE	FDR_lgFCH_IL17_RHE	Status_IL17_RHE	FCH_IL17_KC	lgFCH_IL17_KC	p_IL17_KC	FDR_IL17_KC	Status_IL17_KC	FCH_IL17_Fib	lgFCH_IL17_Fib	Status_IL17_Fib
ZC3H12A	zinc finger CCCH-type containing 12A	5,69	2,51	0,00	0,00	1	19,07	4,25	0,00	0,00	1	11,71	3,55	1
IL1B	interleukin 1, beta	3,94	1,98	0,00	0,01	1	2,64	1,40	0,00	0,03	1	6,90	2,79	1
IL1B	interleukin 1, beta	3,89	1,96	0,00	0,04	1	2,73	1,45	0,00	0,01	1	6,42	2,68	1
SPRR2C	small proline-rich protein 2C (pseudogene)	27,07	4,76	0,00	0,00	1	19,10	4,26	0,00	0,00	1	NA	NA	0
DEFB4A	defensin, beta 4A	24,37	4,61	0,00	0,01	1	238,55	7,90	0,00	0,00	1	NA	NA	0
IL1F9	interleukin 1 family, member 9	16,37	4,03	0,00	0,00	1	15,06	3,91	0,00	0,00	1	NA	NA	0
SERPINB4	serpin peptidase inhibitor, clade B (ovalbumin), member 4	7,03	2,81	0,00	0,00	1	27,53	4,78	0,00	0,00	1	NA	NA	0
S100A12	S100 calcium binding protein A12	4,85	2,28	0,00	0,01	1	30,71	4,94	0,00	0,02	1	NA	NA	0
NDRG2	NDRG family member 2	2,86	1,51	0,00	0,03	1	5,53	2,47	0,00	0,03	1	NA	NA	0
NA	NA	2,70	1,43	0,00	0,00	1	2,09	1,06	0,00	0,16	1	NA	NA	0
SLC6A14	solute carrier family 6 (amino acid transporter), member 14	2,29	1,19	0,00	0,07	1	8,98	3,17	0,00	0,01	1	NA	NA	0
NOD2	nucleotide-binding oligomerization domain containing 2	2,16	1,11	0,00	0,00	1	5,25	2,39	0,00	0,01	1	NA	NA	0
NAV3	neuron navigator 3	2,00	1,00	0,00	0,06	1	1,85	0,89	0,00	0,10	1	NA	NA	0
SLC11A2	solute carrier family 11 (proton-coupled divalent metal ion transporters), member 2	1,86	0,89	0,00	0,05	1	2,17	1,12	0,00	0,00	1	NA	NA	0
ATP1B1	ATPase, Na+/K+ transporting, beta 1 polypeptide	1,74	0,80	0,00	0,09	1	2,18	1,12	0,00	0,12	1	NA	NA	0
CYB5R2	cytochrome b5 reductase 2	1,67	0,74	0,00	0,05	1	1,73	0,79	0,00	0,00	1	NA	NA	0
CHAC1	ChaC, cation transport regulator homolog 1 (E. coli)	11,35	3,50	0,00	0,01	1	1,16	0,22	0,37	1,00	0	1,64	0,71	1
LIF	leukemia inhibitory factor (cholinergic differentiation factor)	3,47	1,79	0,00	0,02	1	-1,01	-0,01	NA	NA	0	2,85	1,51	1
SLCO4A1	solute carrier organic anion transporter family, member 4A1	2,98	1,58	0,00	0,01	1	1,66	0,74	0,09	1,00	0	1,74	0,80	1
IL1A	interleukin 1, alpha	2,85	1,51	0,00	0,04	1	1,39	0,48	0,00	0,24	0	8,76	3,13	1
CSF3	colony stimulating factor 3 (granulocyte)	2,74	1,45	0,00	0,03	1	1,03	0,04	NA	NA	0	213,26	7,74	1
RNASE1	ribonuclease, RNase A family, 1 (pancreatic)	62,15	5,96	0,00	0,00	1	-1,01	-0,01	NA	NA	0	NA	NA	0
NDP	Norrie disease (pseudoglioma)	53,05	5,73	0,00	0,00	1	-1,01	-0,01	NA	NA	0	NA	NA	0
PLIN5	perilipin 5	40,35	5,33	0,00	0,00	1	NA	NA	NA	NA	0	NA	NA	0
NA	NA	24,53	4,62	0,00	0,00	1	NA	NA	NA	NA	0	NA	NA	0
SOST	sclerostin	22,96	4,52	0,00	0,00	1	NA	NA	NA	NA	0	NA	NA	0
CLDN17	claudin 17	21,36	4,42	0,00	0,01	1	3,99	2,00	0,00	0,33	0	NA	NA	0
PIPOX	pipecolic acid oxidase	16,69	4,06	0,00	0,00	1	-1,02	-0,03	NA	NA	0	NA	NA	0
HYAL4	hyaluronoglucosaminidase 4	13,43	3,75	0,00	0,03	1	-1,00	0,00	NA	NA	0	NA	NA	0
ACPP	acid phosphatase, prostate	12,98	3,70	0,00	0,01	1	NA	NA	NA	NA	0	NA	NA	0
IL19	interleukin 19	12,82	3,68	0,00	0,01	1	-1,00	-0,01	NA	NA	0	NA	NA	0
S100A7A	S100 calcium binding protein A7A	12,70	3,67	0,00	0,01	1	NA	NA	NA	NA	0	NA	NA	0
ACPP	acid phosphatase, prostate	12,24	3,61	0,00	0,01	1	-1,01	-0,01	NA	NA	0	NA	NA	0
IL23A	interleukin 23, alpha subunit p19	11,53	3,53	0,00	0,00	1	2,14	1,10	0,07	1,00	0	NA	NA	0
LRG1	leucine-rich alpha-2-glycoprotein 1	10,04	3,33	0,00	0,03	1	NA	NA	NA	NA	0	NA	NA	0
AGPAT9	1-acylglycerol-3-phosphate O-acyltransferase 9	8,98	3,17	0,00	0,00	1	NA	NA	NA	NA	0	NA	NA	0
NA	NA	8,79	3,14	0,00	0,01	1	NA	NA	NA	NA	0	NA	NA	0
KRT24	keratin 24	8,42	3,07	0,00	0,06	1	1,09	0,13	0,62	1,00	0	NA	NA	0
DSCAM	Down syndrome cell adhesion molecule	8,25	3,04	0,00	0,00	1	NA	NA	NA	NA	0	NA	NA	0
SLC26A9	solute carrier family 26, member 9	8,13	3,02	0,00	0,01	1	NA	NA	NA	NA	0	NA	NA	0
XDH	xanthine dehydrogenase	8,03	3,01	0,00	0,01	1	-1,33	-0,41	0,47	1,00	0	NA	NA	0
PRR9	proline rich 9	7,97	3,00	0,00	0,07	1	NA	NA	NA	NA	0	NA	NA	0
NA	NA	7,85	2,97	0,00	0,02	1	NA	NA	NA	NA	0	NA	NA	0
FBXO45	F-box protein 45	7,60	2,93	0,00	0,01	1	NA	NA	NA	NA	0	NA	NA	0
NA	NA	7,23	2,85	0,00	0,06	1	NA	NA	NA	NA	0	NA	NA	0
FCHSD1	FCH and double SH3 domains 1	7,19	2,85	0,00	0,02	1	NA	NA	NA	NA	0	NA	NA	0
TGM3	transglutaminase 3 (E polypeptide, protein-glutamine-gamma-glutamyltransferase)	7,15	2,84	0,00	0,01	1	-1,00	0,00	NA	NA	0	NA	NA	0
TRPM8	transient receptor potential cation channel, subfamily M, member 8	6,86	2,78	0,00	0,09	1	NA	NA	NA	NA	0	NA	NA	0
FCHSD1	FCH and double SH3 domains 1	6,76	2,76	0,00	0,01	1	NA	NA	NA	NA	0	NA	NA	0
KIAA1239	KIAA1239	6,67	2,74	0,00	0,04	1	NA	NA	NA	NA	0	NA	NA	0
KRT75	keratin 75	6,63	2,73	0,00	0,00	1	1,54	0,62	0,03	1,00	0	NA	NA	0
VNN3	vanin 3	6,61	2,72	0,00	0,00	1	1,00	0,00	NA	NA	0	NA	NA	0
NA	NA	6,52	2,70	0,00	0,00	1	NA	NA	NA	NA	0	NA	NA	0
CHI3L2	chitinase 3-like 2	6,41	2,68	0,00	0,03	1	-1,00	0,00	NA	NA	0	NA	NA	0
PMFBP1	polyamine modulated factor 1 binding protein 1	6,21	2,64	0,00	0,01	1	-1,00	0,00	NA	NA	0	NA	NA	0
NA	NA	6,15	2,62	0,00	0,03	1	NA	NA	NA	NA	0	NA	NA	0
TCP11L2	t-complex 11 (mouse)-like 2	6,06	2,60	0,00	0,03	1	NA	NA	NA	NA	0	NA	NA	0
TREX2	three prime repair exonuclease 2	5,93	2,57	0,00	0,05	1	-1,00	0,00	NA	NA	0	NA	NA	0
ATP12A	ATPase, H+/K+ transporting, nongastric, alpha polypeptide	5,89	2,56	0,00	0,00	1	2,40	1,26	0,13	1,00	0	NA	NA	0
NA	NA	5,50	2,46	0,00	0,08	1	NA	NA	NA	NA	0	NA	NA	0
C4orf19	chromosome 4 open reading frame 19	5,42	2,44	0,00	0,04	1	-1,01	-0,01	NA	NA	0	NA	NA	0
NA	NA	5,36	2,42	0,00	0,06	1	NA	NA	NA	NA	0	NA	NA	0
HPSE	heparanase	5,23	2,39	0,00	0,02	1	NA	NA	NA	NA	0	NA	NA	0
SPRR4	small proline-rich protein 4	5,21	2,38	0,00	0,10	1	NA	NA	NA	NA	0	NA	NA	0
NCF2	neutrophil cytosolic factor 2	5,19	2,38	0,00	0,01	1	2,25	1,17	0,00	0,26	0	NA	NA	0
CDH26	cadherin 26	5,10	2,35	0,00	0,01	1	NA	NA	NA	NA	0	NA	NA	0
EXPH5	exophilin 5	5,07	2,34	0,00	0,01	1	1,06	0,09	0,88	1,00	0	NA	NA	0
TGFA	transforming growth factor, alpha	5,04	2,33	0,00	0,04	1	1,15	0,20	0,66	1,00	0	NA	NA	0
GM2A	GM2 ganglioside activator	4,98	2,32	0,00	0,01	1	2,21	1,15	0,26	1,00	0	NA	NA	0
NA	NA	4,92	2,30	0,00	0,01	1	NA	NA	NA	NA	0	NA	NA	0
C15orf62	chromosome 15 open reading frame 62	4,89	2,29	0,00	0,06	1	NA	NA	NA	NA	0	NA	NA	0
KLK13	kallikrein-related peptidase 13	4,75	2,25	0,00	0,10	1	1,03	0,04	0,77	1,00	0	NA	NA	0
SLC35E4	solute carrier family 35, member E4	4,72	2,24	0,00	0,02	1	NA	NA	NA	NA	0	NA	NA	0
CD177	CD177 molecule	4,71	2,23	0,00	0,00	1	-1,01	-0,01	NA	NA	0	NA	NA	0
NA	NA	4,69	2,23	0,00	0,10	1	-1,01	-0,01	NA	NA	0	NA	NA	0
NDRG2	NDRG family member 2	4,58	2,20	0,00	0,02	1	-1,00	-0,01	NA	NA	0	NA	NA	0
KLK13	kallikrein-related peptidase 13	4,57	2,19	0,00	0,08	1	-1,00	-0,01	NA	NA	0	NA	NA	0
ST8SIA4	ST8 alpha-N-acetyl-neuraminide alpha-2,8-sialyltransferase 4	4,48	2,16	0,00	0,03	1	NA	NA	NA	NA	0	NA	NA	0
NDRG2	NDRG family member 2	4,47	2,16	0,00	0,02	1	10,07	3,33	0,01	0,63	0	NA	NA	0
ADAP2	ArfGAP with dual PH domains 2	4,42	2,14	0,00	0,05	1	1,70	0,76	0,03	1,00	0	NA	NA	0
IL1F8	interleukin 1 family, member 8 (eta)	4,34	2,12	0,00	0,09	1	NA	NA	NA	NA	0	NA	NA	0
CEBPA	CCAAT/enhancer binding protein (C/EBP), alpha	4,28	2,10	0,00	0,01	1	1,15	0,20	0,67	1,00	0	NA	NA	0
ADAP2	ArfGAP with dual PH domains 2	4,26	2,09	0,00	0,10	1	NA	NA	NA	NA	0	NA	NA	0
SOX7	SRY (sex determining region Y)-box 7	4,22	2,08	0,00	0,02	1	NA	NA	NA	NA	0	NA	NA	0
NA	NA	4,21	2,07	0,00	0,02	1	NA	NA	NA	NA	0	NA	NA	0
ATP1B1	ATPase, Na+/K+ transporting, beta 1 polypeptide	4,17	2,06	0,00	0,10	1	2,37	1,25	0,01	0,74	0	NA	NA	0
TCP11L2	t-complex 11 (mouse)-like 2	4,13	2,05	0,00	0,08	1	NA	NA	NA	NA	0	NA	NA	0
HPSE	heparanase	4,09	2,03	0,00	0,02	1	1,57	0,65	0,02	0,94	0	NA	NA	0
ZDHHC21	zinc finger, DHHC-type containing 21	4,09	2,03	0,00	0,07	1	NA	NA	NA	NA	0	NA	NA	0
NA	NA	4,04	2,02	0,00	0,01	1	NA	NA	NA	NA	0	NA	NA	0
FAM3D	family with sequence similarity 3, member D	4,03	2,01	0,00	0,04	1	NA	NA	NA	NA	0	NA	NA	0
BMP2	bone morphogenetic protein 2	3,94	1,98	0,00	0,08	1	2,70	1,43	0,01	0,78	0	NA	NA	0
NA	NA	3,91	1,97	0,00	0,00	1	NA	NA	NA	NA	0	NA	NA	0
NA	NA	3,86	1,95	0,00	0,00	1	NA	NA	NA	NA	0	NA	NA	0
NA	NA	3,81	1,93	0,00	0,04	1	NA	NA	NA	NA	0	NA	NA	0
HR	hairless homolog (mouse)	3,80	1,93	0,00	0,02	1	-1,41	-0,50	0,11	1,00	0	NA	NA	0
NFKBIZ	nuclear factor of kappa light polypeptide gene enhancer in B-cells inhibitor, zeta	3,75	1,91	0,00	0,04	1	NA	NA	NA	NA	0	NA	NA	0
NA	NA	3,73	1,90	0,00	0,03	1	1,97	0,97	0,06	1,00	0	NA	NA	0
FBXO45	F-box protein 45	3,65	1,87	0,00	0,00	1	NA	NA	NA	NA	0	NA	NA	0
FAM181B	family with sequence similarity 181, member B	3,65	1,87	0,00	0,01	1	NA	NA	NA	NA	0	NA	NA	0
LIPG	lipase, endothelial	3,57	1,83	0,00	0,00	1	-1,03	-0,04	0,76	1,00	0	0,00	0,00	0
FOSB	FBJ murine osteosarcoma viral oncogene homolog B	3,56	1,83	0,00	0,09	1	1,22	0,28	0,07	1,00	0	NA	NA	0
PRDM1	PR domain containing 1, with ZNF domain	3,55	1,83	0,00	0,04	1	1,64	0,71	0,21	1,00	0	NA	NA	0
NA	NA	3,54	1,82	0,00	0,07	1	NA	NA	NA	NA	0	NA	NA	0
EPN3	epsin 3	3,52	1,81	0,00	0,02	1	1,46	0,54	0,10	1,00	0	NA	NA	0
RNASE7	ribonuclease, RNase A family, 7	3,48	1,80	0,00	0,01	1	NA	NA	NA	NA	0	NA	NA	0
VNN3	vanin 3	3,47	1,80	0,00	0,04	1	NA	NA	NA	NA	0	NA	NA	0
SLC28A3	solute carrier family 28 (sodium-coupled nucleoside transporter), member 3	3,43	1,78	0,00	0,03	1	-1,01	-0,02	0,89	1,00	0	NA	NA	0
IL11	interleukin 11	3,42	1,77	0,00	0,04	1	-1,01	-0,01	0,88	1,00	0	NA	NA	0
C5orf4	chromosome 5 open reading frame 4	3,41	1,77	0,00	0,04	1	-1,01	-0,01	NA	NA	0	NA	NA	0
LOC100129122	hypothetical LOC100129122	3,41	1,77	0,00	0,05	1	NA	NA	NA	NA	0	NA	NA	0
CSGALNACT1	chondroitin sulfate N-acetylgalactosaminyltransferase 1	3,38	1,76	0,00	0,06	1	1,01	0,02	0,90	1,00	0	1,01	0,01	0
GM2A	GM2 ganglioside activator	3,37	1,75	0,00	0,00	1	1,51	0,60	0,32	1,00	0	NA	NA	0
OVOL1	ovo-like 1(Drosophila)	3,36	1,75	0,00	0,06	1	1,24	0,31	0,41	1,00	0	NA	NA	0
NA	NA	3,33	1,74	0,00	0,08	1	NA	NA	NA	NA	0	NA	NA	0
CDH26	cadherin 26	3,33	1,74	0,00	0,04	1	NA	NA	NA	NA	0	NA	NA	0
ADRB1	adrenergic, beta-1-, receptor	3,31	1,73	0,00	0,01	1	NA	NA	NA	NA	0	NA	NA	0
HBEGF	heparin-binding EGF-like growth factor	3,29	1,72	0,00	0,02	1	1,71	0,78	0,01	0,82	0	NA	NA	0
MPHOSPH6	M-phase phosphoprotein 6	3,27	1,71	0,00	0,08	1	NA	NA	NA	NA	0	NA	NA	0

NA	NA	3,27	1,71	0,00	0,09	1	NA	NA	NA	NA	0	NA	NA	0
RNASE7	ribonuclease, RNase A family, 7	3,23	1,69	0,00	0,01	1	NA	NA	NA	NA	0	NA	NA	0
NA	NA	3,23	1,69	0,00	0,04	1	NA	NA	NA	NA	0	NA	NA	0
XDH	xanthine dehydrogenase	3,21	1,68	0,00	0,03	1	NA	NA	NA	NA	0	NA	NA	0
GM2A	GM2 ganglioside activator	3,21	1,68	0,00	0,01	1	1,84	0,88	0,31	1,00	0	NA	NA	0
FBXO45	F-box protein 45	3,18	1,67	0,00	0,01	1	NA	NA	NA	NA	0	NA	NA	0
HELLS	helicase, lymphoid-specific	3,15	1,65	0,00	0,02	1	1,23	0,29	0,39	1,00	0	NA	NA	0
SERPINA1	serpin peptidase inhibitor, clade A (alpha-1 antiproteinase, antitrypsin), member 1	3,08	1,62	0,00	0,06	1	-1,02	-0,03	NA	NA	0	NA	NA	0
TGFA	transforming growth factor, alpha	3,08	1,62	0,00	0,05	1	1,22	0,28	0,68	1,00	0	NA	NA	0
STEAP4	STEAP family member 4	3,08	1,62	0,00	0,00	1	1,02	0,03	0,94	1,00	0	NA	NA	0
WWTR1	WW domain containing transcription regulator 1	3,05	1,61	0,00	0,04	1	-1,12	-0,16	0,42	1,00	0	NA	NA	0
KCND3	potassium voltage-gated channel, Shal-related subfamily, member 3	3,03	1,60	0,00	0,01	1	-1,02	-0,02	NA	NA	0	NA	NA	0
TGM1	transglutaminase 1 (K polypeptide epidermal type I, protein-glutamine-gamma-glutamyltransferase)	3,01	1,59	0,00	0,02	1	1,50	0,59	0,02	0,90	0	NA	NA	0
TMC5	transmembrane channel-like 5	3,00	1,59	0,00	0,01	1	NA	NA	NA	NA	0	NA	NA	0
ACPP	acid phosphatase, prostate	2,98	1,58	0,00	0,01	1	NA	NA	NA	NA	0	NA	NA	0
LOC100134229	hypothetical protein LOC100134229	2,96	1,56	0,00	0,06	1	NA	NA	NA	NA	0	NA	NA	0
FST	follistatin	2,95	1,56	0,00	0,07	1	1,12	0,16	0,10	1,00	0	NA	NA	0
KCNS1	potassium voltage-gated channel, delayed-rectifier, subfamily S, member 1	2,94	1,56	0,00	0,02	1	-1,00	-0,01	NA	NA	0	NA	NA	0
WFDC5	WAP four-disulfide core domain 5	2,92	1,55	0,00	0,07	1	NA	NA	NA	NA	0	NA	NA	0
GFOD2	glucose-fructose oxidoreductase domain containing 2	2,91	1,54	0,00	0,02	1	1,13	0,18	0,26	1,00	0	NA	NA	0
SULT2B1	sulfotransferase family, cytosolic, 2B, member 1	2,89	1,53	0,00	0,06	1	1,20	0,26	0,40	1,00	0	NA	NA	0
KLK6	kallikrein-related peptidase 6	2,89	1,53	0,00	0,04	1	2,48	1,31	0,19	1,00	0	NA	NA	0
HSD17B2	hydroxysteroid (17-beta) dehydrogenase 2	2,89	1,53	0,00	0,01	1	1,38	0,47	0,42	1,00	0	NA	NA	0
SERPINA1	serpin peptidase inhibitor, clade A (alpha-1 antiproteinase, antitrypsin), member 1	2,88	1,53	0,00	0,09	1	-1,01	-0,01	NA	NA	0	NA	NA	0
ANKRD22	ankyrin repeat domain 22	2,88	1,53	0,00	0,03	1	NA	NA	NA	NA	0	NA	NA	0
TMEM38A	transmembrane protein 38A	2,88	1,53	0,00	0,08	1	NA	NA	NA	NA	0	NA	NA	0
FST	follistatin	2,88	1,53	0,00	0,08	1	1,10	0,13	0,44	1,00	0	NA	NA	0
FRY	furry homolog (Drosophila)	2,87	1,52	0,00	0,04	1	1,26	0,33	0,40	1,00	0	-1,23	-0,30	0
NA	NA	2,85	1,51	0,00	0,00	1	NA	NA	NA	NA	0	NA	NA	0
NA	NA	2,84	1,51	0,00	0,08	1	NA	NA	NA	NA	0	NA	NA	0
NA	NA	2,83	1,50	0,00	0,03	1	1,21	0,28	0,33	1,00	0	NA	NA	0
ANKRD22	ankyrin repeat domain 22	2,82	1,50	0,00	0,07	1	NA	NA	NA	NA	0	NA	NA	0
ROBO4	roundabout homolog 4, magic roundabout (Drosophila)	2,81	1,49	0,00	0,09	1	NA	NA	NA	NA	0	NA	NA	0
ADAM8	ADAM metallopeptidase domain 8	2,80	1,49	0,00	0,01	1	1,14	0,19	0,23	1,00	0	NA	NA	0
CNFN	cornifelin	2,80	1,48	0,00	0,05	1	NA	NA	NA	NA	0	NA	NA	0
EPHX3	epoxide hydrolase 3	2,79	1,48	0,00	0,00	1	2,22	1,15	0,01	0,77	0	NA	NA	0
CLCN5	chloride channel 5	2,79	1,48	0,00	0,05	1	-1,02	-0,03	0,79	1,00	0	NA	NA	0
DAPP1	dual adaptor of phosphotyrosine and 3-phosphoinositides	2,78	1,48	0,00	0,06	1	NA	NA	NA	NA	0	NA	NA	0
ITSN2	intersectin 2	2,77	1,47	0,00	0,06	1	NA	NA	NA	NA	0	NA	NA	0
KCNK10	potassium channel, subfamily K, member 10	2,75	1,46	0,00	0,04	1	NA	NA	NA	NA	0	NA	NA	0
TMEM40	transmembrane protein 40	2,75	1,46	0,00	0,07	1	1,27	0,34	0,21	1,00	0	NA	NA	0
PPIF	peptidylprolyl isomerase F	2,74	1,45	0,00	0,02	1	1,51	0,59	0,18	1,00	0	NA	NA	0
HBEGF	heparin-binding EGF-like growth factor	2,73	1,45	0,00	0,03	1	1,65	0,72	0,00	0,45	0	1,03	0,05	0
LOC339400	hypothetical LOC339400	2,73	1,45	0,00	0,04	1	NA	NA	NA	NA	0	NA	NA	0
NA	NA	2,72	1,44	0,00	0,07	1	NA	NA	NA	NA	0	NA	NA	0
PRKCH	protein kinase C, eta	2,72	1,44	0,00	0,03	1	1,03	0,04	0,80	1,00	0	NA	NA	0
NA	NA	2,72	1,44	0,00	0,06	1	NA	NA	NA	NA	0	NA	NA	0
SLC5A1	solute carrier family 5 (sodium/glucose cotransporter), member 1	2,71	1,44	0,00	0,03	1	NA	NA	NA	NA	0	NA	NA	0
METT10D	methyltransferase 10 domain containing	2,70	1,43	0,00	0,04	1	-1,20	-0,27	0,52	1,00	0	NA	NA	0
C17orf96	chromosome 17 open reading frame 96	2,69	1,43	0,00	0,03	1	NA	NA	NA	NA	0	NA	NA	0
IL1F5	interleukin 1 family, member 5 (delta)	2,68	1,42	0,00	0,01	1	2,03	1,02	0,00	0,42	0	NA	NA	0
LRRC20	leucine rich repeat containing 20	2,67	1,42	0,00	0,08	1	1,16	0,22	0,06	1,00	0	NA	NA	0
NA	NA	2,65	1,41	0,00	0,09	1	NA	NA	NA	NA	0	NA	NA	0
DOCK9	dedicator of cytokinesis 9	2,65	1,40	0,00	0,06	1	-1,03	-0,04	0,80	1,00	0	NA	NA	0
LDLR	low density lipoprotein receptor	2,64	1,40	0,00	0,04	1	-1,19	-0,25	0,74	1,00	0	NA	NA	0
FCGBP	Fc fragment of IgG binding protein	2,62	1,39	0,00	0,02	1	-1,00	-0,01	NA	NA	0	NA	NA	0
SMPD1	sphingomyelin phosphodiesterase 1, acid lysosomal	2,61	1,38	0,00	0,01	1	1,52	0,60	0,04	1,00	0	NA	NA	0
HR	hairless homolog (mouse)	2,60	1,38	0,00	0,01	1	NA	NA	NA	NA	0	NA	NA	0
NA	NA	2,60	1,38	0,00	0,03	1	1,26	0,33	0,42	1,00	0	NA	NA	0
HSPB8	heat shock 22kDa protein 8	2,60	1,38	0,00	0,01	1	NA	NA	NA	NA	0	NA	NA	0
PLIN4	perilipin 4	2,59	1,37	0,00	0,00	1	NA	NA	NA	NA	0	NA	NA	0
HMOX2	heme oxygenase (decycling) 2	2,59	1,37	0,00	0,03	1	1,39	0,48	0,00	0,31	0	NA	NA	0
BDH1	3-hydroxybutyrate dehydrogenase, type 1	2,58	1,37	0,00	0,04	1	1,30	0,37	0,22	1,00	0	NA	NA	0
USP53	ubiquitin specific peptidase 53	2,58	1,37	0,00	0,06	1	NA	NA	NA	NA	0	NA	NA	0
MAP3K9	mitogen-activated protein kinase kinase kinase 9	2,58	1,37	0,00	0,04	1	1,07	0,10	0,74	1,00	0	NA	NA	0
C7orf57	chromosome 7 open reading frame 57	2,56	1,36	0,00	0,10	1	NA	NA	NA	NA	0	NA	NA	0
FHDC1	FH2 domain containing 1	2,56	1,35	0,00	0,08	1	NA	NA	NA	NA	0	NA	NA	0
NA	NA	2,55	1,35	0,00	0,04	1	NA	NA	NA	NA	0	NA	NA	0
SPTLC2	serine palmitoyltransferase, long chain base subunit 2	2,54	1,34	0,00	0,08	1	1,30	0,38	0,07	1,00	0	NA	NA	0
SLC5A1	solute carrier family 5 (sodium/glucose cotransporter), member 1	2,53	1,34	0,00	0,04	1	-1,01	-0,01	NA	NA	0	NA	NA	0
FAM43A	family with sequence similarity 43, member A	2,52	1,33	0,00	0,03	1	NA	NA	NA	NA	0	NA	NA	0
ODZ2	odz, odd Oz/ten-m homolog 2 (Drosophila)	2,51	1,33	0,00	0,02	1	NA	NA	NA	NA	0	NA	NA	0
NA	NA	2,51	1,33	0,00	0,08	1	NA	NA	NA	NA	0	NA	NA	0
TMPRSS11D	transmembrane protease, serine 11D	2,50	1,32	0,00	0,07	1	1,01	0,02	0,89	1,00	0	NA	NA	0
CCNE1	cyclin E1	2,50	1,32	0,00	0,02	1	1,12	0,16	0,38	1,00	0	NA	NA	0
ANKRD57	ankyrin repeat domain 57	2,49	1,32	0,00	0,02	1	1,26	0,33	0,04	1,00	0	NA	NA	0
DNAJA4	DnaJ (Hsp40) homolog, subfamily A, member 4	2,48	1,31	0,00	0,04	1	NA	NA	NA	NA	0	NA	NA	0
CDYL	chromodomain protein, Y-like	2,46	1,30	0,00	0,04	1	1,28	0,36	0,10	1,00	0	NA	NA	0
GRHL3	grainyhead-like 3 (Drosophila)	2,46	1,30	0,00	0,04	1	NA	NA	NA	NA	0	NA	NA	0
NA	NA	2,46	1,30	0,00	0,02	1	NA	NA	NA	NA	0	NA	NA	0
CDH4	cadherin 4, type 1, R-cadherin (retinal)	2,45	1,29	0,00	0,02	1	1,03	0,04	0,87	1,00	0	NA	NA	0
ATP6V1C2	ATPase, H+ transporting, lysosomal 42kDa, V1 subunit C2	2,45	1,29	0,00	0,04	1	NA	NA	NA	NA	0	NA	NA	0
EXPH5	exophilin 5	2,45	1,29	0,00	0,02	1	1,09	0,12	0,42	1,00	0	NA	NA	0
ZDHHC13	zinc finger, DHHC-type containing 13	2,45	1,29	0,00	0,04	1	NA	NA	NA	NA	0	NA	NA	0
SEMA7A	semaphorin 7A, GPI membrane anchor (John Milton Hagen blood group)	2,45	1,29	0,00	0,05	1	NA	NA	NA	NA	0	NA	NA	0
GFOD2	glucose-fructose oxidoreductase domain containing 2	2,44	1,29	0,00	0,02	1	1,06	0,09	0,35	1,00	0	NA	NA	0
NA	NA	2,42	1,28	0,00	0,07	1	NA	NA	NA	NA	0	NA	NA	0
LDLR	low density lipoprotein receptor	2,42	1,27	0,00	0,04	1	-1,03	-0,05	0,89	1,00	0	NA	NA	0
TMEM171	transmembrane protein 171	2,40	1,27	0,00	0,01	1	NA	NA	NA	NA	0	NA	NA	0
RPS6KA4	ribosomal protein S6 kinase, 90kDa, polypeptide 4	2,38	1,25	0,00	0,08	1	-1,01	-0,01	0,93	1,00	0	NA	NA	0
NA	NA	2,37	1,25	0,00	0,06	1	NA	NA	NA	NA	0	NA	NA	0
HMGCS1	3-hydroxy-3-methylglutaryl-CoA synthase 1 (soluble)	2,37	1,24	0,00	0,02	1	1,05	0,07	0,82	1,00	0	NA	NA	0
RABGGTA	Rab geranylgeranyltransferase, alpha subunit	2,37	1,24	0,00	0,03	1	1,14	0,19	0,30	1,00	0	NA	NA	0
GLUL	glutamate-ammonia ligase	2,37	1,24	0,00	0,06	1	1,35	0,44	0,38	1,00	0	NA	NA	0
PSG7	pregnancy specific beta-1-glycoprotein 7 (gene/pseudogene)	2,36	1,24	0,00	0,02	1	1,04	0,05	0,77	1,00	0	0,00	0,00	0
NA	NA	2,36	1,24	0,00	0,05	1	NA	NA	NA	NA	0	NA	NA	0
SPTLC2	serine palmitoyltransferase, long chain base subunit 2	2,36	1,24	0,00	0,04	1	1,37	0,45	0,03	0,96	0	NA	NA	0
UPP1	uridine phosphorylase 1	2,36	1,24	0,00	0,08	1	1,21	0,28	0,19	1,00	0	NA	NA	0
LOC152225	hypothetical LOC152225	2,36	1,24	0,00	0,06	1	NA	NA	NA	NA	0	NA	NA	0
TLE3	transducin-like enhancer of split 3 (E(sp1) homolog, Drosophila)	2,34	1,23	0,00	0,04	1	1,20	0,26	0,25	1,00	0	NA	NA	0
TBC1D12	TBC1 domain family, member 12	2,34	1,23	0,00	0,04	1	NA	NA	NA	NA	0	NA	NA	0
BMP2	bone morphogenetic protein 2	2,33	1,22	0,00	0,10	1	2,04	1,03	0,01	0,73	0	NA	NA	0
SQLE	squalene epoxidase	2,33	1,22	0,00	0,03	1	1,05	0,07	0,87	1,00	0	-1,25	-0,32	0
EPN3	epsin 3	2,32	1,22	0,00	0,03	1	NA	NA	NA	NA	0	NA	NA	0
RIMS3	regulating synaptic membrane exocytosis 3	2,31	1,21	0,00	0,04	1	-1,00	0,00	0,99	1,00	0	NA	NA	0
NAV3	neuron navigator 3	2,31	1,21	0,00	0,09	1	NA	NA	NA	NA	0	NA	NA	0
PLIN5	perilipin 5	2,30	1,20	0,00	0,01	1	NA	NA	NA	NA	0	NA	NA	0
ABCG1	ATP-binding cassette, sub-family G (WHITE), member 1	2,30	1,20	0,00	0,06	1	1,37	0,45	0,24	1,00	0	NA	NA	0
RNASE7	ribonuclease, RNase A family, 7	2,30	1,20	0,00	0,03	1	NA	NA	NA	NA	0	NA	NA	0
HMOX2	heme oxygenase (decycling) 2	2,30	1,20	0,00	0,04	1	1,25	0,32	0,06	1,00	0	NA	NA	0
TMEM154	transmembrane protein 154	2,29	1,20	0,00	0,01	1	NA	NA	NA	NA	0	NA	NA	0
DNAJA4	DnaJ (Hsp40) homolog, subfamily A, member 4	2,29	1,20	0,00	0,08	1	NA	NA	NA	NA	0	NA	NA	0
PLCXD1	phosphatidylinositol-specific phospholipase C, X domain containing 1	2,28	1,19	0,00	0,04	1	1,30	0,38	0,13	1,00	0	NA	NA	0
CFLAR	CASP8 and FADD-like apoptosis regulator	2,28	1,19	0,00	0,03	1	1,47	0,56	0,12	1,00	0	NA	NA	0
NA	NA	2,28	1,19	0,00	0,01	1	1,31	0,39	0,26	1,00	0	NA	NA	0
GM2A	GM2 ganglioside activator	2,28	1,19	0,00	0,05	1	NA	NA	NA	NA	0	NA	NA	0
SQLE	squalene epoxidase	2,27	1,18	0,00	0,06	1	-1,04	-0,05	0,90	1,00	0	-1,28	-0,35	0
LDLR	low density lipoprotein receptor	2,27	1,18	0,00	0,08	1	1,14	0,18	0,06	1,00	0	NA	NA	0
LOC100289019	hypothetical LOC100289019	2,27	1,18	0,00	0,05	1	NA	NA	NA	NA	0	NA	NA	0
NAV3	neuron navigator 3	2,27	1,18	0,00	0,05	1	-1,04	-0,05	0,75	1,00	0	NA	NA	0
HECTD1	HECT domain containing 1	2,26	1,17	0,00	0,05	1	NA	NA	NA	NA	0	NA	NA	0
OVOL1	ovo-like 1(Drosophila)	2,25	1,17	0,00	0,02	1	NA	NA	NA	NA	0	NA	NA	0
RAPGEF5	Rap guanine nucleotide exchange factor (GEF) 5	2,24	1,17	0,00	0,07	1	1,62	0,70	0,13	1,00	0	NA	NA	0
B4GALNT3	beta-1,4-N-acetyl-galactosaminyl transferase 3	2,24	1,17	0,00	0,06	1	NA	NA	NA	NA	0	NA	NA	0
OCLN	occludin	2,23	1,16	0,00	0,08	1	1,27	0,34	0,25	1,00	0	NA	NA	0
NA	NA	2,22	1,15	0,00	0,10	1	NA	NA	NA	NA	0	NA	NA	0
LYN	v-yes-1 Yamaguchi sarcoma viral related oncogene homolog	2,22	1,15	0,00	0,10	1	1,36	0,44	0,15	1,00	0	1,11	0,15	0
ATP6V1C2	ATPase, H+ transporting, lysosomal 42kDa, V1 subunit C2	2,20	1,14	0,00	0,06	1	NA	NA	NA	NA	0	NA	NA	0
PI4K2A	phosphatidylinositol 4-kinase type 2 alpha	2,20	1,14	0,00	0,08	1	-1,13	-0,17	0,83	1,00	0	NA	NA	0
ARNTL2	aryl hydrocarbon receptor nuclear translocator-like 2	2,20	1,14	0,00	0,08	1	NA	NA	NA	NA	0	NA	NA	0
ZBED2	zinc finger, BED-type containing 2	2,20	1,14	0,00	0,08	1	-1,08	-0,11	0,31	1,00	0	NA	NA	0
GRHL1	grainyhead-like 1 (Drosophila)	2,20	1,14	0,00	0,03	1	NA	NA	NA	NA	0	NA	NA	0
NA	NA	2,20	1,14	0,00	0,04	1	NA	NA	NA	NA	0	NA	NA	0
ARSF	arylsulfatase F	2,20	1,13	0,00	0,02	1	1,03	0,04	NA	NA	0	NA	NA	0
AFAP1L2	actin filament associated protein 1-like 2	2,19	1,13	0,00	0,03	1	NA	NA	NA	NA	0	NA	NA	0
MPV17L	MPV17 mitochondrial membrane protein-like	2,18	1,12	0,00	0,03	1	NA	NA	NA	NA	0	NA	NA	0
PSG1	pregnancy specific beta-1-glycoprotein 1	2,18	1,12	0,00	0,01	1	1,00	0,00	1,00	1,00	0	NA	NA	0

STAT3	signal transducer and activator of transcription 3 (acute-phase response factor)	2,18	1,12	0,00	0,02	1	1,03	0,04	0,88	1,00	0	-1,14	-0,19	0
DCUN1D3	DCN1, defective in cullin neddylation 1, domain containing 3 (S. cerevisiae)	2,18	1,12	0,00	0,01	1	NA	NA	NA	NA	0	NA	NA	0
ITSN2	intersectin 2	2,17	1,12	0,00	0,02	1	1,79	0,84	0,00	0,31	0	NA	NA	0
TRIM62	tripartite motif-containing 62	2,17	1,12	0,00	0,06	1	1,22	0,28	0,47	1,00	0	1,07	0,10	0
PRKCH	protein kinase C, eta	2,17	1,12	0,00	0,02	1	1,30	0,38	0,02	0,88	0	NA	NA	0
GLUL	glutamate-ammonia ligase	2,17	1,12	0,00	0,05	1	1,23	0,29	0,49	1,00	0	NA	NA	0
NA	NA	2,16	1,11	0,00	0,05	1	NA	NA	NA	NA	0	NA	NA	0
C1orf116	chromosome 1 open reading frame 116	2,16	1,11	0,00	0,06	1	-1,05	-0,07	0,76	1,00	0	NA	NA	0
GMPPB	GDP-mannose pyrophosphorylase B	2,16	1,11	0,00	0,04	1	1,00	0,01	0,96	1,00	0	NA	NA	0
SMPD1	sphingomyelin phosphodiesterase 1, acid lysosomal	2,16	1,11	0,00	0,02	1	1,06	0,09	NA	NA	0	NA	NA	0
DUSP16	dual specificity phosphatase 16	2,15	1,11	0,00	0,05	1	NA	NA	NA	NA	0	NA	NA	0
NAMPT	nicotinamide phosphoribosyltransferase	2,15	1,10	0,00	0,01	1	NA	NA	NA	NA	0	NA	NA	0
SH3KBP1	SH3-domain kinase binding protein 1	2,15	1,10	0,00	0,04	1	NA	NA	NA	NA	0	NA	NA	0
ZDHHC21	zinc finger, DHHC-type containing 21	2,15	1,10	0,00	0,10	1	NA	NA	NA	NA	0	NA	NA	0
SAMD8	sterile alpha motif domain containing 8	2,15	1,10	0,00	0,09	1	NA	NA	NA	NA	0	NA	NA	0
SLC11A2	solute carrier family 11 (proton-coupled divalent metal ion transporters), member 2	2,14	1,10	0,00	0,04	1	3,34	1,74	0,03	1,00	0	1,21	0,27	0
OSBP2	oxysterol binding protein 2	2,14	1,10	0,00	0,06	1	NA	NA	NA	NA	0	NA	NA	0
FAM83D	family with sequence similarity 83, member D	2,13	1,09	0,00	0,10	1	NA	NA	NA	NA	0	NA	NA	0
GPR18	G protein-coupled receptor 18	2,13	1,09	0,00	0,08	1	-1,01	-0,01	NA	NA	0	NA	NA	0
KATNB1	katanin p80 (WD repeat containing) subunit B 1	2,13	1,09	0,00	0,03	1	1,24	0,31	0,09	1,00	0	1,01	0,01	0
KLK10	kallikrein-related peptidase 10	2,13	1,09	0,00	0,01	1	1,20	0,26	0,34	1,00	0	NA	NA	0
TMEM30B	transmembrane protein 30B	2,13	1,09	0,00	0,01	1	1,21	0,27	0,14	1,00	0	NA	NA	0
GBAP1	glucosidase, beta, acid pseudogene 1	2,11	1,08	0,00	0,01	1	1,38	0,47	0,09	1,00	0	NA	NA	0
LMTK2	lemur tyrosine kinase 2	2,11	1,07	0,00	0,08	1	NA	NA	NA	NA	0	NA	NA	0
RUSC2	RUN and SH3 domain containing 2	2,10	1,07	0,00	0,10	1	1,21	0,28	0,08	1,00	0	NA	NA	0
DLX3	distal-less homeobox 3	2,10	1,07	0,00	0,06	1	NA	NA	NA	NA	0	NA	NA	0
LYPD5	LY6/PLAUR domain containing 5	2,09	1,07	0,00	0,08	1	NA	NA	NA	NA	0	NA	NA	0
TLE3	transducin-like enhancer of split 3 (E(sp1) homolog, Drosophila)	2,09	1,06	0,00	0,04	1	1,20	0,26	0,25	1,00	0	NA	NA	0
SDR42E1	short chain dehydrogenase/reductase family 42E, member 1	2,09	1,06	0,00	0,03	1	NA	NA	NA	NA	0	NA	NA	0
CDV3	CDV3 homolog (mouse)	2,08	1,06	0,00	0,08	1	1,17	0,22	0,73	1,00	0	-1,27	-0,34	0
CKAP4	cytoskeleton-associated protein 4	2,07	1,05	0,00	0,02	1	1,26	0,33	0,15	1,00	0	NA	NA	0
ZNF800	zinc finger protein 800	2,06	1,04	0,00	0,09	1	NA	NA	NA	NA	0	NA	NA	0
NSDHL	NAD(P) dependent steroid dehydrogenase-like	2,06	1,04	0,00	0,07	1	1,15	0,20	0,13	1,00	0	NA	NA	0
TAOK1	TAO kinase 1	2,04	1,03	0,00	0,08	1	NA	NA	NA	NA	0	NA	NA	0
NAMPT	nicotinamide phosphoribosyltransferase	2,03	1,02	0,00	0,07	1	NA	NA	NA	NA	0	NA	NA	0
TTC39A	tetratricopeptide repeat domain 39A	2,03	1,02	0,00	0,02	1	1,53	0,61	0,22	1,00	0	NA	NA	0
USP6NL	USP6 N-terminal like	2,03	1,02	0,00	0,03	1	1,28	0,36	0,05	1,00	0	NA	NA	0
RELL2	RELT-like 2	2,02	1,02	0,00	0,09	1	NA	NA	NA	NA	0	NA	NA	0
HECTD1	HECT domain containing 1	2,02	1,02	0,00	0,04	1	NA	NA	NA	NA	0	NA	NA	0
TRIM62	tripartite motif-containing 62	2,02	1,01	0,00	0,07	1	1,37	0,45	0,09	1,00	0	-1,01	-0,01	0
ARNTL2	aryl hydrocarbon receptor nuclear translocator-like 2	2,02	1,01	0,00	0,05	1	NA	NA	NA	NA	0	NA	NA	0
SHB	Src homology 2 domain containing adaptor protein B	2,01	1,01	0,00	0,10	1	NA	NA	NA	NA	0	NA	NA	0
LOC100133660	hypothetical LOC100133660	2,01	1,01	0,00	0,05	1	NA	NA	NA	NA	0	NA	NA	0
NA	NA	2,01	1,01	0,00	0,10	1	NA	NA	NA	NA	0	NA	NA	0
ZDHHC13	zinc finger, DHHC-type containing 13	2,01	1,01	0,00	0,03	1	1,25	0,32	0,48	1,00	0	NA	NA	0
ARNTL2	aryl hydrocarbon receptor nuclear translocator-like 2	2,01	1,00	0,00	0,05	1	1,38	0,47	0,02	0,92	0	-1,52	-0,59	0
PSG4	pregnancy specific beta-1-glycoprotein 4	2,00	1,00	0,00	0,04	1	1,04	0,06	0,95	1,00	0	-1,01	-0,01	0
NA	NA	2,00	1,00	0,00	0,08	1	NA	NA	NA	NA	0	NA	NA	0
ADRB2	adrenergic, beta-2-, receptor, surface	2,00	1,00	0,00	0,04	1	1,17	0,23	0,11	1,00	0	NA	NA	0
SPIRE1	spire homolog 1 (Drosophila)	1,98	0,98	0,00	0,05	1	NA	NA	NA	NA	0	NA	NA	0
FEZ1	fasciculation and elongation protein zeta 1 (zygin I)	1,97	0,98	0,00	0,08	1	1,26	0,34	0,04	1,00	0	NA	NA	0
MAP2K3	mitogen-activated protein kinase kinase 3	1,97	0,98	0,00	0,03	1	1,00	0,00	0,99	1,00	0	NA	NA	0
PSG6	pregnancy specific beta-1-glycoprotein 6	1,97	0,98	0,00	0,01	1	1,00	0,00	NA	NA	0	NA	NA	0
KCTD11	potassium channel tetramerisation domain containing 11	1,96	0,97	0,00	0,07	1	NA	NA	NA	NA	0	NA	NA	0
ODC1	ornithine decarboxylase 1	1,96	0,97	0,00	0,10	1	1,12	0,17	0,15	1,00	0	-1,02	-0,04	0
NA	NA	1,96	0,97	0,00	0,04	1	-1,00	-0,01	NA	NA	0	1,37	0,45	0
UGCG	UDP-glucose ceramide glucosyltransferase	1,95	0,97	0,00	0,09	1	1,08	0,12	0,89	1,00	0	NA	NA	0
NA	NA	1,95	0,96	0,00	0,10	1	NA	NA	NA	NA	0	NA	NA	0
SLC25A25	solute carrier family 25 (mitochondrial carrier; phosphate carrier), member 25	1,95	0,96	0,00	0,07	1	NA	NA	NA	NA	0	NA	NA	0
MPZL2	myelin protein zero-like 2	1,95	0,96	0,00	0,04	1	1,32	0,40	0,34	1,00	0	NA	NA	0
SOX7	SRY (sex determining region Y)-box 7	1,94	0,96	0,00	0,04	1	NA	NA	NA	NA	0	NA	NA	0
SHB	Src homology 2 domain containing adaptor protein B	1,94	0,95	0,00	0,08	1	1,11	0,15	0,41	1,00	0	NA	NA	0
KRT80	keratin 80	1,93	0,95	0,00	0,06	1	NA	NA	NA	NA	0	NA	NA	0
GPR68	G protein-coupled receptor 68	1,93	0,95	0,00	0,03	1	NA	NA	NA	NA	0	NA	NA	0
NA	NA	1,93	0,95	0,00	0,06	1	-1,01	-0,01	NA	NA	0	NA	NA	0
JMY	junction mediating and regulatory protein, p53 cofactor	1,92	0,94	0,00	0,10	1	NA	NA	NA	NA	0	NA	NA	0
PGLYRP4	peptidoglycan recognition protein 4	1,92	0,94	0,00	0,09	1	1,62	0,70	0,09	1,00	0	NA	NA	0
TMEM88	transmembrane protein 88	1,92	0,94	0,00	0,02	1	NA	NA	NA	NA	0	NA	NA	0
CFLAR	CASP8 and FADD-like apoptosis regulator	1,91	0,93	0,00	0,05	1	1,46	0,55	0,12	1,00	0	NA	NA	0
KLF3	Kruppel-like factor 3 (basic)	1,91	0,93	0,00	0,08	1	NA	NA	NA	NA	0	NA	NA	0
CFLAR	CASP8 and FADD-like apoptosis regulator	1,91	0,93	0,00	0,07	1	1,28	0,36	0,17	1,00	0	NA	NA	0
PRMT5	protein arginine methyltransferase 5	1,89	0,92	0,00	0,04	1	-1,06	-0,09	0,25	1,00	0	NA	NA	0
CARHSP1	calcium regulated heat stable protein 1, 24kDa	1,89	0,92	0,00	0,08	1	1,15	0,20	0,21	1,00	0	NA	NA	0
NA	NA	1,89	0,92	0,00	0,04	1	NA	NA	NA	NA	0	NA	NA	0
NA	NA	1,88	0,91	0,00	0,08	1	NA	NA	NA	NA	0	NA	NA	0
CFLAR	CASP8 and FADD-like apoptosis regulator	1,88	0,91	0,00	0,10	1	1,28	0,35	0,08	1,00	0	NA	NA	0
CDCA5	cell division cycle associated 5	1,88	0,91	0,00	0,04	1	NA	NA	NA	NA	0	NA	NA	0
SERPINB3	serpin peptidase inhibitor, clade B (ovalbumin), member 3	1,88	0,91	0,00	0,01	1	1,75	0,81	0,02	0,84	0	NA	NA	0
NLRX1	NLR family member X1	1,87	0,91	0,00	0,09	1	1,15	0,20	0,34	1,00	0	NA	NA	0
STK38L	serine/threonine kinase 38 like	1,87	0,90	0,00	0,10	1	1,11	0,15	0,28	1,00	0	-1,37	-0,45	0
ALAS1	aminolevulinate, delta-, synthase 1	1,86	0,89	0,00	0,07	1	1,12	0,16	0,09	1,00	0	NA	NA	0
ACOX1	acyl-CoA oxidase 1, palmitoyl	1,85	0,89	0,00	0,06	1	1,55	0,63	0,05	1,00	0	NA	NA	0
LPIN2	lipin 2	1,85	0,89	0,00	0,04	1	1,11	0,15	0,18	1,00	0	NA	NA	0
DNAJC5	DnaJ (Hsp40) homolog, subfamily C, member 5	1,85	0,88	0,00	0,04	1	NA	NA	NA	NA	0	NA	NA	0
NA	NA	1,84	0,88	0,00	0,05	1	NA	NA	NA	NA	0	NA	NA	0
PTPN11	protein tyrosine phosphatase, non-receptor type 11	1,83	0,88	0,00	0,07	1	-1,04	-0,06	0,90	1,00	0	-1,12	-0,17	0
PRMT5	protein arginine methyltransferase 5	1,83	0,87	0,00	0,02	1	NA	NA	NA	NA	0	NA	NA	0
FEM1B	fem-1 homolog b (C. elegans)	1,83	0,87	0,00	0,05	1	1,15	0,20	0,13	1,00	0	NA	NA	0
LRRC8B	leucine rich repeat containing 8 family, member B	1,83	0,87	0,00	0,05	1	1,13	0,18	0,06	1,00	0	NA	NA	0
NA	NA	1,82	0,86	0,00	0,08	1	NA	NA	NA	NA	0	NA	NA	0
RRM2	ribonucleotide reductase M2	1,82	0,86	0,00	0,05	1	1,04	0,06	0,62	1,00	0	NA	NA	0
USP38	ubiquitin specific peptidase 38	1,81	0,86	0,00	0,10	1	NA	NA	NA	NA	0	NA	NA	0
NA	NA	1,81	0,85	0,00	0,08	1	NA	NA	NA	NA	0	NA	NA	0
KATNB1	katanin p80 (WD repeat containing) subunit B 1	1,80	0,85	0,00	0,05	1	NA	NA	NA	NA	0	NA	NA	0
NSDHL	NAD(P) dependent steroid dehydrogenase-like	1,80	0,85	0,00	0,08	1	1,05	0,07	0,50	1,00	0	NA	NA	0
NA	NA	1,79	0,84	0,00	0,06	1	NA	NA	NA	NA	0	NA	NA	0
TSC22D1	TSC22 domain family, member 1	1,79	0,84	0,00	0,04	1	-1,20	-0,26	0,05	1,00	0	NA	NA	0
MPRIP	myosin phosphatase Rho interacting protein	1,79	0,84	0,00	0,06	1	NA	NA	NA	NA	0	NA	NA	0
TNFRSF4	tumor necrosis factor receptor superfamily, member 4	1,79	0,84	0,00	0,08	1	-1,01	-0,01	NA	NA	0	NA	NA	0
CSRNP2	cysteine-serine-rich nuclear protein 2	1,78	0,84	0,00	0,08	1	NA	NA	NA	NA	0	NA	NA	0
MYO5B	myosin VB	1,78	0,83	0,00	0,05	1	NA	NA	NA	NA	0	NA	NA	0
TFRC	transferrin receptor (p90, CD71)	1,77	0,83	0,00	0,06	1	NA	NA	NA	NA	0	NA	NA	0
NA	NA	1,77	0,82	0,00	0,09	1	1,29	0,37	0,03	1,00	0	NA	NA	0
CHST2	carbohydrate (N-acetylglucosamine-6-O) sulfotransferase 2	1,77	0,82	0,00	0,02	1	1,27	0,34	0,33	1,00	0	NA	NA	0
TEX101	testis expressed 101	1,76	0,82	0,00	0,10	1	NA	NA	NA	NA	0	NA	NA	0
DUSP16	dual specificity phosphatase 16	1,75	0,81	0,00	0,02	1	NA	NA	NA	NA	0	NA	NA	0
SLC30A1	solute carrier family 30 (zinc transporter), member 1	1,75	0,81	0,00	0,06	1	NA	NA	NA	NA	0	NA	NA	0
IDH3A	isocitrate dehydrogenase 3 (NAD+) alpha	1,74	0,80	0,00	0,04	1	1,22	0,29	0,01	0,77	0	NA	NA	0
NA	NA	1,74	0,80	0,00	0,06	1	NA	NA	NA	NA	0	NA	NA	0
TPM3	tropomyosin 3	1,74	0,80	0,00	0,04	1	NA	NA	NA	NA	0	NA	NA	0
DTL	denticleless homolog (Drosophila)	1,73	0,79	0,00	0,09	1	-1,11	-0,15	0,17	1,00	0	NA	NA	0
N4BP1	NEDD4 binding protein 1	1,72	0,78	0,00	0,06	1	1,42	0,51	0,01	0,83	0	-1,06	-0,08	0
SAV1	salvador homolog 1 (Drosophila)	1,72	0,78	0,00	0,03	1	NA	NA	NA	NA	0	NA	NA	0
C10orf119	chromosome 10 open reading frame 119	1,71	0,77	0,00	0,03	1	1,03	0,05	0,84	1,00	0	NA	NA	0
C1orf104	chromosome 1 open reading frame 104	1,70	0,77	0,00	0,04	1	NA	NA	NA	NA	0	NA	NA	0
SIAH2	seven in absentia homolog 2 (Drosophila)	1,70	0,77	0,00	0,04	1	1,12	0,17	0,08	1,00	0	NA	NA	0
KIF1B	kinesin family member 1B	1,69	0,76	0,00	0,08	1	NA	NA	NA	NA	0	NA	NA	0
ADAM9	ADAM metallopeptidase domain 9	1,69	0,76	0,00	0,08	1	NA	NA	NA	NA	0	NA	NA	0
DNAJC5	DnaJ (Hsp40) homolog, subfamily C, member 5	1,68	0,75	0,00	0,04	1	NA	NA	NA	NA	0	NA	NA	0
CDC25A	cell division cycle 25 homolog A (S. pombe)	1,68	0,75	0,00	0,10	1	1,09	0,12	0,52	1,00	0	NA	NA	0
PSME4	proteasome (prosome, macropain) activator subunit 4	1,67	0,74	0,00	0,10	1	-1,14	-0,20	0,83	1,00	0	-1,19	-0,25	0
ATG13	ATG13 autophagy related 13 homolog (S. cerevisiae)	1,67	0,74	0,00	0,08	1	-1,02	-0,03	0,93	1,00	0	NA	NA	0
C4orf10	chromosome 4 open reading frame 10	1,67	0,74	0,00	0,07	1	-1,01	-0,01	NA	NA	0	NA	NA	0
PTGES2	prostaglandin E synthase 2	1,67	0,74	0,00	0,08	1	-1,01	-0,01	0,98	1,00	0	NA	NA	0
ERCC1	excision repair cross-complementing rodent repair deficiency, complementation group 1 (includes overlapping antisense sequence)	1,66	0,73	0,00	0,08	1	-1,12	-0,17	0,15	1,00	0	NA	NA	0
HSPA8	heat shock 70kDa protein 8	1,66	0,73	0,00	0,02	1	1,01	0,01	0,91	1,00	0	NA	NA	0
CAB39	calcium binding protein 39	1,66	0,73	0,00	0,09	1	NA	NA	NA	NA	0	NA	NA	0
NA	NA	1,66	0,73	0,00	0,10	1	NA	NA	NA	NA	0	NA	NA	0
FAM108C1	family with sequence similarity 108, member C1	1,66	0,73	0,00	0,03	1	NA	NA	NA	NA	0	NA	NA	0
CLCN5	chloride channel 5	1,66	0,73	0,00	0,10	1	NA	NA	NA	NA	0	NA	NA	0
SHC1	SHC (Src homology 2 domain containing) transforming protein 1	1,66	0,73	0,00	0,05	1	-1,13	-0,17	0,61	1,00	0	NA	NA	0
DLG3	discs, large homolog 3 (Drosophila)	1,65	0,73	0,00	0,09	1	-1,01	-0,02	0,89	1,00	0	NA	NA	0
SLC31A1	solute carrier family 31 (copper transporters), member 1	1,65	0,72	0,00	0,05	1	NA	NA	NA	NA	0	NA	NA	0
DHCR7	7-dehydrocholesterol reductase	1,65	0,72	0,00	0,10	1	-1,02	-0,02	0,93	1,00	0	1,18	0,24	0
ARHGEF37	Rho guanine nucleotide exchange factor (GEF) 37	1,65	0,72	0,00	0,07	1	NA	NA	NA	NA	0	NA	NA	0
SLC43A3	solute carrier family 43, member 3	1,64	0,71	0,00	0,04	1	1,35	0,43	0,33	1,00	0	NA	NA	0
PSG6	pregnancy specific beta-1-glycoprotein 6	1,64	0,71	0,00	0,07	1	-1,01	-0,01	0,96	1,00	0	NA	NA	0

NA	NA	1,63	0,71	0,00	0,06	1	1,14	0,19	0,14	1,00	0	NA	NA	0
CARS	cysteinyl-tRNA synthetase	1,63	0,71	0,00	0,07	1	NA	NA	NA	NA	0	NA	NA	0
CWH43	cell wall biogenesis 43 C-terminal homolog (S. cerevisiae)	1,61	0,69	0,00	0,02	1	1,59	0,67	0,04	1,00	0	NA	NA	0
KRT23	keratin 23 (histone deacetylase inducible)	1,61	0,69	0,00	0,10	1	1,92	0,94	0,02	0,94	0	NA	NA	0
PCSK6	proprotein convertase subtilisin/kexin type 6	1,61	0,68	0,00	0,07	1	1,01	0,02	NA	NA	0	NA	NA	0
NA	NA	1,60	0,68	0,00	0,06	1	NA	NA	NA	NA	0	NA	NA	0
MYO1B	myosin IB	1,59	0,67	0,00	0,07	1	1,18	0,24	0,08	1,00	0	NA	NA	0
PRMT5	protein arginine methyltransferase 5	1,58	0,66	0,00	0,05	1	NA	NA	NA	NA	0	NA	NA	0
ATG7	ATG7 autophagy related 7 homolog (S. cerevisiae)	1,58	0,66	0,00	0,10	1	NA	NA	NA	NA	0	NA	NA	0
PPFIBP2	PTPRF interacting protein, binding protein 2 (liprin beta 2)	1,58	0,66	0,00	0,10	1	1,34	0,43	0,07	1,00	0	-1,10	-0,14	0
DTL	denticleless homolog (Drosophila)	1,57	0,65	0,00	0,07	1	NA	NA	NA	NA	0	NA	NA	0
SEC23B	Sec23 homolog B (S. cerevisiae)	1,57	0,65	0,00	0,10	1	1,15	0,20	0,05	1,00	0	NA	NA	0
GBA2	glucosidase, beta (bile acid) 2	1,57	0,65	0,00	0,10	1	NA	NA	NA	NA	0	NA	NA	0
PRDM1	PR domain containing 1, with ZNF domain	1,56	0,65	0,00	0,09	1	NA	NA	NA	NA	0	NA	NA	0
ZNF212	zinc finger protein 212	1,56	0,64	0,00	0,08	1	-1,04	-0,06	0,57	1,00	0	NA	NA	0
CDYL	chromodomain protein, Y-like	1,56	0,64	0,00	0,08	1	1,15	0,21	0,42	1,00	0	NA	NA	0
NA	NA	1,55	0,63	0,00	0,10	1	1,01	0,01	0,93	1,00	0	-1,19	-0,26	0
KIF1B	kinesin family member 1B	1,54	0,62	0,00	0,09	1	NA	NA	NA	NA	0	NA	NA	0
FJX1	four jointed box 1 (Drosophila)	1,54	0,62	0,00	0,09	1	1,05	0,06	0,84	1,00	0	NA	NA	0
NA	NA	1,54	0,62	0,00	0,06	1	NA	NA	NA	NA	0	NA	NA	0
QSOX2	quiescin Q6 sulfhydryl oxidase 2	1,53	0,62	0,00	0,06	1	NA	NA	NA	NA	0	NA	NA	0
ZCCHC6	zinc finger, CCHC domain containing 6	1,53	0,62	0,00	0,08	1	NA	NA	NA	NA	0	NA	NA	0
CTPS	CTP synthase	1,52	0,60	0,00	0,09	1	1,12	0,16	0,18	1,00	0	NA	NA	0
ENSA	endosulfine alpha	1,51	0,60	0,00	0,09	1	1,12	0,16	0,29	1,00	0	NA	NA	0
PPARD	peroxisome proliferator-activated receptor delta	1,51	0,59	0,00	0,10	1	1,25	0,32	0,17	1,00	0	NA	NA	0
ACOX1	acyl-CoA oxidase 1, palmitoyl	1,70	0,77	0,00	0,07	1	1,81	0,85	0,00	0,28	0	-1,56	-0,65	-1
IL8	interleukin 8	2,19	1,13	0,03	0,30	0	14,53	3,86	0,00	0,02	1	9,37	3,23	1
CXCL3	chemokine (C-X-C motif) ligand 3	1,52	0,61	0,21	0,61	0	3,81	1,93	0,00	0,00	1	55,41	5,79	1
MAP3K8	mitogen-activated protein kinase kinase kinase 8	1,48	0,56	0,01	0,19	0	3,56	1,83	0,00	0,03	1	2,41	1,27	1
CXCL5	chemokine (C-X-C motif) ligand 5	1,45	0,54	0,44	0,78	0	5,64	2,50	0,00	0,01	1	9,22	3,20	1
IER3	immediate early response 3	1,36	0,44	0,03	0,30	0	1,73	0,79	0,00	0,00	1	2,35	1,23	1
CXCL2	chemokine (C-X-C motif) ligand 2	1,28	0,35	0,25	0,65	0	6,01	2,59	0,00	0,00	1	35,50	5,15	1
C3	complement component 3	1,27	0,34	0,34	0,72	0	4,05	2,02	0,00	0,10	1	7,79	2,96	1
CXCL1	chemokine (C-X-C motif) ligand 1 (melanoma growth stimulating activity, alpha)	1,14	0,19	0,38	0,75	0	7,69	2,94	0,00	0,00	1	6,75	2,75	1
IL8	interleukin 8	1,14	0,19	0,36	0,74	0	5,04	2,33	0,00	0,00	1	5,82	2,54	1
CXCL6	chemokine (C-X-C motif) ligand 6 (granulocyte chemotactic protein 2)	1,12	0,17	0,70	0,91	0	26,15	4,71	0,00	0,00	1	6,31	2,66	1
NA	NA	1,10	0,14	0,42	0,78	0	2,03	1,02	0,00	0,00	1	1,99	0,99	1
S100A8	S100 calcium binding protein A8	NA	NA	NA	NA	0	1,58	0,66	0,00	0,06	1	NA	NA	0
KLK12	kallikrein-related peptidase 12	5,76	2,53	0,00	0,14	0	4,18	2,06	0,00	0,15	1	NA	NA	0
RHCG	Rh family, C glycoprotein	3,38	1,76	0,01	0,15	0	6,00	2,59	0,00	0,00	1	NA	NA	0
PSORS1C2	psoriasis susceptibility 1 candidate 2	2,73	1,45	0,04	0,32	0	2,44	1,29	0,00	0,16	1	NA	NA	0
CCL20	chemokine (C-C motif) ligand 20	2,34	1,23	0,01	0,15	0	28,31	4,82	0,00	0,00	1	NA	NA	0
RPS6KA5	ribosomal protein S6 kinase, 90kDa, polypeptide 5	2,10	1,07	0,01	0,22	0	1,61	0,69	0,00	0,10	1	NA	NA	0
TYMP	thymidine phosphorylase	1,98	0,98	0,02	0,28	0	1,62	0,69	0,00	0,16	1	1,21	0,27	0
HSPC159	galectin-related protein	1,94	0,96	0,01	0,18	0	1,80	0,85	0,00	0,16	1	NA	NA	0
ALDH1A3	aldehyde dehydrogenase 1 family, member A3	1,83	0,87	0,09	0,46	0	1,55	0,63	0,00	0,10	1	NA	NA	0
LCN2	lipocalin 2	1,61	0,69	0,04	0,32	0	4,53	2,18	0,00	0,01	1	NA	NA	0
NA	NA	1,60	0,68	0,14	0,53	0	25,29	4,66	0,00	0,00	1	NA	NA	0
PDZK1IP1	PDZK1 interacting protein 1	1,58	0,66	0,03	0,29	0	25,95	4,70	0,00	0,00	1	NA	NA	0
TFRC	transferrin receptor (p90, CD71)	1,55	0,64	0,01	0,17	0	2,09	1,06	0,00	0,00	1	NA	NA	0
SAT1	spermidine/spermine N1-acetyltransferase 1	1,50	0,59	0,04	0,35	0	1,64	0,72	0,00	0,05	1	1,04	0,06	0
S100A9	S100 calcium binding protein A9	1,40	0,49	0,03	0,29	0	2,15	1,10	0,00	0,04	1	NA	NA	0
NA	NA	1,39	0,48	0,15	0,55	0	13,50	3,75	0,00	0,00	1	NA	NA	0
PI3	peptidase inhibitor 3, skin-derived	1,39	0,47	0,01	0,18	0	3,67	1,88	0,00	0,00	1	NA	NA	0
MAFF	v-maf musculoaponeurotic fibrosarcoma oncogene homolog F (avian)	1,36	0,44	0,03	0,30	0	1,54	0,62	0,00	0,16	1	NA	NA	0
TFRC	transferrin receptor (p90, CD71)	1,34	0,42	0,14	0,53	0	1,76	0,82	0,00	0,00	1	NA	NA	0
PI3	peptidase inhibitor 3, skin-derived	1,34	0,42	0,02	0,24	0	4,48	2,16	0,00	0,00	1	NA	NA	0
NAMPT	nicotinamide phosphoribosyltransferase	1,28	0,36	0,02	0,28	0	1,59	0,67	0,00	0,01	1	1,23	0,30	0
NA	NA	1,27	0,34	0,03	0,29	0	3,44	1,78	0,00	0,00	1	NA	NA	0
ATG4A	ATG4 autophagy related 4 homolog A (S. cerevisiae)	1,24	0,31	0,09	0,45	0	1,84	0,88	0,00	0,11	1	NA	NA	0
CRABP2	cellular retinoic acid binding protein 2	1,19	0,25	0,14	0,53	0	2,58	1,37	0,00	0,02	1	NA	NA	0
S100A7	S100 calcium binding protein A7	1,06	0,08	0,52	0,83	0	189,38	7,57	0,00	0,00	1	NA	NA	0
S100P	S100 calcium binding protein P	0,99	-0,02	0,92	0,98	0	6,35	2,67	0,00	0,12	1	NA	NA	0
TYW1	tRNA-yW synthesizing protein 1 homolog (S. cerevisiae)	0,97	-0,04	0,78	0,94	0	1,54	0,62	0,00	0,01	1	NA	NA	0
SLC25A12	solute carrier family 25 (mitochondrial carrier, Aralar), member 12	0,93	-0,10	0,66	0,89	0	2,14	1,10	0,00	0,08	1	NA	NA	0
TNFSF10	tumor necrosis factor (ligand) superfamily, member 10	0,81	-0,30	0,50	0,82	0	2,53	1,34	0,00	0,18	1	0,00	0,01	0
NMB	neuromedin B	0,62	-0,69	0,03	0,29	0	2,27	1,18	0,00	0,08	1	1,05	0,07	0
PLK1	polo-like kinase 1	NA	NA	NA	NA	0	1,18	0,24	0,38	1,00	0	5,23	2,39	1
TRIO	triple functional domain (PTPRF interacting)	NA	NA	NA	NA	0	1,07	0,10	0,40	1,00	0	2,31	1,21	1
PTPRR	protein tyrosine phosphatase, receptor type, R	NA	NA	NA	NA	0	1,00	0,00	NA	NA	0	1,78	0,83	1
ELN	elastin	NA	NA	NA	NA	0	-1,01	-0,01	NA	NA	0	5,69	2,51	1
TRIO	triple functional domain (PTPRF interacting)	NA	NA	NA	NA	0	1,05	0,07	NA	NA	0	2,15	1,11	1
BEX1	brain expressed, X-linked 1	NA	NA	NA	NA	0	-1,00	0,00	NA	NA	0	1,75	0,80	1
SOD3	superoxide dismutase 3, extracellular	NA	NA	NA	NA	0	1,00	0,00	NA	NA	0	1,57	0,65	1
CXCL5	chemokine (C-X-C motif) ligand 5	2,25	1,17	0,07	0,40	0	1,26	0,34	0,71	1,00	0	21,97	4,46	1
POU2F2	POU class 2 homeobox 2	1,59	0,67	0,01	0,20	0	1,36	0,44	0,03	1,00	0	4,97	2,31	1
SOCS1	suppressor of cytokine signaling 1	1,56	0,64	0,01	0,21	0	-1,03	-0,05	0,90	1,00	0	4,29	2,10	1
PTGS2	prostaglandin-endoperoxide synthase 2 (prostaglandin G/H synthase and cyclooxygenase)	1,50	0,59	0,13	0,52	0	1,67	0,74	0,01	0,79	0	1,98	0,99	1
CLDN1	claudin 1	1,45	0,54	0,10	0,47	0	-1,04	-0,06	0,88	1,00	0	2,70	1,43	1
AMPD3	adenosine monophosphate deaminase 3	1,40	0,48	0,02	0,24	0	1,30	0,38	0,04	1,00	0	2,21	1,14	1
CORO1B	coronin, actin binding protein, 1B	1,38	0,46	0,27	0,67	0	-1,81	-0,86	0,07	1,00	0	3,05	1,61	1
C1QTNF1	C1q and tumor necrosis factor related protein 1	1,34	0,42	0,20	0,60	0	1,06	0,08	0,40	1,00	0	2,77	1,47	1
IL6	interleukin 6 (interferon, beta 2)	1,26	0,33	0,57	0,86	0	1,05	0,07	0,80	1,00	0	4,54	2,18	1
COL7A1	collagen, type VII, alpha 1	1,25	0,33	0,11	0,49	0	-1,21	-0,28	0,20	1,00	0	2,04	1,02	1
PIK3CD	phosphoinositide-3-kinase, catalytic, delta polypeptide	1,24	0,32	0,10	0,48	0	1,38	0,46	0,02	0,91	0	1,52	0,60	1
TRIP10	thyroid hormone receptor interactor 10	1,24	0,31	0,43	0,78	0	1,15	0,21	0,29	1,00	0	2,09	1,07	1
TMEM132A	transmembrane protein 132A	1,20	0,27	0,47	0,80	0	1,78	0,83	0,01	0,51	0	1,60	0,68	1
ARF3	ADP-ribosylation factor 3	1,20	0,26	0,13	0,52	0	-1,01	-0,02	0,89	1,00	0	1,56	0,64	1
PAPPA	pregnancy-associated plasma protein A, pappalysin 1	1,18	0,24	0,35	0,73	0	-1,01	-0,01	NA	NA	0	1,82	0,86	1
SVIL	supervillin	1,17	0,23	0,62	0,88	0	1,47	0,56	0,10	1,00	0	2,19	1,13	1
RNF24	ring finger protein 24	1,14	0,19	0,62	0,88	0	-1,51	-0,60	0,02	0,94	0	2,05	1,04	1
SOD2	superoxide dismutase 2, mitochondrial	1,10	0,14	0,41	0,77	0	1,74	0,80	0,00	0,45	0	2,49	1,32	1
STC1	stanniocalcin 1	1,09	0,12	0,83	0,95	0	-1,00	0,00	NA	NA	0	3,70	1,89	1
PNPLA6	patatin-like phospholipase domain containing 6	1,08	0,12	0,62	0,88	0	-1,02	-0,03	0,88	1,00	0	3,18	1,67	1
TRIO	triple functional domain (PTPRF interacting)	1,07	0,10	0,54	0,84	0	1,13	0,18	0,18	1,00	0	1,92	0,94	1
PCGF1	polycomb group ring finger 1	1,06	0,08	0,65	0,89	0	1,12	0,16	0,36	1,00	0	2,43	1,28	1
MAZ	MYC-associated zinc finger protein (purine-binding transcription factor)	1,04	0,06	0,70	0,91	0	-1,39	-0,48	0,07	1,00	0	2,11	1,07	1
STC1	stanniocalcin 1	1,04	0,05	0,69	0,91	0	-1,01	-0,01	NA	NA	0	3,39	1,76	1
PAM16	presequence translocase-asssociated motor 16 homolog (S. cerevisiae)	1,03	0,04	0,85	0,96	0	1,00	0,00	0,99	1,00	0	1,63	0,71	1
LRIG1	leucine-rich repeats and immunoglobulin-like domains 1	1,03	0,04	0,87	0,97	0	1,09	0,13	0,48	1,00	0	3,62	1,86	1
SQSTM1	sequestosome 1	1,02	0,02	0,91	0,98	0	1,33	0,41	0,30	1,00	0	2,80	1,49	1
SOD2	superoxide dismutase 2, mitochondrial	1,00	0,00	1,00	1,00	0	1,35	0,44	0,03	1,00	0	2,30	1,20	1
IGFBP4	insulin-like growth factor binding protein 4	0,99	-0,02	0,93	0,98	0	-1,23	-0,30	0,32	1,00	0	1,55	0,63	1
SOD2	superoxide dismutase 2, mitochondrial	0,98	-0,03	0,89	0,97	0	1,38	0,47	0,01	0,54	0	2,07	1,05	1
ENC1	ectodermal-neural cortex 1 (with BTB-like domain)	0,97	-0,04	0,91	0,98	0	1,26	0,34	0,02	0,88	0	1,60	0,68	1
SVIL	supervillin	0,97	-0,04	0,86	0,96	0	1,29	0,37	0,08	1,00	0	1,89	0,92	1
AGRN	agrin	0,96	-0,06	0,81	0,95	0	-1,29	-0,37	0,04	1,00	0	2,16	1,11	1
SLC4A2	solute carrier family 4, anion exchanger, member 2 (erythrocyte membrane protein band 3-like 1)	0,95	-0,07	0,77	0,93	0	-1,19	-0,25	0,24	1,00	0	1,93	0,95	1
CHI3L1	chitinase 3-like 1 (cartilage glycoprotein-39)	0,94	-0,09	0,72	0,91	0	-1,03	-0,04	NA	NA	0	2,23	1,16	1
IRAK3	interleukin-1 receptor-associated kinase 3	0,94	-0,09	0,77	0,93	0	-1,02	-0,03	NA	NA	0	2,46	1,30	1
TNFAIP2	tumor necrosis factor, alpha-induced protein 2	0,93	-0,10	0,81	0,95	0	1,03	0,04	0,88	1,00	0	1,61	0,69	1
AGRN	agrin	0,92	-0,12	0,65	0,89	0	-1,28	-0,36	0,03	1,00	0	2,18	1,12	1
PIGV	phosphatidylinositol glycan anchor biosynthesis, class V	0,91	-0,13	0,36	0,73	0	1,06	0,08	0,33	1,00	0	1,62	0,69	1
COL5A3	collagen, type V, alpha 3	0,90	-0,16	0,82	0,95	0	1,01	0,01	NA	NA	0	2,00	1,00	1
BMP1	bone morphogenetic protein 1	0,89	-0,17	0,75	0,93	0	-1,05	-0,07	0,82	1,00	0	2,33	1,22	1
NA	NA	0,88	-0,19	0,62	0,88	0	-1,00	0,00	NA	NA	0	14,31	3,84	1
KCNJ2	potassium inwardly-rectifying channel, subfamily J, member 2	0,87	-0,20	0,69	0,90	0	-1,00	0,00	0,99	1,00	0	2,01	1,01	1
C8orf4	chromosome 8 open reading frame 4	0,86	-0,22	0,57	0,85	0	-1,02	-0,03	0,78	1,00	0	1,52	0,60	1
DPP4	dipeptidyl-peptidase 4	0,84	-0,25	0,45	0,79	0	-1,00	0,00	NA	NA	0	1,53	0,61	1
CES2	carboxylesterase 2 (intestine, liver)	0,77	-0,37	0,25	0,66	0	-1,03	-0,04	0,91	1,00	0	1,70	0,76	1
DNM1	dynamin 1	0,77	-0,37	0,14	0,53	0	-1,01	-0,01	NA	NA	0	2,40	1,26	1
PKN1	protein kinase N1	0,72	-0,48	0,18	0,58	0	-1,01	-0,01	NA	NA	0	2,90	1,54	1
CH25H	cholesterol 25-hydroxylase	0,69	-0,53	0,16	0,57	0	-1,01	-0,01	NA	NA	0	1,96	0,97	1
CYP1B1	cytochrome P450, family 1, subfamily B, polypeptide 1	0,32	-1,65	0,01	0,17	0	-1,11	-0,15	0,73	1,00	0	2,05	1,04	1
CCL5	chemokine (C-C motif) ligand 5	NA	NA	NA	NA	0	-2,23	-1,16	0,01	0,74	0	NA	NA	0
TOMM20	translocase of outer mitochondrial membrane 20 homolog (yeast)	NA	NA	NA	NA	0	-1,01	-0,01	0,91	1,00	0	NA	NA	0
GPNMB	glycoprotein (transmembrane) nmb	NA	NA	NA	NA	0	-1,34	-0,42	0,27	1,00	0	NA	NA	0
SLC29A1	solute carrier family 29 (nucleoside transporters), member 1	NA	NA	NA	NA	0	-2,10	-1,07	0,31	1,00	0	NA	NA	0
GRK6	G protein-coupled receptor kinase 6	NA	NA	NA	NA	0	-1,03	-0,05	0,79	1,00	0	1,25	0,32	0
CD97	CD97 molecule	NA	NA	NA	NA	0	1,10	0,13	0,64	1,00	0	-1,06	-0,09	0
CYBA	cytochrome b-245, alpha polypeptide	NA	NA	NA	NA	0	1,33	0,41	0,14	1,00	0	NA	NA	0
THBS2	thrombospondin 2	NA	NA	NA	NA	0	-1,20	-0,26	0,57	1,00	0	-1,19	-0,26	0
ITGB1BP1	integrin beta 1 binding protein 1	NA	NA	NA	NA	0	1,01	0,01	0,91	1,00	0	NA	NA	0
ETV5	ets variant 5	NA	NA	NA	NA	0	-1,47	-0,56	0,12	1,00	0	NA	NA	0
FGFR2	fibroblast growth factor receptor 2	NA	NA	NA	NA	0	-1,12	-0,16	0,64	1,00	0	NA	NA	0
RFC3	replication factor C (activator 1) 3, 38kDa	NA	NA	NA	NA	0	1,19	0,26	0,52	1,00	0	-1,23	-0,30	0
VDR	vitamin D (1,25- dihydroxyvitamin D3) receptor	NA	NA	NA	NA	0	-1,58	-0,66	0,05	1,00	0	1,42	0,51	0

PKMYT1	protein kinase, membrane associated tyrosine/threonine 1	NA	NA	NA	NA	0	1,13	0,18	0,24	1,00	0	NA	NA	0
GTSE1	G-2 and S-phase expressed 1	NA	NA	NA	NA	0	1,00	0,00	0,99	1,00	0	NA	NA	0
C15orf39	chromosome 15 open reading frame 39	NA	NA	NA	NA	0	1,16	0,21	0,16	1,00	0	NA	NA	0
TROAP	trophinin associated protein (tastin)	NA	NA	NA	NA	0	1,19	0,25	0,13	1,00	0	NA	NA	0
RRAD	Ras-related associated with diabetes	NA	NA	NA	NA	0	1,39	0,47	0,53	1,00	0	NA	NA	0
GLDC	glycine dehydrogenase (decarboxylating)	NA	NA	NA	NA	0	1,22	0,29	0,13	1,00	0	NA	NA	0
TYMP	thymidine phosphorylase	NA	NA	NA	NA	0	1,50	0,58	0,18	1,00	0	1,01	0,01	0
CDC25C	cell division cycle 25 homolog C (S. pombe)	NA	NA	NA	NA	0	-1,20	-0,27	0,33	1,00	0	NA	NA	0
SPEG	SPEG complex locus	NA	NA	NA	NA	0	-1,25	-0,32	0,07	1,00	0	1,57	0,65	0
SLC44A4	solute carrier family 44, member 4	NA	NA	NA	NA	0	1,01	0,02	0,90	1,00	0	NA	NA	0
CCK	cholecystokinin	NA	NA	NA	NA	0	-1,02	-0,03	0,95	1,00	0	NA	NA	0
HSD17B1	hydroxysteroid (17-beta) dehydrogenase 1	NA	NA	NA	NA	0	1,04	0,06	0,82	1,00	0	NA	NA	0
HIST1H4C	histone cluster 1, H4c	NA	NA	NA	NA	0	1,19	0,25	0,34	1,00	0	NA	NA	0
B4GALT6	UDP-Gal:betaGlcNAc beta 1,4- galactosyltransferase, polypeptide 6	NA	NA	NA	NA	0	-1,18	-0,23	0,62	1,00	0	NA	NA	0
GNAL	guanine nucleotide binding protein (G protein), alpha activating activity polypeptide, olfactory type	NA	NA	NA	NA	0	-1,29	-0,37	0,65	1,00	0	NA	NA	0
KIF14	kinesin family member 14	NA	NA	NA	NA	0	-1,09	-0,12	0,48	1,00	0	NA	NA	0
LPAR2	lysophosphatidic acid receptor 2	NA	NA	NA	NA	0	-1,02	-0,03	0,90	1,00	0	NA	NA	0
HOXA7	homeobox A7	NA	NA	NA	NA	0	1,01	0,02	0,91	1,00	0	NA	NA	0
CDH4	cadherin 4, type 1, R-cadherin (retinal)	NA	NA	NA	NA	0	-1,06	-0,08	0,77	1,00	0	NA	NA	0
CFHR2	complement factor H-related 2	NA	NA	NA	NA	0	-1,02	-0,03	0,84	1,00	0	-1,09	-0,13	0
IL12RB2	interleukin 12 receptor, beta 2	NA	NA	NA	NA	0	-1,11	-0,15	0,46	1,00	0	NA	NA	0
HSF2BP	heat shock transcription factor 2 binding protein	NA	NA	NA	NA	0	1,20	0,26	0,50	1,00	0	NA	NA	0
SLC7A11	solute carrier family 7, (cationic amino acid transporter, y+ system) member 11	NA	NA	NA	NA	0	-1,55	-0,64	0,19	1,00	0	NA	NA	0
CENPI	centromere protein I	NA	NA	NA	NA	0	-1,24	-0,31	0,26	1,00	0	NA	NA	0
ARTN	artemin	NA	NA	NA	NA	0	-1,03	-0,04	0,88	1,00	0	NA	NA	0
ARHGEF16	Rho guanine nucleotide exchange factor (GEF) 16	NA	NA	NA	NA	0	1,12	0,17	0,40	1,00	0	NA	NA	0
CPVL	carboxypeptidase, vitellogenic-like	NA	NA	NA	NA	0	-1,25	-0,32	0,10	1,00	0	NA	NA	0
HIST1H4H	histone cluster 1, H4h	NA	NA	NA	NA	0	1,64	0,71	0,04	1,00	0	NA	NA	0
NRG1	neuregulin 1	NA	NA	NA	NA	0	1,11	0,15	0,69	1,00	0	NA	NA	0
NRG1	neuregulin 1	NA	NA	NA	NA	0	-1,02	-0,03	0,92	1,00	0	NA	NA	0
BRCA2	breast cancer 2, early onset	NA	NA	NA	NA	0	-1,07	-0,09	0,63	1,00	0	NA	NA	0
ATM	ataxia telangiectasia mutated	NA	NA	NA	NA	0	-1,78	-0,83	0,21	1,00	0	NA	NA	0
NA	NA	NA	NA	NA	NA	0	-1,00	0,00	1,00	1,00	0	0,00	0,01	0
HNRNPH3	heterogeneous nuclear ribonucleoprotein H3 (2H9)	NA	NA	NA	NA	0	1,01	0,01	0,93	1,00	0	NA	NA	0
PATZ1	POZ (BTB) and AT hook containing zinc finger 1	NA	NA	NA	NA	0	-1,45	-0,54	0,08	1,00	0	NA	NA	0
ALDH1B1	aldehyde dehydrogenase 1 family, member B1	NA	NA	NA	NA	0	-1,29	-0,37	0,24	1,00	0	-1,28	-0,36	0
SLC35D1	solute carrier family 35 (UDP-glucuronic acid/UDP-N-acetylgalactosamine dual transporter), member D1	NA	NA	NA	NA	0	-1,07	-0,10	0,61	1,00	0	NA	NA	0
HLA-DRB4	major histocompatibility complex, class II, DR beta 4	NA	NA	NA	NA	0	-1,01	-0,01	0,99	1,00	0	0,00	0,01	0
ABCG2	ATP-binding cassette, sub-family G (WHITE), member 2	NA	NA	NA	NA	0	1,01	0,02	0,92	1,00	0	NA	NA	0
HLA-DQB1	major histocompatibility complex, class II, DQ beta 1	NA	NA	NA	NA	0	-1,01	-0,01	0,99	1,00	0	NA	NA	0
CDT1	chromatin licensing and DNA replication factor 1	NA	NA	NA	NA	0	-1,56	-0,64	0,01	0,74	0	NA	NA	0
SELPLG	selectin P ligand	NA	NA	NA	NA	0	-1,00	0,00	1,00	1,00	0	-1,01	-0,01	0
SHMT1	serine hydroxymethyltransferase 1 (soluble)	NA	NA	NA	NA	0	-1,07	-0,10	0,73	1,00	0	NA	NA	0
SOCS1	suppressor of cytokine signaling 1	NA	NA	NA	NA	0	-1,00	-0,01	0,98	1,00	0	NA	NA	0
ABHD14A	abhydrolase domain containing 14A	NA	NA	NA	NA	0	-1,04	-0,06	0,87	1,00	0	NA	NA	0
ART3	ADP-ribosyltransferase 3	NA	NA	NA	NA	0	-1,01	-0,01	0,98	1,00	0	NA	NA	0
STX11	syntaxin 11	NA	NA	NA	NA	0	-1,00	-0,01	0,98	1,00	0	NA	NA	0
SP100	SP100 nuclear antigen	NA	NA	NA	NA	0	1,19	0,25	0,73	1,00	0	-1,11	-0,16	0
RASSF9	Ras association (RalGDS/AF-6) domain family (N-terminal) member 9	NA	NA	NA	NA	0	-1,20	-0,26	0,28	1,00	0	NA	NA	0
PML	promyelocytic leukemia	NA	NA	NA	NA	0	-1,14	-0,19	0,58	1,00	0	1,04	0,06	0
MR1	major histocompatibility complex, class I-related	NA	NA	NA	NA	0	1,09	0,12	0,70	1,00	0	NA	NA	0
AKAP1	A kinase (PRKA) anchor protein 1	NA	NA	NA	NA	0	1,05	0,08	0,80	1,00	0	NA	NA	0
PCDH7	protocadherin 7	NA	NA	NA	NA	0	-1,01	-0,02	0,94	1,00	0	NA	NA	0
COL4A6	collagen, type IV, alpha 6	NA	NA	NA	NA	0	1,05	0,07	0,76	1,00	0	NA	NA	0
NF2	neurofibromin 2 (merlin)	NA	NA	NA	NA	0	-1,08	-0,11	0,79	1,00	0	NA	NA	0
PLK4	polo-like kinase 4	NA	NA	NA	NA	0	-1,59	-0,67	0,26	1,00	0	-1,30	-0,37	0
KITLG	KIT ligand	NA	NA	NA	NA	0	-1,43	-0,52	0,04	1,00	0	-1,10	-0,14	0
HSF2	heat shock transcription factor 2	NA	NA	NA	NA	0	1,03	0,05	0,81	1,00	0	NA	NA	0
COL4A6	collagen, type IV, alpha 6	NA	NA	NA	NA	0	-1,31	-0,38	0,27	1,00	0	NA	NA	0
OGFR	opioid growth factor receptor	NA	NA	NA	NA	0	-1,08	-0,11	0,51	1,00	0	1,03	0,04	0
GRK6	G protein-coupled receptor kinase 6	NA	NA	NA	NA	0	-1,10	-0,14	0,70	1,00	0	NA	NA	0
NA	NA	NA	NA	NA	NA	0	-1,02	-0,03	0,95	1,00	0	NA	NA	0
PTPRO	protein tyrosine phosphatase, receptor type, O	NA	NA	NA	NA	0	-1,21	-0,28	0,29	1,00	0	NA	NA	0
KLF6	Kruppel-like factor 6	NA	NA	NA	NA	0	-1,01	-0,02	0,91	1,00	0	NA	NA	0
HLA-DQB1	major histocompatibility complex, class II, DQ beta 1	NA	NA	NA	NA	0	-1,00	0,00	0,98	1,00	0	NA	NA	0
HLA-DQB1	major histocompatibility complex, class II, DQ beta 1	NA	NA	NA	NA	0	-1,00	-0,01	0,99	1,00	0	NA	NA	0
ZNF551	zinc finger protein 551	NA	NA	NA	NA	0	1,03	0,04	0,78	1,00	0	NA	NA	0
GINS4	GINS complex subunit 4 (Sld5 homolog)	NA	NA	NA	NA	0	-1,61	-0,69	0,25	1,00	0	-1,41	-0,49	0
B3GALNT1	beta-1,3-N-acetylgalactosaminyltransferase 1 (globoside blood group)	NA	NA	NA	NA	0	1,25	0,32	0,26	1,00	0	-1,49	-0,58	0
CCNE2	cyclin E2	NA	NA	NA	NA	0	1,06	0,08	0,88	1,00	0	-1,35	-0,44	0
BRCA1	breast cancer 1, early onset	NA	NA	NA	NA	0	1,16	0,21	0,48	1,00	0	NA	NA	0
MKI67	antigen identified by monoclonal antibody Ki-67	NA	NA	NA	NA	0	-1,00	-0,01	0,98	1,00	0	NA	NA	0
TMEM97	transmembrane protein 97	NA	NA	NA	NA	0	-1,03	-0,05	0,90	1,00	0	NA	NA	0
MXD4	MAX dimerization protein 4	NA	NA	NA	NA	0	1,09	0,13	0,53	1,00	0	1,01	0,01	0
NA	NA	NA	NA	NA	NA	0	-1,01	-0,02	0,97	1,00	0	NA	NA	0
TCF7L2	transcription factor 7-like 2 (T-cell specific, HMG-box)	NA	NA	NA	NA	0	-1,26	-0,33	0,09	1,00	0	NA	NA	0
NCAPD3	non-SMC condensin II complex, subunit D3	NA	NA	NA	NA	0	1,03	0,05	0,68	1,00	0	NA	NA	0
PAXIP1	PAX interacting (with transcription-activation domain) protein 1	NA	NA	NA	NA	0	1,04	0,05	0,61	1,00	0	NA	NA	0
NCAPH	non-SMC condensin I complex, subunit H	NA	NA	NA	NA	0	1,11	0,15	0,57	1,00	0	NA	NA	0
HLA-DQB1	major histocompatibility complex, class II, DQ beta 1	NA	NA	NA	NA	0	-1,00	0,00	1,00	1,00	0	NA	NA	0
NPHP4	nephronophthisis 4	NA	NA	NA	NA	0	-1,12	-0,16	0,39	1,00	0	NA	NA	0
OIP5	Opa interacting protein 5	NA	NA	NA	NA	0	1,13	0,18	0,40	1,00	0	NA	NA	0
GNAL	guanine nucleotide binding protein (G protein), alpha activating activity polypeptide, olfactory type	NA	NA	NA	NA	0	-1,07	-0,09	0,93	1,00	0	NA	NA	0
DHRS2	dehydrogenase/reductase (SDR family) member 2	NA	NA	NA	NA	0	-1,02	-0,02	0,95	1,00	0	NA	NA	0
GAL	galanin prepropeptide	NA	NA	NA	NA	0	1,06	0,09	0,58	1,00	0	NA	NA	0
NA	NA	NA	NA	NA	NA	0	-1,00	-0,01	0,93	1,00	0	0,00	0,00	0
ETS1	v-ets erythroblastosis virus E26 oncogene homolog 1 (avian)	NA	NA	NA	NA	0	-1,00	0,00	0,99	1,00	0	NA	NA	0
SOCS6	suppressor of cytokine signaling 6	NA	NA	NA	NA	0	1,10	0,13	0,62	1,00	0	NA	NA	0
SERPINB10	serpin peptidase inhibitor, clade B (ovalbumin), member 10	NA	NA	NA	NA	0	1,08	0,12	0,88	1,00	0	NA	NA	0
BRCA2	breast cancer 2, early onset	NA	NA	NA	NA	0	-1,17	-0,22	0,64	1,00	0	NA	NA	0
NA	NA	NA	NA	NA	NA	0	-1,03	-0,04	0,87	1,00	0	1,01	0,02	0
FBXL18	F-box and leucine-rich repeat protein 18	NA	NA	NA	NA	0	-1,24	-0,31	0,04	1,00	0	NA	NA	0
SENP3	SUMO1/sentrin/SMT3 specific peptidase 3	NA	NA	NA	NA	0	-1,02	-0,04	0,91	1,00	0	-1,02	-0,03	0
HSDL2	hydroxysteroid dehydrogenase like 2	NA	NA	NA	NA	0	-1,36	-0,45	0,13	1,00	0	NA	NA	0
SAMD4A	sterile alpha motif domain containing 4A	NA	NA	NA	NA	0	1,01	0,02	0,93	1,00	0	NA	NA	0
TUBGCP3	tubulin, gamma complex associated protein 3	NA	NA	NA	NA	0	-1,20	-0,27	0,57	1,00	0	-1,14	-0,19	0
GTSE1	G-2 and S-phase expressed 1	NA	NA	NA	NA	0	1,03	0,05	0,78	1,00	0	NA	NA	0
ARTN	artemin	NA	NA	NA	NA	0	1,03	0,05	0,80	1,00	0	NA	NA	0
PDGFB	platelet-derived growth factor beta polypeptide (simian sarcoma viral (v-sis) oncogene homolog)	NA	NA	NA	NA	0	-1,01	-0,02	0,95	1,00	0	NA	NA	0
MED13L	mediator complex subunit 13-like	NA	NA	NA	NA	0	-1,10	-0,14	0,63	1,00	0	NA	NA	0
SYNJ2	synaptojanin 2	NA	NA	NA	NA	0	-1,00	0,00	0,99	1,00	0	NA	NA	0
SLC7A8	solute carrier family 7 (amino acid transporter, L-type), member 8	NA	NA	NA	NA	0	-2,15	-1,11	0,21	1,00	0	NA	NA	0
B4GALT1	UDP-Gal:betaGlcNAc beta 1,4- galactosyltransferase, polypeptide 1	NA	NA	NA	NA	0	-1,09	-0,12	0,84	1,00	0	NA	NA	0
PHTF2	putative homeodomain transcription factor 2	NA	NA	NA	NA	0	-1,92	-0,94	0,24	1,00	0	-1,35	-0,44	0
STAT2	signal transducer and activator of transcription 2, 113kDa	NA	NA	NA	NA	0	-1,01	-0,01	0,97	1,00	0	NA	NA	0
NCR2	natural cytotoxicity triggering receptor 2	NA	NA	NA	NA	0	1,08	0,11	0,50	1,00	0	NA	NA	0
TIMM50	translocase of inner mitochondrial membrane 50 homolog (S. cerevisiae)	NA	NA	NA	NA	0	1,12	0,16	0,54	1,00	0	NA	NA	0
SKA1	spindle and kinetochore associated complex subunit 1	NA	NA	NA	NA	0	-1,07	-0,10	0,78	1,00	0	NA	NA	0
NR1H2	nuclear receptor subfamily 1, group H, member 2	NA	NA	NA	NA	0	1,28	0,35	0,13	1,00	0	NA	NA	0
NUBP2	nucleotide binding protein 2 (MinD homolog, E. coli)	NA	NA	NA	NA	0	1,03	0,05	0,74	1,00	0	NA	NA	0
SLC35C1	solute carrier family 35, member C1	NA	NA	NA	NA	0	-1,05	-0,07	0,69	1,00	0	NA	NA	0
SYBU	syntabulin (syntaxin-interacting)	NA	NA	NA	NA	0	-1,19	-0,26	0,39	1,00	0	NA	NA	0
TSPAN15	tetraspanin 15	NA	NA	NA	NA	0	-1,03	-0,04	0,95	1,00	0	NA	NA	0
SDPR	serum deprivation response	NA	NA	NA	NA	0	-1,38	-0,47	0,06	1,00	0	NA	NA	0
ANO1	anoctamin 1, calcium activated chloride channel	NA	NA	NA	NA	0	-1,54	-0,63	0,08	1,00	0	NA	NA	0
FBXL15	F-box and leucine-rich repeat protein 15	NA	NA	NA	NA	0	1,06	0,08	0,69	1,00	0	NA	NA	0
PTER	phosphotriesterase related	NA	NA	NA	NA	0	-1,18	-0,24	0,16	1,00	0	NA	NA	0
MANEA	mannosidase, endo-alpha	NA	NA	NA	NA	0	-1,02	-0,02	0,96	1,00	0	-1,23	-0,31	0
LPHN1	latrophilin 1	NA	NA	NA	NA	0	-1,20	-0,26	0,31	1,00	0	NA	NA	0
NA	NA	NA	NA	NA	NA	0	1,06	0,09	0,77	1,00	0	1,18	0,24	0
ERCC6L	excision repair cross-complementing rodent repair deficiency, complementation group 6-like	NA	NA	NA	NA	0	1,14	0,18	0,34	1,00	0	NA	NA	0
NUDT18	nudix (nucleoside diphosphate linked moiety X)-type motif 18	NA	NA	NA	NA	0	-1,00	0,00	0,98	1,00	0	NA	NA	0
TMEM149	transmembrane protein 149	NA	NA	NA	NA	0	-1,00	0,00	1,00	1,00	0	-1,14	-0,18	0
LTBP3	latent transforming growth factor beta binding protein 3	NA	NA	NA	NA	0	-1,09	-0,12	0,58	1,00	0	NA	NA	0
PDCD1LG2	programmed cell death 1 ligand 2	NA	NA	NA	NA	0	-1,00	-0,01	0,97	1,00	0	-1,11	-0,15	0
C12orf48	chromosome 12 open reading frame 48	NA	NA	NA	NA	0	-1,13	-0,18	0,24	1,00	0	NA	NA	0
CCDC134	coiled-coil domain containing 134	NA	NA	NA	NA	0	-1,01	-0,01	0,96	1,00	0	NA	NA	0
DLK2	delta-like 2 homolog (Drosophila)	NA	NA	NA	NA	0	-1,09	-0,12	0,50	1,00	0	NA	NA	0
TRIM17	tripartite motif-containing 17	NA	NA	NA	NA	0	-1,02	-0,03	0,88	1,00	0	NA	NA	0
DEPDC1	DEP domain containing 1	NA	NA	NA	NA	0	-1,02	-0,03	0,95	1,00	0	NA	NA	0
NUP62CL	nucleoporin 62kDa C-terminal like	NA	NA	NA	NA	0	1,04	0,06	0,75	1,00	0	NA	NA	0
FAR2	fatty acyl CoA reductase 2	NA	NA	NA	NA	0	-1,19	-0,25	0,37	1,00	0	NA	NA	0
MCM10	minichromosome maintenance complex component 10	NA	NA	NA	NA	0	1,03	0,04	0,86	1,00	0	NA	NA	0
CIDEB	cell death-inducing DFFA-like effector b	NA	NA	NA	NA	0	-1,23	-0,29	0,13	1,00	0	NA	NA	0
SERINC3	serine incorporator 3	NA	NA	NA	NA	0	-1,16	-0,22	0,12	1,00	0	NA	NA	0
NA	NA	NA	NA	NA	NA	0	-1,01	-0,01	0,98	1,00	0	NA	NA	0
TAF9B	TAF9B RNA polymerase II, TATA box binding protein (TBP)-associated factor, 31kDa	NA	NA	NA	NA	0	-1,16	-0,21	0,75	1,00	0	-1,43	-0,52	0

EHBP1L1	EH domain binding protein 1-like 1	NA	NA	NA	NA	0	-1,04	-0,05	0,87	1,00	0	NA	NA	0
IL27RA	interleukin 27 receptor, alpha	NA	NA	NA	NA	0	-1,51	-0,59	0,22	1,00	0	-1,23	-0,30	0
CX3CL1	chemokine (C-X3-C motif) ligand 1	NA	NA	NA	NA	0	-1,00	0,00	0,99	1,00	0	NA	NA	0
EHBP1L1	EH domain binding protein 1-like 1	NA	NA	NA	NA	0	-1,04	-0,06	0,90	1,00	0	NA	NA	0
S1PR1	sphingosine-1-phosphate receptor 1	NA	NA	NA	NA	0	-1,04	-0,06	0,64	1,00	0	-1,16	-0,22	0
ITGA4	integrin, alpha 4 (antigen CD49D, alpha 4 subunit of VLA-4 receptor)	NA	NA	NA	NA	0	-1,01	-0,02	NA	NA	0	-1,41	-0,49	0
ZNF512B	zinc finger protein 512B	NA	NA	NA	NA	0	-1,07	-0,10	0,48	1,00	0	-1,04	-0,06	0
MET	met proto-oncogene (hepatocyte growth factor receptor)	NA	NA	NA	NA	0	1,59	0,67	0,60	1,00	0	-1,47	-0,55	0
FAM107A	family with sequence similarity 107, member A	NA	NA	NA	NA	0	-1,01	-0,01	0,93	1,00	0	0,00	0,01	0
ANGPT1	angiopoietin 1	NA	NA	NA	NA	0	-1,01	-0,01	NA	NA	0	-1,79	-0,83	0
PIP5K1A	phosphatidylinositol-4-phosphate 5-kinase, type I, alpha	NA	NA	NA	NA	0	-1,44	-0,53	0,52	1,00	0	-1,19	-0,24	0
CCDC28B	coiled-coil domain containing 28B	NA	NA	NA	NA	0	-1,15	-0,20	0,38	1,00	0	-1,05	-0,07	0
THOP1	thimet oligopeptidase 1	NA	NA	NA	NA	0	1,10	0,14	0,27	1,00	0	1,51	0,59	0
CNN1	calponin 1, basic, smooth muscle	NA	NA	NA	NA	0	-1,01	-0,01	NA	NA	0	1,33	0,41	0
NKX3-1	NK3 homeobox 1	NA	NA	NA	NA	0	1,08	0,12	0,40	1,00	0	1,23	0,29	0
ORAI2	ORAI calcium release-activated calcium modulator 2	NA	NA	NA	NA	0	-1,00	0,00	NA	NA	0	1,07	0,10	0
GFRA1	GDNF family receptor alpha 1	NA	NA	NA	NA	0	-1,01	-0,02	NA	NA	0	-1,32	-0,39	0
HSD17B6	hydroxysteroid (17-beta) dehydrogenase 6 homolog (mouse)	NA	NA	NA	NA	0	-1,00	-0,01	NA	NA	0	-1,16	-0,22	0
WSB2	WD repeat and SOCS box-containing 2	NA	NA	NA	NA	0	1,05	0,06	0,50	1,00	0	1,06	0,08	0
NA	NA	NA	NA	NA	NA	0	-1,44	-0,53	0,26	1,00	0	-1,39	-0,48	0
CSNK1G2	casein kinase 1, gamma 2	NA	NA	NA	NA	0	-1,19	-0,25	0,43	1,00	0	1,69	0,76	0
GABBR2	gamma-aminobutyric acid (GABA) B receptor, 2	NA	NA	NA	NA	0	-1,01	-0,01	NA	NA	0	-1,37	-0,45	0
IL7	interleukin 7	NA	NA	NA	NA	0	-1,01	-0,02	0,88	1,00	0	-1,16	-0,22	0
GNL3L	guanine nucleotide binding protein-like 3 (nucleolar)-like	NA	NA	NA	NA	0	1,07	0,09	0,44	1,00	0	-1,01	-0,02	0
OLR1	oxidized low density lipoprotein (lectin-like) receptor 1	NA	NA	NA	NA	0	-1,01	-0,01	NA	NA	0	-1,11	-0,16	0
CLCN7	chloride channel 7	NA	NA	NA	NA	0	1,11	0,15	0,22	1,00	0	1,20	0,26	0
TBC1D5	TBC1 domain family, member 5	NA	NA	NA	NA	0	-1,01	-0,02	0,92	1,00	0	-1,14	-0,18	0
LMAN1	lectin, mannose-binding, 1	NA	NA	NA	NA	0	-1,47	-0,56	0,64	1,00	0	-1,19	-0,25	0
RNFT1	ring finger protein, transmembrane 1	NA	NA	NA	NA	0	1,02	0,03	0,94	1,00	0	-1,37	-0,46	0
EPAS1	endothelial PAS domain protein 1	NA	NA	NA	NA	0	-1,13	-0,18	0,68	1,00	0	1,18	0,24	0
NA	NA	NA	NA	NA	NA	0	-1,03	-0,04	NA	NA	0	-1,32	-0,40	0
CCDC81	coiled-coil domain containing 81	NA	NA	NA	NA	0	-1,01	-0,01	NA	NA	0	1,09	0,12	0
MRPS12	mitochondrial ribosomal protein S12	NA	NA	NA	NA	0	1,41	0,50	0,17	1,00	0	-1,11	-0,16	0
BET1L	blocked early in transport 1 homolog (S. cerevisiae)-like	NA	NA	NA	NA	0	-1,00	0,00	NA	NA	0	-1,09	-0,13	0
HIPK3	homeodomain interacting protein kinase 3	NA	NA	NA	NA	0	-1,31	-0,39	0,66	1,00	0	-1,61	-0,68	0
ASPH	aspartate beta-hydroxylase	NA	NA	NA	NA	0	-1,01	-0,01	0,93	1,00	0	1,04	0,06	0
SIX1	SIX homeobox 1	NA	NA	NA	NA	0	-1,01	-0,01	NA	NA	0	-1,01	-0,02	0
YTHDC2	YTH domain containing 2	NA	NA	NA	NA	0	-1,05	-0,07	0,73	1,00	0	1,01	0,02	0
EEA1	early endosome antigen 1	NA	NA	NA	NA	0	1,14	0,19	0,73	1,00	0	-1,43	-0,51	0
SKP2	S-phase kinase-associated protein 2 (p45)	NA	NA	NA	NA	0	-1,59	-0,67	0,55	1,00	0	-1,47	-0,55	0
LIMS2	LIM and senescent cell antigen-like domains 2	NA	NA	NA	NA	0	-1,00	0,00	NA	NA	0	1,44	0,53	0
ENOX1	ecto-NOX disulfide-thiol exchanger 1	NA	NA	NA	NA	0	-1,02	-0,02	NA	NA	0	1,01	0,02	0
TAF6L	TAF6-like RNA polymerase II, p300/CBP-associated factor (PCAF)-associated factor, 65kDa	NA	NA	NA	NA	0	1,16	0,21	0,44	1,00	0	1,03	0,05	0
ITGA7	integrin, alpha 7	NA	NA	NA	NA	0	-1,01	-0,01	NA	NA	0	1,05	0,07	0
ITGA4	integrin, alpha 4 (antigen CD49D, alpha 4 subunit of VLA-4 receptor)	NA	NA	NA	NA	0	-1,01	-0,01	NA	NA	0	-1,41	-0,50	0
PLCB1	phospholipase C, beta 1 (phosphoinositide-specific)	NA	NA	NA	NA	0	-1,01	-0,01	NA	NA	0	-1,47	-0,55	0
KCNK2	potassium channel, subfamily K, member 2	NA	NA	NA	NA	0	-1,01	-0,01	NA	NA	0	-1,10	-0,13	0
NLGN1	neuroligin 1	NA	NA	NA	NA	0	-1,01	-0,01	NA	NA	0	-1,04	-0,05	0
SLC4A7	solute carrier family 4, sodium bicarbonate cotransporter, member 7	NA	NA	NA	NA	0	-1,58	-0,66	0,53	1,00	0	-1,09	-0,12	0
CDH2	cadherin 2, type 1, N-cadherin (neuronal)	NA	NA	NA	NA	0	-1,00	0,00	NA	NA	0	-1,27	-0,34	0
FRY	furry homolog (Drosophila)	NA	NA	NA	NA	0	-1,00	0,00	NA	NA	0	-1,11	-0,15	0
RAB6A	RAB6A, member RAS oncogene family	NA	NA	NA	NA	0	1,11	0,16	0,86	1,00	0	-1,22	-0,29	0
MBNL2	muscleblind-like 2 (Drosophila)	NA	NA	NA	NA	0	-1,04	-0,06	NA	NA	0	-1,14	-0,19	0
UTRN	utrophin	NA	NA	NA	NA	0	-1,00	0,00	NA	NA	0	-1,39	-0,47	0
CMAH	cytidine monophosphate-N-acetylneuraminic acid hydroxylase (CMP-N-acetylneuraminate monooxygenase) pseudogene	NA	NA	NA	NA	0	-1,01	-0,01	NA	NA	0	1,02	0,02	0
ACOT8	acyl-CoA thioesterase 8	NA	NA	NA	NA	0	1,03	0,04	0,73	1,00	0	3,02	1,59	0
NAB1	NGFI-A binding protein 1 (EGR1 binding protein 1)	NA	NA	NA	NA	0	-1,36	-0,44	0,28	1,00	0	1,31	0,39	0
B3GNT2	UDP-GlcNAc:betaGal beta-1,3-N-acetylglucosaminyltransferase 2	NA	NA	NA	NA	0	1,15	0,20	0,83	1,00	0	-1,39	-0,48	0
C19orf6	chromosome 19 open reading frame 6	NA	NA	NA	NA	0	-1,01	-0,01	0,97	1,00	0	0,00	0,00	0
C11orf75	chromosome 11 open reading frame 75	NA	NA	NA	NA	0	-1,10	-0,13	0,58	1,00	0	-1,43	-0,52	0
NA	NA	NA	NA	NA	NA	0	-1,01	-0,01	NA	NA	0	-1,47	-0,56	0
FBXW11	F-box and WD repeat domain containing 11	NA	NA	NA	NA	0	-1,08	-0,11	0,90	1,00	0	-1,28	-0,36	0
PIAS3	protein inhibitor of activated STAT, 3	NA	NA	NA	NA	0	1,01	0,01	0,95	1,00	0	-1,06	-0,10	0
LZTS1	leucine zipper, putative tumor suppressor 1	NA	NA	NA	NA	0	-1,01	-0,01	0,93	1,00	0	-1,28	-0,36	0
FGF1	fibroblast growth factor 1 (acidic)	NA	NA	NA	NA	0	-1,01	-0,01	NA	NA	0	-1,30	-0,38	0
NA	NA	NA	NA	NA	NA	0	-1,00	-0,01	NA	NA	0	-1,02	-0,04	0
PCDH8	protocadherin 8	NA	NA	NA	NA	0	-1,01	-0,01	NA	NA	0	1,22	0,29	0
SMAD3	SMAD family member 3	NA	NA	NA	NA	0	-1,03	-0,04	NA	NA	0	-1,19	-0,25	0
SCYL2	SCY1-like 2 (S. cerevisiae)	NA	NA	NA	NA	0	-1,49	-0,57	0,69	1,00	0	-1,28	-0,36	0
GPER	G protein-coupled estrogen receptor 1	NA	NA	NA	NA	0	-1,00	0,00	NA	NA	0	-1,02	-0,03	0
G3BP2	GTPase activating protein (SH3 domain) binding protein 2	NA	NA	NA	NA	0	-1,07	-0,09	0,85	1,00	0	-1,22	-0,30	0
PCLO	piccolo (presynaptic cytomatrix protein)	NA	NA	NA	NA	0	-1,01	-0,01	0,90	1,00	0	1,01	0,01	0
CLIC2	chloride intracellular channel 2	NA	NA	NA	NA	0	1,00	0,00	0,99	1,00	0	0,00	0,01	0
SGPP1	sphingosine-1-phosphate phosphatase 1	NA	NA	NA	NA	0	1,54	0,63	0,35	1,00	0	-1,10	-0,13	0
PITX1	paired-like homeodomain 1	NA	NA	NA	NA	0	1,67	0,74	0,12	1,00	0	1,92	0,94	0
FICD	FIC domain containing	NA	NA	NA	NA	0	-1,00	-0,01	0,96	1,00	0	-1,01	-0,02	0
TGFBR1	transforming growth factor, beta receptor 1	NA	NA	NA	NA	0	-1,26	-0,33	0,52	1,00	0	1,20	0,26	0
EXOC5	exocyst complex component 5	NA	NA	NA	NA	0	1,62	0,70	0,54	1,00	0	-1,45	-0,53	0
PRSS12	protease, serine, 12 (neurotrypsin, motopsin)	NA	NA	NA	NA	0	-1,05	-0,07	0,79	1,00	0	-1,54	-0,62	0
MET	met proto-oncogene (hepatocyte growth factor receptor)	NA	NA	NA	NA	0	1,33	0,41	0,75	1,00	0	-1,56	-0,64	0
HLX	H2.0-like homeobox	NA	NA	NA	NA	0	-1,01	-0,01	NA	NA	0	-1,09	-0,12	0
MXD4	MAX dimerization protein 4	NA	NA	NA	NA	0	-1,02	-0,02	NA	NA	0	-1,27	-0,34	0
TMEM160	transmembrane protein 160	NA	NA	NA	NA	0	-1,26	-0,34	0,36	1,00	0	1,02	0,03	0
FLI1	Friend leukemia virus integration 1	NA	NA	NA	NA	0	-1,01	-0,02	NA	NA	0	-1,37	-0,46	0
TRMT2B	TRM2 tRNA methyltransferase 2 homolog B (S. cerevisiae)	NA	NA	NA	NA	0	-1,07	-0,10	0,86	1,00	0	-1,09	-0,11	0
CDK17	cyclin-dependent kinase 17	NA	NA	NA	NA	0	-1,45	-0,54	0,25	1,00	0	-1,32	-0,40	0
OLFM4	olfactomedin 4	NA	NA	NA	NA	0	-1,01	-0,01	NA	NA	0	1,21	0,27	0
PCDH9	protocadherin 9	NA	NA	NA	NA	0	-1,01	-0,02	NA	NA	0	-2,27	-1,20	0
SSX2IP	synovial sarcoma, X breakpoint 2 interacting protein	NA	NA	NA	NA	0	-1,09	-0,13	0,59	1,00	0	-1,41	-0,50	0
MCAM	melanoma cell adhesion molecule	NA	NA	NA	NA	0	1,02	0,03	0,81	1,00	0	-1,06	-0,09	0
SRSF1	serine/arginine-rich splicing factor 1	NA	NA	NA	NA	0	-1,13	-0,17	0,09	1,00	0	1,01	0,02	0
MLLT10	myeloid/lymphoid or mixed-lineage leukemia (trithorax homolog, Drosophila); translocated to, 10	NA	NA	NA	NA	0	1,30	0,38	0,56	1,00	0	-1,04	-0,06	0
TRBC1	T cell receptor beta constant 1	NA	NA	NA	NA	0	-1,00	-0,01	NA	NA	0	-1,02	-0,02	0
TAP2	transporter 2, ATP-binding cassette, sub-family B (MDR/TAP)	NA	NA	NA	NA	0	-1,01	-0,01	NA	NA	0	0,00	0,00	0
CREBZF	CREB/ATF bZIP transcription factor	NA	NA	NA	NA	0	1,07	0,09	0,90	1,00	0	-1,49	-0,57	0
DBT	dihydrolipoamide branched chain transacylase E2	NA	NA	NA	NA	0	1,15	0,20	0,77	1,00	0	-1,49	-0,57	0
LMOD1	leiomodin 1 (smooth muscle)	NA	NA	NA	NA	0	-1,01	-0,01	NA	NA	0	1,30	0,38	0
ZNF22	zinc finger protein 22 (KOX 15)	NA	NA	NA	NA	0	-1,10	-0,14	0,77	1,00	0	-1,43	-0,51	0
HHEX	hematopoietically expressed homeobox	NA	NA	NA	NA	0	-1,01	-0,01	NA	NA	0	0,00	0,01	0
SLC1A1	solute carrier family 1 (neuronal/epithelial high affinity glutamate transporter, system Xag), member 1	NA	NA	NA	NA	0	-1,01	-0,02	NA	NA	0	-1,30	-0,37	0
NGF	nerve growth factor (beta polypeptide)	NA	NA	NA	NA	0	-1,00	0,00	NA	NA	0	1,60	0,68	0
KCTD20	potassium channel tetramerisation domain containing 20	NA	NA	NA	NA	0	-1,03	-0,04	0,68	1,00	0	1,01	0,02	0
PLCB4	phospholipase C, beta 4	NA	NA	NA	NA	0	-1,01	-0,01	NA	NA	0	1,06	0,08	0
L1CAM	L1 cell adhesion molecule	NA	NA	NA	NA	0	-1,01	-0,01	NA	NA	0	1,17	0,22	0
GALK1	galactokinase 1	NA	NA	NA	NA	0	-1,01	-0,01	NA	NA	0	1,18	0,24	0
UCP2	uncoupling protein 2 (mitochondrial, proton carrier)	NA	NA	NA	NA	0	1,00	0,00	NA	NA	0	1,54	0,62	0
WFDC1	WAP four-disulfide core domain 1	NA	NA	NA	NA	0	-1,01	-0,01	NA	NA	0	-1,67	-0,73	0
GNA13	guanine nucleotide binding protein (G protein), alpha 13	NA	NA	NA	NA	0	-1,43	-0,51	0,40	1,00	0	-1,12	-0,16	0
CSF1	colony stimulating factor 1 (macrophage)	NA	NA	NA	NA	0	-1,01	-0,01	NA	NA	0	1,01	0,02	0
PAPSS2	3'-phosphoadenosine 5'-phosphosulfate synthase 2	NA	NA	NA	NA	0	-1,01	-0,01	NA	NA	0	-1,19	-0,24	0
TRABD	TraB domain containing	NA	NA	NA	NA	0	1,22	0,29	0,34	1,00	0	1,46	0,55	0
NF1	neurofibromin 1	NA	NA	NA	NA	0	-1,02	-0,03	NA	NA	0	1,09	0,12	0
UNC93B1	unc-93 homolog B1 (C. elegans)	NA	NA	NA	NA	0	-1,01	-0,02	NA	NA	0	-1,01	-0,01	0
BMP4	bone morphogenetic protein 4	NA	NA	NA	NA	0	-1,01	-0,01	NA	NA	0	1,01	0,01	0
CD7	CD7 molecule	NA	NA	NA	NA	0	-1,01	-0,01	NA	NA	0	1,01	0,02	0
GREM2	gremlin 2	NA	NA	NA	NA	0	-1,00	0,00	NA	NA	0	-1,27	-0,34	0
ATP2B4	ATPase, Ca++ transporting, plasma membrane 4	NA	NA	NA	NA	0	-1,06	-0,08	0,65	1,00	0	1,28	0,36	0
KITLG	KIT ligand	NA	NA	NA	NA	0	1,05	0,08	0,79	1,00	0	-1,11	-0,15	0
MARK2	MAP/microtubule affinity-regulating kinase 2	NA	NA	NA	NA	0	-1,01	-0,02	0,83	1,00	0	1,02	0,03	0
TRPV2	transient receptor potential cation channel, subfamily V, member 2	NA	NA	NA	NA	0	-1,01	-0,01	NA	NA	0	1,01	0,01	0
GULP1	GULP, engulfment adaptor PTB domain containing 1	NA	NA	NA	NA	0	1,05	0,07	0,76	1,00	0	-1,43	-0,52	0
SOS1	son of sevenless homolog 1 (Drosophila)	NA	NA	NA	NA	0	-1,14	-0,19	0,51	1,00	0	-1,04	-0,06	0
TRIM23	tripartite motif-containing 23	NA	NA	NA	NA	0	-1,29	-0,36	0,13	1,00	0	-1,33	-0,42	0
MED16	mediator complex subunit 16	NA	NA	NA	NA	0	-1,07	-0,09	NA	NA	0	1,01	0,02	0
NR2F6	nuclear receptor subfamily 2, group F, member 6	NA	NA	NA	NA	0	1,03	0,04	0,82	1,00	0	1,25	0,33	0
FOSL2	FOS-like antigen 2	NA	NA	NA	NA	0	-1,11	-0,15	0,35	1,00	0	-1,14	-0,18	0
RB1	retinoblastoma 1	NA	NA	NA	NA	0	-1,07	-0,10	0,36	1,00	0	-1,20	-0,28	0
TLR4	toll-like receptor 4	NA	NA	NA	NA	0	-1,00	-0,01	NA	NA	0	-1,35	-0,42	0
AKT2	v-akt murine thymoma viral oncogene homolog 2	NA	NA	NA	NA	0	-1,01	-0,01	NA	NA	0	-1,01	-0,02	0
LMOD1	leiomodin 1 (smooth muscle)	NA	NA	NA	NA	0	-1,01	-0,01	NA	NA	0	1,43	0,51	0
HSD17B6	hydroxysteroid (17-beta) dehydrogenase 6 homolog (mouse)	NA	NA	NA	NA	0	-1,03	-0,04	NA	NA	0	-1,15	-0,20	0
SLAMF8	SLAM family member 8	NA	NA	NA	NA	0	-1,02	-0,02	NA	NA	0	1,01	0,02	0
ROCK2	Rho-associated, coiled-coil containing protein kinase 2	NA	NA	NA	NA	0	-1,42	-0,51	0,04	1,00	0	-1,20	-0,28	0
FNDC3A	fibronectin type III domain containing 3A	NA	NA	NA	NA	0	-1,11	-0,15	0,24	1,00	0	-1,33	-0,41	0
SH2B2	SH2B adaptor protein 2	NA	NA	NA	NA	0	1,00	0,00	0,98	1,00	0	1,06	0,08	0
LPIN1	lipin 1	NA	NA	NA	NA	0	1,39	0,47	0,59	1,00	0	-1,10	-0,14	0
PAWR	PRKC, apoptosis, WT1, regulator	NA	NA	NA	NA	0	1,21	0,27	0,12	1,00	0	-1,19	-0,25	0

SLC39A9	solute carrier family 39 (zinc transporter), member 9	NA	NA	NA	NA	0	-1,11	-0,16	0,65	1,00	0	-1,27	-0,34	0
BRIP1	BRCA1 interacting protein C-terminal helicase 1	NA	NA	NA	NA	0	-1,02	-0,03	0,87	1,00	0	-1,14	-0,18	0
TOR1AIP1	torsin A interacting protein 1	NA	NA	NA	NA	0	1,67	0,74	0,39	1,00	0	-1,01	-0,01	0
BATF3	basic leucine zipper transcription factor, ATF-like 3	NA	NA	NA	NA	0	-1,01	-0,01	NA	NA	0	1,18	0,24	0
CUEDC1	CUE domain containing 1	NA	NA	NA	NA	0	1,24	0,31	0,09	1,00	0	1,20	0,27	0
PRKCI	protein kinase C, iota	NA	NA	NA	NA	0	-1,22	-0,29	0,70	1,00	0	-1,64	-0,72	0
06-set	septin 6	NA	NA	NA	NA	0	-1,01	-0,01	NA	NA	0	-1,25	-0,32	0
ARG1	arginase, liver	10,16	3,35	0,01	0,22	0	1,44	0,52	0,46	1,00	0	NA	NA	0
OASL	2'-5'-oligoadenylate synthetase-like	3,71	1,89	0,01	0,20	0	1,13	0,18	0,61	1,00	0	1,14	0,19	0
KLK10	kallikrein-related peptidase 10	3,53	1,82	0,00	0,10	0	2,20	1,13	0,03	0,96	0	NA	NA	0
ISG20	interferon stimulated exonuclease gene 20kDa	3,00	1,59	0,02	0,25	0	1,02	0,02	0,91	1,00	0	0,00	0,00	0
RNF39	ring finger protein 39	2,99	1,58	0,00	0,11	0	1,07	0,10	0,78	1,00	0	NA	NA	0
OASL	2'-5'-oligoadenylate synthetase-like	2,96	1,57	0,02	0,26	0	-1,14	-0,19	0,72	1,00	0	-1,01	-0,01	0
ISG20	interferon stimulated exonuclease gene 20kDa	2,84	1,50	0,01	0,21	0	1,00	0,00	0,99	1,00	0	0,00	0,00	0
C18orf25	chromosome 18 open reading frame 25	2,70	1,43	0,01	0,17	0	1,00	0,00	1,00	1,00	0	-1,02	-0,03	0
NRG1	neuregulin 1	2,67	1,42	0,00	0,14	0	1,17	0,23	0,35	1,00	0	NA	NA	0
SERPINB1	serpin peptidase inhibitor, clade B (ovalbumin), member 1	2,57	1,36	0,07	0,42	0	1,22	0,29	0,25	1,00	0	NA	NA	0
PTPN12	protein tyrosine phosphatase, non-receptor type 12	2,56	1,36	0,01	0,18	0	1,40	0,48	0,53	1,00	0	1,20	0,26	0
CCL7	chemokine (C-C motif) ligand 7	2,45	1,30	0,14	0,54	0	-1,02	-0,03	0,83	1,00	0	1,68	0,74	0
ZNF426	zinc finger protein 426	2,39	1,26	0,01	0,22	0	1,31	0,39	0,18	1,00	0	1,02	0,02	0
FUT3	fucosyltransferase 3 (galactoside 3(4)-L-fucosyltransferase, Lewis blood group)	2,37	1,25	0,05	0,36	0	1,09	0,12	0,70	1,00	0	NA	NA	0
TSC22D2	TSC22 domain family, member 2	2,32	1,21	0,06	0,39	0	-1,01	-0,01	0,97	1,00	0	NA	NA	0
EGR1	early growth response 1	2,24	1,16	0,14	0,54	0	-1,04	-0,06	0,90	1,00	0	-1,08	-0,10	0
CEACAM1	carcinoembryonic antigen-related cell adhesion molecule 1 (biliary glycoprotein)	2,23	1,16	0,34	0,73	0	1,02	0,03	0,83	1,00	0	1,01	0,01	0
TGFA	transforming growth factor, alpha	2,20	1,14	0,00	0,11	0	1,26	0,33	0,05	1,00	0	NA	NA	0
ATP13A3	ATPase type 13A3	2,17	1,12	0,01	0,21	0	1,82	0,87	0,38	1,00	0	1,10	0,14	0
EGR3	early growth response 3	2,14	1,10	0,01	0,15	0	-1,03	-0,04	0,92	1,00	0	NA	NA	0
KRT34	keratin 34	2,14	1,10	0,01	0,16	0	2,05	1,03	0,00	0,26	0	1,14	0,19	0
BTBD7	BTB (POZ) domain containing 7	2,13	1,09	0,00	0,11	0	1,06	0,09	0,76	1,00	0	NA	NA	0
IL22RA1	interleukin 22 receptor, alpha 1	2,13	1,09	0,01	0,15	0	1,42	0,51	0,05	1,00	0	NA	NA	0
EHD1	EH-domain containing 1	2,12	1,08	0,00	0,14	0	1,00	0,01	0,98	1,00	0	NA	NA	0
INHBA	inhibin, beta A	2,10	1,07	0,08	0,44	0	1,22	0,28	0,27	1,00	0	-1,05	-0,07	0
GAB1	GRB2-associated binding protein 1	2,08	1,06	0,02	0,23	0	-1,01	-0,02	0,95	1,00	0	NA	NA	0
RPS6KA5	ribosomal protein S6 kinase, 90kDa, polypeptide 5	2,07	1,05	0,01	0,19	0	1,45	0,54	0,22	1,00	0	NA	NA	0
LYN	v-yes-1 Yamaguchi sarcoma viral related oncogene homolog	2,06	1,04	0,02	0,27	0	2,11	1,07	0,04	1,00	0	-1,02	-0,02	0
CEACAM1	carcinoembryonic antigen-related cell adhesion molecule 1 (biliary glycoprotein)	2,04	1,03	0,40	0,76	0	-1,01	-0,01	0,98	1,00	0	NA	NA	0
PRSS8	protease, serine, 8	2,04	1,03	0,01	0,15	0	-1,00	0,00	0,99	1,00	0	NA	NA	0
SOX9	SRY (sex determining region Y)-box 9	2,03	1,02	0,06	0,39	0	-1,24	-0,31	0,32	1,00	0	NA	NA	0
POLQ	polymerase (DNA directed), theta	2,02	1,01	0,01	0,20	0	1,20	0,27	0,38	1,00	0	NA	NA	0
BUB1	budding uninhibited by benzimidazoles 1 homolog (yeast)	2,00	1,00	0,02	0,24	0	1,02	0,03	0,84	1,00	0	NA	NA	0
STK38L	serine/threonine kinase 38 like	1,99	0,99	0,00	0,12	0	1,20	0,26	0,08	1,00	0	NA	NA	0
CYP3A5	cytochrome P450, family 3, subfamily A, polypeptide 5	1,98	0,99	0,02	0,28	0	1,01	0,02	0,94	1,00	0	NA	NA	0
ARHGAP25	Rho GTPase activating protein 25	1,97	0,98	0,00	0,10	0	1,18	0,24	0,50	1,00	0	NA	NA	0
CEACAM1	carcinoembryonic antigen-related cell adhesion molecule 1 (biliary glycoprotein)	1,97	0,98	0,49	0,81	0	1,01	0,02	0,86	1,00	0	0,00	0,00	0
AKR1C3	aldo-keto reductase family 1, member C3 (3-alpha hydroxysteroid dehydrogenase, type II)	1,95	0,96	0,01	0,20	0	1,65	0,72	0,12	1,00	0	-1,41	-0,50	0
FEM1B	fem-1 homolog b (C. elegans)	1,94	0,96	0,03	0,29	0	1,05	0,07	0,82	1,00	0	NA	NA	0
ALOXE3	arachidonate lipoxygenase 3	1,94	0,96	0,02	0,24	0	1,25	0,32	0,31	1,00	0	NA	NA	0
PRSS3	protease, serine, 3	1,94	0,95	0,01	0,15	0	1,12	0,17	0,30	1,00	0	-1,37	-0,45	0
EDN1	endothelin 1	1,92	0,94	0,04	0,35	0	1,33	0,41	0,13	1,00	0	-1,12	-0,17	0
KLF4	Kruppel-like factor 4 (gut)	1,91	0,93	0,01	0,17	0	1,37	0,45	0,16	1,00	0	-1,01	-0,01	0
MYO1B	myosin IB	1,90	0,93	0,03	0,31	0	-1,01	-0,01	0,97	1,00	0	NA	NA	0
DUSP1	dual specificity phosphatase 1	1,90	0,93	0,03	0,30	0	1,34	0,42	0,18	1,00	0	NA	NA	0
PRSS3	protease, serine, 3	1,90	0,92	0,00	0,11	0	1,23	0,30	0,07	1,00	0	-1,30	-0,38	0
NRIP1	nuclear receptor interacting protein 1	1,90	0,92	0,14	0,53	0	1,15	0,21	0,62	1,00	0	-1,72	-0,78	0
TANK	TRAF family member-associated NFKB activator	1,88	0,91	0,02	0,24	0	1,11	0,15	0,47	1,00	0	NA	NA	0
HS3ST1	heparan sulfate (glucosamine) 3-O-sulfotransferase 1	1,88	0,91	0,37	0,75	0	1,32	0,40	0,32	1,00	0	NA	NA	0
NRG1	neuregulin 1	1,86	0,90	0,05	0,35	0	1,58	0,66	0,16	1,00	0	NA	NA	0
NA	NA	1,86	0,89	0,02	0,24	0	1,01	0,01	0,97	1,00	0	NA	NA	0
PLAGL2	pleiomorphic adenoma gene-like 2	1,86	0,89	0,01	0,21	0	1,60	0,68	0,03	0,96	0	NA	NA	0
PICALM	phosphatidylinositol binding clathrin assembly protein	1,85	0,89	0,08	0,43	0	1,04	0,05	0,91	1,00	0	-1,12	-0,18	0
FZD10	frizzled homolog 10 (Drosophila)	1,85	0,89	0,01	0,17	0	-1,09	-0,13	0,76	1,00	0	NA	NA	0
PPP2R5B	protein phosphatase 2, regulatory subunit B', beta	1,85	0,89	0,01	0,20	0	1,37	0,45	0,09	1,00	0	NA	NA	0
EHD1	EH-domain containing 1	1,84	0,88	0,00	0,14	0	1,04	0,06	0,73	1,00	0	NA	NA	0
SMURF1	SMAD specific E3 ubiquitin protein ligase 1	1,84	0,88	0,02	0,23	0	-1,09	-0,12	0,59	1,00	0	NA	NA	0
KIF20A	kinesin family member 20A	1,84	0,88	0,04	0,34	0	-1,01	-0,01	0,93	1,00	0	NA	NA	0
EDEM3	ER degradation enhancer, mannosidase alpha-like 3	1,83	0,87	0,11	0,50	0	-1,21	-0,28	0,73	1,00	0	-1,52	-0,60	0
DLG1	discs, large homolog 1 (Drosophila)	1,83	0,87	0,06	0,39	0	1,11	0,14	0,84	1,00	0	-1,05	-0,07	0
FUT1	fucosyltransferase 1 (galactoside 2-alpha-L-fucosyltransferase, H blood group)	1,81	0,86	0,00	0,14	0	-1,01	-0,02	0,90	1,00	0	NA	NA	0
PHACTR4	phosphatase and actin regulator 4	1,81	0,86	0,03	0,31	0	1,20	0,26	0,12	1,00	0	-1,08	-0,10	0
PDZD2	PDZ domain containing 2	1,81	0,86	0,00	0,12	0	-1,11	-0,15	0,38	1,00	0	NA	NA	0
ACOT7	acyl-CoA thioesterase 7	1,81	0,85	0,00	0,13	0	-1,05	-0,06	0,58	1,00	0	NA	NA	0
MAPK7	mitogen-activated protein kinase 7	1,80	0,85	0,02	0,23	0	1,04	0,05	0,79	1,00	0	NA	NA	0
NA	NA	1,80	0,85	0,01	0,15	0	-1,01	-0,01	NA	NA	0	-1,01	-0,01	0
EDNRA	endothelin receptor type A	1,79	0,84	0,04	0,32	0	-1,01	-0,02	0,96	1,00	0	NA	NA	0
PHLDA2	pleckstrin homology-like domain, family A, member 2	1,79	0,84	0,00	0,12	0	1,00	0,00	0,98	1,00	0	NA	NA	0
TICAM1	toll-like receptor adaptor molecule 1	1,79	0,84	0,04	0,34	0	1,17	0,22	0,20	1,00	0	NA	NA	0
CRCT1	cysteine-rich C-terminal 1	1,79	0,84	0,03	0,28	0	1,91	0,94	0,02	0,84	0	NA	NA	0
MAOA	monoamine oxidase A	1,79	0,84	0,01	0,22	0	1,19	0,26	0,09	1,00	0	NA	NA	0
ARG2	arginase, type II	1,78	0,83	0,01	0,20	0	1,09	0,12	0,39	1,00	0	-1,12	-0,17	0
CBLC	Cas-Br-M (murine) ecotropic retroviral transforming sequence c	1,78	0,83	0,07	0,41	0	1,04	0,06	0,71	1,00	0	NA	NA	0
LOC100288939	similar to hCG1987955	1,78	0,83	0,02	0,28	0	-1,03	-0,05	0,88	1,00	0	NA	NA	0
KDM6B	lysine (K)-specific demethylase 6B	1,78	0,83	0,01	0,21	0	1,13	0,18	0,57	1,00	0	NA	NA	0
CIT	citron (rho-interacting, serine/threonine kinase 21)	1,77	0,83	0,02	0,24	0	1,02	0,03	0,78	1,00	0	NA	NA	0
SORL1	sortilin-related receptor, L(DLR class) A repeats-containing	1,77	0,82	0,02	0,27	0	-1,25	-0,32	0,09	1,00	0	NA	NA	0
CFLAR	CASP8 and FADD-like apoptosis regulator	1,77	0,82	0,00	0,12	0	1,24	0,31	0,17	1,00	0	NA	NA	0
MMP10	matrix metallopeptidase 10 (stromelysin 2)	1,77	0,82	0,02	0,23	0	3,78	1,92	0,01	0,48	0	NA	NA	0
MAOA	monoamine oxidase A	1,76	0,82	0,01	0,17	0	1,25	0,32	0,01	0,73	0	NA	NA	0
LRRN3	leucine rich repeat neuronal 3	1,76	0,82	0,23	0,64	0	-1,00	-0,01	NA	NA	0	1,23	0,30	0
PLA2G3	phospholipase A2, group III	1,76	0,82	0,01	0,15	0	1,63	0,70	0,02	0,88	0	NA	NA	0
UBE2H	ubiquitin-conjugating enzyme E2H (UBC8 homolog, yeast)	1,76	0,81	0,03	0,28	0	1,35	0,43	0,40	1,00	0	-1,01	-0,02	0
ALOX12	arachidonate 12-lipoxygenase	1,76	0,81	0,01	0,20	0	-1,02	-0,03	0,89	1,00	0	NA	NA	0
CYP26B1	cytochrome P450, family 26, subfamily B, polypeptide 1	1,75	0,81	0,01	0,22	0	1,00	0,00	1,00	1,00	0	NA	NA	0
TOP2A	topoisomerase (DNA) II alpha 170kDa	1,75	0,81	0,05	0,38	0	-1,16	-0,22	0,31	1,00	0	NA	NA	0
RRM2	ribonucleotide reductase M2	1,74	0,80	0,01	0,21	0	1,12	0,16	0,34	1,00	0	NA	NA	0
ACAD8	acyl-CoA dehydrogenase family, member 8	1,74	0,80	0,00	0,14	0	1,10	0,14	0,29	1,00	0	NA	NA	0
SERPINB1	serpin peptidase inhibitor, clade B (ovalbumin), member 1	1,74	0,80	0,06	0,39	0	1,14	0,19	0,57	1,00	0	NA	NA	0
NA	NA	1,74	0,80	0,04	0,33	0	-1,07	-0,10	0,62	1,00	0	NA	NA	0
MAP3K9	mitogen-activated protein kinase kinase kinase 9	1,74	0,79	0,02	0,22	0	1,04	0,06	0,82	1,00	0	NA	NA	0
RC3H2	ring finger and CCCH-type zinc finger domains 2	1,73	0,79	0,00	0,12	0	1,02	0,03	0,88	1,00	0	NA	NA	0
SPRY4	sprouty homolog 4 (Drosophila)	1,73	0,79	0,00	0,14	0	-1,10	-0,13	0,61	1,00	0	NA	NA	0
DOCK6	dedicator of cytokinesis 6	1,73	0,79	0,00	0,11	0	1,13	0,17	0,45	1,00	0	NA	NA	0
RANBP9	RAN binding protein 9	1,73	0,79	0,01	0,21	0	1,38	0,46	0,20	1,00	0	-1,47	-0,57	0
CAMSAP1L1	calmodulin regulated spectrin-associated protein 1-like 1	1,72	0,79	0,00	0,14	0	-1,52	-0,60	0,10	1,00	0	-1,32	-0,39	0
PPIF	peptidylprolyl isomerase F	1,72	0,78	0,00	0,11	0	1,48	0,57	0,02	0,94	0	NA	NA	0
ABCA12	ATP-binding cassette, sub-family A (ABC1), member 12	1,72	0,78	0,00	0,12	0	1,29	0,37	0,15	1,00	0	NA	NA	0
TGM2	transglutaminase 2 (C polypeptide, protein-glutamine-gamma-glutamyltransferase)	1,71	0,77	0,02	0,24	0	-1,17	-0,23	0,34	1,00	0	-1,10	-0,13	0
AURKB	aurora kinase B	1,71	0,77	0,02	0,24	0	1,17	0,22	0,34	1,00	0	NA	NA	0
SH3PXD2A	SH3 and PX domains 2A	1,71	0,77	0,01	0,16	0	-1,20	-0,26	0,47	1,00	0	NA	NA	0
PLAUR	plasminogen activator, urokinase receptor	1,71	0,77	0,01	0,16	0	1,41	0,50	0,12	1,00	0	NA	NA	0
SIKE1	suppressor of IKBKE 1	1,71	0,77	0,09	0,45	0	-1,31	-0,39	0,48	1,00	0	-1,54	-0,63	0
CLIC3	chloride intracellular channel 3	1,70	0,77	0,03	0,29	0	1,10	0,14	0,67	1,00	0	1,27	0,35	0
CITED2	Cbp/p300-interacting transactivator, with Glu/Asp-rich carboxy-terminal domain, 2	1,70	0,77	0,01	0,20	0	1,32	0,40	0,31	1,00	0	NA	NA	0
WNT7A	wingless-type MMTV integration site family, member 7A	1,70	0,77	0,01	0,15	0	-1,16	-0,21	0,26	1,00	0	NA	NA	0
DSG3	desmoglein 3	1,70	0,76	0,04	0,34	0	1,07	0,10	0,70	1,00	0	NA	NA	0
RBM47	RNA binding motif protein 47	1,70	0,76	0,03	0,29	0	1,56	0,64	0,01	0,48	0	NA	NA	0
CEACAM6	carcinoembryonic antigen-related cell adhesion molecule 6 (non-specific cross reacting antigen)	1,69	0,76	0,40	0,76	0	5,25	2,39	0,01	0,70	0	NA	NA	0
USP13	ubiquitin specific peptidase 13 (isopeptidase T-3)	1,69	0,75	0,01	0,18	0	1,13	0,18	0,38	1,00	0	-1,33	-0,42	0
INSIG1	insulin induced gene 1	1,69	0,75	0,01	0,17	0	1,16	0,22	0,37	1,00	0	NA	NA	0
ARHGAP25	Rho GTPase activating protein 25	1,68	0,75	0,01	0,17	0	1,27	0,34	0,11	1,00	0	NA	NA	0
GNA15	guanine nucleotide binding protein (G protein), alpha 15 (Gq class)	1,68	0,75	0,01	0,17	0	1,15	0,20	0,16	1,00	0	NA	NA	0
ALOX12B	arachidonate 12-lipoxygenase, 12R type	1,68	0,75	0,01	0,17	0	2,82	1,50	0,01	0,64	0	NA	NA	0
EHD1	EH-domain containing 1	1,68	0,75	0,01	0,20	0	1,02	0,02	0,86	1,00	0	NA	NA	0
PPARD	peroxisome proliferator-activated receptor delta	1,67	0,74	0,00	0,13	0	1,56	0,64	0,04	1,00	0	-1,03	-0,04	0
ULBP2	UL16 binding protein 2	1,67	0,74	0,00	0,10	0	1,21	0,27	0,35	1,00	0	1,11	0,15	0
PTPN11	protein tyrosine phosphatase, non-receptor type 11	1,67	0,74	0,03	0,29	0	-1,02	-0,03	0,96	1,00	0	-1,16	-0,22	0
OSMR	oncostatin M receptor	1,67	0,74	0,01	0,15	0	1,70	0,77	0,21	1,00	0	1,12	0,16	0
ZNF770	zinc finger protein 770	1,67	0,74	0,01	0,20	0	-2,98	-1,58	0,09	1,00	0	NA	NA	0
CEACAM6	carcinoembryonic antigen-related cell adhesion molecule 6 (non-specific cross reacting antigen)	1,67	0,74	0,49	0,81	0	4,22	2,08	0,10	1,00	0	NA	NA	0
CALM1	calmodulin 1 (phosphorylase kinase, delta)	1,66	0,73	0,02	0,26	0	1,21	0,27	0,32	1,00	0	-1,12	-0,16	0
EREG	epiregulin	1,66	0,73	0,03	0,30	0	-1,07	-0,10	0,59	1,00	0	1,43	0,52	0
FAM59A	family with sequence similarity 59, member A	1,66	0,73	0,00	0,14	0	1,37	0,45	0,06	1,00	0	NA	NA	0
UGCG	UDP-glucose ceramide glucosyltransferase	1,65	0,73	0,01	0,15	0	1,15	0,20	0,24	1,00	0	NA	NA	0
SMURF1	SMAD specific E3 ubiquitin protein ligase 1	1,65	0,73	0,05	0,36	0	1,11	0,16	0,31	1,00	0	NA	NA	0
EDC3	enhancer of mRNA decapping 3 homolog (S. cerevisiae)	1,65	0,72	0,01	0,20	0	1,17	0,23	0,38	1,00	0	NA	NA	0
ABCG1	ATP-binding cassette, sub-family G (WHITE), member 1	1,65	0,72	0,01	0,20	0	1,21	0,27	0,26	1,00	0	NA	NA	0
CFLAR	CASP8 and FADD-like apoptosis regulator	1,64	0,72	0,02	0,25	0	1,36	0,45	0,19	1,00	0	NA	NA	0
PCDH1	protocadherin 1	1,64	0,72	0,18	0,58	0	-1,09	-0,12	0,70	1,00	0	NA	NA	0

MYO1E	myosin IE	1,64	0,71	0,01	0,17	0	1,22	0,29	0,01	0,66	0	NA	NA	0
KATNB1	katanin p80 (WD repeat containing) subunit B 1	1,64	0,71	0,01	0,15	0	1,40	0,48	0,14	1,00	0	NA	NA	0
MAST4	microtubule associated serine/threonine kinase family member 4	1,64	0,71	0,03	0,31	0	-1,22	-0,29	0,39	1,00	0	NA	NA	0
ZNF710	zinc finger protein 710	1,63	0,71	0,01	0,16	0	1,30	0,38	0,16	1,00	0	NA	NA	0
CFLAR	CASP8 and FADD-like apoptosis regulator	1,63	0,71	0,01	0,18	0	1,21	0,28	0,24	1,00	0	NA	NA	0
KIF4A	kinesin family member 4A	1,63	0,70	0,01	0,22	0	-1,02	-0,03	0,90	1,00	0	NA	NA	0
NA	NA	1,62	0,70	0,15	0,54	0	-1,93	-0,95	0,03	0,98	0	1,06	0,08	0
CDK1	cyclin-dependent kinase 1	1,62	0,70	0,06	0,39	0	1,13	0,18	0,50	1,00	0	NA	NA	0
PDAP1	PDGFA associated protein 1	1,62	0,70	0,01	0,15	0	1,09	0,12	0,71	1,00	0	NA	NA	0
ENSA	endosulfine alpha	1,62	0,70	0,06	0,38	0	1,15	0,21	0,18	1,00	0	1,06	0,09	0
RGS5	regulator of G-protein signaling 5	1,62	0,70	0,31	0,70	0	-1,00	-0,01	NA	NA	0	-1,02	-0,02	0
PAFAH1B1	platelet-activating factor acetylhydrolase 1b, regulatory subunit 1 (45kDa)	1,62	0,69	0,01	0,20	0	1,03	0,04	0,95	1,00	0	-1,12	-0,16	0
MFSD6	major facilitator superfamily domain containing 6	1,62	0,69	0,00	0,14	0	-1,13	-0,17	0,70	1,00	0	-1,20	-0,27	0
INSIG1	insulin induced gene 1	1,61	0,69	0,00	0,13	0	1,33	0,41	0,28	1,00	0	NA	NA	0
PI4K2A	phosphatidylinositol 4-kinase type 2 alpha	1,61	0,69	0,01	0,15	0	-1,02	-0,03	0,80	1,00	0	1,01	0,02	0
SC5DL	sterol-C5-desaturase (ERG3 delta-5-desaturase homolog, S. cerevisiae)-like	1,60	0,68	0,02	0,23	0	-1,36	-0,45	0,22	1,00	0	NA	NA	0
MTF1	metal-regulatory transcription factor 1	1,60	0,68	0,04	0,34	0	1,15	0,20	0,24	1,00	0	NA	NA	0
CAMSAP1L1	calmodulin regulated spectrin-associated protein 1-like 1	1,60	0,68	0,01	0,22	0	-1,05	-0,07	0,66	1,00	0	-1,02	-0,03	0
PRC1	protein regulator of cytokinesis 1	1,60	0,68	0,05	0,37	0	1,04	0,06	0,62	1,00	0	NA	NA	0
EHD1	EH-domain containing 1	1,59	0,67	0,01	0,18	0	-1,01	-0,01	0,95	1,00	0	NA	NA	0
MRPS12	mitochondrial ribosomal protein S12	1,59	0,67	0,02	0,27	0	1,09	0,12	0,57	1,00	0	NA	NA	0
HSPA13	heat shock protein 70kDa family, member 13	1,59	0,67	0,02	0,28	0	-1,09	-0,12	0,63	1,00	0	-1,10	-0,14	0
BLM	Bloom syndrome, RecQ helicase-like	1,59	0,67	0,02	0,27	0	-1,10	-0,13	0,63	1,00	0	NA	NA	0
LRP8	low density lipoprotein receptor-related protein 8, apolipoprotein e receptor	1,59	0,67	0,01	0,21	0	-1,09	-0,13	0,63	1,00	0	NA	NA	0
TMOD3	tropomodulin 3 (ubiquitous)	1,59	0,67	0,01	0,15	0	-1,27	-0,35	0,48	1,00	0	-1,18	-0,23	0
LYN	v-yes-1 Yamaguchi sarcoma viral related oncogene homolog	1,59	0,67	0,06	0,40	0	1,53	0,62	0,00	0,34	0	1,23	0,30	0
SPC25	SPC25, NDC80 kinetochore complex component, homolog (S. cerevisiae)	1,59	0,67	0,01	0,21	0	-1,04	-0,06	0,84	1,00	0	NA	NA	0
NFKB2	nuclear factor of kappa light polypeptide gene enhancer in B-cells 2 (p49/p100)	1,58	0,66	0,01	0,20	0	-1,17	-0,22	0,70	1,00	0	1,13	0,18	0
CDA	cytidine deaminase	1,58	0,66	0,03	0,31	0	1,18	0,23	0,40	1,00	0	1,33	0,41	0
BAZ2A	bromodomain adjacent to zinc finger domain, 2A	1,58	0,66	0,03	0,30	0	1,04	0,05	0,72	1,00	0	1,03	0,05	0
CEACAM1	carcinoembryonic antigen-related cell adhesion molecule 1 (biliary glycoprotein)	1,58	0,66	0,55	0,84	0	-1,01	-0,01	0,97	1,00	0	NA	NA	0
PLCG2	phospholipase C, gamma 2 (phosphatidylinositol-specific)	1,58	0,66	0,02	0,26	0	-1,03	-0,04	0,90	1,00	0	NA	NA	0
NFKBIA	nuclear factor of kappa light polypeptide gene enhancer in B-cells inhibitor, alpha	1,58	0,66	0,01	0,20	0	1,30	0,38	0,12	1,00	0	NA	NA	0
AJAP1	adherens junctions associated protein 1	1,58	0,66	0,02	0,26	0	-1,12	-0,16	0,57	1,00	0	NA	NA	0
MAST4	microtubule associated serine/threonine kinase family member 4	1,57	0,65	0,07	0,41	0	-1,01	-0,01	0,97	1,00	0	NA	NA	0
SLC2A6	solute carrier family 2 (facilitated glucose transporter), member 6	1,57	0,65	0,06	0,40	0	1,03	0,05	0,76	1,00	0	NA	NA	0
LRP8	low density lipoprotein receptor-related protein 8, apolipoprotein e receptor	1,56	0,65	0,07	0,41	0	1,00	0,00	1,00	1,00	0	-1,08	-0,10	0
SLC5A3	solute carrier family 5 (sodium/myo-inositol cotransporter), member 3	1,56	0,65	0,14	0,54	0	-1,04	-0,05	NA	NA	0	-1,11	-0,14	0
AKR1C2	aldo-keto reductase family 1, member C2 (dihydrodiol dehydrogenase 2; bile acid binding protein; 3-alpha hydroxysteroid dehydrogenase, type III)	1,56	0,65	0,02	0,24	0	1,49	0,57	0,05	1,00	0	-1,05	-0,07	0
CFLAR	CASP8 and FADD-like apoptosis regulator	1,56	0,65	0,01	0,15	0	1,21	0,28	0,22	1,00	0	NA	NA	0
VRK1	vaccinia related kinase 1	1,56	0,64	0,02	0,27	0	1,06	0,08	0,45	1,00	0	NA	NA	0
CEBPG	CCAAT/enhancer binding protein (C/EBP), gamma	1,56	0,64	0,00	0,12	0	1,07	0,09	0,40	1,00	0	1,01	0,02	0
SERPINB3	serpin peptidase inhibitor, clade B (ovalbumin), member 3	1,56	0,64	0,02	0,23	0	1,57	0,65	0,02	0,94	0	NA	NA	0
PLCL2	phospholipase C-like 2	1,56	0,64	0,06	0,38	0	-1,00	0,00	NA	NA	0	1,13	0,18	0
G3BP1	GTPase activating protein (SH3 domain) binding protein 1	1,56	0,64	0,03	0,30	0	1,14	0,19	0,72	1,00	0	-1,54	-0,62	0
BIRC5	baculoviral IAP repeat-containing 5	1,56	0,64	0,04	0,32	0	1,06	0,09	0,58	1,00	0	NA	NA	0
RSC1A1	regulatory solute carrier protein, family 1, member 1	1,56	0,64	0,02	0,23	0	1,38	0,47	0,13	1,00	0	NA	NA	0
IL4R	interleukin 4 receptor	1,55	0,64	0,00	0,12	0	1,07	0,09	0,52	1,00	0	NA	NA	0
TMED2	transmembrane emp24 domain trafficking protein 2	1,55	0,64	0,02	0,27	0	1,06	0,09	0,88	1,00	0	-1,33	-0,42	0
PTPN2	protein tyrosine phosphatase, non-receptor type 2	1,55	0,64	0,02	0,23	0	1,20	0,27	0,28	1,00	0	NA	NA	0
RAP2A	RAP2A, member of RAS oncogene family	1,55	0,64	0,07	0,40	0	1,02	0,02	0,94	1,00	0	-1,61	-0,68	0
ZNF394	zinc finger protein 394	1,55	0,63	0,01	0,22	0	1,31	0,38	0,02	0,84	0	NA	NA	0
BIRC5	baculoviral IAP repeat-containing 5	1,55	0,63	0,14	0,53	0	1,21	0,27	0,52	1,00	0	NA	NA	0
CYP27B1	cytochrome P450, family 27, subfamily B, polypeptide 1	1,55	0,63	0,14	0,54	0	1,08	0,11	0,48	1,00	0	NA	NA	0
RNF19B	ring finger protein 19B	1,55	0,63	0,24	0,64	0	1,39	0,48	0,02	0,85	0	1,09	0,13	0
ZNF652	zinc finger protein 652	1,54	0,63	0,11	0,49	0	-1,36	-0,44	0,47	1,00	0	-1,23	-0,30	0
SLAMF7	SLAM family member 7	1,54	0,63	0,29	0,69	0	1,04	0,05	0,83	1,00	0	NA	NA	0
MN1	meningioma (disrupted in balanced translocation) 1	1,54	0,63	0,01	0,20	0	-1,13	-0,18	0,24	1,00	0	NA	NA	0
HIST2H2BE	histone cluster 2, H2be	1,54	0,63	0,00	0,14	0	1,43	0,51	0,10	1,00	0	NA	NA	0
TRIP13	thyroid hormone receptor interactor 13	1,54	0,62	0,02	0,26	0	1,01	0,02	0,90	1,00	0	NA	NA	0
LINS1	lines homolog 1 (Drosophila)	1,54	0,62	0,02	0,27	0	-1,00	0,00	1,00	1,00	0	NA	NA	0
TIPRL	TIP41, TOR signaling pathway regulator-like (S. cerevisiae)	1,54	0,62	0,26	0,66	0	-1,10	-0,13	0,60	1,00	0	-1,37	-0,45	0
CD47	CD47 molecule	1,54	0,62	0,08	0,42	0	-1,01	-0,01	0,97	1,00	0	-1,02	-0,03	0
DNAJB12	DnaJ (Hsp40) homolog, subfamily B, member 12	1,54	0,62	0,02	0,22	0	1,15	0,20	0,49	1,00	0	NA	NA	0
CDK1	cyclin-dependent kinase 1	1,54	0,62	0,07	0,42	0	1,24	0,31	0,35	1,00	0	NA	NA	0
ITSN2	intersectin 2	1,54	0,62	0,01	0,16	0	1,21	0,28	0,14	1,00	0	NA	NA	0
KCNK1	potassium channel, subfamily K, member 1	1,54	0,62	0,08	0,43	0	1,30	0,37	0,21	1,00	0	NA	NA	0
PSMD11	proteasome (prosome, macropain) 26S subunit, non-ATPase, 11	1,53	0,62	0,04	0,33	0	1,15	0,20	0,45	1,00	0	NA	NA	0
PVR	poliovirus receptor	1,53	0,62	0,03	0,29	0	1,03	0,04	0,84	1,00	0	NA	NA	0
DCBLD2	discoidin, CUB and LCCL domain containing 2	1,53	0,62	0,21	0,61	0	-3,01	-1,59	0,20	1,00	0	-1,85	-0,90	0
NCAPG	non-SMC condensin I complex, subunit G	1,53	0,61	0,04	0,32	0	-1,11	-0,15	0,35	1,00	0	NA	NA	0
SLC2A9	solute carrier family 2 (facilitated glucose transporter), member 9	1,53	0,61	0,05	0,35	0	1,01	0,02	0,94	1,00	0	NA	NA	0
MGRN1	mahogunin, ring finger 1	1,53	0,61	0,03	0,29	0	1,07	0,10	0,62	1,00	0	NA	NA	0
IL1R2	interleukin 1 receptor, type II	1,53	0,61	0,08	0,43	0	-1,44	-0,53	0,43	1,00	0	NA	NA	0
KRT33A	keratin 33A	1,53	0,61	0,07	0,42	0	1,53	0,62	0,05	1,00	0	1,32	0,40	0
SENP5	SUMO1/sentrin specific peptidase 5	1,53	0,61	0,03	0,30	0	1,11	0,15	0,26	1,00	0	NA	NA	0
NASP	nuclear autoantigenic sperm protein (histone-binding)	1,52	0,61	0,02	0,25	0	1,08	0,11	0,71	1,00	0	NA	NA	0
NRP2	neuropilin 2	1,52	0,61	0,01	0,18	0	1,00	0,00	NA	NA	0	NA	NA	0
MTF1	metal-regulatory transcription factor 1	1,52	0,61	0,00	0,12	0	1,09	0,13	0,62	1,00	0	1,10	0,14	0
MAP7D1	MAP7 domain containing 1	1,52	0,61	0,01	0,19	0	1,15	0,20	0,14	1,00	0	NA	NA	0
FEN1	flap structure-specific endonuclease 1	1,52	0,61	0,01	0,18	0	1,01	0,01	0,93	1,00	0	NA	NA	0
TOP1	topoisomerase (DNA) I	1,52	0,60	0,01	0,19	0	-1,14	-0,19	0,61	1,00	0	NA	NA	0
ABCC1	ATP-binding cassette, sub-family C (CFTR/MRP), member 1	1,52	0,60	0,01	0,20	0	-1,10	-0,14	0,46	1,00	0	NA	NA	0
UBE2H	ubiquitin-conjugating enzyme E2H (UBC8 homolog, yeast)	1,52	0,60	0,05	0,37	0	1,17	0,23	0,48	1,00	0	1,08	0,12	0
PLAUR	plasminogen activator, urokinase receptor	1,52	0,60	0,01	0,17	0	1,50	0,59	0,02	0,94	0	NA	NA	0
DHRS1	dehydrogenase/reductase (SDR family) member 1	1,52	0,60	0,01	0,15	0	1,04	0,05	0,83	1,00	0	NA	NA	0
SOCS3	suppressor of cytokine signaling 3	1,51	0,60	0,15	0,55	0	-1,01	-0,01	0,96	1,00	0	1,02	0,03	0
MANSC1	MANSC domain containing 1	1,51	0,60	0,02	0,25	0	-1,30	-0,37	0,11	1,00	0	NA	NA	0
CPM	carboxypeptidase M	1,51	0,60	0,01	0,21	0	1,39	0,47	0,38	1,00	0	NA	NA	0
MKI67	antigen identified by monoclonal antibody Ki-67	1,51	0,59	0,17	0,58	0	-1,07	-0,10	0,55	1,00	0	NA	NA	0
NA	NA	1,51	0,59	0,09	0,46	0	1,17	0,22	0,50	1,00	0	NA	NA	0
TNFAIP6	tumor necrosis factor, alpha-induced protein 6	1,51	0,59	0,26	0,66	0	-1,01	-0,01	0,99	1,00	0	NA	NA	0
MCM2	minichromosome maintenance complex component 2	1,51	0,59	0,01	0,16	0	1,03	0,04	0,73	1,00	0	NA	NA	0
SLC22A4	solute carrier family 22 (organic cation/ergothioneine transporter), member 4	1,51	0,59	0,15	0,54	0	-1,00	0,00	NA	NA	0	1,33	0,41	0
TOE1	target of EGR1, member 1 (nuclear)	1,50	0,59	0,00	0,12	0	1,19	0,26	0,15	1,00	0	NA	NA	0
TMCC1	transmembrane and coiled-coil domain family 1	1,50	0,59	0,01	0,20	0	1,44	0,52	0,25	1,00	0	NA	NA	0
GTPBP1	GTP binding protein 1	1,50	0,58	0,04	0,33	0	-1,05	-0,08	0,66	1,00	0	1,08	0,11	0
FUT2	fucosyltransferase 2 (secretor status included)	1,50	0,58	0,14	0,54	0	1,04	0,06	0,64	1,00	0	NA	NA	0
KAT2B	K(lysine) acetyltransferase 2B	1,50	0,58	0,01	0,22	0	1,61	0,69	0,08	1,00	0	NA	NA	0
KIF18B	kinesin family member 18B	1,50	0,58	0,08	0,44	0	1,08	0,12	0,57	1,00	0	NA	NA	0
TUBG1	tubulin, gamma 1	1,50	0,58	0,01	0,18	0	-1,06	-0,08	0,41	1,00	0	NA	NA	0
LPHN2	latrophilin 2	1,50	0,58	0,03	0,30	0	1,18	0,24	0,44	1,00	0	-1,15	-0,21	0
TLK1	tousled-like kinase 1	1,49	0,58	0,03	0,31	0	-1,08	-0,11	0,52	1,00	0	-1,52	-0,60	0
JHDM1D	jumonji C domain containing histone demethylase 1 homolog D (S. cerevisiae)	1,49	0,58	0,00	0,11	0	1,20	0,26	0,20	1,00	0	NA	NA	0
NDC80	NDC80 homolog, kinetochore complex component (S. cerevisiae)	1,49	0,58	0,08	0,42	0	1,00	0,00	0,99	1,00	0	NA	NA	0
MED13	mediator complex subunit 13	1,49	0,58	0,05	0,37	0	-1,09	-0,13	0,50	1,00	0	-1,64	-0,71	0
MFHAS1	malignant fibrous histiocytoma amplified sequence 1	1,49	0,58	0,08	0,43	0	1,13	0,18	0,63	1,00	0	NA	NA	0
KIAA0494	KIAA0494	1,49	0,58	0,03	0,29	0	-1,42	-0,50	0,45	1,00	0	-1,37	-0,45	0
TPX2	TPX2, microtubule-associated, homolog (Xenopus laevis)	1,49	0,58	0,05	0,37	0	-1,07	-0,10	0,48	1,00	0	NA	NA	0
CYR61	cysteine-rich, angiogenic inducer, 61	1,49	0,58	0,11	0,48	0	-1,02	-0,03	0,82	1,00	0	NA	NA	0
ASF1B	ASF1 anti-silencing function 1 homolog B (S. cerevisiae)	1,49	0,58	0,02	0,27	0	1,17	0,23	0,45	1,00	0	NA	NA	0
GLCE	glucuronic acid epimerase	1,49	0,57	0,01	0,18	0	1,89	0,92	0,00	0,33	0	-1,28	-0,36	0
DLGAP4	discs, large (Drosophila) homolog-associated protein 4	1,49	0,57	0,05	0,35	0	1,17	0,23	0,35	1,00	0	1,05	0,07	0
RNF19B	ring finger protein 19B	1,49	0,57	0,29	0,69	0	1,17	0,22	0,10	1,00	0	1,10	0,14	0
SHCBP1	SHC SH2-domain binding protein 1	1,48	0,57	0,17	0,58	0	-1,01	-0,02	0,93	1,00	0	NA	NA	0
HJURP	Holliday junction recognition protein	1,48	0,57	0,07	0,42	0	1,10	0,14	0,44	1,00	0	NA	NA	0
ASPM	asp (abnormal spindle) homolog, microcephaly associated (Drosophila)	1,48	0,57	0,09	0,46	0	-1,14	-0,20	0,21	1,00	0	NA	NA	0
PIM1	pim-1 oncogene	1,48	0,57	0,05	0,35	0	1,48	0,57	0,00	0,46	0	NA	NA	0
RNASEH1	ribonuclease H1	1,48	0,56	0,02	0,23	0	1,10	0,14	0,61	1,00	0	NA	NA	0
PVRL2	poliovirus receptor-related 2 (herpesvirus entry mediator B)	1,48	0,56	0,11	0,48	0	-1,07	-0,10	0,46	1,00	0	NA	NA	0
TCOF1	Treacher Collins-Franceschetti syndrome 1	1,48	0,56	0,03	0,31	0	-1,04	-0,05	0,79	1,00	0	NA	NA	0
SERPINB9	serpin peptidase inhibitor, clade B (ovalbumin), member 9	1,48	0,56	0,07	0,41	0	-1,00	-0,01	0,98	1,00	0	NA	NA	0
GOLIM4	golgi integral membrane protein 4	1,48	0,56	0,11	0,49	0	-1,79	-0,84	0,23	1,00	0	NA	NA	0
JUNB	jun B proto-oncogene	1,47	0,56	0,10	0,46	0	1,12	0,16	0,41	1,00	0	1,17	0,22	0
ARIH1	ariadne homolog, ubiquitin-conjugating enzyme E2 binding protein, 1 (Drosophila)	1,47	0,56	0,00	0,14	0	1,06	0,08	0,83	1,00	0	-1,23	-0,30	0
MAFF	v-maf musculoaponeurotic fibrosarcoma oncogene homolog F (avian)	1,47	0,56	0,02	0,23	0	1,26	0,34	0,07	1,00	0	NA	NA	0
PROCR	protein C receptor, endothelial	1,47	0,56	0,01	0,21	0	1,04	0,06	0,68	1,00	0	NA	NA	0
SH3GL1	SH3-domain GRB2-like 1	1,47	0,55	0,06	0,39	0	1,19	0,25	0,12	1,00	0	NA	NA	0
GINS2	GINS complex subunit 2 (Psf2 homolog)	1,47	0,55	0,02	0,26	0	-1,01	-0,01	0,95	1,00	0	NA	NA	0
WWC1	WW and C2 domain containing 1	1,47	0,55	0,13	0,52	0	1,05	0,07	0,76	1,00	0	NA	NA	0
SBNO2	strawberry notch homolog 2 (Drosophila)	1,47	0,55	0,03	0,31	0	1,40	0,48	0,16	1,00	0	1,24	0,31	0
CD274	CD274 molecule	1,47	0,55	0,25	0,65	0	NA	NA	NA	NA	0	NA	NA	0
TBC1D12	TBC1 domain family, member 12	1,47	0,55	0,18	0,59	0	1,03	0,04	0,86	1,00	0	-1,19	-0,24	0
TLR2	toll-like receptor 2	1,47	0,55	0,08	0,43	0	-1,03	-0,04	0,92	1,00	0	NA	NA	0
LIG3	ligase III, DNA, ATP-dependent	1,46	0,55	0,06	0,38	0	1,05	0,07	0,80	1,00	0	NA	NA	0
PBK	PDZ binding kinase	1,46	0,55	0,07	0,41	0	1,05	0,07	0,56	1,00	0	NA	NA	0
SRM	spermidine synthase	1,46	0,55	0,03	0,28	0	-1,03	-0,05	0,83	1,00	0	NA	NA	0

ZFR	zinc finger RNA binding protein	1,46	0,55	0,12	0,50	0	-1,27	-0,34	0,26	1,00	0	-1,23	-0,31	0
GABRE	gamma-aminobutyric acid (GABA) A receptor, epsilon	1,46	0,55	0,18	0,59	0	1,48	0,56	0,16	1,00	0	NA	NA	0
GMIP	GEM interacting protein	1,46	0,55	0,19	0,60	0	1,23	0,30	0,37	1,00	0	NA	NA	0
QPCT	glutaminyl-peptide cyclotransferase	1,46	0,55	0,03	0,30	0	1,44	0,53	0,02	0,88	0	1,05	0,08	0
FOSL1	FOS-like antigen 1	1,46	0,55	0,01	0,22	0	1,05	0,07	0,70	1,00	0	1,43	0,52	0
ZNF750	zinc finger protein 750	1,46	0,55	0,02	0,24	0	1,30	0,38	0,12	1,00	0	NA	NA	0
TNFAIP1	tumor necrosis factor, alpha-induced protein 1 (endothelial)	1,46	0,55	0,02	0,24	0	1,03	0,04	0,85	1,00	0	NA	NA	0
STYK1	serine/threonine/tyrosine kinase 1	1,46	0,55	0,10	0,48	0	-1,03	-0,05	0,90	1,00	0	NA	NA	0
ENC1	ectodermal-neural cortex 1 (with BTB-like domain)	1,46	0,55	0,11	0,49	0	1,11	0,15	0,58	1,00	0	2,17	1,12	0
NUDT4	nudix (nucleoside diphosphate linked moiety X)-type motif 4	1,46	0,54	0,01	0,20	0	-1,09	-0,13	0,69	1,00	0	NA	NA	0
H2AFV	H2A histone family, member V	1,46	0,54	0,08	0,44	0	-1,10	-0,14	0,40	1,00	0	-1,04	-0,05	0
RAB31	RAB31, member RAS oncogene family	1,46	0,54	0,06	0,40	0	1,17	0,23	0,25	1,00	0	-1,14	-0,18	0
CLN6	ceroid-lipofuscinosis, neuronal 6, late infantile, variant	1,46	0,54	0,02	0,23	0	NA	NA	NA	NA	0	NA	NA	0
TBRG4	transforming growth factor beta regulator 4	1,45	0,54	0,00	0,09	0	1,04	0,06	0,81	1,00	0	NA	NA	0
VPS37B	vacuolar protein sorting 37 homolog B (S. cerevisiae)	1,45	0,54	0,12	0,50	0	1,30	0,38	0,02	0,94	0	1,25	0,32	0
IL1R2	interleukin 1 receptor, type II	1,45	0,54	0,12	0,50	0	-1,55	-0,63	0,57	1,00	0	NA	NA	0
NA	NA	1,45	0,54	0,42	0,77	0	1,13	0,17	0,37	1,00	0	0,00	0,00	0
FLJ36031	hypothetical protein FLJ36031	1,45	0,53	0,05	0,35	0	NA	NA	NA	NA	0	NA	NA	0
GPX3	glutathione peroxidase 3 (plasma)	1,45	0,53	0,07	0,41	0	-1,08	-0,11	0,80	1,00	0	NA	NA	0
GCAT	glycine C-acetyltransferase	1,45	0,53	0,03	0,31	0	-1,16	-0,22	0,49	1,00	0	1,08	0,11	0
BICD2	bicaudal D homolog 2 (Drosophila)	1,45	0,53	0,00	0,14	0	1,18	0,23	0,04	1,00	0	-1,09	-0,12	0
PTPN11	protein tyrosine phosphatase, non-receptor type 11	1,45	0,53	0,01	0,20	0	-1,97	-0,98	0,34	1,00	0	-1,09	-0,12	0
CCDC85C	coiled-coil domain containing 85C	1,45	0,53	0,14	0,53	0	1,01	0,02	0,89	1,00	0	NA	NA	0
RFWD3	ring finger and WD repeat domain 3	1,45	0,53	0,08	0,42	0	-1,10	-0,14	0,47	1,00	0	NA	NA	0
10-set	septin 10	1,44	0,53	0,07	0,40	0	-1,05	-0,07	0,91	1,00	0	-1,23	-0,31	0
C20orf27	chromosome 20 open reading frame 27	1,44	0,53	0,01	0,19	0	-1,19	-0,25	0,65	1,00	0	NA	NA	0
LDLRAP1	low density lipoprotein receptor adaptor protein 1	1,44	0,53	0,04	0,35	0	-1,01	-0,02	0,92	1,00	0	NA	NA	0
GRAMD3	GRAM domain containing 3	1,44	0,53	0,03	0,31	0	-1,23	-0,30	0,01	0,57	0	NA	NA	0
DYSF	dysferlin, limb girdle muscular dystrophy 2B (autosomal recessive)	1,44	0,53	0,03	0,31	0	-1,00	0,00	NA	NA	0	NA	NA	0
DLGAP4	discs, large (Drosophila) homolog-associated protein 4	1,44	0,53	0,01	0,22	0	1,19	0,25	0,42	1,00	0	NA	NA	0
VSNL1	visinin-like 1	1,44	0,53	0,14	0,53	0	1,48	0,57	0,29	1,00	0	NA	NA	0
CENPN	centromere protein N	1,44	0,53	0,03	0,31	0	-1,13	-0,17	0,46	1,00	0	NA	NA	0
N4BP1	NEDD4 binding protein 1	1,44	0,53	0,00	0,11	0	1,15	0,20	0,17	1,00	0	-1,22	-0,28	0
EXO1	exonuclease 1	1,44	0,53	0,02	0,24	0	1,07	0,09	0,67	1,00	0	NA	NA	0
FEN1	flap structure-specific endonuclease 1	1,44	0,52	0,00	0,08	0	1,03	0,05	0,77	1,00	0	NA	NA	0
LMAN1	lectin, mannose-binding, 1	1,44	0,52	0,16	0,56	0	1,33	0,41	0,66	1,00	0	-1,18	-0,23	0
CLIP4	CAP-GLY domain containing linker protein family, member 4	1,44	0,52	0,01	0,20	0	1,12	0,17	0,33	1,00	0	NA	NA	0
ME1	malic enzyme 1, NADP(+)-dependent, cytosolic	1,44	0,52	0,07	0,42	0	1,15	0,20	0,15	1,00	0	-1,01	-0,02	0
MCM4	minichromosome maintenance complex component 4	1,44	0,52	0,01	0,18	0	1,02	0,02	0,92	1,00	0	NA	NA	0
VWA5A	von Willebrand factor A domain containing 5A	1,44	0,52	0,04	0,32	0	-1,48	-0,57	0,11	1,00	0	NA	NA	0
USP34	ubiquitin specific peptidase 34	1,44	0,52	0,04	0,33	0	1,00	0,01	0,99	1,00	0	1,11	0,16	0
KIF2C	kinesin family member 2C	1,44	0,52	0,11	0,49	0	1,14	0,19	0,28	1,00	0	NA	NA	0
PLAGL2	pleiomorphic adenoma gene-like 2	1,44	0,52	0,08	0,43	0	1,20	0,26	0,12	1,00	0	NA	NA	0
IPPK	inositol 1,3,4,5,6-pentakisphosphate 2-kinase	1,43	0,52	0,01	0,21	0	1,20	0,27	0,02	0,84	0	NA	NA	0
SORT1	sortilin 1	1,43	0,52	0,04	0,32	0	-1,22	-0,28	0,37	1,00	0	-1,02	-0,02	0
BCL2L1	BCL2-like 1	1,43	0,52	0,04	0,32	0	-1,08	-0,11	0,35	1,00	0	1,28	0,36	0
NTM	neurotrimin	1,43	0,52	0,04	0,33	0	-1,46	-0,55	0,16	1,00	0	NA	NA	0
RNASEH2A	ribonuclease H2, subunit A	1,43	0,52	0,01	0,19	0	-1,13	-0,17	0,34	1,00	0	NA	NA	0
SEMA3C	sema domain, immunoglobulin domain (Ig), short basic domain, secreted, (semaphorin) 3C	1,43	0,52	0,27	0,67	0	-1,04	-0,05	0,96	1,00	0	-1,14	-0,18	0
HOMER1	homer homolog 1 (Drosophila)	1,43	0,52	0,02	0,26	0	1,01	0,01	0,96	1,00	0	NA	NA	0
PLEKHA1	pleckstrin homology domain containing, family A (phosphoinositide binding specific) member 1	1,43	0,51	0,18	0,59	0	-1,11	-0,15	0,33	1,00	0	NA	NA	0
ADAMTSL4	ADAMTS-like 4	1,43	0,51	0,04	0,32	0	-1,01	-0,02	0,82	1,00	0	NA	NA	0
PTPN12	protein tyrosine phosphatase, non-receptor type 12	1,43	0,51	0,01	0,16	0	1,18	0,24	0,17	1,00	0	NA	NA	0
BUB1B	budding uninhibited by benzimidazoles 1 homolog beta (yeast)	1,43	0,51	0,13	0,52	0	1,01	0,02	0,87	1,00	0	NA	NA	0
TRIM16	tripartite motif-containing 16	1,43	0,51	0,01	0,16	0	1,15	0,20	0,36	1,00	0	-1,05	-0,07	0
PI4K2A	phosphatidylinositol 4-kinase type 2 alpha	1,43	0,51	0,10	0,47	0	1,03	0,04	0,75	1,00	0	1,15	0,20	0
LSM12	LSM12 homolog (S. cerevisiae)	1,43	0,51	0,07	0,42	0	1,09	0,13	0,41	1,00	0	NA	NA	0
TTK	TTK protein kinase	1,43	0,51	0,06	0,39	0	-1,01	-0,02	0,91	1,00	0	NA	NA	0
ETV4	ets variant 4	1,43	0,51	0,03	0,28	0	-1,37	-0,45	0,03	0,99	0	NA	NA	0
ATP10B	ATPase, class V, type 10B	1,42	0,51	0,08	0,44	0	1,06	0,08	0,84	1,00	0	NA	NA	0
SMURF1	SMAD specific E3 ubiquitin protein ligase 1	1,42	0,51	0,16	0,57	0	1,00	0,01	0,96	1,00	0	NA	NA	0
CCNF	cyclin F	1,42	0,51	0,01	0,21	0	1,29	0,37	0,14	1,00	0	NA	NA	0
CREB1	cAMP responsive element binding protein 1	1,42	0,50	0,09	0,45	0	1,12	0,16	0,21	1,00	0	NA	NA	0
ME1	malic enzyme 1, NADP(+)-dependent, cytosolic	1,42	0,50	0,14	0,54	0	1,05	0,07	0,64	1,00	0	0,00	0,00	0
NA	NA	1,41	0,50	0,02	0,26	0	1,17	0,23	0,14	1,00	0	NA	NA	0
RAB27A	RAB27A, member RAS oncogene family	1,41	0,50	0,03	0,31	0	1,07	0,10	0,52	1,00	0	NA	NA	0
CDCP1	CUB domain containing protein 1	1,41	0,50	0,07	0,42	0	1,14	0,19	0,16	1,00	0	1,38	0,47	0
PTPN11	protein tyrosine phosphatase, non-receptor type 11	1,41	0,50	0,03	0,30	0	-1,26	-0,34	0,23	1,00	0	1,53	0,61	0
CDC20	cell division cycle 20 homolog (S. cerevisiae)	1,41	0,50	0,16	0,57	0	1,01	0,02	0,90	1,00	0	NA	NA	0
EGLN3	egl nine homolog 3 (C. elegans)	1,41	0,50	0,13	0,53	0	1,01	0,02	0,96	1,00	0	NA	NA	0
DNAJA1	DnaJ (Hsp40) homolog, subfamily A, member 1	1,41	0,50	0,01	0,21	0	1,07	0,09	0,60	1,00	0	-1,09	-0,12	0
ATXN1	ataxin 1	1,41	0,50	0,18	0,59	0	1,03	0,05	0,88	1,00	0	1,13	0,18	0
BCL3	B-cell CLL/lymphoma 3	1,41	0,50	0,17	0,57	0	2,23	1,16	0,01	0,47	0	NA	NA	0
PAK6	p21 protein (Cdc42/Rac)-activated kinase 6	1,41	0,50	0,04	0,34	0	-1,13	-0,17	0,28	1,00	0	NA	NA	0
CDK2	cyclin-dependent kinase 2	1,41	0,50	0,04	0,32	0	-1,13	-0,17	0,50	1,00	0	NA	NA	0
MKI67	antigen identified by monoclonal antibody Ki-67	1,41	0,49	0,40	0,76	0	1,02	0,03	0,71	1,00	0	NA	NA	0
ARHGAP11A	Rho GTPase activating protein 11A	1,41	0,49	0,05	0,37	0	1,04	0,06	0,66	1,00	0	-1,32	-0,40	0
EIF1	eukaryotic translation initiation factor 1	1,41	0,49	0,18	0,59	0	1,30	0,38	0,08	1,00	0	NA	NA	0
GALNT6	UDP-N-acetyl-alpha-D-galactosamine:polypeptide N-acetylgalactosaminyltransferase 6 (GalNAc-T6)	1,41	0,49	0,12	0,51	0	1,05	0,07	0,61	1,00	0	NA	NA	0
SH2B3	SH2B adaptor protein 3	1,41	0,49	0,01	0,18	0	1,02	0,04	0,83	1,00	0	NA	NA	0
RIT1	Ras-like without CAAX 1	1,41	0,49	0,02	0,27	0	1,67	0,74	0,03	0,99	0	-1,06	-0,08	0
HLA-E	major histocompatibility complex, class I, E	1,41	0,49	0,01	0,17	0	1,12	0,17	0,35	1,00	0	-1,01	-0,01	0
BUB1	budding uninhibited by benzimidazoles 1 homolog (yeast)	1,40	0,49	0,03	0,28	0	-1,23	-0,30	0,69	1,00	0	-1,03	-0,05	0
RALB	v-ral simian leukemia viral oncogene homolog B (ras related; GTP binding protein)	1,40	0,49	0,13	0,52	0	1,18	0,24	0,61	1,00	0	NA	NA	0
APC	adenomatous polyposis coli	1,40	0,49	0,18	0,58	0	-1,30	-0,38	0,20	1,00	0	-1,11	-0,16	0
CNIH3	cornichon homolog 3 (Drosophila)	1,40	0,49	0,15	0,55	0	-1,01	-0,02	NA	NA	0	-1,16	-0,21	0
SPEN	spen homolog, transcriptional regulator (Drosophila)	1,40	0,49	0,16	0,56	0	1,53	0,61	0,09	1,00	0	1,53	0,62	0
MFAP3L	microfibrillar-associated protein 3-like	1,40	0,49	0,12	0,50	0	1,09	0,12	0,78	1,00	0	NA	NA	0
IPO4	importin 4	1,40	0,49	0,01	0,20	0	-1,09	-0,12	0,47	1,00	0	NA	NA	0
TPM4	tropomyosin 4	1,40	0,48	0,03	0,29	0	1,14	0,19	0,33	1,00	0	NA	NA	0
PDLIM5	PDZ and LIM domain 5	1,40	0,48	0,42	0,77	0	1,04	0,05	0,95	1,00	0	-1,02	-0,03	0
ADAM12	ADAM metallopeptidase domain 12	1,40	0,48	0,14	0,54	0	-1,01	-0,01	NA	NA	0	1,54	0,62	0
SRPK1	SRSF protein kinase 1	1,40	0,48	0,08	0,44	0	-1,08	-0,11	0,83	1,00	0	-1,19	-0,26	0
LGALS8	lectin, galactoside-binding, soluble, 8	1,40	0,48	0,04	0,32	0	1,35	0,44	0,28	1,00	0	-1,09	-0,12	0
PPP1R15A	protein phosphatase 1, regulatory (inhibitor) subunit 15A	1,40	0,48	0,21	0,61	0	1,65	0,72	0,11	1,00	0	NA	NA	0
AKR1C1	aldo-keto reductase family 1, member C1 (dihydrodiol dehydrogenase 1; 20-alpha (3-alpha)-hydroxysteroid dehydrogenase)	1,40	0,48	0,05	0,36	0	1,48	0,57	0,03	0,99	0	-1,02	-0,02	0
ELOVL4	elongation of very long chain fatty acids (FEN1/Elo2, SUR4/Elo3, yeast)-like 4	1,40	0,48	0,03	0,32	0	1,23	0,30	0,28	1,00	0	-1,39	-0,47	0
TMEM177	transmembrane protein 177	1,39	0,48	0,04	0,33	0	-1,08	-0,11	0,44	1,00	0	NA	NA	0
UBE2C	ubiquitin-conjugating enzyme E2C	1,39	0,48	0,04	0,32	0	1,06	0,09	0,53	1,00	0	NA	NA	0
EPS8L1	EPS8-like 1	1,39	0,48	0,09	0,45	0	1,35	0,43	0,26	1,00	0	NA	NA	0
ALG13	asparagine-linked glycosylation 13 homolog (S. cerevisiae)	1,39	0,48	0,20	0,60	0	1,61	0,69	0,05	1,00	0	-1,14	-0,18	0
RANBP2	RAN binding protein 2	1,39	0,48	0,08	0,42	0	1,16	0,21	0,76	1,00	0	-1,15	-0,20	0
BARD1	BRCA1 associated RING domain 1	1,39	0,48	0,24	0,64	0	-1,06	-0,08	0,82	1,00	0	NA	NA	0
STAT3	signal transducer and activator of transcription 3 (acute-phase response factor)	1,39	0,48	0,01	0,22	0	1,04	0,06	0,50	1,00	0	-1,35	-0,43	0
GPX3	glutathione peroxidase 3 (plasma)	1,39	0,47	0,07	0,42	0	-1,11	-0,15	0,70	1,00	0	NA	NA	0
MED8	mediator complex subunit 8	1,39	0,47	0,06	0,38	0	1,20	0,26	0,06	1,00	0	NA	NA	0
FAM131A	family with sequence similarity 131, member A	1,39	0,47	0,01	0,17	0	1,06	0,08	0,66	1,00	0	-1,09	-0,12	0
SKIL	SKI-like oncogene	1,39	0,47	0,25	0,65	0	1,02	0,03	0,86	1,00	0	1,41	0,49	0
SLC23A2	solute carrier family 23 (nucleobase transporters), member 2	1,39	0,47	0,01	0,19	0	1,17	0,22	0,11	1,00	0	-1,18	-0,24	0
SUV39H1	suppressor of variegation 3-9 homolog 1 (Drosophila)	1,39	0,47	0,19	0,59	0	1,12	0,16	0,47	1,00	0	NA	NA	0
G6PC3	glucose 6 phosphatase, catalytic, 3	1,38	0,47	0,19	0,59	0	-1,05	-0,07	0,80	1,00	0	1,29	0,37	0
NA	NA	1,38	0,47	0,01	0,19	0	1,03	0,04	0,82	1,00	0	NA	NA	0
CCL2	chemokine (C-C motif) ligand 2	1,38	0,47	0,04	0,32	0	-1,01	-0,01	0,96	1,00	0	1,22	0,29	0
STX11	syntaxin 11	1,38	0,47	0,19	0,59	0	NA	NA	NA	NA	0	NA	NA	0
RAB27B	RAB27B, member RAS oncogene family	1,38	0,47	0,19	0,60	0	-1,21	-0,28	0,47	1,00	0	1,04	0,05	0
MBNL1	muscleblind-like (Drosophila)	1,38	0,47	0,21	0,61	0	1,20	0,27	0,72	1,00	0	-1,15	-0,21	0
ZBTB43	zinc finger and BTB domain containing 43	1,38	0,46	0,03	0,30	0	1,39	0,48	0,08	1,00	0	NA	NA	0
KIAA0247	KIAA0247	1,38	0,46	0,06	0,39	0	1,15	0,20	0,10	1,00	0	NA	NA	0
PNPO	pyridoxamine 5'-phosphate oxidase	1,38	0,46	0,04	0,32	0	NA	NA	NA	NA	0	NA	NA	0
CDC6	cell division cycle 6 homolog (S. cerevisiae)	1,38	0,46	0,04	0,34	0	-1,09	-0,12	0,50	1,00	0	NA	NA	0
KCTD15	potassium channel tetramerisation domain containing 15	1,38	0,46	0,01	0,22	0	-1,25	-0,33	0,04	1,00	0	NA	NA	0
STARD5	StAR-related lipid transfer (START) domain containing 5	1,38	0,46	0,03	0,28	0	1,51	0,59	0,02	0,95	0	NA	NA	0
PAPOLA	poly(A) polymerase alpha	1,38	0,46	0,02	0,28	0	1,92	0,94	0,27	1,00	0	-1,10	-0,13	0
VIPR1	vasoactive intestinal peptide receptor 1	1,38	0,46	0,11	0,49	0	1,20	0,26	0,12	1,00	0	NA	NA	0
KIF2C	kinesin family member 2C	1,37	0,46	0,22	0,63	0	-1,01	-0,01	0,98	1,00	0	NA	NA	0
ZMYND8	zinc finger, MYND-type containing 8	1,37	0,46	0,11	0,50	0	1,21	0,27	0,18	1,00	0	-1,02	-0,02	0
PLXNA2	plexin A2	1,37	0,46	0,38	0,75	0	-1,01	-0,02	0,90	1,00	0	-1,04	-0,06	0
PALLD	palladin, cytoskeletal associated protein	1,37	0,46	0,09	0,45	0	-1,05	-0,07	0,84	1,00	0	-1,18	-0,23	0
ITCH	itchy E3 ubiquitin protein ligase homolog (mouse)	1,37	0,46	0,07	0,41	0	1,07	0,10	0,52	1,00	0	NA	NA	0
AQR	aquarius homolog (mouse)	1,37	0,45	0,05	0,37	0	-1,02	-0,02	0,90	1,00	0	NA	NA	0
DOCK9	dedicator of cytokinesis 9	1,37	0,45	0,02	0,27	0	-1,01	-0,01	0,95	1,00	0	-1,23	-0,30	0
SLC38A1	solute carrier family 38, member 1	1,37	0,45	0,03	0,31	0	-1,01	-0,01	0,91	1,00	0	1,02	0,03	0
G0S2	G0/G1switch 2	1,37	0,45	0,11	0,48	0	-1,43	-0,52	0,27	1,00	0	NA	NA	0
RRM1	ribonucleotide reductase M1	1,37	0,45	0,07	0,41	0	1,00	0,00	0,99	1,00	0	NA	NA	0
CREB1	cAMP responsive element binding protein 1	1,36	0,45	0,33	0,71	0	1,12	0,16	0,73	1,00	0	NA	NA	0
PDLIM7	PDZ and LIM domain 7 (enigma)	1,36	0,45	0,04	0,34	0	-1,01	-0,02	NA	NA	0	1,22	0,28	0
MCM3	minichromosome maintenance complex component 3	1,36	0,45	0,05	0,36	0	-1,21	-0,27	0,03	1,00	0	NA	NA	0

MICAL2	microtubule associated monoxygenase, calponin and LIM domain containing 2	1,36	0,45	0,03	0,30	0	1,05	0,08	0,71	1,00	0	1,11	0,15	0
EDNRA	endothelin receptor type A	1,36	0,45	0,18	0,59	0	-1,08	-0,11	0,84	1,00	0	NA	NA	0
SRA1	steroid receptor RNA activator 1	1,36	0,44	0,03	0,31	0	NA	NA	NA	NA	0	NA	NA	0
RAB27A	RAB27A, member RAS oncogene family	1,36	0,44	0,08	0,44	0	1,17	0,23	0,08	1,00	0	NA	NA	0
NA	NA	1,36	0,44	0,08	0,43	0	1,29	0,37	0,35	1,00	0	NA	NA	0
RNGTT	RNA guanylyltransferase and 5'-phosphatase	1,36	0,44	0,06	0,38	0	1,13	0,18	0,74	1,00	0	-1,03	-0,04	0
UBE4B	ubiquitination factor E4B (UFD2 homolog, yeast)	1,36	0,44	0,08	0,43	0	-1,99	-0,99	0,16	1,00	0	-1,08	-0,10	0
CCDC46	coiled-coil domain containing 46	1,36	0,44	0,37	0,74	0	-1,01	-0,02	NA	NA	0	-1,28	-0,36	0
CCL8	chemokine (C-C motif) ligand 8	1,36	0,44	0,26	0,66	0	1,02	0,02	0,91	1,00	0	1,03	0,04	0
PXDN	peroxidasin homolog (Drosophila)	1,36	0,44	0,15	0,54	0	1,18	0,23	0,17	1,00	0	1,06	0,08	0
ATXN7	ataxin 7	1,36	0,44	0,33	0,71	0	1,10	0,13	0,57	1,00	0	-1,09	-0,12	0
TRIM25	tripartite motif-containing 25	1,35	0,44	0,09	0,45	0	1,05	0,08	0,87	1,00	0	NA	NA	0
SFT2D2	SFT2 domain containing 2	1,35	0,44	0,16	0,56	0	1,15	0,21	0,29	1,00	0	1,04	0,06	0
TOP2A	topoisomerase (DNA) II alpha 170kDa	1,35	0,44	0,14	0,53	0	-1,03	-0,04	0,77	1,00	0	NA	NA	0
NA	NA	1,35	0,44	0,07	0,40	0	1,04	0,06	0,89	1,00	0	NA	NA	0
CYTH1	cytohesin 1	1,35	0,43	0,06	0,39	0	-1,03	-0,05	0,72	1,00	0	-1,32	-0,40	0
CEP72	centrosomal protein 72kDa	1,35	0,43	0,10	0,48	0	-1,10	-0,14	0,46	1,00	0	NA	NA	0
MALT1	mucosa associated lymphoid tissue lymphoma translocation gene 1	1,35	0,43	0,09	0,44	0	1,01	0,02	0,89	1,00	0	NA	NA	0
GPR109B	G protein-coupled receptor 109B	1,35	0,43	0,52	0,83	0	1,03	0,05	0,89	1,00	0	NA	NA	0
HIST3H2A	histone cluster 3, H2a	1,35	0,43	0,15	0,55	0	-1,13	-0,17	0,43	1,00	0	NA	NA	0
ST3GAL5	ST3 beta-galactoside alpha-2,3-sialyltransferase 5	1,35	0,43	0,09	0,46	0	1,01	0,01	0,98	1,00	0	-1,25	-0,32	0
AVPI1	arginine vasopressin-induced 1	1,35	0,43	0,09	0,45	0	-1,06	-0,08	0,73	1,00	0	NA	NA	0
NT5E	5'-nucleotidase, ecto (CD73)	1,35	0,43	0,02	0,25	0	1,01	0,02	0,92	1,00	0	NA	NA	0
DLGAP5	discs, large (Drosophila) homolog-associated protein 5	1,35	0,43	0,21	0,61	0	-1,02	-0,03	0,84	1,00	0	NA	NA	0
MALT1	mucosa associated lymphoid tissue lymphoma translocation gene 1	1,35	0,43	0,04	0,32	0	-1,08	-0,11	0,49	1,00	0	NA	NA	0
RSAD1	radical S-adenosyl methionine domain containing 1	1,35	0,43	0,01	0,15	0	1,10	0,14	0,41	1,00	0	NA	NA	0
BOK	BCL2-related ovarian killer	1,35	0,43	0,04	0,34	0	NA	NA	NA	NA	0	NA	NA	0
TAOK3	TAO kinase 3	1,35	0,43	0,21	0,62	0	-1,08	-0,11	0,31	1,00	0	NA	NA	0
EIF2C2	eukaryotic translation initiation factor 2C, 2	1,35	0,43	0,03	0,30	0	1,02	0,03	0,84	1,00	0	1,14	0,18	0
PID1	phosphotyrosine interaction domain containing 1	1,35	0,43	0,13	0,52	0	1,57	0,65	0,18	1,00	0	NA	NA	0
PPPDE2	PPPDE peptidase domain containing 2	1,35	0,43	0,03	0,28	0	1,14	0,19	0,09	1,00	0	1,12	0,17	0
DHCR7	7-dehydrocholesterol reductase	1,35	0,43	0,03	0,30	0	1,07	0,09	0,37	1,00	0	1,03	0,04	0
AKR1C2	aldo-keto reductase family 1, member C2 (dihydrodiol dehydrogenase 2; bile acid binding protein; 3-alpha hydroxysteroid dehydrogenase, type III)	1,35	0,43	0,08	0,42	0	1,42	0,50	0,08	1,00	0	-1,01	-0,01	0
LRRN3	leucine rich repeat neuronal 3	1,35	0,43	0,58	0,86	0	-1,01	-0,01	NA	NA	0	1,37	0,46	0
STX5	syntaxin 5	1,34	0,43	0,05	0,35	0	-1,08	-0,11	0,61	1,00	0	1,58	0,66	0
ADRM1	adhesion regulating molecule 1	1,34	0,43	0,04	0,34	0	1,05	0,07	0,50	1,00	0	NA	NA	0
BCL11A	B-cell CLL/lymphoma 11A (zinc finger protein)	1,34	0,43	0,05	0,37	0	1,25	0,32	0,21	1,00	0	NA	NA	0
TMCC1	transmembrane and coiled-coil domain family 1	1,34	0,43	0,22	0,63	0	1,09	0,12	0,54	1,00	0	-1,18	-0,24	0
EPS8L1	EPS8-like 1	1,34	0,43	0,08	0,44	0	1,27	0,35	0,30	1,00	0	NA	NA	0
COL8A1	collagen, type VIII, alpha 1	1,34	0,43	0,25	0,65	0	-1,47	-0,56	0,44	1,00	0	1,03	0,05	0
MED27	mediator complex subunit 27	1,34	0,42	0,03	0,30	0	-1,07	-0,10	0,60	1,00	0	NA	NA	0
MPHOSPH6	M-phase phosphoprotein 6	1,34	0,42	0,09	0,46	0	1,27	0,34	0,02	0,84	0	NA	NA	0
KLF7	Kruppel-like factor 7 (ubiquitous)	1,34	0,42	0,17	0,58	0	1,13	0,17	0,25	1,00	0	-2,78	-1,47	0
AURKA	aurora kinase A	1,34	0,42	0,10	0,48	0	1,03	0,04	0,72	1,00	0	NA	NA	0
CHAF1A	chromatin assembly factor 1, subunit A (p150)	1,34	0,42	0,08	0,43	0	-1,16	-0,22	0,20	1,00	0	NA	NA	0
ATAD2	ATPase family, AAA domain containing 2	1,34	0,42	0,10	0,47	0	1,02	0,02	0,94	1,00	0	-1,06	-0,08	0
MICAL2	microtubule associated monoxygenase, calponin and LIM domain containing 2	1,34	0,42	0,02	0,27	0	-1,01	-0,02	0,90	1,00	0	1,14	0,19	0
SYMPK	symplekin	1,34	0,42	0,06	0,39	0	-1,04	-0,05	NA	NA	0	NA	NA	0
NRBF2	nuclear receptor binding factor 2	1,34	0,42	0,12	0,51	0	1,16	0,22	0,05	1,00	0	NA	NA	0
CUX1	cut-like homeobox 1	1,34	0,42	0,17	0,57	0	1,02	0,03	0,85	1,00	0	-1,02	-0,02	0
TRIB2	tribbles homolog 2 (Drosophila)	1,34	0,42	0,13	0,53	0	-1,14	-0,18	0,41	1,00	0	-1,20	-0,27	0
AP1S2	adaptor-related protein complex 1, sigma 2 subunit	1,34	0,42	0,46	0,80	0	-1,35	-0,44	0,38	1,00	0	-1,52	-0,60	0
NA	NA	1,34	0,42	0,01	0,17	0	-1,02	-0,03	0,85	1,00	0	1,06	0,08	0
KIAA0040	KIAA0040	1,34	0,42	0,41	0,77	0	-1,44	-0,52	0,02	0,84	0	NA	NA	0
EPS8L1	EPS8-like 1	1,34	0,42	0,09	0,45	0	1,26	0,33	0,37	1,00	0	NA	NA	0
LARP4	La ribonucleoprotein domain family, member 4	1,34	0,42	0,09	0,45	0	1,03	0,04	0,77	1,00	0	-1,20	-0,27	0
TAB2	TGF-beta activated kinase 1/MAP3K7 binding protein 2	1,34	0,42	0,03	0,30	0	-1,09	-0,12	0,66	1,00	0	-1,09	-0,12	0
SLC7A8	solute carrier family 7 (amino acid transporter, L-type), member 8	1,34	0,42	0,37	0,74	0	-1,68	-0,75	0,21	1,00	0	NA	NA	0
METTL11A	methyltransferase like 11A	1,33	0,42	0,02	0,27	0	NA	NA	NA	NA	0	NA	NA	0
MDC1	mediator of DNA-damage checkpoint 1	1,33	0,42	0,17	0,58	0	-1,25	-0,33	0,06	1,00	0	NA	NA	0
GALNT1	UDP-N-acetyl-alpha-D-galactosamine:polypeptide N-acetylgalactosaminyltransferase 1 (GalNAc-T1)	1,33	0,42	0,07	0,41	0	1,03	0,05	0,71	1,00	0	-1,27	-0,34	0
ZFP36	zinc finger protein 36, C3H type, homolog (mouse)	1,33	0,42	0,12	0,51	0	1,17	0,23	0,29	1,00	0	-1,25	-0,33	0
SLC7A6	solute carrier family 7 (cationic amino acid transporter, y+ system), member 6	1,33	0,42	0,01	0,21	0	1,03	0,05	0,85	1,00	0	1,05	0,07	0
CRYAB	crystallin, alpha B	1,33	0,41	0,08	0,42	0	1,40	0,48	0,11	1,00	0	-1,04	-0,06	0
SLC43A3	solute carrier family 43, member 3	1,33	0,41	0,01	0,21	0	1,45	0,53	0,01	0,67	0	NA	NA	0
TEAD4	TEA domain family member 4	1,33	0,41	0,08	0,44	0	1,00	0,00	0,98	1,00	0	NA	NA	0
GLIPR1	GLI pathogenesis-related 1	1,33	0,41	0,20	0,60	0	1,05	0,07	0,82	1,00	0	NA	NA	0
SLC19A1	solute carrier family 19 (folate transporter), member 1	1,33	0,41	0,13	0,52	0	1,12	0,16	0,47	1,00	0	1,12	0,16	0
RCC1	regulator of chromosome condensation 1	1,33	0,41	0,04	0,35	0	1,03	0,04	0,88	1,00	0	NA	NA	0
FLVCR2	feline leukemia virus subgroup C cellular receptor family, member 2	1,33	0,41	0,19	0,60	0	-1,01	-0,01	0,97	1,00	0	NA	NA	0
EDEM1	ER degradation enhancer, mannosidase alpha-like 1	1,33	0,41	0,01	0,19	0	1,08	0,10	0,51	1,00	0	1,02	0,03	0
DSC2	desmocollin 2	1,33	0,41	0,16	0,57	0	1,36	0,44	0,28	1,00	0	NA	NA	0
MFAP3	microfibrillar-associated protein 3	1,33	0,41	0,06	0,39	0	-1,48	-0,56	0,02	0,94	0	-1,08	-0,10	0
SORL1	sortilin-related receptor, L(DLR class) A repeats-containing	1,33	0,41	0,14	0,53	0	-1,29	-0,37	0,05	1,00	0	NA	NA	0
KIAA0494	KIAA0494	1,33	0,41	0,04	0,34	0	1,11	0,15	0,72	1,00	0	-1,27	-0,35	0
DDX19A	DEAD (Asp-Glu-Ala-As) box polypeptide 19A	1,33	0,41	0,15	0,55	0	-1,08	-0,11	0,68	1,00	0	NA	NA	0
BCR	breakpoint cluster region	1,32	0,41	0,11	0,49	0	-1,01	-0,02	0,89	1,00	0	NA	NA	0
NAPA	N-ethylmaleimide-sensitive factor attachment protein, alpha	1,32	0,41	0,13	0,52	0	1,10	0,14	0,68	1,00	0	1,58	0,66	0
KIF18A	kinesin family member 18A	1,32	0,40	0,10	0,48	0	-1,05	-0,06	0,81	1,00	0	NA	NA	0
NA	NA	1,32	0,40	0,44	0,78	0	1,17	0,23	0,27	1,00	0	-1,41	-0,50	0
CSNK1A1	casein kinase 1, alpha 1	1,32	0,40	0,08	0,43	0	1,20	0,26	0,37	1,00	0	-1,35	-0,44	0
AIM1	absent in melanoma 1	1,32	0,40	0,01	0,19	0	-1,04	-0,06	0,55	1,00	0	-1,11	-0,16	0
KCTD14	potassium channel tetramerisation domain containing 14	1,32	0,40	0,57	0,85	0	-1,20	-0,26	0,42	1,00	0	1,04	0,06	0
ENTPD7	ectonucleoside triphosphate diphosphohydrolase 7	1,32	0,40	0,02	0,27	0	1,13	0,18	0,32	1,00	0	1,14	0,18	0
ZNF35	zinc finger protein 35	1,32	0,40	0,13	0,53	0	1,32	0,40	0,04	1,00	0	-1,11	-0,16	0
PRKAR2A	protein kinase, cAMP-dependent, regulatory, type II, alpha	1,32	0,40	0,04	0,33	0	1,09	0,12	0,53	1,00	0	1,05	0,08	0
KIF11	kinesin family member 11	1,32	0,40	0,11	0,49	0	-1,01	-0,02	0,89	1,00	0	NA	NA	0
STX17	syntaxin 17	1,32	0,40	0,12	0,51	0	-1,00	-0,01	0,98	1,00	0	NA	NA	0
FOSL2	FOS-like antigen 2	1,32	0,40	0,23	0,64	0	-1,01	-0,02	0,95	1,00	0	1,20	0,27	0
MTPAP	mitochondrial poly(A) polymerase	1,32	0,40	0,06	0,39	0	1,06	0,09	0,74	1,00	0	-1,19	-0,26	0
ST14	suppression of tumorigenicity 14 (colon carcinoma)	1,32	0,40	0,36	0,74	0	1,10	0,13	0,32	1,00	0	NA	NA	0
YY1	YY1 transcription factor	1,32	0,40	0,07	0,41	0	-1,04	-0,06	0,85	1,00	0	NA	NA	0
PLK4	polo-like kinase 4	1,32	0,40	0,11	0,49	0	1,02	0,02	0,87	1,00	0	NA	NA	0
RBP1	retinol binding protein 1, cellular	1,32	0,40	0,58	0,86	0	1,29	0,37	0,12	1,00	0	NA	NA	0
PSENEN	presenilin enhancer 2 homolog (C. elegans)	1,32	0,40	0,22	0,62	0	1,37	0,46	0,14	1,00	0	NA	NA	0
FUT8	fucosyltransferase 8 (alpha (1,6) fucosyltransferase)	1,32	0,40	0,33	0,71	0	1,73	0,79	0,05	1,00	0	NA	NA	0
PDLIM4	PDZ and LIM domain 4	1,32	0,40	0,21	0,61	0	1,78	0,83	0,00	0,45	0	NA	NA	0
LARP4B	La ribonucleoprotein domain family, member 4B	1,31	0,40	0,03	0,29	0	1,17	0,23	0,17	1,00	0	-1,09	-0,11	0
KPNA2	karyopherin alpha 2 (RAG cohort 1, importin alpha 1)	1,31	0,40	0,04	0,32	0	1,04	0,05	0,55	1,00	0	NA	NA	0
PPP1R12A	protein phosphatase 1, regulatory (inhibitor) subunit 12A	1,31	0,39	0,24	0,64	0	-1,03	-0,05	0,83	1,00	0	-1,56	-0,64	0
PLAUR	plasminogen activator, urokinase receptor	1,31	0,39	0,03	0,30	0	1,10	0,14	0,58	1,00	0	NA	NA	0
SPHK1	sphingosine kinase 1	1,31	0,39	0,06	0,39	0	1,31	0,39	0,19	1,00	0	NA	NA	0
C3orf75	chromosome 3 open reading frame 75	1,31	0,39	0,08	0,42	0	NA	NA	NA	NA	0	NA	NA	0
SPRR3	small proline-rich protein 3	1,31	0,39	0,17	0,57	0	2,60	1,38	0,00	0,29	0	NA	NA	0
ELF3	E74-like factor 3 (ets domain transcription factor, epithelial-specific)	1,31	0,39	0,69	0,90	0	1,11	0,16	0,71	1,00	0	NA	NA	0
PTHLH	parathyroid hormone-like hormone	1,31	0,39	0,65	0,89	0	1,15	0,20	0,39	1,00	0	NA	NA	0
STS	steroid sulfatase (microsomal), isozyme S	1,31	0,39	0,33	0,72	0	1,01	0,02	0,91	1,00	0	NA	NA	0
C20orf54	chromosome 20 open reading frame 54	1,31	0,39	0,04	0,34	0	NA	NA	NA	NA	0	NA	NA	0
TANK	TRAF family member-associated NFKB activator	1,31	0,39	0,11	0,49	0	1,04	0,06	0,85	1,00	0	NA	NA	0
DKC1	dyskeratosis congenita 1, dyskerin	1,31	0,39	0,13	0,52	0	1,14	0,19	0,58	1,00	0	NA	NA	0
TTC3	tetratricopeptide repeat domain 3	1,31	0,39	0,17	0,58	0	-1,40	-0,49	0,32	1,00	0	-1,28	-0,35	0
JMJD8	jumonji domain containing 8	1,31	0,39	0,03	0,30	0	NA	NA	NA	NA	0	NA	NA	0
GABPA	GA binding protein transcription factor, alpha subunit 60kDa	1,31	0,39	0,19	0,59	0	-1,16	-0,21	0,21	1,00	0	-1,37	-0,45	0
MAX	MYC associated factor X	1,31	0,39	0,14	0,54	0	1,33	0,41	0,48	1,00	0	-1,45	-0,54	0
HDAC9	histone deacetylase 9	1,31	0,39	0,27	0,67	0	1,42	0,50	0,09	1,00	0	NA	NA	0
STAP2	signal transducing adaptor family member 2	1,31	0,39	0,20	0,60	0	1,18	0,24	0,13	1,00	0	NA	NA	0
DLG1	discs, large homolog 1 (Drosophila)	1,31	0,39	0,07	0,41	0	1,14	0,19	0,72	1,00	0	-1,20	-0,27	0
CXXC1	CXXC finger 1 (PHD domain)	1,31	0,39	0,04	0,33	0	1,05	0,07	0,78	1,00	0	NA	NA	0
CPD	carboxypeptidase D	1,31	0,39	0,20	0,61	0	-1,19	-0,26	0,60	1,00	0	1,04	0,06	0
ARF3	ADP-ribosylation factor 3	1,31	0,39	0,11	0,48	0	-1,06	-0,09	0,76	1,00	0	2,00	1,00	0
PLEK2	pleckstrin 2	1,31	0,39	0,31	0,70	0	-1,12	-0,16	0,12	1,00	0	-1,35	-0,44	0
C21orf45	chromosome 21 open reading frame 45	1,31	0,39	0,20	0,60	0	1,11	0,15	0,21	1,00	0	NA	NA	0
MLX	MAX-like protein X	1,31	0,39	0,02	0,26	0	1,19	0,25	0,24	1,00	0	NA	NA	0
EDNRA	endothelin receptor type A	1,31	0,38	0,41	0,77	0	-1,01	-0,01	0,99	1,00	0	NA	NA	0
TNIP1	TNFAIP3 interacting protein 1	1,31	0,38	0,10	0,46	0	1,37	0,46	0,03	0,96	0	NA	NA	0
HOXC13	homeobox C13	1,30	0,38	0,10	0,46	0	-1,02	-0,03	0,89	1,00	0	NA	NA	0
SDF2L1	stromal cell-derived factor 2-like 1	1,30	0,38	0,13	0,52	0	-1,21	-0,27	0,15	1,00	0	NA	NA	0
CARS	cysteinyl-tRNA synthetase	1,30	0,38	0,09	0,45	0	-1,12	-0,17	0,26	1,00	0	NA	NA	0
SIRPA	signal-regulatory protein alpha	1,30	0,38	0,09	0,46	0	1,25	0,32	0,12	1,00	0	1,14	0,19	0
TFDP1	transcription factor Dp-1	1,30	0,38	0,01	0,22	0	1,10	0,13	0,76	1,00	0	-1,27	-0,35	0
ADAM10	ADAM metallopeptidase domain 10	1,30	0,38	0,21	0,61	0	1,16	0,21	0,82	1,00	0	-1,45	-0,53	0
DYNC1LI2	dynein, cytoplasmic 1, light intermediate chain 2	1,30	0,38	0,45	0,79	0	-1,01	-0,01	NA	NA	0	0,00	-0,01	0
NUSAP1	nucleolar and spindle associated protein 1	1,30	0,38	0,11	0,49	0	-1,02	-0,03	0,85	1,00	0	NA	NA	0
NR2C2	nuclear receptor subfamily 2, group C, member 2	1,30	0,38	0,13	0,52	0	1,15	0,21	0,16	1,00	0	NA	NA	0
AIM1L	absent in melanoma 1-like	1,30	0,38	0,50	0,82	0	1,39	0,48	0,19	1,00	0	NA	NA	0
RANGAP1	Ran GTPase activating protein 1	1,30	0,38	0,08	0,44	0	1,02	0,02	0,87	1,00	0	NA	NA	0
CALML5	calmodulin-like 5	1,30	0,38	0,55	0,85	0	-1,04	-0,05	0,86	1,00	0	NA	NA	0

ZFR	zinc finger RNA binding protein	1,30	0,38	0,09	0,45	0	-1,51	-0,59	0,00	0,33	0	-1,30	-0,38	0
SMC4	structural maintenance of chromosomes 4	1,30	0,38	0,11	0,48	0	1,04	0,06	0,82	1,00	0	NA	NA	0
ARNT2	aryl-hydrocarbon receptor nuclear translocator 2	1,30	0,38	0,50	0,82	0	-1,01	-0,01	NA	NA	0	-1,08	-0,11	0
DAAM1	dishevelled associated activator of morphogenesis 1	1,30	0,38	0,05	0,37	0	-1,14	-0,18	0,06	1,00	0	-1,11	-0,15	0
MFN1	mitofusin 1	1,30	0,38	0,09	0,46	0	1,37	0,45	0,43	1,00	0	-1,32	-0,39	0
ITCH	itchy E3 ubiquitin protein ligase homolog (mouse)	1,30	0,38	0,13	0,51	0	1,24	0,31	0,34	1,00	0	NA	NA	0
DOT1L	DOT1-like, histone H3 methyltransferase (S. cerevisiae)	1,30	0,38	0,02	0,27	0	NA	NA	NA	NA	0	NA	NA	0
PHGDH	phosphoglycerate dehydrogenase	1,30	0,38	0,02	0,22	0	-1,17	-0,23	0,06	1,00	0	1,07	0,09	0
LGMN	legumain	1,30	0,38	0,32	0,71	0	-1,00	0,00	0,96	1,00	0	-1,05	-0,07	0
ADARB1	adenosine deaminase, RNA-specific, B1	1,30	0,37	0,08	0,43	0	-1,48	-0,56	0,03	0,96	0	NA	NA	0
USP1	ubiquitin specific peptidase 1	1,30	0,37	0,19	0,59	0	-1,18	-0,24	0,25	1,00	0	NA	NA	0
MED28	mediator complex subunit 28	1,30	0,37	0,05	0,36	0	-1,02	-0,03	0,84	1,00	0	-1,16	-0,22	0
AURKA	aurora kinase A	1,30	0,37	0,08	0,43	0	1,08	0,12	0,43	1,00	0	NA	NA	0
NA	NA	1,30	0,37	0,03	0,31	0	1,02	0,03	0,76	1,00	0	1,10	0,13	0
LAMC2	laminin, gamma 2	1,30	0,37	0,71	0,91	0	1,01	0,01	0,97	1,00	0	NA	NA	0
VGLL4	vestigial like 4 (Drosophila)	1,30	0,37	0,20	0,61	0	-1,03	-0,04	0,87	1,00	0	-1,20	-0,27	0
KCTD14	potassium channel tetramerisation domain containing 14	1,30	0,37	0,40	0,76	0	-1,31	-0,39	0,16	1,00	0	-1,22	-0,28	0
TMSB15A	thymosin beta 15a	1,29	0,37	0,41	0,77	0	-1,01	-0,01	NA	NA	0	-1,30	-0,38	0
NCAPG2	non-SMC condensin II complex, subunit G2	1,29	0,37	0,03	0,30	0	1,10	0,14	0,37	1,00	0	NA	NA	0
GLS	glutaminase	1,29	0,37	0,19	0,60	0	1,27	0,34	0,14	1,00	0	-1,22	-0,29	0
GFPT2	glutamine-fructose-6-phosphate transaminase 2	1,29	0,37	0,22	0,63	0	-1,01	-0,01	NA	NA	0	-1,16	-0,21	0
CPNE3	copine III	1,29	0,37	0,27	0,67	0	-1,03	-0,05	0,95	1,00	0	-1,33	-0,41	0
SQRDL	sulfide quinone reductase-like (yeast)	1,29	0,37	0,02	0,25	0	1,34	0,42	0,01	0,71	0	1,18	0,24	0
HMBS	hydroxymethylbilane synthase	1,29	0,37	0,03	0,30	0	-1,02	-0,03	0,82	1,00	0	NA	NA	0
CENPF	centromere protein F, 350/400kDa (mitosin)	1,29	0,37	0,24	0,65	0	-1,16	-0,21	0,61	1,00	0	NA	NA	0
NA	NA	1,29	0,37	0,09	0,46	0	-1,14	-0,19	0,31	1,00	0	NA	NA	0
ARIH1	ariadne homolog, ubiquitin-conjugating enzyme E2 binding protein, 1 (Drosophila)	1,29	0,37	0,02	0,26	0	1,21	0,28	0,38	1,00	0	-1,32	-0,39	0
ATRN	attractin	1,29	0,37	0,21	0,62	0	-1,22	-0,29	0,19	1,00	0	-1,37	-0,46	0
RGS5	regulator of G-protein signaling 5	1,29	0,37	0,33	0,72	0	-1,01	-0,01	NA	NA	0	0,00	-0,01	0
GSPT1	G1 to S phase transition 1	1,29	0,37	0,16	0,56	0	1,03	0,05	0,88	1,00	0	-1,06	-0,10	0
NA	NA	1,29	0,37	0,33	0,72	0	NA	NA	NA	NA	0	NA	NA	0
PION	pigeon homolog (Drosophila)	1,29	0,37	0,26	0,66	0	1,04	0,06	0,76	1,00	0	-1,19	-0,25	0
MTG1	mitochondrial GTPase 1 homolog (S. cerevisiae)	1,29	0,37	0,25	0,65	0	-1,10	-0,14	0,17	1,00	0	NA	NA	0
ATP2B1	ATPase, Ca++ transporting, plasma membrane 1	1,29	0,37	0,27	0,67	0	1,06	0,08	0,87	1,00	0	NA	NA	0
TNFRSF25	tumor necrosis factor receptor superfamily, member 25	1,29	0,37	0,41	0,77	0	-1,02	-0,02	0,92	1,00	0	NA	NA	0
IFNGR1	interferon gamma receptor 1	1,29	0,36	0,05	0,35	0	1,44	0,53	0,08	1,00	0	-1,14	-0,19	0
AK1	adenylate kinase 1	1,29	0,36	0,11	0,50	0	1,05	0,08	0,55	1,00	0	1,03	0,05	0
BAK1	BCL2-antagonist/killer 1	1,29	0,36	0,27	0,67	0	-1,26	-0,33	0,02	0,84	0	1,16	0,22	0
IREB2	iron-responsive element binding protein 2	1,29	0,36	0,13	0,52	0	1,06	0,09	0,63	1,00	0	-1,27	-0,34	0
TFAP2A	transcription factor AP-2 alpha (activating enhancer binding protein 2 alpha)	1,29	0,36	0,18	0,58	0	1,13	0,17	0,81	1,00	0	2,17	1,12	0
CCNB2	cyclin B2	1,29	0,36	0,23	0,63	0	-1,07	-0,09	0,51	1,00	0	NA	NA	0
SPATS2L	spermatogenesis associated, serine-rich 2-like	1,29	0,36	0,30	0,69	0	-1,01	-0,02	NA	NA	0	-1,37	-0,45	0
HLA-E	major histocompatibility complex, class I, E	1,29	0,36	0,02	0,28	0	1,10	0,14	0,45	1,00	0	-1,01	-0,01	0
AKR1C1	aldo-keto reductase family 1, member C1 (dihydrodiol dehydrogenase 1; 20-alpha (3-alpha)-hydroxysteroid dehydrogenase)	1,29	0,36	0,16	0,57	0	1,61	0,69	0,04	1,00	0	1,07	0,10	0
DNAJA1	DnaJ (Hsp40) homolog, subfamily A, member 1	1,28	0,36	0,05	0,36	0	1,05	0,07	0,33	1,00	0	NA	NA	0
DDA1	DET1 and DDB1 associated 1	1,28	0,36	0,14	0,53	0	1,23	0,30	0,42	1,00	0	NA	NA	0
TP53BP2	tumor protein p53 binding protein, 2	1,28	0,36	0,14	0,54	0	1,28	0,36	0,03	1,00	0	NA	NA	0
KLF10	Kruppel-like factor 10	1,28	0,36	0,04	0,34	0	-1,01	-0,01	0,95	1,00	0	NA	NA	0
RRS1	RRS1 ribosome biogenesis regulator homolog (S. cerevisiae)	1,28	0,36	0,02	0,25	0	-1,08	-0,11	0,39	1,00	0	NA	NA	0
GINS3	GINS complex subunit 3 (Psf3 homolog)	1,28	0,36	0,02	0,27	0	1,39	0,48	0,01	0,74	0	NA	NA	0
FZD5	frizzled homolog 5 (Drosophila)	1,28	0,36	0,06	0,38	0	1,38	0,47	0,00	0,42	0	-1,01	-0,02	0
PRPS1	phosphoribosyl pyrophosphate synthetase 1	1,28	0,36	0,03	0,30	0	1,01	0,01	0,96	1,00	0	NA	NA	0
GPR107	G protein-coupled receptor 107	1,28	0,36	0,02	0,23	0	1,09	0,13	0,48	1,00	0	NA	NA	0
TMEM158	transmembrane protein 158 (gene/pseudogene)	1,28	0,36	0,11	0,48	0	1,36	0,44	0,36	1,00	0	1,40	0,49	0
MID2	midline 2	1,28	0,36	0,21	0,61	0	-1,32	-0,41	0,28	1,00	0	NA	NA	0
SHROOM2	shroom family member 2	1,28	0,36	0,16	0,57	0	1,16	0,21	0,48	1,00	0	NA	NA	0
NRIP1	nuclear receptor interacting protein 1	1,28	0,36	0,34	0,72	0	1,34	0,43	0,08	1,00	0	-1,23	-0,30	0
CLDN7	claudin 7	1,28	0,36	0,67	0,90	0	1,53	0,61	0,02	0,88	0	NA	NA	0
ST5	suppression of tumorigenicity 5	1,28	0,36	0,10	0,47	0	1,19	0,25	0,19	1,00	0	NA	NA	0
ABHD5	abhydrolase domain containing 5	1,28	0,36	0,22	0,62	0	1,26	0,34	0,25	1,00	0	NA	NA	0
CASK	calcium/calmodulin-dependent serine protein kinase (MAGUK family)	1,28	0,36	0,08	0,43	0	1,16	0,21	0,58	1,00	0	-1,61	-0,68	0
GRB2	growth factor receptor-bound protein 2	1,28	0,36	0,07	0,42	0	-1,10	-0,14	0,57	1,00	0	NA	NA	0
CPSF6	cleavage and polyadenylation specific factor 6, 68kDa	1,28	0,36	0,03	0,31	0	-1,11	-0,15	0,64	1,00	0	NA	NA	0
TSSC4	tumor suppressing subtransferable candidate 4	1,28	0,36	0,13	0,52	0	1,19	0,25	0,11	1,00	0	NA	NA	0
GLIPR1	GLI pathogenesis-related 1	1,28	0,36	0,17	0,58	0	1,24	0,32	0,34	1,00	0	NA	NA	0
ZNF24	zinc finger protein 24	1,28	0,36	0,16	0,56	0	-1,27	-0,35	0,30	1,00	0	-1,30	-0,38	0
CMAH	cytidine monophosphate-N-acetylneuraminic acid hydroxylase (CMP-N-acetylneuraminate monooxygenase) pseudogene	1,28	0,35	0,37	0,75	0	-1,01	-0,01	NA	NA	0	1,11	0,15	0
PTP4A1	protein tyrosine phosphatase type IVA, member 1	1,28	0,35	0,10	0,47	0	1,02	0,02	0,91	1,00	0	-1,15	-0,20	0
MOBKL1B	MOB1, Mps One Binder kinase activator-like 1B (yeast)	1,28	0,35	0,16	0,57	0	-1,20	-0,26	0,82	1,00	0	-1,92	-0,94	0
STYK1	serine/threonine/tyrosine kinase 1	1,28	0,35	0,07	0,41	0	-1,04	-0,05	0,89	1,00	0	NA	NA	0
HNRNPD	heterogeneous nuclear ribonucleoprotein D (AU-rich element RNA binding protein 1, 37kDa)	1,28	0,35	0,07	0,42	0	-1,01	-0,01	0,94	1,00	0	NA	NA	0
DNAJC1	DnaJ (Hsp40) homolog, subfamily C, member 1	1,28	0,35	0,11	0,49	0	1,09	0,12	0,36	1,00	0	NA	NA	0
KCNJ15	potassium inwardly-rectifying channel, subfamily J, member 15	1,28	0,35	0,38	0,75	0	-1,00	0,00	0,99	1,00	0	NA	NA	0
PMAIP1	phorbol-12-myristate-13-acetate-induced protein 1	1,28	0,35	0,33	0,71	0	1,20	0,26	0,18	1,00	0	1,05	0,08	0
PRKDC	protein kinase, DNA-activated, catalytic polypeptide	1,28	0,35	0,43	0,78	0	-1,17	-0,23	0,52	1,00	0	NA	NA	0
COL7A1	collagen, type VII, alpha 1	1,28	0,35	0,08	0,43	0	-1,13	-0,18	0,28	1,00	0	NA	NA	0
TRIB1	tribbles homolog 1 (Drosophila)	1,28	0,35	0,06	0,39	0	1,08	0,11	0,41	1,00	0	NA	NA	0
DTYMK	deoxythymidylate kinase (thymidylate kinase)	1,27	0,35	0,09	0,45	0	-1,05	-0,06	0,52	1,00	0	NA	NA	0
FAM64A	family with sequence similarity 64, member A	1,27	0,35	0,26	0,66	0	1,03	0,04	0,89	1,00	0	NA	NA	0
TCF7L2	transcription factor 7-like 2 (T-cell specific, HMG-box)	1,27	0,35	0,48	0,81	0	-1,31	-0,38	0,11	1,00	0	NA	NA	0
DHX30	DEAH (Asp-Glu-Ala-His) box polypeptide 30	1,27	0,35	0,21	0,62	0	1,01	0,01	0,95	1,00	0	NA	NA	0
SIK3	SIK family kinase 3	1,27	0,35	0,11	0,49	0	1,21	0,28	0,29	1,00	0	1,01	0,02	0
TEAD4	TEA domain family member 4	1,27	0,35	0,20	0,60	0	-1,15	-0,20	0,33	1,00	0	1,09	0,12	0
DARS2	aspartyl-tRNA synthetase 2, mitochondrial	1,27	0,35	0,17	0,57	0	-1,22	-0,29	0,27	1,00	0	NA	NA	0
MCM4	minichromosome maintenance complex component 4	1,27	0,35	0,15	0,55	0	1,08	0,11	0,51	1,00	0	NA	NA	0
U2AF2	U2 small nuclear RNA auxiliary factor 2	1,27	0,35	0,09	0,44	0	-1,03	-0,05	0,87	1,00	0	1,03	0,04	0
FANCI	Fanconi anemia, complementation group I	1,27	0,35	0,06	0,39	0	1,03	0,05	0,76	1,00	0	NA	NA	0
FLNB	filamin B, beta	1,27	0,35	0,39	0,76	0	-1,15	-0,20	0,08	1,00	0	NA	NA	0
E2F8	E2F transcription factor 8	1,27	0,35	0,06	0,39	0	1,19	0,26	0,10	1,00	0	-1,05	-0,07	0
CUL3	cullin 3	1,27	0,35	0,10	0,47	0	1,03	0,04	0,79	1,00	0	-1,23	-0,30	0
PANX1	pannexin 1	1,27	0,35	0,04	0,33	0	1,19	0,25	0,11	1,00	0	-1,11	-0,15	0
CHAF1B	chromatin assembly factor 1, subunit B (p60)	1,27	0,34	0,19	0,60	0	-1,08	-0,11	0,44	1,00	0	NA	NA	0
TPR	translocated promoter region (to activated MET oncogene)	1,27	0,34	0,16	0,57	0	-1,94	-0,96	0,01	0,50	0	-1,10	-0,14	0
CCNB1	cyclin B1	1,27	0,34	0,26	0,66	0	1,06	0,09	0,46	1,00	0	NA	NA	0
LARS2	leucyl-tRNA synthetase 2, mitochondrial	1,27	0,34	0,07	0,41	0	1,07	0,10	0,56	1,00	0	-1,33	-0,42	0
MGC2752	hypothetical LOC65996	1,27	0,34	0,06	0,40	0	1,03	0,04	0,88	1,00	0	-1,27	-0,34	0
UBXN7	UBX domain protein 7	1,27	0,34	0,20	0,61	0	-1,16	-0,21	0,23	1,00	0	-1,06	-0,09	0
RAB31	RAB31, member RAS oncogene family	1,27	0,34	0,22	0,62	0	1,16	0,21	0,18	1,00	0	-1,01	-0,02	0
PPP1R15A	protein phosphatase 1, regulatory (inhibitor) subunit 15A	1,27	0,34	0,34	0,72	0	1,58	0,66	0,18	1,00	0	NA	NA	0
PAFAH1B1	platelet-activating factor acetylhydrolase 1b, regulatory subunit 1 (45kDa)	1,27	0,34	0,02	0,28	0	1,03	0,04	0,73	1,00	0	1,06	0,09	0
CDK1	cyclin-dependent kinase 1	1,27	0,34	0,14	0,54	0	-1,02	-0,03	0,73	1,00	0	NA	NA	0
USP3	ubiquitin specific peptidase 3	1,27	0,34	0,04	0,35	0	-1,02	-0,02	0,86	1,00	0	NA	NA	0
SEC24A	SEC24 family, member A (S. cerevisiae)	1,27	0,34	0,17	0,57	0	1,14	0,18	0,44	1,00	0	NA	NA	0
DEFB1	defensin, beta 1	1,27	0,34	0,19	0,59	0	1,36	0,45	0,17	1,00	0	NA	NA	0
PDLIM4	PDZ and LIM domain 4	1,27	0,34	0,13	0,53	0	-1,05	-0,07	0,68	1,00	0	1,35	0,43	0
SIK3	SIK family kinase 3	1,27	0,34	0,13	0,53	0	1,05	0,07	0,51	1,00	0	NA	NA	0
SH3BP2	SH3-domain binding protein 2	1,27	0,34	0,04	0,34	0	1,11	0,15	0,29	1,00	0	NA	NA	0
NUCKS1	nuclear casein kinase and cyclin-dependent kinase substrate 1	1,27	0,34	0,30	0,69	0	1,14	0,19	0,41	1,00	0	NA	NA	0
KCTD5	potassium channel tetramerisation domain containing 5	1,26	0,34	0,14	0,53	0	1,25	0,32	0,05	1,00	0	NA	NA	0
FOSL2	FOS-like antigen 2	1,26	0,34	0,12	0,51	0	1,31	0,38	0,09	1,00	0	1,23	0,30	0
DNAJB9	DnaJ (Hsp40) homolog, subfamily B, member 9	1,26	0,34	0,35	0,73	0	1,07	0,10	0,67	1,00	0	1,15	0,21	0
ABCC1	ATP-binding cassette, sub-family C (CFTR/MRP), member 1	1,26	0,34	0,02	0,27	0	1,01	0,02	0,89	1,00	0	NA	NA	0
TOP1	topoisomerase (DNA) I	1,26	0,34	0,09	0,44	0	1,08	0,12	0,42	1,00	0	NA	NA	0
FOLR1	folate receptor 1 (adult)	1,26	0,34	0,37	0,74	0	1,15	0,21	0,26	1,00	0	1,05	0,06	0
GNE	glucosamine (UDP-N-acetyl)-2-epimerase/N-acetylmannosamine kinase	1,26	0,34	0,11	0,49	0	1,14	0,19	0,37	1,00	0	-1,05	-0,08	0
C17orf39	chromosome 17 open reading frame 39	1,26	0,34	0,12	0,51	0	-1,09	-0,13	0,60	1,00	0	NA	NA	0
PCNXL2	pecanex-like 2 (Drosophila)	1,26	0,34	0,30	0,69	0	-1,20	-0,26	0,09	1,00	0	NA	NA	0
SLC7A6	solute carrier family 7 (cationic amino acid transporter, y+ system), member 6	1,26	0,34	0,07	0,41	0	1,11	0,15	0,31	1,00	0	1,01	0,02	0
TIMELESS	timeless homolog (Drosophila)	1,26	0,34	0,09	0,44	0	-1,12	-0,17	0,25	1,00	0	NA	NA	0
ITPKC	inositol 1,4,5-trisphosphate 3-kinase C	1,26	0,34	0,28	0,68	0	-1,01	-0,02	0,93	1,00	0	NA	NA	0
NBN	nibrin	1,26	0,34	0,04	0,33	0	-1,03	-0,05	0,89	1,00	0	-1,49	-0,58	0
PAFAH1B1	platelet-activating factor acetylhydrolase 1b, regulatory subunit 1 (45kDa)	1,26	0,34	0,08	0,44	0	1,08	0,11	0,63	1,00	0	NA	NA	0
GPD2	glycerol-3-phosphate dehydrogenase 2 (mitochondrial)	1,26	0,34	0,05	0,35	0	-1,26	-0,33	0,46	1,00	0	-1,67	-0,73	0
KCNS3	potassium voltage-gated channel, delayed-rectifier, subfamily S, member 3	1,26	0,34	0,07	0,41	0	1,08	0,11	0,67	1,00	0	-1,02	-0,02	0
BCL7A	B-cell CLL/lymphoma 7A	1,26	0,34	0,25	0,66	0	-1,09	-0,13	0,44	1,00	0	NA	NA	0
WARS	tryptophanyl-tRNA synthetase	1,26	0,34	0,49	0,81	0	-1,18	-0,24	0,15	1,00	0	-1,09	-0,13	0
GLA	galactosidase, alpha	1,26	0,33	0,15	0,55	0	1,01	0,02	0,92	1,00	0	1,01	0,02	0
EPS8L1	EPS8-like 1	1,26	0,33	0,11	0,49	0	1,25	0,32	0,30	1,00	0	NA	NA	0
PDPN	podoplanin	1,26	0,33	0,07	0,42	0	-1,08	-0,11	0,57	1,00	0	NA	NA	0
GIT2	G protein-coupled receptor kinase interacting ArfGAP 2	1,26	0,33	0,12	0,51	0	1,06	0,08	0,71	1,00	0	NA	NA	0
SLC26A2	solute carrier family 26 (sulfate transporter), member 2	1,26	0,33	0,57	0,86	0	-1,19	-0,25	0,57	1,00	0	NA	NA	0
EGFR	epidermal growth factor receptor	1,26	0,33	0,20	0,60	0	-1,23	-0,30	0,14	1,00	0	NA	NA	0
GCDH	glutaryl-CoA dehydrogenase	1,26	0,33	0,16	0,56	0	-1,19	-0,25	0,02	0,89	0	NA	NA	0
SMEK1	SMEK homolog 1, suppressor of mek1 (Dictyostelium)	1,26	0,33	0,11	0,49	0	1,17	0,23	0,66	1,00	0	-1,15	-0,20	0
MMACHC	methylmalonic aciduria (cobalamin deficiency) cblC type, with homocystinuria	1,26	0,33	0,10	0,47	0	1,04	0,06	0,80	1,00	0	NA	NA	0
KCNAB1	potassium voltage-gated channel, shaker-related subfamily, beta member 1	1,26	0,33	0,19	0,60	0	-1,27	-0,34	0,35	1,00	0	1,03	0,05	0
ZWILCH	Zwilch, kinetochore associated, homolog (Drosophila)	1,26	0,33	0,18	0,58	0	-1,11	-0,14	0,59	1,00	0	NA	NA	0

TAGLN	transgelin	1,26	0,33	0,56	0,85	0	-1,04	-0,06	0,87	1,00	0	NA	NA	0
NA	NA	1,25	0,33	0,14	0,54	0	-1,09	-0,12	0,45	1,00	0	-1,11	-0,16	0
FBXL5	F-box and leucine-rich repeat protein 5	1,25	0,33	0,07	0,41	0	1,17	0,23	0,50	1,00	0	NA	NA	0
GSTM3	glutathione S-transferase mu 3 (brain)	1,25	0,33	0,26	0,66	0	-1,02	-0,03	0,89	1,00	0	-1,03	-0,05	0
KIAA1199	KIAA1199	1,25	0,33	0,06	0,40	0	1,60	0,67	0,19	1,00	0	-1,15	-0,20	0
HMMR	hyaluronan-mediated motility receptor (RHAMM)	1,25	0,33	0,30	0,70	0	-1,08	-0,11	0,58	1,00	0	NA	NA	0
MCM7	minichromosome maintenance complex component 7	1,25	0,33	0,10	0,48	0	-1,13	-0,18	0,31	1,00	0	NA	NA	0
RPS6KB2	ribosomal protein S6 kinase, 70kDa, polypeptide 2	1,25	0,32	0,13	0,53	0	1,17	0,23	0,29	1,00	0	1,40	0,49	0
SLC1A4	solute carrier family 1 (glutamate/neutral amino acid transporter), member 4	1,25	0,32	0,26	0,67	0	1,05	0,07	0,77	1,00	0	0,00	0,00	0
FHOD3	formin homology 2 domain containing 3	1,25	0,32	0,14	0,53	0	-1,01	-0,01	NA	NA	0	-1,59	-0,66	0
DHRS7B	dehydrogenase/reductase (SDR family) member 7B	1,25	0,32	0,18	0,59	0	-1,11	-0,15	0,33	1,00	0	NA	NA	0
KIAA0913	KIAA0913	1,25	0,32	0,19	0,59	0	1,11	0,15	0,49	1,00	0	NA	NA	0
EIF2AK1	eukaryotic translation initiation factor 2-alpha kinase 1	1,25	0,32	0,32	0,71	0	1,00	0,00	NA	NA	0	-1,14	-0,19	0
AKAP1	A kinase (PRKA) anchor protein 1	1,25	0,32	0,25	0,65	0	-1,02	-0,04	0,79	1,00	0	NA	NA	0
EIF2B3	eukaryotic translation initiation factor 2B, subunit 3 gamma, 58kDa	1,25	0,32	0,09	0,45	0	1,06	0,09	0,58	1,00	0	NA	NA	0
CHD1	chromodomain helicase DNA binding protein 1	1,25	0,32	0,09	0,45	0	1,05	0,07	0,55	1,00	0	-1,14	-0,18	0
GTPBP2	GTP binding protein 2	1,25	0,32	0,04	0,34	0	1,06	0,09	0,57	1,00	0	NA	NA	0
NRP1	neuropilin 1	1,25	0,32	0,10	0,47	0	-1,07	-0,10	0,70	1,00	0	NA	NA	0
B9D1	B9 protein domain 1	1,25	0,32	0,17	0,57	0	-1,19	-0,25	0,40	1,00	0	0,00	-0,01	0
SULT1E1	sulfotransferase family 1E, estrogen-preferring, member 1	1,25	0,32	0,29	0,68	0	-1,92	-0,94	0,03	1,00	0	NA	NA	0
AMIGO2	adhesion molecule with Ig-like domain 2	1,25	0,32	0,15	0,55	0	-1,13	-0,17	0,23	1,00	0	-1,09	-0,12	0
HSPA13	heat shock protein 70kDa family, member 13	1,25	0,32	0,21	0,61	0	1,17	0,23	0,17	1,00	0	-1,14	-0,18	0
BAT2L2	HLA-B associated transcript 2-like 2	1,25	0,32	0,29	0,69	0	-1,29	-0,36	0,09	1,00	0	-1,16	-0,21	0
RRAD	Ras-related associated with diabetes	1,25	0,32	0,27	0,67	0	1,59	0,67	0,20	1,00	0	NA	NA	0
C18orf22	chromosome 18 open reading frame 22	1,25	0,32	0,19	0,60	0	-1,06	-0,09	0,59	1,00	0	NA	NA	0
CLIP1	CAP-GLY domain containing linker protein 1	1,25	0,32	0,24	0,64	0	1,35	0,43	0,36	1,00	0	-1,27	-0,35	0
SIGMAR1	sigma non-opioid intracellular receptor 1	1,25	0,32	0,15	0,55	0	-1,23	-0,30	0,21	1,00	0	NA	NA	0
ZMYND8	zinc finger, MYND-type containing 8	1,25	0,32	0,17	0,57	0	1,01	0,01	0,96	1,00	0	NA	NA	0
RBBP8	retinoblastoma binding protein 8	1,25	0,32	0,15	0,55	0	1,07	0,10	0,34	1,00	0	NA	NA	0
06-mar	membrane-associated ring finger (C3HC4) 6	1,25	0,32	0,26	0,66	0	-1,25	-0,33	0,29	1,00	0	NA	NA	0
DOCK4	dedicator of cytokinesis 4	1,25	0,32	0,25	0,65	0	-1,18	-0,24	0,32	1,00	0	NA	NA	0
BRD4	bromodomain containing 4	1,25	0,32	0,15	0,55	0	-1,02	-0,02	0,91	1,00	0	-1,05	-0,08	0
CENPE	centromere protein E, 312kDa	1,25	0,32	0,52	0,83	0	1,15	0,20	0,35	1,00	0	NA	NA	0
CANT1	calcium activated nucleotidase 1	1,25	0,32	0,18	0,59	0	-1,06	-0,09	0,52	1,00	0	NA	NA	0
RECQL4	RecQ protein-like 4	1,25	0,32	0,11	0,48	0	-1,06	-0,08	0,64	1,00	0	NA	NA	0
HSPA9	heat shock 70kDa protein 9 (mortalin)	1,25	0,32	0,04	0,34	0	1,04	0,05	0,77	1,00	0	NA	NA	0
RNF41	ring finger protein 41	1,25	0,32	0,07	0,41	0	-1,03	-0,04	0,90	1,00	0	1,06	0,08	0
ORC6L	origin recognition complex, subunit 6 like (yeast)	1,24	0,32	0,10	0,48	0	-1,04	-0,06	0,79	1,00	0	NA	NA	0
TINF2	TERF1 (TRF1)-interacting nuclear factor 2	1,24	0,32	0,11	0,48	0	1,00	0,00	0,99	1,00	0	NA	NA	0
WHSC1	Wolf-Hirschhorn syndrome candidate 1	1,24	0,31	0,09	0,45	0	-1,01	-0,02	0,90	1,00	0	NA	NA	0
CEP55	centrosomal protein 55kDa	1,24	0,31	0,31	0,70	0	-1,02	-0,03	0,82	1,00	0	NA	NA	0
WRAP53	WD repeat containing, antisense to TP53	1,24	0,31	0,04	0,33	0	1,14	0,19	0,23	1,00	0	NA	NA	0
CALM1	calmodulin 1 (phosphorylase kinase, delta)	1,24	0,31	0,06	0,40	0	1,51	0,59	0,00	0,26	0	NA	NA	0
CYR61	cysteine-rich, angiogenic inducer, 61	1,24	0,31	0,16	0,56	0	1,12	0,16	0,34	1,00	0	NA	NA	0
KRT31	keratin 31	1,24	0,31	0,41	0,77	0	1,38	0,46	0,03	1,00	0	NA	NA	0
SUGP2	SURP and G patch domain containing 2	1,24	0,31	0,14	0,53	0	-1,05	-0,07	0,79	1,00	0	NA	NA	0
CYB5R1	cytochrome b5 reductase 1	1,24	0,31	0,12	0,51	0	1,13	0,17	0,23	1,00	0	NA	NA	0
FKBP14	FK506 binding protein 14, 22 kDa	1,24	0,31	0,18	0,58	0	-1,06	-0,08	0,70	1,00	0	-1,20	-0,28	0
PPRC1	peroxisome proliferator-activated receptor gamma, coactivator-related 1	1,24	0,31	0,07	0,42	0	1,03	0,05	0,76	1,00	0	NA	NA	0
VSNL1	visinin-like 1	1,24	0,31	0,27	0,67	0	1,50	0,58	0,06	1,00	0	NA	NA	0
DKC1	dyskeratosis congenita 1, dyskerin	1,24	0,31	0,11	0,49	0	-1,02	-0,03	0,86	1,00	0	NA	NA	0
BNIP3L	BCL2/adenovirus E1B 19kDa interacting protein 3-like	1,24	0,31	0,11	0,48	0	1,33	0,42	0,23	1,00	0	NA	NA	0
RMND5A	required for meiotic nuclear division 5 homolog A (S. cerevisiae)	1,24	0,31	0,22	0,63	0	-1,01	-0,02	0,93	1,00	0	NA	NA	0
TRAF3	TNF receptor-associated factor 3	1,24	0,31	0,11	0,50	0	1,03	0,05	0,69	1,00	0	-1,10	-0,14	0
CALCOCO1	calcium binding and coiled-coil domain 1	1,24	0,31	0,09	0,44	0	-1,59	-0,67	0,06	1,00	0	1,47	0,56	0
KIAA0284	KIAA0284	1,24	0,31	0,12	0,51	0	-1,01	-0,02	0,89	1,00	0	NA	NA	0
DHFR	dihydrofolate reductase	1,24	0,31	0,20	0,61	0	-1,04	-0,06	0,77	1,00	0	NA	NA	0
RAD51	RAD51 homolog (RecA homolog, E. coli) (S. cerevisiae)	1,24	0,31	0,20	0,60	0	-1,03	-0,04	0,76	1,00	0	NA	NA	0
C11orf2	chromosome 11 open reading frame 2	1,24	0,31	0,11	0,48	0	-1,01	-0,01	0,92	1,00	0	NA	NA	0
OXSR1	oxidative-stress responsive 1	1,24	0,31	0,05	0,35	0	1,30	0,38	0,01	0,65	0	NA	NA	0
ARHGEF4	Rho guanine nucleotide exchange factor (GEF) 4	1,24	0,31	0,13	0,52	0	-1,06	-0,09	0,74	1,00	0	NA	NA	0
DENND3	DENN/MADD domain containing 3	1,24	0,31	0,29	0,69	0	1,07	0,10	0,75	1,00	0	NA	NA	0
KIAA0355	KIAA0355	1,24	0,30	0,06	0,38	0	-1,06	-0,08	0,71	1,00	0	-1,35	-0,43	0
NSFL1C	NSFL1 (p97) cofactor (p47)	1,24	0,30	0,20	0,61	0	1,32	0,40	0,14	1,00	0	NA	NA	0
SEMA4D	sema domain, immunoglobulin domain (Ig), transmembrane domain (TM) and short cytoplasmic domain, (semaphorin) 4D	1,24	0,30	0,16	0,57	0	1,23	0,30	0,24	1,00	0	NA	NA	0
KIAA1539	KIAA1539	1,23	0,30	0,30	0,69	0	1,19	0,26	0,13	1,00	0	NA	NA	0
C1orf135	chromosome 1 open reading frame 135	1,23	0,30	0,32	0,71	0	1,05	0,07	0,79	1,00	0	NA	NA	0
TNFAIP6	tumor necrosis factor, alpha-induced protein 6	1,23	0,30	0,47	0,80	0	-1,02	-0,03	0,98	1,00	0	1,26	0,33	0
ARID1A	AT rich interactive domain 1A (SWI-like)	1,23	0,30	0,34	0,72	0	-1,06	-0,09	0,61	1,00	0	1,99	0,99	0
SERPINB2	serpin peptidase inhibitor, clade B (ovalbumin), member 2	1,23	0,30	0,20	0,61	0	1,12	0,16	0,33	1,00	0	NA	NA	0
GNAQ	guanine nucleotide binding protein (G protein), q polypeptide	1,23	0,30	0,15	0,55	0	-1,02	-0,03	0,81	1,00	0	NA	NA	0
TRAK1	trafficking protein, kinesin binding 1	1,23	0,30	0,08	0,43	0	-1,00	0,00	0,99	1,00	0	0,00	-0,01	0
DDX28	DEAD (Asp-Glu-Ala-Asp) box polypeptide 28	1,23	0,30	0,17	0,58	0	-1,04	-0,06	0,67	1,00	0	NA	NA	0
FAM20B	family with sequence similarity 20, member B	1,23	0,30	0,15	0,55	0	-1,09	-0,12	0,54	1,00	0	NA	NA	0
GOSR2	golgi SNAP receptor complex member 2	1,23	0,30	0,21	0,62	0	-1,03	-0,04	0,84	1,00	0	NA	NA	0
GPSM2	G-protein signaling modulator 2	1,23	0,30	0,21	0,62	0	1,07	0,10	0,63	1,00	0	NA	NA	0
PML	promyelocytic leukemia	1,23	0,30	0,56	0,85	0	-1,02	-0,02	0,89	1,00	0	1,13	0,18	0
NETO2	neuropilin (NRP) and tolloid (TLL)-like 2	1,23	0,30	0,09	0,46	0	1,06	0,08	0,47	1,00	0	-1,14	-0,18	0
SEL1L	sel-1 suppressor of lin-12-like (C. elegans)	1,23	0,30	0,23	0,64	0	-1,36	-0,44	0,06	1,00	0	-1,18	-0,24	0
POLD2	polymerase (DNA directed), delta 2, regulatory subunit 50kDa	1,23	0,30	0,09	0,45	0	-1,16	-0,22	0,26	1,00	0	NA	NA	0
IQGAP1	IQ motif containing GTPase activating protein 1	1,23	0,30	0,46	0,80	0	-3,16	-1,66	0,23	1,00	0	-1,67	-0,74	0
CXCR7	chemokine (C-X-C motif) receptor 7	1,23	0,30	0,04	0,34	0	1,34	0,42	0,07	1,00	0	NA	NA	0
MYO6	myosin VI	1,23	0,30	0,33	0,71	0	1,27	0,34	0,35	1,00	0	-1,18	-0,24	0
SAT1	spermidine/spermine N1-acetyltransferase 1	1,23	0,30	0,10	0,48	0	1,39	0,48	0,00	0,06	0	1,15	0,20	0
DPY19L1	dpy-19-like 1 (C. elegans)	1,23	0,30	0,15	0,54	0	-1,08	-0,11	0,38	1,00	0	-1,14	-0,18	0
LARS	leucyl-tRNA synthetase	1,23	0,30	0,05	0,37	0	-1,08	-0,11	0,66	1,00	0	NA	NA	0
RAD23A	RAD23 homolog A (S. cerevisiae)	1,23	0,30	0,13	0,52	0	1,05	0,06	0,91	1,00	0	NA	NA	0
PLK4	polo-like kinase 4	1,23	0,30	0,19	0,59	0	1,09	0,13	0,68	1,00	0	-1,27	-0,34	0
MYO1B	myosin IB	1,23	0,30	0,18	0,59	0	1,20	0,27	0,02	0,94	0	NA	NA	0
PION	pigeon homolog (Drosophila)	1,23	0,30	0,35	0,73	0	1,10	0,14	0,57	1,00	0	-1,20	-0,27	0
AFF4	AF4/FMR2 family, member 4	1,23	0,30	0,39	0,76	0	1,68	0,75	0,03	0,96	0	NA	NA	0
DICER1	dicer 1, ribonuclease type III	1,23	0,30	0,26	0,67	0	-1,09	-0,12	0,56	1,00	0	NA	NA	0
APOOL	apolipoprotein O-like	1,23	0,30	0,21	0,61	0	1,12	0,17	0,49	1,00	0	-1,10	-0,14	0
SARS	seryl-tRNA synthetase	1,23	0,30	0,07	0,40	0	-1,05	-0,07	0,40	1,00	0	NA	NA	0
BRD3	bromodomain containing 3	1,23	0,30	0,14	0,53	0	-1,20	-0,26	0,30	1,00	0	1,11	0,14	0
IL13RA1	interleukin 13 receptor, alpha 1	1,23	0,30	0,08	0,43	0	-1,06	-0,08	0,76	1,00	0	NA	NA	0
SIGMAR1	sigma non-opioid intracellular receptor 1	1,23	0,30	0,05	0,36	0	-1,01	-0,02	0,83	1,00	0	NA	NA	0
WNT5A	wingless-type MMTV integration site family, member 5A	1,23	0,29	0,19	0,60	0	-1,07	-0,09	0,77	1,00	0	-1,03	-0,05	0
ARFGEF1	ADP-ribosylation factor guanine nucleotide-exchange factor 1(brefeldin A-inhibited)	1,23	0,29	0,27	0,67	0	1,01	0,02	0,97	1,00	0	-1,18	-0,24	0
ID1	inhibitor of DNA binding 1, dominant negative helix-loop-helix protein	1,23	0,29	0,67	0,90	0	1,64	0,72	0,08	1,00	0	NA	NA	0
FAM175B	family with sequence similarity 175, member B	1,23	0,29	0,24	0,65	0	1,17	0,22	0,66	1,00	0	-1,52	-0,61	0
CPD	carboxypeptidase D	1,22	0,29	0,35	0,73	0	-1,05	-0,08	0,69	1,00	0	1,03	0,04	0
TUSC3	tumor suppressor candidate 3	1,22	0,29	0,31	0,70	0	-1,36	-0,44	0,03	0,97	0	NA	NA	0
GPR125	G protein-coupled receptor 125	1,22	0,29	0,25	0,65	0	1,12	0,16	0,09	1,00	0	1,13	0,17	0
RHBDF2	rhomboid 5 homolog 2 (Drosophila)	1,22	0,29	0,39	0,76	0	1,05	0,07	0,59	1,00	0	1,07	0,10	0
NAPA	N-ethylmaleimide-sensitive factor attachment protein, alpha	1,22	0,29	0,23	0,64	0	1,03	0,05	0,89	1,00	0	1,93	0,95	0
CD320	CD320 molecule	1,22	0,29	0,17	0,58	0	1,15	0,20	0,53	1,00	0	NA	NA	0
EMR2	egf-like module containing, mucin-like, hormone receptor-like 2	1,22	0,29	0,80	0,94	0	1,50	0,59	0,08	1,00	0	NA	NA	0
DENND1A	DENN/MADD domain containing 1A	1,22	0,29	0,19	0,59	0	1,18	0,24	0,41	1,00	0	-1,11	-0,15	0
COL4A1	collagen, type IV, alpha 1	1,22	0,29	0,46	0,80	0	-1,06	-0,09	0,80	1,00	0	-1,06	-0,09	0
BLNK	B-cell linker	1,22	0,29	0,13	0,52	0	-1,07	-0,10	0,74	1,00	0	NA	NA	0
RAD9A	RAD9 homolog A (S. pombe)	1,22	0,29	0,31	0,70	0	1,10	0,14	0,65	1,00	0	1,17	0,23	0
ARSJ	arylsulfatase family, member J	1,22	0,29	0,20	0,61	0	1,09	0,12	0,52	1,00	0	NA	NA	0
NA	NA	1,22	0,29	0,69	0,90	0	-1,00	-0,01	0,92	1,00	0	0,00	0,00	0
DDX11	DEAD/H (Asp-Glu-Ala-Asp/His) box polypeptide 11	1,22	0,29	0,06	0,38	0	-1,16	-0,22	0,26	1,00	0	NA	NA	0
CREB1	cAMP responsive element binding protein 1	1,22	0,29	0,19	0,59	0	1,28	0,36	0,05	1,00	0	NA	NA	0
PIP5K1A	phosphatidylinositol-4-phosphate 5-kinase, type I, alpha	1,22	0,28	0,07	0,42	0	-1,05	-0,07	0,86	1,00	0	-1,28	-0,36	0
TPCN1	two pore segment channel 1	1,22	0,28	0,30	0,69	0	1,10	0,14	0,30	1,00	0	1,39	0,47	0
LMNB1	lamin B1	1,22	0,28	0,27	0,67	0	-1,07	-0,10	0,74	1,00	0	-1,39	-0,48	0
ATXN7	ataxin 7	1,22	0,28	0,16	0,56	0	-1,08	-0,11	0,36	1,00	0	-1,22	-0,29	0
HMMR	hyaluronan-mediated motility receptor (RHAMM)	1,22	0,28	0,45	0,79	0	-1,04	-0,05	0,77	1,00	0	NA	NA	0
FAM38A	family with sequence similarity 38, member A	1,22	0,28	0,26	0,66	0	1,10	0,14	0,31	1,00	0	NA	NA	0
SEC23A	Sec23 homolog A (S. cerevisiae)	1,22	0,28	0,30	0,69	0	-1,22	-0,29	0,57	1,00	0	-1,23	-0,31	0
RFC3	replication factor C (activator 1) 3, 38kDa	1,22	0,28	0,29	0,68	0	1,06	0,08	0,52	1,00	0	NA	NA	0
C21orf63	chromosome 21 open reading frame 63	1,22	0,28	0,18	0,59	0	NA	NA	NA	NA	0	NA	NA	0
NOLC1	nucleolar and coiled-body phosphoprotein 1	1,22	0,28	0,06	0,38	0	1,03	0,05	0,64	1,00	0	NA	NA	0
MEIS3P1	Meis homeobox 3 pseudogene 1	1,22	0,28	0,42	0,78	0	-1,36	-0,44	0,12	1,00	0	-1,22	-0,29	0
PALLD	palladin, cytoskeletal associated protein	1,21	0,28	0,12	0,50	0	1,18	0,24	0,18	1,00	0	-1,09	-0,12	0
DAG1	dystroglycan 1 (dystrophin-associated glycoprotein 1)	1,21	0,28	0,15	0,55	0	1,17	0,23	0,54	1,00	0	-1,15	-0,21	0
MOAP1	modulator of apoptosis 1	1,21	0,28	0,09	0,45	0	1,03	0,05	0,66	1,00	0	-1,05	-0,08	0
PKP4	plakophilin 4	1,21	0,28	0,20	0,61	0	-1,17	-0,22	0,64	1,00	0	-1,19	-0,26	0
UNKL	unkempt homolog (Drosophila)-like	1,21	0,28	0,08	0,42	0	-1,09	-0,12	0,48	1,00	0	1,04	0,06	0
WDR45	WD repeat domain 45	1,21	0,28	0,15	0,55	0	-1,12	-0,16	0,14	1,00	0	NA	NA	0
CHIC2	cysteine-rich hydrophobic domain 2	1,21	0,28	0,53	0,83	0	1,03	0,05	0,77	1,00	0	NA	NA	0
SLC24A6	solute carrier family 24 (sodium/potassium/calcium exchanger), member 6	1,21	0,28	0,18	0,59	0	-1,09	-0,12	0,62	1,00	0	NA	NA	0
CAP2	CAP, adenylate cyclase-associated protein, 2 (yeast)	1,21	0,28	0,28	0,68	0	1,22	0,29	0,13	1,00	0	-1,11	-0,15	0
JAG1	jagged 1	1,21	0,28	0,55	0,84	0	1,22	0,29	0,56	1,00	0	-1,05	-0,07	0

CAD	carbamoyl-phosphate synthetase 2, aspartate transcarbamylase, and dihydroorotase	1,21	0,28	0,18	0,59	0	-1,22	-0,29	0,02	0,88	0	NA	NA	0
CDCA3	cell division cycle associated 3	1,21	0,28	0,13	0,52	0	1,00	0,01	0,98	1,00	0	NA	NA	0
C1orf106	chromosome 1 open reading frame 106	1,21	0,28	0,31	0,70	0	1,07	0,10	0,41	1,00	0	NA	NA	0
POLD4	polymerase (DNA-directed), delta 4	1,21	0,28	0,44	0,78	0	-1,15	-0,20	0,10	1,00	0	NA	NA	0
SLC29A2	solute carrier family 29 (nucleoside transporters), member 2	1,21	0,28	0,19	0,60	0	1,31	0,39	0,18	1,00	0	NA	NA	0
NA	NA	1,21	0,28	0,13	0,52	0	1,01	0,02	0,95	1,00	0	NA	NA	0
KLF6	Kruppel-like factor 6	1,21	0,28	0,55	0,84	0	1,10	0,13	0,77	1,00	0	-1,47	-0,55	0
DRAP1	DR1-associated protein 1 (negative cofactor 2 alpha)	1,21	0,28	0,12	0,50	0	1,12	0,16	0,45	1,00	0	NA	NA	0
DHRS7	dehydrogenase/reductase (SDR family) member 7	1,21	0,28	0,22	0,62	0	-1,06	-0,08	0,58	1,00	0	-1,12	-0,17	0
NOTCH2	notch 2	1,21	0,28	0,15	0,55	0	-1,45	-0,54	0,36	1,00	0	-1,25	-0,33	0
MAP4K5	mitogen-activated protein kinase kinase kinase kinase 5	1,21	0,28	0,12	0,50	0	1,06	0,08	0,78	1,00	0	-1,20	-0,28	0
RMND5A	required for meiotic nuclear division 5 homolog A (S. cerevisiae)	1,21	0,28	0,15	0,55	0	-1,07	-0,09	0,65	1,00	0	1,02	0,04	0
URB1	URB1 ribosome biogenesis 1 homolog (S. cerevisiae)	1,21	0,28	0,09	0,45	0	1,04	0,06	0,86	1,00	0	NA	NA	0
NCAPG	non-SMC condensin I complex, subunit G	1,21	0,27	0,36	0,73	0	-1,02	-0,02	0,89	1,00	0	NA	NA	0
TRAF6	TNF receptor-associated factor 6	1,21	0,27	0,27	0,67	0	1,17	0,23	0,12	1,00	0	NA	NA	0
SCLY	selenocysteine lyase	1,21	0,27	0,06	0,38	0	-1,08	-0,10	0,58	1,00	0	NA	NA	0
ZNF91	zinc finger protein 91	1,21	0,27	0,29	0,69	0	1,08	0,11	0,71	1,00	0	-1,37	-0,45	0
RPL28	ribosomal protein L28	1,21	0,27	0,25	0,65	0	-1,10	-0,14	0,58	1,00	0	1,02	0,03	0
SLC33A1	solute carrier family 33 (acetyl-CoA transporter), member 1	1,21	0,27	0,29	0,69	0	-1,12	-0,16	0,49	1,00	0	-1,12	-0,16	0
ARHGEF5	Rho guanine nucleotide exchange factor (GEF) 5	1,21	0,27	0,32	0,71	0	-1,09	-0,13	0,38	1,00	0	NA	NA	0
CADM1	cell adhesion molecule 1	1,21	0,27	0,35	0,73	0	-1,10	-0,14	0,46	1,00	0	NA	NA	0
UBN1	ubinuclein 1	1,21	0,27	0,17	0,57	0	-1,08	-0,11	0,73	1,00	0	-1,25	-0,33	0
LARP1	La ribonucleoprotein domain family, member 1	1,21	0,27	0,16	0,56	0	1,09	0,12	0,55	1,00	0	1,74	0,80	0
SENP3	SUMO1/sentrin/SMT3 specific peptidase 3	1,21	0,27	0,21	0,62	0	1,08	0,11	0,71	1,00	0	NA	NA	0
SPRED2	sprouty-related, EVH1 domain containing 2	1,21	0,27	0,10	0,47	0	-1,03	-0,04	0,80	1,00	0	-1,06	-0,10	0
POLA2	polymerase (DNA directed), alpha 2 (70kD subunit)	1,21	0,27	0,23	0,63	0	1,06	0,08	0,54	1,00	0	NA	NA	0
TNFRSF25	tumor necrosis factor receptor superfamily, member 25	1,21	0,27	0,43	0,78	0	-1,09	-0,13	0,49	1,00	0	NA	NA	0
APOBEC3B	apolipoprotein B mRNA editing enzyme, catalytic polypeptide-like 3B	1,21	0,27	0,57	0,86	0	-1,18	-0,24	0,44	1,00	0	NA	NA	0
STXBP1	syntaxin binding protein 1	1,21	0,27	0,17	0,57	0	1,03	0,04	0,71	1,00	0	NA	NA	0
GINS3	GINS complex subunit 3 (Psf3 homolog)	1,21	0,27	0,09	0,45	0	1,67	0,74	0,00	0,44	0	NA	NA	0
C1orf144	chromosome 1 open reading frame 144	1,21	0,27	0,16	0,56	0	1,26	0,33	0,26	1,00	0	NA	NA	0
PLD2	phospholipase D2	1,21	0,27	0,33	0,72	0	1,14	0,18	0,19	1,00	0	NA	NA	0
IKBKE	inhibitor of kappa light polypeptide gene enhancer in B-cells, kinase epsilon	1,20	0,27	0,38	0,75	0	1,02	0,03	0,84	1,00	0	1,20	0,26	0
FDX1L	ferredoxin 1-like	1,20	0,27	0,16	0,56	0	NA	NA	NA	NA	0	NA	NA	0
KLF4	Kruppel-like factor 4 (gut)	1,20	0,27	0,14	0,54	0	1,26	0,33	0,02	0,94	0	1,04	0,06	0
KLF6	Kruppel-like factor 6	1,20	0,27	0,56	0,85	0	1,12	0,16	0,52	1,00	0	-1,27	-0,34	0
NUP188	nucleoporin 188kDa	1,20	0,27	0,10	0,47	0	-1,03	-0,05	0,75	1,00	0	NA	NA	0
EDEM3	ER degradation enhancer, mannosidase alpha-like 3	1,20	0,27	0,32	0,71	0	1,03	0,04	0,76	1,00	0	-1,08	-0,10	0
POLRMT	polymerase (RNA) mitochondrial (DNA directed)	1,20	0,27	0,23	0,63	0	-1,18	-0,24	0,40	1,00	0	NA	NA	0
NEIL3	nei endonuclease VIII-like 3 (E. coli)	1,20	0,27	0,37	0,74	0	1,00	0,00	0,98	1,00	0	NA	NA	0
GNB5	guanine nucleotide binding protein (G protein), beta 5	1,20	0,27	0,19	0,59	0	-1,34	-0,42	0,25	1,00	0	NA	NA	0
UTP20	UTP20, small subunit (SSU) processome component, homolog (yeast)	1,20	0,27	0,26	0,66	0	-1,05	-0,07	0,79	1,00	0	NA	NA	0
RIPK2	receptor-interacting serine-threonine kinase 2	1,20	0,27	0,31	0,70	0	1,07	0,10	0,40	1,00	0	0,00	0,01	0
SLC46A3	solute carrier family 46, member 3	1,20	0,26	0,23	0,63	0	-1,19	-0,25	0,28	1,00	0	NA	NA	0
DGCR8	DiGeorge syndrome critical region gene 8	1,20	0,26	0,08	0,42	0	1,05	0,07	0,78	1,00	0	NA	NA	0
DDX3X	DEAD (Asp-Glu-Ala-Asp) box polypeptide 3, X-linked	1,20	0,26	0,28	0,68	0	1,13	0,18	0,84	1,00	0	-1,12	-0,16	0
CREB3L2	cAMP responsive element binding protein 3-like 2	1,20	0,26	0,11	0,49	0	1,09	0,12	0,45	1,00	0	-1,10	-0,13	0
EGR1	early growth response 1	1,20	0,26	0,21	0,62	0	-1,00	0,00	0,99	1,00	0	NA	NA	0
CEBPD	CCAAT/enhancer binding protein (C/EBP), delta	1,20	0,26	0,50	0,82	0	1,12	0,17	0,31	1,00	0	1,31	0,39	0
TUBD1	tubulin, delta 1	1,20	0,26	0,22	0,62	0	1,87	0,90	0,00	0,25	0	NA	NA	0
KLHL36	kelch-like 36 (Drosophila)	1,20	0,26	0,23	0,64	0	-1,07	-0,10	0,39	1,00	0	NA	NA	0
XPO7	exportin 7	1,20	0,26	0,30	0,69	0	-1,39	-0,47	0,24	1,00	0	-1,47	-0,55	0
PLBD1	phospholipase B domain containing 1	1,20	0,26	0,22	0,63	0	1,51	0,59	0,11	1,00	0	NA	NA	0
CANX	calnexin	1,20	0,26	0,21	0,62	0	-1,14	-0,18	0,76	1,00	0	-1,28	-0,36	0
UBE4B	ubiquitination factor E4B (UFD2 homolog, yeast)	1,20	0,26	0,27	0,67	0	-1,46	-0,54	0,23	1,00	0	-1,10	-0,13	0
CHAF1A	chromatin assembly factor 1, subunit A (p150)	1,20	0,26	0,18	0,59	0	-1,06	-0,09	0,59	1,00	0	NA	NA	0
BIN1	bridging integrator 1	1,20	0,26	0,16	0,56	0	1,13	0,17	0,57	1,00	0	1,20	0,26	0
KIF22	kinesin family member 22	1,20	0,26	0,06	0,40	0	1,02	0,03	0,86	1,00	0	-1,05	-0,08	0
AURKAIP1	aurora kinase A interacting protein 1	1,20	0,26	0,08	0,43	0	NA	NA	NA	NA	0	NA	NA	0
KIF23	kinesin family member 23	1,20	0,26	0,12	0,51	0	1,06	0,08	0,60	1,00	0	NA	NA	0
GLT8D2	glycosyltransferase 8 domain containing 2	1,20	0,26	0,56	0,85	0	-1,01	-0,01	NA	NA	0	-1,39	-0,48	0
RNF25	ring finger protein 25	1,20	0,26	0,11	0,49	0	-1,09	-0,12	0,21	1,00	0	NA	NA	0
SPTLC1	serine palmitoyltransferase, long chain base subunit 1	1,20	0,26	0,09	0,45	0	-1,00	0,00	1,00	1,00	0	-1,25	-0,32	0
GGCT	gamma-glutamylcyclotransferase	1,20	0,26	0,32	0,71	0	1,04	0,05	0,65	1,00	0	NA	NA	0
CAB39	calcium binding protein 39	1,20	0,26	0,07	0,40	0	1,13	0,18	0,18	1,00	0	NA	NA	0
CDC42	cell division cycle 42 (GTP binding protein, 25kDa)	1,20	0,26	0,21	0,61	0	1,28	0,36	0,02	0,86	0	NA	NA	0
CALU	calumenin	1,19	0,26	0,13	0,52	0	-1,12	-0,16	0,54	1,00	0	NA	NA	0
C1orf112	chromosome 1 open reading frame 112	1,19	0,26	0,37	0,75	0	1,16	0,21	0,28	1,00	0	NA	NA	0
DLGAP4	discs, large (Drosophila) homolog-associated protein 4	1,19	0,26	0,18	0,58	0	1,17	0,22	0,17	1,00	0	NA	NA	0
LCMT1	leucine carboxyl methyltransferase 1	1,19	0,26	0,15	0,55	0	1,08	0,11	0,49	1,00	0	NA	NA	0
TTC3	tetratricopeptide repeat domain 3	1,19	0,26	0,35	0,73	0	-1,04	-0,06	0,66	1,00	0	-1,28	-0,35	0
SIRPA	signal-regulatory protein alpha	1,19	0,26	0,14	0,53	0	1,08	0,11	0,54	1,00	0	0,00	0,00	0
YLPM1	YLP motif containing 1	1,19	0,26	0,24	0,65	0	1,03	0,04	0,80	1,00	0	NA	NA	0
CLDN4	claudin 4	1,19	0,26	0,57	0,85	0	-1,28	-0,36	0,50	1,00	0	NA	NA	0
LIG4	ligase IV, DNA, ATP-dependent	1,19	0,26	0,10	0,48	0	1,12	0,16	0,78	1,00	0	-1,47	-0,55	0
UCK2	uridine-cytidine kinase 2	1,19	0,25	0,33	0,72	0	1,02	0,02	0,86	1,00	0	NA	NA	0
SUB1	SUB1 homolog (S. cerevisiae)	1,19	0,25	0,28	0,68	0	1,28	0,36	0,17	1,00	0	NA	NA	0
CBR3	carbonyl reductase 3	1,19	0,25	0,42	0,78	0	1,00	0,00	0,99	1,00	0	-1,14	-0,19	0
XYLT1	xylosyltransferase I	1,19	0,25	0,23	0,63	0	1,02	0,03	0,84	1,00	0	-1,19	-0,25	0
B3GNT3	UDP-GlcNAc:betaGal beta-1,3-N-acetylglucosaminyltransferase 3	1,19	0,25	0,49	0,81	0	1,13	0,18	0,61	1,00	0	NA	NA	0
PAPPA	pregnancy-associated plasma protein A, pappalysin 1	1,19	0,25	0,49	0,81	0	-1,00	-0,01	NA	NA	0	2,11	1,08	0
SPAG9	sperm associated antigen 9	1,19	0,25	0,31	0,70	0	1,26	0,33	0,58	1,00	0	-1,15	-0,20	0
LRP4	low density lipoprotein receptor-related protein 4	1,19	0,25	0,20	0,61	0	-1,12	-0,16	0,57	1,00	0	NA	NA	0
PITPNA	phosphatidylinositol transfer protein, alpha	1,19	0,25	0,14	0,53	0	-1,14	-0,19	0,12	1,00	0	NA	NA	0
AKAP12	A kinase (PRKA) anchor protein 12	1,19	0,25	0,40	0,76	0	-1,19	-0,25	0,41	1,00	0	1,01	0,02	0
MCTP2	multiple C2 domains, transmembrane 2	1,19	0,25	0,24	0,64	0	-1,02	-0,02	0,90	1,00	0	NA	NA	0
RPAIN	RPA interacting protein	1,19	0,25	0,46	0,79	0	1,13	0,17	0,72	1,00	0	NA	NA	0
SBNO2	strawberry notch homolog 2 (Drosophila)	1,19	0,25	0,24	0,64	0	1,08	0,11	0,83	1,00	0	NA	NA	0
RANGRF	RAN guanine nucleotide release factor	1,19	0,25	0,18	0,59	0	1,04	0,05	0,74	1,00	0	NA	NA	0
RGNEF	190 kDa guanine nucleotide exchange factor	1,19	0,25	0,46	0,79	0	1,13	0,18	0,41	1,00	0	1,04	0,06	0
COL4A2	collagen, type IV, alpha 2	1,19	0,25	0,47	0,80	0	1,01	0,01	0,97	1,00	0	1,28	0,36	0
SNX16	sorting nexin 16	1,19	0,25	0,31	0,70	0	-1,23	-0,29	0,11	1,00	0	NA	NA	0
EIF4E	eukaryotic translation initiation factor 4E	1,19	0,25	0,28	0,68	0	-1,08	-0,11	0,49	1,00	0	NA	NA	0
TUFT1	tuftelin 1	1,19	0,25	0,22	0,63	0	1,19	0,25	0,05	1,00	0	-1,10	-0,13	0
SSRP1	structure specific recognition protein 1	1,19	0,25	0,47	0,80	0	-1,31	-0,39	0,02	0,95	0	NA	NA	0
SNRNP40	small nuclear ribonucleoprotein 40kDa (U5)	1,19	0,25	0,33	0,71	0	1,07	0,10	0,74	1,00	0	NA	NA	0
GLIPR1	GLI pathogenesis-related 1	1,19	0,24	0,26	0,66	0	-1,02	-0,02	0,95	1,00	0	NA	NA	0
CHST11	carbohydrate (chondroitin 4) sulfotransferase 11	1,18	0,24	0,48	0,81	0	1,08	0,11	0,65	1,00	0	NA	NA	0
SPAG9	sperm associated antigen 9	1,18	0,24	0,11	0,49	0	1,16	0,21	0,09	1,00	0	NA	NA	0
WDR46	WD repeat domain 46	1,18	0,24	0,17	0,57	0	-1,07	-0,10	0,48	1,00	0	NA	NA	0
NUMB	numb homolog (Drosophila)	1,18	0,24	0,30	0,69	0	-1,12	-0,16	0,13	1,00	0	NA	NA	0
MYO6	myosin VI	1,18	0,24	0,38	0,75	0	1,12	0,17	0,67	1,00	0	-1,89	-0,92	0
TNFRSF1B	tumor necrosis factor receptor superfamily, member 1B	1,18	0,24	0,39	0,76	0	1,01	0,02	0,93	1,00	0	1,04	0,06	0
TNIP2	TNFAIP3 interacting protein 2	1,18	0,24	0,37	0,74	0	1,00	0,00	0,98	1,00	0	NA	NA	0
NBN	nibrin	1,18	0,24	0,26	0,66	0	-1,13	-0,17	0,64	1,00	0	-1,19	-0,26	0
MMP14	matrix metallopeptidase 14 (membrane-inserted)	1,18	0,24	0,13	0,52	0	-1,00	0,00	1,00	1,00	0	1,02	0,03	0
SMARCA4	SWI/SNF related, matrix associated, actin dependent regulator of chromatin, subfamily a, member 4	1,18	0,24	0,57	0,85	0	-1,17	-0,23	0,20	1,00	0	NA	NA	0
BCR	breakpoint cluster region	1,18	0,24	0,31	0,70	0	-1,15	-0,20	0,39	1,00	0	NA	NA	0
TM4SF1	transmembrane 4 L six family member 1	1,18	0,24	0,18	0,59	0	-1,02	-0,03	0,79	1,00	0	-1,30	-0,38	0
C9orf16	chromosome 9 open reading frame 16	1,18	0,24	0,25	0,65	0	1,06	0,09	0,70	1,00	0	NA	NA	0
UBAP2L	ubiquitin associated protein 2-like	1,18	0,24	0,19	0,59	0	-1,04	-0,05	0,74	1,00	0	NA	NA	0
SOX9	SRY (sex determining region Y)-box 9	1,18	0,24	0,45	0,79	0	-1,17	-0,23	0,26	1,00	0	NA	NA	0
ST14	suppression of tumorigenicity 14 (colon carcinoma)	1,18	0,24	0,52	0,83	0	1,12	0,16	0,37	1,00	0	NA	NA	0
C2CD2	C2 calcium-dependent domain containing 2	1,18	0,24	0,12	0,50	0	-1,08	-0,11	0,35	1,00	0	NA	NA	0
WARS	tryptophanyl-tRNA synthetase	1,18	0,24	0,47	0,80	0	-1,21	-0,28	0,07	1,00	0	-1,18	-0,23	0
YIPF6	Yip1 domain family, member 6	1,18	0,24	0,32	0,71	0	1,10	0,13	0,47	1,00	0	-1,08	-0,10	0
RPS6KB1	ribosomal protein S6 kinase, 70kDa, polypeptide 1	1,18	0,24	0,31	0,70	0	1,35	0,44	0,43	1,00	0	-1,35	-0,43	0
ADNP2	ADNP homeobox 2	1,18	0,24	0,16	0,57	0	-1,01	-0,01	0,91	1,00	0	-1,52	-0,59	0
NEDD4L	neural precursor cell expressed, developmentally down-regulated 4-like	1,18	0,24	0,42	0,78	0	-1,17	-0,23	0,38	1,00	0	1,24	0,31	0
CDK2AP2	cyclin-dependent kinase 2 associated protein 2	1,18	0,24	0,33	0,72	0	-1,19	-0,25	0,24	1,00	0	NA	NA	0
NA	NA	1,18	0,24	0,53	0,84	0	-1,37	-0,46	0,10	1,00	0	NA	NA	0
ESF1	ESF1, nucleolar pre-rRNA processing protein, homolog (S. cerevisiae)	1,18	0,24	0,08	0,44	0	-1,12	-0,16	0,43	1,00	0	NA	NA	0
IVD	isovaleryl-CoA dehydrogenase	1,18	0,24	0,35	0,73	0	1,15	0,20	0,15	1,00	0	NA	NA	0
RAP1GAP2	RAP1 GTPase activating protein 2	1,18	0,24	0,44	0,78	0	1,08	0,12	0,25	1,00	0	1,15	0,20	0
ATP6V1B2	ATPase, H+ transporting, lysosomal 56/58kDa, V1 subunit B2	1,18	0,24	0,15	0,55	0	1,11	0,15	0,34	1,00	0	NA	NA	0
NTHL1	nth endonuclease III-like 1 (E. coli)	1,18	0,24	0,11	0,49	0	-1,04	-0,06	0,81	1,00	0	NA	NA	0
JUP	junction plakoglobin	1,18	0,24	0,17	0,57	0	1,13	0,17	0,22	1,00	0	1,02	0,04	0
PURB	purine-rich element binding protein B	1,18	0,24	0,15	0,55	0	NA	NA	NA	NA	0	NA	NA	0
CCDC94	coiled-coil domain containing 94	1,18	0,24	0,43	0,78	0	1,06	0,09	0,40	1,00	0	NA	NA	0
NFYA	nuclear transcription factor Y, alpha	1,18	0,24	0,23	0,63	0	-1,22	-0,28	0,08	1,00	0	-1,27	-0,34	0
AIFM1	apoptosis-inducing factor, mitochondrion-associated, 1	1,18	0,24	0,29	0,68	0	1,02	0,03	0,81	1,00	0	NA	NA	0
PDE8A	phosphodiesterase 8A	1,18	0,24	0,62	0,88	0	1,16	0,21	0,26	1,00	0	-1,33	-0,42	0
RPS6KA2	ribosomal protein S6 kinase, 90kDa, polypeptide 2	1,18	0,24	0,41	0,77	0	1,02	0,02	0,91	1,00	0	-1,18	-0,24	0
C12orf49	chromosome 12 open reading frame 49	1,18	0,24	0,53	0,83	0	1,17	0,23	0,25	1,00	0	-1,10	-0,14	0
TTC3	tetratricopeptide repeat domain 3	1,18	0,24	0,40	0,76	0	-1,13	-0,18	0,75	1,00	0	-1,72	-0,79	0
NA	NA	1,18	0,24	0,13	0,52	0	-1,04	-0,06	0,70	1,00	0	NA	NA	0
SCG5	secretogranin V (7B2 protein)	1,18	0,23	0,29	0,68	0	1,74	0,80	0,03	1,00	0	-1,03	-0,04	0

ATAD2B	ATPase family, AAA domain containing 2B	1,18	0,23	0,36	0,74	0	-1,07	-0,10	0,65	1,00	0	NA	NA	0
PRRG4	proline rich Gla (G-carboxyglutamic acid) 4 (transmembrane)	1,18	0,23	0,67	0,90	0	-1,01	-0,02	0,91	1,00	0	0,00	0,01	0
ETS2	v-ets erythroblastosis virus E26 oncogene homolog 2 (avian)	1,18	0,23	0,42	0,78	0	1,08	0,12	0,59	1,00	0	NA	NA	0
C14orf169	chromosome 14 open reading frame 169	1,18	0,23	0,16	0,56	0	1,01	0,01	0,95	1,00	0	NA	NA	0
SLAMF7	SLAM family member 7	1,18	0,23	0,51	0,82	0	NA	NA	NA	NA	0	NA	NA	0
UGGT2	UDP-glucose glycoprotein glucosyltransferase 2	1,17	0,23	0,42	0,78	0	-1,04	-0,05	0,90	1,00	0	-1,06	-0,10	0
WNT5A	wingless-type MMTV integration site family, member 5A	1,17	0,23	0,24	0,65	0	1,27	0,34	0,20	1,00	0	0,00	0,00	0
NA	NA	1,17	0,23	0,31	0,70	0	-1,45	-0,54	0,12	1,00	0	NA	NA	0
NT5DC2	5'-nucleotidase domain containing 2	1,17	0,23	0,37	0,74	0	-1,01	-0,01	0,98	1,00	0	1,27	0,34	0
NT5C2	5'-nucleotidase, cytosolic II	1,17	0,23	0,34	0,72	0	1,08	0,11	0,43	1,00	0	NA	NA	0
C17orf91	chromosome 17 open reading frame 91	1,17	0,23	0,22	0,62	0	1,22	0,28	0,14	1,00	0	-1,02	-0,03	0
LGALS8	lectin, galactoside-binding, soluble, 8	1,17	0,23	0,28	0,68	0	1,07	0,10	0,35	1,00	0	NA	NA	0
WDHD1	WD repeat and HMG-box DNA binding protein 1	1,17	0,23	0,38	0,75	0	-1,07	-0,10	0,76	1,00	0	-1,47	-0,56	0
LRP10	low density lipoprotein receptor-related protein 10	1,17	0,23	0,31	0,70	0	-1,05	-0,06	0,69	1,00	0	NA	NA	0
DNAJC10	DnaJ (Hsp40) homolog, subfamily C, member 10	1,17	0,23	0,28	0,68	0	-1,01	-0,02	0,96	1,00	0	-1,20	-0,28	0
MAN1A2	mannosidase, alpha, class 1A, member 2	1,17	0,23	0,39	0,76	0	-1,01	-0,01	0,95	1,00	0	-1,14	-0,18	0
CYP3A5	cytochrome P450, family 3, subfamily A, polypeptide 5	1,17	0,23	0,42	0,78	0	-1,05	-0,07	0,84	1,00	0	NA	NA	0
IL13RA2	interleukin 13 receptor, alpha 2	1,17	0,23	0,42	0,77	0	-1,00	-0,01	0,98	1,00	0	1,64	0,71	0
RORA	RAR-related orphan receptor A	1,17	0,23	0,33	0,71	0	1,26	0,34	0,47	1,00	0	NA	NA	0
CA12	carbonic anhydrase XII	1,17	0,23	0,43	0,78	0	-1,22	-0,29	0,45	1,00	0	NA	NA	0
VEGFC	vascular endothelial growth factor C	1,17	0,23	0,41	0,77	0	-1,01	-0,02	0,88	1,00	0	1,01	0,02	0
GALNT1	UDP-N-acetyl-alpha-D-galactosamine:polypeptide N-acetylgalactosaminyltransferase 1 (GalNAc-T1)	1,17	0,23	0,25	0,65	0	-1,02	-0,03	0,81	1,00	0	NA	NA	0
ACY1	aminoacylase 1	1,17	0,23	0,23	0,64	0	-1,15	-0,20	0,10	1,00	0	NA	NA	0
STX4	syntaxin 4	1,17	0,23	0,24	0,64	0	1,05	0,08	0,48	1,00	0	NA	NA	0
TUBGCP5	tubulin, gamma complex associated protein 5	1,17	0,23	0,27	0,67	0	1,10	0,13	0,67	1,00	0	NA	NA	0
PAWR	PRKC, apoptosis, WT1, regulator	1,17	0,23	0,59	0,86	0	1,03	0,05	0,75	1,00	0	NA	NA	0
MCM7	minichromosome maintenance complex component 7	1,17	0,23	0,24	0,65	0	-1,04	-0,06	0,61	1,00	0	NA	NA	0
ARTN	artemin	1,17	0,23	0,55	0,85	0	1,07	0,10	0,48	1,00	0	NA	NA	0
PCYT1A	phosphate cytidylyltransferase 1, choline, alpha	1,17	0,23	0,39	0,75	0	1,13	0,17	0,60	1,00	0	1,14	0,19	0
MAFB	v-maf musculoaponeurotic fibrosarcoma oncogene homolog B (avian)	1,17	0,23	0,42	0,78	0	1,68	0,75	0,02	0,88	0	NA	NA	0
NA	NA	1,17	0,23	0,16	0,56	0	1,02	0,04	0,77	1,00	0	-1,02	-0,03	0
C21orf7	chromosome 21 open reading frame 7	1,17	0,23	0,27	0,67	0	1,20	0,26	0,04	1,00	0	NA	NA	0
GBP5	guanylate binding protein 5	1,17	0,23	0,71	0,91	0	NA	NA	NA	NA	0	NA	NA	0
NLE1	notchless homolog 1 (Drosophila)	1,17	0,23	0,35	0,73	0	-1,14	-0,19	0,27	1,00	0	NA	NA	0
SYMPK	symplekin	1,17	0,23	0,26	0,66	0	-1,17	-0,23	0,08	1,00	0	NA	NA	0
DLG1	discs, large homolog 1 (Drosophila)	1,17	0,23	0,10	0,47	0	1,06	0,08	0,76	1,00	0	-1,22	-0,28	0
METAP2	methionyl aminopeptidase 2	1,17	0,23	0,12	0,50	0	-1,02	-0,02	0,86	1,00	0	NA	NA	0
NUAK1	NUAK family, SNF1-like kinase, 1	1,17	0,23	0,29	0,69	0	1,23	0,29	0,33	1,00	0	-1,02	-0,03	0
GABPB1	GA binding protein transcription factor, beta subunit 1	1,17	0,23	0,33	0,72	0	1,29	0,37	0,09	1,00	0	NA	NA	0
RAD51AP1	RAD51 associated protein 1	1,17	0,23	0,29	0,68	0	-1,07	-0,10	0,37	1,00	0	NA	NA	0
APPL2	adaptor protein, phosphotyrosine interaction, PH domain and leucine zipper containing 2	1,17	0,22	0,20	0,60	0	1,04	0,06	0,85	1,00	0	1,32	0,40	0
RNASEH2B	ribonuclease H2, subunit B	1,17	0,22	0,51	0,82	0	-1,13	-0,18	0,59	1,00	0	NA	NA	0
DUSP14	dual specificity phosphatase 14	1,17	0,22	0,34	0,73	0	1,08	0,11	0,39	1,00	0	-1,04	-0,07	0
CCDC99	coiled-coil domain containing 99	1,17	0,22	0,42	0,78	0	1,17	0,23	0,22	1,00	0	-1,27	-0,33	0
NA	NA	1,17	0,22	0,30	0,70	0	-1,16	-0,21	0,27	1,00	0	-1,25	-0,31	0
UBIAD1	UbiA prenyltransferase domain containing 1	1,17	0,22	0,14	0,54	0	-1,02	-0,03	0,83	1,00	0	NA	NA	0
RIPK2	receptor-interacting serine-threonine kinase 2	1,17	0,22	0,52	0,83	0	-1,12	-0,16	0,36	1,00	0	1,02	0,03	0
SLC25A11	solute carrier family 25 (mitochondrial carrier; oxoglutarate carrier), member 11	1,17	0,22	0,33	0,72	0	-1,17	-0,23	0,17	1,00	0	NA	NA	0
TNFAIP3	tumor necrosis factor, alpha-induced protein 3	1,17	0,22	0,28	0,68	0	1,10	0,13	0,59	1,00	0	NA	NA	0
ERMP1	endoplasmic reticulum metallopeptidase 1	1,17	0,22	0,14	0,54	0	1,01	0,02	0,93	1,00	0	NA	NA	0
LARP1	La ribonucleoprotein domain family, member 1	1,17	0,22	0,22	0,63	0	1,12	0,16	0,22	1,00	0	1,81	0,86	0
SLC1A4	solute carrier family 1 (glutamate/neutral amino acid transporter), member 4	1,17	0,22	0,37	0,74	0	-1,03	-0,05	0,85	1,00	0	-1,09	-0,12	0
TBX3	T-box 3	1,17	0,22	0,23	0,63	0	1,18	0,23	0,14	1,00	0	NA	NA	0
ERF	Ets2 repressor factor	1,17	0,22	0,23	0,63	0	-1,04	-0,05	0,85	1,00	0	NA	NA	0
HMGB3	high-mobility group box 3	1,17	0,22	0,19	0,59	0	-1,06	-0,08	0,64	1,00	0	NA	NA	0
SMC1A	structural maintenance of chromosomes 1A	1,17	0,22	0,17	0,58	0	-1,03	-0,04	0,62	1,00	0	NA	NA	0
DDX17	DEAD (Asp-Glu-Ala-Asp) box polypeptide 17	1,17	0,22	0,39	0,76	0	-1,00	-0,01	NA	NA	0	-1,04	-0,06	0
SMARCA4	SWI/SNF related, matrix associated, actin dependent regulator of chromatin, subfamily a, member 4	1,16	0,22	0,48	0,81	0	-1,28	-0,36	0,14	1,00	0	NA	NA	0
CEP170	centrosomal protein 170kDa	1,16	0,22	0,46	0,80	0	1,28	0,36	0,33	1,00	0	NA	NA	0
IDE	insulin-degrading enzyme	1,16	0,22	0,40	0,76	0	1,03	0,04	0,89	1,00	0	-1,35	-0,44	0
WSB1	WD repeat and SOCS box-containing 1	1,16	0,22	0,47	0,80	0	1,00	0,01	0,96	1,00	0	-1,19	-0,26	0
WDR74	WD repeat domain 74	1,16	0,22	0,25	0,65	0	-1,09	-0,12	0,45	1,00	0	NA	NA	0
DOCK10	dedicator of cytokinesis 10	1,16	0,22	0,37	0,74	0	-1,01	-0,02	NA	NA	0	-1,20	-0,27	0
FAM120A	family with sequence similarity 120A	1,16	0,22	0,24	0,64	0	1,27	0,35	0,71	1,00	0	-1,20	-0,28	0
HERPUD1	homocysteine-inducible, endoplasmic reticulum stress-inducible, ubiquitin-like domain member 1	1,16	0,22	0,26	0,66	0	-1,17	-0,23	0,09	1,00	0	-1,02	-0,04	0
KIAA0513	KIAA0513	1,16	0,22	0,28	0,68	0	1,10	0,14	0,41	1,00	0	-1,11	-0,14	0
CTSO	cathepsin O	1,16	0,22	0,44	0,79	0	-1,02	-0,03	0,94	1,00	0	-1,22	-0,28	0
HSPA4L	heat shock 70kDa protein 4-like	1,16	0,22	0,47	0,80	0	1,04	0,06	0,62	1,00	0	NA	NA	0
NDST2	N-deacetylase/N-sulfotransferase (heparan glucosaminyl) 2	1,16	0,22	0,11	0,48	0	1,13	0,17	0,08	1,00	0	NA	NA	0
SLC2A3	solute carrier family 2 (facilitated glucose transporter), member 3	1,16	0,22	0,52	0,83	0	-1,92	-0,94	0,00	0,37	0	NA	NA	0
CDC45	cell division cycle 45 homolog (S. cerevisiae)	1,16	0,22	0,10	0,48	0	-1,00	0,00	0,99	1,00	0	NA	NA	0
MFAP5	microfibrillar associated protein 5	1,16	0,22	0,53	0,83	0	-1,26	-0,34	0,02	0,86	0	-1,33	-0,42	0
NCBP2	nuclear cap binding protein subunit 2, 20kDa	1,16	0,22	0,28	0,68	0	-1,04	-0,06	0,73	1,00	0	-1,27	-0,34	0
SOCS6	suppressor of cytokine signaling 6	1,16	0,22	0,31	0,70	0	1,16	0,21	0,13	1,00	0	NA	NA	0
GCH1	GTP cyclohydrolase 1	1,16	0,22	0,50	0,82	0	1,20	0,26	0,13	1,00	0	-1,02	-0,03	0
03-mar	membrane-associated ring finger (C3HC4) 3	1,16	0,22	0,63	0,88	0	1,24	0,31	0,37	1,00	0	-1,20	-0,27	0
WIPF2	WAS/WASL interacting protein family, member 2	1,16	0,22	0,37	0,75	0	1,08	0,11	0,44	1,00	0	NA	NA	0
PLEKHF1	pleckstrin homology domain containing, family F (with FYVE domain) member 1	1,16	0,21	0,37	0,74	0	1,78	0,83	0,00	0,31	0	NA	NA	0
PTP4A1	protein tyrosine phosphatase type IVA, member 1	1,16	0,21	0,15	0,55	0	1,11	0,15	0,08	1,00	0	-1,11	-0,16	0
MREG	melanoregulin	1,16	0,21	0,33	0,72	0	1,12	0,17	0,07	1,00	0	1,20	0,27	0
CFLAR	CASP8 and FADD-like apoptosis regulator	1,16	0,21	0,14	0,53	0	1,06	0,08	0,66	1,00	0	-1,27	-0,34	0
NFKB2	nuclear factor of kappa light polypeptide gene enhancer in B-cells 2 (p49/p100)	1,16	0,21	0,30	0,69	0	-1,00	0,00	1,00	1,00	0	NA	NA	0
BIRC3	baculoviral IAP repeat-containing 3	1,16	0,21	0,52	0,83	0	-1,17	-0,23	0,48	1,00	0	NA	NA	0
SEMA3F	sema domain, immunoglobulin domain (Ig), short basic domain, secreted, (semaphorin) 3F	1,16	0,21	0,30	0,69	0	-1,06	-0,08	0,62	1,00	0	1,08	0,11	0
GNS	glucosamine (N-acetyl)-6-sulfatase	1,16	0,21	0,31	0,70	0	-1,61	-0,68	0,34	1,00	0	1,01	0,02	0
EIF5B	eukaryotic translation initiation factor 5B	1,16	0,21	0,18	0,59	0	-1,30	-0,38	0,27	1,00	0	-1,33	-0,42	0
TNFAIP3	tumor necrosis factor, alpha-induced protein 3	1,16	0,21	0,57	0,85	0	1,17	0,23	0,39	1,00	0	NA	NA	0
EPAS1	endothelial PAS domain protein 1	1,16	0,21	0,15	0,55	0	1,01	0,02	0,86	1,00	0	0,00	-0,01	0
CLCF1	cardiotrophin-like cytokine factor 1	1,16	0,21	0,31	0,70	0	1,28	0,36	0,27	1,00	0	1,44	0,52	0
TRAP1	TNF receptor-associated protein 1	1,16	0,21	0,27	0,67	0	1,07	0,09	0,38	1,00	0	NA	NA	0
MCM5	minichromosome maintenance complex component 5	1,16	0,21	0,26	0,66	0	1,03	0,04	0,73	1,00	0	NA	NA	0
PPP3R1	protein phosphatase 3, regulatory subunit B, alpha	1,16	0,21	0,25	0,66	0	1,36	0,44	0,04	1,00	0	-1,64	-0,71	0
HNRNPUL1	heterogeneous nuclear ribonucleoprotein U-like 1	1,16	0,21	0,19	0,60	0	-1,05	-0,07	0,81	1,00	0	1,48	0,57	0
ZNF654	zinc finger protein 654	1,16	0,21	0,55	0,84	0	-1,03	-0,05	0,81	1,00	0	NA	NA	0
CCNL1	cyclin L1	1,16	0,21	0,35	0,73	0	1,24	0,31	0,07	1,00	0	1,04	0,05	0
SLC7A5	solute carrier family 7 (cationic amino acid transporter, y+ system), member 5	1,16	0,21	0,20	0,60	0	1,01	0,01	0,90	1,00	0	-1,18	-0,23	0
RRBP1	ribosome binding protein 1 homolog 180kDa (dog)	1,16	0,21	0,26	0,66	0	-1,00	0,00	NA	NA	0	-1,16	-0,22	0
LYPD3	LY6/PLAUR domain containing 3	1,15	0,21	0,14	0,54	0	1,19	0,25	0,26	1,00	0	NA	NA	0
SIK3	SIK family kinase 3	1,15	0,21	0,20	0,60	0	1,05	0,07	0,73	1,00	0	-1,03	-0,05	0
CHMP6	chromatin modifying protein 6	1,15	0,21	0,42	0,77	0	1,14	0,19	0,31	1,00	0	NA	NA	0
CAMSAP1L1	calmodulin regulated spectrin-associated protein 1-like 1	1,15	0,21	0,23	0,63	0	-1,07	-0,10	0,23	1,00	0	NA	NA	0
TTC12	tetratricopeptide repeat domain 12	1,15	0,21	0,25	0,65	0	-1,11	-0,15	0,59	1,00	0	NA	NA	0
NA	NA	1,15	0,21	0,52	0,83	0	-1,97	-0,98	0,02	0,92	0	NA	NA	0
PDIA4	protein disulfide isomerase family A, member 4	1,15	0,21	0,20	0,60	0	-1,09	-0,13	0,62	1,00	0	NA	NA	0
PCIF1	PDX1 C-terminal inhibiting factor 1	1,15	0,21	0,59	0,86	0	1,02	0,03	0,87	1,00	0	1,39	0,48	0
NF2	neurofibromin 2 (merlin)	1,15	0,21	0,37	0,74	0	1,05	0,07	0,61	1,00	0	NA	NA	0
MCM6	minichromosome maintenance complex component 6	1,15	0,21	0,37	0,75	0	-1,02	-0,03	0,83	1,00	0	NA	NA	0
ITPK1	inositol 1,3,4-triphosphate 5/6 kinase	1,15	0,21	0,20	0,61	0	-1,13	-0,18	0,18	1,00	0	NA	NA	0
ZNF785	zinc finger protein 785	1,15	0,20	0,54	0,84	0	NA	NA	NA	NA	0	NA	NA	0
C11orf24	chromosome 11 open reading frame 24	1,15	0,20	0,21	0,62	0	1,06	0,09	0,50	1,00	0	NA	NA	0
PTPN2	protein tyrosine phosphatase, non-receptor type 2	1,15	0,20	0,32	0,71	0	1,19	0,25	0,03	1,00	0	NA	NA	0
LRRFIP2	leucine rich repeat (in FLII) interacting protein 2	1,15	0,20	0,31	0,70	0	1,03	0,05	0,73	1,00	0	NA	NA	0
METTL1	methyltransferase like 1	1,15	0,20	0,27	0,67	0	1,10	0,14	0,43	1,00	0	NA	NA	0
CDH13	cadherin 13, H-cadherin (heart)	1,15	0,20	0,36	0,74	0	-1,04	-0,06	0,66	1,00	0	1,06	0,09	0
CHD8	chromodomain helicase DNA binding protein 8	1,15	0,20	0,33	0,72	0	-1,16	-0,21	0,19	1,00	0	1,23	0,29	0
PPP1R10	protein phosphatase 1, regulatory (inhibitor) subunit 10	1,15	0,20	0,48	0,81	0	1,14	0,20	0,26	1,00	0	-1,06	-0,10	0
C9orf167	chromosome 9 open reading frame 167	1,15	0,20	0,39	0,76	0	-1,37	-0,45	0,01	0,63	0	NA	NA	0
H2AFV	H2A histone family, member V	1,15	0,20	0,43	0,78	0	1,00	0,01	0,97	1,00	0	1,04	0,05	0
ACACA	acetyl-CoA carboxylase alpha	1,15	0,20	0,20	0,60	0	-1,07	-0,10	0,34	1,00	0	NA	NA	0
PCDH7	protocadherin 7	1,15	0,20	0,75	0,93	0	-1,11	-0,15	0,62	1,00	0	NA	NA	0
RPS28	ribosomal protein S28	1,15	0,20	0,25	0,65	0	1,05	0,07	0,85	1,00	0	NA	NA	0
KIF20B	kinesin family member 20B	1,15	0,20	0,38	0,75	0	-1,00	0,00	1,00	1,00	0	NA	NA	0
GOLM1	golgi membrane protein 1	1,15	0,20	0,43	0,78	0	1,01	0,01	0,94	1,00	0	-1,23	-0,30	0
TMEM110	transmembrane protein 110	1,15	0,20	0,43	0,78	0	-1,02	-0,03	0,79	1,00	0	-1,09	-0,13	0
FKBP1A	FK506 binding protein 1A, 12kDa	1,15	0,20	0,44	0,79	0	-1,21	-0,27	0,35	1,00	0	1,44	0,52	0
TRIM23	tripartite motif-containing 23	1,15	0,20	0,23	0,64	0	-1,09	-0,12	0,62	1,00	0	-1,30	-0,37	0
SLC12A4	solute carrier family 12 (potassium/chloride transporters), member 4	1,15	0,20	0,16	0,56	0	1,10	0,14	0,47	1,00	0	NA	NA	0
NOP56	NOP56 ribonucleoprotein homolog (yeast)	1,15	0,20	0,35	0,73	0	1,12	0,16	0,28	1,00	0	NA	NA	0
PXDN	peroxidasin homolog (Drosophila)	1,15	0,20	0,41	0,77	0	1,30	0,38	0,01	0,64	0	1,04	0,05	0
SLC36A1	solute carrier family 36 (proton/amino acid symporter), member 1	1,15	0,20	0,23	0,64	0	1,03	0,04	0,82	1,00	0	-1,06	-0,09	0
NA	NA	1,15	0,20	0,55	0,84	0	-1,03	-0,04	0,84	1,00	0	NA	NA	0
ELK4	ELK4, ETS-domain protein (SRF accessory protein 1)	1,15	0,20	0,31	0,70	0	-1,06	-0,09	0,68	1,00	0	NA	NA	0
GLS	glutaminase	1,15	0,20	0,51	0,83	0	1,10	0,14	0,71	1,00	0	-1,25	-0,32	0
PAK1IP1	PAK1 interacting protein 1	1,15	0,20	0,43	0,78	0	-1,30	-0,38	0,06	1,00	0	NA	NA	0
H1F0	H1 histone family, member 0	1,15	0,20	0,17	0,57	0	-1,03	-0,04	0,78	1,00	0	NA	NA	0
ATRN	attractin	1,15	0,20	0,22	0,62	0	-1,07	-0,10	0,43	1,00	0	NA	NA	0
LARS2	leucyl-tRNA synthetase 2, mitochondrial	1,15	0,20	0,19	0,59	0	1,19	0,25	0,11	1,00	0	-1,32	-0,40	0

DOLK	dolichol kinase	1,15	0,20	0,20	0,60	0	1,07	0,10	0,52	1,00	0	NA	NA	0
BACH1	BTB and CNC homology 1, basic leucine zipper transcription factor 1	1,15	0,20	0,31	0,70	0	1,07	0,10	0,65	1,00	0	NA	NA	0
SLC30A5	solute carrier family 30 (zinc transporter), member 5	1,15	0,20	0,17	0,58	0	1,07	0,09	0,61	1,00	0	NA	NA	0
HNRNPA3	heterogeneous nuclear ribonucleoprotein A3	1,15	0,20	0,24	0,65	0	-1,07	-0,10	0,76	1,00	0	-1,22	-0,28	0
IL6ST	interleukin 6 signal transducer (gp130, oncostatin M receptor)	1,15	0,20	0,46	0,80	0	1,06	0,08	0,86	1,00	0	-1,12	-0,17	0
ATP1B3	ATPase, Na+/K+ transporting, beta 3 polypeptide	1,14	0,19	0,26	0,66	0	-1,06	-0,09	0,26	1,00	0	NA	NA	0
LGALS8	lectin, galactoside-binding, soluble, 8	1,14	0,19	0,16	0,56	0	1,08	0,11	0,69	1,00	0	NA	NA	0
CSRNP2	cysteine-serine-rich nuclear protein 2	1,14	0,19	0,34	0,73	0	1,13	0,17	0,18	1,00	0	-1,09	-0,12	0
QKI	quaking homolog, KH domain RNA binding (mouse)	1,14	0,19	0,41	0,77	0	1,17	0,23	0,44	1,00	0	NA	NA	0
ZNF672	zinc finger protein 672	1,14	0,19	0,27	0,67	0	-1,02	-0,03	0,90	1,00	0	NA	NA	0
RAD50	RAD50 homolog (S. cerevisiae)	1,14	0,19	0,33	0,71	0	-1,19	-0,25	0,40	1,00	0	NA	NA	0
TRAPPC3	trafficking protein particle complex 3	1,14	0,19	0,18	0,59	0	1,06	0,08	0,56	1,00	0	NA	NA	0
LRRC1	leucine rich repeat containing 1	1,14	0,19	0,24	0,64	0	1,05	0,08	0,54	1,00	0	-1,25	-0,31	0
SERPINB7	serpin peptidase inhibitor, clade B (ovalbumin), member 7	1,14	0,19	0,28	0,68	0	1,03	0,05	0,80	1,00	0	NA	NA	0
KIAA0101	KIAA0101	1,14	0,19	0,38	0,75	0	1,01	0,01	0,92	1,00	0	NA	NA	0
PTHLH	parathyroid hormone-like hormone	1,14	0,19	0,74	0,92	0	1,06	0,08	0,68	1,00	0	NA	NA	0
LONP1	lon peptidase 1, mitochondrial	1,14	0,19	0,38	0,75	0	1,00	0,00	1,00	1,00	0	NA	NA	0
SV2A	synaptic vesicle glycoprotein 2A	1,14	0,19	0,62	0,88	0	-1,01	-0,01	NA	NA	0	1,10	0,13	0
NA	NA	1,14	0,19	0,56	0,85	0	-1,03	-0,04	0,85	1,00	0	NA	NA	0
KLF9	Kruppel-like factor 9	1,14	0,19	0,41	0,77	0	-1,01	-0,02	NA	NA	0	-1,32	-0,39	0
TMEM97	transmembrane protein 97	1,14	0,19	0,39	0,76	0	-1,02	-0,03	0,85	1,00	0	-1,23	-0,30	0
AGPAT5	1-acylglycerol-3-phosphate O-acyltransferase 5 (lysophosphatidic acid acyltransferase, epsilon)	1,14	0,19	0,37	0,75	0	1,01	0,01	0,97	1,00	0	-1,23	-0,31	0
CCNA2	cyclin A2	1,14	0,19	0,53	0,84	0	1,04	0,06	0,75	1,00	0	NA	NA	0
SLC4A7	solute carrier family 4, sodium bicarbonate cotransporter, member 7	1,14	0,19	0,44	0,78	0	-1,02	-0,03	0,84	1,00	0	1,03	0,05	0
CD3EAP	CD3e molecule, epsilon associated protein	1,14	0,19	0,30	0,69	0	1,09	0,13	0,54	1,00	0	NA	NA	0
NKIRAS2	NFKB inhibitor interacting Ras-like 2	1,14	0,19	0,34	0,72	0	1,35	0,43	0,03	0,96	0	NA	NA	0
IDE	insulin-degrading enzyme	1,14	0,19	0,47	0,81	0	1,09	0,13	0,69	1,00	0	-1,27	-0,33	0
RCAN1	regulator of calcineurin 1	1,14	0,19	0,49	0,81	0	1,02	0,03	0,89	1,00	0	1,05	0,07	0
PTGIR	prostaglandin I2 (prostacyclin) receptor (IP)	1,14	0,19	0,46	0,79	0	-1,00	0,00	NA	NA	0	1,16	0,21	0
TUG1	taurine upregulated 1 (non-protein coding)	1,14	0,19	0,30	0,70	0	-1,18	-0,24	0,39	1,00	0	-1,12	-0,17	0
WWP2	WW domain containing E3 ubiquitin protein ligase 2	1,14	0,19	0,25	0,66	0	1,11	0,15	0,25	1,00	0	NA	NA	0
CACNA1A	calcium channel, voltage-dependent, P/Q type, alpha 1A subunit	1,14	0,19	0,50	0,82	0	-1,01	-0,02	NA	NA	0	-1,01	-0,02	0
HSP90B1	heat shock protein 90kDa beta (Grp94), member 1	1,14	0,19	0,32	0,71	0	-1,19	-0,25	0,46	1,00	0	NA	NA	0
DCPS	decapping enzyme, scavenger	1,14	0,19	0,33	0,72	0	1,03	0,05	0,85	1,00	0	NA	NA	0
KCNJ15	potassium inwardly-rectifying channel, subfamily J, member 15	1,14	0,19	0,48	0,81	0	-1,21	-0,28	0,20	1,00	0	NA	NA	0
NSFL1C	NSFL1 (p97) cofactor (p47)	1,14	0,19	0,35	0,73	0	1,26	0,33	0,15	1,00	0	NA	NA	0
MRAS	muscle RAS oncogene homolog	1,14	0,19	0,25	0,65	0	-1,00	-0,01	NA	NA	0	-1,02	-0,03	0
LAS1L	LAS1-like (S. cerevisiae)	1,14	0,18	0,28	0,68	0	1,04	0,05	0,75	1,00	0	NA	NA	0
WDR48	WD repeat domain 48	1,14	0,18	0,22	0,62	0	1,09	0,12	0,53	1,00	0	NA	NA	0
CDK19	cyclin-dependent kinase 19	1,14	0,18	0,42	0,78	0	-1,01	-0,01	0,96	1,00	0	-1,09	-0,12	0
NF2	neurofibromin 2 (merlin)	1,14	0,18	0,42	0,77	0	1,18	0,24	0,36	1,00	0	NA	NA	0
XIAP	X-linked inhibitor of apoptosis	1,14	0,18	0,32	0,71	0	1,01	0,02	0,96	1,00	0	NA	NA	0
CAP2	CAP, adenylate cyclase-associated protein, 2 (yeast)	1,13	0,18	0,44	0,78	0	1,31	0,39	0,02	0,94	0	-1,04	-0,05	0
PKP1	plakophilin 1 (ectodermal dysplasia/skin fragility syndrome)	1,13	0,18	0,47	0,80	0	1,27	0,35	0,32	1,00	0	NA	NA	0
SLC25A32	solute carrier family 25, member 32	1,13	0,18	0,42	0,77	0	1,04	0,06	0,55	1,00	0	NA	NA	0
GINS1	GINS complex subunit 1 (Psf1 homolog)	1,13	0,18	0,46	0,80	0	-1,00	0,00	1,00	1,00	0	NA	NA	0
MT1M	metallothionein 1M	1,13	0,18	0,24	0,64	0	1,02	0,02	NA	NA	0	1,41	0,50	0
LGALS8	lectin, galactoside-binding, soluble, 8	1,13	0,18	0,22	0,62	0	1,11	0,15	0,41	1,00	0	NA	NA	0
DAG1	dystroglycan 1 (dystrophin-associated glycoprotein 1)	1,13	0,18	0,46	0,79	0	1,04	0,05	0,68	1,00	0	NA	NA	0
CYB561	cytochrome b-561	1,13	0,18	0,47	0,80	0	-1,04	-0,05	0,87	1,00	0	NA	NA	0
CD58	CD58 molecule	1,13	0,18	0,54	0,84	0	-1,01	-0,01	0,97	1,00	0	-1,09	-0,12	0
METTL8	methyltransferase like 8	1,13	0,18	0,59	0,86	0	-1,02	-0,03	0,88	1,00	0	1,11	0,15	0
HMGB3L1	high-mobility group box 3-like 1	1,13	0,18	0,31	0,70	0	1,03	0,04	0,81	1,00	0	NA	NA	0
GPR153	G protein-coupled receptor 153	1,13	0,18	0,33	0,71	0	-1,02	-0,03	0,91	1,00	0	NA	NA	0
FAM60A	family with sequence similarity 60, member A	1,13	0,18	0,30	0,69	0	1,11	0,15	0,15	1,00	0	NA	NA	0
DHFR	dihydrofolate reductase	1,13	0,18	0,38	0,75	0	1,04	0,06	0,75	1,00	0	NA	NA	0
APOO	apolipoprotein O	1,13	0,18	0,39	0,76	0	1,29	0,37	0,07	1,00	0	NA	NA	0
SLC48A1	solute carrier family 48 (heme transporter), member 1	1,13	0,18	0,47	0,80	0	-1,06	-0,08	0,62	1,00	0	NA	NA	0
WBP11	WW domain binding protein 11	1,13	0,18	0,55	0,84	0	-1,04	-0,06	0,76	1,00	0	NA	NA	0
SNAPC1	small nuclear RNA activating complex, polypeptide 1, 43kDa	1,13	0,18	0,50	0,82	0	1,00	0,00	0,99	1,00	0	NA	NA	0
KIAA0415	KIAA0415	1,13	0,18	0,32	0,70	0	1,07	0,10	0,55	1,00	0	NA	NA	0
CANT1	calcium activated nucleotidase 1	1,13	0,18	0,41	0,77	0	-1,01	-0,01	0,95	1,00	0	NA	NA	0
PINK1	PTEN induced putative kinase 1	1,13	0,18	0,56	0,85	0	-1,14	-0,19	0,41	1,00	0	1,14	0,19	0
SLC15A3	solute carrier family 15, member 3	1,13	0,18	0,42	0,77	0	-1,01	-0,01	0,95	1,00	0	-1,41	-0,48	0
MRTO4	mRNA turnover 4 homolog (S. cerevisiae)	1,13	0,18	0,43	0,78	0	1,03	0,04	0,86	1,00	0	NA	NA	0
MAPKBP1	mitogen-activated protein kinase binding protein 1	1,13	0,18	0,21	0,61	0	1,01	0,01	0,94	1,00	0	1,43	0,52	0
HNRNPL	heterogeneous nuclear ribonucleoprotein L	1,13	0,18	0,52	0,83	0	-1,10	-0,13	0,59	1,00	0	NA	NA	0
ITGB6	integrin, beta 6	1,13	0,18	0,78	0,94	0	-1,89	-0,92	0,45	1,00	0	NA	NA	0
PML	promyelocytic leukemia	1,13	0,18	0,76	0,93	0	1,09	0,12	0,42	1,00	0	1,27	0,34	0
SCD	stearoyl-CoA desaturase (delta-9-desaturase)	1,13	0,18	0,48	0,81	0	-1,20	-0,27	0,08	1,00	0	NA	NA	0
MCM5	minichromosome maintenance complex component 5	1,13	0,18	0,31	0,70	0	-1,00	0,00	1,00	1,00	0	NA	NA	0
TMEM97	transmembrane protein 97	1,13	0,18	0,51	0,83	0	-1,01	-0,01	0,94	1,00	0	NA	NA	0
UXS1	UDP-glucuronate decarboxylase 1	1,13	0,18	0,18	0,59	0	-1,18	-0,24	0,18	1,00	0	NA	NA	0
PPFIA1	protein tyrosine phosphatase, receptor type, f polypeptide (PTPRF), interacting protein (liprin), alpha 1	1,13	0,18	0,34	0,72	0	1,31	0,38	0,12	1,00	0	NA	NA	0
HERC4	hect domain and RLD 4	1,13	0,18	0,37	0,74	0	-1,07	-0,10	0,81	1,00	0	-1,22	-0,29	0
ABLIM1	actin binding LIM protein 1	1,13	0,18	0,26	0,66	0	-1,07	-0,09	0,42	1,00	0	-1,30	-0,38	0
EEF1E1	eukaryotic translation elongation factor 1 epsilon 1	1,13	0,18	0,56	0,85	0	1,14	0,19	0,36	1,00	0	-1,16	-0,22	0
OGFR	opioid growth factor receptor	1,13	0,17	0,51	0,82	0	1,12	0,16	0,44	1,00	0	1,12	0,17	0
SALL1	sal-like 1 (Drosophila)	1,13	0,17	0,64	0,88	0	-1,01	-0,01	NA	NA	0	1,15	0,20	0
ZNF215	zinc finger protein 215	1,13	0,17	0,50	0,82	0	-1,00	0,00	0,99	1,00	0	NA	NA	0
TNC	tenascin C	1,13	0,17	0,23	0,64	0	1,76	0,82	0,00	0,45	0	1,33	0,41	0
MAP4K5	mitogen-activated protein kinase kinase kinase kinase 5	1,13	0,17	0,48	0,81	0	-1,39	-0,48	0,56	1,00	0	-1,01	-0,01	0
ANTXR1	anthrax toxin receptor 1	1,13	0,17	0,68	0,90	0	-1,02	-0,02	0,89	1,00	0	1,13	0,18	0
CDKN3	cyclin-dependent kinase inhibitor 3	1,13	0,17	0,62	0,88	0	-1,07	-0,10	0,75	1,00	0	NA	NA	0
NUBP1	nucleotide binding protein 1 (MinD homolog, E. coli)	1,13	0,17	0,35	0,73	0	1,01	0,01	0,95	1,00	0	NA	NA	0
BAT2L2	HLA-B associated transcript 2-like 2	1,13	0,17	0,38	0,75	0	-1,03	-0,04	0,81	1,00	0	-2,94	-1,57	0
PIK3IP1	phosphoinositide-3-kinase interacting protein 1	1,13	0,17	0,71	0,91	0	1,10	0,13	0,57	1,00	0	NA	NA	0
BLZF1	basic leucine zipper nuclear factor 1	1,13	0,17	0,31	0,70	0	1,01	0,01	0,93	1,00	0	NA	NA	0
SHOX	short stature homeobox	1,13	0,17	0,40	0,76	0	-1,00	0,00	NA	NA	0	1,36	0,44	0
SYNJ2	synaptojanin 2	1,13	0,17	0,51	0,82	0	1,20	0,26	0,32	1,00	0	NA	NA	0
IFNAR2	interferon (alpha, beta and omega) receptor 2	1,13	0,17	0,64	0,88	0	-1,10	-0,14	0,60	1,00	0	NA	NA	0
HNRNPA1	heterogeneous nuclear ribonucleoprotein A1	1,13	0,17	0,60	0,87	0	1,06	0,09	0,85	1,00	0	NA	NA	0
CBFB	core-binding factor, beta subunit	1,13	0,17	0,41	0,77	0	-1,17	-0,23	0,80	1,00	0	-1,39	-0,48	0
NFYA	nuclear transcription factor Y, alpha	1,13	0,17	0,23	0,64	0	-1,14	-0,19	0,35	1,00	0	-1,41	-0,49	0
TMF1	TATA element modulatory factor 1	1,13	0,17	0,25	0,65	0	-1,03	-0,04	0,74	1,00	0	1,06	0,09	0
RBM12	RNA binding motif protein 12	1,13	0,17	0,31	0,70	0	1,05	0,08	0,67	1,00	0	-1,20	-0,27	0
IL13RA1	interleukin 13 receptor, alpha 1	1,12	0,17	0,29	0,69	0	1,37	0,45	0,42	1,00	0	NA	NA	0
CAMSAP1	calmodulin regulated spectrin-associated protein 1	1,12	0,17	0,48	0,81	0	1,24	0,31	0,10	1,00	0	-1,28	-0,35	0
CDC6	cell division cycle 6 homolog (S. cerevisiae)	1,12	0,17	0,37	0,75	0	-1,17	-0,23	0,13	1,00	0	NA	NA	0
TMEM127	transmembrane protein 127	1,12	0,17	0,25	0,65	0	1,08	0,11	0,57	1,00	0	NA	NA	0
MST1R	macrophage stimulating 1 receptor (c-met-related tyrosine kinase)	1,12	0,17	0,66	0,90	0	-1,23	-0,30	0,06	1,00	0	NA	NA	0
ETV5	ets variant 5	1,12	0,17	0,36	0,74	0	-1,23	-0,30	0,25	1,00	0	NA	NA	0
FER	fer (fps/fes related) tyrosine kinase	1,12	0,17	0,50	0,82	0	1,06	0,08	0,59	1,00	0	0,00	0,00	0
NA	NA	1,12	0,17	0,40	0,76	0	-1,03	-0,04	0,93	1,00	0	-1,02	-0,03	0
DSCC1	defective in sister chromatid cohesion 1 homolog (S. cerevisiae)	1,12	0,17	0,50	0,82	0	1,11	0,15	0,60	1,00	0	NA	NA	0
DKC1	dyskeratosis congenita 1, dyskerin	1,12	0,17	0,39	0,76	0	1,02	0,03	0,78	1,00	0	NA	NA	0
LPPR4	lipid phosphate phosphatase-related protein type 4	1,12	0,17	0,75	0,93	0	-1,01	-0,02	NA	NA	0	1,30	0,38	0
DERL1	Der1-like domain family, member 1	1,12	0,17	0,21	0,62	0	-1,03	-0,05	0,76	1,00	0	NA	NA	0
CITED2	Cbp/p300-interacting transactivator, with Glu/Asp-rich carboxy-terminal domain, 2	1,12	0,17	0,49	0,81	0	1,32	0,40	0,23	1,00	0	NA	NA	0
KNTC1	kinetochore associated 1	1,12	0,17	0,58	0,86	0	-1,17	-0,22	0,26	1,00	0	NA	NA	0
PA2G4	proliferation-associated 2G4, 38kDa	1,12	0,17	0,37	0,74	0	-1,07	-0,10	0,49	1,00	0	NA	NA	0
CHAF1A	chromatin assembly factor 1, subunit A (p150)	1,12	0,17	0,21	0,61	0	-1,01	-0,02	0,86	1,00	0	NA	NA	0
GNL3	guanine nucleotide binding protein-like 3 (nucleolar)	1,12	0,17	0,38	0,75	0	1,07	0,10	0,26	1,00	0	NA	NA	0
PRIM1	primase, DNA, polypeptide 1 (49kDa)	1,12	0,17	0,36	0,74	0	1,05	0,07	0,74	1,00	0	NA	NA	0
IRF3	interferon regulatory factor 3	1,12	0,17	0,41	0,77	0	-1,42	-0,50	0,01	0,51	0	1,43	0,52	0
CD58	CD58 molecule	1,12	0,17	0,58	0,86	0	1,12	0,16	0,28	1,00	0	-1,03	-0,05	0
TAF4	TAF4 RNA polymerase II, TATA box binding protein (TBP)-associated factor, 135kDa	1,12	0,17	0,26	0,66	0	1,03	0,04	0,73	1,00	0	NA	NA	0
PTGS1	prostaglandin-endoperoxide synthase 1 (prostaglandin G/H synthase and cyclooxygenase)	1,12	0,17	0,68	0,90	0	-1,01	-0,01	NA	NA	0	-1,10	-0,13	0
SAT1	spermidine/spermine N1-acetyltransferase 1	1,12	0,16	0,46	0,80	0	1,28	0,36	0,00	0,18	0	1,06	0,08	0
EXOC7	exocyst complex component 7	1,12	0,16	0,52	0,83	0	-1,15	-0,20	0,16	1,00	0	1,05	0,08	0
HLA-E	major histocompatibility complex, class I, E	1,12	0,16	0,48	0,81	0	1,10	0,14	0,44	1,00	0	-1,01	-0,02	0
NAMPT	nicotinamide phosphoribosyltransferase	1,12	0,16	0,32	0,71	0	1,47	0,56	0,00	0,03	0	1,15	0,20	0
HMGA2	high mobility group AT-hook 2	1,12	0,16	0,36	0,74	0	1,05	0,08	0,52	1,00	0	NA	NA	0
EIF3B	eukaryotic translation initiation factor 3, subunit B	1,12	0,16	0,29	0,69	0	1,06	0,08	0,38	1,00	0	NA	NA	0
FADS1	fatty acid desaturase 1	1,12	0,16	0,61	0,87	0	-1,02	-0,03	0,87	1,00	0	NA	NA	0
CHMP1B	chromatin modifying protein 1B	1,12	0,16	0,32	0,71	0	1,15	0,20	0,30	1,00	0	1,06	0,08	0
TMX1	thioredoxin-related transmembrane protein 1	1,12	0,16	0,42	0,78	0	1,45	0,53	0,59	1,00	0	-1,54	-0,62	0
RAB4A	RAB4A, member RAS oncogene family	1,12	0,16	0,40	0,76	0	-1,05	-0,07	0,79	1,00	0	-1,15	-0,19	0
IPO5	importin 5	1,12	0,16	0,31	0,70	0	-1,03	-0,04	0,74	1,00	0	NA	NA	0
AMPD2	adenosine monophosphate deaminase 2	1,12	0,16	0,30	0,69	0	-1,01	-0,01	0,96	1,00	0	NA	NA	0
USE1	unconventional SNARE in the ER 1 homolog (S. cerevisiae)	1,12	0,16	0,42	0,77	0	1,02	0,03	0,93	1,00	0	-1,11	-0,14	0
ATM	ataxia telangiectasia mutated	1,12	0,16	0,39	0,76	0	-1,35	-0,43	0,34	1,00	0	NA	NA	0
MFN1	mitofusin 1	1,12	0,16	0,49	0,81	0	1,16	0,21	0,11	1,00	0	-1,15	-0,20	0
TRMT2A	TRM2 tRNA methyltransferase 2 homolog A (S. cerevisiae)	1,12	0,16	0,32	0,71	0	-1,20	-0,27	0,09	1,00	0	NA	NA	0
EXOC2	exocyst complex component 2	1,12	0,16	0,40	0,76	0	-1,08	-0,12	0,69	1,00	0	NA	NA	0
TBC1D9	TBC1 domain family, member 9 (with GRAM domain)	1,12	0,16	0,47	0,80	0	1,19	0,25	0,05	1,00	0	NA	NA	0
PDZRN3	PDZ domain containing ring finger 3	1,12	0,16	0,56	0,85	0	-1,03	-0,04	0,77	1,00	0	-1,15	-0,20	0

AP1AR	adaptor-related protein complex 1 associated regulatory protein	1,12	0,16	0,57	0,85	0	1,07	0,10	0,72	1,00	0	-1,54	-0,62	0
DIS3	DIS3 mitotic control homolog (S. cerevisiae)	1,12	0,16	0,54	0,84	0	1,12	0,16	0,54	1,00	0	1,39	0,47	0
NUCB2	nucleobindin 2	1,12	0,16	0,41	0,77	0	-1,16	-0,21	0,17	1,00	0	NA	NA	0
FLNB	filamin B, beta	1,12	0,16	0,65	0,89	0	-1,26	-0,34	0,00	0,46	0	NA	NA	0
TMEM50A	transmembrane protein 50A	1,12	0,16	0,28	0,68	0	1,05	0,08	0,45	1,00	0	NA	NA	0
PUS1	pseudouridylate synthase 1	1,12	0,16	0,23	0,63	0	1,05	0,07	0,56	1,00	0	NA	NA	0
SMAD7	SMAD family member 7	1,12	0,16	0,38	0,75	0	1,10	0,13	0,35	1,00	0	NA	NA	0
C3orf52	chromosome 3 open reading frame 52	1,12	0,16	0,56	0,85	0	1,03	0,05	0,74	1,00	0	NA	NA	0
FANCG	Fanconi anemia, complementation group G	1,12	0,16	0,36	0,74	0	1,05	0,07	0,65	1,00	0	NA	NA	0
ACVR2A	activin A receptor, type IIA	1,12	0,16	0,40	0,76	0	-1,40	-0,49	0,01	0,51	0	NA	NA	0
NFIB	nuclear factor I/B	1,12	0,16	0,56	0,85	0	-1,15	-0,20	0,37	1,00	0	-1,11	-0,15	0
RNF41	ring finger protein 41	1,12	0,16	0,40	0,76	0	-1,05	-0,07	0,58	1,00	0	-1,02	-0,04	0
SLC1A4	solute carrier family 1 (glutamate/neutral amino acid transporter), member 4	1,11	0,16	0,53	0,84	0	-1,05	-0,07	0,80	1,00	0	-1,20	-0,27	0
IARS	isoleucyl-tRNA synthetase	1,11	0,16	0,28	0,68	0	-1,05	-0,07	0,44	1,00	0	NA	NA	0
POLE2	polymerase (DNA directed), epsilon 2 (p59 subunit)	1,11	0,16	0,76	0,93	0	1,08	0,10	0,49	1,00	0	-1,41	-0,49	0
SMARCC1	SWI/SNF related, matrix associated, actin dependent regulator of chromatin, subfamily c, member 1	1,11	0,16	0,40	0,76	0	-1,16	-0,21	0,19	1,00	0	NA	NA	0
CA12	carbonic anhydrase XII	1,11	0,16	0,52	0,83	0	1,08	0,11	0,70	1,00	0	NA	NA	0
JMJD6	jumonji domain containing 6	1,11	0,16	0,63	0,88	0	1,19	0,25	0,26	1,00	0	NA	NA	0
FARSA	phenylalanyl-tRNA synthetase, alpha subunit	1,11	0,15	0,45	0,79	0	-1,01	-0,01	0,98	1,00	0	NA	NA	0
TNFRSF25	tumor necrosis factor receptor superfamily, member 25	1,11	0,15	0,61	0,88	0	1,20	0,26	0,53	1,00	0	NA	NA	0
RAB4B	RAB4B, member RAS oncogene family	1,11	0,15	0,28	0,68	0	-1,03	-0,04	0,75	1,00	0	NA	NA	0
NIPBL	Nipped-B homolog (Drosophila)	1,11	0,15	0,46	0,80	0	-1,34	-0,43	0,43	1,00	0	-1,41	-0,50	0
TM4SF1	transmembrane 4 L six family member 1	1,11	0,15	0,36	0,74	0	-1,06	-0,08	0,65	1,00	0	-1,43	-0,51	0
SPRY2	sprouty homolog 2 (Drosophila)	1,11	0,15	0,58	0,86	0	-1,12	-0,17	0,29	1,00	0	NA	NA	0
CTSC	cathepsin C	1,11	0,15	0,60	0,87	0	1,13	0,18	0,23	1,00	0	-1,15	-0,20	0
NF2	neurofibromin 2 (merlin)	1,11	0,15	0,62	0,88	0	-1,05	-0,08	0,72	1,00	0	NA	NA	0
LSM5	LSM5 homolog, U6 small nuclear RNA associated (S. cerevisiae)	1,11	0,15	0,65	0,89	0	-1,05	-0,08	0,78	1,00	0	NA	NA	0
MLEC	malectin	1,11	0,15	0,52	0,83	0	-1,07	-0,10	0,25	1,00	0	NA	NA	0
SPTBN1	spectrin, beta, non-erythrocytic 1	1,11	0,15	0,80	0,94	0	-1,59	-0,66	0,33	1,00	0	-1,64	-0,71	0
MED14	mediator complex subunit 14	1,11	0,15	0,49	0,81	0	1,10	0,14	0,47	1,00	0	-1,06	-0,08	0
NUPL1	nucleoporin like 1	1,11	0,15	0,43	0,78	0	1,09	0,13	0,57	1,00	0	NA	NA	0
NMT1	N-myristoyltransferase 1	1,11	0,15	0,51	0,82	0	-1,27	-0,35	0,14	1,00	0	1,12	0,16	0
PMVK	phosphomevalonate kinase	1,11	0,15	0,31	0,70	0	-1,13	-0,17	0,24	1,00	0	NA	NA	0
PDPN	podoplanin	1,11	0,15	0,31	0,70	0	-1,11	-0,15	0,26	1,00	0	NA	NA	0
PREPL	prolyl endopeptidase-like	1,11	0,15	0,45	0,79	0	-1,11	-0,15	0,42	1,00	0	-1,20	-0,26	0
PPME1	protein phosphatase methylesterase 1	1,11	0,15	0,45	0,79	0	1,02	0,02	0,87	1,00	0	1,14	0,18	0
SMC3	structural maintenance of chromosomes 3	1,11	0,15	0,48	0,81	0	-1,25	-0,32	0,42	1,00	0	NA	NA	0
NA	NA	1,11	0,15	0,36	0,74	0	-1,05	-0,06	0,50	1,00	0	-1,08	-0,11	0
USP11	ubiquitin specific peptidase 11	1,11	0,15	0,48	0,81	0	-1,21	-0,27	0,26	1,00	0	NA	NA	0
NDUFAF4	NADH dehydrogenase (ubiquinone) 1 alpha subcomplex, assembly factor 4	1,11	0,15	0,57	0,86	0	1,19	0,25	0,19	1,00	0	NA	NA	0
ERI3	ERI1 exoribonuclease family member 3	1,11	0,15	0,45	0,79	0	-1,03	-0,04	0,87	1,00	0	NA	NA	0
PPME1	protein phosphatase methylesterase 1	1,11	0,15	0,41	0,77	0	1,05	0,06	0,50	1,00	0	1,17	0,23	0
NUFIP1	nuclear fragile X mental retardation protein interacting protein 1	1,11	0,15	0,46	0,80	0	1,20	0,27	0,11	1,00	0	NA	NA	0
CTSS	cathepsin S	1,11	0,15	0,79	0,94	0	-1,01	-0,01	0,95	1,00	0	1,01	0,02	0
KIAA0226	KIAA0226	1,11	0,15	0,51	0,83	0	1,07	0,10	0,34	1,00	0	NA	NA	0
TRIM23	tripartite motif-containing 23	1,11	0,15	0,52	0,83	0	1,56	0,64	0,08	1,00	0	-1,69	-0,76	0
PABPN1	poly(A) binding protein, nuclear 1	1,11	0,15	0,58	0,86	0	1,04	0,05	0,83	1,00	0	NA	NA	0
FANCI	Fanconi anemia, complementation group I	1,11	0,15	0,50	0,82	0	-1,04	-0,06	0,69	1,00	0	NA	NA	0
SCAP	SREBF chaperone	1,11	0,15	0,40	0,76	0	-1,00	-0,01	0,96	1,00	0	NA	NA	0
NAGK	N-acetylglucosamine kinase	1,11	0,15	0,39	0,76	0	1,43	0,52	0,04	1,00	0	-1,04	-0,05	0
OGFR	opioid growth factor receptor	1,11	0,15	0,56	0,85	0	1,07	0,09	0,73	1,00	0	1,04	0,06	0
C13orf27	chromosome 13 open reading frame 27	1,11	0,15	0,68	0,90	0	-1,00	0,00	0,98	1,00	0	NA	NA	0
PLTP	phospholipid transfer protein	1,11	0,15	0,62	0,88	0	-1,01	-0,01	0,97	1,00	0	NA	NA	0
NA	NA	1,11	0,14	0,64	0,89	0	1,10	0,14	0,57	1,00	0	NA	NA	0
ARL5A	ADP-ribosylation factor-like 5A	1,11	0,14	0,67	0,90	0	-1,09	-0,13	0,12	1,00	0	NA	NA	0
BAG5	BCL2-associated athanogene 5	1,10	0,14	0,45	0,79	0	-1,28	-0,36	0,36	1,00	0	-1,19	-0,24	0
C21orf59	chromosome 21 open reading frame 59	1,10	0,14	0,44	0,78	0	-1,04	-0,06	0,65	1,00	0	NA	NA	0
CLCC1	chloride channel CLIC-like 1	1,10	0,14	0,31	0,70	0	1,01	0,02	0,92	1,00	0	NA	NA	0
MSH6	mutS homolog 6 (E. coli)	1,10	0,14	0,67	0,90	0	-1,19	-0,26	0,35	1,00	0	NA	NA	0
CD40	CD40 molecule, TNF receptor superfamily member 5	1,10	0,14	0,64	0,88	0	-1,10	-0,14	0,35	1,00	0	1,10	0,14	0
TSC22D2	TSC22 domain family, member 2	1,10	0,14	0,64	0,89	0	1,12	0,17	0,38	1,00	0	NA	NA	0
CTAGE9	CTAGE family, member 9	1,10	0,14	0,64	0,89	0	-1,41	-0,50	0,23	1,00	0	NA	NA	0
SLC25A16	solute carrier family 25 (mitochondrial carrier; Graves disease autoantigen), member 16	1,10	0,14	0,54	0,84	0	1,12	0,16	0,50	1,00	0	-1,35	-0,43	0
GRAMD4	GRAM domain containing 4	1,10	0,14	0,55	0,85	0	-1,01	-0,02	0,93	1,00	0	NA	NA	0
CLK3	CDC-like kinase 3	1,10	0,14	0,57	0,85	0	1,14	0,19	0,19	1,00	0	NA	NA	0
RHOF	ras homolog gene family, member F (in filopodia)	1,10	0,14	0,81	0,95	0	1,18	0,24	0,16	1,00	0	NA	NA	0
CDCA4	cell division cycle associated 4	1,10	0,14	0,45	0,79	0	1,14	0,19	0,15	1,00	0	NA	NA	0
TNPO3	transportin 3	1,10	0,14	0,44	0,79	0	-1,04	-0,05	0,63	1,00	0	1,06	0,09	0
C8orf41	chromosome 8 open reading frame 41	1,10	0,14	0,33	0,72	0	-1,14	-0,19	0,43	1,00	0	NA	NA	0
IL6ST	interleukin 6 signal transducer (gp130, oncostatin M receptor)	1,10	0,14	0,66	0,90	0	-1,10	-0,14	0,79	1,00	0	-1,72	-0,79	0
GOLGA4	golgin A4	1,10	0,14	0,62	0,88	0	-1,15	-0,21	0,41	1,00	0	NA	NA	0
ACD	adrenocortical dysplasia homolog (mouse)	1,10	0,14	0,53	0,84	0	-1,08	-0,11	0,44	1,00	0	NA	NA	0
ADAM10	ADAM metallopeptidase domain 10	1,10	0,14	0,44	0,78	0	-1,14	-0,19	0,76	1,00	0	-1,35	-0,43	0
TLN1	talin 1	1,10	0,14	0,67	0,90	0	-1,22	-0,29	0,44	1,00	0	1,71	0,78	0
CELSR2	cadherin, EGF LAG seven-pass G-type receptor 2 (flamingo homolog, Drosophila)	1,10	0,14	0,51	0,83	0	-1,10	-0,13	0,24	1,00	0	NA	NA	0
STAT6	signal transducer and activator of transcription 6, interleukin-4 induced	1,10	0,14	0,64	0,88	0	-1,13	-0,17	0,53	1,00	0	1,18	0,23	0
HP1BP3	heterochromatin protein 1, binding protein 3	1,10	0,14	0,63	0,88	0	1,08	0,10	0,80	1,00	0	-1,09	-0,12	0
NA	NA	1,10	0,14	0,46	0,80	0	-1,19	-0,26	0,34	1,00	0	NA	NA	0
ALDH6A1	aldehyde dehydrogenase 6 family, member A1	1,10	0,14	0,35	0,73	0	-1,22	-0,29	0,09	1,00	0	NA	NA	0
XPO4	exportin 4	1,10	0,14	0,64	0,88	0	-1,14	-0,19	0,50	1,00	0	NA	NA	0
ASNS	asparagine synthetase (glutamine-hydrolyzing)	1,10	0,14	0,62	0,88	0	-1,15	-0,20	0,15	1,00	0	NA	NA	0
TNPO2	transportin 2	1,10	0,14	0,51	0,82	0	-1,15	-0,20	0,13	1,00	0	NA	NA	0
CA12	carbonic anhydrase XII	1,10	0,14	0,41	0,77	0	-1,01	-0,02	0,92	1,00	0	1,15	0,20	0
JAK2	Janus kinase 2	1,10	0,14	0,70	0,91	0	-1,06	-0,08	0,85	1,00	0	-1,45	-0,53	0
APP	amyloid beta (A4) precursor protein	1,10	0,14	0,48	0,81	0	-1,17	-0,22	0,33	1,00	0	-1,28	-0,36	0
NA	NA	1,10	0,14	0,54	0,84	0	-1,12	-0,16	0,29	1,00	0	-1,32	-0,39	0
FAM86B1	family with sequence similarity 86, member B1	1,10	0,13	0,61	0,88	0	-1,27	-0,34	0,23	1,00	0	NA	NA	0
NA	NA	1,10	0,13	0,34	0,72	0	-1,28	-0,36	0,22	1,00	0	NA	NA	0
MAMLD1	mastermind-like domain containing 1	1,10	0,13	0,81	0,95	0	-1,00	-0,01	NA	NA	0	1,10	0,14	0
TULP4	tubby like protein 4	1,10	0,13	0,31	0,70	0	1,03	0,05	0,77	1,00	0	-1,20	-0,27	0
DHFR	dihydrofolate reductase	1,10	0,13	0,61	0,87	0	1,08	0,11	0,54	1,00	0	NA	NA	0
KIAA0090	KIAA0090	1,10	0,13	0,58	0,86	0	-1,20	-0,26	0,41	1,00	0	NA	NA	0
TAPBP	TAP binding protein (tapasin)	1,10	0,13	0,52	0,83	0	-1,25	-0,32	0,09	1,00	0	1,11	0,15	0
WDR45	WD repeat domain 45	1,10	0,13	0,49	0,81	0	-1,17	-0,23	0,18	1,00	0	1,13	0,17	0
MFAP3	microfibrillar-associated protein 3	1,10	0,13	0,45	0,79	0	1,19	0,26	0,07	1,00	0	-1,01	-0,02	0
RNF146	ring finger protein 146	1,10	0,13	0,45	0,79	0	1,22	0,28	0,51	1,00	0	-1,67	-0,73	0
POLD1	polymerase (DNA directed), delta 1, catalytic subunit 125kDa	1,10	0,13	0,42	0,77	0	-1,13	-0,18	0,22	1,00	0	NA	NA	0
GOLGA4	golgin A4	1,10	0,13	0,37	0,75	0	1,06	0,09	0,36	1,00	0	-1,15	-0,20	0
PLIN3	perilipin 3	1,10	0,13	0,66	0,89	0	-1,08	-0,11	0,66	1,00	0	NA	NA	0
ZFAND3	zinc finger, AN1-type domain 3	1,10	0,13	0,63	0,88	0	1,30	0,37	0,05	1,00	0	NA	NA	0
NUCB1	nucleobindin 1	1,10	0,13	0,55	0,85	0	-1,08	-0,11	0,64	1,00	0	1,52	0,60	0
ANPEP	alanyl (membrane) aminopeptidase	1,10	0,13	0,47	0,80	0	-1,01	-0,02	NA	NA	0	1,07	0,09	0
SIAH1	seven in absentia homolog 1 (Drosophila)	1,10	0,13	0,60	0,87	0	-1,03	-0,04	0,88	1,00	0	-1,32	-0,40	0
PAFAH1B1	platelet-activating factor acetylhydrolase 1b, regulatory subunit 1 (45kDa)	1,10	0,13	0,39	0,75	0	1,10	0,14	0,17	1,00	0	-1,09	-0,12	0
HSD11B1	hydroxysteroid (11-beta) dehydrogenase 1	1,10	0,13	0,69	0,91	0	-1,23	-0,29	0,38	1,00	0	NA	NA	0
BUB3	budding uninhibited by benzimidazoles 3 homolog (yeast)	1,10	0,13	0,40	0,76	0	-1,01	-0,02	0,95	1,00	0	NA	NA	0
LGALS8	lectin, galactoside-binding, soluble, 8	1,10	0,13	0,36	0,73	0	1,03	0,04	0,84	1,00	0	NA	NA	0
MCM4	minichromosome maintenance complex component 4	1,10	0,13	0,52	0,83	0	-1,05	-0,07	0,62	1,00	0	NA	NA	0
PDE4DIP	phosphodiesterase 4D interacting protein	1,10	0,13	0,74	0,92	0	1,11	0,15	0,52	1,00	0	-1,22	-0,28	0
NCAPD2	non-SMC condensin I complex, subunit D2	1,09	0,13	0,61	0,87	0	-1,28	-0,35	0,23	1,00	0	NA	NA	0
C10orf2	chromosome 10 open reading frame 2	1,09	0,13	0,57	0,85	0	-1,03	-0,04	0,85	1,00	0	NA	NA	0
IL1RAP	interleukin 1 receptor accessory protein	1,09	0,13	0,46	0,80	0	-1,11	-0,15	0,23	1,00	0	NA	NA	0
BCL11A	B-cell CLL/lymphoma 11A (zinc finger protein)	1,09	0,13	0,54	0,84	0	-1,02	-0,02	0,95	1,00	0	NA	NA	0
GRB14	growth factor receptor-bound protein 14	1,09	0,13	0,74	0,92	0	-1,23	-0,30	0,25	1,00	0	NA	NA	0
OBSL1	obscurin-like 1	1,09	0,13	0,53	0,84	0	-1,10	-0,14	0,44	1,00	0	NA	NA	0
SLC3A2	solute carrier family 3 (activators of dibasic and neutral amino acid transport), member 2	1,09	0,13	0,53	0,84	0	-1,00	0,00	1,00	1,00	0	NA	NA	0
PSMA4	proteasome (prosome, macropain) subunit, alpha type, 4	1,09	0,13	0,43	0,78	0	1,01	0,02	0,91	1,00	0	-1,10	-0,14	0
JOSD1	Josephin domain containing 1	1,09	0,13	0,58	0,86	0	-1,01	-0,02	0,88	1,00	0	NA	NA	0
CD74	CD74 molecule, major histocompatibility complex, class II invariant chain	1,09	0,13	0,73	0,92	0	-1,00	0,00	0,98	1,00	0	1,01	0,01	0
CALD1	caldesmon 1	1,09	0,13	0,61	0,87	0	-1,41	-0,49	0,41	1,00	0	-1,30	-0,38	0
MRPS2	mitochondrial ribosomal protein S2	1,09	0,13	0,55	0,85	0	-1,05	-0,08	0,62	1,00	0	NA	NA	0
DHTKD1	dehydrogenase E1 and transketolase domain containing 1	1,09	0,13	0,64	0,88	0	-1,06	-0,08	0,65	1,00	0	-1,19	-0,25	0
KARS	lysyl-tRNA synthetase	1,09	0,13	0,54	0,84	0	-1,01	-0,01	0,92	1,00	0	-1,15	-0,19	0
GLRX5	glutaredoxin 5	1,09	0,13	0,34	0,72	0	1,02	0,03	0,79	1,00	0	NA	NA	0
SNCA	synuclein, alpha (non A4 component of amyloid precursor)	1,09	0,13	0,47	0,80	0	-1,02	-0,03	0,87	1,00	0	-1,05	-0,08	0
PCDH7	protocadherin 7	1,09	0,13	0,83	0,95	0	-1,13	-0,18	0,19	1,00	0	NA	NA	0
SCLY	selenocysteine lyase	1,09	0,13	0,51	0,82	0	-1,22	-0,29	0,33	1,00	0	NA	NA	0
PTPN11	protein tyrosine phosphatase, non-receptor type 11	1,09	0,13	0,48	0,81	0	1,01	0,02	0,88	1,00	0	-1,02	-0,03	0
NUFIP1	nuclear fragile X mental retardation protein interacting protein 1	1,09	0,13	0,59	0,86	0	1,06	0,08	0,70	1,00	0	NA	NA	0
ZWINT	ZW10 interactor	1,09	0,12	0,64	0,89	0	1,09	0,12	0,37	1,00	0	NA	NA	0
ANKLE2	ankyrin repeat and LEM domain containing 2	1,09	0,12	0,73	0,92	0	-1,09	-0,13	0,47	1,00	0	NA	NA	0
INPP1	inositol polyphosphate-1-phosphatase	1,09	0,12	0,55	0,84	0	1,08	0,11	0,48	1,00	0	NA	NA	0
BLZF1	basic leucine zipper nuclear factor 1	1,09	0,12	0,43	0,78	0	1,01	0,01	0,96	1,00	0	NA	NA	0
BCAR3	breast cancer anti-estrogen resistance 3	1,09	0,12	0,81	0,95	0	-1,11	-0,16	0,13	1,00	0	-1,14	-0,18	0
GART	phosphoribosylglycinamide formyltransferase, phosphoribosylglycinamide synthetase, phosphoribosylaminoimidazole synthetase	1,09	0,12	0,57	0,86	0	-1,04	-0,05	0,75	1,00	0	NA	NA	0
IFI16	interferon, gamma-inducible protein 16	1,09	0,12	0,51	0,82	0	1,00	0,00	1,00	1,00	0	-1,64	-0,72	0
SSRP1	structure specific recognition protein 1	1,09	0,12	0,35	0,73	0	-1,12	-0,16	0,26	1,00	0	NA	NA	0

ARHGEF2	Rho/Rac guanine nucleotide exchange factor (GEF) 2	1,09	0,12	0,48	0,81	0	-1,08	-0,11	0,70	1,00	0	NA	NA	0
RELA	v-rel reticuloendotheliosis viral oncogene homolog A (avian)	1,09	0,12	0,54	0,84	0	-1,64	-0,71	0,23	1,00	0	NA	NA	0
ZBED4	zinc finger, BED-type containing 4	1,09	0,12	0,56	0,85	0	1,07	0,09	0,81	1,00	0	-1,28	-0,36	0
POLE	polymerase (DNA directed), epsilon	1,09	0,12	0,59	0,87	0	-1,04	-0,06	0,75	1,00	0	NA	NA	0
DHFR	dihydrofolate reductase	1,09	0,12	0,47	0,80	0	-1,09	-0,12	0,66	1,00	0	NA	NA	0
TMEM194A	transmembrane protein 194A	1,09	0,12	0,50	0,82	0	1,06	0,09	0,79	1,00	0	-1,27	-0,34	0
KIAA0101	KIAA0101	1,09	0,12	0,40	0,76	0	-1,04	-0,06	0,77	1,00	0	-1,06	-0,08	0
RANBP2	RAN binding protein 2	1,09	0,12	0,33	0,71	0	-1,02	-0,03	0,76	1,00	0	NA	NA	0
PSKH1	protein serine kinase H1	1,09	0,12	0,45	0,79	0	-1,01	-0,02	0,89	1,00	0	1,45	0,54	0
PFAS	phosphoribosylformylglycinamidine synthase	1,09	0,12	0,57	0,86	0	1,02	0,03	0,89	1,00	0	NA	NA	0
GLS	glutaminase	1,09	0,12	0,44	0,79	0	1,22	0,28	0,12	1,00	0	-1,16	-0,22	0
PALM2-AKAP2	PALM2-AKAP2 readthrough	1,09	0,12	0,43	0,78	0	-1,00	-0,01	0,98	1,00	0	NA	NA	0
KTELC1	KTEL (Lys-Tyr-Glu-Leu) containing 1	1,09	0,12	0,48	0,81	0	-1,19	-0,25	0,46	1,00	0	NA	NA	0
TIMM8A	translocase of inner mitochondrial membrane 8 homolog A (yeast)	1,09	0,12	0,78	0,94	0	1,04	0,06	0,79	1,00	0	NA	NA	0
CNOT1	CCR4-NOT transcription complex, subunit 1	1,09	0,12	0,49	0,81	0	-1,18	-0,24	0,23	1,00	0	1,46	0,55	0
DDX23	DEAD (Asp-Glu-Ala-Asp) box polypeptide 23	1,09	0,12	0,65	0,89	0	1,12	0,17	0,18	1,00	0	1,07	0,10	0
FAM168B	family with sequence similarity 168, member B	1,09	0,12	0,46	0,79	0	-1,03	-0,05	0,74	1,00	0	-1,12	-0,17	0
FRAT2	frequently rearranged in advanced T-cell lymphomas 2	1,09	0,12	0,53	0,84	0	-1,06	-0,08	0,71	1,00	0	NA	NA	0
ZNF131	zinc finger protein 131	1,09	0,12	0,57	0,86	0	1,15	0,20	0,19	1,00	0	NA	NA	0
CDC40	cell division cycle 40 homolog (S. cerevisiae)	1,09	0,12	0,54	0,84	0	-1,06	-0,08	0,74	1,00	0	-1,35	-0,44	0
TPM4	tropomyosin 4	1,09	0,12	0,39	0,76	0	-1,02	-0,02	0,90	1,00	0	NA	NA	0
GORASP2	golgi reassembly stacking protein 2, 55kDa	1,09	0,12	0,61	0,87	0	-1,05	-0,07	0,57	1,00	0	-1,19	-0,26	0
RRP12	ribosomal RNA processing 12 homolog (S. cerevisiae)	1,09	0,12	0,40	0,76	0	-1,04	-0,06	0,73	1,00	0	NA	NA	0
C15orf39	chromosome 15 open reading frame 39	1,08	0,12	0,79	0,94	0	1,20	0,26	0,20	1,00	0	NA	NA	0
ZNF516	zinc finger protein 516	1,08	0,12	0,57	0,86	0	-1,05	-0,07	0,77	1,00	0	1,06	0,08	0
TYRO3	TYRO3 protein tyrosine kinase	1,08	0,12	0,52	0,83	0	1,03	0,04	0,90	1,00	0	-1,02	-0,03	0
COL16A1	collagen, type XVI, alpha 1	1,08	0,12	0,45	0,79	0	-1,04	-0,05	0,70	1,00	0	1,49	0,57	0
ZHX3	zinc fingers and homeoboxes 3	1,08	0,12	0,55	0,85	0	-1,06	-0,08	0,64	1,00	0	-1,05	-0,07	0
KPNA1	karyopherin alpha 1 (importin alpha 5)	1,08	0,12	0,43	0,78	0	-1,04	-0,06	0,49	1,00	0	NA	NA	0
COX11	COX11 cytochrome c oxidase assembly homolog (yeast)	1,08	0,12	0,63	0,88	0	1,09	0,12	0,30	1,00	0	-1,11	-0,15	0
CKS1B	CDC28 protein kinase regulatory subunit 1B	1,08	0,12	0,62	0,88	0	1,03	0,04	0,81	1,00	0	NA	NA	0
MFNG	MFNG O-fucosylpeptide 3-beta-N-acetylglucosaminyltransferase	1,08	0,12	0,80	0,94	0	-1,12	-0,17	0,65	1,00	0	NA	NA	0
RANBP2	RAN binding protein 2	1,08	0,12	0,46	0,80	0	1,08	0,11	0,57	1,00	0	NA	NA	0
RELB	v-rel reticuloendotheliosis viral oncogene homolog B	1,08	0,12	0,52	0,83	0	-1,68	-0,74	0,00	0,33	0	NA	NA	0
C16orf88	chromosome 16 open reading frame 88	1,08	0,12	0,70	0,91	0	-1,15	-0,20	0,37	1,00	0	NA	NA	0
SLC16A3	solute carrier family 16, member 3 (monocarboxylic acid transporter 4)	1,08	0,12	0,66	0,89	0	1,10	0,13	0,68	1,00	0	NA	NA	0
SRSF6	serine/arginine-rich splicing factor 6	1,08	0,12	0,43	0,78	0	1,11	0,15	0,15	1,00	0	NA	NA	0
RNF24	ring finger protein 24	1,08	0,11	0,66	0,89	0	-1,11	-0,14	0,54	1,00	0	1,87	0,90	0
C19orf28	chromosome 19 open reading frame 28	1,08	0,11	0,59	0,87	0	-1,01	-0,02	0,82	1,00	0	-1,06	-0,08	0
LRP12	low density lipoprotein receptor-related protein 12	1,08	0,11	0,50	0,82	0	1,20	0,26	0,05	1,00	0	NA	NA	0
ABCE1	ATP-binding cassette, sub-family E (OABP), member 1	1,08	0,11	0,55	0,84	0	1,03	0,05	0,61	1,00	0	NA	NA	0
DCAF8	DDB1 and CUL4 associated factor 8	1,08	0,11	0,41	0,77	0	-1,09	-0,13	0,72	1,00	0	NA	NA	0
RNGTT	RNA guanylyltransferase and 5'-phosphatase	1,08	0,11	0,70	0,91	0	-1,97	-0,98	0,01	0,67	0	NA	NA	0
SLC31A2	solute carrier family 31 (copper transporters), member 2	1,08	0,11	0,62	0,88	0	1,10	0,14	0,38	1,00	0	NA	NA	0
JAG1	jagged 1	1,08	0,11	0,49	0,81	0	1,14	0,19	0,10	1,00	0	1,18	0,24	0
STK10	serine/threonine kinase 10	1,08	0,11	0,60	0,87	0	-1,12	-0,16	0,29	1,00	0	1,23	0,30	0
CCDC137	coiled-coil domain containing 137	1,08	0,11	0,54	0,84	0	NA	NA	NA	NA	0	NA	NA	0
CELSR2	cadherin, EGF LAG seven-pass G-type receptor 2 (flamingo homolog, Drosophila)	1,08	0,11	0,61	0,88	0	-1,10	-0,14	0,26	1,00	0	NA	NA	0
GPSM2	G-protein signaling modulator 2	1,08	0,11	0,57	0,85	0	-1,02	-0,03	0,73	1,00	0	NA	NA	0
HK2	hexokinase 2	1,08	0,11	0,45	0,79	0	1,09	0,13	0,30	1,00	0	1,02	0,02	0
CENPA	centromere protein A	1,08	0,11	0,76	0,93	0	1,01	0,01	0,91	1,00	0	NA	NA	0
DOPEY1	dopey family member 1	1,08	0,11	0,41	0,77	0	1,10	0,14	0,31	1,00	0	-1,15	-0,21	0
TTI1	Tel2 interacting protein 1 homolog (S. pombe)	1,08	0,11	0,57	0,86	0	-1,03	-0,04	0,77	1,00	0	NA	NA	0
PSD4	pleckstrin and Sec7 domain containing 4	1,08	0,11	0,68	0,90	0	1,13	0,17	0,30	1,00	0	NA	NA	0
ZNF114	zinc finger protein 114	1,08	0,11	0,81	0,95	0	NA	NA	NA	NA	0	NA	NA	0
IVNS1ABP	influenza virus NS1A binding protein	1,08	0,11	0,59	0,87	0	-1,00	0,00	0,98	1,00	0	NA	NA	0
LMNA	lamin A/C	1,08	0,11	0,45	0,79	0	-1,30	-0,38	0,05	1,00	0	NA	NA	0
SEPHS1	selenophosphate synthetase 1	1,08	0,11	0,59	0,87	0	1,05	0,06	0,57	1,00	0	NA	NA	0
RIPK1	receptor (TNFRSF)-interacting serine-threonine kinase 1	1,08	0,11	0,69	0,90	0	-1,02	-0,02	0,92	1,00	0	1,06	0,09	0
DDX11	DEAD/H (Asp-Glu-Ala-Asp/His) box polypeptide 11	1,08	0,11	0,58	0,86	0	-1,16	-0,22	0,17	1,00	0	NA	NA	0
TGIF2	TGFB-induced factor homeobox 2	1,08	0,11	0,75	0,93	0	-1,17	-0,23	0,51	1,00	0	NA	NA	0
TWF1	twinfilin, actin-binding protein, homolog 1 (Drosophila)	1,08	0,11	0,53	0,83	0	1,43	0,51	0,55	1,00	0	-1,12	-0,17	0
C12orf10	chromosome 12 open reading frame 10	1,08	0,11	0,60	0,87	0	-1,17	-0,23	0,09	1,00	0	NA	NA	0
IRF6	interferon regulatory factor 6	1,08	0,11	0,62	0,88	0	1,02	0,04	0,76	1,00	0	NA	NA	0
MTFR1	mitochondrial fission regulator 1	1,08	0,11	0,46	0,80	0	1,09	0,13	0,58	1,00	0	-1,23	-0,30	0
SLC20A1	solute carrier family 20 (phosphate transporter), member 1	1,08	0,11	0,39	0,76	0	-1,07	-0,10	0,40	1,00	0	NA	NA	0
SIRT6	sirtuin 6	1,08	0,11	0,58	0,86	0	1,06	0,08	0,42	1,00	0	1,09	0,12	0
XPOT	exportin, tRNA (nuclear export receptor for tRNAs)	1,08	0,11	0,51	0,82	0	-1,01	-0,01	0,92	1,00	0	NA	NA	0
SRPRB	signal recognition particle receptor, B subunit	1,08	0,11	0,60	0,87	0	1,24	0,31	0,06	1,00	0	NA	NA	0
DCP1A	DCP1 decapping enzyme homolog A (S. cerevisiae)	1,08	0,11	0,68	0,90	0	1,26	0,33	0,15	1,00	0	-1,03	-0,05	0
MOSPD1	motile sperm domain containing 1	1,08	0,11	0,67	0,90	0	1,11	0,15	0,18	1,00	0	-1,12	-0,17	0
RRM1	ribonucleotide reductase M1	1,08	0,11	0,48	0,81	0	1,01	0,01	0,91	1,00	0	NA	NA	0
MRPL2	mitochondrial ribosomal protein L2	1,08	0,11	0,49	0,81	0	-1,01	-0,02	0,91	1,00	0	NA	NA	0
SEPHS1	selenophosphate synthetase 1	1,08	0,11	0,54	0,84	0	1,22	0,29	0,07	1,00	0	-1,10	-0,14	0
AFG3L2	AFG3 ATPase family gene 3-like 2 (S. cerevisiae)	1,08	0,11	0,51	0,82	0	1,33	0,41	0,24	1,00	0	NA	NA	0
STX6	syntaxin 6	1,08	0,11	0,57	0,86	0	1,05	0,07	0,78	1,00	0	-1,43	-0,52	0
CEBPD	CCAAT/enhancer binding protein (C/EBP), delta	1,08	0,11	0,69	0,90	0	-1,00	-0,01	0,95	1,00	0	-1,27	-0,34	0
NEK2	NIMA (never in mitosis gene a)-related kinase 2	1,08	0,11	0,69	0,90	0	-1,04	-0,05	0,71	1,00	0	NA	NA	0
NAP1L1	nucleosome assembly protein 1-like 1	1,08	0,11	0,63	0,88	0	-1,03	-0,04	0,71	1,00	0	NA	NA	0
CENPF	centromere protein F, 350/400kDa (mitosin)	1,08	0,11	0,66	0,89	0	-1,11	-0,16	0,68	1,00	0	NA	NA	0
EXOSC2	exosome component 2	1,08	0,11	0,63	0,88	0	-1,02	-0,03	0,88	1,00	0	NA	NA	0
PNN	pinin, desmosome associated protein	1,08	0,11	0,71	0,91	0	1,09	0,13	0,44	1,00	0	NA	NA	0
FYN	FYN oncogene related to SRC, FGR, YES	1,08	0,11	0,68	0,90	0	-1,13	-0,17	0,43	1,00	0	1,07	0,10	0
APBB2	amyloid beta (A4) precursor protein-binding, family B, member 2	1,08	0,10	0,54	0,84	0	-1,59	-0,67	0,12	1,00	0	NA	NA	0
GAS2L1	growth arrest-specific 2 like 1	1,08	0,10	0,45	0,79	0	1,01	0,01	NA	NA	0	1,08	0,11	0
MAP2	microtubule-associated protein 2	1,07	0,10	0,78	0,94	0	-1,40	-0,49	0,27	1,00	0	1,01	0,01	0
CLIC4	chloride intracellular channel 4	1,07	0,10	0,51	0,83	0	-1,21	-0,27	0,72	1,00	0	-1,23	-0,31	0
GPS1	G protein pathway suppressor 1	1,07	0,10	0,52	0,83	0	1,27	0,35	0,13	1,00	0	NA	NA	0
FBXO5	F-box protein 5	1,07	0,10	0,61	0,87	0	1,01	0,02	0,86	1,00	0	NA	NA	0
H2AFY	H2A histone family, member Y	1,07	0,10	0,69	0,90	0	-1,69	-0,76	0,30	1,00	0	-1,20	-0,28	0
BTN2A2	butyrophilin, subfamily 2, member A2	1,07	0,10	0,54	0,84	0	1,12	0,17	0,47	1,00	0	NA	NA	0
TMEM161A	transmembrane protein 161A	1,07	0,10	0,51	0,83	0	-1,02	-0,02	0,88	1,00	0	NA	NA	0
PPAT	phosphoribosyl pyrophosphate amidotransferase	1,07	0,10	0,56	0,85	0	1,13	0,17	0,19	1,00	0	NA	NA	0
PCYT2	phosphate cytidylyltransferase 2, ethanolamine	1,07	0,10	0,71	0,91	0	-1,07	-0,09	0,59	1,00	0	NA	NA	0
ACYP1	acylphosphatase 1, erythrocyte (common) type	1,07	0,10	0,69	0,91	0	-1,03	-0,05	0,79	1,00	0	NA	NA	0
DPH2	DPH2 homolog (S. cerevisiae)	1,07	0,10	0,59	0,87	0	-1,01	-0,02	0,94	1,00	0	NA	NA	0
NA	NA	1,07	0,10	0,68	0,90	0	NA	NA	NA	NA	0	NA	NA	0
EEF1D	eukaryotic translation elongation factor 1 delta (guanine nucleotide exchange protein)	1,07	0,10	0,52	0,83	0	-1,03	-0,04	0,93	1,00	0	NA	NA	0
ETV7	ets variant 7	1,07	0,10	0,83	0,96	0	-1,01	-0,01	0,94	1,00	0	-1,01	-0,01	0
TESK2	testis-specific kinase 2	1,07	0,10	0,47	0,81	0	-1,01	-0,01	0,98	1,00	0	-1,01	-0,01	0
ANKRD13D	ankyrin repeat domain 13 family, member D	1,07	0,10	0,44	0,78	0	NA	NA	NA	NA	0	NA	NA	0
PDE4B	phosphodiesterase 4B, cAMP-specific	1,07	0,10	0,79	0,94	0	-1,01	-0,01	NA	NA	0	-1,04	-0,06	0
FERMT2	fermitin family member 2	1,07	0,10	0,65	0,89	0	-1,12	-0,17	0,36	1,00	0	NA	NA	0
PSMA6	proteasome (prosome, macropain) subunit, alpha type, 6	1,07	0,10	0,49	0,81	0	1,17	0,23	0,07	1,00	0	NA	NA	0
NA	NA	1,07	0,10	0,64	0,89	0	-1,09	-0,13	0,54	1,00	0	-1,15	-0,20	0
PRPF19	PRP19/PSO4 pre-mRNA processing factor 19 homolog (S. cerevisiae)	1,07	0,10	0,56	0,85	0	1,01	0,02	0,93	1,00	0	NA	NA	0
PLCL2	phospholipase C-like 2	1,07	0,10	0,78	0,94	0	-1,02	-0,03	NA	NA	0	1,20	0,26	0
CELF1	CUGBP, Elav-like family member 1	1,07	0,10	0,71	0,91	0	1,03	0,04	0,88	1,00	0	-1,49	-0,58	0
RQCD1	RCD1 required for cell differentiation1 homolog (S. pombe)	1,07	0,10	0,47	0,81	0	-1,11	-0,15	0,50	1,00	0	NA	NA	0
PIGN	phosphatidylinositol glycan anchor biosynthesis, class N	1,07	0,10	0,63	0,88	0	-1,22	-0,29	0,23	1,00	0	-1,32	-0,40	0
IFT122	intraflagellar transport 122 homolog (Chlamydomonas)	1,07	0,10	0,77	0,93	0	-1,06	-0,08	0,44	1,00	0	-1,03	-0,04	0
PTCD3	Pentatricopeptide repeat domain 3	1,07	0,10	0,70	0,91	0	1,01	0,01	0,94	1,00	0	NA	NA	0
C22orf29	chromosome 22 open reading frame 29	1,07	0,10	0,62	0,88	0	-1,27	-0,35	0,09	1,00	0	NA	NA	0
TM4SF1	transmembrane 4 L six family member 1	1,07	0,10	0,56	0,85	0	-1,09	-0,12	0,38	1,00	0	-1,28	-0,35	0
NA	NA	1,07	0,10	0,61	0,87	0	-1,08	-0,11	0,43	1,00	0	NA	NA	0
CSTF1	cleavage stimulation factor, 3' pre-RNA, subunit 1, 50kDa	1,07	0,10	0,67	0,90	0	1,25	0,32	0,24	1,00	0	NA	NA	0
NAB1	NGFI-A binding protein 1 (EGR1 binding protein 1)	1,07	0,10	0,79	0,94	0	-1,18	-0,24	0,57	1,00	0	1,20	0,26	0
LSR	lipolysis stimulated lipoprotein receptor	1,07	0,10	0,77	0,93	0	-1,27	-0,34	0,14	1,00	0	NA	NA	0
DHRS11	dehydrogenase/reductase (SDR family) member 11	1,07	0,10	0,76	0,93	0	-1,15	-0,20	0,54	1,00	0	NA	NA	0
CDCA8	cell division cycle associated 8	1,07	0,10	0,72	0,92	0	1,02	0,03	0,82	1,00	0	NA	NA	0
THG1L	tRNA-histidine guanylyltransferase 1-like (S. cerevisiae)	1,07	0,10	0,63	0,88	0	-1,21	-0,28	0,33	1,00	0	NA	NA	0
PLOD2	procollagen-lysine, 2-oxoglutarate 5-dioxygenase 2	1,07	0,10	0,80	0,95	0	1,24	0,31	0,67	1,00	0	NA	NA	0
STOM	stomatin	1,07	0,10	0,52	0,83	0	1,05	0,07	0,72	1,00	0	-1,05	-0,07	0
MPHOSPH9	M-phase phosphoprotein 9	1,07	0,10	0,60	0,87	0	-1,21	-0,27	0,17	1,00	0	-1,28	-0,37	0
LY6G6C	lymphocyte antigen 6 complex, locus G6C	1,07	0,10	0,65	0,89	0	-1,61	-0,68	0,09	1,00	0	NA	NA	0
AQP3	aquaporin 3 (Gill blood group)	1,07	0,10	0,85	0,96	0	-1,15	-0,20	0,59	1,00	0	NA	NA	0
COQ10B	coenzyme Q10 homolog B (S. cerevisiae)	1,07	0,10	0,68	0,90	0	1,16	0,22	0,16	1,00	0	NA	NA	0
SIN3B	SIN3 homolog B, transcription regulator (yeast)	1,07	0,09	0,69	0,90	0	1,04	0,05	0,76	1,00	0	NA	NA	0
NHP2L1	NHP2 non-histone chromosome protein 2-like 1 (S. cerevisiae)	1,07	0,09	0,54	0,84	0	-1,12	-0,16	0,30	1,00	0	NA	NA	0
WIPF2	WAS/WASL interacting protein family, member 2	1,07	0,09	0,70	0,91	0	1,13	0,18	0,44	1,00	0	NA	NA	0
RYBP	RING1 and YY1 binding protein	1,07	0,09	0,68	0,90	0	-1,04	-0,05	0,76	1,00	0	NA	NA	0
POLR3K	polymerase (RNA) III (DNA directed) polypeptide K, 12.3 kDa	1,07	0,09	0,71	0,91	0	1,25	0,32	0,29	1,00	0	NA	NA	0
NA	NA	1,07	0,09	0,52	0,83	0	-1,10	-0,13	0,29	1,00	0	NA	NA	0
PSMA5	proteasome (prosome, macropain) subunit, alpha type, 5	1,07	0,09	0,69	0,90	0	1,13	0,18	0,15	1,00	0	NA	NA	0
CLINT1	clathrin interactor 1	1,07	0,09	0,71	0,91	0	-1,02	-0,03	0,97	1,00	0	-1,27	-0,35	0
VPS16	vacuolar protein sorting 16 homolog (S. cerevisiae)	1,07	0,09	0,66	0,89	0	-1,12	-0,16	0,28	1,00	0	NA	NA	0
CSE1L	CSE1 chromosome segregation 1-like (yeast)	1,07	0,09	0,63	0,88	0	-1,02	-0,03	0,83	1,00	0	NA	NA	0

GEMIN4	gem (nuclear organelle) associated protein 4	1,07	0,09	0,65	0,89	0	1,09	0,12	0,42	1,00	0	NA	NA	0
STOM	stomatin	1,07	0,09	0,55	0,84	0	-1,06	-0,08	0,49	1,00	0	-1,04	-0,06	0
PPP1CB	protein phosphatase 1, catalytic subunit, beta isozyme	1,07	0,09	0,66	0,89	0	1,06	0,08	0,56	1,00	0	-1,02	-0,03	0
PSEN1	presenilin 1	1,07	0,09	0,54	0,84	0	1,02	0,03	0,79	1,00	0	NA	NA	0
SIAH1	seven in absentia homolog 1 (Drosophila)	1,07	0,09	0,61	0,88	0	1,12	0,16	0,15	1,00	0	-1,19	-0,26	0
SF3B1	splicing factor 3b, subunit 1, 155kDa	1,07	0,09	0,56	0,85	0	-1,11	-0,15	0,83	1,00	0	-1,20	-0,26	0
KDM4B	lysine (K)-specific demethylase 4B	1,07	0,09	0,80	0,94	0	1,14	0,18	0,36	1,00	0	NA	NA	0
UBXN7	UBX domain protein 7	1,07	0,09	0,64	0,89	0	-1,14	-0,19	0,38	1,00	0	-1,35	-0,43	0
TXNDC5	thioredoxin domain containing 5 (endoplasmic reticulum)	1,07	0,09	0,56	0,85	0	-1,07	-0,10	0,47	1,00	0	NA	NA	0
SETD2	SET domain containing 2	1,07	0,09	0,73	0,92	0	-1,01	-0,02	0,91	1,00	0	0,00	0,00	0
PDE4B	phosphodiesterase 4B, cAMP-specific	1,07	0,09	0,85	0,96	0	-1,01	-0,01	NA	NA	0	-1,20	-0,26	0
CHEK2	CHK2 checkpoint homolog (S. pombe)	1,07	0,09	0,65	0,89	0	1,04	0,06	0,79	1,00	0	NA	NA	0
RAB31	RAB31, member RAS oncogene family	1,06	0,09	0,51	0,83	0	1,23	0,29	0,03	0,99	0	1,05	0,08	0
P2RX4	purinergic receptor P2X, ligand-gated ion channel, 4	1,06	0,09	0,67	0,90	0	1,05	0,07	0,90	1,00	0	NA	NA	0
TNFRSF25	tumor necrosis factor receptor superfamily, member 25	1,06	0,09	0,81	0,95	0	-1,01	-0,02	0,94	1,00	0	NA	NA	0
DLG5	discs, large homolog 5 (Drosophila)	1,06	0,09	0,49	0,81	0	1,00	0,01	0,97	1,00	0	NA	NA	0
RCN2	reticulocalbin 2, EF-hand calcium binding domain	1,06	0,09	0,72	0,92	0	1,10	0,14	0,55	1,00	0	-1,12	-0,16	0
OGFR	opioid growth factor receptor	1,06	0,09	0,49	0,81	0	-1,02	-0,02	0,93	1,00	0	NA	NA	0
ZFYVE26	zinc finger, FYVE domain containing 26	1,06	0,09	0,61	0,87	0	1,03	0,05	0,82	1,00	0	-1,10	-0,14	0
RFC4	replication factor C (activator 1) 4, 37kDa	1,06	0,09	0,69	0,91	0	-1,03	-0,04	0,82	1,00	0	NA	NA	0
CDC25B	cell division cycle 25 homolog B (S. pombe)	1,06	0,09	0,60	0,87	0	-1,09	-0,13	0,53	1,00	0	NA	NA	0
WEE1	WEE1 homolog (S. pombe)	1,06	0,09	0,78	0,94	0	1,10	0,14	0,70	1,00	0	-1,12	-0,17	0
NIT1	nitrilase 1	1,06	0,09	0,55	0,84	0	1,03	0,04	0,67	1,00	0	NA	NA	0
NA	NA	1,06	0,09	0,65	0,89	0	-1,01	-0,02	0,92	1,00	0	NA	NA	0
SPINK5	serine peptidase inhibitor, Kazal type 5	1,06	0,09	0,52	0,83	0	1,12	0,16	0,62	1,00	0	NA	NA	0
FGF1	fibroblast growth factor 1 (acidic)	1,06	0,09	0,85	0,96	0	-1,00	-0,01	NA	NA	0	-1,33	-0,42	0
LMF2	lipase maturation factor 2	1,06	0,09	0,78	0,94	0	1,05	0,07	NA	NA	0	1,37	0,45	0
FKBP1B	FK506 binding protein 1B, 12.6 kDa	1,06	0,09	0,72	0,91	0	-1,22	-0,29	0,12	1,00	0	-1,10	-0,14	0
NA	NA	1,06	0,09	0,82	0,95	0	-1,93	-0,95	0,02	0,86	0	NA	NA	0
DDX23	DEAD (Asp-Glu-Ala-Asp) box polypeptide 23	1,06	0,08	0,74	0,92	0	1,14	0,19	0,11	1,00	0	1,12	0,17	0
LRPPRC	leucine-rich PPR-motif containing	1,06	0,08	0,58	0,86	0	-1,05	-0,07	0,57	1,00	0	NA	NA	0
GEMIN4	gem (nuclear organelle) associated protein 4	1,06	0,08	0,76	0,93	0	1,01	0,01	0,92	1,00	0	NA	NA	0
EXOC7	exocyst complex component 7	1,06	0,08	0,79	0,94	0	-1,10	-0,14	0,61	1,00	0	-1,06	-0,09	0
WDR55	WD repeat domain 55	1,06	0,08	0,57	0,85	0	1,07	0,10	0,51	1,00	0	NA	NA	0
PUS7	pseudouridylate synthase 7 homolog (S. cerevisiae)	1,06	0,08	0,60	0,87	0	1,08	0,11	0,42	1,00	0	NA	NA	0
THBS1	thrombospondin 1	1,06	0,08	0,67	0,90	0	1,05	0,07	0,84	1,00	0	-1,12	-0,17	0
APLP2	amyloid beta (A4) precursor-like protein 2	1,06	0,08	0,76	0,93	0	-1,08	-0,11	0,66	1,00	0	-1,28	-0,37	0
BCS1L	BCS1-like (S. cerevisiae)	1,06	0,08	0,62	0,88	0	1,00	0,00	0,97	1,00	0	NA	NA	0
EDEM2	ER degradation enhancer, mannosidase alpha-like 2	1,06	0,08	0,71	0,91	0	-1,09	-0,12	0,47	1,00	0	-1,14	-0,18	0
C16orf80	chromosome 16 open reading frame 80	1,06	0,08	0,63	0,88	0	1,06	0,09	0,49	1,00	0	NA	NA	0
UMPS	uridine monophosphate synthetase	1,06	0,08	0,59	0,87	0	1,08	0,11	0,48	1,00	0	NA	NA	0
TIPIN	TIMELESS interacting protein	1,06	0,08	0,79	0,94	0	1,02	0,03	0,90	1,00	0	NA	NA	0
KLHDC10	kelch domain containing 10	1,06	0,08	0,70	0,91	0	1,82	0,87	0,06	1,00	0	-1,41	-0,49	0
FAM69A	family with sequence similarity 69, member A	1,06	0,08	0,66	0,89	0	1,39	0,47	0,02	0,88	0	-1,11	-0,15	0
SOS1	son of sevenless homolog 1 (Drosophila)	1,06	0,08	0,71	0,91	0	-1,05	-0,07	0,71	1,00	0	NA	NA	0
PRRX1	paired related homeobox 1	1,06	0,08	0,79	0,94	0	-1,01	-0,02	NA	NA	0	1,14	0,19	0
BAT1	HLA-B associated transcript 1	1,06	0,08	0,56	0,85	0	-1,06	-0,08	0,50	1,00	0	NA	NA	0
RPA1	replication protein A1, 70kDa	1,06	0,08	0,74	0,92	0	-1,12	-0,16	0,52	1,00	0	NA	NA	0
KIAA0664L3	KIAA0664-like 3	1,06	0,08	0,83	0,95	0	1,11	0,15	0,49	1,00	0	NA	NA	0
ERLIN1	ER lipid raft associated 1	1,06	0,08	0,76	0,93	0	-1,15	-0,20	0,83	1,00	0	-1,02	-0,02	0
PDLIM4	PDZ and LIM domain 4	1,06	0,08	0,62	0,88	0	-1,01	-0,01	0,95	1,00	0	NA	NA	0
ST6GAL1	ST6 beta-galactosamide alpha-2,6-sialyltranferase 1	1,06	0,08	0,83	0,95	0	-1,28	-0,35	0,25	1,00	0	1,03	0,04	0
KPNA1	karyopherin alpha 1 (importin alpha 5)	1,06	0,08	0,59	0,86	0	1,30	0,38	0,16	1,00	0	-1,41	-0,50	0
PLA2G7	phospholipase A2, group VII (platelet-activating factor acetylhydrolase, plasma)	1,06	0,08	0,78	0,94	0	-1,32	-0,40	0,10	1,00	0	NA	NA	0
C7orf49	chromosome 7 open reading frame 49	1,06	0,08	0,72	0,91	0	-1,18	-0,23	0,04	1,00	0	-1,11	-0,16	0
ZNF318	zinc finger protein 318	1,06	0,08	0,84	0,96	0	-1,47	-0,55	0,09	1,00	0	-1,43	-0,52	0
ATR	ataxia telangiectasia and Rad3 related	1,06	0,08	0,69	0,90	0	-1,05	-0,07	0,62	1,00	0	NA	NA	0
PBRM1	polybromo 1	1,06	0,08	0,71	0,91	0	-1,18	-0,24	0,29	1,00	0	NA	NA	0
ACTG2	actin, gamma 2, smooth muscle, enteric	1,06	0,08	0,65	0,89	0	-1,01	-0,01	NA	NA	0	1,29	0,37	0
NA	NA	1,06	0,08	0,81	0,95	0	-1,14	-0,19	0,17	1,00	0	NA	NA	0
LAPTM4B	lysosomal protein transmembrane 4 beta	1,06	0,08	0,54	0,84	0	1,03	0,05	0,63	1,00	0	NA	NA	0
CENPI	centromere protein I	1,06	0,08	0,72	0,92	0	-1,03	-0,04	0,94	1,00	0	NA	NA	0
TTLL12	tubulin tyrosine ligase-like family, member 12	1,06	0,08	0,63	0,88	0	1,08	0,11	0,48	1,00	0	NA	NA	0
GSDMD	gasdermin D	1,06	0,08	0,74	0,93	0	1,05	0,07	0,61	1,00	0	1,11	0,15	0
ENTPD4	ectonucleoside triphosphate diphosphohydrolase 4	1,06	0,08	0,64	0,89	0	1,01	0,01	0,92	1,00	0	NA	NA	0
NA	NA	1,06	0,08	0,68	0,90	0	1,06	0,09	0,80	1,00	0	NA	NA	0
PRKDC	protein kinase, DNA-activated, catalytic polypeptide	1,06	0,08	0,66	0,89	0	-1,15	-0,20	0,18	1,00	0	NA	NA	0
G6PC3	glucose 6 phosphatase, catalytic, 3	1,05	0,08	0,68	0,90	0	-1,03	-0,05	0,80	1,00	0	NA	NA	0
CSE1L	CSE1 chromosome segregation 1-like (yeast)	1,05	0,08	0,61	0,87	0	-1,02	-0,03	0,74	1,00	0	NA	NA	0
RAB23	RAB23, member RAS oncogene family	1,05	0,08	0,74	0,92	0	-1,03	-0,04	0,94	1,00	0	-1,22	-0,29	0
C12orf24	chromosome 12 open reading frame 24	1,05	0,08	0,83	0,95	0	1,22	0,29	0,32	1,00	0	-1,10	-0,13	0
TUBB2A	tubulin, beta 2A	1,05	0,08	0,70	0,91	0	1,10	0,14	0,20	1,00	0	NA	NA	0
BRCA1	breast cancer 1, early onset	1,05	0,08	0,59	0,87	0	1,07	0,10	0,66	1,00	0	NA	NA	0
BAZ1A	bromodomain adjacent to zinc finger domain, 1A	1,05	0,08	0,62	0,88	0	-1,00	0,00	0,96	1,00	0	-1,14	-0,19	0
PURA	purine-rich element binding protein A	1,05	0,08	0,67	0,90	0	1,00	0,00	0,98	1,00	0	1,01	0,01	0
TNIP2	TNFAIP3 interacting protein 2	1,05	0,07	0,67	0,90	0	1,01	0,02	0,93	1,00	0	NA	NA	0
HSPA9	heat shock 70kDa protein 9 (mortalin)	1,05	0,07	0,56	0,85	0	1,03	0,04	0,75	1,00	0	NA	NA	0
PSAT1	phosphoserine aminotransferase 1	1,05	0,07	0,76	0,93	0	-1,13	-0,17	0,72	1,00	0	0,00	-0,01	0
B4GALT5	UDP-Gal:betaGlcNAc beta 1,4- galactosyltransferase, polypeptide 5	1,05	0,07	0,82	0,95	0	1,01	0,02	0,91	1,00	0	1,02	0,03	0
BAG2	BCL2-associated athanogene 2	1,05	0,07	0,84	0,96	0	1,17	0,22	0,56	1,00	0	NA	NA	0
MEPCE	methylphosphate capping enzyme	1,05	0,07	0,69	0,91	0	1,04	0,06	0,66	1,00	0	NA	NA	0
SEH1L	SEH1-like (S. cerevisiae)	1,05	0,07	0,70	0,91	0	1,04	0,06	0,72	1,00	0	NA	NA	0
NA	NA	1,05	0,07	0,68	0,90	0	1,07	0,10	0,73	1,00	0	NA	NA	0
TNFRSF21	tumor necrosis factor receptor superfamily, member 21	1,05	0,07	0,80	0,95	0	-1,03	-0,04	0,86	1,00	0	1,02	0,02	0
RABEPK	Rab9 effector protein with kelch motifs	1,05	0,07	0,69	0,90	0	1,01	0,01	0,94	1,00	0	-1,16	-0,23	0
TCF4	transcription factor 4	1,05	0,07	0,74	0,93	0	-1,54	-0,62	0,02	0,88	0	NA	NA	0
UBE2O	ubiquitin-conjugating enzyme E2O	1,05	0,07	0,77	0,93	0	1,18	0,24	0,23	1,00	0	-1,15	-0,20	0
TPST2	tyrosylprotein sulfotransferase 2	1,05	0,07	0,67	0,90	0	1,02	0,02	0,85	1,00	0	-1,06	-0,09	0
RABEP2	rabaptin, RAB GTPase binding effector protein 2	1,05	0,07	0,77	0,93	0	1,05	0,06	0,78	1,00	0	1,32	0,40	0
FUCA1	fucosidase, alpha-L- 1, tissue	1,05	0,07	0,89	0,97	0	1,26	0,33	0,04	1,00	0	-1,35	-0,44	0
SLC5A6	solute carrier family 5 (sodium-dependent vitamin transporter), member 6	1,05	0,07	0,64	0,89	0	-1,04	-0,06	0,56	1,00	0	NA	NA	0
EIF1AX	eukaryotic translation initiation factor 1A, X-linked	1,05	0,07	0,63	0,88	0	1,11	0,15	0,16	1,00	0	-1,22	-0,29	0
CTSB	cathepsin B	1,05	0,07	0,77	0,94	0	-1,16	-0,22	0,56	1,00	0	NA	NA	0
SLC6A15	solute carrier family 6 (neutral amino acid transporter), member 15	1,05	0,07	0,70	0,91	0	1,09	0,13	0,41	1,00	0	NA	NA	0
PPP1CB	protein phosphatase 1, catalytic subunit, beta isozyme	1,05	0,07	0,63	0,88	0	1,05	0,08	0,74	1,00	0	-1,09	-0,11	0
ARNT	aryl hydrocarbon receptor nuclear translocator	1,05	0,07	0,77	0,93	0	-1,09	-0,13	0,39	1,00	0	-1,41	-0,50	0
TTLL4	tubulin tyrosine ligase-like family, member 4	1,05	0,07	0,63	0,88	0	1,02	0,03	0,85	1,00	0	NA	NA	0
SCARB2	scavenger receptor class B, member 2	1,05	0,07	0,70	0,91	0	1,06	0,08	0,90	1,00	0	-1,14	-0,18	0
CCDC88C	coiled-coil domain containing 88C	1,05	0,07	0,63	0,88	0	-1,08	-0,11	0,30	1,00	0	NA	NA	0
SEPHS1	selenophosphate synthetase 1	1,05	0,07	0,66	0,89	0	1,06	0,09	0,83	1,00	0	1,02	0,03	0
SART3	squamous cell carcinoma antigen recognized by T cells 3	1,05	0,07	0,63	0,88	0	1,03	0,04	0,84	1,00	0	NA	NA	0
NA	NA	1,05	0,07	0,82	0,95	0	NA	NA	NA	NA	0	NA	NA	0
EMP1	epithelial membrane protein 1	1,05	0,07	0,90	0,97	0	-1,60	-0,68	0,12	1,00	0	1,22	0,29	0
HYI	hydroxypyruvate isomerase (putative)	1,05	0,07	0,67	0,90	0	-1,03	-0,04	0,80	1,00	0	1,06	0,08	0
CEP57	centrosomal protein 57kDa	1,05	0,07	0,75	0,93	0	-1,61	-0,68	0,15	1,00	0	NA	NA	0
PMAIP1	phorbol-12-myristate-13-acetate-induced protein 1	1,05	0,07	0,80	0,94	0	1,17	0,23	0,21	1,00	0	1,06	0,08	0
NA	NA	1,05	0,07	0,64	0,88	0	1,11	0,15	0,49	1,00	0	1,05	0,07	0
CLASP2	cytoplasmic linker associated protein 2	1,05	0,07	0,69	0,91	0	1,01	0,02	0,85	1,00	0	NA	NA	0
MAX	MYC associated factor X	1,05	0,07	0,64	0,89	0	-1,05	-0,08	0,59	1,00	0	-1,33	-0,42	0
CRISPLD2	cysteine-rich secretory protein LCCL domain containing 2	1,05	0,07	0,78	0,94	0	1,71	0,77	0,06	1,00	0	NA	NA	0
BRD1	bromodomain containing 1	1,05	0,07	0,60	0,87	0	-1,03	-0,04	0,69	1,00	0	-1,14	-0,18	0
CREB1	cAMP responsive element binding protein 1	1,05	0,07	0,85	0,96	0	1,08	0,12	0,78	1,00	0	NA	NA	0
CASK	calcium/calmodulin-dependent serine protein kinase (MAGUK family)	1,05	0,07	0,68	0,90	0	-1,08	-0,11	0,41	1,00	0	NA	NA	0
CCNG2	cyclin G2	1,05	0,07	0,84	0,96	0	1,04	0,06	0,91	1,00	0	-1,47	-0,56	0
ATP2B1	ATPase, Ca++ transporting, plasma membrane 1	1,05	0,07	0,75	0,93	0	-1,15	-0,20	0,22	1,00	0	NA	NA	0
CD40	CD40 molecule, TNF receptor superfamily member 5	1,05	0,07	0,88	0,97	0	-1,02	-0,02	0,89	1,00	0	-1,01	-0,02	0
PJA1	praja ring finger 1	1,05	0,07	0,59	0,86	0	1,11	0,15	0,12	1,00	0	NA	NA	0
NDUFA6	NADH dehydrogenase (ubiquinone) 1 alpha subcomplex, 6, 14kDa	1,05	0,07	0,70	0,91	0	1,09	0,12	0,60	1,00	0	NA	NA	0
OBFC2A	oligonucleotide/oligosaccharide-binding fold containing 2A	1,05	0,07	0,84	0,96	0	1,22	0,29	0,07	1,00	0	-1,03	-0,04	0
CHD3	chromodomain helicase DNA binding protein 3	1,05	0,07	0,81	0,95	0	-1,08	-0,12	0,68	1,00	0	-1,12	-0,17	0
IPO5	importin 5	1,05	0,07	0,70	0,91	0	-1,05	-0,08	0,48	1,00	0	-1,02	-0,04	0
CHSY1	chondroitin sulfate synthase 1	1,05	0,07	0,69	0,91	0	1,04	0,06	0,54	1,00	0	NA	NA	0
RFC5	replication factor C (activator 1) 5, 36.5kDa	1,05	0,07	0,61	0,88	0	1,40	0,49	0,24	1,00	0	NA	NA	0
PIAS1	protein inhibitor of activated STAT, 1	1,05	0,07	0,69	0,90	0	-1,05	-0,07	0,41	1,00	0	NA	NA	0
SEL1L	sel-1 suppressor of lin-12-like (C. elegans)	1,05	0,07	0,65	0,89	0	-1,11	-0,14	0,15	1,00	0	NA	NA	0
HADH	hydroxyacyl-CoA dehydrogenase	1,05	0,07	0,68	0,90	0	1,47	0,55	0,03	0,95	0	NA	NA	0
GYS1	glycogen synthase 1 (muscle)	1,05	0,07	0,82	0,95	0	1,12	0,16	0,39	1,00	0	NA	NA	0
CD83	CD83 molecule	1,05	0,07	0,79	0,94	0	-1,08	-0,10	0,74	1,00	0	NA	NA	0
SLC35A4	solute carrier family 35, member A4	1,05	0,07	0,70	0,91	0	NA	NA	NA	NA	0	NA	NA	0
H2AFX	H2A histone family, member X	1,05	0,07	0,67	0,90	0	1,02	0,03	0,86	1,00	0	NA	NA	0
LIMK1	LIM domain kinase 1	1,05	0,07	0,70	0,91	0	-1,00	-0,01	0,94	1,00	0	-1,09	-0,12	0
TKT	transketolase	1,05	0,07	0,75	0,93	0	-1,10	-0,14	0,21	1,00	0	NA	NA	0
DAPK3	death-associated protein kinase 3	1,05	0,07	0,70	0,91	0	1,17	0,22	0,20	1,00	0	NA	NA	0
POLA1	polymerase (DNA directed), alpha 1, catalytic subunit	1,05	0,07	0,67	0,90	0	-1,16	-0,22	0,35	1,00	0	NA	NA	0
BEX4	brain expressed, X-linked 4	1,05	0,07	0,72	0,91	0	-1,19	-0,25	0,03	1,00	0	-1,33	-0,42	0
SUGP2	SURP and G patch domain containing 2	1,05	0,07	0,79	0,94	0	1,03	0,05	0,79	1,00	0	NA	NA	0
NA	NA	1,05	0,07	0,68	0,90	0	-1,02	-0,03	0,87	1,00	0	NA	NA	0

NRP2	neuropilin 2	1,05	0,07	0,73	0,92	0	1,14	0,18	0,39	1,00	0	NA	NA	0
TLK2	tousled-like kinase 2	1,05	0,07	0,86	0,96	0	-1,02	-0,03	0,95	1,00	0	NA	NA	0
NCAPH2	non-SMC condensin II complex, subunit H2	1,05	0,06	0,77	0,93	0	-1,00	0,00	NA	NA	0	NA	NA	0
DENND2D	DENN/MADD domain containing 2D	1,05	0,06	0,66	0,90	0	1,01	0,01	0,94	1,00	0	-1,04	-0,05	0
NPEPPS	aminopeptidase puromycin sensitive	1,05	0,06	0,85	0,96	0	1,15	0,20	0,34	1,00	0	NA	NA	0
TKT	transketolase	1,05	0,06	0,76	0,93	0	-1,14	-0,19	0,11	1,00	0	NA	NA	0
C8orf82	chromosome 8 open reading frame 82	1,05	0,06	0,70	0,91	0	NA	NA	NA	NA	0	NA	NA	0
MTAP	methylthioadenosine phosphorylase	1,05	0,06	0,69	0,90	0	-1,05	-0,07	0,62	1,00	0	NA	NA	0
MAP7D3	MAP7 domain containing 3	1,05	0,06	0,72	0,92	0	-1,18	-0,24	0,25	1,00	0	-1,47	-0,57	0
ZNF114	zinc finger protein 114	1,05	0,06	0,90	0,98	0	NA	NA	NA	NA	0	NA	NA	0
ZNF131	zinc finger protein 131	1,05	0,06	0,72	0,92	0	-1,03	-0,04	0,80	1,00	0	NA	NA	0
HEATR2	HEAT repeat containing 2	1,05	0,06	0,66	0,90	0	1,03	0,04	0,71	1,00	0	NA	NA	0
ATP11B	ATPase, class VI, type 11B	1,05	0,06	0,69	0,90	0	-1,09	-0,12	0,50	1,00	0	-1,12	-0,16	0
TRAF1	TNF receptor-associated factor 1	1,04	0,06	0,70	0,91	0	-1,00	-0,01	0,98	1,00	0	NA	NA	0
IVNS1ABP	influenza virus NS1A binding protein	1,04	0,06	0,78	0,94	0	-1,01	-0,02	0,92	1,00	0	NA	NA	0
PSMC3IP	PSMC3 interacting protein	1,04	0,06	0,67	0,90	0	1,06	0,09	0,60	1,00	0	NA	NA	0
PHTF2	putative homeodomain transcription factor 2	1,04	0,06	0,79	0,94	0	-1,01	-0,02	0,85	1,00	0	-1,22	-0,29	0
SF3B1	splicing factor 3b, subunit 1, 155kDa	1,04	0,06	0,74	0,92	0	1,05	0,07	0,95	1,00	0	-1,37	-0,46	0
MTMR9	myotubularin related protein 9	1,04	0,06	0,71	0,91	0	-1,15	-0,21	0,29	1,00	0	-1,33	-0,41	0
NA	NA	1,04	0,06	0,87	0,97	0	-1,05	-0,08	0,51	1,00	0	-1,03	-0,04	0
FYN	FYN oncogene related to SRC, FGR, YES	1,04	0,06	0,76	0,93	0	-1,44	-0,52	0,19	1,00	0	NA	NA	0
NA	NA	1,04	0,06	0,74	0,92	0	1,02	0,03	0,77	1,00	0	1,13	0,17	0
MLX	MAX-like protein X	1,04	0,06	0,68	0,90	0	1,05	0,07	0,51	1,00	0	NA	NA	0
GALNT7	UDP-N-acetyl-alpha-D-galactosamine:polypeptide N-acetylgalactosaminyltransferase 7 (GalNAc-T7)	1,04	0,06	0,85	0,96	0	1,08	0,11	0,35	1,00	0	-1,23	-0,30	0
HNRNPA0	heterogeneous nuclear ribonucleoprotein A0	1,04	0,06	0,62	0,88	0	1,01	0,01	0,96	1,00	0	-1,11	-0,16	0
ALG3	asparagine-linked glycosylation 3, alpha-1,3- mannosyltransferase homolog (S. cerevisiae)	1,04	0,06	0,75	0,93	0	1,16	0,21	0,15	1,00	0	NA	NA	0
ADSL	adenylosuccinate lyase	1,04	0,06	0,73	0,92	0	-1,00	0,00	0,99	1,00	0	NA	NA	0
TXNRD1	thioredoxin reductase 1	1,04	0,06	0,69	0,90	0	1,21	0,28	0,09	1,00	0	NA	NA	0
OGFOD1	2-oxoglutarate and iron-dependent oxygenase domain containing 1	1,04	0,06	0,72	0,92	0	-1,06	-0,08	0,61	1,00	0	NA	NA	0
TNFAIP1	tumor necrosis factor, alpha-induced protein 1 (endothelial)	1,04	0,06	0,71	0,91	0	1,13	0,18	0,19	1,00	0	NA	NA	0
B4GALT1	UDP-Gal:betaGlcNAc beta 1,4- galactosyltransferase, polypeptide 1	1,04	0,06	0,72	0,91	0	1,36	0,45	0,40	1,00	0	-1,02	-0,03	0
SLC25A4	solute carrier family 25 (mitochondrial carrier; adenine nucleotide translocator), member 4	1,04	0,06	0,77	0,94	0	1,13	0,18	0,12	1,00	0	1,02	0,02	0
PLXNA1	plexin A1	1,04	0,06	0,77	0,93	0	-1,21	-0,28	0,01	0,65	0	1,23	0,30	0
FKBP1A	FK506 binding protein 1A, 12kDa	1,04	0,06	0,78	0,94	0	-1,22	-0,29	0,28	1,00	0	1,38	0,47	0
RHOB	ras homolog gene family, member B	1,04	0,06	0,86	0,96	0	1,19	0,25	0,44	1,00	0	NA	NA	0
NA	NA	1,04	0,06	0,67	0,90	0	-1,03	-0,04	0,88	1,00	0	-1,82	-0,85	0
SETX	senataxin	1,04	0,06	0,73	0,92	0	-1,03	-0,04	0,74	1,00	0	-1,33	-0,42	0
WDR43	WD repeat domain 43	1,04	0,06	0,67	0,90	0	-1,04	-0,05	0,83	1,00	0	NA	NA	0
MSH6	mutS homolog 6 (E. coli)	1,04	0,06	0,68	0,90	0	-1,05	-0,06	0,54	1,00	0	NA	NA	0
SEC63	SEC63 homolog (S. cerevisiae)	1,04	0,06	0,73	0,92	0	-1,05	-0,07	0,63	1,00	0	NA	NA	0
ASB1	ankyrin repeat and SOCS box-containing 1	1,04	0,06	0,78	0,94	0	-1,07	-0,09	0,53	1,00	0	1,11	0,15	0
STK3	serine/threonine kinase 3	1,04	0,06	0,79	0,94	0	1,32	0,40	0,62	1,00	0	-1,45	-0,53	0
FHOD1	formin homology 2 domain containing 1	1,04	0,06	0,86	0,97	0	-1,15	-0,20	0,33	1,00	0	NA	NA	0
QSOX1	quiescin Q6 sulfhydryl oxidase 1	1,04	0,06	0,77	0,93	0	1,08	0,11	0,68	1,00	0	NA	NA	0
MAP1S	microtubule-associated protein 1S	1,04	0,06	0,89	0,97	0	-1,12	-0,16	0,68	1,00	0	1,11	0,15	0
TPM2	tropomyosin 2 (beta)	1,04	0,06	0,78	0,94	0	-1,11	-0,15	0,46	1,00	0	NA	NA	0
INO80B	INO80 complex subunit B	1,04	0,06	0,82	0,95	0	-1,06	-0,08	0,52	1,00	0	NA	NA	0
NME7	non-metastatic cells 7, protein expressed in (nucleoside-diphosphate kinase)	1,04	0,06	0,77	0,94	0	-1,08	-0,12	0,50	1,00	0	NA	NA	0
RYBP	RING1 and YY1 binding protein	1,04	0,06	0,88	0,97	0	-1,03	-0,05	0,82	1,00	0	NA	NA	0
RALGAPA1	Ral GTPase activating protein, alpha subunit 1 (catalytic)	1,04	0,06	0,69	0,90	0	-1,07	-0,10	0,49	1,00	0	NA	NA	0
KRT19	keratin 19	1,04	0,06	0,96	0,99	0	-1,14	-0,19	0,20	1,00	0	-1,47	-0,57	0
WASF3	WAS protein family, member 3	1,04	0,05	0,80	0,94	0	-1,07	-0,10	0,64	1,00	0	-1,04	-0,06	0
GALNT10	UDP-N-acetyl-alpha-D-galactosamine:polypeptide N-acetylgalactosaminyltransferase 10 (GalNAc-T10)	1,04	0,05	0,81	0,95	0	-1,30	-0,37	0,09	1,00	0	-1,10	-0,14	0
NPRL3	nitrogen permease regulator-like 3 (S. cerevisiae)	1,04	0,05	0,73	0,92	0	1,11	0,15	0,39	1,00	0	NA	NA	0
ANKRD12	ankyrin repeat domain 12	1,04	0,05	0,77	0,93	0	-1,48	-0,56	0,43	1,00	0	-1,49	-0,58	0
ORMDL2	ORM1-like 2 (S. cerevisiae)	1,04	0,05	0,75	0,93	0	1,11	0,15	0,27	1,00	0	NA	NA	0
ABCE1	ATP-binding cassette, sub-family E (OABP), member 1	1,04	0,05	0,78	0,94	0	-1,03	-0,05	0,77	1,00	0	NA	NA	0
SYNJ2	synaptojanin 2	1,04	0,05	0,79	0,94	0	1,02	0,03	0,84	1,00	0	NA	NA	0
BTG1	B-cell translocation gene 1, anti-proliferative	1,04	0,05	0,86	0,96	0	-1,23	-0,29	0,17	1,00	0	NA	NA	0
SLC35F2	solute carrier family 35, member F2	1,04	0,05	0,72	0,91	0	1,04	0,06	0,58	1,00	0	-1,39	-0,48	0
NA	NA	1,04	0,05	0,91	0,98	0	-1,02	-0,03	NA	NA	0	1,38	0,47	0
EVI5	ecotropic viral integration site 5	1,04	0,05	0,79	0,94	0	1,01	0,01	0,92	1,00	0	-1,01	-0,02	0
ORAI2	ORAI calcium release-activated calcium modulator 2	1,04	0,05	0,89	0,97	0	-1,05	-0,06	NA	NA	0	1,10	0,13	0
TTC3	tetratricopeptide repeat domain 3	1,04	0,05	0,83	0,95	0	-1,17	-0,22	0,66	1,00	0	-1,56	-0,64	0
ZMYND11	zinc finger, MYND domain containing 11	1,04	0,05	0,85	0,96	0	1,19	0,25	0,67	1,00	0	-1,20	-0,27	0
WSB1	WD repeat and SOCS box-containing 1	1,04	0,05	0,89	0,97	0	-1,43	-0,52	0,52	1,00	0	-1,92	-0,95	0
PAK2	p21 protein (Cdc42/Rac)-activated kinase 2	1,04	0,05	0,77	0,93	0	-1,07	-0,09	0,59	1,00	0	NA	NA	0
TAP2	transporter 2, ATP-binding cassette, sub-family B (MDR/TAP)	1,04	0,05	0,88	0,97	0	1,00	0,00	0,99	1,00	0	-1,05	-0,08	0
GIMAP2	GTPase, IMAP family member 2	1,04	0,05	0,72	0,91	0	NA	NA	NA	NA	0	NA	NA	0
ME2	malic enzyme 2, NAD(+)-dependent, mitochondrial	1,04	0,05	0,77	0,93	0	-1,06	-0,09	0,76	1,00	0	-1,19	-0,25	0
PAX8	paired box 8	1,04	0,05	0,74	0,92	0	-1,13	-0,18	0,34	1,00	0	NA	NA	0
BZW2	basic leucine zipper and W2 domains 2	1,04	0,05	0,71	0,91	0	1,03	0,04	0,75	1,00	0	NA	NA	0
MTDH	metadherin	1,03	0,05	0,82	0,95	0	1,06	0,09	0,49	1,00	0	NA	NA	0
CTNNB1	catenin (cadherin-associated protein), beta 1, 88kDa	1,03	0,05	0,72	0,91	0	1,09	0,13	0,60	1,00	0	-1,09	-0,13	0
QKI	quaking homolog, KH domain RNA binding (mouse)	1,03	0,05	0,83	0,95	0	1,05	0,07	0,54	1,00	0	NA	NA	0
NDUFA9	NADH dehydrogenase (ubiquinone) 1 alpha subcomplex, 9, 39kDa	1,03	0,05	0,76	0,93	0	1,11	0,15	0,17	1,00	0	-1,09	-0,12	0
CD47	CD47 molecule	1,03	0,05	0,79	0,94	0	1,17	0,22	0,03	1,00	0	-1,19	-0,25	0
SS18L1	synovial sarcoma translocation gene on chromosome 18-like 1	1,03	0,05	0,77	0,93	0	1,02	0,02	0,91	1,00	0	NA	NA	0
ANAPC1	anaphase promoting complex subunit 1	1,03	0,05	0,83	0,95	0	-1,04	-0,06	0,72	1,00	0	NA	NA	0
NR3C1	nuclear receptor subfamily 3, group C, member 1 (glucocorticoid receptor)	1,03	0,05	0,83	0,96	0	-1,10	-0,14	0,46	1,00	0	-1,04	-0,06	0
XPO7	exportin 7	1,03	0,05	0,70	0,91	0	-1,12	-0,16	0,27	1,00	0	NA	NA	0
ASB1	ankyrin repeat and SOCS box-containing 1	1,03	0,05	0,79	0,94	0	-1,06	-0,08	0,25	1,00	0	1,16	0,22	0
WSB1	WD repeat and SOCS box-containing 1	1,03	0,05	0,82	0,95	0	-1,03	-0,04	0,85	1,00	0	NA	NA	0
MAGT1	magnesium transporter 1	1,03	0,05	0,75	0,93	0	1,02	0,03	0,93	1,00	0	NA	NA	0
SMC4	structural maintenance of chromosomes 4	1,03	0,05	0,77	0,94	0	-1,05	-0,07	0,50	1,00	0	NA	NA	0
ELAC2	elaC homolog 2 (E. coli)	1,03	0,05	0,81	0,95	0	-1,04	-0,05	0,65	1,00	0	NA	NA	0
KLHL9	kelch-like 9 (Drosophila)	1,03	0,05	0,83	0,95	0	-1,34	-0,42	0,01	0,70	0	-1,16	-0,23	0
ERGIC2	ERGIC and golgi 2	1,03	0,05	0,77	0,94	0	NA	NA	NA	NA	0	NA	NA	0
C19orf2	chromosome 19 open reading frame 2	1,03	0,05	0,76	0,93	0	-1,18	-0,23	0,33	1,00	0	NA	NA	0
NSFL1C	NSFL1 (p97) cofactor (p47)	1,03	0,05	0,85	0,96	0	1,39	0,48	0,03	1,00	0	NA	NA	0
JAG1	jagged 1	1,03	0,05	0,75	0,93	0	1,14	0,19	0,11	1,00	0	1,39	0,47	0
ORC5L	origin recognition complex, subunit 5-like (yeast)	1,03	0,05	0,83	0,95	0	-1,02	-0,03	0,92	1,00	0	NA	NA	0
WRN	Werner syndrome, RecQ helicase-like	1,03	0,05	0,76	0,93	0	-1,28	-0,36	0,21	1,00	0	-1,11	-0,14	0
FOXO1	forkhead box O1	1,03	0,05	0,89	0,97	0	-1,19	-0,25	0,13	1,00	0	-1,08	-0,11	0
CREB3	cAMP responsive element binding protein 3	1,03	0,05	0,81	0,95	0	1,26	0,34	0,14	1,00	0	NA	NA	0
WWTR1	WW domain containing transcription regulator 1	1,03	0,05	0,76	0,93	0	1,01	0,02	0,91	1,00	0	NA	NA	0
NA	NA	1,03	0,05	0,81	0,95	0	-1,25	-0,32	0,21	1,00	0	NA	NA	0
ERO1L	ERO1-like (S. cerevisiae)	1,03	0,04	0,83	0,96	0	1,09	0,12	0,48	1,00	0	-1,10	-0,14	0
ABAT	4-aminobutyrate aminotransferase	1,03	0,04	0,90	0,97	0	1,21	0,27	0,19	1,00	0	NA	NA	0
RBMS2	RNA binding motif, single stranded interacting protein 2	1,03	0,04	0,83	0,95	0	1,05	0,07	0,80	1,00	0	-1,30	-0,37	0
NA	NA	1,03	0,04	0,81	0,95	0	-1,14	-0,19	0,41	1,00	0	NA	NA	0
ACP2	acid phosphatase 2, lysosomal	1,03	0,04	0,89	0,97	0	1,24	0,31	0,14	1,00	0	-1,19	-0,25	0
ANXA7	annexin A7	1,03	0,04	0,81	0,95	0	1,14	0,19	0,21	1,00	0	NA	NA	0
ARFGEF1	ADP-ribosylation factor guanine nucleotide-exchange factor 1(brefeldin A-inhibited)	1,03	0,04	0,76	0,93	0	-1,05	-0,07	0,77	1,00	0	-1,19	-0,26	0
CCDC85B	coiled-coil domain containing 85B	1,03	0,04	0,83	0,96	0	-1,31	-0,38	0,04	1,00	0	NA	NA	0
SCPEP1	serine carboxypeptidase 1	1,03	0,04	0,84	0,96	0	-1,28	-0,36	0,03	0,97	0	-1,22	-0,28	0
UBQLN2	ubiquilin 2	1,03	0,04	0,80	0,95	0	1,05	0,07	0,57	1,00	0	NA	NA	0
APC	adenomatous polyposis coli	1,03	0,04	0,75	0,93	0	-1,03	-0,05	0,64	1,00	0	-1,22	-0,29	0
FZD3	frizzled homolog 3 (Drosophila)	1,03	0,04	0,88	0,97	0	1,08	0,11	0,78	1,00	0	NA	NA	0
IPO7	importin 7	1,03	0,04	0,76	0,93	0	-1,15	-0,20	0,18	1,00	0	NA	NA	0
PRPF4	PRP4 pre-mRNA processing factor 4 homolog (yeast)	1,03	0,04	0,78	0,94	0	1,06	0,09	0,57	1,00	0	NA	NA	0
SCARB1	scavenger receptor class B, member 1	1,03	0,04	0,81	0,95	0	1,16	0,22	0,19	1,00	0	NA	NA	0
ECT2	epithelial cell transforming sequence 2 oncogene	1,03	0,04	0,86	0,96	0	-1,14	-0,19	0,17	1,00	0	NA	NA	0
KDM3A	lysine (K)-specific demethylase 3A	1,03	0,04	0,86	0,96	0	1,15	0,21	0,10	1,00	0	NA	NA	0
PRKD2	protein kinase D2	1,03	0,04	0,91	0,98	0	-1,10	-0,14	0,46	1,00	0	1,15	0,20	0
BDKRB1	bradykinin receptor B1	1,03	0,04	0,96	0,99	0	-1,10	-0,13	0,62	1,00	0	NA	NA	0
RBM9	RNA binding motif protein 9	1,03	0,04	0,85	0,96	0	-1,00	-0,01	NA	NA	0	-1,03	-0,04	0
NF2	neurofibromin 2 (merlin)	1,03	0,04	0,87	0,97	0	-1,26	-0,33	0,25	1,00	0	NA	NA	0
CEBPD	CCAAT/enhancer binding protein (C/EBP), delta	1,03	0,04	0,87	0,97	0	1,19	0,25	0,03	1,00	0	NA	NA	0
SIRT7	sirtuin 7	1,03	0,04	0,84	0,96	0	1,07	0,09	0,53	1,00	0	NA	NA	0
PSD3	pleckstrin and Sec7 domain containing 3	1,03	0,04	0,86	0,96	0	1,07	0,10	0,67	1,00	0	-1,52	-0,61	0
IMP3	IMP3, U3 small nucleolar ribonucleoprotein, homolog (yeast)	1,03	0,04	0,85	0,96	0	-1,11	-0,15	0,13	1,00	0	NA	NA	0
FXN	frataxin	1,03	0,04	0,86	0,96	0	-1,07	-0,10	0,49	1,00	0	NA	NA	0
XAF1	XIAP associated factor 1	1,03	0,04	0,91	0,98	0	NA	NA	NA	NA	0	NA	NA	0
NXT1	NTF2-like export factor 1	1,03	0,04	0,86	0,96	0	1,09	0,12	0,35	1,00	0	NA	NA	0
USP34	ubiquitin specific peptidase 34	1,03	0,04	0,79	0,94	0	1,02	0,03	0,74	1,00	0	-1,03	-0,05	0
PGAP2	post-GPI attachment to proteins 2	1,03	0,04	0,80	0,94	0	-1,07	-0,10	0,45	1,00	0	NA	NA	0
PALLD	palladin, cytoskeletal associated protein	1,03	0,04	0,78	0,94	0	1,09	0,12	0,23	1,00	0	-1,11	-0,15	0
ENOSF1	enolase superfamily member 1	1,03	0,04	0,86	0,96	0	1,06	0,08	0,56	1,00	0	NA	NA	0
STEAP1	six transmembrane epithelial antigen of the prostate 1	1,03	0,04	0,88	0,97	0	1,08	0,11	0,51	1,00	0	NA	NA	0
RALB	v-ral simian leukemia viral oncogene homolog B (ras related; GTP binding protein)	1,03	0,04	0,89	0,97	0	1,09	0,12	0,35	1,00	0	NA	NA	0
GTF2B	general transcription factor IIB	1,03	0,04	0,83	0,95	0	1,08	0,11	0,31	1,00	0	NA	NA	0
OBFC2B	oligonucleotide/oligosaccharide-binding fold containing 2B	1,03	0,04	0,82	0,95	0	-1,33	-0,41	0,03	1,00	0	NA	NA	0
PFKP	phosphofructokinase, platelet	1,03	0,04	0,90	0,97	0	1,04	0,06	0,52	1,00	0	-1,02	-0,04	0
SERINC5	serine incorporator 5	1,03	0,04	0,84	0,96	0	1,12	0,16	0,23	1,00	0	-1,23	-0,30	0
SH3BP2	SH3-domain binding protein 2	1,03	0,04	0,91	0,98	0	1,02	0,02	0,93	1,00	0	-1,04	-0,06	0
GUK1	guanylate kinase 1	1,03	0,04	0,83	0,96	0	1,00	0,00	NA	NA	0	NA	NA	0

MDC1	mediator of DNA-damage checkpoint 1	1,03	0,04	0,84	0,96	0	-1,20	-0,26	0,30	1,00	0	NA	NA	0
WDR3	WD repeat domain 3	1,03	0,04	0,83	0,95	0	1,13	0,18	0,15	1,00	0	NA	NA	0
CD40	CD40 molecule, TNF receptor superfamily member 5	1,03	0,04	0,93	0,98	0	1,02	0,03	0,90	1,00	0	1,06	0,09	0
BCL11B	B-cell CLL/lymphoma 11B (zinc finger protein)	1,03	0,04	0,93	0,98	0	-1,10	-0,14	0,41	1,00	0	NA	NA	0
IL32	interleukin 32	1,03	0,04	0,89	0,97	0	-1,98	-0,99	0,20	1,00	0	0,00	0,00	0
NA	NA	1,03	0,04	0,91	0,98	0	1,11	0,15	0,20	1,00	0	-1,09	-0,11	0
EIF4B	eukaryotic translation initiation factor 4B	1,03	0,04	0,79	0,94	0	-2,14	-1,10	0,08	1,00	0	NA	NA	0
TNPO2	transportin 2	1,03	0,04	0,88	0,97	0	-1,12	-0,16	0,32	1,00	0	NA	NA	0
RNMT	RNA (guanine-7-) methyltransferase	1,02	0,04	0,81	0,95	0	-1,00	-0,01	0,97	1,00	0	NA	NA	0
ETFB	electron-transfer-flavoprotein, beta polypeptide	1,02	0,04	0,90	0,97	0	-1,04	-0,05	0,81	1,00	0	NA	NA	0
POSTN	periostin, osteoblast specific factor	1,02	0,03	0,91	0,98	0	-1,05	-0,07	0,49	1,00	0	1,10	0,14	0
FOXN3	forkhead box N3	1,02	0,03	0,87	0,97	0	-1,07	-0,10	0,62	1,00	0	-1,79	-0,83	0
ARPC4	actin related protein 2/3 complex, subunit 4, 20kDa	1,02	0,03	0,87	0,97	0	1,77	0,82	0,20	1,00	0	NA	NA	0
C3orf64	chromosome 3 open reading frame 64	1,02	0,03	0,88	0,97	0	-1,18	-0,23	0,16	1,00	0	NA	NA	0
NA	NA	1,02	0,03	0,79	0,94	0	NA	NA	NA	NA	0	NA	NA	0
LIN37	lin-37 homolog (C. elegans)	1,02	0,03	0,84	0,96	0	-1,01	-0,01	0,94	1,00	0	NA	NA	0
COPS2	COP9 constitutive photomorphogenic homolog subunit 2 (Arabidopsis)	1,02	0,03	0,86	0,97	0	1,01	0,02	0,83	1,00	0	-1,09	-0,12	0
NRP1	neuropilin 1	1,02	0,03	0,89	0,97	0	-1,01	-0,01	NA	NA	0	-1,22	-0,29	0
TDG	thymine-DNA glycosylase	1,02	0,03	0,81	0,95	0	1,04	0,05	0,68	1,00	0	-1,09	-0,11	0
PON2	paraoxonase 2	1,02	0,03	0,90	0,98	0	1,01	0,02	0,91	1,00	0	NA	NA	0
PKP4	plakophilin 4	1,02	0,03	0,88	0,97	0	-1,27	-0,34	0,00	0,44	0	-1,30	-0,37	0
ATP2A2	ATPase, Ca++ transporting, cardiac muscle, slow twitch 2	1,02	0,03	0,88	0,97	0	1,03	0,04	0,78	1,00	0	0,00	0,01	0
FAM20B	family with sequence similarity 20, member B	1,02	0,03	0,83	0,96	0	1,07	0,10	0,59	1,00	0	NA	NA	0
PAOX	polyamine oxidase (exo-N4-amino)	1,02	0,03	0,87	0,97	0	-1,07	-0,10	0,69	1,00	0	NA	NA	0
ZHX2	zinc fingers and homeoboxes 2	1,02	0,03	0,87	0,97	0	1,06	0,08	0,46	1,00	0	1,10	0,14	0
DZIP1	DAZ interacting protein 1	1,02	0,03	0,92	0,98	0	-1,06	-0,08	0,86	1,00	0	NA	NA	0
GALNT3	UDP-N-acetyl-alpha-D-galactosamine:polypeptide N-acetylgalactosaminyltransferase 3 (GalNAc-T3)	1,02	0,03	0,89	0,97	0	-1,05	-0,07	0,68	1,00	0	-1,16	-0,22	0
ARG2	arginase, type II	1,02	0,03	0,89	0,97	0	-1,00	-0,01	0,99	1,00	0	NA	NA	0
ACP5	acid phosphatase 5, tartrate resistant	1,02	0,03	0,89	0,97	0	-1,07	-0,10	0,37	1,00	0	NA	NA	0
ADAT1	adenosine deaminase, tRNA-specific 1	1,02	0,03	0,91	0,98	0	1,08	0,10	0,58	1,00	0	NA	NA	0
KPNA1	karyopherin alpha 1 (importin alpha 5)	1,02	0,03	0,81	0,95	0	-1,13	-0,18	0,46	1,00	0	-1,15	-0,21	0
MAD2L1	MAD2 mitotic arrest deficient-like 1 (yeast)	1,02	0,03	0,92	0,98	0	-1,08	-0,11	0,26	1,00	0	NA	NA	0
MED16	mediator complex subunit 16	1,02	0,03	0,92	0,98	0	1,08	0,11	0,65	1,00	0	NA	NA	0
NBEAL2	neurobeachin-like 2	1,02	0,03	0,90	0,98	0	1,19	0,26	0,34	1,00	0	NA	NA	0
C7orf41	chromosome 7 open reading frame 41	1,02	0,03	0,87	0,97	0	NA	NA	NA	NA	0	NA	NA	0
S100A13	S100 calcium binding protein A13	1,02	0,03	0,89	0,97	0	-1,02	-0,02	0,91	1,00	0	NA	NA	0
SLC7A8	solute carrier family 7 (amino acid transporter, L-type), member 8	1,02	0,03	0,85	0,96	0	-1,12	-0,17	0,49	1,00	0	NA	NA	0
R3HCC1	R3H domain and coiled-coil containing 1	1,02	0,03	0,87	0,97	0	-1,04	-0,06	0,70	1,00	0	NA	NA	0
NA	NA	1,02	0,03	0,92	0,98	0	1,00	0,00	0,98	1,00	0	NA	NA	0
FAH	fumarylacetoacetate hydrolase (fumarylacetoacetase)	1,02	0,03	0,91	0,98	0	-1,05	-0,07	0,54	1,00	0	1,05	0,07	0
E2F3	E2F transcription factor 3	1,02	0,03	0,85	0,96	0	1,18	0,24	0,25	1,00	0	NA	NA	0
ASMTL	acetylserotonin O-methyltransferase-like	1,02	0,03	0,91	0,98	0	-1,03	-0,04	0,87	1,00	0	-1,14	-0,18	0
ZNF814	zinc finger protein 814	1,02	0,03	0,85	0,96	0	-1,39	-0,48	0,50	1,00	0	1,65	0,72	0
MED16	mediator complex subunit 16	1,02	0,03	0,89	0,97	0	1,02	0,03	0,91	1,00	0	1,54	0,62	0
HSP90B1	heat shock protein 90kDa beta (Grp94), member 1	1,02	0,03	0,83	0,95	0	-1,12	-0,16	0,45	1,00	0	NA	NA	0
C7orf68	chromosome 7 open reading frame 68	1,02	0,03	0,93	0,98	0	1,13	0,18	0,28	1,00	0	-1,03	-0,04	0
CSE1L	CSE1 chromosome segregation 1-like (yeast)	1,02	0,03	0,83	0,95	0	1,02	0,03	0,84	1,00	0	NA	NA	0
ICAM2	intercellular adhesion molecule 2	1,02	0,03	0,93	0,98	0	-1,13	-0,17	0,64	1,00	0	NA	NA	0
DICER1	dicer 1, ribonuclease type III	1,02	0,03	0,85	0,96	0	1,14	0,18	0,10	1,00	0	NA	NA	0
RELA	v-rel reticuloendotheliosis viral oncogene homolog A (avian)	1,02	0,03	0,86	0,96	0	1,01	0,01	0,96	1,00	0	NA	NA	0
EIF3A	eukaryotic translation initiation factor 3, subunit A	1,02	0,03	0,88	0,97	0	-1,07	-0,10	0,37	1,00	0	NA	NA	0
GADD45B	growth arrest and DNA-damage-inducible, beta	1,02	0,03	0,92	0,98	0	1,37	0,45	0,59	1,00	0	NA	NA	0
SOX4	SRY (sex determining region Y)-box 4	1,02	0,03	0,96	0,99	0	-1,07	-0,10	0,75	1,00	0	NA	NA	0
MTMR14	myotubularin related protein 14	1,02	0,03	0,86	0,96	0	1,01	0,02	0,89	1,00	0	NA	NA	0
FBXL5	F-box and leucine-rich repeat protein 5	1,02	0,03	0,89	0,97	0	-1,02	-0,02	0,80	1,00	0	NA	NA	0
CTGF	connective tissue growth factor	1,02	0,03	0,95	0,99	0	-1,30	-0,38	0,10	1,00	0	-1,10	-0,14	0
NPM3	nucleophosmin/nucleoplasmin 3	1,02	0,03	0,92	0,98	0	-1,00	-0,01	0,97	1,00	0	-1,19	-0,26	0
RRP1B	ribosomal RNA processing 1 homolog B (S. cerevisiae)	1,02	0,03	0,88	0,97	0	-1,10	-0,14	0,26	1,00	0	NA	NA	0
RBL1	retinoblastoma-like 1 (p107)	1,02	0,03	0,92	0,98	0	-1,09	-0,13	0,54	1,00	0	NA	NA	0
GUK1	guanylate kinase 1	1,02	0,03	0,89	0,97	0	-1,05	-0,08	0,37	1,00	0	1,10	0,14	0
PARD3	par-3 partitioning defective 3 homolog (C. elegans)	1,02	0,03	0,91	0,98	0	1,01	0,02	0,96	1,00	0	NA	NA	0
FAHD2A	fumarylacetoacetate hydrolase domain containing 2A	1,02	0,02	0,91	0,98	0	-1,22	-0,28	0,02	0,94	0	NA	NA	0
CTDSP2	CTD (carboxy-terminal domain, RNA polymerase II, polypeptide A) small phosphatase 2	1,02	0,02	0,93	0,98	0	-1,08	-0,11	0,55	1,00	0	NA	NA	0
NA	NA	1,02	0,02	0,92	0,98	0	-1,06	-0,08	0,76	1,00	0	1,38	0,46	0
TMEM41B	transmembrane protein 41B	1,02	0,02	0,89	0,97	0	1,08	0,11	0,48	1,00	0	-1,20	-0,26	0
FASTKD1	FAST kinase domains 1	1,02	0,02	0,90	0,97	0	1,09	0,12	0,34	1,00	0	NA	NA	0
EML6	echinoderm microtubule associated protein like 6	1,02	0,02	0,91	0,98	0	NA	NA	NA	NA	0	NA	NA	0
TFPT	TCF3 (E2A) fusion partner (in childhood Leukemia)	1,02	0,02	0,93	0,98	0	-1,04	-0,06	0,74	1,00	0	NA	NA	0
KIAA0232	KIAA0232	1,02	0,02	0,87	0,97	0	1,10	0,14	0,40	1,00	0	-1,01	-0,02	0
CLIP1	CAP-GLY domain containing linker protein 1	1,02	0,02	0,89	0,97	0	1,10	0,13	0,42	1,00	0	NA	NA	0
RRP15	ribosomal RNA processing 15 homolog (S. cerevisiae)	1,02	0,02	0,90	0,98	0	-1,21	-0,28	0,18	1,00	0	-1,12	-0,17	0
ASMTL	acetylserotonin O-methyltransferase-like	1,02	0,02	0,93	0,98	0	-1,41	-0,49	0,14	1,00	0	-1,09	-0,12	0
PDP1	pyruvate dehyrogenase phosphatase catalytic subunit 1	1,02	0,02	0,96	0,99	0	-1,06	-0,08	0,92	1,00	0	-1,67	-0,73	0
KIAA1539	KIAA1539	1,02	0,02	0,91	0,98	0	1,18	0,23	0,11	1,00	0	NA	NA	0
PTPLAD1	protein tyrosine phosphatase-like A domain containing 1	1,02	0,02	0,87	0,97	0	-1,10	-0,14	0,66	1,00	0	NA	NA	0
GPN3	GPN-loop GTPase 3	1,02	0,02	0,91	0,98	0	-1,11	-0,15	0,42	1,00	0	NA	NA	0
SGPL1	sphingosine-1-phosphate lyase 1	1,02	0,02	0,91	0,98	0	-1,04	-0,05	0,68	1,00	0	1,06	0,08	0
C1orf163	chromosome 1 open reading frame 163	1,02	0,02	0,89	0,97	0	1,37	0,45	0,02	0,88	0	NA	NA	0
RB1CC1	RB1-inducible coiled-coil 1	1,02	0,02	0,90	0,98	0	-1,06	-0,08	0,31	1,00	0	-1,10	-0,14	0
GART	phosphoribosylglycinamide formyltransferase, phosphoribosylglycinamide synthetase, phosphoribosylaminoimidazole synthetase	1,02	0,02	0,93	0,98	0	-1,00	0,00	NA	NA	0	0,00	0,01	0
NA	NA	1,02	0,02	0,88	0,97	0	1,01	0,01	0,96	1,00	0	-1,03	-0,04	0
SH3TC1	SH3 domain and tetratricopeptide repeats 1	1,02	0,02	0,97	1,00	0	-1,32	-0,40	0,07	1,00	0	NA	NA	0
SMAD3	SMAD family member 3	1,01	0,02	0,94	0,99	0	-1,12	-0,16	0,47	1,00	0	NA	NA	0
PDCD2	programmed cell death 2	1,01	0,02	0,90	0,98	0	-1,00	0,00	1,00	1,00	0	NA	NA	0
UBE4B	ubiquitination factor E4B (UFD2 homolog, yeast)	1,01	0,02	0,90	0,97	0	-1,07	-0,09	0,44	1,00	0	1,02	0,03	0
ANXA11	annexin A11	1,01	0,02	0,95	0,99	0	1,01	0,01	0,93	1,00	0	NA	NA	0
GGA3	golgi-associated, gamma adaptin ear containing, ARF binding protein 3	1,01	0,02	0,93	0,98	0	1,13	0,17	0,37	1,00	0	NA	NA	0
PNN	pinin, desmosome associated protein	1,01	0,02	0,92	0,98	0	1,07	0,09	0,48	1,00	0	NA	NA	0
PTGES	prostaglandin E synthase	1,01	0,02	0,90	0,97	0	-1,04	-0,06	0,76	1,00	0	NA	NA	0
EXT1	exostosin 1	1,01	0,02	0,93	0,98	0	1,04	0,06	NA	NA	0	1,39	0,48	0
NUP160	nucleoporin 160kDa	1,01	0,02	0,94	0,98	0	-1,67	-0,74	0,13	1,00	0	NA	NA	0
TRAK2	trafficking protein, kinesin binding 2	1,01	0,02	0,90	0,97	0	-1,01	-0,01	0,93	1,00	0	-1,39	-0,47	0
SDC1	syndecan 1	1,01	0,02	0,89	0,97	0	1,01	0,01	0,94	1,00	0	-1,12	-0,17	0
MYO9B	myosin IXB	1,01	0,02	0,95	0,99	0	-1,07	-0,10	0,69	1,00	0	1,41	0,50	0
RPUSD2	RNA pseudouridylate synthase domain containing 2	1,01	0,02	0,92	0,98	0	1,00	0,00	0,99	1,00	0	NA	NA	0
SEMA3F	sema domain, immunoglobulin domain (Ig), short basic domain, secreted, (semaphorin) 3F	1,01	0,02	0,92	0,98	0	-1,12	-0,17	0,33	1,00	0	1,29	0,37	0
SLC7A8	solute carrier family 7 (amino acid transporter, L-type), member 8	1,01	0,02	0,90	0,98	0	-1,10	-0,13	0,64	1,00	0	NA	NA	0
GGA1	golgi-associated, gamma adaptin ear containing, ARF binding protein 1	1,01	0,02	0,94	0,98	0	1,06	0,09	0,61	1,00	0	1,15	0,20	0
ANP32E	acidic (leucine-rich) nuclear phosphoprotein 32 family, member E	1,01	0,02	0,93	0,98	0	1,03	0,05	0,65	1,00	0	NA	NA	0
BRD3	bromodomain containing 3	1,01	0,02	0,90	0,98	0	-1,18	-0,24	0,14	1,00	0	NA	NA	0
APOL6	apolipoprotein L, 6	1,01	0,02	0,96	0,99	0	NA	NA	NA	NA	0	NA	NA	0
COG5	component of oligomeric golgi complex 5	1,01	0,02	0,93	0,98	0	1,19	0,24	0,45	1,00	0	-1,49	-0,57	0
SLC17A5	solute carrier family 17 (anion/sugar transporter), member 5	1,01	0,02	0,99	1,00	0	-1,08	-0,11	0,57	1,00	0	-1,35	-0,44	0
FMR1	fragile X mental retardation 1	1,01	0,02	0,93	0,98	0	-1,04	-0,06	0,52	1,00	0	-1,15	-0,21	0
UBE4B	ubiquitination factor E4B (UFD2 homolog, yeast)	1,01	0,02	0,91	0,98	0	-1,10	-0,14	0,59	1,00	0	NA	NA	0
SLC16A1	solute carrier family 16, member 1 (monocarboxylic acid transporter 1)	1,01	0,02	0,92	0,98	0	1,17	0,22	0,32	1,00	0	NA	NA	0
GRSF1	G-rich RNA sequence binding factor 1	1,01	0,02	0,97	0,99	0	1,20	0,26	0,57	1,00	0	0,00	-0,01	0
CBX5	chromobox homolog 5	1,01	0,01	0,92	0,98	0	-1,06	-0,08	0,65	1,00	0	NA	NA	0
EIF4B	eukaryotic translation initiation factor 4B	1,01	0,01	0,91	0,98	0	-1,17	-0,23	0,16	1,00	0	NA	NA	0
EPPK1	epiplakin 1	1,01	0,01	0,94	0,99	0	-1,07	-0,10	0,55	1,00	0	NA	NA	0
NAP1L1	nucleosome assembly protein 1-like 1	1,01	0,01	0,95	0,99	0	-1,03	-0,04	0,74	1,00	0	NA	NA	0
SLC16A1	solute carrier family 16, member 1 (monocarboxylic acid transporter 1)	1,01	0,01	0,93	0,98	0	1,03	0,05	0,78	1,00	0	-1,30	-0,37	0
EMP2	epithelial membrane protein 2	1,01	0,01	0,93	0,98	0	-1,27	-0,34	0,02	0,92	0	NA	NA	0
BSPRY	B-box and SPRY domain containing	1,01	0,01	0,94	0,99	0	1,26	0,33	0,31	1,00	0	NA	NA	0
FECH	ferrochelatase	1,01	0,01	0,96	0,99	0	1,20	0,27	0,34	1,00	0	-1,14	-0,19	0
ELOVL5	ELOVL family member 5, elongation of long chain fatty acids (FEN1/Elo2, SUR4/Elo3-like, yeast)	1,01	0,01	0,94	0,98	0	-1,09	-0,12	0,24	1,00	0	NA	NA	0
TSPYL4	TSPY-like 4	1,01	0,01	0,96	0,99	0	-1,13	-0,18	0,48	1,00	0	-1,14	-0,18	0
05-mar	membrane-associated ring finger (C3HC4) 5	1,01	0,01	0,95	0,99	0	1,15	0,21	0,18	1,00	0	NA	NA	0
CDH11	cadherin 11, type 2, OB-cadherin (osteoblast)	1,01	0,01	0,97	0,99	0	1,13	0,17	NA	NA	0	-1,10	-0,13	0
NBN	nibrin	1,01	0,01	0,95	0,99	0	-1,24	-0,31	0,08	1,00	0	-1,25	-0,32	0
PFKM	phosphofructokinase, muscle	1,01	0,01	0,95	0,99	0	-1,04	-0,06	0,61	1,00	0	NA	NA	0
PTAFR	platelet-activating factor receptor	1,01	0,01	0,96	0,99	0	1,09	0,13	0,76	1,00	0	NA	NA	0
FADS1	fatty acid desaturase 1	1,01	0,01	0,97	0,99	0	1,12	0,17	0,42	1,00	0	NA	NA	0
CISH	cytokine inducible SH2-containing protein	1,01	0,01	0,95	0,99	0	1,06	0,08	0,34	1,00	0	NA	NA	0
CAV2	caveolin 2	1,01	0,01	0,96	0,99	0	1,06	0,08	0,56	1,00	0	-1,15	-0,20	0
MAT2A	methionine adenosyltransferase II, alpha	1,01	0,01	0,93	0,98	0	1,04	0,05	0,57	1,00	0	NA	NA	0
MED22	mediator complex subunit 22	1,01	0,01	0,97	0,99	0	-1,04	-0,05	0,59	1,00	0	1,22	0,29	0
HK2	hexokinase 2	1,01	0,01	0,97	1,00	0	-1,11	-0,15	0,71	1,00	0	NA	NA	0
HK1	hexokinase 1	1,01	0,01	0,94	0,98	0	-1,07	-0,10	0,24	1,00	0	NA	NA	0
GADD45B	growth arrest and DNA-damage-inducible, beta	1,01	0,01	0,97	1,00	0	1,03	0,04	0,86	1,00	0	NA	NA	0
UBXN4	UBX domain protein 4	1,01	0,01	0,95	0,99	0	1,24	0,31	0,73	1,00	0	-1,22	-0,29	0
NDRG1	N-myc downstream regulated 1	1,01	0,01	0,95	0,99	0	-1,17	-0,22	0,20	1,00	0	NA	NA	0
FAT1	FAT tumor suppressor homolog 1 (Drosophila)	1,01	0,01	0,94	0,99	0	-1,13	-0,17	0,18	1,00	0	NA	NA	0
RBM15	RNA binding motif protein 15	1,01	0,01	0,95	0,99	0	1,08	0,11	0,43	1,00	0	NA	NA	0
VPS13A	vacuolar protein sorting 13 homolog A (S. cerevisiae)	1,01	0,01	0,98	1,00	0	-1,12	-0,17	0,26	1,00	0	-1,14	-0,19	0
SMCR7L	Smith-Magenis syndrome chromosome region, candidate 7-like	1,01	0,01	0,96	0,99	0	1,02	0,03	0,79	1,00	0	NA	NA	0
TGFBR2	transforming growth factor, beta receptor II (70/80kDa)	1,01	0,01	0,96	0,99	0	-1,14	-0,19	0,05	1,00	0	NA	NA	0

TMPO	thymopoietin	1,01	0,01	0,96	0,99	0	1,09	0,13	0,30	1,00	0	NA	NA	0
RPL37A	ribosomal protein L37a	1,01	0,01	0,98	1,00	0	1,11	0,16	0,55	1,00	0	NA	NA	0
CORO1B	coronin, actin binding protein, 1B	1,01	0,01	0,98	1,00	0	1,00	0,00	0,98	1,00	0	NA	NA	0
CPNE1	copine I	1,01	0,01	0,96	0,99	0	1,24	0,32	0,26	1,00	0	NA	NA	0
BCAT2	branched chain amino-acid transaminase 2, mitochondrial	1,01	0,01	0,97	1,00	0	-1,12	-0,16	0,46	1,00	0	1,01	0,01	0
ALDH6A1	aldehyde dehydrogenase 6 family, member A1	1,01	0,01	0,97	0,99	0	-1,19	-0,25	0,28	1,00	0	NA	NA	0
RASAL2	RAS protein activator like 2	1,01	0,01	0,98	1,00	0	-1,01	-0,01	0,95	1,00	0	NA	NA	0
PDXK	pyridoxal (pyridoxine, vitamin B6) kinase	1,01	0,01	0,97	0,99	0	-1,20	-0,26	0,03	0,97	0	NA	NA	0
PGK1	phosphoglycerate kinase 1	1,01	0,01	0,97	0,99	0	1,04	0,06	0,53	1,00	0	NA	NA	0
NA	NA	1,01	0,01	0,97	0,99	0	-1,23	-0,30	0,25	1,00	0	NA	NA	0
STX19	syntaxin 19	1,01	0,01	0,97	0,99	0	NA	NA	NA	NA	0	NA	NA	0
STXBP6	syntaxin binding protein 6 (amisyn)	1,01	0,01	0,99	1,00	0	1,05	0,07	0,76	1,00	0	-1,35	-0,43	0
PCNX	pecanex homolog (Drosophila)	1,01	0,01	0,98	1,00	0	-1,21	-0,28	0,27	1,00	0	-1,30	-0,38	0
HERC2	hect domain and RLD 2	1,00	0,01	0,96	0,99	0	-1,20	-0,26	0,21	1,00	0	NA	NA	0
LPGAT1	lysophosphatidylglycerol acyltransferase 1	1,00	0,01	0,97	1,00	0	-1,06	-0,09	0,45	1,00	0	NA	NA	0
POLR1C	polymerase (RNA) I polypeptide C, 30kDa	1,00	0,01	0,97	1,00	0	1,00	0,01	0,97	1,00	0	NA	NA	0
ACTR3B	ARP3 actin-related protein 3 homolog B (yeast)	1,00	0,01	0,97	1,00	0	-1,11	-0,15	0,26	1,00	0	0,00	0,00	0
PRRC1	proline-rich coiled-coil 1	1,00	0,01	0,97	1,00	0	1,00	0,00	1,00	1,00	0	-1,32	-0,39	0
HCG26	HLA complex group 26 (non-protein coding)	1,00	0,01	0,99	1,00	0	-1,05	-0,07	NA	NA	0	NA	NA	0
CHEK1	CHK1 checkpoint homolog (S. pombe)	1,00	0,01	0,99	1,00	0	-1,17	-0,22	0,25	1,00	0	NA	NA	0
INTS6	integrator complex subunit 6	1,00	0,01	0,98	1,00	0	1,06	0,09	0,55	1,00	0	-1,39	-0,47	0
SLC25A22	solute carrier family 25 (mitochondrial carrier: glutamate), member 22	1,00	0,01	0,98	1,00	0	1,36	0,44	0,01	0,65	0	1,42	0,51	0
GPR126	G protein-coupled receptor 126	1,00	0,01	0,98	1,00	0	-1,10	-0,14	0,35	1,00	0	-1,32	-0,39	0
RPGRIP1L	RPGRIP1-like	1,00	0,01	0,98	1,00	0	-1,02	-0,03	0,92	1,00	0	-1,45	-0,54	0
IPO5	importin 5	1,00	0,01	0,98	1,00	0	1,11	0,14	0,37	1,00	0	NA	NA	0
TBC1D16	TBC1 domain family, member 16	1,00	0,00	0,98	1,00	0	-1,06	-0,09	0,56	1,00	0	NA	NA	0
SLC2A3	solute carrier family 2 (facilitated glucose transporter), member 3	1,00	0,00	0,98	1,00	0	-2,26	-1,18	0,01	0,51	0	NA	NA	0
CCNE2	cyclin E2	1,00	0,00	0,99	1,00	0	-1,07	-0,10	0,59	1,00	0	NA	NA	0
MMP1	matrix metallopeptidase 1 (interstitial collagenase)	1,00	0,00	0,97	1,00	0	3,00	1,59	0,01	0,63	0	NA	NA	0
CA12	carbonic anhydrase XII	1,00	0,00	0,98	1,00	0	-1,00	0,00	0,99	1,00	0	1,35	0,43	0
NA	NA	1,00	0,00	0,98	1,00	0	-1,14	-0,19	0,66	1,00	0	NA	NA	0
CASP10	caspase 10, apoptosis-related cysteine peptidase	1,00	0,00	0,99	1,00	0	1,44	0,53	0,04	1,00	0	0,00	0,00	0
SLC25A17	solute carrier family 25 (mitochondrial carrier; peroxisomal membrane protein, 34kDa), member 17	1,00	0,00	0,99	1,00	0	1,26	0,33	0,20	1,00	0	NA	NA	0
HLA-DMB	major histocompatibility complex, class II, DM beta	1,00	0,00	1,00	1,00	0	-1,05	-0,07	0,71	1,00	0	-1,01	-0,02	0
ZFR	zinc finger RNA binding protein	1,00	0,00	0,99	1,00	0	1,08	0,11	0,92	1,00	0	-1,28	-0,36	0
MICAL3	microtubule associated monoxygenase, calponin and LIM domain containing 3	1,00	0,00	0,99	1,00	0	-1,14	-0,19	0,34	1,00	0	NA	NA	0
CDK4	cyclin-dependent kinase 4	1,00	0,00	0,99	1,00	0	-1,05	-0,07	0,55	1,00	0	NA	NA	0
NA	NA	1,00	0,00	0,99	1,00	0	-1,11	-0,15	0,44	1,00	0	NA	NA	0
PRKAA1	protein kinase, AMP-activated, alpha 1 catalytic subunit	1,00	0,00	0,99	1,00	0	-1,29	-0,37	0,29	1,00	0	-1,28	-0,36	0
SGCB	sarcoglycan, beta (43kDa dystrophin-associated glycoprotein)	1,00	0,00	1,00	1,00	0	-1,07	-0,10	NA	NA	0	-1,37	-0,45	0
CXCL11	chemokine (C-X-C motif) ligand 11	1,00	0,00	1,00	1,00	0	-1,01	-0,02	0,95	1,00	0	0,00	0,01	0
INPP5D	inositol polyphosphate-5-phosphatase, 145kDa	1,00	0,00	1,00	1,00	0	-1,70	-0,77	0,01	0,51	0	NA	NA	0
CIITA	class II, major histocompatibility complex, transactivator	1,00	0,00	1,00	1,00	0	-1,00	-0,01	0,99	1,00	0	1,01	0,01	0
TOPORS	topoisomerase I binding, arginine/serine-rich	1,00	0,00	1,00	1,00	0	-1,05	-0,07	0,51	1,00	0	NA	NA	0
RHOQ	ras homolog gene family, member Q	1,00	0,00	1,00	1,00	0	-1,01	-0,01	0,95	1,00	0	-1,06	-0,09	0
HLA-DPA1	major histocompatibility complex, class II, DP alpha 1	1,00	0,00	1,00	1,00	0	-1,00	0,00	1,00	1,00	0	1,01	0,02	0
HLA-DPA1	major histocompatibility complex, class II, DP alpha 1	1,00	0,00	1,00	1,00	0	-1,01	-0,01	0,94	1,00	0	1,01	0,01	0
ACSL5	acyl-CoA synthetase long-chain family member 5	1,00	0,00	1,00	1,00	0	-1,02	-0,04	0,78	1,00	0	1,01	0,01	0
DOK4	docking protein 4	1,00	0,00	1,00	1,00	0	-1,11	-0,15	0,45	1,00	0	NA	NA	0
C17orf70	chromosome 17 open reading frame 70	1,00	0,00	1,00	1,00	0	1,16	0,22	0,20	1,00	0	1,09	0,12	0
MMP25	matrix metallopeptidase 25	1,00	0,00	1,00	1,00	0	-1,01	-0,01	0,99	1,00	0	NA	NA	0
HLA-DRA	major histocompatibility complex, class II, DR alpha	1,00	0,00	1,00	1,00	0	-1,01	-0,01	0,90	1,00	0	0,00	0,01	0
CXCL11	chemokine (C-X-C motif) ligand 11	1,00	0,00	1,00	1,00	0	-1,02	-0,02	0,92	1,00	0	1,01	0,01	0
NA	NA	1,00	0,00	1,00	1,00	0	NA	NA	NA	NA	0	NA	NA	0
FSTL4	follistatin-like 4	1,00	0,00	1,00	1,00	0	NA	NA	NA	NA	0	NA	NA	0
HLA-DRA	major histocompatibility complex, class II, DR alpha	1,00	0,00	1,00	1,00	0	-1,01	-0,01	0,89	1,00	0	0,00	0,00	0
IDO1	indoleamine 2,3-dioxygenase 1	1,00	0,00	1,00	1,00	0	-1,00	0,00	0,99	1,00	0	0,00	0,01	0
GBP4	guanylate binding protein 4	1,00	0,00	1,00	1,00	0	NA	NA	NA	NA	0	NA	NA	0
CXCL9	chemokine (C-X-C motif) ligand 9	1,00	0,00	1,00	1,00	0	-1,01	-0,02	0,81	1,00	0	0,00	0,00	0
GBP5	guanylate binding protein 5	1,00	0,00	1,00	1,00	0	NA	NA	NA	NA	0	NA	NA	0
IL12RB1	interleukin 12 receptor, beta 1	1,00	0,00	1,00	1,00	0	NA	NA	NA	NA	0	NA	NA	0
CD74	CD74 molecule, major histocompatibility complex, class II invariant chain	1,00	0,00	1,00	1,00	0	NA	NA	NA	NA	0	NA	NA	0
GPC1	glypican 1	1,00	0,00	1,00	1,00	0	-1,10	-0,14	0,65	1,00	0	NA	NA	0
PLEKHM2	pleckstrin homology domain containing, family M (with RUN domain) member 2	1,00	0,00	1,00	1,00	0	1,17	0,23	0,13	1,00	0	NA	NA	0
AZGP1	alpha-2-glycoprotein 1, zinc-binding	1,00	0,00	1,00	1,00	0	1,18	0,24	0,71	1,00	0	NA	NA	0
CCL5	chemokine (C-C motif) ligand 5	1,00	0,00	1,00	1,00	0	-2,30	-1,20	0,04	1,00	0	NA	NA	0
CD58	CD58 molecule	1,00	0,00	1,00	1,00	0	1,12	0,16	0,23	1,00	0	1,01	0,01	0
SECISBP2L	SECIS binding protein 2-like	1,00	0,00	1,00	1,00	0	1,12	0,17	0,35	1,00	0	NA	NA	0
EPM2AIP1	EPM2A (laforin) interacting protein 1	1,00	0,00	1,00	1,00	0	-1,08	-0,11	0,56	1,00	0	-1,08	-0,11	0
RAB27A	RAB27A, member RAS oncogene family	1,00	0,00	0,99	1,00	0	1,12	0,16	0,24	1,00	0	NA	NA	0
NA	NA	1,00	0,00	0,99	1,00	0	NA	NA	NA	NA	0	NA	NA	0
NAB1	NGFI-A binding protein 1 (EGR1 binding protein 1)	1,00	0,00	0,99	1,00	0	1,06	0,09	0,58	1,00	0	NA	NA	0
MED15	mediator complex subunit 15	1,00	0,00	1,00	1,00	0	-1,09	-0,12	0,37	1,00	0	NA	NA	0
NID1	nidogen 1	1,00	0,00	0,99	1,00	0	-1,01	-0,01	NA	NA	0	1,10	0,14	0
BCLAF1	BCL2-associated transcription factor 1	1,00	0,00	0,99	1,00	0	-1,14	-0,19	0,69	1,00	0	-1,28	-0,36	0
IGSF3	immunoglobulin superfamily, member 3	1,00	0,00	0,99	1,00	0	-1,08	-0,11	0,40	1,00	0	NA	NA	0
DSN1	DSN1, MIND kinetochore complex component, homolog (S. cerevisiae)	1,00	0,00	0,98	1,00	0	-1,10	-0,14	0,65	1,00	0	NA	NA	0
NPAS2	neuronal PAS domain protein 2	1,00	0,00	0,98	1,00	0	-1,06	-0,08	0,33	1,00	0	1,11	0,15	0
DOM3Z	dom-3 homolog Z (C. elegans)	1,00	0,00	0,98	1,00	0	-1,06	-0,09	0,75	1,00	0	NA	NA	0
SIRT1	sirtuin 1	1,00	0,00	0,98	1,00	0	1,36	0,44	0,02	0,91	0	-1,20	-0,27	0
STOML1	stomatin (EPB72)-like 1	1,00	0,00	0,99	1,00	0	1,73	0,79	0,00	0,44	0	NA	NA	0
MORC3	MORC family CW-type zinc finger 3	1,00	0,00	0,98	1,00	0	1,12	0,16	0,17	1,00	0	NA	NA	0
ICAM1	intercellular adhesion molecule 1	1,00	0,00	0,98	1,00	0	-1,01	-0,02	0,96	1,00	0	-1,06	-0,08	0
SOX4	SRY (sex determining region Y)-box 4	1,00	0,00	0,99	1,00	0	1,08	0,11	0,77	1,00	0	NA	NA	0
ZNF354A	zinc finger protein 354A	1,00	0,00	0,98	1,00	0	1,05	0,07	0,81	1,00	0	NA	NA	0
PBLD	phenazine biosynthesis-like protein domain containing	1,00	0,00	0,98	1,00	0	-1,28	-0,35	0,17	1,00	0	-1,41	-0,49	0
APPBP2	amyloid beta precursor protein (cytoplasmic tail) binding protein 2	1,00	0,00	0,98	1,00	0	1,30	0,37	0,26	1,00	0	NA	NA	0
ZFYVE26	zinc finger, FYVE domain containing 26	1,00	0,00	0,98	1,00	0	1,13	0,18	0,47	1,00	0	0,00	0,01	0
HS3ST3A1	heparan sulfate (glucosamine) 3-O-sulfotransferase 3A1	1,00	0,00	0,98	1,00	0	-1,02	-0,02	0,95	1,00	0	-1,35	-0,42	0
SRPK2	SRSF protein kinase 2	1,00	-0,01	0,98	1,00	0	-1,27	-0,35	0,18	1,00	0	-1,10	-0,14	0
MYO6	myosin VI	1,00	-0,01	0,98	1,00	0	1,09	0,12	0,33	1,00	0	-1,30	-0,37	0
PML	promyelocytic leukemia	1,00	-0,01	0,99	1,00	0	1,04	0,06	0,58	1,00	0	-1,04	-0,06	0
MAP3K7	mitogen-activated protein kinase kinase kinase 7	1,00	-0,01	0,97	1,00	0	-1,17	-0,23	0,07	1,00	0	-1,19	-0,25	0
OTUD4	OTU domain containing 4	1,00	-0,01	0,97	1,00	0	-1,01	-0,01	0,92	1,00	0	1,05	0,07	0
ZMYND11	zinc finger, MYND domain containing 11	1,00	-0,01	0,96	0,99	0	1,02	0,02	0,87	1,00	0	-1,19	-0,25	0
SUN2	Sad1 and UNC84 domain containing 2	1,00	-0,01	0,98	1,00	0	-1,02	-0,03	0,74	1,00	0	0,00	0,00	0
TM2D1	TM2 domain containing 1	1,00	-0,01	0,98	1,00	0	-1,21	-0,28	0,11	1,00	0	NA	NA	0
TMEM194A	transmembrane protein 194A	1,00	-0,01	0,97	0,99	0	1,01	0,01	0,96	1,00	0	NA	NA	0
MCAT	malonyl CoA:ACP acyltransferase (mitochondrial)	0,99	-0,01	0,98	1,00	0	1,10	0,14	0,32	1,00	0	NA	NA	0
TCERG1	transcription elongation regulator 1	0,99	-0,01	0,97	0,99	0	-1,05	-0,07	0,60	1,00	0	NA	NA	0
ZNF365	zinc finger protein 365	0,99	-0,01	0,98	1,00	0	-1,15	-0,20	0,58	1,00	0	-1,22	-0,28	0
PTGES	prostaglandin E synthase	0,99	-0,01	0,95	0,99	0	-1,19	-0,25	0,55	1,00	0	NA	NA	0
BIN1	bridging integrator 1	0,99	-0,01	0,96	0,99	0	1,51	0,60	0,00	0,31	0	1,37	0,45	0
EIF4B	eukaryotic translation initiation factor 4B	0,99	-0,01	0,95	0,99	0	-1,15	-0,20	0,05	1,00	0	NA	NA	0
PANK3	pantothenate kinase 3	0,99	-0,01	0,98	1,00	0	1,01	0,01	0,90	1,00	0	-1,08	-0,11	0
KLHDC10	kelch domain containing 10	0,99	-0,01	0,97	0,99	0	1,34	0,43	0,08	1,00	0	-1,20	-0,27	0
BAZ1B	bromodomain adjacent to zinc finger domain, 1B	0,99	-0,01	0,96	0,99	0	-1,05	-0,07	0,58	1,00	0	NA	NA	0
MRPL9	mitochondrial ribosomal protein L9	0,99	-0,01	0,95	0,99	0	-1,10	-0,13	0,23	1,00	0	NA	NA	0
FHL1	four and a half LIM domains 1	0,99	-0,01	0,97	1,00	0	-1,06	-0,09	0,59	1,00	0	-1,01	-0,02	0
COQ6	coenzyme Q6 homolog, monooxygenase (S. cerevisiae)	0,99	-0,01	0,96	0,99	0	-1,06	-0,08	0,55	1,00	0	-1,37	-0,46	0
RALGAPA1	Ral GTPase activating protein, alpha subunit 1 (catalytic)	0,99	-0,01	0,95	0,99	0	-1,04	-0,05	0,78	1,00	0	NA	NA	0
CUL5	cullin 5	0,99	-0,01	0,96	0,99	0	-1,05	-0,07	0,75	1,00	0	-1,92	-0,94	0
SUPT16H	suppressor of Ty 16 homolog (S. cerevisiae)	0,99	-0,01	0,94	0,98	0	-1,05	-0,08	0,38	1,00	0	NA	NA	0
RFC5	replication factor C (activator 1) 5, 36.5kDa	0,99	-0,01	0,94	0,98	0	1,00	0,00	0,98	1,00	0	NA	NA	0
APIP	APAF1 interacting protein	0,99	-0,01	0,96	0,99	0	1,07	0,10	0,56	1,00	0	-1,16	-0,22	0
PHF20	PHD finger protein 20	0,99	-0,01	0,95	0,99	0	1,01	0,01	0,94	1,00	0	NA	NA	0
POLM	polymerase (DNA directed), mu	0,99	-0,01	0,97	1,00	0	-1,01	-0,01	NA	NA	0	1,06	0,08	0
SLC5A3	solute carrier family 5 (sodium/myo-inositol cotransporter), member 3	0,99	-0,01	0,96	0,99	0	-1,07	-0,10	0,61	1,00	0	1,20	0,26	0
THBS1	thrombospondin 1	0,99	-0,01	0,98	1,00	0	-1,59	-0,67	0,51	1,00	0	NA	NA	0
ARFGAP3	ADP-ribosylation factor GTPase activating protein 3	0,99	-0,01	0,94	0,99	0	-1,07	-0,09	0,51	1,00	0	NA	NA	0
ZNF45	zinc finger protein 45	0,99	-0,01	0,96	0,99	0	-1,10	-0,13	0,21	1,00	0	-1,45	-0,53	0
MSX1	msh homeobox 1	0,99	-0,01	0,95	0,99	0	-1,00	-0,01	NA	NA	0	-1,14	-0,18	0
ZMIZ2	zinc finger, MIZ-type containing 2	0,99	-0,01	0,94	0,98	0	1,05	0,07	0,65	1,00	0	NA	NA	0
POP7	processing of precursor 7, ribonuclease P/MRP subunit (S. cerevisiae)	0,99	-0,01	0,95	0,99	0	1,01	0,02	0,91	1,00	0	NA	NA	0
NT5DC3	5'-nucleotidase domain containing 3	0,99	-0,01	0,97	0,99	0	-1,15	-0,20	0,49	1,00	0	NA	NA	0
CYLD	cylindromatosis (turban tumor syndrome)	0,99	-0,01	0,95	0,99	0	1,12	0,16	0,48	1,00	0	-1,22	-0,29	0
NUBPL	nucleotide binding protein-like	0,99	-0,01	0,93	0,98	0	-1,19	-0,25	0,19	1,00	0	NA	NA	0
NFRKB	nuclear factor related to kappaB binding protein	0,99	-0,01	0,92	0,98	0	-1,05	-0,07	0,72	1,00	0	NA	NA	0
CEP170	centrosomal protein 170kDa	0,99	-0,01	0,95	0,99	0	-1,08	-0,11	0,46	1,00	0	NA	NA	0
MPP6	membrane protein, palmitoylated 6 (MAGUK p55 subfamily member 6)	0,99	-0,01	0,96	0,99	0	1,14	0,19	0,19	1,00	0	-1,37	-0,45	0
RAB25	RAB25, member RAS oncogene family	0,99	-0,01	0,93	0,98	0	1,09	0,13	0,42	1,00	0	NA	NA	0
NA	NA	0,99	-0,01	0,97	1,00	0	-1,49	-0,57	0,07	1,00	0	NA	NA	0
SLC48A1	solute carrier family 48 (heme transporter), member 1	0,99	-0,01	0,92	0,98	0	-1,02	-0,02	0,85	1,00	0	NA	NA	0
SNX11	sorting nexin 11	0,99	-0,01	0,93	0,98	0	1,24	0,31	0,04	1,00	0	NA	NA	0
C12orf4	chromosome 12 open reading frame 4	0,99	-0,02	0,95	0,99	0	1,13	0,18	0,58	1,00	0	NA	NA	0
LGALS3BP	lectin, galactoside-binding, soluble, 3 binding protein	0,99	-0,02	0,98	1,00	0	-1,24	-0,32	0,19	1,00	0	-1,08	-0,10	0
NINJ1	ninjurin 1	0,99	-0,02	0,95	0,99	0	-1,00	0,00	1,00	1,00	0	NA	NA	0

ZNF276	zinc finger protein 276	0,99	-0,02	0,91	0,98	0	-1,04	-0,06	0,81	1,00	0	NA	NA	0
CCND2	cyclin D2	0,99	-0,02	0,92	0,98	0	-1,00	-0,01	0,94	1,00	0	-1,22	-0,28	0
HLA-DRB6	major histocompatibility complex, class II, DR beta 6 (pseudogene)	0,99	-0,02	0,96	0,99	0	-1,00	-0,01	0,99	1,00	0	1,01	0,01	0
NKRF	NFKB repressing factor	0,99	-0,02	0,90	0,97	0	1,08	0,11	0,46	1,00	0	-1,04	-0,06	0
CPD	carboxypeptidase D	0,99	-0,02	0,95	0,99	0	-1,07	-0,09	0,78	1,00	0	NA	NA	0
CTDSPL	CTD (carboxy-terminal domain, RNA polymerase II, polypeptide A) small phosphatase-like	0,99	-0,02	0,95	0,99	0	-1,10	-0,14	0,19	1,00	0	NA	NA	0
EIF2C1	eukaryotic translation initiation factor 2C, 1	0,99	-0,02	0,91	0,98	0	1,00	0,01	0,98	1,00	0	-1,19	-0,25	0
LMCD1	LIM and cysteine-rich domains 1	0,99	-0,02	0,97	0,99	0	-1,00	0,00	NA	NA	0	-1,41	-0,50	0
LOC388796	hypothetical LOC388796	0,99	-0,02	0,89	0,97	0	-1,01	-0,01	0,95	1,00	0	NA	NA	0
LIN7C	lin-7 homolog C (C. elegans)	0,99	-0,02	0,93	0,98	0	-1,21	-0,28	0,37	1,00	0	-1,52	-0,60	0
PSME2	proteasome (prosome, macropain) activator subunit 2 (PA28 beta)	0,99	-0,02	0,91	0,98	0	-1,12	-0,16	0,29	1,00	0	-1,16	-0,21	0
PPWD1	peptidylprolyl isomerase domain and WD repeat containing 1	0,99	-0,02	0,94	0,98	0	-1,06	-0,09	0,54	1,00	0	NA	NA	0
SLC2A3	solute carrier family 2 (facilitated glucose transporter), member 3	0,99	-0,02	0,94	0,98	0	-2,38	-1,25	0,00	0,20	0	NA	NA	0
HLA-F	major histocompatibility complex, class I, F	0,99	-0,02	0,93	0,98	0	-1,13	-0,18	0,26	1,00	0	1,04	0,06	0
DEXI	Dexi homolog (mouse)	0,99	-0,02	0,93	0,98	0	-1,04	-0,06	0,71	1,00	0	1,08	0,11	0
CTSB	cathepsin B	0,99	-0,02	0,89	0,97	0	1,06	0,08	0,60	1,00	0	NA	NA	0
ELF4	E74-like factor 4 (ets domain transcription factor)	0,99	-0,02	0,92	0,98	0	-1,05	-0,06	0,49	1,00	0	NA	NA	0
DCLRE1B	DNA cross-link repair 1B	0,99	-0,02	0,89	0,97	0	1,46	0,55	0,01	0,55	0	NA	NA	0
HGSNAT	heparan-alpha-glucosaminide N-acetyltransferase	0,99	-0,02	0,93	0,98	0	1,06	0,09	0,59	1,00	0	1,55	0,63	0
RABGGTB	Rab geranylgeranyltransferase, beta subunit	0,99	-0,02	0,94	0,98	0	1,16	0,22	0,09	1,00	0	-1,08	-0,11	0
CDKN1A	cyclin-dependent kinase inhibitor 1A (p21, Cip1)	0,99	-0,02	0,90	0,97	0	1,11	0,15	0,37	1,00	0	NA	NA	0
PTHLH	parathyroid hormone-like hormone	0,99	-0,02	0,98	1,00	0	1,06	0,08	0,70	1,00	0	NA	NA	0
C12orf52	chromosome 12 open reading frame 52	0,99	-0,02	0,93	0,98	0	1,19	0,25	0,15	1,00	0	-1,08	-0,11	0
MMAA	methylmalonic aciduria (cobalamin deficiency) cblA type	0,99	-0,02	0,93	0,98	0	NA	NA	NA	NA	0	NA	NA	0
IDE	insulin-degrading enzyme	0,99	-0,02	0,91	0,98	0	-1,10	-0,14	0,44	1,00	0	-1,16	-0,21	0
HADH	hydroxyacyl-CoA dehydrogenase	0,99	-0,02	0,91	0,98	0	1,39	0,48	0,01	0,47	0	NA	NA	0
PARD3	par-3 partitioning defective 3 homolog (C. elegans)	0,99	-0,02	0,95	0,99	0	-1,04	-0,05	0,72	1,00	0	-1,03	-0,05	0
CPOX	coproporphyrinogen oxidase	0,99	-0,02	0,92	0,98	0	1,02	0,02	0,86	1,00	0	NA	NA	0
POLR1C	polymerase (RNA) I polypeptide C, 30kDa	0,99	-0,02	0,92	0,98	0	1,15	0,20	0,45	1,00	0	-1,43	-0,52	0
ACSL5	acyl-CoA synthetase long-chain family member 5	0,99	-0,02	0,90	0,97	0	NA	NA	NA	NA	0	NA	NA	0
CYLD	cylindromatosis (turban tumor syndrome)	0,99	-0,02	0,93	0,98	0	1,26	0,33	0,12	1,00	0	NA	NA	0
EMP3	epithelial membrane protein 3	0,98	-0,02	0,90	0,97	0	1,07	0,10	0,77	1,00	0	NA	NA	0
NA	NA	0,98	-0,02	0,91	0,98	0	NA	NA	NA	NA	0	NA	NA	0
NPAS2	neuronal PAS domain protein 2	0,98	-0,02	0,89	0,97	0	-1,14	-0,19	0,17	1,00	0	1,05	0,08	0
PHIP	pleckstrin homology domain interacting protein	0,98	-0,02	0,93	0,98	0	-1,02	-0,03	0,86	1,00	0	NA	NA	0
NCKIPSD	NCK interacting protein with SH3 domain	0,98	-0,02	0,90	0,97	0	-1,26	-0,33	0,09	1,00	0	NA	NA	0
TMEM185B	transmembrane protein 185B (pseudogene)	0,98	-0,02	0,92	0,98	0	-1,13	-0,18	0,23	1,00	0	NA	NA	0
SUPT7L	suppressor of Ty 7 (S. cerevisiae)-like	0,98	-0,02	0,89	0,97	0	1,05	0,07	0,77	1,00	0	-1,19	-0,25	0
ZNF22	zinc finger protein 22 (KOX 15)	0,98	-0,02	0,91	0,98	0	-1,02	-0,03	0,85	1,00	0	NA	NA	0
FLAD1	FAD1 flavin adenine dinucleotide synthetase homolog (S. cerevisiae)	0,98	-0,02	0,90	0,97	0	1,02	0,03	0,86	1,00	0	NA	NA	0
PARD3	par-3 partitioning defective 3 homolog (C. elegans)	0,98	-0,02	0,95	0,99	0	-1,02	-0,03	0,77	1,00	0	-1,09	-0,12	0
LPAR1	lysophosphatidic acid receptor 1	0,98	-0,02	0,91	0,98	0	-1,09	-0,12	0,60	1,00	0	-1,05	-0,07	0
CD74	CD74 molecule, major histocompatibility complex, class II invariant chain	0,98	-0,02	0,96	0,99	0	NA	NA	NA	NA	0	NA	NA	0
RHOG	ras homolog gene family, member G (rho G)	0,98	-0,02	0,89	0,97	0	1,11	0,16	0,13	1,00	0	NA	NA	0
STAT6	signal transducer and activator of transcription 6, interleukin-4 induced	0,98	-0,02	0,84	0,96	0	1,01	0,01	0,90	1,00	0	1,12	0,17	0
ISYNA1	inositol-3-phosphate synthase 1	0,98	-0,02	0,93	0,98	0	-1,43	-0,52	0,02	0,94	0	NA	NA	0
BCAP29	B-cell receptor-associated protein 29	0,98	-0,02	0,87	0,97	0	-1,01	-0,01	0,94	1,00	0	NA	NA	0
POLD3	polymerase (DNA-directed), delta 3, accessory subunit	0,98	-0,03	0,86	0,96	0	1,03	0,04	0,81	1,00	0	NA	NA	0
XPNPEP1	X-prolyl aminopeptidase (aminopeptidase P) 1, soluble	0,98	-0,03	0,89	0,97	0	-1,06	-0,08	0,72	1,00	0	-1,03	-0,05	0
NA	NA	0,98	-0,03	0,91	0,98	0	-1,02	-0,03	0,90	1,00	0	-1,15	-0,20	0
TIMM10	translocase of inner mitochondrial membrane 10 homolog (yeast)	0,98	-0,03	0,93	0,98	0	1,19	0,25	0,45	1,00	0	NA	NA	0
DYNC1H1	dynein, cytoplasmic 1, heavy chain 1	0,98	-0,03	0,91	0,98	0	-1,05	-0,08	0,54	1,00	0	1,29	0,36	0
TMEM48	transmembrane protein 48	0,98	-0,03	0,90	0,97	0	-1,04	-0,06	0,75	1,00	0	-1,02	-0,03	0
TSN	translin	0,98	-0,03	0,88	0,97	0	-1,09	-0,13	0,54	1,00	0	NA	NA	0
FAM65A	family with sequence similarity 65, member A	0,98	-0,03	0,91	0,98	0	-1,01	-0,02	0,89	1,00	0	NA	NA	0
AK4	adenylate kinase 4	0,98	-0,03	0,87	0,97	0	-1,12	-0,16	0,59	1,00	0	-1,02	-0,04	0
IMPDH2	IMP (inosine 5'-monophosphate) dehydrogenase 2	0,98	-0,03	0,85	0,96	0	1,01	0,01	0,93	1,00	0	NA	NA	0
ADO	2-aminoethanethiol (cysteamine) dioxygenase	0,98	-0,03	0,84	0,96	0	-1,01	-0,02	0,91	1,00	0	NA	NA	0
BNIP3	BCL2/adenovirus E1B 19kDa interacting protein 3	0,98	-0,03	0,93	0,98	0	1,01	0,02	0,92	1,00	0	NA	NA	0
TSPYL5	TSPY-like 5	0,98	-0,03	0,88	0,97	0	-1,01	-0,01	0,95	1,00	0	NA	NA	0
NOTCH1	notch 1	0,98	-0,03	0,90	0,97	0	1,04	0,05	0,76	1,00	0	NA	NA	0
NA	NA	0,98	-0,03	0,86	0,96	0	-1,03	-0,04	0,89	1,00	0	-1,04	-0,06	0
TPM1	tropomyosin 1 (alpha)	0,98	-0,03	0,87	0,97	0	1,02	0,03	0,74	1,00	0	NA	NA	0
MFAP4	microfibrillar-associated protein 4	0,98	-0,03	0,93	0,98	0	-1,01	-0,01	NA	NA	0	-1,25	-0,32	0
RBM3	RNA binding motif (RNP1, RRM) protein 3	0,98	-0,03	0,87	0,97	0	1,04	0,05	0,75	1,00	0	-1,30	-0,38	0
TMEM30A	transmembrane protein 30A	0,98	-0,03	0,88	0,97	0	1,05	0,07	0,62	1,00	0	-1,06	-0,09	0
USP33	ubiquitin specific peptidase 33	0,98	-0,03	0,82	0,95	0	-1,02	-0,02	NA	NA	0	1,01	0,01	0
SIP1	survival of motor neuron protein interacting protein 1	0,98	-0,03	0,91	0,98	0	1,01	0,02	0,94	1,00	0	NA	NA	0
ALMS1	Alstrom syndrome 1	0,98	-0,03	0,85	0,96	0	-1,02	-0,03	0,92	1,00	0	NA	NA	0
PREPL	prolyl endopeptidase-like	0,98	-0,03	0,90	0,97	0	1,03	0,04	0,80	1,00	0	-1,10	-0,13	0
UBTF	upstream binding transcription factor, RNA polymerase I	0,98	-0,03	0,94	0,98	0	-1,47	-0,55	0,17	1,00	0	NA	NA	0
NOTCH2NL	notch 2 N-terminal like	0,98	-0,03	0,86	0,96	0	-1,18	-0,24	0,35	1,00	0	NA	NA	0
FAM69A	family with sequence similarity 69, member A	0,98	-0,03	0,86	0,96	0	1,36	0,44	0,03	1,00	0	-1,12	-0,16	0
DNMBP	dynamin binding protein	0,98	-0,03	0,85	0,96	0	-1,16	-0,21	0,54	1,00	0	NA	NA	0
CREM	cAMP responsive element modulator	0,98	-0,03	0,89	0,97	0	1,01	0,02	0,98	1,00	0	-1,15	-0,21	0
HINFP	histone H4 transcription factor	0,98	-0,03	0,87	0,97	0	-1,10	-0,14	0,31	1,00	0	NA	NA	0
CSRP1	cysteine and glycine-rich protein 1	0,98	-0,03	0,87	0,97	0	-1,12	-0,16	0,14	1,00	0	NA	NA	0
LPAR1	lysophosphatidic acid receptor 1	0,98	-0,03	0,89	0,97	0	-1,14	-0,19	0,35	1,00	0	NA	NA	0
FBXO11	F-box protein 11	0,98	-0,03	0,81	0,95	0	-1,09	-0,13	0,62	1,00	0	NA	NA	0
CALM3	calmodulin 3 (phosphorylase kinase, delta)	0,98	-0,03	0,88	0,97	0	1,06	0,08	0,43	1,00	0	NA	NA	0
AZGP1	alpha-2-glycoprotein 1, zinc-binding	0,98	-0,03	0,91	0,98	0	-1,15	-0,20	0,72	1,00	0	NA	NA	0
BTN3A1	butyrophilin, subfamily 3, member A1	0,98	-0,03	0,91	0,98	0	-1,01	-0,02	0,96	1,00	0	-1,01	-0,01	0
PDE8A	phosphodiesterase 8A	0,98	-0,03	0,90	0,97	0	1,23	0,30	0,02	0,86	0	NA	NA	0
WTAP	Wilms tumor 1 associated protein	0,98	-0,03	0,85	0,96	0	1,08	0,12	0,60	1,00	0	NA	NA	0
LAPTM4B	lysosomal protein transmembrane 4 beta	0,98	-0,03	0,84	0,96	0	-1,02	-0,02	0,83	1,00	0	NA	NA	0
NOC3L	nucleolar complex associated 3 homolog (S. cerevisiae)	0,98	-0,03	0,87	0,97	0	-1,09	-0,12	0,29	1,00	0	NA	NA	0
FMR1	fragile X mental retardation 1	0,98	-0,03	0,86	0,96	0	-1,01	-0,01	0,94	1,00	0	-1,35	-0,43	0
RACGAP1	Rac GTPase activating protein 1	0,98	-0,03	0,86	0,96	0	1,04	0,05	0,70	1,00	0	NA	NA	0
TACC3	transforming, acidic coiled-coil containing protein 3	0,98	-0,03	0,78	0,94	0	1,03	0,04	0,65	1,00	0	NA	NA	0
API5	apoptosis inhibitor 5	0,98	-0,03	0,87	0,97	0	-1,01	-0,01	0,98	1,00	0	-1,39	-0,47	0
STX7	syntaxin 7	0,98	-0,03	0,81	0,95	0	1,13	0,18	0,21	1,00	0	-1,14	-0,18	0
PYGB	phosphorylase, glycogen; brain	0,98	-0,03	0,97	0,99	0	-1,18	-0,24	0,29	1,00	0	NA	NA	0
KPNA5	karyopherin alpha 5 (importin alpha 6)	0,98	-0,03	0,84	0,96	0	1,03	0,04	0,87	1,00	0	-1,49	-0,58	0
NCAPH2	non-SMC condensin II complex, subunit H2	0,98	-0,03	0,86	0,96	0	NA	NA	NA	NA	0	NA	NA	0
PPP1R13L	protein phosphatase 1, regulatory (inhibitor) subunit 13 like	0,98	-0,03	0,81	0,95	0	-1,05	-0,07	0,71	1,00	0	1,11	0,15	0
DEK	DEK oncogene	0,98	-0,03	0,81	0,95	0	-1,02	-0,04	0,73	1,00	0	NA	NA	0
TRIM33	tripartite motif-containing 33	0,98	-0,03	0,80	0,95	0	-1,02	-0,03	0,77	1,00	0	NA	NA	0
HLA-G	major histocompatibility complex, class I, G	0,98	-0,03	0,89	0,97	0	-1,10	-0,14	0,60	1,00	0	1,01	0,01	0
GPRC5A	G protein-coupled receptor, family C, group 5, member A	0,98	-0,04	0,96	0,99	0	-1,65	-0,73	0,07	1,00	0	NA	NA	0
ARMC9	armadillo repeat containing 9	0,98	-0,04	0,90	0,97	0	-1,23	-0,30	0,10	1,00	0	-1,30	-0,38	0
CALU	calumenin	0,98	-0,04	0,81	0,95	0	1,05	0,07	0,58	1,00	0	NA	NA	0
EP400	E1A binding protein p400	0,98	-0,04	0,85	0,96	0	-1,02	-0,03	0,91	1,00	0	NA	NA	0
GALNT11	UDP-N-acetyl-alpha-D-galactosamine:polypeptide N-acetylgalactosaminyltransferase 11 (GalNAc-T11)	0,98	-0,04	0,83	0,95	0	1,02	0,02	0,91	1,00	0	-1,12	-0,16	0
ARHGAP19	Rho GTPase activating protein 19	0,97	-0,04	0,87	0,97	0	1,06	0,08	0,59	1,00	0	NA	NA	0
NA	NA	0,97	-0,04	0,83	0,95	0	NA	NA	NA	NA	0	NA	NA	0
LGALS1	lectin, galactoside-binding, soluble, 1	0,97	-0,04	0,84	0,96	0	-1,13	-0,18	0,56	1,00	0	NA	NA	0
EXOSC2	exosome component 2	0,97	-0,04	0,82	0,95	0	-1,12	-0,16	0,65	1,00	0	NA	NA	0
SMARCD2	SWI/SNF related, matrix associated, actin dependent regulator of chromatin, subfamily d, member 2	0,97	-0,04	0,81	0,95	0	-1,16	-0,21	0,07	1,00	0	NA	NA	0
MTA1	metastasis associated 1	0,97	-0,04	0,78	0,94	0	-1,07	-0,09	0,36	1,00	0	NA	NA	0
GTPBP3	GTP binding protein 3 (mitochondrial)	0,97	-0,04	0,81	0,95	0	1,07	0,10	0,32	1,00	0	NA	NA	0
CCDC93	coiled-coil domain containing 93	0,97	-0,04	0,86	0,96	0	1,10	0,14	0,30	1,00	0	NA	NA	0
RPA1	replication protein A1, 70kDa	0,97	-0,04	0,80	0,94	0	1,01	0,01	0,90	1,00	0	NA	NA	0
RAB11FIP5	RAB11 family interacting protein 5 (class I)	0,97	-0,04	0,85	0,96	0	1,08	0,11	0,52	1,00	0	NA	NA	0
CTSB	cathepsin B	0,97	-0,04	0,80	0,95	0	1,03	0,05	0,82	1,00	0	NA	NA	0
NA	NA	0,97	-0,04	0,84	0,96	0	-1,07	-0,10	0,63	1,00	0	-1,33	-0,42	0
TLK2	tousled-like kinase 2	0,97	-0,04	0,78	0,94	0	1,03	0,04	0,70	1,00	0	NA	NA	0
PHF15	PHD finger protein 15	0,97	-0,04	0,90	0,98	0	-1,20	-0,26	0,14	1,00	0	1,09	0,12	0
RHBDF1	rhomboid 5 homolog 1 (Drosophila)	0,97	-0,04	0,85	0,96	0	-1,01	-0,02	0,95	1,00	0	NA	NA	0
PRKCSH	protein kinase C substrate 80K-H	0,97	-0,04	0,90	0,97	0	-1,16	-0,22	0,43	1,00	0	2,20	1,14	0
HMGB2	high-mobility group box 2	0,97	-0,04	0,79	0,94	0	-1,01	-0,01	0,93	1,00	0	NA	NA	0
SCMH1	sex comb on midleg homolog 1 (Drosophila)	0,97	-0,04	0,85	0,96	0	-1,14	-0,18	0,40	1,00	0	NA	NA	0
NUB1	negative regulator of ubiquitin-like proteins 1	0,97	-0,04	0,79	0,94	0	NA	NA	NA	NA	0	NA	NA	0
PDS5A	PDS5, regulator of cohesion maintenance, homolog A (S. cerevisiae)	0,97	-0,04	0,86	0,97	0	-1,08	-0,11	0,64	1,00	0	NA	NA	0
C8orf33	chromosome 8 open reading frame 33	0,97	-0,04	0,83	0,96	0	1,09	0,12	0,25	1,00	0	NA	NA	0
NFIB	nuclear factor I/B	0,97	-0,04	0,85	0,96	0	-1,11	-0,14	0,55	1,00	0	-1,03	-0,04	0
NA	NA	0,97	-0,04	0,91	0,98	0	1,08	0,11	0,69	1,00	0	1,27	0,35	0
OXA1L	oxidase (cytochrome c) assembly 1-like	0,97	-0,04	0,80	0,95	0	-1,13	-0,17	0,06	1,00	0	NA	NA	0
TK2	thymidine kinase 2, mitochondrial	0,97	-0,04	0,88	0,97	0	-1,03	-0,04	0,92	1,00	0	NA	NA	0
MLKL	mixed lineage kinase domain-like	0,97	-0,04	0,87	0,97	0	NA	NA	NA	NA	0	NA	NA	0
C10orf57	chromosome 10 open reading frame 57	0,97	-0,04	0,84	0,96	0	-1,03	-0,05	0,64	1,00	0	NA	NA	0
TSPAN13	tetraspanin 13	0,97	-0,04	0,80	0,94	0	1,09	0,12	0,39	1,00	0	-1,02	-0,02	0
DKFZP586I142(	hypothetical protein DKFZp586I1420	0,97	-0,04	0,89	0,97	0	1,00	0,00	1,00	1,00	0	NA	NA	0
DYRK4	dual-specificity tyrosine-(Y)-phosphorylation regulated kinase 4	0,97	-0,04	0,85	0,96	0	-1,07	-0,09	0,67	1,00	0	-1,14	-0,18	0
LAMP2	lysosomal-associated membrane protein 2	0,97	-0,04	0,85	0,96	0	-1,01	-0,01	0,92	1,00	0	NA	NA	0
FYN	FYN oncogene related to SRC, FGR, YES	0,97	-0,04	0,74	0,92	0	-1,27	-0,34	0,29	1,00	0	1,01	0,01	0
PSMA2	proteasome (prosome, macropain) subunit, alpha type, 2	0,97	-0,04	0,83	0,95	0	1,06	0,08	0,67	1,00	0	-1,22	-0,29	0
CRTAP	cartilage associated protein	0,97	-0,04	0,83	0,95	0	-1,21	-0,28	0,13	1,00	0	NA	NA	0

ZBTB20	zinc finger and BTB domain containing 20	0,97	-0,04	0,87	0,97	0	1,01	0,01	NA	NA	0	-1,37	-0,45	0
ZYX	zyxin	0,97	-0,04	0,85	0,96	0	1,03	0,04	0,85	1,00	0	1,58	0,66	0
ZNF146	zinc finger protein 146	0,97	-0,04	0,76	0,93	0	-1,03	-0,04	0,70	1,00	0	NA	NA	0
PARP2	poly (ADP-ribose) polymerase 2	0,97	-0,04	0,79	0,94	0	-1,07	-0,10	0,67	1,00	0	NA	NA	0
NRF1	nuclear respiratory factor 1	0,97	-0,04	0,82	0,95	0	1,05	0,08	0,78	1,00	0	NA	NA	0
ADORA2B	adenosine A2b receptor	0,97	-0,04	0,88	0,97	0	1,06	0,09	0,68	1,00	0	1,49	0,58	0
JUN	jun proto-oncogene	0,97	-0,04	0,78	0,94	0	1,18	0,24	0,05	1,00	0	NA	NA	0
SIP1	survival of motor neuron protein interacting protein 1	0,97	-0,04	0,85	0,96	0	-1,07	-0,10	0,73	1,00	0	NA	NA	0
TGFBR3	transforming growth factor, beta receptor III	0,97	-0,04	0,90	0,97	0	-1,38	-0,47	0,01	0,67	0	NA	NA	0
FLI1	Friend leukemia virus integration 1	0,97	-0,04	0,84	0,96	0	-1,01	-0,01	NA	NA	0	1,03	0,05	0
ZC3H14	zinc finger CCCH-type containing 14	0,97	-0,04	0,77	0,93	0	-1,02	-0,03	0,79	1,00	0	NA	NA	0
MPHOSPH9	M-phase phosphoprotein 9	0,97	-0,04	0,83	0,95	0	1,15	0,21	0,59	1,00	0	-1,22	-0,30	0
IMPDH1	IMP (inosine 5'-monophosphate) dehydrogenase 1	0,97	-0,04	0,73	0,92	0	-1,03	-0,04	0,79	1,00	0	NA	NA	0
CAPN3	calpain 3, (p94)	0,97	-0,04	0,91	0,98	0	-1,01	-0,01	0,98	1,00	0	NA	NA	0
C20orf27	chromosome 20 open reading frame 27	0,97	-0,04	0,80	0,94	0	1,02	0,03	0,81	1,00	0	NA	NA	0
MDN1	MDN1, midasin homolog (yeast)	0,97	-0,05	0,82	0,95	0	-1,02	-0,03	0,92	1,00	0	NA	NA	0
NA	NA	0,97	-0,05	0,86	0,97	0	NA	NA	NA	NA	0	NA	NA	0
TYMS	thymidylate synthetase	0,97	-0,05	0,81	0,95	0	-1,04	-0,05	0,58	1,00	0	NA	NA	0
KDELC1	KDEL (Lys-Asp-Glu-Leu) containing 1	0,97	-0,05	0,82	0,95	0	-1,14	-0,19	0,26	1,00	0	-1,15	-0,21	0
SIP1	survival of motor neuron protein interacting protein 1	0,97	-0,05	0,82	0,95	0	1,10	0,14	0,62	1,00	0	NA	NA	0
WDHD1	WD repeat and HMG-box DNA binding protein 1	0,97	-0,05	0,83	0,95	0	-1,16	-0,21	0,28	1,00	0	NA	NA	0
FAM82A2	family with sequence similarity 82, member A2	0,97	-0,05	0,84	0,96	0	1,23	0,30	0,08	1,00	0	-1,05	-0,07	0
ANP32A	acidic (leucine-rich) nuclear phosphoprotein 32 family, member A	0,97	-0,05	0,80	0,94	0	-1,01	-0,02	0,92	1,00	0	NA	NA	0
RND3	Rho family GTPase 3	0,97	-0,05	0,74	0,92	0	1,33	0,42	0,00	0,44	0	NA	NA	0
THUMPD1	THUMP domain containing 1	0,97	-0,05	0,80	0,94	0	1,16	0,21	0,14	1,00	0	1,17	0,22	0
CRY1	cryptochrome 1 (photolyase-like)	0,97	-0,05	0,89	0,97	0	-1,09	-0,13	0,28	1,00	0	-1,11	-0,16	0
TMED10	transmembrane emp24-like trafficking protein 10 (yeast)	0,97	-0,05	0,76	0,93	0	-1,03	-0,05	0,59	1,00	0	NA	NA	0
MEX3C	mex-3 homolog C (C. elegans)	0,97	-0,05	0,78	0,94	0	-1,16	-0,22	0,07	1,00	0	-1,22	-0,28	0
NPEPPS	aminopeptidase puromycin sensitive	0,97	-0,05	0,82	0,95	0	1,04	0,06	0,60	1,00	0	NA	NA	0
RBCK1	RanBP-type and C3HC4-type zinc finger containing 1	0,97	-0,05	0,76	0,93	0	-1,12	-0,16	0,36	1,00	0	-1,11	-0,15	0
CAMKK2	calcium/calmodulin-dependent protein kinase kinase 2, beta	0,97	-0,05	0,86	0,96	0	1,12	0,17	0,12	1,00	0	1,02	0,02	0
MMP19	matrix metallopeptidase 19	0,97	-0,05	0,85	0,96	0	-1,17	-0,22	0,43	1,00	0	NA	NA	0
ELMO2	engulfment and cell motility 2	0,97	-0,05	0,90	0,97	0	1,11	0,14	0,82	1,00	0	1,32	0,40	0
C20orf3	chromosome 20 open reading frame 3	0,97	-0,05	0,73	0,92	0	-1,10	-0,13	0,30	1,00	0	NA	NA	0
ZNF124	zinc finger protein 124	0,97	-0,05	0,84	0,96	0	-1,01	-0,02	0,96	1,00	0	NA	NA	0
NA	NA	0,97	-0,05	0,77	0,93	0	1,11	0,15	0,54	1,00	0	NA	NA	0
CYB561D2	cytochrome b-561 domain containing 2	0,97	-0,05	0,78	0,94	0	1,18	0,24	0,09	1,00	0	NA	NA	0
ZNF692	zinc finger protein 692	0,97	-0,05	0,82	0,95	0	1,07	0,09	0,59	1,00	0	1,79	0,84	0
GOLGA8B	golgin A8 family, member B	0,97	-0,05	0,86	0,96	0	-1,06	-0,08	0,79	1,00	0	-1,47	-0,56	0
ABCC3	ATP-binding cassette, sub-family C (CFTR/MRP), member 3	0,97	-0,05	0,81	0,95	0	-1,10	-0,13	0,36	1,00	0	NA	NA	0
QTRT1	queuine tRNA-ribosyltransferase 1	0,97	-0,05	0,78	0,94	0	-1,09	-0,12	0,56	1,00	0	NA	NA	0
SOCS5	suppressor of cytokine signaling 5	0,97	-0,05	0,82	0,95	0	1,01	0,01	0,90	1,00	0	NA	NA	0
LMF2	lipase maturation factor 2	0,97	-0,05	0,86	0,96	0	1,09	0,12	0,35	1,00	0	NA	NA	0
HLA-B	major histocompatibility complex, class I, B	0,97	-0,05	0,79	0,94	0	-1,27	-0,34	0,19	1,00	0	1,04	0,06	0
ABCA1	ATP-binding cassette, sub-family A (ABC1), member 1	0,97	-0,05	0,77	0,93	0	1,09	0,12	0,77	1,00	0	NA	NA	0
OTUB1	OTU domain, ubiquitin aldehyde binding 1	0,97	-0,05	0,82	0,95	0	1,17	0,22	0,47	1,00	0	NA	NA	0
NA	NA	0,97	-0,05	0,88	0,97	0	-1,01	-0,02	0,87	1,00	0	1,18	0,24	0
MYNN	myoneurin	0,97	-0,05	0,83	0,95	0	-1,15	-0,20	0,47	1,00	0	NA	NA	0
CSTF2T	cleavage stimulation factor, 3' pre-RNA, subunit 2, 64kDa, tau variant	0,97	-0,05	0,72	0,92	0	-1,12	-0,16	0,60	1,00	0	-1,52	-0,60	0
SORBS2	sorbin and SH3 domain containing 2	0,97	-0,05	0,92	0,98	0	NA	NA	NA	NA	0	NA	NA	0
TGFB2	transforming growth factor, beta 2	0,97	-0,05	0,91	0,98	0	-1,56	-0,64	0,10	1,00	0	1,11	0,15	0
PPAT	phosphoribosyl pyrophosphate amidotransferase	0,97	-0,05	0,76	0,93	0	1,09	0,13	0,74	1,00	0	NA	NA	0
MAPK12	mitogen-activated protein kinase 12	0,97	-0,05	0,72	0,92	0	-1,08	-0,11	0,56	1,00	0	NA	NA	0
LRPPRC	leucine-rich PPR-motif containing	0,97	-0,05	0,71	0,91	0	-1,03	-0,05	0,61	1,00	0	NA	NA	0
MTMR9	myotubularin related protein 9	0,97	-0,05	0,71	0,91	0	1,08	0,11	0,62	1,00	0	NA	NA	0
KARS	lysyl-tRNA synthetase	0,97	-0,05	0,72	0,91	0	-1,04	-0,05	0,48	1,00	0	-1,06	-0,10	0
BTN3A2	butyrophilin, subfamily 3, member A2	0,97	-0,05	0,88	0,97	0	-1,73	-0,79	0,04	1,00	0	-1,35	-0,43	0
RYK	RYK receptor-like tyrosine kinase	0,97	-0,05	0,74	0,93	0	1,16	0,21	0,69	1,00	0	-1,18	-0,24	0
LEF1	lymphoid enhancer-binding factor 1	0,96	-0,05	0,92	0,98	0	1,03	0,04	0,90	1,00	0	NA	NA	0
TPM1	tropomyosin 1 (alpha)	0,96	-0,05	0,87	0,97	0	-1,00	0,00	0,97	1,00	0	1,07	0,10	0
CCDC88A	coiled-coil domain containing 88A	0,96	-0,05	0,89	0,97	0	-1,56	-0,64	0,02	0,94	0	-1,12	-0,17	0
SYNCRIP	synaptotagmin binding, cytoplasmic RNA interacting protein	0,96	-0,05	0,67	0,90	0	-1,09	-0,12	0,49	1,00	0	-1,25	-0,32	0
LMO2	LIM domain only 2 (rhombotin-like 1)	0,96	-0,05	0,93	0,98	0	1,22	0,29	0,24	1,00	0	0,00	0,01	0
RHOD	ras homolog gene family, member D	0,96	-0,05	0,80	0,94	0	-1,06	-0,09	0,45	1,00	0	NA	NA	0
SLC16A4	solute carrier family 16, member 4 (monocarboxylic acid transporter 5)	0,96	-0,05	0,80	0,94	0	-1,02	-0,03	0,94	1,00	0	NA	NA	0
SS18	synovial sarcoma translocation, chromosome 18	0,96	-0,05	0,76	0,93	0	1,26	0,33	0,52	1,00	0	-1,19	-0,26	0
IL13RA1	interleukin 13 receptor, alpha 1	0,96	-0,05	0,70	0,91	0	1,05	0,07	0,44	1,00	0	NA	NA	0
HOOK3	hook homolog 3 (Drosophila)	0,96	-0,05	0,71	0,91	0	NA	NA	NA	NA	0	NA	NA	0
F3	coagulation factor III (thromboplastin, tissue factor)	0,96	-0,05	0,78	0,94	0	1,23	0,29	0,21	1,00	0	-1,19	-0,25	0
DENND5A	DENN/MADD domain containing 5A	0,96	-0,05	0,76	0,93	0	1,04	0,05	0,76	1,00	0	-1,16	-0,21	0
SLC43A1	solute carrier family 43, member 1	0,96	-0,05	0,79	0,94	0	-1,09	-0,13	0,28	1,00	0	-1,08	-0,11	0
KIF5B	kinesin family member 5B	0,96	-0,05	0,78	0,94	0	-1,05	-0,07	0,88	1,00	0	-1,27	-0,33	0
ZEB1	zinc finger E-box binding homeobox 1	0,96	-0,05	0,78	0,94	0	-1,02	-0,03	NA	NA	0	-1,35	-0,44	0
GGH	gamma-glutamyl hydrolase (conjugase, folylpolygammaglutamyl hydrolase)	0,96	-0,05	0,84	0,96	0	1,02	0,02	0,81	1,00	0	NA	NA	0
NPAS2	neuronal PAS domain protein 2	0,96	-0,05	0,75	0,93	0	-1,22	-0,28	0,13	1,00	0	1,04	0,06	0
SREBF1	sterol regulatory element binding transcription factor 1	0,96	-0,05	0,73	0,92	0	1,27	0,34	0,16	1,00	0	1,12	0,17	0
HADH	hydroxyacyl-CoA dehydrogenase	0,96	-0,05	0,71	0,91	0	1,38	0,46	0,00	0,33	0	NA	NA	0
PPP1R8	protein phosphatase 1, regulatory (inhibitor) subunit 8	0,96	-0,06	0,79	0,94	0	1,01	0,02	NA	NA	0	-1,01	-0,01	0
NOTCH3	notch 3	0,96	-0,06	0,81	0,95	0	1,09	0,12	0,48	1,00	0	1,07	0,10	0
NFIB	nuclear factor I/B	0,96	-0,06	0,85	0,96	0	1,09	0,13	0,40	1,00	0	NA	NA	0
PPP1R12A	protein phosphatase 1, regulatory (inhibitor) subunit 12A	0,96	-0,06	0,77	0,93	0	1,15	0,20	0,35	1,00	0	-1,11	-0,15	0
OVOL2	ovo-like 2 (Drosophila)	0,96	-0,06	0,73	0,92	0	-1,07	-0,09	0,68	1,00	0	NA	NA	0
SNTB2	syntrophin, beta 2 (dystrophin-associated protein A1, 59kDa, basic component 2)	0,96	-0,06	0,78	0,94	0	-1,05	-0,07	0,75	1,00	0	NA	NA	0
TMEM45A	transmembrane protein 45A	0,96	-0,06	0,73	0,92	0	-1,04	-0,05	0,79	1,00	0	NA	NA	0
LLGL2	lethal giant larvae homolog 2 (Drosophila)	0,96	-0,06	0,84	0,96	0	1,09	0,12	0,67	1,00	0	NA	NA	0
GBP1	guanylate binding protein 1, interferon-inducible, 67kDa	0,96	-0,06	0,82	0,95	0	NA	NA	NA	NA	0	NA	NA	0
RTP4	receptor (chemosensory) transporter protein 4	0,96	-0,06	0,81	0,95	0	-1,01	-0,01	0,98	1,00	0	-1,02	-0,02	0
TOP2B	topoisomerase (DNA) II beta 180kDa	0,96	-0,06	0,74	0,92	0	-1,08	-0,11	0,26	1,00	0	NA	NA	0
ALDH1B1	aldehyde dehydrogenase 1 family, member B1	0,96	-0,06	0,69	0,91	0	-1,05	-0,07	0,71	1,00	0	-1,32	-0,39	0
NUMB	numb homolog (Drosophila)	0,96	-0,06	0,79	0,94	0	1,04	0,05	0,76	1,00	0	NA	NA	0
SCRN1	secernin 1	0,96	-0,06	0,81	0,95	0	-1,01	-0,01	0,93	1,00	0	NA	NA	0
TTC3	tetratricopeptide repeat domain 3	0,96	-0,06	0,68	0,90	0	-1,08	-0,11	0,26	1,00	0	-1,10	-0,14	0
CTNNBL1	catenin, beta like 1	0,96	-0,06	0,80	0,94	0	1,08	0,11	0,32	1,00	0	-1,09	-0,12	0
KIAA0562	KIAA0562	0,96	-0,06	0,70	0,91	0	1,00	0,01	0,96	1,00	0	0,00	0,00	0
SLC25A15	solute carrier family 25 (mitochondrial carrier; ornithine transporter) member 15	0,96	-0,06	0,75	0,93	0	1,15	0,21	0,51	1,00	0	-1,30	-0,38	0
EXOSC9	exosome component 9	0,96	-0,06	0,71	0,91	0	-1,06	-0,09	0,57	1,00	0	NA	NA	0
MAX	MYC associated factor X	0,96	-0,06	0,69	0,90	0	1,11	0,15	0,23	1,00	0	NA	NA	0
PSMA2	proteasome (prosome, macropain) subunit, alpha type, 2	0,96	-0,06	0,66	0,90	0	1,11	0,15	0,32	1,00	0	-1,06	-0,09	0
CASP4	caspase 4, apoptosis-related cysteine peptidase	0,96	-0,06	0,70	0,91	0	1,01	0,01	0,93	1,00	0	-1,10	-0,13	0
ERAP1	endoplasmic reticulum aminopeptidase 1	0,96	-0,06	0,76	0,93	0	-1,17	-0,23	0,15	1,00	0	1,65	0,72	0
PARP3	poly (ADP-ribose) polymerase family, member 3	0,96	-0,06	0,79	0,94	0	-1,00	0,00	0,98	1,00	0	1,07	0,10	0
PSMB9	proteasome (prosome, macropain) subunit, beta type, 9 (large multifunctional peptidase 2)	0,96	-0,06	0,92	0,98	0	NA	NA	NA	NA	0	NA	NA	0
SYNGR2	synaptogyrin 2	0,96	-0,06	0,76	0,93	0	1,14	0,19	0,42	1,00	0	1,19	0,25	0
C12orf35	chromosome 12 open reading frame 35	0,96	-0,06	0,83	0,96	0	1,05	0,07	0,80	1,00	0	NA	NA	0
ADSL	adenylosuccinate lyase	0,96	-0,06	0,72	0,91	0	-1,02	-0,03	0,87	1,00	0	NA	NA	0
CUL5	cullin 5	0,96	-0,06	0,77	0,93	0	-1,24	-0,31	0,06	1,00	0	NA	NA	0
C16orf68	chromosome 16 open reading frame 68	0,96	-0,06	0,73	0,92	0	1,14	0,19	0,31	1,00	0	-1,12	-0,16	0
TMEM66	transmembrane protein 66	0,96	-0,06	0,70	0,91	0	-1,04	-0,06	0,60	1,00	0	NA	NA	0
PEG10	paternally expressed 10	0,96	-0,06	0,85	0,96	0	-1,16	-0,21	0,63	1,00	0	-1,27	-0,34	0
HLA-C	major histocompatibility complex, class I, C	0,96	-0,06	0,79	0,94	0	-1,13	-0,18	0,33	1,00	0	1,03	0,04	0
AGAP1	ArfGAP with GTPase domain, ankyrin repeat and PH domain 1	0,96	-0,06	0,72	0,92	0	-1,04	-0,06	0,76	1,00	0	-1,33	-0,42	0
OBSL1	obscurin-like 1	0,96	-0,06	0,77	0,93	0	-1,23	-0,30	0,30	1,00	0	NA	NA	0
CPD	carboxypeptidase D	0,96	-0,06	0,81	0,95	0	1,05	0,07	0,73	1,00	0	NA	NA	0
ROCK1	Rho-associated, coiled-coil containing protein kinase 1	0,96	-0,06	0,78	0,94	0	1,05	0,07	0,79	1,00	0	-1,12	-0,17	0
COL1A1	collagen, type I, alpha 1	0,96	-0,06	0,83	0,96	0	-1,04	-0,06	NA	NA	0	-1,39	-0,47	0
TUBG2	tubulin, gamma 2	0,96	-0,06	0,74	0,92	0	1,04	0,06	0,74	1,00	0	NA	NA	0
MRPL24	mitochondrial ribosomal protein L24	0,96	-0,06	0,67	0,90	0	1,00	0,01	0,97	1,00	0	NA	NA	0
NBN	nibrin	0,96	-0,06	0,80	0,94	0	1,02	0,03	0,73	1,00	0	-1,23	-0,31	0
C5orf56	chromosome 5 open reading frame 56	0,96	-0,06	0,73	0,92	0	NA	NA	NA	NA	0	NA	NA	0
NUP155	nucleoporin 155kDa	0,96	-0,06	0,72	0,91	0	-1,04	-0,05	0,71	1,00	0	NA	NA	0
NF1	neurofibromin 1	0,96	-0,06	0,78	0,94	0	-1,67	-0,74	0,13	1,00	0	NA	NA	0
SPCS3	signal peptidase complex subunit 3 homolog (S. cerevisiae)	0,96	-0,06	0,68	0,90	0	-1,15	-0,20	0,09	1,00	0	NA	NA	0
NA	NA	0,96	-0,06	0,64	0,89	0	1,07	0,09	0,75	1,00	0	1,05	0,07	0
APLP2	amyloid beta (A4) precursor-like protein 2	0,96	-0,06	0,77	0,93	0	-1,41	-0,50	0,17	1,00	0	NA	NA	0
SNHG3	small nucleolar RNA host gene 3 (non-protein coding)	0,96	-0,06	0,90	0,98	0	-1,10	-0,14	0,73	1,00	0	-1,22	-0,28	0
BCOR	BCL6 corepressor	0,96	-0,06	0,72	0,92	0	-1,06	-0,09	0,50	1,00	0	1,20	0,27	0
MR1	major histocompatibility complex, class I-related	0,96	-0,06	0,73	0,92	0	1,00	0,00	0,99	1,00	0	0,00	-0,01	0
FASN	fatty acid synthase	0,96	-0,06	0,81	0,95	0	-1,03	-0,04	0,80	1,00	0	NA	NA	0
NEDD4L	neural precursor cell expressed, developmentally down-regulated 4-like	0,96	-0,06	0,87	0,97	0	-1,11	-0,14	0,33	1,00	0	1,03	0,04	0
RAB22A	RAB22A, member RAS oncogene family	0,96	-0,07	0,65	0,89	0	1,15	0,20	0,33	1,00	0	-1,18	-0,23	0
MINA	MYC induced nuclear antigen	0,96	-0,07	0,61	0,87	0	-1,04	-0,06	0,57	1,00	0	NA	NA	0
ENOSF1	enolase superfamily member 1	0,96	-0,07	0,81	0,95	0	-1,04	-0,05	0,79	1,00	0	NA	NA	0
AXL	AXL receptor tyrosine kinase	0,96	-0,07	0,75	0,93	0	-1,14	-0,19	0,30	1,00	0	NA	NA	0
POMT1	protein-O-mannosyltransferase 1	0,96	-0,07	0,74	0,92	0	1,19	0,25	0,41	1,00	0	-1,30	-0,37	0
NUP133	nucleoporin 133kDa	0,96	-0,07	0,67	0,90	0	1,01	0,01	0,93	1,00	0	NA	NA	0
WSB1	WD repeat and SOCS box-containing 1	0,96	-0,07	0,73	0,92	0	1,06	0,08	0,55	1,00	0	NA	NA	0
MUTYH	mutY homolog (E. coli)	0,96	-0,07	0,64	0,89	0	-1,04	-0,05	0,69	1,00	0	NA	NA	0

PCYT1A	phosphate cytidylyltransferase 1, choline, alpha	0,96	-0,07	0,76	0,93	0	1,05	0,07	0,81	1,00	0	NA	NA	0
TROVE2	TROVE domain family, member 2	0,96	-0,07	0,68	0,90	0	-1,08	-0,12	0,40	1,00	0	-1,14	-0,19	0
ITSN1	intersectin 1 (SH3 domain protein)	0,96	-0,07	0,67	0,90	0	-1,04	-0,05	0,75	1,00	0	-1,14	-0,18	0
NEK9	NIMA (never in mitosis gene a)- related kinase 9	0,95	-0,07	0,64	0,89	0	-1,04	-0,05	0,74	1,00	0	NA	NA	0
CREM	cAMP responsive element modulator	0,95	-0,07	0,76	0,93	0	1,08	0,11	0,65	1,00	0	-1,22	-0,29	0
KIF3A	kinesin family member 3A	0,95	-0,07	0,72	0,91	0	-1,04	-0,06	0,67	1,00	0	-1,20	-0,26	0
RUNX1	runt-related transcription factor 1	0,95	-0,07	0,70	0,91	0	1,00	0,00	1,00	1,00	0	NA	NA	0
ARHGEF1	Rho guanine nucleotide exchange factor (GEF) 1	0,95	-0,07	0,74	0,92	0	1,08	0,12	0,49	1,00	0	2,25	1,17	0
PTPRA	protein tyrosine phosphatase, receptor type, A	0,95	-0,07	0,63	0,88	0	-1,12	-0,17	0,30	1,00	0	1,11	0,15	0
NA	NA	0,95	-0,07	0,69	0,91	0	NA	NA	NA	NA	0	NA	NA	0
BTN2A1	butyrophilin, subfamily 2, member A1	0,95	-0,07	0,80	0,94	0	-1,00	0,00	1,00	1,00	0	NA	NA	0
NARS2	asparaginyl-tRNA synthetase 2, mitochondrial (putative)	0,95	-0,07	0,66	0,89	0	1,09	0,13	0,53	1,00	0	-1,25	-0,32	0
MID1	midline 1 (Opitz/BBB syndrome)	0,95	-0,07	0,76	0,93	0	-1,13	-0,18	0,36	1,00	0	NA	NA	0
NIF3L1	NIF3 NGG1 interacting factor 3-like 1 (S. pombe)	0,95	-0,07	0,66	0,90	0	1,04	0,06	0,64	1,00	0	NA	NA	0
VAMP5	vesicle-associated membrane protein 5 (myobrevin)	0,95	-0,07	0,69	0,91	0	-1,01	-0,01	NA	NA	0	0,00	-0,01	0
CERK	ceramide kinase	0,95	-0,07	0,67	0,90	0	1,02	0,03	0,88	1,00	0	-1,02	-0,03	0
OGFOD2	2-oxoglutarate and iron-dependent oxygenase domain containing 2	0,95	-0,07	0,72	0,92	0	-1,12	-0,16	0,67	1,00	0	NA	NA	0
ERLIN1	ER lipid raft associated 1	0,95	-0,07	0,61	0,87	0	1,00	0,01	0,94	1,00	0	NA	NA	0
TUBGCP3	tubulin, gamma complex associated protein 3	0,95	-0,07	0,71	0,91	0	-1,13	-0,17	0,16	1,00	0	NA	NA	0
PRPF38B	PRP38 pre-mRNA processing factor 38 (yeast) domain containing B	0,95	-0,07	0,76	0,93	0	1,03	0,05	0,75	1,00	0	NA	NA	0
SCAMP1	secretory carrier membrane protein 1	0,95	-0,07	0,61	0,87	0	-1,01	-0,01	0,99	1,00	0	-1,37	-0,45	0
SIP1	survival of motor neuron protein interacting protein 1	0,95	-0,07	0,80	0,94	0	1,11	0,15	0,51	1,00	0	NA	NA	0
ENG	endoglin	0,95	-0,07	0,78	0,94	0	1,00	0,00	NA	NA	0	1,07	0,10	0
PAQR3	progestin and adipoQ receptor family member III	0,95	-0,07	0,79	0,94	0	1,11	0,15	0,38	1,00	0	NA	NA	0
FHL1	four and a half LIM domains 1	0,95	-0,07	0,72	0,92	0	-1,18	-0,24	0,50	1,00	0	1,07	0,10	0
FAM189B	family with sequence similarity 189, member B	0,95	-0,07	0,66	0,90	0	1,10	0,14	0,19	1,00	0	1,08	0,11	0
KDM6A	lysine (K)-specific demethylase 6A	0,95	-0,07	0,82	0,95	0	1,11	0,15	0,52	1,00	0	-1,08	-0,11	0
SPRY1	sprouty homolog 1, antagonist of FGF signaling (Drosophila)	0,95	-0,07	0,81	0,95	0	-1,31	-0,39	0,42	1,00	0	NA	NA	0
PHLDB1	pleckstrin homology-like domain, family B, member 1	0,95	-0,07	0,68	0,90	0	-1,10	-0,14	0,30	1,00	0	NA	NA	0
PON2	paraoxonase 2	0,95	-0,07	0,74	0,92	0	1,02	0,03	0,69	1,00	0	NA	NA	0
TPD52	tumor protein D52	0,95	-0,07	0,80	0,95	0	1,15	0,20	0,69	1,00	0	-1,49	-0,58	0
RC3H2	ring finger and CCCH-type zinc finger domains 2	0,95	-0,07	0,79	0,94	0	1,20	0,26	0,16	1,00	0	NA	NA	0
IFT81	intraflagellar transport 81 homolog (Chlamydomonas)	0,95	-0,07	0,75	0,93	0	1,02	0,03	0,90	1,00	0	-1,11	-0,15	0
DIMT1L	DIM1 dimethyladenosine transferase 1-like (S. cerevisiae)	0,95	-0,07	0,75	0,93	0	1,06	0,08	0,62	1,00	0	-1,09	-0,12	0
ERAP2	endoplasmic reticulum aminopeptidase 2	0,95	-0,07	0,69	0,90	0	NA	NA	NA	NA	0	NA	NA	0
MRPS34	mitochondrial ribosomal protein S34	0,95	-0,07	0,64	0,88	0	-1,09	-0,12	0,24	1,00	0	NA	NA	0
TNFSF10	tumor necrosis factor (ligand) superfamily, member 10	0,95	-0,07	0,84	0,96	0	1,85	0,88	0,14	1,00	0	1,01	0,01	0
OAS2	2'-5'-oligoadenylate synthetase 2, 69/71kDa	0,95	-0,07	0,85	0,96	0	-1,02	-0,03	0,92	1,00	0	-1,19	-0,24	0
MYD88	myeloid differentiation primary response gene (88)	0,95	-0,07	0,78	0,94	0	1,16	0,21	0,11	1,00	0	-1,18	-0,23	0
POPDC3	popeye domain containing 3	0,95	-0,07	0,73	0,92	0	1,00	0,01	NA	NA	0	-1,15	-0,20	0
ATF1	activating transcription factor 1	0,95	-0,07	0,76	0,93	0	1,03	0,04	0,71	1,00	0	NA	NA	0
MR1	major histocompatibility complex, class I-related	0,95	-0,07	0,77	0,93	0	-1,23	-0,30	0,03	0,96	0	-1,22	-0,28	0
NA	NA	0,95	-0,07	0,74	0,92	0	1,24	0,31	0,37	1,00	0	-1,43	-0,51	0
ANKRD5	ankyrin repeat domain 5	0,95	-0,07	0,66	0,89	0	-1,24	-0,31	0,06	1,00	0	NA	NA	0
ATR	ataxia telangiectasia and Rad3 related	0,95	-0,07	0,74	0,92	0	-1,09	-0,12	0,46	1,00	0	NA	NA	0
ITSN1	intersectin 1 (SH3 domain protein)	0,95	-0,07	0,69	0,90	0	-1,12	-0,16	0,55	1,00	0	-1,06	-0,09	0
LAMB1	laminin, beta 1	0,95	-0,07	0,79	0,94	0	-1,20	-0,26	0,05	1,00	0	NA	NA	0
SLC41A3	solute carrier family 41, member 3	0,95	-0,07	0,68	0,90	0	1,42	0,50	0,02	0,94	0	1,13	0,17	0
UTY	ubiquitously transcribed tetratricopeptide repeat gene, Y-linked	0,95	-0,07	0,62	0,88	0	-1,11	-0,16	0,36	1,00	0	NA	NA	0
SLC25A36	solute carrier family 25, member 36	0,95	-0,07	0,74	0,92	0	-1,14	-0,18	0,58	1,00	0	-2,22	-1,15	0
TCTN3	tectonic family member 3	0,95	-0,07	0,70	0,91	0	1,02	0,03	0,79	1,00	0	NA	NA	0
PKP4	plakophilin 4	0,95	-0,07	0,59	0,86	0	-1,11	-0,15	0,29	1,00	0	-1,02	-0,03	0
USP15	ubiquitin specific peptidase 15	0,95	-0,07	0,65	0,89	0	1,04	0,06	0,59	1,00	0	-1,28	-0,36	0
ICAM1	intercellular adhesion molecule 1	0,95	-0,07	0,68	0,90	0	-1,06	-0,08	0,73	1,00	0	1,07	0,10	0
CASP1	caspase 1, apoptosis-related cysteine peptidase (interleukin 1, beta, convertase)	0,95	-0,08	0,64	0,88	0	-1,05	-0,07	0,63	1,00	0	-1,32	-0,40	0
STEAP3	STEAP family member 3	0,95	-0,08	0,75	0,93	0	-1,05	-0,07	0,63	1,00	0	NA	NA	0
TMX4	thioredoxin-related transmembrane protein 4	0,95	-0,08	0,74	0,92	0	-1,10	-0,13	0,42	1,00	0	-1,22	-0,28	0
APOBEC3A	apolipoprotein B mRNA editing enzyme, catalytic polypeptide-like 3A	0,95	-0,08	0,95	0,99	0	1,01	0,01	0,99	1,00	0	NA	NA	0
CALM3	calmodulin 3 (phosphorylase kinase, delta)	0,95	-0,08	0,67	0,90	0	-1,16	-0,22	0,26	1,00	0	NA	NA	0
CDC7	cell division cycle 7 homolog (S. cerevisiae)	0,95	-0,08	0,75	0,93	0	-1,04	-0,06	0,74	1,00	0	NA	NA	0
SNAPC4	small nuclear RNA activating complex, polypeptide 4, 190kDa	0,95	-0,08	0,74	0,92	0	-1,13	-0,18	0,47	1,00	0	NA	NA	0
ATP2B1	ATPase, Ca++ transporting, plasma membrane 1	0,95	-0,08	0,77	0,93	0	-1,15	-0,20	0,35	1,00	0	NA	NA	0
TBL1XR1	transducin (beta)-like 1 X-linked receptor 1	0,95	-0,08	0,79	0,94	0	1,04	0,06	0,86	1,00	0	-1,32	-0,39	0
NA	NA	0,95	-0,08	0,65	0,89	0	NA	NA	NA	NA	0	NA	NA	0
TAF5	TAF5 RNA polymerase II, TATA box binding protein (TBP)-associated factor, 100kDa	0,95	-0,08	0,62	0,88	0	1,03	0,05	0,85	1,00	0	NA	NA	0
SCO2	SCO cytochrome oxidase deficient homolog 2 (yeast)	0,95	-0,08	0,81	0,95	0	1,09	0,12	0,52	1,00	0	NA	NA	0
PAICS	phosphoribosylaminoimidazole carboxylase, phosphoribosylaminoimidazole succinocarboxamide synthetase	0,95	-0,08	0,67	0,90	0	-1,06	-0,08	0,45	1,00	0	NA	NA	0
TUBGCP4	tubulin, gamma complex associated protein 4	0,95	-0,08	0,71	0,91	0	1,04	0,05	0,76	1,00	0	NA	NA	0
APOOL	apolipoprotein O-like	0,95	-0,08	0,58	0,86	0	1,04	0,05	0,75	1,00	0	-1,12	-0,17	0
DIO2	deiodinase, iodothyronine, type II	0,95	-0,08	0,88	0,97	0	1,04	0,06	NA	NA	0	1,31	0,39	0
GTF2H1	general transcription factor IIH, polypeptide 1, 62kDa	0,95	-0,08	0,70	0,91	0	1,17	0,23	0,44	1,00	0	-1,25	-0,32	0
CRELD2	cysteine-rich with EGF-like domains 2	0,95	-0,08	0,72	0,91	0	-1,09	-0,12	0,25	1,00	0	NA	NA	0
SR140	U2-associated SR140 protein	0,95	-0,08	0,62	0,88	0	-1,02	-0,03	0,82	1,00	0	-1,37	-0,46	0
STX12	syntaxin 12	0,95	-0,08	0,64	0,89	0	1,31	0,39	0,00	0,20	0	NA	NA	0
ATF2	activating transcription factor 2	0,95	-0,08	0,75	0,93	0	-1,50	-0,58	0,48	1,00	0	-1,37	-0,45	0
FOXO1	forkhead box O1	0,95	-0,08	0,61	0,87	0	-1,32	-0,40	0,06	1,00	0	-1,64	-0,71	0
PARP2	poly (ADP-ribose) polymerase 2	0,95	-0,08	0,58	0,86	0	1,08	0,11	0,62	1,00	0	NA	NA	0
MAP4K5	mitogen-activated protein kinase kinase kinase kinase 5	0,95	-0,08	0,74	0,93	0	1,00	0,00	0,96	1,00	0	-1,05	-0,07	0
ATP1A1	ATPase, Na+/K+ transporting, alpha 1 polypeptide	0,95	-0,08	0,75	0,93	0	-1,12	-0,16	0,04	1,00	0	NA	NA	0
DOM3Z	dom-3 homolog Z (C. elegans)	0,95	-0,08	0,60	0,87	0	-1,43	-0,52	0,10	1,00	0	NA	NA	0
SDCCAG1	serologically defined colon cancer antigen 1	0,95	-0,08	0,60	0,87	0	-1,17	-0,23	0,11	1,00	0	NA	NA	0
ZNHIT6	zinc finger, HIT type 6	0,95	-0,08	0,67	0,90	0	1,03	0,04	0,69	1,00	0	NA	NA	0
VDR	vitamin D (1,25- dihydroxyvitamin D3) receptor	0,95	-0,08	0,67	0,90	0	-1,20	-0,27	0,07	1,00	0	NA	NA	0
PIAS2	protein inhibitor of activated STAT, 2	0,95	-0,08	0,77	0,93	0	1,33	0,41	0,04	1,00	0	-1,25	-0,32	0
IFT27	intraflagellar transport 27 homolog (Chlamydomonas)	0,95	-0,08	0,69	0,91	0	-1,25	-0,32	0,14	1,00	0	NA	NA	0
PDSS1	prenyl (decaprenyl) diphosphate synthase, subunit 1	0,95	-0,08	0,83	0,96	0	1,16	0,21	0,30	1,00	0	NA	NA	0
MRPS27	mitochondrial ribosomal protein S27	0,95	-0,08	0,68	0,90	0	-1,15	-0,20	0,04	1,00	0	NA	NA	0
PRKCI	protein kinase C, iota	0,95	-0,08	0,62	0,88	0	-1,04	-0,05	0,71	1,00	0	-1,43	-0,52	0
SACS	spastic ataxia of Charlevoix-Saguenay (sacsin)	0,95	-0,08	0,72	0,92	0	1,20	0,26	0,14	1,00	0	1,10	0,14	0
KIF3C	kinesin family member 3C	0,95	-0,08	0,86	0,96	0	1,12	0,17	0,53	1,00	0	-1,30	-0,38	0
HLA-B	major histocompatibility complex, class I, B	0,95	-0,08	0,64	0,88	0	-1,16	-0,22	0,16	1,00	0	-1,08	-0,10	0
NFIB	nuclear factor I/B	0,95	-0,08	0,68	0,90	0	-1,15	-0,20	0,48	1,00	0	-1,06	-0,09	0
TNFRSF1A	tumor necrosis factor receptor superfamily, member 1A	0,95	-0,08	0,64	0,88	0	-1,01	-0,02	0,90	1,00	0	NA	NA	0
SLC19A2	solute carrier family 19 (thiamine transporter), member 2	0,95	-0,08	0,73	0,92	0	1,05	0,07	0,68	1,00	0	-1,08	-0,11	0
BAT5	HLA-B associated transcript 5	0,95	-0,08	0,73	0,92	0	NA	NA	NA	NA	0	NA	NA	0
NEK1	NIMA (never in mitosis gene a)-related kinase 1	0,95	-0,08	0,66	0,89	0	-1,01	-0,02	NA	NA	0	-1,09	-0,13	0
PIK3IP1	phosphoinositide-3-kinase interacting protein 1	0,95	-0,08	0,91	0,98	0	1,20	0,27	0,30	1,00	0	NA	NA	0
OPA1	optic atrophy 1 (autosomal dominant)	0,95	-0,08	0,66	0,90	0	1,11	0,15	0,34	1,00	0	-1,45	-0,54	0
HNRNPA3	heterogeneous nuclear ribonucleoprotein A3	0,95	-0,08	0,64	0,89	0	-1,12	-0,16	0,21	1,00	0	-1,16	-0,23	0
PYGL	phosphorylase, glycogen, liver	0,95	-0,08	0,79	0,94	0	-1,03	-0,05	0,69	1,00	0	-1,22	-0,29	0
LSM4	LSM4 homolog, U6 small nuclear RNA associated (S. cerevisiae)	0,95	-0,08	0,56	0,85	0	-1,10	-0,14	0,23	1,00	0	NA	NA	0
RPL23	ribosomal protein L23	0,95	-0,08	0,81	0,95	0	1,23	0,30	0,24	1,00	0	-1,23	-0,31	0
HLA-B	major histocompatibility complex, class I, B	0,95	-0,08	0,70	0,91	0	-1,28	-0,36	0,21	1,00	0	1,05	0,07	0
TINAGL1	tubulointerstitial nephritis antigen-like 1	0,94	-0,08	0,84	0,96	0	-1,12	-0,16	0,41	1,00	0	1,02	0,03	0
NA	NA	0,94	-0,08	0,76	0,93	0	-1,15	-0,20	0,41	1,00	0	NA	NA	0
PER1	period homolog 1 (Drosophila)	0,94	-0,08	0,75	0,93	0	-1,03	-0,04	0,83	1,00	0	NA	NA	0
LGR4	leucine-rich repeat-containing G protein-coupled receptor 4	0,94	-0,08	0,72	0,92	0	1,05	0,07	0,58	1,00	0	NA	NA	0
NUMA1	nuclear mitotic apparatus protein 1	0,94	-0,08	0,66	0,89	0	-1,26	-0,34	0,04	1,00	0	NA	NA	0
CREM	cAMP responsive element modulator	0,94	-0,08	0,71	0,91	0	1,27	0,35	0,20	1,00	0	-1,22	-0,28	0
POLG2	polymerase (DNA directed), gamma 2, accessory subunit	0,94	-0,08	0,72	0,92	0	1,02	0,03	0,90	1,00	0	NA	NA	0
YLPM1	YLP motif containing 1	0,94	-0,08	0,65	0,89	0	1,10	0,13	0,16	1,00	0	NA	NA	0
C11orf17	chromosome 11 open reading frame 17	0,94	-0,08	0,55	0,84	0	-1,12	-0,16	0,41	1,00	0	NA	NA	0
TCF3	transcription factor 3 (E2A immunoglobulin enhancer binding factors E12/E47)	0,94	-0,08	0,62	0,88	0	-1,13	-0,18	0,30	1,00	0	NA	NA	0
NFIC	nuclear factor I/C (CCAAT-binding transcription factor)	0,94	-0,08	0,77	0,93	0	1,10	0,14	0,54	1,00	0	1,14	0,19	0
SRSF8	serine/arginine-rich splicing factor 8	0,94	-0,08	0,62	0,88	0	1,01	0,02	0,91	1,00	0	-1,20	-0,26	0
CYP20A1	cytochrome P450, family 20, subfamily A, polypeptide 1	0,94	-0,08	0,58	0,86	0	-1,19	-0,25	0,32	1,00	0	NA	NA	0
PPP3CA	protein phosphatase 3, catalytic subunit, alpha isozyme	0,94	-0,08	0,66	0,90	0	-1,08	-0,11	0,30	1,00	0	NA	NA	0
SCYL3	SCY1-like 3 (S. cerevisiae)	0,94	-0,08	0,65	0,89	0	-1,03	-0,05	0,75	1,00	0	-1,43	-0,53	0
APOL1	apolipoprotein L, 1	0,94	-0,08	0,94	0,98	0	-1,02	-0,03	0,85	1,00	0	0,00	0,00	0
PSPH	phosphoserine phosphatase	0,94	-0,08	0,67	0,90	0	-1,29	-0,36	0,06	1,00	0	NA	NA	0
SPAG5	sperm associated antigen 5	0,94	-0,08	0,74	0,93	0	1,09	0,12	0,61	1,00	0	NA	NA	0
ROD1	ROD1 regulator of differentiation 1 (S. pombe)	0,94	-0,08	0,75	0,93	0	-1,08	-0,11	0,61	1,00	0	-1,43	-0,51	0
THOC5	THO complex 5	0,94	-0,08	0,58	0,86	0	-1,07	-0,09	0,36	1,00	0	NA	NA	0
PAM	peptidylglycine alpha-amidating monooxygenase	0,94	-0,08	0,58	0,86	0	1,02	0,03	0,85	1,00	0	NA	NA	0
CTDSP2	CTD (carboxy-terminal domain, RNA polymerase II, polypeptide A) small phosphatase 2	0,94	-0,08	0,59	0,87	0	-1,10	-0,14	0,20	1,00	0	NA	NA	0
TM7SF3	transmembrane 7 superfamily member 3	0,94	-0,08	0,68	0,90	0	-1,09	-0,12	0,59	1,00	0	-1,35	-0,43	0
NAT6	N-acetyltransferase 6 (GCN5-related)	0,94	-0,08	0,65	0,89	0	-1,43	-0,51	0,13	1,00	0	NA	NA	0
R3HDM1	R3H domain containing 1	0,94	-0,08	0,59	0,87	0	-1,05	-0,06	0,63	1,00	0	NA	NA	0
GMPR2	guanosine monophosphate reductase 2	0,94	-0,09	0,59	0,87	0	-1,20	-0,26	0,05	1,00	0	NA	NA	0
TBC1D5	TBC1 domain family, member 5	0,94	-0,09	0,65	0,89	0	-1,13	-0,18	0,16	1,00	0	NA	NA	0
NA	NA	0,94	-0,09	0,58	0,86	0	1,03	0,04	0,85	1,00	0	1,05	0,07	0
CPEB3	cytoplasmic polyadenylation element binding protein 3	0,94	-0,09	0,75	0,93	0	-1,13	-0,18	0,43	1,00	0	1,02	0,02	0
SDAD1	SDA1 domain containing 1	0,94	-0,09	0,62	0,88	0	-1,13	-0,18	0,21	1,00	0	1,38	0,47	0
MAP3K11	mitogen-activated protein kinase kinase kinase 11	0,94	-0,09	0,77	0,93	0	-1,04	-0,05	0,87	1,00	0	NA	NA	0
LEPRE1	leucine proline-enriched proteoglycan (leprecan) 1	0,94	-0,09	0,73	0,92	0	-1,12	-0,16	0,19	1,00	0	NA	NA	0
MUC1	mucin 1, cell surface associated	0,94	-0,09	0,89	0,97	0	-1,01	-0,01	0,98	1,00	0	NA	NA	0
DNPEP	aspartyl aminopeptidase	0,94	-0,09	0,70	0,91	0	-1,03	-0,04	0,78	1,00	0	-1,01	-0,01	0

NEAT1	nuclear paraspeckle assembly transcript 1 (non-protein coding)	0,94	-0,09	0,75	0,93	0	1,14	0,18	0,69	1,00	0	-1,89	-0,91	0
SSX2IP	synovial sarcoma, X breakpoint 2 interacting protein	0,94	-0,09	0,60	0,87	0	-1,05	-0,08	0,77	1,00	0	-1,47	-0,56	0
RER1	RER1 retention in endoplasmic reticulum 1 homolog (S. cerevisiae)	0,94	-0,09	0,59	0,87	0	1,01	0,02	NA	NA	0	-1,67	-0,73	0
TTLL5	tubulin tyrosine ligase-like family, member 5	0,94	-0,09	0,50	0,82	0	-1,08	-0,11	0,57	1,00	0	NA	NA	0
SEC62	SEC62 homolog (S. cerevisiae)	0,94	-0,09	0,71	0,91	0	NA	NA	NA	NA	0	NA	NA	0
C11orf30	chromosome 11 open reading frame 30	0,94	-0,09	0,61	0,88	0	-1,03	-0,05	0,64	1,00	0	-1,18	-0,23	0
CLCA2	chloride channel accessory 2	0,94	-0,09	0,67	0,90	0	-1,25	-0,32	0,07	1,00	0	NA	NA	0
SH3BP2	SH3-domain binding protein 2	0,94	-0,09	0,50	0,82	0	1,02	0,03	0,94	1,00	0	-1,06	-0,08	0
PPM1D	protein phosphatase, Mg2+/Mn2+ dependent, 1D	0,94	-0,09	0,68	0,90	0	1,21	0,28	0,33	1,00	0	-1,27	-0,34	0
RABGGTB	Rab geranylgeranyltransferase, beta subunit	0,94	-0,09	0,80	0,94	0	1,00	0,00	0,99	1,00	0	NA	NA	0
CD2AP	CD2-associated protein	0,94	-0,09	0,67	0,90	0	1,03	0,04	0,73	1,00	0	-1,09	-0,13	0
CDC37L1	cell division cycle 37 homolog (S. cerevisiae)-like 1	0,94	-0,09	0,70	0,91	0	-1,13	-0,17	0,35	1,00	0	-1,43	-0,52	0
TMCO3	transmembrane and coiled-coil domains 3	0,94	-0,09	0,78	0,94	0	-1,04	-0,05	0,88	1,00	0	1,07	0,09	0
DOM3Z	dom-3 homolog Z (C. elegans)	0,94	-0,09	0,62	0,88	0	-1,13	-0,18	0,47	1,00	0	NA	NA	0
RBMX	RNA binding motif protein, X-linked	0,94	-0,09	0,48	0,81	0	-1,13	-0,17	0,07	1,00	0	NA	NA	0
SLC25A36	solute carrier family 25, member 36	0,94	-0,09	0,68	0,90	0	-1,36	-0,44	0,23	1,00	0	-1,59	-0,66	0
AKT3	v-akt murine thymoma viral oncogene homolog 3 (protein kinase B, gamma)	0,94	-0,09	0,65	0,89	0	1,14	0,19	0,31	1,00	0	1,09	0,12	0
JUN	jun proto-oncogene	0,94	-0,09	0,76	0,93	0	1,17	0,22	0,45	1,00	0	NA	NA	0
FLRT2	fibronectin leucine rich transmembrane protein 2	0,94	-0,09	0,54	0,84	0	-1,16	-0,22	0,28	1,00	0	-1,32	-0,40	0
SF3A3	splicing factor 3a, subunit 3, 60kDa	0,94	-0,09	0,63	0,88	0	1,13	0,17	0,53	1,00	0	NA	NA	0
MICB	MHC class I polypeptide-related sequence B	0,94	-0,09	0,79	0,94	0	1,13	0,18	0,51	1,00	0	-1,03	-0,05	0
KPNA1	karyopherin alpha 1 (importin alpha 5)	0,94	-0,09	0,53	0,84	0	-1,22	-0,29	0,63	1,00	0	NA	NA	0
ZYX	zyxin	0,94	-0,09	0,69	0,91	0	-1,03	-0,04	0,79	1,00	0	1,58	0,66	0
LSM4	LSM4 homolog, U6 small nuclear RNA associated (S. cerevisiae)	0,94	-0,09	0,56	0,85	0	-1,11	-0,15	0,19	1,00	0	-1,11	-0,15	0
PMS1	PMS1 postmeiotic segregation increased 1 (S. cerevisiae)	0,94	-0,09	0,67	0,90	0	-1,02	-0,03	0,89	1,00	0	NA	NA	0
FEM1C	fem-1 homolog c (C. elegans)	0,94	-0,09	0,59	0,86	0	1,03	0,04	0,83	1,00	0	NA	NA	0
TRIP4	thyroid hormone receptor interactor 4	0,94	-0,09	0,60	0,87	0	1,00	0,00	0,96	1,00	0	NA	NA	0
FAM171A1	family with sequence similarity 171, member A1	0,94	-0,09	0,65	0,89	0	1,04	0,06	0,80	1,00	0	-1,35	-0,43	0
LBR	lamin B receptor	0,94	-0,09	0,53	0,83	0	1,02	0,03	0,82	1,00	0	NA	NA	0
ARHGAP19	Rho GTPase activating protein 19	0,94	-0,09	0,67	0,90	0	-1,07	-0,10	0,73	1,00	0	NA	NA	0
ORC2L	origin recognition complex, subunit 2-like (yeast)	0,94	-0,09	0,57	0,85	0	-1,02	-0,04	0,80	1,00	0	NA	NA	0
ARF3	ADP-ribosylation factor 3	0,94	-0,09	0,66	0,89	0	1,03	0,04	0,63	1,00	0	NA	NA	0
DBF4	DBF4 homolog (S. cerevisiae)	0,94	-0,09	0,63	0,88	0	1,13	0,17	0,27	1,00	0	NA	NA	0
SLC16A3	solute carrier family 16, member 3 (monocarboxylic acid transporter 4)	0,94	-0,09	0,80	0,94	0	-1,13	-0,17	0,87	1,00	0	NA	NA	0
CUL4B	cullin 4B	0,94	-0,09	0,62	0,88	0	-1,33	-0,42	0,39	1,00	0	-1,27	-0,34	0
TNS4	tensin 4	0,94	-0,09	0,87	0,97	0	-1,56	-0,64	0,02	0,94	0	NA	NA	0
FZD6	frizzled homolog 6 (Drosophila)	0,94	-0,09	0,68	0,90	0	1,08	0,11	0,54	1,00	0	-1,25	-0,32	0
TFAM	transcription factor A, mitochondrial	0,94	-0,09	0,65	0,89	0	1,06	0,09	0,52	1,00	0	NA	NA	0
GADD45B	growth arrest and DNA-damage-inducible, beta	0,94	-0,09	0,73	0,92	0	1,00	0,00	0,99	1,00	0	NA	NA	0
ADCY7	adenylate cyclase 7	0,94	-0,09	0,71	0,91	0	1,24	0,31	0,24	1,00	0	-1,27	-0,34	0
TNKS	tankyrase, TRF1-interacting ankyrin-related ADP-ribose polymerase	0,94	-0,09	0,50	0,82	0	-1,11	-0,16	0,53	1,00	0	-1,06	-0,10	0
CAND2	cullin-associated and neddylation-dissociated 2 (putative)	0,94	-0,09	0,78	0,94	0	-1,10	-0,14	0,55	1,00	0	NA	NA	0
FANCE	Fanconi anemia, complementation group E	0,94	-0,09	0,52	0,83	0	1,00	0,00	0,98	1,00	0	-1,12	-0,17	0
INTS7	integrator complex subunit 7	0,94	-0,09	0,65	0,89	0	-1,04	-0,06	0,83	1,00	0	NA	NA	0
FOS	FBJ murine osteosarcoma viral oncogene homolog	0,94	-0,09	0,93	0,98	0	-1,15	-0,20	0,78	1,00	0	NA	NA	0
RSBN1	round spermatid basic protein 1	0,94	-0,09	0,62	0,88	0	-1,13	-0,18	0,47	1,00	0	-1,08	-0,10	0
APH1B	anterior pharynx defective 1 homolog B (C. elegans)	0,94	-0,09	0,67	0,90	0	-1,02	-0,03	0,90	1,00	0	NA	NA	0
GAS1	growth arrest-specific 1	0,94	-0,10	0,56	0,85	0	-1,14	-0,18	0,69	1,00	0	-1,12	-0,18	0
CAMSAP1	calmodulin regulated spectrin-associated protein 1	0,94	-0,10	0,70	0,91	0	1,19	0,25	0,07	1,00	0	-1,16	-0,21	0
BUB3	budding uninhibited by benzimidazoles 3 homolog (yeast)	0,94	-0,10	0,52	0,83	0	-1,13	-0,17	0,10	1,00	0	NA	NA	0
SPATS2	spermatogenesis associated, serine-rich 2	0,94	-0,10	0,61	0,87	0	-1,17	-0,22	0,63	1,00	0	NA	NA	0
PAIP1	poly(A) binding protein interacting protein 1	0,94	-0,10	0,71	0,91	0	-1,04	-0,06	0,66	1,00	0	NA	NA	0
FBXO21	F-box protein 21	0,94	-0,10	0,48	0,81	0	-1,02	-0,03	0,82	1,00	0	NA	NA	0
STX18	syntaxin 18	0,94	-0,10	0,70	0,91	0	1,14	0,19	0,48	1,00	0	-1,14	-0,19	0
PPP3CB	protein phosphatase 3, catalytic subunit, beta isozyme	0,94	-0,10	0,47	0,81	0	-1,02	-0,02	0,77	1,00	0	-1,08	-0,10	0
RPS6KC1	ribosomal protein S6 kinase, 52kDa, polypeptide 1	0,94	-0,10	0,62	0,88	0	1,13	0,17	0,33	1,00	0	NA	NA	0
FAM158A	family with sequence similarity 158, member A	0,94	-0,10	0,62	0,88	0	1,04	0,06	0,76	1,00	0	-1,04	-0,06	0
IL6ST	interleukin 6 signal transducer (gp130, oncostatin M receptor)	0,94	-0,10	0,66	0,90	0	1,19	0,25	0,02	0,84	0	NA	NA	0
DCI	dodecenoyl-CoA isomerase	0,94	-0,10	0,52	0,83	0	1,01	0,02	0,92	1,00	0	NA	NA	0
NA	NA	0,94	-0,10	0,60	0,87	0	NA	NA	NA	NA	0	NA	NA	0
DBN1	drebrin 1	0,94	-0,10	0,66	0,90	0	-1,09	-0,12	0,57	1,00	0	NA	NA	0
CAND1	cullin-associated and neddylation-dissociated 1	0,94	-0,10	0,61	0,88	0	-1,09	-0,12	0,33	1,00	0	NA	NA	0
LUC7L2	LUC7-like 2 (S. cerevisiae)	0,93	-0,10	0,47	0,80	0	-1,04	-0,06	0,78	1,00	0	NA	NA	0
SLC26A6	solute carrier family 26, member 6	0,93	-0,10	0,75	0,93	0	-1,12	-0,16	0,23	1,00	0	NA	NA	0
ARMC9	armadillo repeat containing 9	0,93	-0,10	0,75	0,93	0	-1,14	-0,19	0,23	1,00	0	-1,25	-0,33	0
IER3IP1	immediate early response 3 interacting protein 1	0,93	-0,10	0,64	0,89	0	-1,14	-0,19	0,70	1,00	0	NA	NA	0
CLCC1	chloride channel CLIC-like 1	0,93	-0,10	0,48	0,81	0	-1,10	-0,13	0,40	1,00	0	NA	NA	0
DIXDC1	DIX domain containing 1	0,93	-0,10	0,70	0,91	0	-1,05	-0,07	0,78	1,00	0	-1,15	-0,20	0
NA	NA	0,93	-0,10	0,81	0,95	0	1,03	0,05	0,92	1,00	0	NA	NA	0
CTSB	cathepsin B	0,93	-0,10	0,71	0,91	0	1,01	0,01	0,96	1,00	0	NA	NA	0
PEX7	peroxisomal biogenesis factor 7	0,93	-0,10	0,56	0,85	0	-1,05	-0,07	0,67	1,00	0	NA	NA	0
CASP1	caspase 1, apoptosis-related cysteine peptidase (interleukin 1, beta, convertase)	0,93	-0,10	0,61	0,88	0	-1,10	-0,14	0,39	1,00	0	-1,27	-0,34	0
MAN2A2	mannosidase, alpha, class 2A, member 2	0,93	-0,10	0,73	0,92	0	-1,12	-0,16	0,42	1,00	0	NA	NA	0
MYL9	myosin, light chain 9, regulatory	0,93	-0,10	0,79	0,94	0	-1,06	-0,08	0,70	1,00	0	NA	NA	0
API5	apoptosis inhibitor 5	0,93	-0,10	0,56	0,85	0	1,27	0,35	0,54	1,00	0	-1,19	-0,24	0
ECH1	enoyl CoA hydratase 1, peroxisomal	0,93	-0,10	0,66	0,89	0	-1,01	-0,02	0,90	1,00	0	NA	NA	0
MIOS	missing oocyte, meiosis regulator, homolog (Drosophila)	0,93	-0,10	0,49	0,81	0	-1,01	-0,02	0,92	1,00	0	-1,49	-0,58	0
SGCE	sarcoglycan, epsilon	0,93	-0,10	0,67	0,90	0	-1,09	-0,12	0,78	1,00	0	NA	NA	0
ADSS	adenylosuccinate synthase	0,93	-0,10	0,64	0,89	0	1,01	0,02	0,88	1,00	0	NA	NA	0
TNFRSF10B	tumor necrosis factor receptor superfamily, member 10b	0,93	-0,10	0,52	0,83	0	-1,02	-0,02	0,88	1,00	0	NA	NA	0
POLR1D	polymerase (RNA) I polypeptide D, 16kDa	0,93	-0,10	0,54	0,84	0	-1,08	-0,11	0,37	1,00	0	NA	NA	0
SPARC	secreted protein, acidic, cysteine-rich (osteonectin)	0,93	-0,10	0,77	0,93	0	1,01	0,01	0,99	1,00	0	-1,08	-0,11	0
CCDC25	coiled-coil domain containing 25	0,93	-0,10	0,52	0,83	0	-1,06	-0,08	0,74	1,00	0	NA	NA	0
HDGFRP3	hepatoma-derived growth factor, related protein 3	0,93	-0,10	0,50	0,82	0	-1,07	-0,10	0,61	1,00	0	NA	NA	0
FHL2	four and a half LIM domains 2	0,93	-0,10	0,67	0,90	0	-1,01	-0,01	0,95	1,00	0	-1,11	-0,15	0
BLVRA	biliverdin reductase A	0,93	-0,10	0,75	0,93	0	1,04	0,05	0,87	1,00	0	NA	NA	0
SPTAN1	spectrin, alpha, non-erythrocytic 1 (alpha-fodrin)	0,93	-0,10	0,77	0,93	0	-1,05	-0,07	0,74	1,00	0	1,90	0,92	0
HLA-C	major histocompatibility complex, class I, C	0,93	-0,10	0,59	0,87	0	-1,10	-0,14	0,35	1,00	0	-1,06	-0,10	0
LAP3	leucine aminopeptidase 3	0,93	-0,10	0,74	0,92	0	1,03	0,05	0,79	1,00	0	-1,41	-0,49	0
SETBP1	SET binding protein 1	0,93	-0,10	0,72	0,91	0	-1,08	-0,11	0,57	1,00	0	-1,02	-0,03	0
ZCCHC14	zinc finger, CCHC domain containing 14	0,93	-0,10	0,64	0,89	0	1,17	0,23	0,48	1,00	0	-1,47	-0,56	0
FBXW2	F-box and WD repeat domain containing 2	0,93	-0,10	0,50	0,82	0	-1,10	-0,13	0,27	1,00	0	NA	NA	0
FAM65A	family with sequence similarity 65, member A	0,93	-0,10	0,59	0,87	0	-1,04	-0,06	0,70	1,00	0	NA	NA	0
ANP32A	acidic (leucine-rich) nuclear phosphoprotein 32 family, member A	0,93	-0,10	0,65	0,89	0	-1,14	-0,19	0,12	1,00	0	NA	NA	0
PCCA	propionyl CoA carboxylase, alpha polypeptide	0,93	-0,10	0,43	0,78	0	-1,18	-0,24	0,14	1,00	0	NA	NA	0
C16orf7	chromosome 16 open reading frame 7	0,93	-0,10	0,71	0,91	0	-1,00	0,00	0,98	1,00	0	1,15	0,20	0
MFAP5	microfibrillar associated protein 5	0,93	-0,10	0,83	0,96	0	-1,21	-0,28	0,25	1,00	0	-1,32	-0,40	0
TASP1	taspase, threonine aspartase, 1	0,93	-0,10	0,75	0,93	0	1,02	0,03	0,88	1,00	0	-1,52	-0,61	0
STRA13	stimulated by retinoic acid 13 homolog (mouse)	0,93	-0,10	0,56	0,85	0	1,06	0,08	0,55	1,00	0	NA	NA	0
CDKN1C	cyclin-dependent kinase inhibitor 1C (p57, Kip2)	0,93	-0,10	0,63	0,88	0	1,03	0,04	0,90	1,00	0	NA	NA	0
HLA-G	major histocompatibility complex, class I, G	0,93	-0,10	0,68	0,90	0	-1,06	-0,08	0,82	1,00	0	-1,01	-0,01	0
AKAP1	A kinase (PRKA) anchor protein 1	0,93	-0,10	0,47	0,80	0	-1,03	-0,05	0,79	1,00	0	NA	NA	0
GOLGA8A	golgin A8 family, member A	0,93	-0,10	0,66	0,89	0	-1,01	-0,01	0,95	1,00	0	-1,09	-0,11	0
MYO9B	myosin IXB	0,93	-0,10	0,66	0,90	0	-1,19	-0,25	0,32	1,00	0	NA	NA	0
IVNS1ABP	influenza virus NS1A binding protein	0,93	-0,10	0,50	0,82	0	-1,07	-0,09	0,67	1,00	0	NA	NA	0
KLHDC10	kelch domain containing 10	0,93	-0,10	0,58	0,86	0	1,06	0,08	0,64	1,00	0	-1,04	-0,05	0
MPDZ	multiple PDZ domain protein	0,93	-0,10	0,78	0,94	0	-1,01	-0,01	0,95	1,00	0	-1,39	-0,48	0
CDC42BPA	CDC42 binding protein kinase alpha (DMPK-like)	0,93	-0,10	0,61	0,87	0	-1,08	-0,11	0,31	1,00	0	1,10	0,14	0
PLXNC1	plexin C1	0,93	-0,10	0,79	0,94	0	-1,01	-0,01	NA	NA	0	1,03	0,04	0
PREPL	prolyl endopeptidase-like	0,93	-0,10	0,70	0,91	0	-1,05	-0,07	0,84	1,00	0	-1,09	-0,12	0
SCYL3	SCY1-like 3 (S. cerevisiae)	0,93	-0,10	0,56	0,85	0	-1,01	-0,01	0,96	1,00	0	-1,22	-0,29	0
TNFAIP8	tumor necrosis factor, alpha-induced protein 8	0,93	-0,10	0,57	0,86	0	-1,06	-0,09	0,47	1,00	0	NA	NA	0
NA	NA	0,93	-0,10	0,67	0,90	0	-1,18	-0,24	0,46	1,00	0	-1,28	-0,37	0
FAS	Fas (TNF receptor superfamily, member 6)	0,93	-0,11	0,62	0,88	0	-1,02	-0,03	0,89	1,00	0	-1,02	-0,02	0
FTO	fat mass and obesity associated	0,93	-0,11	0,54	0,84	0	1,04	0,06	0,71	1,00	0	NA	NA	0
MPDZ	multiple PDZ domain protein	0,93	-0,11	0,64	0,88	0	-1,19	-0,25	0,18	1,00	0	-1,23	-0,30	0
AIMP2	aminoacyl tRNA synthetase complex-interacting multifunctional protein 2	0,93	-0,11	0,50	0,82	0	-1,17	-0,22	0,14	1,00	0	-1,08	-0,11	0
MAN1A1	mannosidase, alpha, class 1A, member 1	0,93	-0,11	0,70	0,91	0	-1,26	-0,34	0,54	1,00	0	-1,19	-0,25	0
POLR2A	polymerase (RNA) II (DNA directed) polypeptide A, 220kDa	0,93	-0,11	0,43	0,78	0	1,08	0,11	0,64	1,00	0	1,53	0,61	0
HLA-A	major histocompatibility complex, class I, A	0,93	-0,11	0,39	0,76	0	-1,10	-0,14	0,15	1,00	0	1,01	0,02	0
PRKD2	protein kinase D2	0,93	-0,11	0,79	0,94	0	-1,01	-0,01	0,95	1,00	0	1,04	0,06	0
UBR5	ubiquitin protein ligase E3 component n-recognin 5	0,93	-0,11	0,49	0,81	0	-1,16	-0,21	0,23	1,00	0	NA	NA	0
HDGFRP3	hepatoma-derived growth factor, related protein 3	0,93	-0,11	0,62	0,88	0	-1,05	-0,07	0,49	1,00	0	NA	NA	0
RGS3	regulator of G-protein signaling 3	0,93	-0,11	0,66	0,89	0	1,07	0,10	0,63	1,00	0	1,35	0,43	0
SRSF8	serine/arginine-rich splicing factor 8	0,93	-0,11	0,53	0,83	0	1,02	0,02	0,81	1,00	0	NA	NA	0
APEX1	APEX nuclease (multifunctional DNA repair enzyme) 1	0,93	-0,11	0,46	0,79	0	-1,02	-0,03	0,87	1,00	0	NA	NA	0
C16orf53	chromosome 16 open reading frame 53	0,93	-0,11	0,52	0,83	0	1,06	0,08	0,62	1,00	0	-1,19	-0,26	0
RRN3	RRN3 RNA polymerase I transcription factor homolog (S. cerevisiae)	0,93	-0,11	0,49	0,81	0	1,01	0,02	0,93	1,00	0	NA	NA	0
TMCO3	transmembrane and coiled-coil domains 3	0,93	-0,11	0,64	0,88	0	-1,24	-0,31	0,50	1,00	0	0,00	0,00	0
HSPA12A	heat shock 70kDa protein 12A	0,93	-0,11	0,61	0,87	0	-1,04	-0,06	0,83	1,00	0	-1,03	-0,04	0
NA	NA	0,93	-0,11	0,43	0,78	0	-1,06	-0,09	0,31	1,00	0	NA	NA	0
GOSR1	golgi SNAP receptor complex member 1	0,93	-0,11	0,61	0,88	0	1,09	0,12	0,47	1,00	0	NA	NA	0
SIDT2	SID1 transmembrane family, member 2	0,93	-0,11	0,52	0,83	0	1,19	0,25	0,44	1,00	0	-1,09	-0,13	0
UST	uronyl-2-sulfotransferase	0,93	-0,11	0,61	0,88	0	-1,10	-0,13	0,63	1,00	0	1,18	0,24	0
SMCR7L	Smith-Magenis syndrome chromosome region, candidate 7-like	0,93	-0,11	0,49	0,81	0	1,09	0,13	0,56	1,00	0	NA	NA	0
MST4	serine/threonine protein kinase MST4	0,93	-0,11	0,60	0,87	0	-1,07	-0,10	0,21	1,00	0	-1,23	-0,31	0
LEPROTL1	leptin receptor overlapping transcript-like 1	0,93	-0,11	0,53	0,83	0	1,00	0,00	0,96	1,00	0	NA	NA	0

CHST12	carbohydrate (chondroitin 4) sulfotransferase 12	0,93	-0,11	0,58	0,86	0	-1,01	-0,02	0,92	1,00	0	NA	NA	0
DSG2	desmoglein 2	0,93	-0,11	0,85	0,96	0	-1,00	0,00	0,96	1,00	0	NA	NA	0
KIAA0495	KIAA0495	0,93	-0,11	0,42	0,78	0	-1,13	-0,18	0,19	1,00	0	NA	NA	0
DIDO1	death inducer-obliterator 1	0,93	-0,11	0,43	0,78	0	-1,05	-0,07	0,55	1,00	0	-1,28	-0,35	0
RHOQ	ras homolog gene family, member Q	0,93	-0,11	0,57	0,85	0	1,09	0,13	0,20	1,00	0	-1,03	-0,04	0
RRP8	ribosomal RNA processing 8, methyltransferase, homolog (yeast)	0,93	-0,11	0,48	0,81	0	1,16	0,22	0,36	1,00	0	NA	NA	0
PGAP2	post-GPI attachment to proteins 2	0,93	-0,11	0,41	0,77	0	-1,05	-0,07	0,60	1,00	0	NA	NA	0
PAM	peptidylglycine alpha-amidating monooxygenase	0,93	-0,11	0,54	0,84	0	-1,02	-0,03	0,69	1,00	0	NA	NA	0
FTH1	ferritin, heavy polypeptide 1	0,93	-0,11	0,61	0,88	0	-1,04	-0,05	0,70	1,00	0	NA	NA	0
STAG1	stromal antigen 1	0,93	-0,11	0,40	0,76	0	1,11	0,16	0,18	1,00	0	NA	NA	0
NA	NA	0,93	-0,11	0,46	0,80	0	-1,17	-0,23	0,21	1,00	0	NA	NA	0
HAUS5	HAUS augmin-like complex, subunit 5	0,93	-0,11	0,56	0,85	0	-1,11	-0,15	0,59	1,00	0	NA	NA	0
SEC24A	SEC24 family, member A (S. cerevisiae)	0,92	-0,11	0,57	0,86	0	-1,02	-0,03	0,80	1,00	0	NA	NA	0
RHEB	Ras homolog enriched in brain	0,92	-0,11	0,48	0,81	0	-1,16	-0,21	0,51	1,00	0	NA	NA	0
CCBL1	cysteine conjugate-beta lyase, cytoplasmic	0,92	-0,11	0,72	0,92	0	-1,30	-0,38	0,16	1,00	0	NA	NA	0
C16orf45	chromosome 16 open reading frame 45	0,92	-0,11	0,56	0,85	0	-1,12	-0,16	0,20	1,00	0	-1,35	-0,44	0
DCHS1	dachsous 1 (Drosophila)	0,92	-0,11	0,64	0,88	0	-1,00	0,00	NA	NA	0	1,04	0,06	0
SUV420H1	suppressor of variegation 4-20 homolog 1 (Drosophila)	0,92	-0,11	0,42	0,78	0	-1,01	-0,01	0,94	1,00	0	-1,37	-0,46	0
PPP3CB	protein phosphatase 3, catalytic subunit, beta isozyme	0,92	-0,11	0,46	0,79	0	1,08	0,11	0,50	1,00	0	-1,08	-0,10	0
EGFR	epidermal growth factor receptor	0,92	-0,11	0,54	0,84	0	-1,00	-0,01	0,97	1,00	0	NA	NA	0
CRYBG3	beta-gamma crystallin domain containing 3	0,92	-0,11	0,50	0,82	0	1,03	0,04	0,74	1,00	0	NA	NA	0
NA	NA	0,92	-0,11	0,55	0,85	0	1,08	0,11	0,39	1,00	0	NA	NA	0
STAT2	signal transducer and activator of transcription 2, 113kDa	0,92	-0,11	0,52	0,83	0	-1,13	-0,18	0,79	1,00	0	-1,52	-0,60	0
ZMYM1	zinc finger, MYM-type 1	0,92	-0,12	0,48	0,81	0	-1,05	-0,07	0,71	1,00	0	NA	NA	0
CSRP2	cysteine and glycine-rich protein 2	0,92	-0,12	0,69	0,91	0	-1,04	-0,05	0,75	1,00	0	-1,22	-0,29	0
DVL3	dishevelled, dsh homolog 3 (Drosophila)	0,92	-0,12	0,47	0,80	0	1,02	0,03	0,90	1,00	0	1,01	0,01	0
EPB41L2	erythrocyte membrane protein band 4.1-like 2	0,92	-0,12	0,67	0,90	0	-1,38	-0,47	0,37	1,00	0	-1,39	-0,47	0
TRIM26	tripartite motif-containing 26	0,92	-0,12	0,71	0,91	0	1,03	0,05	0,86	1,00	0	-1,01	-0,01	0
PCNT	pericentrin	0,92	-0,12	0,53	0,84	0	-1,05	-0,07	0,78	1,00	0	NA	NA	0
ANGPTL4	angiopoietin-like 4	0,92	-0,12	0,69	0,90	0	-1,12	-0,17	0,38	1,00	0	NA	NA	0
ETS2	v-ets erythroblastosis virus E26 oncogene homolog 2 (avian)	0,92	-0,12	0,48	0,81	0	1,36	0,44	0,02	0,84	0	NA	NA	0
MAN1A2	mannosidase, alpha, class 1A, member 2	0,92	-0,12	0,46	0,80	0	-1,08	-0,11	0,48	1,00	0	-1,33	-0,41	0
MIPEP	mitochondrial intermediate peptidase	0,92	-0,12	0,56	0,85	0	1,06	0,09	0,60	1,00	0	NA	NA	0
EVC	Ellis van Creveld syndrome	0,92	-0,12	0,59	0,87	0	-1,74	-0,80	0,05	1,00	0	-1,09	-0,12	0
ARRB2	arrestin, beta 2	0,92	-0,12	0,62	0,88	0	-1,36	-0,45	0,09	1,00	0	NA	NA	0
ZFP36L1	zinc finger protein 36, C3H type-like 1	0,92	-0,12	0,47	0,80	0	-1,20	-0,26	0,20	1,00	0	NA	NA	0
LASS6	LAG1 homolog, ceramide synthase 6	0,92	-0,12	0,68	0,90	0	1,04	0,06	0,72	1,00	0	NA	NA	0
PEMT	phosphatidylethanolamine N-methyltransferase	0,92	-0,12	0,44	0,78	0	-1,13	-0,18	0,42	1,00	0	NA	NA	0
USP47	ubiquitin specific peptidase 47	0,92	-0,12	0,45	0,79	0	NA	NA	NA	NA	0	NA	NA	0
RNF6	ring finger protein (C3H2C3 type) 6	0,92	-0,12	0,75	0,93	0	-1,23	-0,29	0,50	1,00	0	-1,12	-0,16	0
RTF1	Rtf1, Paf1/RNA polymerase II complex component, homolog (S. cerevisiae)	0,92	-0,12	0,39	0,76	0	-1,26	-0,33	0,00	0,44	0	NA	NA	0
MME	membrane metallo-endopeptidase	0,92	-0,12	0,47	0,81	0	-1,01	-0,01	NA	NA	0	0,00	0,00	0
TGFBRAP1	transforming growth factor, beta receptor associated protein 1	0,92	-0,12	0,42	0,77	0	1,23	0,29	0,12	1,00	0	NA	NA	0
NA	NA	0,92	-0,12	0,48	0,81	0	-1,02	-0,02	0,93	1,00	0	NA	NA	0
PPP1R12A	protein phosphatase 1, regulatory (inhibitor) subunit 12A	0,92	-0,12	0,36	0,74	0	1,01	0,02	0,89	1,00	0	-1,04	-0,06	0
CLCA2	chloride channel accessory 2	0,92	-0,12	0,61	0,87	0	-1,16	-0,21	0,19	1,00	0	NA	NA	0
NMT1	N-myristoyltransferase 1	0,92	-0,12	0,63	0,88	0	-1,07	-0,09	0,74	1,00	0	1,31	0,39	0
INSR	insulin receptor	0,92	-0,12	0,47	0,80	0	-1,03	-0,04	0,92	1,00	0	NA	NA	0
WDR6	WD repeat domain 6	0,92	-0,12	0,42	0,78	0	-1,01	-0,01	0,94	1,00	0	NA	NA	0
C14orf159	chromosome 14 open reading frame 159	0,92	-0,12	0,43	0,78	0	-1,42	-0,51	0,02	0,85	0	-1,32	-0,40	0
MAPKSP1	MAPK scaffold protein 1	0,92	-0,12	0,59	0,87	0	1,04	0,05	0,76	1,00	0	NA	NA	0
MIOS	missing oocyte, meiosis regulator, homolog (Drosophila)	0,92	-0,12	0,39	0,76	0	-1,04	-0,06	0,68	1,00	0	-1,18	-0,23	0
C6orf120	chromosome 6 open reading frame 120	0,92	-0,12	0,42	0,78	0	1,03	0,04	0,85	1,00	0	NA	NA	0
TTC19	tetratricopeptide repeat domain 19	0,92	-0,12	0,52	0,83	0	-1,08	-0,11	0,47	1,00	0	NA	NA	0
ATXN1	ataxin 1	0,92	-0,12	0,59	0,86	0	1,09	0,12	0,50	1,00	0	0,00	0,00	0
SERPINE1	serpin peptidase inhibitor, clade E (nexin, plasminogen activator inhibitor type 1), member 1	0,92	-0,12	0,71	0,91	0	-1,12	-0,16	0,27	1,00	0	NA	NA	0
MYBL1	v-myb myeloblastosis viral oncogene homolog (avian)-like 1	0,92	-0,12	0,70	0,91	0	-1,29	-0,36	0,20	1,00	0	NA	NA	0
PTPLB	protein tyrosine phosphatase-like (proline instead of catalytic arginine), member b	0,92	-0,12	0,50	0,82	0	1,03	0,05	0,53	1,00	0	NA	NA	0
WDHD1	WD repeat and HMG-box DNA binding protein 1	0,92	-0,12	0,54	0,84	0	-1,12	-0,16	0,47	1,00	0	-1,35	-0,43	0
HSPBAP1	HSPB (heat shock 27kDa) associated protein 1	0,92	-0,12	0,49	0,81	0	-1,17	-0,23	0,09	1,00	0	NA	NA	0
INSIG2	insulin induced gene 2	0,92	-0,12	0,47	0,80	0	1,02	0,03	0,84	1,00	0	NA	NA	0
TSKU	tsukushi small leucine rich proteoglycan homolog (Xenopus laevis)	0,92	-0,12	0,43	0,78	0	1,01	0,01	0,97	1,00	0	1,27	0,35	0
ABHD6	abhydrolase domain containing 6	0,92	-0,12	0,55	0,84	0	-1,44	-0,52	0,11	1,00	0	NA	NA	0
SRSF10	serine/arginine-rich splicing factor 10	0,92	-0,12	0,38	0,75	0	-1,04	-0,05	0,79	1,00	0	NA	NA	0
NAE1	NEDD8 activating enzyme E1 subunit 1	0,92	-0,12	0,43	0,78	0	1,01	0,01	0,89	1,00	0	NA	NA	0
EXOSC8	exosome component 8	0,92	-0,13	0,56	0,85	0	1,07	0,10	0,63	1,00	0	NA	NA	0
RXRA	retinoid X receptor, alpha	0,92	-0,13	0,49	0,81	0	-1,05	-0,07	0,71	1,00	0	NA	NA	0
MYST2	MYST histone acetyltransferase 2	0,92	-0,13	0,45	0,79	0	-1,21	-0,27	0,21	1,00	0	NA	NA	0
WDFY3	WD repeat and FYVE domain containing 3	0,92	-0,13	0,43	0,78	0	-1,16	-0,21	0,39	1,00	0	NA	NA	0
ANKFY1	ankyrin repeat and FYVE domain containing 1	0,92	-0,13	0,70	0,91	0	-1,16	-0,21	0,29	1,00	0	-1,11	-0,15	0
ELF4	E74-like factor 4 (ets domain transcription factor)	0,92	-0,13	0,63	0,88	0	1,13	0,18	0,32	1,00	0	NA	NA	0
EML1	echinoderm microtubule associated protein like 1	0,92	-0,13	0,57	0,85	0	1,19	0,25	0,26	1,00	0	1,29	0,37	0
RMI1	RMI1, RecQ mediated genome instability 1, homolog (S. cerevisiae)	0,92	-0,13	0,58	0,86	0	1,01	0,02	0,88	1,00	0	NA	NA	0
MAVS	mitochondrial antiviral signaling protein	0,92	-0,13	0,48	0,81	0	-1,20	-0,27	0,23	1,00	0	NA	NA	0
FARP1	FERM, RhoGEF (ARHGEF) and pleckstrin domain protein 1 (chondrocyte-derived)	0,92	-0,13	0,58	0,86	0	1,01	0,01	0,96	1,00	0	-1,01	-0,01	0
PHF10	PHD finger protein 10	0,92	-0,13	0,34	0,73	0	1,03	0,04	0,76	1,00	0	-1,25	-0,32	0
HNRNPA0	heterogeneous nuclear ribonucleoprotein A0	0,92	-0,13	0,33	0,72	0	1,01	0,02	0,93	1,00	0	-1,32	-0,39	0
UBE2I	ubiquitin-conjugating enzyme E2I (UBC9 homolog, yeast)	0,92	-0,13	0,52	0,83	0	-1,06	-0,08	0,71	1,00	0	-1,19	-0,25	0
SMARCC1	SWI/SNF related, matrix associated, actin dependent regulator of chromatin, subfamily c, member 1	0,92	-0,13	0,46	0,80	0	-1,01	-0,01	0,95	1,00	0	NA	NA	0
RSL1D1	ribosomal L1 domain containing 1	0,92	-0,13	0,47	0,80	0	-1,05	-0,07	0,84	1,00	0	NA	NA	0
PAM	peptidylglycine alpha-amidating monooxygenase	0,92	-0,13	0,44	0,79	0	1,11	0,15	0,14	1,00	0	NA	NA	0
EXOSC7	exosome component 7	0,92	-0,13	0,49	0,81	0	1,00	0,00	0,99	1,00	0	NA	NA	0
F12	coagulation factor XII (Hageman factor)	0,92	-0,13	0,54	0,84	0	-1,08	-0,11	0,70	1,00	0	NA	NA	0
GAS2L1	growth arrest-specific 2 like 1	0,91	-0,13	0,37	0,75	0	-1,03	-0,04	0,77	1,00	0	NA	NA	0
NHLRC2	NHL repeat containing 2	0,91	-0,13	0,63	0,88	0	-1,12	-0,16	0,39	1,00	0	1,03	0,05	0
AP1S2	adaptor-related protein complex 1, sigma 2 subunit	0,91	-0,13	0,63	0,88	0	1,02	0,02	NA	NA	0	-1,14	-0,19	0
DDX3X	DEAD (Asp-Glu-Ala-Asp) box polypeptide 3, X-linked	0,91	-0,13	0,44	0,78	0	-1,03	-0,04	0,94	1,00	0	-1,06	-0,09	0
HLA-G	major histocompatibility complex, class I, G	0,91	-0,13	0,66	0,89	0	1,00	0,00	0,99	1,00	0	1,06	0,09	0
IL1R1	interleukin 1 receptor, type I	0,91	-0,13	0,57	0,85	0	1,17	0,23	0,31	1,00	0	NA	NA	0
CHD9	chromodomain helicase DNA binding protein 9	0,91	-0,13	0,52	0,83	0	-1,14	-0,19	0,10	1,00	0	NA	NA	0
NAP1L1	nucleosome assembly protein 1-like 1	0,91	-0,13	0,31	0,70	0	-1,08	-0,11	0,28	1,00	0	NA	NA	0
WDR12	WD repeat domain 12	0,91	-0,13	0,58	0,86	0	1,00	0,00	1,00	1,00	0	NA	NA	0
PSMG1	proteasome (prosome, macropain) assembly chaperone 1	0,91	-0,13	0,49	0,81	0	-1,02	-0,03	0,82	1,00	0	NA	NA	0
S100PBP	S100P binding protein	0,91	-0,13	0,41	0,77	0	-1,17	-0,23	0,49	1,00	0	NA	NA	0
PABPC4	poly(A) binding protein, cytoplasmic 4 (inducible form)	0,91	-0,13	0,48	0,81	0	1,06	0,08	0,74	1,00	0	NA	NA	0
STAT5B	signal transducer and activator of transcription 5B	0,91	-0,13	0,54	0,84	0	-1,02	-0,03	0,76	1,00	0	1,11	0,15	0
ELOVL6	ELOVL family member 6, elongation of long chain fatty acids (FEN1/Elo2, SUR4/Elo3-like, yeast)	0,91	-0,13	0,37	0,75	0	-1,04	-0,06	0,67	1,00	0	NA	NA	0
FBXO21	F-box protein 21	0,91	-0,13	0,47	0,80	0	-1,04	-0,06	0,87	1,00	0	NA	NA	0
PYCR1	pyrroline-5-carboxylate reductase 1	0,91	-0,13	0,49	0,81	0	-1,09	-0,12	0,50	1,00	0	1,16	0,21	0
PILRB	paired immunoglobin-like type 2 receptor beta	0,91	-0,13	0,53	0,83	0	-1,16	-0,21	0,34	1,00	0	NA	NA	0
PHTF2	putative homeodomain transcription factor 2	0,91	-0,13	0,54	0,84	0	-1,38	-0,46	0,34	1,00	0	-1,43	-0,52	0
TNFAIP8	tumor necrosis factor, alpha-induced protein 8	0,91	-0,13	0,44	0,79	0	-1,09	-0,12	0,14	1,00	0	NA	NA	0
ASMTL	acetylserotonin O-methyltransferase-like	0,91	-0,13	0,39	0,75	0	-1,17	-0,23	0,35	1,00	0	1,02	0,03	0
MTFR1	mitochondrial fission regulator 1	0,91	-0,13	0,45	0,79	0	1,02	0,03	0,82	1,00	0	-1,28	-0,35	0
SERPINB6	serpin peptidase inhibitor, clade B (ovalbumin), member 6	0,91	-0,13	0,60	0,87	0	-1,02	-0,02	0,86	1,00	0	NA	NA	0
NA	NA	0,91	-0,13	0,38	0,75	0	-1,11	-0,15	0,41	1,00	0	NA	NA	0
FBXO11	F-box protein 11	0,91	-0,13	0,45	0,79	0	-1,22	-0,28	0,30	1,00	0	NA	NA	0
CA12	carbonic anhydrase XII	0,91	-0,13	0,49	0,81	0	-1,01	-0,02	0,89	1,00	0	NA	NA	0
MAP4K4	mitogen-activated protein kinase kinase kinase kinase 4	0,91	-0,13	0,43	0,78	0	-1,17	-0,23	0,14	1,00	0	NA	NA	0
NA	NA	0,91	-0,13	0,69	0,90	0	1,14	0,19	0,53	1,00	0	NA	NA	0
PIK3CB	phosphoinositide-3-kinase, catalytic, beta polypeptide	0,91	-0,13	0,28	0,68	0	-1,01	-0,02	0,90	1,00	0	-1,27	-0,33	0
ZNF331	zinc finger protein 331	0,91	-0,13	0,77	0,93	0	-1,06	-0,09	0,59	1,00	0	NA	NA	0
PPP3CA	protein phosphatase 3, catalytic subunit, alpha isozyme	0,91	-0,13	0,44	0,78	0	-1,11	-0,15	0,14	1,00	0	NA	NA	0
VAMP8	vesicle-associated membrane protein 8 (endobrevin)	0,91	-0,13	0,50	0,82	0	1,14	0,19	0,26	1,00	0	NA	NA	0
ACOT11	acyl-CoA thioesterase 11	0,91	-0,14	0,57	0,85	0	1,20	0,27	0,37	1,00	0	NA	NA	0
FAM168A	family with sequence similarity 168, member A	0,91	-0,14	0,55	0,85	0	-1,01	-0,02	0,90	1,00	0	-1,27	-0,35	0
APOL3	apolipoprotein L, 3	0,91	-0,14	0,62	0,88	0	-1,01	-0,02	0,79	1,00	0	-1,18	-0,24	0
STK3	serine/threonine kinase 3	0,91	-0,14	0,28	0,68	0	-1,03	-0,04	0,72	1,00	0	NA	NA	0
ZMAT5	zinc finger, matrin type 5	0,91	-0,14	0,61	0,87	0	1,00	0,00	0,99	1,00	0	NA	NA	0
DBN1	drebrin 1	0,91	-0,14	0,65	0,89	0	-1,09	-0,12	0,76	1,00	0	NA	NA	0
SNX4	sorting nexin 4	0,91	-0,14	0,44	0,79	0	1,08	0,11	0,78	1,00	0	-1,32	-0,40	0
UBR2	ubiquitin protein ligase E3 component n-recognin 2	0,91	-0,14	0,44	0,78	0	-1,05	-0,07	0,62	1,00	0	0,00	0,00	0
PRRG1	proline rich Gla (G-carboxyglutamic acid) 1	0,91	-0,14	0,47	0,81	0	-1,07	-0,10	0,44	1,00	0	NA	NA	0
SLBP	stem-loop binding protein	0,91	-0,14	0,37	0,75	0	-1,00	-0,01	0,94	1,00	0	NA	NA	0
TSPAN31	tetraspanin 31	0,91	-0,14	0,46	0,80	0	1,02	0,03	0,90	1,00	0	NA	NA	0
TPST1	tyrosylprotein sulfotransferase 1	0,91	-0,14	0,36	0,74	0	-1,05	-0,07	0,84	1,00	0	NA	NA	0
COL15A1	collagen, type XV, alpha 1	0,91	-0,14	0,78	0,94	0	-1,00	-0,01	NA	NA	0	-1,10	-0,14	0
LY6E	lymphocyte antigen 6 complex, locus E	0,91	-0,14	0,80	0,95	0	-1,33	-0,41	0,16	1,00	0	1,30	0,38	0
EPCAM	epithelial cell adhesion molecule	0,91	-0,14	0,76	0,93	0	1,20	0,27	0,02	0,84	0	NA	NA	0
TBC1D2B	TBC1 domain family, member 2B	0,91	-0,14	0,51	0,82	0	-1,02	-0,03	0,86	1,00	0	NA	NA	0
TTC13	tetratricopeptide repeat domain 13	0,91	-0,14	0,35	0,73	0	1,07	0,10	0,55	1,00	0	NA	NA	0
RAB32	RAB32, member RAS oncogene family	0,91	-0,14	0,58	0,86	0	1,11	0,15	0,36	1,00	0	1,10	0,14	0
NA	NA	0,91	-0,14	0,46	0,80	0	1,05	0,08	0,70	1,00	0	NA	NA	0
RARRES1	retinoic acid receptor responder (tazarotene induced) 1	0,91	-0,14	0,82	0,95	0	-1,01	-0,01	0,99	1,00	0	0,00	0,00	0
FERMT2	fermitin family member 2	0,91	-0,14	0,74	0,92	0	-1,08	-0,11	0,67	1,00	0	-1,12	-0,17	0
ADI1	acireductone dioxygenase 1	0,91	-0,14	0,39	0,76	0	1,11	0,15	0,11	1,00	0	-1,06	-0,09	0
ITGB6	integrin, beta 6	0,91	-0,14	0,91	0,98	0	-1,45	-0,53	0,65	1,00	0	NA	NA	0

SLC35D1	solute carrier family 35 (UDP-glucuronic acid/UDP-N-acetylgalactosamine dual transporter), member D1	0,91	-0,14	0,57	0,86	0	1,02	0,02	0,95	1,00	0	NA	NA	0
TRIM24	tripartite motif-containing 24	0,91	-0,14	0,31	0,70	0	1,05	0,07	0,72	1,00	0	NA	NA	0
ICAM1	intercellular adhesion molecule 1	0,91	-0,14	0,71	0,91	0	-1,05	-0,07	0,70	1,00	0	1,44	0,53	0
TNS1	tensin 1	0,91	-0,14	0,41	0,77	0	-1,00	0,00	1,00	1,00	0	-1,01	-0,02	0
SIGIRR	single immunoglobulin and toll-interleukin 1 receptor (TIR) domain	0,91	-0,14	0,69	0,90	0	-1,02	-0,03	0,78	1,00	0	1,14	0,19	0
MAP3K5	mitogen-activated protein kinase kinase kinase 5	0,91	-0,14	0,53	0,84	0	-1,10	-0,14	0,48	1,00	0	NA	NA	0
CXADR	coxsackie virus and adenovirus receptor	0,91	-0,14	0,58	0,86	0	1,03	0,04	0,71	1,00	0	NA	NA	0
ASS1	argininosuccinate synthase 1	0,91	-0,14	0,69	0,90	0	-1,12	-0,16	0,59	1,00	0	NA	NA	0
HPS1	Hermansky-Pudlak syndrome 1	0,91	-0,14	0,34	0,72	0	1,18	0,24	0,12	1,00	0	NA	NA	0
NA	NA	0,91	-0,14	0,43	0,78	0	1,05	0,07	0,77	1,00	0	-1,09	-0,12	0
FSTL3	follistatin-like 3 (secreted glycoprotein)	0,91	-0,14	0,63	0,88	0	1,04	0,06	0,77	1,00	0	1,36	0,44	0
AGR2	anterior gradient homolog 2 (Xenopus laevis)	0,91	-0,14	0,85	0,96	0	-1,07	-0,10	0,83	1,00	0	NA	NA	0
SLC5A3	solute carrier family 5 (sodium/myo-inositol cotransporter), member 3	0,91	-0,14	0,63	0,88	0	1,15	0,20	0,12	1,00	0	-1,02	-0,03	0
TNFRSF10B	tumor necrosis factor receptor superfamily, member 10b	0,91	-0,14	0,59	0,87	0	-1,09	-0,12	0,76	1,00	0	-1,09	-0,12	0
SNAP23	synaptosomal-associated protein, 23kDa	0,91	-0,14	0,30	0,69	0	-1,01	-0,01	0,98	1,00	0	-1,22	-0,28	0
LAGE3	L antigen family, member 3	0,91	-0,14	0,57	0,85	0	1,01	0,02	0,92	1,00	0	-1,04	-0,06	0
SLFN12	schlafen family member 12	0,91	-0,14	0,52	0,83	0	-1,25	-0,32	0,20	1,00	0	-1,37	-0,46	0
LSS	lanosterol synthase (2,3-oxidosqualene-lanosterol cyclase)	0,91	-0,14	0,57	0,85	0	1,01	0,01	0,96	1,00	0	NA	NA	0
CTSS	cathepsin S	0,91	-0,14	0,75	0,93	0	-1,03	-0,04	0,93	1,00	0	1,12	0,16	0
UBR2	ubiquitin protein ligase E3 component n-recognin 2	0,91	-0,14	0,44	0,78	0	1,15	0,20	0,17	1,00	0	-1,37	-0,45	0
CDS2	CDP-diacylglycerol synthase (phosphatidate cytidylyltransferase) 2	0,91	-0,14	0,33	0,72	0	1,24	0,32	0,21	1,00	0	NA	NA	0
DDIT4	DNA-damage-inducible transcript 4	0,91	-0,14	0,28	0,68	0	-1,27	-0,35	0,01	0,78	0	1,16	0,22	0
ERBB2	v-erb-b2 erythroblastic leukemia viral oncogene homolog 2, neuro/glioblastoma derived oncogene homolog (avian)	0,91	-0,14	0,57	0,86	0	-1,22	-0,28	0,03	1,00	0	NA	NA	0
TFPI2	tissue factor pathway inhibitor 2	0,91	-0,14	0,68	0,90	0	-1,57	-0,65	0,06	1,00	0	-1,06	-0,09	0
PDGFA	platelet-derived growth factor alpha polypeptide	0,91	-0,14	0,45	0,79	0	-1,10	-0,14	0,82	1,00	0	-1,09	-0,11	0
COG2	component of oligomeric golgi complex 2	0,91	-0,14	0,40	0,76	0	-1,03	-0,04	0,70	1,00	0	-1,30	-0,37	0
MICA	MHC class I polypeptide-related sequence A	0,91	-0,14	0,31	0,70	0	1,12	0,17	0,25	1,00	0	NA	NA	0
BPHL	biphenyl hydrolase-like (serine hydrolase)	0,91	-0,14	0,46	0,80	0	-1,13	-0,18	0,29	1,00	0	-1,16	-0,22	0
GBP4	guanylate binding protein 4	0,91	-0,14	0,85	0,96	0	NA	NA	NA	NA	0	NA	NA	0
KDM5B	lysine (K)-specific demethylase 5B	0,90	-0,14	0,44	0,79	0	-1,08	-0,11	0,36	1,00	0	-1,06	-0,08	0
ANXA6	annexin A6	0,90	-0,14	0,62	0,88	0	-1,19	-0,25	0,52	1,00	0	NA	NA	0
ABCB6	ATP-binding cassette, sub-family B (MDR/TAP), member 6	0,90	-0,14	0,53	0,83	0	1,06	0,09	0,67	1,00	0	NA	NA	0
RNF114	ring finger protein 114	0,90	-0,14	0,39	0,75	0	1,09	0,13	0,28	1,00	0	-1,15	-0,20	0
ADAR	adenosine deaminase, RNA-specific	0,90	-0,14	0,37	0,74	0	-1,07	-0,09	0,28	1,00	0	-1,16	-0,22	0
TNFRSF21	tumor necrosis factor receptor superfamily, member 21	0,90	-0,14	0,42	0,78	0	1,02	0,03	0,80	1,00	0	NA	NA	0
TSEN2	tRNA splicing endonuclease 2 homolog (S. cerevisiae)	0,90	-0,14	0,52	0,83	0	-1,07	-0,10	0,65	1,00	0	NA	NA	0
TTC3	tetratricopeptide repeat domain 3	0,90	-0,14	0,33	0,72	0	-1,06	-0,08	0,44	1,00	0	-1,05	-0,07	0
UBR7	ubiquitin protein ligase E3 component n-recognin 7 (putative)	0,90	-0,14	0,33	0,71	0	-1,04	-0,06	0,65	1,00	0	NA	NA	0
PIP4K2C	phosphatidylinositol-5-phosphate 4-kinase, type II, gamma	0,90	-0,14	0,35	0,73	0	1,06	0,08	0,62	1,00	0	NA	NA	0
ARHGAP29	Rho GTPase activating protein 29	0,90	-0,15	0,63	0,88	0	-1,12	-0,17	0,18	1,00	0	NA	NA	0
STAM2	signal transducing adaptor molecule (SH3 domain and ITAM motif) 2	0,90	-0,15	0,48	0,81	0	1,13	0,18	0,27	1,00	0	NA	NA	0
FAM190B	family with sequence similarity 190, member B	0,90	-0,15	0,43	0,78	0	1,10	0,14	0,42	1,00	0	NA	NA	0
NPAT	nuclear protein, ataxia-telangiectasia locus	0,90	-0,15	0,43	0,78	0	-1,16	-0,21	0,31	1,00	0	NA	NA	0
HSPG2	heparan sulfate proteoglycan 2	0,90	-0,15	0,69	0,90	0	-1,04	-0,06	0,82	1,00	0	NA	NA	0
BPGM	2,3-bisphosphoglycerate mutase	0,90	-0,15	0,55	0,84	0	1,22	0,28	0,15	1,00	0	NA	NA	0
BRD8	bromodomain containing 8	0,90	-0,15	0,38	0,75	0	1,02	0,03	0,75	1,00	0	NA	NA	0
TLE4	transducin-like enhancer of split 4 (E(sp1) homolog, Drosophila)	0,90	-0,15	0,30	0,69	0	-1,07	-0,10	0,33	1,00	0	NA	NA	0
PRKRIR	protein-kinase, interferon-inducible double stranded RNA dependent inhibitor, repressor of (P58 repressor)	0,90	-0,15	0,40	0,77	0	1,06	0,08	0,35	1,00	0	-1,19	-0,25	0
TCF4	transcription factor 4	0,90	-0,15	0,62	0,88	0	-1,17	-0,22	0,53	1,00	0	NA	NA	0
HLA-C	major histocompatibility complex, class I, C	0,90	-0,15	0,37	0,75	0	-1,11	-0,15	0,22	1,00	0	-1,02	-0,03	0
NACAD	NAC alpha domain containing	0,90	-0,15	0,51	0,82	0	-1,01	-0,02	0,94	1,00	0	NA	NA	0
TDP1	tyrosyl-DNA phosphodiesterase 1	0,90	-0,15	0,46	0,80	0	-1,01	-0,02	0,88	1,00	0	NA	NA	0
CPS1	carbamoyl-phosphate synthase 1, mitochondrial	0,90	-0,15	0,49	0,81	0	-1,43	-0,52	0,12	1,00	0	NA	NA	0
KRT8	keratin 8	0,90	-0,15	0,86	0,96	0	-1,15	-0,20	0,34	1,00	0	NA	NA	0
LOC283357	hypothetical protein LOC283357	0,90	-0,15	0,45	0,79	0	NA	NA	NA	NA	0	NA	NA	0
NA	NA	0,90	-0,15	0,30	0,69	0	-1,07	-0,10	0,33	1,00	0	NA	NA	0
NFIB	nuclear factor I/B	0,90	-0,15	0,47	0,81	0	-1,06	-0,08	0,64	1,00	0	1,04	0,05	0
MYH10	myosin, heavy chain 10, non-muscle	0,90	-0,15	0,39	0,76	0	-1,08	-0,12	0,19	1,00	0	1,02	0,02	0
TPD52	tumor protein D52	0,90	-0,15	0,60	0,87	0	1,02	0,03	0,88	1,00	0	-1,45	-0,53	0
SLC35E2B	solute carrier family 35, member E2B	0,90	-0,15	0,38	0,75	0	-1,03	-0,04	0,77	1,00	0	NA	NA	0
BBX	bobby sox homolog (Drosophila)	0,90	-0,15	0,69	0,90	0	-1,26	-0,33	0,33	1,00	0	NA	NA	0
TMEM229B	transmembrane protein 229B	0,90	-0,15	0,72	0,91	0	NA	NA	NA	NA	0	NA	NA	0
SIK2	salt-inducible kinase 2	0,90	-0,15	0,43	0,78	0	-1,15	-0,21	0,18	1,00	0	1,13	0,18	0
PEX7	peroxisomal biogenesis factor 7	0,90	-0,15	0,33	0,71	0	-1,17	-0,23	0,30	1,00	0	-1,45	-0,53	0
ENDOG	endonuclease G	0,90	-0,15	0,56	0,85	0	1,02	0,03	0,91	1,00	0	-1,16	-0,22	0
NACC2	NACC family member 2, BEN and BTB (POZ) domain containing	0,90	-0,15	0,44	0,78	0	1,17	0,23	0,41	1,00	0	1,05	0,07	0
MTMR4	myotubularin related protein 4	0,90	-0,15	0,28	0,68	0	-1,17	-0,23	0,18	1,00	0	NA	NA	0
CENPQ	centromere protein Q	0,90	-0,15	0,57	0,85	0	-1,04	-0,05	0,79	1,00	0	NA	NA	0
CLCA2	chloride channel accessory 2	0,90	-0,15	0,52	0,83	0	-1,19	-0,25	0,09	1,00	0	NA	NA	0
VKORC1	vitamin K epoxide reductase complex, subunit 1	0,90	-0,15	0,53	0,84	0	-1,11	-0,15	0,30	1,00	0	NA	NA	0
LOC100129250	hypothetical LOC100129250	0,90	-0,15	0,58	0,86	0	-1,24	-0,31	0,37	1,00	0	-1,28	-0,36	0
POLE3	polymerase (DNA directed), epsilon 3 (p17 subunit)	0,90	-0,15	0,56	0,85	0	1,06	0,08	0,54	1,00	0	NA	NA	0
MGA	MAX gene associated	0,90	-0,15	0,33	0,71	0	-1,21	-0,28	0,42	1,00	0	NA	NA	0
TAPBPL	TAP binding protein-like	0,90	-0,15	0,60	0,87	0	-1,02	-0,02	0,93	1,00	0	-1,08	-0,10	0
H2AFY2	H2A histone family, member Y2	0,90	-0,15	0,30	0,69	0	-1,03	-0,05	0,71	1,00	0	NA	NA	0
HLTF	helicase-like transcription factor	0,90	-0,15	0,48	0,81	0	-1,21	-0,28	0,25	1,00	0	-1,28	-0,36	0
CDC42EP4	CDC42 effector protein (Rho GTPase binding) 4	0,90	-0,15	0,37	0,75	0	-1,08	-0,11	0,42	1,00	0	NA	NA	0
PCNA	proliferating cell nuclear antigen	0,90	-0,15	0,46	0,79	0	-1,01	-0,02	0,87	1,00	0	NA	NA	0
HLA-J	major histocompatibility complex, class I, J (pseudogene)	0,90	-0,15	0,35	0,73	0	-1,14	-0,19	0,36	1,00	0	1,09	0,13	0
SOCS5	suppressor of cytokine signaling 5	0,90	-0,15	0,39	0,76	0	1,05	0,07	0,59	1,00	0	NA	NA	0
ATG5	ATG5 autophagy related 5 homolog (S. cerevisiae)	0,90	-0,15	0,51	0,83	0	-1,09	-0,12	0,56	1,00	0	-1,32	-0,40	0
LIG1	ligase I, DNA, ATP-dependent	0,90	-0,15	0,43	0,78	0	-1,17	-0,23	0,18	1,00	0	NA	NA	0
HECTD3	HECT domain containing 3	0,90	-0,15	0,33	0,71	0	1,06	0,09	0,64	1,00	0	NA	NA	0
TSTA3	tissue specific transplantation antigen P35B	0,90	-0,15	0,41	0,77	0	1,00	0,00	0,97	1,00	0	NA	NA	0
FAM119B	family with sequence similarity 119, member B	0,90	-0,15	0,53	0,84	0	-1,17	-0,22	0,20	1,00	0	-1,49	-0,57	0
RHOQ	ras homolog gene family, member Q	0,90	-0,15	0,66	0,89	0	1,07	0,10	0,63	1,00	0	-1,02	-0,03	0
IFI16	interferon, gamma-inducible protein 16	0,90	-0,15	0,35	0,73	0	-1,00	0,00	0,99	1,00	0	-1,39	-0,48	0
TMEM38B	transmembrane protein 38B	0,90	-0,15	0,69	0,91	0	-1,01	-0,02	0,93	1,00	0	-1,30	-0,37	0
COL5A1	collagen, type V, alpha 1	0,90	-0,15	0,67	0,90	0	-1,01	-0,01	NA	NA	0	-1,06	-0,08	0
UTP18	UTP18, small subunit (SSU) processome component, homolog (yeast)	0,90	-0,15	0,36	0,74	0	-1,06	-0,09	0,39	1,00	0	NA	NA	0
VPS54	vacuolar protein sorting 54 homolog (S. cerevisiae)	0,90	-0,15	0,31	0,70	0	1,10	0,14	0,25	1,00	0	NA	NA	0
PPT1	palmitoyl-protein thioesterase 1	0,90	-0,15	0,37	0,74	0	-1,05	-0,07	0,47	1,00	0	NA	NA	0
NA	NA	0,90	-0,15	0,33	0,72	0	-1,30	-0,38	0,05	1,00	0	-1,14	-0,19	0
AUTS2	autism susceptibility candidate 2	0,90	-0,15	0,48	0,81	0	-1,24	-0,31	0,04	1,00	0	NA	NA	0
NA	NA	0,90	-0,15	0,59	0,87	0	-1,06	-0,08	0,76	1,00	0	-1,20	-0,26	0
CDK14	cyclin-dependent kinase 14	0,90	-0,16	0,35	0,73	0	-1,26	-0,34	0,14	1,00	0	NA	NA	0
CIR1	corepressor interacting with RBPJ, 1	0,90	-0,16	0,48	0,81	0	1,04	0,05	0,68	1,00	0	-1,10	-0,13	0
ZNF532	zinc finger protein 532	0,90	-0,16	0,71	0,91	0	-1,02	-0,04	0,75	1,00	0	-1,39	-0,47	0
TCTN3	tectonic family member 3	0,90	-0,16	0,38	0,75	0	1,06	0,08	0,62	1,00	0	NA	NA	0
GGA2	golgi-associated, gamma adaptin ear containing, ARF binding protein 2	0,90	-0,16	0,35	0,73	0	-1,54	-0,62	0,16	1,00	0	NA	NA	0
MDM2	Mdm2 p53 binding protein homolog (mouse)	0,90	-0,16	0,42	0,78	0	-1,01	-0,02	0,94	1,00	0	NA	NA	0
IFITM3	interferon induced transmembrane protein 3 (1-8U)	0,90	-0,16	0,43	0,78	0	-1,06	-0,09	0,62	1,00	0	-1,19	-0,25	0
WASF1	WAS protein family, member 1	0,90	-0,16	0,38	0,75	0	-1,07	-0,10	0,50	1,00	0	NA	NA	0
ELTD1	EGF, latrophilin and seven transmembrane domain containing 1	0,90	-0,16	0,63	0,88	0	-1,02	-0,03	NA	NA	0	-1,27	-0,33	0
MLF1	myeloid leukemia factor 1	0,90	-0,16	0,38	0,75	0	1,40	0,48	0,04	1,00	0	NA	NA	0
ANGEL1	angel homolog 1 (Drosophila)	0,90	-0,16	0,25	0,66	0	-1,27	-0,34	0,06	1,00	0	NA	NA	0
NECAP2	NECAP endocytosis associated 2	0,90	-0,16	0,34	0,72	0	-1,02	-0,03	0,73	1,00	0	NA	NA	0
SLIT2	slit homolog 2 (Drosophila)	0,90	-0,16	0,45	0,79	0	-1,06	-0,08	0,74	1,00	0	-1,32	-0,40	0
SENP6	SUMO1/sentrin specific peptidase 6	0,90	-0,16	0,62	0,88	0	-1,17	-0,22	0,59	1,00	0	-1,75	-0,80	0
STAU2	staufen, RNA binding protein, homolog 2 (Drosophila)	0,90	-0,16	0,33	0,72	0	1,28	0,36	0,09	1,00	0	-1,72	-0,79	0
VAMP5	vesicle-associated membrane protein 5 (myobrevin)	0,90	-0,16	0,40	0,76	0	-1,00	-0,01	0,95	1,00	0	-1,16	-0,21	0
RFC2	replication factor C (activator 1) 2, 40kDa	0,90	-0,16	0,50	0,82	0	-1,08	-0,11	0,41	1,00	0	NA	NA	0
TM2D1	TM2 domain containing 1	0,90	-0,16	0,60	0,87	0	1,00	0,00	NA	NA	0	-1,15	-0,20	0
LPIN1	lipin 1	0,90	-0,16	0,61	0,87	0	1,05	0,07	0,60	1,00	0	NA	NA	0
KDM5B	lysine (K)-specific demethylase 5B	0,90	-0,16	0,39	0,75	0	-1,11	-0,14	0,22	1,00	0	-1,04	-0,06	0
DTWD1	DTW domain containing 1	0,90	-0,16	0,57	0,85	0	-1,10	-0,14	0,47	1,00	0	NA	NA	0
UBA6	ubiquitin-like modifier activating enzyme 6	0,90	-0,16	0,69	0,91	0	1,27	0,34	0,56	1,00	0	NA	NA	0
NUCKS1	nuclear casein kinase and cyclin-dependent kinase substrate 1	0,90	-0,16	0,22	0,62	0	-1,02	-0,03	0,81	1,00	0	NA	NA	0
DSTYK	dual serine/threonine and tyrosine protein kinase	0,90	-0,16	0,45	0,79	0	-1,18	-0,23	0,23	1,00	0	NA	NA	0
FOXD1	forkhead box D1	0,90	-0,16	0,45	0,79	0	1,10	0,14	0,48	1,00	0	-1,11	-0,15	0
HFE	hemochromatosis	0,90	-0,16	0,28	0,68	0	-1,19	-0,25	0,18	1,00	0	NA	NA	0
ATP7B	ATPase, Cu++ transporting, beta polypeptide	0,90	-0,16	0,58	0,86	0	-1,00	0,00	0,99	1,00	0	NA	NA	0
FHL1	four and a half LIM domains 1	0,89	-0,16	0,61	0,87	0	-1,21	-0,27	0,29	1,00	0	1,05	0,07	0
NA	NA	0,89	-0,16	0,55	0,85	0	NA	NA	NA	NA	0	NA	NA	0
HIST1H2AC	histone cluster 1, H2ac	0,89	-0,16	0,36	0,73	0	1,18	0,24	0,23	1,00	0	NA	NA	0
OGT	O-linked N-acetylglucosamine (GlcNAc) transferase (UDP-N-acetylglucosamine:polypeptide-N-acetylglucosaminyl transferase)	0,89	-0,16	0,55	0,85	0	-1,26	-0,34	0,14	1,00	0	0,00	0,00	0
NCRNA00094	non-protein coding RNA 94	0,89	-0,16	0,45	0,79	0	1,07	0,09	0,69	1,00	0	-1,18	-0,23	0
CEP135	centrosomal protein 135kDa	0,89	-0,16	0,38	0,75	0	-1,19	-0,26	0,04	1,00	0	-1,19	-0,25	0
HEPH	hephaestin	0,89	-0,16	0,56	0,85	0	-1,00	-0,01	NA	NA	0	-1,64	-0,72	0
UPF3B	UPF3 regulator of nonsense transcripts homolog B (yeast)	0,89	-0,16	0,25	0,65	0	1,05	0,06	0,69	1,00	0	NA	NA	0
FNBP4	formin binding protein 4	0,89	-0,16	0,23	0,64	0	1,17	0,23	0,16	1,00	0	-1,11	-0,16	0
IL18R1	interleukin 18 receptor 1	0,89	-0,16	0,49	0,81	0	-1,01	-0,02	0,96	1,00	0	NA	NA	0
TMEM80	transmembrane protein 80	0,89	-0,16	0,49	0,81	0	1,05	0,07	0,74	1,00	0	-1,16	-0,22	0
IRF2	interferon regulatory factor 2	0,89	-0,16	0,40	0,76	0	1,05	0,07	0,45	1,00	0	1,16	0,22	0
ALDH4A1	aldehyde dehydrogenase 4 family, member A1	0,89	-0,16	0,55	0,85	0	-1,31	-0,39	0,19	1,00	0	1,07	0,09	0
SRSF7	serine/arginine-rich splicing factor 7	0,89	-0,16	0,30	0,70	0	1,03	0,05	0,75	1,00	0	NA	NA	0
PPP1R8	protein phosphatase 1, regulatory (inhibitor) subunit 8	0,89	-0,16	0,38	0,75	0	-1,02	-0,03	0,82	1,00	0	NA	NA	0

HLA-C	major histocompatibility complex, class I, C	0,89	-0,16	0,34	0,72	0	-1,11	-0,16	0,30	1,00	0	-1,03	-0,05	0
PAN2	PAN2 poly(A) specific ribonuclease subunit homolog (S. cerevisiae)	0,89	-0,16	0,46	0,80	0	-1,03	-0,04	0,83	1,00	0	NA	NA	0
ENOSF1	enolase superfamily member 1	0,89	-0,16	0,44	0,78	0	-1,03	-0,04	0,81	1,00	0	NA	NA	0
TNS3	tensin 3	0,89	-0,16	0,48	0,81	0	-1,02	-0,03	0,95	1,00	0	NA	NA	0
SOX4	SRY (sex determining region Y)-box 4	0,89	-0,16	0,63	0,88	0	1,08	0,11	0,56	1,00	0	-1,47	-0,56	0
RCN2	reticulocalbin 2, EF-hand calcium binding domain	0,89	-0,16	0,51	0,82	0	-1,09	-0,12	0,14	1,00	0	NA	NA	0
WDR25	WD repeat domain 25	0,89	-0,16	0,30	0,69	0	-1,01	-0,01	0,95	1,00	0	0,00	0,00	0
FLOT2	flotillin 2	0,89	-0,16	0,59	0,86	0	-1,42	-0,51	0,00	0,18	0	NA	NA	0
HIRIP3	HIRA interacting protein 3	0,89	-0,16	0,26	0,66	0	-1,00	0,00	0,99	1,00	0	NA	NA	0
AGBL5	ATP/GTP binding protein-like 5	0,89	-0,16	0,48	0,81	0	-1,13	-0,18	0,28	1,00	0	-1,03	-0,05	0
IRS1	insulin receptor substrate 1	0,89	-0,16	0,44	0,78	0	-1,08	-0,11	0,42	1,00	0	NA	NA	0
LAMC1	laminin, gamma 1 (formerly LAMB2)	0,89	-0,16	0,38	0,75	0	-1,10	-0,13	0,18	1,00	0	NA	NA	0
FADS3	fatty acid desaturase 3	0,89	-0,16	0,56	0,85	0	-1,01	-0,02	0,96	1,00	0	NA	NA	0
HNRPDL	heterogeneous nuclear ribonucleoprotein D-like	0,89	-0,16	0,45	0,79	0	-1,20	-0,26	0,10	1,00	0	NA	NA	0
HAUS6	HAUS augmin-like complex, subunit 6	0,89	-0,16	0,34	0,72	0	-1,08	-0,11	0,65	1,00	0	NA	NA	0
CBY1	chibby homolog 1 (Drosophila)	0,89	-0,16	0,28	0,68	0	-1,01	-0,02	0,92	1,00	0	NA	NA	0
TOMM70A	translocase of outer mitochondrial membrane 70 homolog A (S. cerevisiae)	0,89	-0,16	0,38	0,75	0	-1,03	-0,04	0,76	1,00	0	NA	NA	0
INPP5A	inositol polyphosphate-5-phosphatase, 40kDa	0,89	-0,16	0,34	0,72	0	1,04	0,06	0,74	1,00	0	-1,11	-0,16	0
HSDL2	hydroxysteroid dehydrogenase like 2	0,89	-0,17	0,43	0,78	0	-1,09	-0,13	0,46	1,00	0	-1,19	-0,26	0
RBCK1	RanBP-type and C3HC4-type zinc finger containing 1	0,89	-0,17	0,48	0,81	0	-1,04	-0,06	0,83	1,00	0	1,16	0,21	0
SLC25A14	solute carrier family 25 (mitochondrial carrier, brain), member 14	0,89	-0,17	0,41	0,77	0	1,18	0,24	0,27	1,00	0	-1,19	-0,26	0
NA	NA	0,89	-0,17	0,41	0,77	0	1,00	0,00	1,00	1,00	0	NA	NA	0
PLOD2	procollagen-lysine, 2-oxoglutarate 5-dioxygenase 2	0,89	-0,17	0,51	0,82	0	1,11	0,15	0,45	1,00	0	NA	NA	0
BTN2A1	butyrophilin, subfamily 2, member A1	0,89	-0,17	0,52	0,83	0	1,00	0,00	0,98	1,00	0	NA	NA	0
PTPRF	protein tyrosine phosphatase, receptor type, F	0,89	-0,17	0,58	0,86	0	-1,09	-0,12	0,35	1,00	0	1,12	0,17	0
STX12	syntaxin 12	0,89	-0,17	0,19	0,60	0	1,12	0,16	0,24	1,00	0	NA	NA	0
PHTF1	putative homeodomain transcription factor 1	0,89	-0,17	0,55	0,85	0	-1,28	-0,36	0,20	1,00	0	NA	NA	0
SFPQ	splicing factor proline/glutamine-rich	0,89	-0,17	0,41	0,77	0	-1,07	-0,09	0,70	1,00	0	NA	NA	0
COX11	COX11 cytochrome c oxidase assembly homolog (yeast)	0,89	-0,17	0,35	0,73	0	1,11	0,15	0,63	1,00	0	-1,09	-0,12	0
RNF114	ring finger protein 114	0,89	-0,17	0,37	0,74	0	-1,06	-0,08	0,51	1,00	0	-1,11	-0,16	0
HLA-A	major histocompatibility complex, class I, A	0,89	-0,17	0,21	0,62	0	-1,10	-0,14	0,23	1,00	0	-1,01	-0,01	0
C9orf91	chromosome 9 open reading frame 91	0,89	-0,17	0,39	0,76	0	-1,17	-0,22	0,48	1,00	0	0,00	0,00	0
NUP37	nucleoporin 37kDa	0,89	-0,17	0,39	0,76	0	1,06	0,09	0,67	1,00	0	NA	NA	0
CUTA	cutA divalent cation tolerance homolog (E. coli)	0,89	-0,17	0,23	0,63	0	-1,16	-0,21	0,08	1,00	0	NA	NA	0
PCMTD2	protein-L-isoaspartate (D-aspartate) O-methyltransferase domain containing 2	0,89	-0,17	0,43	0,78	0	-1,04	-0,05	0,82	1,00	0	-1,43	-0,52	0
ANK3	ankyrin 3, node of Ranvier (ankyrin G)	0,89	-0,17	0,54	0,84	0	-1,05	-0,06	0,81	1,00	0	NA	NA	0
C19orf60	chromosome 19 open reading frame 60	0,89	-0,17	0,34	0,72	0	-1,19	-0,25	0,12	1,00	0	NA	NA	0
C17orf62	chromosome 17 open reading frame 62	0,89	-0,17	0,35	0,73	0	-1,17	-0,23	0,22	1,00	0	NA	NA	0
COPS7A	COP9 constitutive photomorphogenic homolog subunit 7A (Arabidopsis)	0,89	-0,17	0,22	0,63	0	-1,17	-0,23	0,25	1,00	0	NA	NA	0
AHI1	Abelson helper integration site 1	0,89	-0,17	0,49	0,81	0	-1,09	-0,13	0,54	1,00	0	NA	NA	0
METTL3	methyltransferase like 3	0,89	-0,17	0,56	0,85	0	-1,13	-0,18	0,48	1,00	0	NA	NA	0
TMX1	thioredoxin-related transmembrane protein 1	0,89	-0,17	0,45	0,79	0	1,02	0,03	0,78	1,00	0	-1,05	-0,07	0
SENP6	SUMO1/sentrin specific peptidase 6	0,89	-0,17	0,39	0,76	0	-1,04	-0,06	0,87	1,00	0	NA	NA	0
HLA-F	major histocompatibility complex, class I, F	0,89	-0,17	0,41	0,77	0	-1,08	-0,11	0,49	1,00	0	-1,01	-0,02	0
C19orf54	chromosome 19 open reading frame 54	0,89	-0,17	0,32	0,71	0	1,09	0,13	0,79	1,00	0	NA	NA	0
NA	NA	0,89	-0,17	0,44	0,78	0	NA	NA	NA	NA	0	NA	NA	0
SMC2	structural maintenance of chromosomes 2	0,89	-0,17	0,47	0,80	0	-1,15	-0,20	0,32	1,00	0	NA	NA	0
TRIM27	tripartite motif-containing 27	0,89	-0,17	0,43	0,78	0	-1,03	-0,04	0,78	1,00	0	NA	NA	0
CASP1	caspase 1, apoptosis-related cysteine peptidase (interleukin 1, beta, convertase)	0,89	-0,17	0,40	0,76	0	-1,12	-0,16	0,32	1,00	0	-1,09	-0,12	0
OAZ2	ornithine decarboxylase antizyme 2	0,89	-0,17	0,23	0,64	0	-1,10	-0,14	0,19	1,00	0	-1,02	-0,03	0
DNPEP	aspartyl aminopeptidase	0,89	-0,17	0,40	0,76	0	-1,06	-0,09	0,52	1,00	0	-1,06	-0,10	0
NRSN2	neurensin 2	0,89	-0,17	0,60	0,87	0	-1,19	-0,25	0,04	1,00	0	1,46	0,54	0
CDK10	cyclin-dependent kinase 10	0,89	-0,17	0,36	0,73	0	-1,06	-0,08	0,69	1,00	0	NA	NA	0
MSC	musculin	0,89	-0,17	0,59	0,86	0	-1,01	-0,01	NA	NA	0	1,09	0,12	0
EFNB2	ephrin-B2	0,89	-0,17	0,82	0,95	0	-1,43	-0,52	0,41	1,00	0	-1,14	-0,19	0
COQ3	coenzyme Q3 homolog, methyltransferase (S. cerevisiae)	0,89	-0,17	0,29	0,68	0	1,06	0,09	0,65	1,00	0	NA	NA	0
DUSP10	dual specificity phosphatase 10	0,89	-0,17	0,66	0,89	0	1,09	0,12	0,81	1,00	0	-1,37	-0,46	0
WFS1	Wolfram syndrome 1 (wolframin)	0,89	-0,17	0,51	0,82	0	-1,32	-0,40	0,15	1,00	0	NA	NA	0
PRMT3	protein arginine methyltransferase 3	0,89	-0,17	0,61	0,87	0	1,02	0,03	0,75	1,00	0	NA	NA	0
DIAPH2	diaphanous homolog 2 (Drosophila)	0,89	-0,17	0,33	0,72	0	1,02	0,03	0,87	1,00	0	NA	NA	0
SEMA4F	sema domain, immunoglobulin domain (Ig), transmembrane domain (TM) and short cytoplasmic domain, (semaphorin) 4F	0,89	-0,17	0,54	0,84	0	-1,06	-0,09	0,62	1,00	0	NA	NA	0
GPR161	G protein-coupled receptor 161	0,89	-0,17	0,57	0,85	0	-1,07	-0,10	0,75	1,00	0	NA	NA	0
LRP12	low density lipoprotein receptor-related protein 12	0,89	-0,17	0,33	0,71	0	1,26	0,33	0,33	1,00	0	-1,11	-0,16	0
SNX6	sorting nexin 6	0,89	-0,17	0,22	0,63	0	-1,00	0,00	0,97	1,00	0	NA	NA	0
TSPAN3	tetraspanin 3	0,89	-0,17	0,38	0,75	0	-1,18	-0,24	0,26	1,00	0	NA	NA	0
PDHX	pyruvate dehydrogenase complex, component X	0,89	-0,17	0,21	0,61	0	-1,02	-0,03	0,71	1,00	0	NA	NA	0
NA	NA	0,89	-0,17	0,40	0,76	0	-1,01	-0,01	NA	NA	0	-1,12	-0,17	0
ERLIN2	ER lipid raft associated 2	0,89	-0,17	0,42	0,77	0	-1,06	-0,09	0,39	1,00	0	NA	NA	0
STX7	syntaxin 7	0,89	-0,17	0,40	0,76	0	-1,03	-0,04	0,86	1,00	0	-1,28	-0,36	0
TRIM2	tripartite motif-containing 2	0,89	-0,17	0,46	0,80	0	-1,06	-0,08	0,58	1,00	0	-1,39	-0,47	0
PRKCDBP	protein kinase C, delta binding protein	0,89	-0,17	0,31	0,70	0	-1,21	-0,28	0,08	1,00	0	NA	NA	0
BATF2	basic leucine zipper transcription factor, ATF-like 2	0,89	-0,17	0,68	0,90	0	NA	NA	NA	NA	0	NA	NA	0
ZNF43	zinc finger protein 43	0,89	-0,17	0,52	0,83	0	-1,13	-0,17	0,47	1,00	0	NA	NA	0
TPP2	tripeptidyl peptidase II	0,89	-0,17	0,25	0,66	0	-1,24	-0,31	0,11	1,00	0	NA	NA	0
KLHL22	kelch-like 22 (Drosophila)	0,89	-0,17	0,36	0,74	0	-1,01	-0,01	0,92	1,00	0	0,00	0,00	0
HLA-DRB4	major histocompatibility complex, class II, DR beta 4	0,89	-0,17	0,67	0,90	0	1,02	0,03	0,81	1,00	0	0,00	0,00	0
MSH2	mutS homolog 2, colon cancer, nonpolyposis type 1 (E. coli)	0,89	-0,18	0,31	0,70	0	-1,08	-0,11	0,48	1,00	0	NA	NA	0
N6AMT1	N-6 adenine-specific DNA methyltransferase 1 (putative)	0,89	-0,18	0,43	0,78	0	-1,34	-0,42	0,25	1,00	0	NA	NA	0
CBX6	chromobox homolog 6	0,89	-0,18	0,46	0,80	0	1,03	0,05	0,76	1,00	0	1,18	0,24	0
ANGEL2	angel homolog 2 (Drosophila)	0,89	-0,18	0,39	0,75	0	-1,12	-0,17	0,27	1,00	0	NA	NA	0
NA	NA	0,89	-0,18	0,21	0,62	0	-1,05	-0,07	0,63	1,00	0	1,08	0,11	0
PRR3	proline rich 3	0,89	-0,18	0,39	0,76	0	1,00	0,00	NA	NA	0	-1,45	-0,53	0
MUT	methylmalonyl CoA mutase	0,88	-0,18	0,29	0,68	0	-1,04	-0,06	0,61	1,00	0	NA	NA	0
PARP2	poly (ADP-ribose) polymerase 2	0,88	-0,18	0,22	0,63	0	-1,07	-0,09	0,65	1,00	0	NA	NA	0
PNMAL1	PNMA-like 1	0,88	-0,18	0,58	0,86	0	-1,17	-0,22	0,42	1,00	0	NA	NA	0
LZTFL1	leucine zipper transcription factor-like 1	0,88	-0,18	0,40	0,76	0	-1,22	-0,29	0,09	1,00	0	-1,11	-0,16	0
NA	NA	0,88	-0,18	0,40	0,76	0	NA	NA	NA	NA	0	NA	NA	0
RRN3P1	RNA polymerase I transcription factor homolog (S. cerevisiae) pseudogene 1	0,88	-0,18	0,51	0,82	0	-1,11	-0,15	0,77	1,00	0	NA	NA	0
FAM114A1	family with sequence similarity 114, member A1	0,88	-0,18	0,46	0,80	0	-1,06	-0,08	0,56	1,00	0	-1,06	-0,09	0
RPA3	replication protein A3, 14kDa	0,88	-0,18	0,52	0,83	0	-1,00	-0,01	0,98	1,00	0	NA	NA	0
MLH1	mutL homolog 1, colon cancer, nonpolyposis type 2 (E. coli)	0,88	-0,18	0,29	0,69	0	-1,06	-0,08	0,57	1,00	0	NA	NA	0
NA	NA	0,88	-0,18	0,54	0,84	0	-1,01	-0,01	NA	NA	0	-1,54	-0,62	0
ARMCX2	armadillo repeat containing, X-linked 2	0,88	-0,18	0,44	0,79	0	-1,08	-0,11	0,49	1,00	0	NA	NA	0
GRSF1	G-rich RNA sequence binding factor 1	0,88	-0,18	0,32	0,71	0	1,06	0,09	0,60	1,00	0	-1,79	-0,84	0
SYNPO	synaptopodin	0,88	-0,18	0,60	0,87	0	1,05	0,07	0,82	1,00	0	-1,03	-0,05	0
SLC22A5	solute carrier family 22 (organic cation/carnitine transporter), member 5	0,88	-0,18	0,42	0,78	0	-1,05	-0,06	0,66	1,00	0	NA	NA	0
RDX	radixin	0,88	-0,18	0,54	0,84	0	-1,15	-0,20	0,53	1,00	0	-1,27	-0,34	0
LRP12	low density lipoprotein receptor-related protein 12	0,88	-0,18	0,35	0,73	0	1,23	0,30	0,66	1,00	0	-1,11	-0,16	0
SPARC	secreted protein, acidic, cysteine-rich (osteonectin)	0,88	-0,18	0,31	0,70	0	-1,09	-0,13	0,47	1,00	0	NA	NA	0
AFAP1	actin filament associated protein 1	0,88	-0,18	0,32	0,71	0	-1,08	-0,11	0,34	1,00	0	NA	NA	0
OPA1	optic atrophy 1 (autosomal dominant)	0,88	-0,18	0,40	0,76	0	-1,09	-0,12	0,75	1,00	0	NA	NA	0
NA	NA	0,88	-0,18	0,37	0,75	0	-1,01	-0,01	0,94	1,00	0	1,01	0,01	0
CTDSPL	CTD (carboxy-terminal domain, RNA polymerase II, polypeptide A) small phosphatase-like	0,88	-0,18	0,23	0,63	0	-1,17	-0,22	0,03	1,00	0	NA	NA	0
VRK2	vaccinia related kinase 2	0,88	-0,18	0,34	0,72	0	1,10	0,14	0,22	1,00	0	-1,20	-0,27	0
USPL1	ubiquitin specific peptidase like 1	0,88	-0,18	0,51	0,82	0	1,26	0,33	0,09	1,00	0	NA	NA	0
CASP1	caspase 1, apoptosis-related cysteine peptidase (interleukin 1, beta, convertase)	0,88	-0,18	0,38	0,75	0	-1,01	-0,01	0,97	1,00	0	-1,23	-0,30	0
ACYP2	acylphosphatase 2, muscle type	0,88	-0,18	0,29	0,69	0	-1,11	-0,16	0,36	1,00	0	-1,11	-0,15	0
TRPM7	transient receptor potential cation channel, subfamily M, member 7	0,88	-0,18	0,48	0,81	0	NA	NA	NA	NA	0	NA	NA	0
PSRC1	proline/serine-rich coiled-coil 1	0,88	-0,18	0,43	0,78	0	1,01	0,01	0,93	1,00	0	NA	NA	0
CD47	CD47 molecule	0,88	-0,18	0,22	0,63	0	1,11	0,16	0,26	1,00	0	-1,27	-0,33	0
VEZF1	vascular endothelial zinc finger 1	0,88	-0,18	0,27	0,67	0	-1,16	-0,22	0,31	1,00	0	NA	NA	0
PSIP1	PC4 and SFRS1 interacting protein 1	0,88	-0,18	0,48	0,81	0	-1,12	-0,16	0,35	1,00	0	NA	NA	0
NFYB	nuclear transcription factor Y, beta	0,88	-0,18	0,31	0,70	0	-1,06	-0,09	0,69	1,00	0	-1,19	-0,26	0
FNBP1	formin binding protein 1	0,88	-0,18	0,22	0,63	0	-1,02	-0,02	0,89	1,00	0	-1,09	-0,12	0
CREBZF	CREB/ATF bZIP transcription factor	0,88	-0,18	0,40	0,76	0	-1,15	-0,20	0,08	1,00	0	-1,23	-0,31	0
CLCA2	chloride channel accessory 2	0,88	-0,18	0,59	0,87	0	-1,27	-0,34	0,06	1,00	0	NA	NA	0
GCFC1	GC-rich sequence DNA-binding factor 1	0,88	-0,18	0,21	0,61	0	-1,08	-0,11	0,32	1,00	0	-1,20	-0,27	0
TTC17	tetratricopeptide repeat domain 17	0,88	-0,18	0,30	0,69	0	-1,21	-0,28	0,24	1,00	0	-1,15	-0,20	0
SRPK2	SRSF protein kinase 2	0,88	-0,18	0,20	0,60	0	-1,07	-0,10	0,38	1,00	0	-1,22	-0,28	0
COL3A1	collagen, type III, alpha 1	0,88	-0,18	0,31	0,70	0	-1,01	-0,01	NA	NA	0	-1,05	-0,08	0
TNFSF10	tumor necrosis factor (ligand) superfamily, member 10	0,88	-0,18	0,69	0,91	0	1,70	0,76	0,08	1,00	0	1,01	0,01	0
TPP2	tripeptidyl peptidase II	0,88	-0,18	0,24	0,65	0	1,08	0,11	0,83	1,00	0	-1,35	-0,42	0
CABIN1	calcineurin binding protein 1	0,88	-0,18	0,46	0,80	0	1,07	0,10	0,36	1,00	0	1,22	0,28	0
PAPOLG	poly(A) polymerase gamma	0,88	-0,18	0,51	0,82	0	-1,23	-0,29	0,56	1,00	0	NA	NA	0
LPCAT3	lysophosphatidylcholine acyltransferase 3	0,88	-0,18	0,38	0,75	0	1,19	0,25	0,38	1,00	0	NA	NA	0
KIAA1797	KIAA1797	0,88	-0,18	0,48	0,81	0	-1,00	0,00	0,98	1,00	0	-1,35	-0,43	0
EHBP1	EH domain binding protein 1	0,88	-0,18	0,23	0,64	0	-1,19	-0,24	0,07	1,00	0	-1,16	-0,22	0
ZFAND1	zinc finger, AN1-type domain 1	0,88	-0,18	0,33	0,71	0	-1,03	-0,04	0,79	1,00	0	-1,25	-0,32	0
WDR67	WD repeat domain 67	0,88	-0,18	0,39	0,76	0	1,32	0,40	0,07	1,00	0	NA	NA	0
USP46	ubiquitin specific peptidase 46	0,88	-0,18	0,35	0,73	0	1,08	0,11	0,73	1,00	0	-1,30	-0,38	0
SERPINE2	serpin peptidase inhibitor, clade E (nexin, plasminogen activator inhibitor type 1), member 2	0,88	-0,18	0,30	0,69	0	-1,22	-0,29	0,09	1,00	0	NA	NA	0
HIST1H2BD	histone cluster 1, H2bd	0,88	-0,18	0,26	0,66	0	1,29	0,37	0,05	1,00	0	NA	NA	0
PTK7	PTK7 protein tyrosine kinase 7	0,88	-0,19	0,39	0,76	0	-1,26	-0,34	0,09	1,00	0	-1,27	-0,34	0
ACSL3	acyl-CoA synthetase long-chain family member 3	0,88	-0,19	0,45	0,79	0	-1,15	-0,20	0,12	1,00	0	NA	NA	0
PRPS1	phosphoribosyl pyrophosphate synthetase 1	0,88	-0,19	0,44	0,78	0	1,05	0,08	0,59	1,00	0	NA	NA	0
08-set	septin 8	0,88	-0,19	0,52	0,83	0	1,05	0,06	0,83	1,00	0	-1,25	-0,32	0
GSTA4	glutathione S-transferase alpha 4	0,88	-0,19	0,53	0,83	0	-1,13	-0,18	0,14	1,00	0	-1,35	-0,44	0

NNT	nicotinamide nucleotide transhydrogenase	0,88	-0,19	0,51	0,82	0	1,04	0,06	0,92	1,00	0	-1,39	-0,47	0
NA	NA	0,88	-0,19	0,38	0,75	0	1,00	0,00	0,99	1,00	0	NA	NA	0
SLC39A8	solute carrier family 39 (zinc transporter), member 8	0,88	-0,19	0,62	0,88	0	1,19	0,25	0,63	1,00	0	-1,23	-0,30	0
APPBP2	amyloid beta precursor protein (cytoplasmic tail) binding protein 2	0,88	-0,19	0,33	0,72	0	-1,05	-0,07	0,76	1,00	0	NA	NA	0
CCNB1IP1	cyclin B1 interacting protein 1	0,88	-0,19	0,35	0,73	0	-1,14	-0,19	0,18	1,00	0	NA	NA	0
FZD2	frizzled homolog 2 (Drosophila)	0,88	-0,19	0,49	0,81	0	-1,32	-0,40	0,14	1,00	0	NA	NA	0
TOPBP1	topoisomerase (DNA) II binding protein 1	0,88	-0,19	0,22	0,63	0	1,01	0,02	0,86	1,00	0	NA	NA	0
MIPEP	mitochondrial intermediate peptidase	0,88	-0,19	0,37	0,74	0	1,08	0,11	0,47	1,00	0	NA	NA	0
AGPS	alkylglycerone phosphate synthase	0,88	-0,19	0,28	0,67	0	-1,14	-0,19	0,53	1,00	0	-1,32	-0,40	0
PODXL	podocalyxin-like	0,88	-0,19	0,45	0,79	0	1,07	0,10	0,52	1,00	0	-1,43	-0,51	0
ECM1	extracellular matrix protein 1	0,88	-0,19	0,45	0,79	0	1,00	0,00	0,99	1,00	0	NA	NA	0
COG6	component of oligomeric golgi complex 6	0,88	-0,19	0,21	0,62	0	NA	NA	NA	NA	0	NA	NA	0
02-mar	membrane-associated ring finger (C3HC4) 2	0,88	-0,19	0,40	0,76	0	1,04	0,05	0,82	1,00	0	NA	NA	0
FIG4	FIG4 homolog, SAC1 lipid phosphatase domain containing (S. cerevisiae)	0,88	-0,19	0,34	0,72	0	-1,11	-0,15	0,39	1,00	0	-1,43	-0,51	0
CTNNBIP1	catenin, beta interacting protein 1	0,88	-0,19	0,40	0,76	0	1,04	0,06	0,53	1,00	0	NA	NA	0
ASB13	ankyrin repeat and SOCS box-containing 13	0,88	-0,19	0,35	0,73	0	-1,08	-0,11	0,64	1,00	0	NA	NA	0
KIAA0427	KIAA0427	0,88	-0,19	0,43	0,78	0	-1,06	-0,08	NA	NA	0	-1,01	-0,02	0
TCF4	transcription factor 4	0,88	-0,19	0,33	0,72	0	-1,41	-0,50	0,26	1,00	0	NA	NA	0
TFPI2	tissue factor pathway inhibitor 2	0,88	-0,19	0,50	0,82	0	-1,49	-0,57	0,02	0,88	0	1,02	0,03	0
CDK19	cyclin-dependent kinase 19	0,88	-0,19	0,20	0,61	0	1,02	0,02	0,90	1,00	0	-1,12	-0,17	0
CDR2L	cerebellar degeneration-related protein 2-like	0,88	-0,19	0,50	0,82	0	1,02	0,02	0,90	1,00	0	NA	NA	0
AQP3	aquaporin 3 (Gill blood group)	0,88	-0,19	0,35	0,73	0	-1,07	-0,10	0,54	1,00	0	NA	NA	0
MBD5	methyl-CpG binding domain protein 5	0,88	-0,19	0,28	0,68	0	1,00	0,01	0,97	1,00	0	-1,19	-0,25	0
B2M	beta-2-microglobulin	0,88	-0,19	0,19	0,60	0	-1,05	-0,08	0,33	1,00	0	NA	NA	0
ITSN1	intersectin 1 (SH3 domain protein)	0,88	-0,19	0,35	0,73	0	-1,11	-0,15	0,24	1,00	0	NA	NA	0
NA	NA	0,88	-0,19	0,17	0,57	0	-1,10	-0,14	0,17	1,00	0	NA	NA	0
PTGS1	prostaglandin-endoperoxide synthase 1 (prostaglandin G/H synthase and cyclooxygenase)	0,88	-0,19	0,34	0,72	0	-1,35	-0,43	0,25	1,00	0	NA	NA	0
KLF9	Kruppel-like factor 9	0,88	-0,19	0,39	0,76	0	-1,19	-0,26	0,14	1,00	0	-1,12	-0,17	0
MYST4	MYST histone acetyltransferase (monocytic leukemia) 4	0,88	-0,19	0,32	0,71	0	-1,16	-0,21	0,04	1,00	0	-1,28	-0,37	0
DPYSL2	dihydropyrimidinase-like 2	0,88	-0,19	0,25	0,65	0	-1,20	-0,27	0,20	1,00	0	NA	NA	0
HLA-DPB1	major histocompatibility complex, class II, DP beta 1	0,88	-0,19	0,43	0,78	0	-1,02	-0,03	0,93	1,00	0	-1,28	-0,36	0
TMEM231	transmembrane protein 231	0,87	-0,19	0,56	0,85	0	1,01	0,02	0,91	1,00	0	NA	NA	0
COL5A2	collagen, type V, alpha 2	0,87	-0,19	0,48	0,81	0	-1,32	-0,40	0,45	1,00	0	NA	NA	0
FHL1	four and a half LIM domains 1	0,87	-0,19	0,36	0,74	0	-1,15	-0,20	0,48	1,00	0	1,07	0,09	0
C1GALT1C1	C1GALT1-specific chaperone 1	0,87	-0,19	0,30	0,69	0	1,08	0,11	0,51	1,00	0	-1,20	-0,27	0
RHOQ	ras homolog gene family, member Q	0,87	-0,19	0,36	0,74	0	-1,05	-0,07	0,68	1,00	0	-1,14	-0,19	0
FTSJD2	FtsJ methyltransferase domain containing 2	0,87	-0,19	0,44	0,78	0	-1,01	-0,01	0,92	1,00	0	-1,06	-0,09	0
NA	NA	0,87	-0,19	0,46	0,80	0	-1,05	-0,07	0,66	1,00	0	NA	NA	0
C5orf15	chromosome 5 open reading frame 15	0,87	-0,19	0,21	0,62	0	-1,04	-0,05	0,74	1,00	0	-1,09	-0,13	0
ISL1	ISL LIM homeobox 1	0,87	-0,19	0,41	0,77	0	-1,29	-0,37	0,16	1,00	0	NA	NA	0
HNRNPD	heterogeneous nuclear ribonucleoprotein D (AU-rich element RNA binding protein 1, 37kDa)	0,87	-0,19	0,61	0,88	0	-1,09	-0,12	0,55	1,00	0	1,02	0,03	0
RARRES1	retinoic acid receptor responder (tazarotene induced) 1	0,87	-0,19	0,67	0,90	0	-1,01	-0,01	0,99	1,00	0	-1,72	-0,80	0
PLEKHA5	pleckstrin homology domain containing, family A member 5	0,87	-0,19	0,32	0,71	0	-1,05	-0,07	0,65	1,00	0	-1,39	-0,48	0
TRPM4	transient receptor potential cation channel, subfamily M, member 4	0,87	-0,19	0,58	0,86	0	-1,06	-0,09	0,60	1,00	0	NA	NA	0
CCDC6	coiled-coil domain containing 6	0,87	-0,19	0,24	0,64	0	-1,27	-0,34	0,59	1,00	0	-1,03	-0,05	0
ZNF195	zinc finger protein 195	0,87	-0,19	0,35	0,73	0	1,22	0,29	0,45	1,00	0	-1,30	-0,37	0
SOCS5	suppressor of cytokine signaling 5	0,87	-0,19	0,32	0,71	0	-1,03	-0,04	0,76	1,00	0	NA	NA	0
RIPK4	receptor-interacting serine-threonine kinase 4	0,87	-0,20	0,62	0,88	0	-1,13	-0,17	0,35	1,00	0	NA	NA	0
GMCL1	germ cell-less homolog 1 (Drosophila)	0,87	-0,20	0,46	0,80	0	1,03	0,04	0,89	1,00	0	NA	NA	0
AASDHPPT	aminoadipate-semialdehyde dehydrogenase-phosphopantetheinyl transferase	0,87	-0,20	0,43	0,78	0	-1,03	-0,04	0,79	1,00	0	NA	NA	0
RAPGEFL1	Rap guanine nucleotide exchange factor (GEF)-like 1	0,87	-0,20	0,22	0,62	0	-1,03	-0,05	0,83	1,00	0	NA	NA	0
ABAT	4-aminobutyrate aminotransferase	0,87	-0,20	0,36	0,74	0	1,19	0,25	0,32	1,00	0	NA	NA	0
BNIP3	BCL2/adenovirus E1B 19kDa interacting protein 3	0,87	-0,20	0,29	0,69	0	1,00	0,01	0,95	1,00	0	NA	NA	0
DDX10	DEAD (Asp-Glu-Ala-Asp) box polypeptide 10	0,87	-0,20	0,39	0,76	0	1,14	0,19	0,37	1,00	0	NA	NA	0
CELF2	CUGBP, Elav-like family member 2	0,87	-0,20	0,45	0,79	0	-1,13	-0,18	0,42	1,00	0	NA	NA	0
C16orf88	chromosome 16 open reading frame 88	0,87	-0,20	0,27	0,67	0	-1,13	-0,18	0,08	1,00	0	NA	NA	0
HRH1	histamine receptor H1	0,87	-0,20	0,42	0,77	0	-1,02	-0,02	0,90	1,00	0	NA	NA	0
DENND2A	DENN/MADD domain containing 2A	0,87	-0,20	0,48	0,81	0	-1,01	-0,02	0,90	1,00	0	-1,14	-0,18	0
PECI	peroxisomal D3,D2-enoyl-CoA isomerase	0,87	-0,20	0,23	0,63	0	-1,17	-0,22	0,18	1,00	0	-1,20	-0,27	0
BLMH	bleomycin hydrolase	0,87	-0,20	0,64	0,89	0	-1,31	-0,39	0,11	1,00	0	NA	NA	0
TST	thiosulfate sulfurtransferase (rhodanese)	0,87	-0,20	0,26	0,66	0	-1,01	-0,02	0,90	1,00	0	1,08	0,11	0
NUP43	nucleoporin 43kDa	0,87	-0,20	0,50	0,82	0	-1,08	-0,11	0,57	1,00	0	NA	NA	0
CHST15	carbohydrate (N-acetylgalactosamine 4-sulfate 6-O) sulfotransferase 15	0,87	-0,20	0,25	0,65	0	1,02	0,02	0,85	1,00	0	NA	NA	0
C5orf56	chromosome 5 open reading frame 56	0,87	-0,20	0,50	0,82	0	NA	NA	NA	NA	0	NA	NA	0
AP3S2	adaptor-related protein complex 3, sigma 2 subunit	0,87	-0,20	0,33	0,71	0	-1,08	-0,11	0,66	1,00	0	NA	NA	0
MTMR2	myotubularin related protein 2	0,87	-0,20	0,21	0,62	0	-1,01	-0,02	0,84	1,00	0	-1,06	-0,08	0
NTF3	neurotrophin 3	0,87	-0,20	0,58	0,86	0	-1,01	-0,01	NA	NA	0	1,08	0,12	0
PDS5B	PDS5, regulator of cohesion maintenance, homolog B (S. cerevisiae)	0,87	-0,20	0,34	0,72	0	-1,22	-0,28	0,26	1,00	0	NA	NA	0
FBXO9	F-box protein 9	0,87	-0,20	0,30	0,69	0	1,09	0,13	0,18	1,00	0	-1,22	-0,29	0
GPX4	glutathione peroxidase 4 (phospholipid hydroperoxidase)	0,87	-0,20	0,18	0,59	0	-1,14	-0,18	0,18	1,00	0	1,07	0,10	0
TCF3	transcription factor 3 (E2A immunoglobulin enhancer binding factors E12/E47)	0,87	-0,20	0,45	0,79	0	-1,17	-0,23	0,29	1,00	0	NA	NA	0
ABCA1	ATP-binding cassette, sub-family A (ABC1), member 1	0,87	-0,20	0,18	0,59	0	1,23	0,30	0,23	1,00	0	NA	NA	0
JUN	jun proto-oncogene	0,87	-0,20	0,12	0,51	0	1,17	0,23	0,23	1,00	0	NA	NA	0
HIST1H2BI	histone cluster 1, H2bi	0,87	-0,20	0,26	0,66	0	1,27	0,35	0,06	1,00	0	NA	NA	0
MOXD1	monooxygenase, DBH-like 1	0,87	-0,20	0,45	0,79	0	-1,00	-0,01	NA	NA	0	-1,25	-0,32	0
MAGED2	melanoma antigen family D, 2	0,87	-0,20	0,34	0,72	0	1,01	0,01	0,91	1,00	0	NA	NA	0
THBD	thrombomodulin	0,87	-0,20	0,67	0,90	0	-1,15	-0,20	0,28	1,00	0	NA	NA	0
NHEJ1	nonhomologous end-joining factor 1	0,87	-0,20	0,19	0,59	0	-1,19	-0,25	0,06	1,00	0	-1,25	-0,33	0
B3GNT1	UDP-GlcNAc:betaGal beta-1,3-N-acetylglucosaminyltransferase 1	0,87	-0,20	0,42	0,78	0	-1,04	-0,06	0,73	1,00	0	NA	NA	0
NOL8	nucleolar protein 8	0,87	-0,20	0,18	0,59	0	-1,03	-0,04	0,69	1,00	0	NA	NA	0
AKR7A2	aldo-keto reductase family 7, member A2 (aflatoxin aldehyde reductase)	0,87	-0,20	0,30	0,69	0	-1,14	-0,19	0,32	1,00	0	NA	NA	0
TRIM2	tripartite motif-containing 2	0,87	-0,20	0,61	0,88	0	-1,03	-0,04	0,84	1,00	0	-1,19	-0,24	0
ACAP2	ArfGAP with coiled-coil, ankyrin repeat and PH domains 2	0,87	-0,20	0,41	0,77	0	-1,16	-0,22	0,06	1,00	0	NA	NA	0
PTPRF	protein tyrosine phosphatase, receptor type, F	0,87	-0,20	0,64	0,89	0	-1,15	-0,20	0,66	1,00	0	1,07	0,10	0
ABR	active BCR-related gene	0,87	-0,20	0,30	0,69	0	1,08	0,11	0,28	1,00	0	NA	NA	0
PARD3	par-3 partitioning defective 3 homolog (C. elegans)	0,87	-0,20	0,38	0,75	0	-1,20	-0,26	0,22	1,00	0	NA	NA	0
IFT74	intraflagellar transport 74 homolog (Chlamydomonas)	0,87	-0,20	0,37	0,74	0	-1,11	-0,15	0,58	1,00	0	NA	NA	0
LOC399491	GPS, PLAT and transmembrane domain-containing protein	0,87	-0,20	0,24	0,64	0	-1,21	-0,28	0,10	1,00	0	NA	NA	0
CCNA2	cyclin A2	0,87	-0,20	0,49	0,81	0	1,09	0,12	0,53	1,00	0	NA	NA	0
C14orf2	chromosome 14 open reading frame 2	0,87	-0,20	0,48	0,81	0	1,02	0,02	0,91	1,00	0	NA	NA	0
RNF31	ring finger protein 31	0,87	-0,20	0,48	0,81	0	-1,12	-0,16	0,41	1,00	0	-1,14	-0,18	0
NA	NA	0,87	-0,20	0,38	0,75	0	NA	NA	NA	NA	0	NA	NA	0
FLG	filaggrin	0,87	-0,20	0,49	0,81	0	-2,83	-1,50	0,11	1,00	0	NA	NA	0
FHL1	four and a half LIM domains 1	0,87	-0,20	0,21	0,61	0	-1,13	-0,17	0,30	1,00	0	-1,05	-0,08	0
TNS1	tensin 1	0,87	-0,20	0,43	0,78	0	1,02	0,02	0,94	1,00	0	NA	NA	0
EBAG9	estrogen receptor binding site associated, antigen, 9	0,87	-0,20	0,18	0,58	0	-1,16	-0,22	0,23	1,00	0	NA	NA	0
DIMT1L	DIM1 dimethyladenosine transferase 1-like (S. cerevisiae)	0,87	-0,20	0,33	0,71	0	-1,05	-0,07	0,50	1,00	0	-1,06	-0,09	0
AKR7A3	aldo-keto reductase family 7, member A3 (aflatoxin aldehyde reductase)	0,87	-0,20	0,31	0,70	0	-1,13	-0,18	0,30	1,00	0	NA	NA	0
OSBPL3	oxysterol binding protein-like 3	0,87	-0,20	0,36	0,74	0	1,01	0,02	0,91	1,00	0	NA	NA	0
TMEM5	transmembrane protein 5	0,87	-0,21	0,18	0,59	0	-1,09	-0,12	0,56	1,00	0	NA	NA	0
NFYB	nuclear transcription factor Y, beta	0,87	-0,21	0,31	0,70	0	1,04	0,05	0,76	1,00	0	-1,14	-0,18	0
JAG2	jagged 2	0,87	-0,21	0,55	0,84	0	-1,03	-0,04	0,80	1,00	0	NA	NA	0
GPT2	glutamic pyruvate transaminase (alanine aminotransferase) 2	0,87	-0,21	0,28	0,68	0	NA	NA	NA	NA	0	NA	NA	0
GALC	galactosylceramidase	0,87	-0,21	0,24	0,64	0	-1,27	-0,35	0,05	1,00	0	NA	NA	0
HOXA10	homeobox A10	0,87	-0,21	0,30	0,69	0	-1,04	-0,06	0,57	1,00	0	-1,30	-0,38	0
TGFB1	transforming growth factor, beta 1	0,87	-0,21	0,69	0,90	0	1,06	0,09	0,78	1,00	0	NA	NA	0
GGA2	golgi-associated, gamma adaptin ear containing, ARF binding protein 2	0,87	-0,21	0,18	0,58	0	-1,05	-0,06	0,79	1,00	0	NA	NA	0
GCLM	glutamate-cysteine ligase, modifier subunit	0,87	-0,21	0,51	0,82	0	1,15	0,20	0,36	1,00	0	NA	NA	0
CAPN3	calpain 3, (p94)	0,87	-0,21	0,43	0,78	0	1,11	0,15	0,61	1,00	0	0,00	0,00	0
CHMP5	chromatin modifying protein 5	0,87	-0,21	0,29	0,69	0	1,07	0,10	0,47	1,00	0	NA	NA	0
FANCL	Fanconi anemia, complementation group L	0,87	-0,21	0,32	0,71	0	-1,06	-0,08	0,55	1,00	0	NA	NA	0
PASK	PAS domain containing serine/threonine kinase	0,87	-0,21	0,19	0,60	0	-1,18	-0,24	0,02	0,84	0	NA	NA	0
ATP9B	ATPase, class II, type 9B	0,87	-0,21	0,29	0,68	0	-1,09	-0,12	0,59	1,00	0	NA	NA	0
F2R	coagulation factor II (thrombin) receptor	0,87	-0,21	0,23	0,63	0	-1,55	-0,64	0,01	0,77	0	-1,39	-0,46	0
HERC1	hect (homologous to the E6-AP (UBE3A) carboxyl terminus) domain and RCC1 (CHC1)-like domain (RLD) 1	0,87	-0,21	0,16	0,57	0	1,14	0,19	0,49	1,00	0	NA	NA	0
PER2	period homolog 2 (Drosophila)	0,87	-0,21	0,42	0,78	0	1,22	0,29	0,29	1,00	0	NA	NA	0
LOC220594	ubiquitin specific peptidase 6 (Tre-2 oncogene) pseudogene	0,87	-0,21	0,33	0,71	0	-1,06	-0,09	0,32	1,00	0	NA	NA	0
ID3	inhibitor of DNA binding 3, dominant negative helix-loop-helix protein	0,86	-0,21	0,59	0,87	0	-1,06	-0,09	0,75	1,00	0	NA	NA	0
CES2	carboxylesterase 2 (intestine, liver)	0,86	-0,21	0,65	0,89	0	-1,06	-0,09	0,70	1,00	0	NA	NA	0
MCCC1	methylcrotonoyl-CoA carboxylase 1 (alpha)	0,86	-0,21	0,16	0,56	0	-1,03	-0,05	0,67	1,00	0	NA	NA	0
TPD52	tumor protein D52	0,86	-0,21	0,46	0,80	0	1,11	0,15	0,06	1,00	0	-1,37	-0,46	0
FBXO28	F-box protein 28	0,86	-0,21	0,15	0,55	0	1,00	0,00	0,97	1,00	0	0,00	0,00	0
FAM46A	family with sequence similarity 46, member A	0,86	-0,21	0,40	0,76	0	1,16	0,21	0,55	1,00	0	-1,20	-0,27	0
NA	NA	0,86	-0,21	0,38	0,75	0	NA	NA	NA	NA	0	NA	NA	0
NSL1	NSL1, MIND kinetochore complex component, homolog (S. cerevisiae)	0,86	-0,21	0,38	0,75	0	1,10	0,14	0,33	1,00	0	NA	NA	0
RAB27A	RAB27A, member RAS oncogene family	0,86	-0,21	0,38	0,75	0	1,06	0,09	0,80	1,00	0	NA	NA	0
TMEM106C	transmembrane protein 106C	0,86	-0,21	0,32	0,71	0	-1,06	-0,08	0,29	1,00	0	NA	NA	0
RNF130	ring finger protein 130	0,86	-0,21	0,28	0,68	0	1,16	0,21	0,31	1,00	0	-1,14	-0,19	0
JAG2	jagged 2	0,86	-0,21	0,40	0,76	0	-1,08	-0,11	0,57	1,00	0	NA	NA	0
FLJ32255	hypothetical protein LOC643977	0,86	-0,21	0,63	0,88	0	NA	NA	NA	NA	0	NA	NA	0
ALG6	asparagine-linked glycosylation 6, alpha-1,3-glucosyltransferase homolog (S. cerevisiae)	0,86	-0,21	0,34	0,72	0	1,08	0,11	0,32	1,00	0	NA	NA	0
RPL31	ribosomal protein L31	0,86	-0,21	0,20	0,60	0	1,01	0,01	0,99	1,00	0	-1,16	-0,22	0
BCLAF1	BCL2-associated transcription factor 1	0,86	-0,21	0,22	0,62	0	-1,01	-0,01	0,85	1,00	0	NA	NA	0
SHQ1	SHQ1 homolog (S. cerevisiae)	0,86	-0,21	0,16	0,56	0	1,05	0,07	0,46	1,00	0	NA	NA	0
TOMM70A	translocase of outer mitochondrial membrane 70 homolog A (S. cerevisiae)	0,86	-0,21	0,19	0,60	0	1,02	0,02	0,79	1,00	0	NA	NA	0
SNX5	sorting nexin 5	0,86	-0,21	0,27	0,67	0	-1,03	-0,04	0,72	1,00	0	NA	NA	0
DHRS3	dehydrogenase/reductase (SDR family) member 3	0,86	-0,21	0,80	0,94	0	-1,02	-0,02	0,92	1,00	0	-1,16	-0,23	0

ERAP1	endoplasmic reticulum aminopeptidase 1	0,86	-0,21	0,55	0,84	0	-1,22	-0,29	0,40	1,00	0	1,23	0,30	0
GJD3	gap junction protein, delta 3, 31.9kDa	0,86	-0,21	0,60	0,87	0	NA	NA	NA	NA	0	NA	NA	0
EHMT2	euchromatic histone-lysine N-methyltransferase 2	0,86	-0,21	0,41	0,77	0	-1,02	-0,03	0,89	1,00	0	NA	NA	0
PLEKHG3	pleckstrin homology domain containing, family G (with RhoGef domain) member 3	0,86	-0,21	0,59	0,87	0	-1,32	-0,40	0,24	1,00	0	NA	NA	0
ETV7	ets variant 7	0,86	-0,21	0,76	0,93	0	NA	NA	NA	NA	0	NA	NA	0
SCCPDH	saccharopine dehydrogenase (putative)	0,86	-0,21	0,41	0,77	0	1,08	0,11	0,37	1,00	0	-1,27	-0,35	0
PEX1	peroxisomal biogenesis factor 1	0,86	-0,21	0,38	0,75	0	-1,02	-0,03	0,93	1,00	0	-1,61	-0,68	0
RYK	RYK receptor-like tyrosine kinase	0,86	-0,21	0,16	0,57	0	1,00	0,01	0,97	1,00	0	NA	NA	0
MAGEH1	melanoma antigen family H, 1	0,86	-0,21	0,47	0,80	0	-1,06	-0,08	0,78	1,00	0	-1,20	-0,27	0
YAF2	YY1 associated factor 2	0,86	-0,22	0,44	0,79	0	1,12	0,16	0,56	1,00	0	-1,47	-0,56	0
CDR2	cerebellar degeneration-related protein 2, 62kDa	0,86	-0,22	0,29	0,69	0	1,04	0,06	0,62	1,00	0	NA	NA	0
LOC440434	hypothetical protein FLJ11822	0,86	-0,22	0,24	0,64	0	1,10	0,13	0,49	1,00	0	NA	NA	0
CLU	clusterin	0,86	-0,22	0,42	0,78	0	-1,01	-0,01	0,96	1,00	0	NA	NA	0
SCML1	sex comb on midleg-like 1 (Drosophila)	0,86	-0,22	0,51	0,83	0	-1,02	-0,02	0,90	1,00	0	0,00	-0,01	0
SLC35D1	solute carrier family 35 (UDP-glucuronic acid/UDP-N-acetylgalactosamine dual transporter), member D1	0,86	-0,22	0,27	0,67	0	1,14	0,19	0,17	1,00	0	NA	NA	0
C15orf59	chromosome 15 open reading frame 59	0,86	-0,22	0,24	0,64	0	NA	NA	NA	NA	0	NA	NA	0
ZBTB24	zinc finger and BTB domain containing 24	0,86	-0,22	0,28	0,68	0	1,01	0,02	0,93	1,00	0	NA	NA	0
FBXO31	F-box protein 31	0,86	-0,22	0,34	0,72	0	1,24	0,32	0,54	1,00	0	-1,22	-0,28	0
IFT74	intraflagellar transport 74 homolog (Chlamydomonas)	0,86	-0,22	0,27	0,67	0	-1,08	-0,11	0,66	1,00	0	NA	NA	0
CCDC109B	coiled-coil domain containing 109B	0,86	-0,22	0,57	0,85	0	1,06	0,08	0,55	1,00	0	-1,22	-0,28	0
MKKS	McKusick-Kaufman syndrome	0,86	-0,22	0,30	0,70	0	1,14	0,19	0,40	1,00	0	NA	NA	0
RFX5	regulatory factor X, 5 (influences HLA class II expression)	0,86	-0,22	0,31	0,70	0	-1,16	-0,22	0,18	1,00	0	-1,16	-0,21	0
MAN2B2	mannosidase, alpha, class 2B, member 2	0,86	-0,22	0,37	0,74	0	-1,17	-0,23	0,21	1,00	0	NA	NA	0
NARG2	NMDA receptor regulated 2	0,86	-0,22	0,12	0,51	0	-1,10	-0,14	0,26	1,00	0	NA	NA	0
NA	NA	0,86	-0,22	0,27	0,67	0	1,05	0,07	0,79	1,00	0	-1,32	-0,40	0
TRAFD1	TRAF-type zinc finger domain containing 1	0,86	-0,22	0,36	0,73	0	NA	NA	NA	NA	0	NA	NA	0
MTR	5-methyltetrahydrofolate-homocysteine methyltransferase	0,86	-0,22	0,17	0,57	0	-1,04	-0,06	0,70	1,00	0	NA	NA	0
NA	NA	0,86	-0,22	0,24	0,65	0	1,01	0,02	0,88	1,00	0	NA	NA	0
CEP68	centrosomal protein 68kDa	0,86	-0,22	0,15	0,55	0	-1,48	-0,56	0,03	1,00	0	-1,59	-0,67	0
P4HA2	prolyl 4-hydroxylase, alpha polypeptide II	0,86	-0,22	0,17	0,57	0	1,15	0,20	0,13	1,00	0	NA	NA	0
CREBZF	CREB/ATF bZIP transcription factor	0,86	-0,22	0,40	0,76	0	1,03	0,04	0,84	1,00	0	-1,43	-0,52	0
FBXO9	F-box protein 9	0,86	-0,22	0,32	0,71	0	-1,01	-0,02	0,90	1,00	0	-1,09	-0,12	0
DOK4	docking protein 4	0,86	-0,22	0,23	0,63	0	1,12	0,16	0,42	1,00	0	NA	NA	0
P2RY6	pyrimidinergic receptor P2Y, G-protein coupled, 6	0,86	-0,22	0,54	0,84	0	1,05	0,07	0,42	1,00	0	NA	NA	0
PPAP2A	phosphatidic acid phosphatase type 2A	0,86	-0,22	0,14	0,54	0	1,05	0,06	0,63	1,00	0	-1,01	-0,01	0
EXOSC7	exosome component 7	0,86	-0,22	0,26	0,66	0	-1,15	-0,20	0,09	1,00	0	NA	NA	0
C19orf40	chromosome 19 open reading frame 40	0,86	-0,22	0,41	0,77	0	-1,03	-0,04	0,84	1,00	0	1,09	0,13	0
ME3	malic enzyme 3, NADP(+)-dependent, mitochondrial	0,86	-0,22	0,33	0,72	0	1,01	0,02	0,91	1,00	0	NA	NA	0
MID1	midline 1 (Opitz/BBB syndrome)	0,86	-0,22	0,36	0,74	0	1,29	0,37	0,08	1,00	0	NA	NA	0
ERLIN2	ER lipid raft associated 2	0,86	-0,22	0,15	0,55	0	1,00	0,00	0,99	1,00	0	-1,19	-0,26	0
SCNN1A	sodium channel, nonvoltage-gated 1 alpha	0,86	-0,22	0,25	0,65	0	-1,47	-0,56	0,22	1,00	0	NA	NA	0
CUTC	cutC copper transporter homolog (E. coli)	0,86	-0,22	0,29	0,69	0	1,04	0,05	0,81	1,00	0	-1,45	-0,54	0
TREX1	three prime repair exonuclease 1	0,86	-0,22	0,41	0,77	0	-1,06	-0,09	0,47	1,00	0	1,08	0,11	0
DST	dystonin	0,86	-0,22	0,09	0,46	0	-1,25	-0,32	0,02	0,84	0	NA	NA	0
MTERFD1	MTERF domain containing 1	0,86	-0,22	0,29	0,69	0	-1,05	-0,07	0,59	1,00	0	NA	NA	0
SMC5	structural maintenance of chromosomes 5	0,86	-0,22	0,29	0,68	0	-1,22	-0,29	0,11	1,00	0	NA	NA	0
AHCYL2	adenosylhomocysteinase-like 2	0,86	-0,22	0,40	0,76	0	-1,10	-0,14	0,33	1,00	0	NA	NA	0
NA	NA	0,86	-0,22	0,14	0,54	0	NA	NA	NA	NA	0	NA	NA	0
NA	NA	0,86	-0,22	0,20	0,61	0	1,03	0,04	0,82	1,00	0	NA	NA	0
NPIP	nuclear pore complex interacting protein	0,86	-0,22	0,19	0,60	0	-1,05	-0,08	0,63	1,00	0	NA	NA	0
ORC3L	origin recognition complex, subunit 3-like (yeast)	0,86	-0,22	0,26	0,66	0	1,06	0,09	0,53	1,00	0	NA	NA	0
C2CD3	C2 calcium-dependent domain containing 3	0,86	-0,22	0,18	0,58	0	-1,14	-0,19	0,34	1,00	0	-1,15	-0,20	0
PFN2	profilin 2	0,86	-0,22	0,13	0,52	0	-1,16	-0,21	0,02	0,84	0	NA	NA	0
RNF219	ring finger protein 219	0,86	-0,22	0,25	0,65	0	-1,13	-0,17	0,32	1,00	0	NA	NA	0
TCIRG1	T-cell, immune regulator 1, ATPase, H+ transporting, lysosomal V0 subunit A3	0,86	-0,22	0,43	0,78	0	-1,03	-0,04	0,85	1,00	0	NA	NA	0
UNC119B	unc-119 homolog B (C. elegans)	0,86	-0,23	0,10	0,47	0	-1,08	-0,12	0,45	1,00	0	-1,12	-0,17	0
GLS	glutaminase	0,86	-0,23	0,38	0,75	0	-1,16	-0,21	0,10	1,00	0	-1,10	-0,14	0
CAPN3	calpain 3, (p94)	0,86	-0,23	0,60	0,87	0	1,00	0,01	0,99	1,00	0	NA	NA	0
STX2	syntaxin 2	0,85	-0,23	0,30	0,69	0	1,02	0,03	0,92	1,00	0	NA	NA	0
DECR1	2,4-dienoyl CoA reductase 1, mitochondrial	0,85	-0,23	0,23	0,63	0	-1,13	-0,17	0,26	1,00	0	-1,18	-0,23	0
N4BP2L2	NEDD4 binding protein 2-like 2	0,85	-0,23	0,16	0,57	0	-1,26	-0,33	0,28	1,00	0	NA	NA	0
DNAJC9	DnaJ (Hsp40) homolog, subfamily C, member 9	0,85	-0,23	0,12	0,51	0	1,04	0,05	0,66	1,00	0	NA	NA	0
LSM5	LSM5 homolog, U6 small nuclear RNA associated (S. cerevisiae)	0,85	-0,23	0,58	0,86	0	1,05	0,07	0,84	1,00	0	NA	NA	0
SYT11	synaptotagmin XI	0,85	-0,23	0,59	0,87	0	-1,00	0,00	NA	NA	0	-1,23	-0,30	0
C18orf10	chromosome 18 open reading frame 10	0,85	-0,23	0,33	0,72	0	-1,11	-0,15	0,51	1,00	0	-1,04	-0,06	0
TCF7L2	transcription factor 7-like 2 (T-cell specific, HMG-box)	0,85	-0,23	0,24	0,65	0	-1,14	-0,19	0,22	1,00	0	NA	NA	0
KDELR3	KDEL (Lys-Asp-Glu-Leu) endoplasmic reticulum protein retention receptor 3	0,85	-0,23	0,29	0,69	0	-1,53	-0,62	0,01	0,48	0	-1,22	-0,29	0
C2	complement component 2	0,85	-0,23	0,49	0,81	0	-1,01	-0,01	NA	NA	0	NA	NA	0
SNX13	sorting nexin 13	0,85	-0,23	0,17	0,57	0	-1,22	-0,29	0,09	1,00	0	NA	NA	0
OPN3	opsin 3	0,85	-0,23	0,10	0,46	0	-1,07	-0,10	0,49	1,00	0	NA	NA	0
TMEM50B	transmembrane protein 50B	0,85	-0,23	0,40	0,76	0	-1,24	-0,31	0,14	1,00	0	NA	NA	0
PKD1P1	polycystic kidney disease 1 (autosomal dominant) pseudogene 1	0,85	-0,23	0,20	0,61	0	-1,06	-0,09	0,58	1,00	0	NA	NA	0
MYL6	myosin, light chain 6, alkali, smooth muscle and non-muscle	0,85	-0,23	0,13	0,53	0	-1,05	-0,07	0,54	1,00	0	1,22	0,29	0
IMPA2	inositol(myo)-1(or 4)-monophosphatase 2	0,85	-0,23	0,37	0,75	0	-1,03	-0,04	0,74	1,00	0	NA	NA	0
PDCD4	programmed cell death 4 (neoplastic transformation inhibitor)	0,85	-0,23	0,12	0,50	0	-1,21	-0,28	0,05	1,00	0	NA	NA	0
PARP12	poly (ADP-ribose) polymerase family, member 12	0,85	-0,23	0,45	0,79	0	1,19	0,25	0,24	1,00	0	-1,39	-0,48	0
FAM149B1	family with sequence similarity 149, member B1	0,85	-0,23	0,26	0,66	0	1,07	0,10	0,48	1,00	0	-1,25	-0,33	0
RAB4A	RAB4A, member RAS oncogene family	0,85	-0,23	0,28	0,68	0	-1,03	-0,04	0,64	1,00	0	-1,16	-0,22	0
PSIP1	PC4 and SFRS1 interacting protein 1	0,85	-0,23	0,35	0,73	0	-1,19	-0,26	0,37	1,00	0	NA	NA	0
TRO	trophinin	0,85	-0,23	0,42	0,78	0	-1,01	-0,01	NA	NA	0	-1,12	-0,16	0
UBL3	ubiquitin-like 3	0,85	-0,23	0,33	0,72	0	-1,05	-0,07	0,82	1,00	0	-1,16	-0,22	0
C1R	complement component 1, r subcomponent	0,85	-0,23	0,41	0,77	0	-1,01	-0,01	0,97	1,00	0	-1,02	-0,02	0
TIMM9	translocase of inner mitochondrial membrane 9 homolog (yeast)	0,85	-0,23	0,31	0,70	0	-1,04	-0,06	0,59	1,00	0	NA	NA	0
IRF7	interferon regulatory factor 7	0,85	-0,23	0,48	0,81	0	-1,09	-0,13	0,43	1,00	0	-1,47	-0,55	0
IFITM2	interferon induced transmembrane protein 2 (1-8D)	0,85	-0,23	0,26	0,66	0	-1,09	-0,12	0,59	1,00	0	-1,18	-0,24	0
UBR5	ubiquitin protein ligase E3 component n-recognin 5	0,85	-0,23	0,22	0,63	0	1,11	0,15	0,43	1,00	0	NA	NA	0
VCAN	versican	0,85	-0,23	0,54	0,84	0	-1,00	-0,01	NA	NA	0	-1,52	-0,60	0
COX7C	cytochrome c oxidase subunit VIIc	0,85	-0,23	0,20	0,60	0	-1,03	-0,04	0,92	1,00	0	-1,25	-0,32	0
RAB11FIP2	RAB11 family interacting protein 2 (class I)	0,85	-0,23	0,41	0,77	0	-1,11	-0,15	0,41	1,00	0	NA	NA	0
NA	NA	0,85	-0,23	0,18	0,58	0	-1,06	-0,09	0,58	1,00	0	NA	NA	0
CABIN1	calcineurin binding protein 1	0,85	-0,23	0,31	0,70	0	-1,15	-0,20	0,14	1,00	0	1,22	0,28	0
TIA1	TIA1 cytotoxic granule-associated RNA binding protein	0,85	-0,23	0,33	0,72	0	1,09	0,12	0,80	1,00	0	-1,30	-0,38	0
MOSPD2	motile sperm domain containing 2	0,85	-0,23	0,37	0,75	0	-1,11	-0,15	0,16	1,00	0	-1,27	-0,34	0
BLVRA	biliverdin reductase A	0,85	-0,23	0,26	0,66	0	-1,03	-0,04	0,81	1,00	0	-1,14	-0,19	0
C13orf34	chromosome 13 open reading frame 34	0,85	-0,23	0,26	0,66	0	1,14	0,19	0,38	1,00	0	NA	NA	0
CASD1	CAS1 domain containing 1	0,85	-0,23	0,47	0,80	0	-1,20	-0,26	0,20	1,00	0	-1,49	-0,58	0
TRAF5	TNF receptor-associated factor 5	0,85	-0,23	0,36	0,74	0	-1,39	-0,48	0,19	1,00	0	1,11	0,15	0
CNP	2',3'-cyclic nucleotide 3' phosphodiesterase	0,85	-0,23	0,47	0,80	0	1,05	0,07	0,67	1,00	0	-1,30	-0,37	0
ITGAV	integrin, alpha V (vitronectin receptor, alpha polypeptide, antigen CD51)	0,85	-0,23	0,28	0,68	0	1,01	0,02	0,86	1,00	0	-1,10	-0,13	0
MIA3	melanoma inhibitory activity family, member 3	0,85	-0,23	0,33	0,72	0	-1,31	-0,39	0,03	1,00	0	NA	NA	0
HDGFRP3	hepatoma-derived growth factor, related protein 3	0,85	-0,24	0,18	0,59	0	-1,05	-0,08	0,63	1,00	0	NA	NA	0
ZNF238	zinc finger protein 238	0,85	-0,24	0,16	0,57	0	1,01	0,01	0,93	1,00	0	NA	NA	0
FBXO6	F-box protein 6	0,85	-0,24	0,32	0,71	0	NA	NA	NA	NA	0	NA	NA	0
FN1	fibronectin 1	0,85	-0,24	0,12	0,51	0	-1,14	-0,18	0,49	1,00	0	NA	NA	0
PCTP	phosphatidylcholine transfer protein	0,85	-0,24	0,31	0,70	0	1,06	0,09	0,44	1,00	0	NA	NA	0
HSD17B8	hydroxysteroid (17-beta) dehydrogenase 8	0,85	-0,24	0,18	0,59	0	-1,23	-0,29	0,06	1,00	0	NA	NA	0
LUC7L2	LUC7-like 2 (S. cerevisiae)	0,85	-0,24	0,20	0,61	0	-1,07	-0,10	0,27	1,00	0	NA	NA	0
PAIP1	poly(A) binding protein interacting protein 1	0,85	-0,24	0,48	0,81	0	-1,14	-0,19	0,10	1,00	0	NA	NA	0
WEE1	WEE1 homolog (S. pombe)	0,85	-0,24	0,19	0,59	0	1,07	0,10	0,31	1,00	0	NA	NA	0
ERAP1	endoplasmic reticulum aminopeptidase 1	0,85	-0,24	0,17	0,57	0	-1,02	-0,02	0,79	1,00	0	-1,14	-0,19	0
KCNE4	potassium voltage-gated channel, Isk-related family, member 4	0,85	-0,24	0,81	0,95	0	-1,01	-0,01	NA	NA	0	-1,06	-0,08	0
COL5A1	collagen, type V, alpha 1	0,85	-0,24	0,33	0,71	0	-1,35	-0,43	0,09	1,00	0	NA	NA	0
LAMA2	laminin, alpha 2	0,85	-0,24	0,53	0,84	0	-1,10	-0,14	0,64	1,00	0	-1,33	-0,42	0
FKTN	fukutin	0,85	-0,24	0,21	0,61	0	-1,10	-0,13	0,63	1,00	0	-1,10	-0,14	0
RGS10	regulator of G-protein signaling 10	0,85	-0,24	0,24	0,65	0	-1,00	0,00	NA	NA	0	0,00	0,00	0
HMGN4	high mobility group nucleosomal binding domain 4	0,85	-0,24	0,11	0,49	0	-1,07	-0,10	0,34	1,00	0	NA	NA	0
CD44	CD44 molecule (Indian blood group)	0,85	-0,24	0,58	0,86	0	-1,18	-0,24	0,54	1,00	0	NA	NA	0
PSME1	proteasome (prosome, macropain) activator subunit 1 (PA28 alpha)	0,85	-0,24	0,22	0,62	0	-1,10	-0,13	0,21	1,00	0	-1,15	-0,19	0
HIST1H2BF	histone cluster 1, H2bf	0,85	-0,24	0,25	0,65	0	1,25	0,32	0,22	1,00	0	NA	NA	0
NA	NA	0,85	-0,24	0,36	0,74	0	-1,20	-0,27	0,65	1,00	0	-1,18	-0,23	0
NA	NA	0,85	-0,24	0,32	0,71	0	-1,14	-0,19	0,24	1,00	0	NA	NA	0
ZCCHC2	zinc finger, CCHC domain containing 2	0,85	-0,24	0,21	0,61	0	1,03	0,05	0,88	1,00	0	-1,09	-0,12	0
AEBP1	AE binding protein 1	0,85	-0,24	0,29	0,69	0	1,00	0,01	0,98	1,00	0	NA	NA	0
ISOC1	isochorismatase domain containing 1	0,85	-0,24	0,25	0,65	0	1,08	0,11	0,22	1,00	0	NA	NA	0
RPIA	ribose 5-phosphate isomerase A	0,85	-0,24	0,16	0,56	0	1,07	0,09	0,37	1,00	0	NA	NA	0
FN1	fibronectin 1	0,85	-0,24	0,09	0,45	0	-1,14	-0,19	0,42	1,00	0	NA	NA	0
NAA16	N(alpha)-acetyltransferase 16, NatA auxiliary subunit	0,85	-0,24	0,23	0,64	0	1,02	0,03	0,87	1,00	0	NA	NA	0
TSPAN6	tetraspanin 6	0,85	-0,24	0,26	0,67	0	1,04	0,05	0,59	1,00	0	NA	NA	0
C1orf216	chromosome 1 open reading frame 216	0,85	-0,24	0,31	0,70	0	-1,17	-0,23	0,21	1,00	0	NA	NA	0
KIAA0240	KIAA0240	0,85	-0,24	0,20	0,60	0	-1,17	-0,23	0,25	1,00	0	NA	NA	0
R3HCC1	R3H domain and coiled-coil containing 1	0,85	-0,24	0,48	0,81	0	-1,05	-0,07	0,71	1,00	0	NA	NA	0
RNF113A	ring finger protein 113A	0,85	-0,24	0,20	0,61	0	-1,04	-0,06	0,70	1,00	0	NA	NA	0
MUC1	mucin 1, cell surface associated	0,85	-0,24	0,76	0,93	0	-1,13	-0,17	0,84	1,00	0	-1,35	-0,43	0
RALGPS2	Ral GEF with PH domain and SH3 binding motif 2	0,85	-0,24	0,30	0,69	0	-1,06	-0,09	0,88	1,00	0	NA	NA	0
ULK2	unc-51-like kinase 2 (C. elegans)	0,84	-0,24	0,11	0,49	0	-1,07	-0,10	0,55	1,00	0	NA	NA	0
ABCD3	ATP-binding cassette, sub-family D (ALD), member 3	0,84	-0,24	0,28	0,68	0	1,03	0,04	0,77	1,00	0	-1,14	-0,19	0
C11orf95	chromosome 11 open reading frame 95	0,84	-0,24	0,19	0,60	0	1,05	0,07	0,51	1,00	0	-1,18	-0,23	0

SETD6	SET domain containing 6	0,84	-0,24	0,12	0,51	0	-1,27	-0,34	0,36	1,00	0	NA	NA	0
CEP164	centrosomal protein 164kDa	0,84	-0,24	0,21	0,62	0	-1,10	-0,14	0,16	1,00	0	-1,30	-0,38	0
ITGB5	integrin, beta 5	0,84	-0,25	0,15	0,54	0	-1,21	-0,27	0,01	0,65	0	NA	NA	0
IL18BP	interleukin 18 binding protein	0,84	-0,25	0,27	0,67	0	NA	NA	NA	NA	0	NA	NA	0
TPM1	tropomyosin 1 (alpha)	0,84	-0,25	0,25	0,66	0	-1,02	-0,03	NA	NA	0	-1,02	-0,03	0
ID2	inhibitor of DNA binding 2, dominant negative helix-loop-helix protein	0,84	-0,25	0,67	0,90	0	1,19	0,25	0,36	1,00	0	NA	NA	0
TAP2	transporter 2, ATP-binding cassette, sub-family B (MDR/TAP)	0,84	-0,25	0,37	0,75	0	-1,09	-0,12	0,62	1,00	0	-1,20	-0,28	0
RIOK3	RIO kinase 3 (yeast)	0,84	-0,25	0,42	0,77	0	1,09	0,13	0,82	1,00	0	-1,28	-0,36	0
CD46	CD46 molecule, complement regulatory protein	0,84	-0,25	0,48	0,81	0	1,25	0,32	0,62	1,00	0	1,06	0,08	0
IRS2	insulin receptor substrate 2	0,84	-0,25	0,13	0,52	0	1,06	0,08	0,65	1,00	0	-1,03	-0,04	0
GPR161	G protein-coupled receptor 161	0,84	-0,25	0,48	0,81	0	-1,05	-0,07	0,81	1,00	0	-1,18	-0,23	0
AKAP11	A kinase (PRKA) anchor protein 11	0,84	-0,25	0,36	0,74	0	-1,04	-0,06	0,59	1,00	0	NA	NA	0
TRMT61B	tRNA methyltransferase 61 homolog B (S. cerevisiae)	0,84	-0,25	0,14	0,53	0	1,04	0,06	0,62	1,00	0	-1,14	-0,19	0
TMEM168	transmembrane protein 168	0,84	-0,25	0,15	0,55	0	-1,09	-0,13	0,45	1,00	0	-1,10	-0,13	0
RNF115	ring finger protein 115	0,84	-0,25	0,22	0,62	0	-1,10	-0,14	0,30	1,00	0	NA	NA	0
MTMR2	myotubularin related protein 2	0,84	-0,25	0,22	0,62	0	-1,05	-0,07	0,79	1,00	0	-1,12	-0,17	0
WNK1	WNK lysine deficient protein kinase 1	0,84	-0,25	0,09	0,44	0	-1,05	-0,06	0,63	1,00	0	NA	NA	0
DST	dystonin	0,84	-0,25	0,32	0,71	0	-1,23	-0,29	0,37	1,00	0	NA	NA	0
GSPT2	G1 to S phase transition 2	0,84	-0,25	0,32	0,71	0	-1,18	-0,24	0,45	1,00	0	NA	NA	0
METRN	meteorin, glial cell differentiation regulator	0,84	-0,25	0,20	0,60	0	-1,12	-0,17	0,24	1,00	0	NA	NA	0
TPM1	tropomyosin 1 (alpha)	0,84	-0,25	0,13	0,52	0	1,02	0,02	0,85	1,00	0	NA	NA	0
TNS1	tensin 1	0,84	-0,25	0,33	0,72	0	-1,64	-0,71	0,15	1,00	0	NA	NA	0
NEK7	NIMA (never in mitosis gene a)-related kinase 7	0,84	-0,25	0,22	0,63	0	-1,05	-0,07	0,53	1,00	0	NA	NA	0
ZNF232	zinc finger protein 232	0,84	-0,25	0,29	0,69	0	1,05	0,08	0,76	1,00	0	NA	NA	0
PTPRF	protein tyrosine phosphatase, receptor type, F	0,84	-0,25	0,39	0,76	0	-1,07	-0,10	0,36	1,00	0	1,10	0,13	0
FBLN1	fibulin 1	0,84	-0,25	0,22	0,62	0	-1,30	-0,38	0,06	1,00	0	NA	NA	0
PIGK	phosphatidylinositol glycan anchor biosynthesis, class K	0,84	-0,25	0,29	0,69	0	-1,26	-0,33	0,16	1,00	0	NA	NA	0
PLA2G4A	phospholipase A2, group IVA (cytosolic, calcium-dependent)	0,84	-0,25	0,54	0,84	0	-1,00	0,00	0,99	1,00	0	-1,27	-0,34	0
PASK	PAS domain containing serine/threonine kinase	0,84	-0,25	0,07	0,41	0	-1,07	-0,09	0,57	1,00	0	NA	NA	0
SMPDL3A	sphingomyelin phosphodiesterase, acid-like 3A	0,84	-0,25	0,32	0,71	0	-1,01	-0,01	0,96	1,00	0	NA	NA	0
TWSG1	twisted gastrulation homolog 1 (Drosophila)	0,84	-0,25	0,26	0,66	0	-1,24	-0,31	0,31	1,00	0	-1,16	-0,22	0
NA	NA	0,84	-0,25	0,16	0,56	0	NA	NA	NA	NA	0	NA	NA	0
SP110	SP110 nuclear body protein	0,84	-0,25	0,60	0,87	0	-1,02	-0,02	0,93	1,00	0	-1,20	-0,27	0
GRB10	growth factor receptor-bound protein 10	0,84	-0,25	0,38	0,75	0	-1,03	-0,05	0,85	1,00	0	NA	NA	0
ZNF107	zinc finger protein 107	0,84	-0,25	0,41	0,77	0	NA	NA	NA	NA	0	NA	NA	0
IGF1R	insulin-like growth factor 1 receptor	0,84	-0,25	0,40	0,76	0	-1,03	-0,04	0,82	1,00	0	NA	NA	0
PTS	6-pyruvoyltetrahydropterin synthase	0,84	-0,26	0,40	0,76	0	1,06	0,08	0,62	1,00	0	-1,01	-0,02	0
PXMP2	peroxisomal membrane protein 2, 22kDa	0,84	-0,26	0,35	0,73	0	-1,16	-0,21	0,20	1,00	0	NA	NA	0
CALCOCO2	calcium binding and coiled-coil domain 2	0,84	-0,26	0,10	0,48	0	1,05	0,06	0,46	1,00	0	-1,10	-0,14	0
C7orf10	chromosome 7 open reading frame 10	0,84	-0,26	0,26	0,66	0	1,02	0,03	0,93	1,00	0	-1,04	-0,06	0
CNOT7	CCR4-NOT transcription complex, subunit 7	0,84	-0,26	0,21	0,62	0	NA	NA	NA	NA	0	NA	NA	0
FN1	fibronectin 1	0,84	-0,26	0,10	0,47	0	-1,15	-0,20	0,43	1,00	0	NA	NA	0
INTS3	integrator complex subunit 3	0,84	-0,26	0,27	0,67	0	-1,18	-0,24	0,31	1,00	0	-1,20	-0,26	0
NA	NA	0,84	-0,26	0,30	0,69	0	1,02	0,03	0,90	1,00	0	-1,12	-0,17	0
FDXR	ferredoxin reductase	0,84	-0,26	0,16	0,57	0	-1,12	-0,17	0,24	1,00	0	-1,16	-0,22	0
SLC35D2	solute carrier family 35, member D2	0,84	-0,26	0,16	0,56	0	-1,05	-0,07	0,70	1,00	0	NA	NA	0
ATM	ataxia telangiectasia mutated	0,84	-0,26	0,18	0,59	0	-1,15	-0,20	0,52	1,00	0	NA	NA	0
PDS5B	PDS5, regulator of cohesion maintenance, homolog B (S. cerevisiae)	0,84	-0,26	0,19	0,60	0	-1,02	-0,03	0,89	1,00	0	NA	NA	0
PTPRM	protein tyrosine phosphatase, receptor type, M	0,84	-0,26	0,19	0,59	0	-1,11	-0,16	0,56	1,00	0	NA	NA	0
FAM168A	family with sequence similarity 168, member A	0,84	-0,26	0,14	0,54	0	-1,02	-0,03	0,87	1,00	0	-1,19	-0,24	0
SOAT1	sterol O-acyltransferase 1	0,84	-0,26	0,25	0,65	0	-1,03	-0,04	0,79	1,00	0	NA	NA	0
DMXL2	Dmx-like 2	0,84	-0,26	0,31	0,70	0	-1,09	-0,12	0,59	1,00	0	NA	NA	0
NA	NA	0,84	-0,26	0,30	0,69	0	-1,02	-0,03	0,87	1,00	0	NA	NA	0
APOBEC3C	apolipoprotein B mRNA editing enzyme, catalytic polypeptide-like 3C	0,84	-0,26	0,54	0,84	0	1,02	0,02	0,94	1,00	0	1,01	0,02	0
ATP9A	ATPase, class II, type 9A	0,84	-0,26	0,18	0,58	0	1,29	0,37	0,19	1,00	0	NA	NA	0
NA	NA	0,84	-0,26	0,23	0,63	0	-1,14	-0,19	0,12	1,00	0	NA	NA	0
IFIT5	interferon-induced protein with tetratricopeptide repeats 5	0,84	-0,26	0,21	0,62	0	-1,06	-0,09	0,64	1,00	0	-1,27	-0,35	0
HTRA1	HtrA serine peptidase 1	0,84	-0,26	0,19	0,60	0	-1,35	-0,43	0,01	0,49	0	NA	NA	0
GALT	galactose-1-phosphate uridylyltransferase	0,84	-0,26	0,12	0,51	0	-1,06	-0,09	0,44	1,00	0	0,00	0,01	0
USP15	ubiquitin specific peptidase 15	0,84	-0,26	0,18	0,59	0	1,04	0,05	0,62	1,00	0	-1,49	-0,58	0
HIST1H2BD	histone cluster 1, H2bd	0,84	-0,26	0,14	0,53	0	1,44	0,53	0,02	0,94	0	NA	NA	0
NCRNA00081	non-protein coding RNA 81	0,83	-0,26	0,21	0,61	0	-1,40	-0,48	0,19	1,00	0	NA	NA	0
CKAP2	cytoskeleton associated protein 2	0,83	-0,26	0,34	0,72	0	1,02	0,03	0,71	1,00	0	NA	NA	0
RYK	RYK receptor-like tyrosine kinase	0,83	-0,26	0,52	0,83	0	1,33	0,41	0,65	1,00	0	-1,28	-0,36	0
PLA2G12A	phospholipase A2, group XIIA	0,83	-0,26	0,16	0,56	0	1,07	0,09	0,45	1,00	0	-1,25	-0,32	0
CD302	CD302 molecule	0,83	-0,26	0,30	0,69	0	1,10	0,14	0,17	1,00	0	-1,18	-0,23	0
CAMK2N1	calcium/calmodulin-dependent protein kinase II inhibitor 1	0,83	-0,26	0,21	0,62	0	-1,08	-0,12	0,42	1,00	0	NA	NA	0
PGRMC2	progesterone receptor membrane component 2	0,83	-0,26	0,09	0,44	0	-1,24	-0,31	0,05	1,00	0	NA	NA	0
CETN3	centrin, EF-hand protein, 3	0,83	-0,26	0,24	0,64	0	1,03	0,05	0,78	1,00	0	NA	NA	0
CHPT1	choline phosphotransferase 1	0,83	-0,26	0,35	0,73	0	1,07	0,10	0,73	1,00	0	NA	NA	0
NOL12	nucleolar protein 12	0,83	-0,26	0,56	0,85	0	-1,22	-0,29	0,29	1,00	0	NA	NA	0
GXYLT2	glucoside xylosyltransferase 2	0,83	-0,26	0,26	0,66	0	NA	NA	NA	NA	0	NA	NA	0
ZFP106	zinc finger protein 106 homolog (mouse)	0,83	-0,26	0,15	0,55	0	-1,01	-0,01	0,94	1,00	0	NA	NA	0
NA	NA	0,83	-0,26	0,23	0,63	0	NA	NA	NA	NA	0	NA	NA	0
PIK3R3	phosphoinositide-3-kinase, regulatory subunit 3 (gamma)	0,83	-0,26	0,70	0,91	0	1,23	0,30	0,36	1,00	0	-1,54	-0,62	0
EFNA5	ephrin-A5	0,83	-0,26	0,29	0,69	0	1,19	0,26	0,26	1,00	0	NA	NA	0
CREBL2	cAMP responsive element binding protein-like 2	0,83	-0,26	0,21	0,62	0	-1,06	-0,08	0,70	1,00	0	-1,19	-0,25	0
TRAK2	trafficking protein, kinesin binding 2	0,83	-0,26	0,25	0,65	0	-1,00	-0,01	0,98	1,00	0	1,04	0,05	0
C2orf34	chromosome 2 open reading frame 34	0,83	-0,26	0,17	0,58	0	1,07	0,09	0,66	1,00	0	-1,05	-0,07	0
STC2	stanniocalcin 2	0,83	-0,27	0,29	0,69	0	-1,57	-0,65	0,17	1,00	0	1,30	0,37	0
NA	NA	0,83	-0,27	0,33	0,72	0	-1,01	-0,01	0,96	1,00	0	NA	NA	0
CSTF2T	cleavage stimulation factor, 3' pre-RNA, subunit 2, 64kDa, tau variant	0,83	-0,27	0,26	0,66	0	-1,06	-0,08	0,84	1,00	0	NA	NA	0
GAS2L1	growth arrest-specific 2 like 1	0,83	-0,27	0,17	0,58	0	1,04	0,06	0,71	1,00	0	NA	NA	0
CLASP2	cytoplasmic linker associated protein 2	0,83	-0,27	0,22	0,63	0	-1,12	-0,17	0,37	1,00	0	NA	NA	0
DRAM1	DNA-damage regulated autophagy modulator 1	0,83	-0,27	0,35	0,73	0	-1,20	-0,26	0,15	1,00	0	NA	NA	0
KDELR3	KDEL (Lys-Asp-Glu-Leu) endoplasmic reticulum protein retention receptor 3	0,83	-0,27	0,20	0,60	0	-1,14	-0,19	0,64	1,00	0	-1,20	-0,27	0
IRF9	interferon regulatory factor 9	0,83	-0,27	0,17	0,57	0	-1,26	-0,33	0,11	1,00	0	-1,28	-0,36	0
ARL4C	ADP-ribosylation factor-like 4C	0,83	-0,27	0,13	0,52	0	1,02	0,04	0,87	1,00	0	NA	NA	0
NA	NA	0,83	-0,27	0,26	0,66	0	-1,00	0,00	0,98	1,00	0	-1,52	-0,59	0
MRE11A	MRE11 meiotic recombination 11 homolog A (S. cerevisiae)	0,83	-0,27	0,23	0,64	0	-1,01	-0,02	0,89	1,00	0	NA	NA	0
NA	NA	0,83	-0,27	0,29	0,69	0	1,08	0,11	0,66	1,00	0	NA	NA	0
CEP70	centrosomal protein 70kDa	0,83	-0,27	0,17	0,58	0	-1,18	-0,24	0,33	1,00	0	NA	NA	0
SSPN	sarcospan (Kras oncogene-associated gene)	0,83	-0,27	0,44	0,78	0	-1,09	-0,12	0,66	1,00	0	NA	NA	0
DNAJC24	DnaJ (Hsp40) homolog, subfamily C, member 24	0,83	-0,27	0,29	0,69	0	1,01	0,02	0,94	1,00	0	-1,15	-0,20	0
ANKRD46	ankyrin repeat domain 46	0,83	-0,27	0,24	0,65	0	1,10	0,14	0,57	1,00	0	-1,18	-0,23	0
CREBL2	cAMP responsive element binding protein-like 2	0,83	-0,27	0,22	0,62	0	1,04	0,06	0,51	1,00	0	-1,23	-0,30	0
UNG	uracil-DNA glycosylase	0,83	-0,27	0,07	0,40	0	-1,07	-0,10	0,37	1,00	0	NA	NA	0
FAM115A	family with sequence similarity 115, member A	0,83	-0,27	0,49	0,81	0	-1,10	-0,13	0,56	1,00	0	-1,32	-0,39	0
PEX1	peroxisomal biogenesis factor 1	0,83	-0,27	0,15	0,55	0	-1,26	-0,34	0,03	0,97	0	NA	NA	0
NA	NA	0,83	-0,27	0,28	0,68	0	NA	NA	NA	NA	0	NA	NA	0
CASP8	caspase 8, apoptosis-related cysteine peptidase	0,83	-0,27	0,44	0,79	0	-1,17	-0,23	0,22	1,00	0	-1,45	-0,53	0
NA	NA	0,83	-0,27	0,12	0,50	0	NA	NA	NA	NA	0	NA	NA	0
GCLC	glutamate-cysteine ligase, catalytic subunit	0,83	-0,27	0,13	0,52	0	-1,01	-0,02	0,91	1,00	0	NA	NA	0
ULK2	unc-51-like kinase 2 (C. elegans)	0,83	-0,27	0,23	0,64	0	-1,15	-0,21	0,46	1,00	0	NA	NA	0
ERAP2	endoplasmic reticulum aminopeptidase 2	0,83	-0,27	0,20	0,61	0	-1,34	-0,42	0,07	1,00	0	-1,47	-0,55	0
REEP5	receptor accessory protein 5	0,83	-0,27	0,07	0,41	0	-1,03	-0,04	0,79	1,00	0	NA	NA	0
MRPS14	mitochondrial ribosomal protein S14	0,83	-0,27	0,27	0,67	0	-1,54	-0,62	0,03	0,97	0	-1,49	-0,58	0
FAT2	FAT tumor suppressor homolog 2 (Drosophila)	0,83	-0,27	0,44	0,78	0	-1,15	-0,20	0,11	1,00	0	NA	NA	0
C12orf41	chromosome 12 open reading frame 41	0,83	-0,27	0,09	0,45	0	1,06	0,09	0,35	1,00	0	NA	NA	0
EIF1AY	eukaryotic translation initiation factor 1A, Y-linked	0,83	-0,27	0,26	0,66	0	1,13	0,18	0,36	1,00	0	-1,16	-0,21	0
OGT	O-linked N-acetylglucosamine (GlcNAc) transferase (UDP-N-acetylglucosamine:polypeptide-N-acetylglucosaminyl transferase)	0,83	-0,27	0,35	0,73	0	1,06	0,09	0,77	1,00	0	-1,35	-0,44	0
B2M	beta-2-microglobulin	0,83	-0,27	0,17	0,57	0	1,02	0,03	0,74	1,00	0	NA	NA	0
PKD2	polycystic kidney disease 2 (autosomal dominant)	0,83	-0,27	0,21	0,62	0	-1,20	-0,27	0,13	1,00	0	NA	NA	0
BST1	bone marrow stromal cell antigen 1	0,83	-0,27	0,14	0,53	0	-1,01	-0,01	NA	NA	0	-1,27	-0,35	0
RIOK3	RIO kinase 3 (yeast)	0,83	-0,27	0,38	0,75	0	1,01	0,02	0,97	1,00	0	-1,27	-0,34	0
SULT1A1	sulfotransferase family, cytosolic, 1A, phenol-preferring, member 1	0,83	-0,27	0,26	0,66	0	-1,03	-0,04	0,77	1,00	0	NA	NA	0
LAMA2	laminin, alpha 2	0,83	-0,27	0,40	0,76	0	-1,17	-0,23	0,45	1,00	0	-1,54	-0,63	0
PSMB10	proteasome (prosome, macropain) subunit, beta type, 10	0,83	-0,27	0,46	0,80	0	-1,11	-0,15	0,68	1,00	0	-1,28	-0,35	0
B3GALT4	UDP-Gal:betaGlcNAc beta 1,3-galactosyltransferase, polypeptide 4	0,83	-0,27	0,48	0,81	0	-1,34	-0,42	0,32	1,00	0	NA	NA	0
APOL4	apolipoprotein L, 4	0,83	-0,27	0,63	0,88	0	NA	NA	NA	NA	0	NA	NA	0
SLC25A36	solute carrier family 25, member 36	0,83	-0,27	0,14	0,54	0	-1,01	-0,02	0,92	1,00	0	-1,37	-0,45	0
ZNF573	zinc finger protein 573	0,83	-0,27	0,23	0,63	0	1,01	0,01	0,97	1,00	0	NA	NA	0
CSF1	colony stimulating factor 1 (macrophage)	0,83	-0,27	0,38	0,75	0	-1,00	0,00	0,99	1,00	0	1,01	0,01	0
PATL2	protein associated with topoisomerase II homolog 2 (yeast)	0,83	-0,27	0,25	0,65	0	NA	NA	NA	NA	0	NA	NA	0
SPTAN1	spectrin, alpha, non-erythrocytic 1 (alpha-fodrin)	0,83	-0,27	0,27	0,67	0	-1,04	-0,05	0,88	1,00	0	NA	NA	0
MTF2	metal response element binding transcription factor 2	0,83	-0,27	0,06	0,39	0	1,03	0,04	0,82	1,00	0	-1,28	-0,35	0
AKR7A3	aldo-keto reductase family 7, member A3 (aflatoxin aldehyde reductase)	0,83	-0,27	0,05	0,35	0	-1,02	-0,03	0,84	1,00	0	NA	NA	0
BHLHB9	basic helix-loop-helix domain containing, class B, 9	0,83	-0,27	0,22	0,63	0	-1,30	-0,38	0,11	1,00	0	-1,25	-0,32	0
SLC39A8	solute carrier family 39 (zinc transporter), member 8	0,83	-0,28	0,46	0,79	0	1,20	0,27	0,16	1,00	0	-1,12	-0,17	0
C14orf138	chromosome 14 open reading frame 138	0,83	-0,28	0,26	0,66	0	1,05	0,07	0,73	1,00	0	NA	NA	0
CSRP2	cysteine and glycine-rich protein 2	0,83	-0,28	0,29	0,69	0	-1,02	-0,03	0,85	1,00	0	-1,33	-0,42	0
LHFP	lipoma HMGIC fusion partner	0,83	-0,28	0,28	0,68	0	-1,19	-0,25	0,17	1,00	0	NA	NA	0
NA	NA	0,83	-0,28	0,21	0,62	0	NA	NA	NA	NA	0	NA	NA	0
PAAF1	proteasomal ATPase-associated factor 1	0,83	-0,28	0,16	0,56	0	-1,19	-0,25	0,16	1,00	0	NA	NA	0
VWA5A	von Willebrand factor A domain containing 5A	0,83	-0,28	0,15	0,55	0	-1,52	-0,61	0,02	0,91	0	NA	NA	0
RRAGC	Ras-related GTP binding C	0,83	-0,28	0,12	0,50	0	1,11	0,16	0,28	1,00	0	NA	NA	0
YEATS4	YEATS domain containing 4	0,83	-0,28	0,12	0,51	0	-1,05	-0,07	0,65	1,00	0	NA	NA	0

ZDHHC17	zinc finger, DHHC-type containing 17	0,83	-0,28	0,25	0,65	0	-1,05	-0,06	0,52	1,00	0	-1,15	-0,20	0
SFRS18	splicing factor, arginine/serine-rich 18	0,83	-0,28	0,14	0,54	0	-1,15	-0,20	0,17	1,00	0	-1,33	-0,42	0
WWP1	WW domain containing E3 ubiquitin protein ligase 1	0,83	-0,28	0,23	0,63	0	-1,07	-0,10	0,37	1,00	0	-1,25	-0,33	0
CCDC76	coiled-coil domain containing 76	0,82	-0,28	0,19	0,59	0	-1,16	-0,21	0,15	1,00	0	NA	NA	0
ACSL3	acyl-CoA synthetase long-chain family member 3	0,82	-0,28	0,34	0,72	0	-1,15	-0,20	0,02	0,86	0	NA	NA	0
TCTN1	tectonic family member 1	0,82	-0,28	0,22	0,62	0	1,15	0,20	0,25	1,00	0	-1,23	-0,31	0
TMEM223	transmembrane protein 223	0,82	-0,28	0,09	0,45	0	-1,08	-0,11	0,52	1,00	0	-1,23	-0,30	0
ITGB3	integrin, beta 3 (platelet glycoprotein IIIa, antigen CD61)	0,82	-0,28	0,26	0,66	0	-1,01	-0,01	NA	NA	0	-1,45	-0,54	0
SOX4	SRY (sex determining region Y)-box 4	0,82	-0,28	0,48	0,81	0	-1,29	-0,37	0,34	1,00	0	NA	NA	0
TRAM2	translocation associated membrane protein 2	0,82	-0,28	0,24	0,64	0	1,04	0,05	0,80	1,00	0	NA	NA	0
CASP8AP2	caspase 8 associated protein 2	0,82	-0,28	0,20	0,61	0	-1,08	-0,11	0,40	1,00	0	NA	NA	0
DIMT1L	DIM1 dimethyladenosine transferase 1-like (S. cerevisiae)	0,82	-0,28	0,18	0,59	0	-1,04	-0,05	0,59	1,00	0	-1,05	-0,08	0
EBAG9	estrogen receptor binding site associated, antigen, 9	0,82	-0,28	0,12	0,51	0	-1,10	-0,14	0,36	1,00	0	NA	NA	0
06-set	septin 6	0,82	-0,28	0,14	0,54	0	-1,02	-0,02	NA	NA	0	-1,15	-0,20	0
TCF7L1	transcription factor 7-like 1 (T-cell specific, HMG-box)	0,82	-0,28	0,61	0,88	0	-1,18	-0,24	0,20	1,00	0	NA	NA	0
AP3M2	adaptor-related protein complex 3, mu 2 subunit	0,82	-0,28	0,12	0,50	0	-1,13	-0,18	0,51	1,00	0	NA	NA	0
ZNF266	zinc finger protein 266	0,82	-0,28	0,20	0,61	0	-1,09	-0,12	0,62	1,00	0	NA	NA	0
PDSS2	prenyl (decaprenyl) diphosphate synthase, subunit 2	0,82	-0,28	0,08	0,43	0	NA	NA	NA	NA	0	NA	NA	0
MTHFD2L	methylenetetrahydrofolate dehydrogenase (NADP+ dependent) 2-like	0,82	-0,28	0,39	0,76	0	-1,16	-0,21	0,21	1,00	0	NA	NA	0
DSC1	desmocollin 1	0,82	-0,28	0,38	0,75	0	-1,72	-0,78	0,27	1,00	0	NA	NA	0
PTPRG	protein tyrosine phosphatase, receptor type, G	0,82	-0,28	0,28	0,68	0	1,05	0,08	0,61	1,00	0	NA	NA	0
HS2ST1	heparan sulfate 2-O-sulfotransferase 1	0,82	-0,28	0,20	0,61	0	-1,24	-0,31	0,42	1,00	0	-1,15	-0,20	0
RNF14	ring finger protein 14	0,82	-0,28	0,13	0,52	0	1,06	0,08	0,70	1,00	0	NA	NA	0
ENDOD1	endonuclease domain containing 1	0,82	-0,28	0,27	0,67	0	1,01	0,01	0,96	1,00	0	-1,32	-0,40	0
06-set	septin 6	0,82	-0,28	0,59	0,86	0	-1,01	-0,01	NA	NA	0	0,00	0,00	0
TRIM24	tripartite motif-containing 24	0,82	-0,28	0,08	0,42	0	-1,19	-0,25	0,22	1,00	0	NA	NA	0
SLC35A3	solute carrier family 35 (UDP-N-acetylglucosamine (UDP-GlcNAc) transporter), member A3	0,82	-0,28	0,36	0,74	0	-1,33	-0,41	0,03	1,00	0	NA	NA	0
ZNF267	zinc finger protein 267	0,82	-0,28	0,13	0,52	0	-1,17	-0,22	0,09	1,00	0	-1,20	-0,27	0
TRAFD1	TRAF-type zinc finger domain containing 1	0,82	-0,28	0,32	0,71	0	1,15	0,20	0,39	1,00	0	1,06	0,08	0
CASP7	caspase 7, apoptosis-related cysteine peptidase	0,82	-0,28	0,26	0,66	0	1,11	0,15	0,19	1,00	0	-1,19	-0,24	0
RAGE	renal tumor antigen	0,82	-0,28	0,14	0,54	0	-1,06	-0,09	0,52	1,00	0	-1,23	-0,30	0
MCAM	melanoma cell adhesion molecule	0,82	-0,28	0,50	0,82	0	1,18	0,24	0,38	1,00	0	-1,25	-0,32	0
CP110	CP110 protein	0,82	-0,28	0,14	0,53	0	1,09	0,13	0,73	1,00	0	NA	NA	0
SULT1A1	sulfotransferase family, cytosolic, 1A, phenol-preferring, member 1	0,82	-0,28	0,24	0,64	0	-1,18	-0,24	0,25	1,00	0	NA	NA	0
MANBA	mannosidase, beta A, lysosomal	0,82	-0,28	0,08	0,42	0	-1,09	-0,12	0,53	1,00	0	NA	NA	0
BTBD3	BTB (POZ) domain containing 3	0,82	-0,28	0,07	0,42	0	-1,18	-0,24	0,29	1,00	0	NA	NA	0
SLC6A8	solute carrier family 6 (neurotransmitter transporter, creatine), member 8	0,82	-0,28	0,53	0,84	0	1,03	0,04	0,80	1,00	0	1,39	0,48	0
C6orf48	chromosome 6 open reading frame 48	0,82	-0,29	0,15	0,55	0	1,05	0,07	0,71	1,00	0	NA	NA	0
LIPA	lipase A, lysosomal acid, cholesterol esterase	0,82	-0,29	0,16	0,56	0	-1,04	-0,05	0,52	1,00	0	-1,23	-0,31	0
CDC42EP4	CDC42 effector protein (Rho GTPase binding) 4	0,82	-0,29	0,16	0,57	0	-1,08	-0,11	0,49	1,00	0	1,01	0,02	0
ENDOD1	endonuclease domain containing 1	0,82	-0,29	0,05	0,36	0	-1,06	-0,09	0,60	1,00	0	-1,16	-0,22	0
GCLC	glutamate-cysteine ligase, catalytic subunit	0,82	-0,29	0,19	0,60	0	-1,13	-0,18	0,32	1,00	0	NA	NA	0
RBM7	RNA binding motif protein 7	0,82	-0,29	0,17	0,57	0	1,22	0,29	0,29	1,00	0	NA	NA	0
NA	NA	0,82	-0,29	0,19	0,60	0	1,25	0,32	0,15	1,00	0	NA	NA	0
FN1	fibronectin 1	0,82	-0,29	0,06	0,38	0	-1,17	-0,22	0,41	1,00	0	NA	NA	0
USP46	ubiquitin specific peptidase 46	0,82	-0,29	0,25	0,65	0	1,12	0,16	0,36	1,00	0	-1,16	-0,21	0
MBP	myelin basic protein	0,82	-0,29	0,22	0,62	0	-1,02	-0,02	0,97	1,00	0	NA	NA	0
AK4	adenylate kinase 4	0,82	-0,29	0,20	0,61	0	1,01	0,02	0,92	1,00	0	NA	NA	0
FERMT2	fermitin family member 2	0,82	-0,29	0,23	0,64	0	-1,05	-0,07	0,50	1,00	0	NA	NA	0
GALNT2	UDP-N-acetyl-alpha-D-galactosamine:polypeptide N-acetylgalactosaminyltransferase 2 (GalNAc-T2)	0,82	-0,29	0,24	0,64	0	-1,07	-0,10	0,52	1,00	0	NA	NA	0
PCF11	PCF11, cleavage and polyadenylation factor subunit, homolog (S. cerevisiae)	0,82	-0,29	0,12	0,51	0	-1,22	-0,29	0,17	1,00	0	NA	NA	0
ELOVL6	ELOVL family member 6, elongation of long chain fatty acids (FEN1/Elo2, SUR4/Elo3-like, yeast)	0,82	-0,29	0,13	0,52	0	-1,01	-0,02	0,87	1,00	0	NA	NA	0
MRPL34	mitochondrial ribosomal protein L34	0,82	-0,29	0,25	0,66	0	-1,01	-0,01	0,91	1,00	0	NA	NA	0
AKAP9	A kinase (PRKA) anchor protein (yotiao) 9	0,82	-0,29	0,18	0,59	0	-1,88	-0,91	0,04	1,00	0	NA	NA	0
IRS2	insulin receptor substrate 2	0,82	-0,29	0,06	0,39	0	-1,03	-0,05	0,82	1,00	0	-1,01	-0,02	0
DMD	dystrophin	0,82	-0,29	0,22	0,62	0	-1,20	-0,26	0,14	1,00	0	-1,32	-0,40	0
FZD7	frizzled homolog 7 (Drosophila)	0,82	-0,29	0,28	0,68	0	-1,02	-0,03	0,78	1,00	0	-1,14	-0,19	0
PRPF4B	PRP4 pre-mRNA processing factor 4 homolog B (yeast)	0,82	-0,29	0,30	0,70	0	-1,04	-0,06	0,83	1,00	0	-1,08	-0,10	0
LYPD1	LY6/PLAUR domain containing 1	0,82	-0,29	0,71	0,91	0	-1,01	-0,01	NA	NA	0	-1,09	-0,12	0
PHF11	PHD finger protein 11	0,82	-0,29	0,09	0,45	0	-1,02	-0,03	0,86	1,00	0	-1,14	-0,19	0
MR1	major histocompatibility complex, class I-related	0,82	-0,29	0,29	0,69	0	-1,08	-0,10	0,57	1,00	0	-1,41	-0,49	0
SNUPN	snurportin 1	0,82	-0,29	0,07	0,40	0	-1,10	-0,14	0,54	1,00	0	NA	NA	0
ZNF580	zinc finger protein 580	0,82	-0,29	0,14	0,53	0	-1,24	-0,31	0,25	1,00	0	NA	NA	0
NA	NA	0,82	-0,29	0,27	0,67	0	NA	NA	NA	NA	0	NA	NA	0
FADS3	fatty acid desaturase 3	0,82	-0,29	0,23	0,63	0	1,31	0,39	0,11	1,00	0	NA	NA	0
SLC25A28	solute carrier family 25, member 28	0,82	-0,29	0,10	0,48	0	1,01	0,01	0,96	1,00	0	-1,03	-0,04	0
CXCL10	chemokine (C-X-C motif) ligand 10	0,82	-0,29	0,16	0,56	0	-1,03	-0,04	0,95	1,00	0	1,01	0,01	0
PHKB	phosphorylase kinase, beta	0,82	-0,29	0,40	0,76	0	-1,17	-0,23	0,18	1,00	0	NA	NA	0
ZC3HAV1	zinc finger CCCH-type, antiviral 1	0,82	-0,29	0,25	0,66	0	1,05	0,07	0,51	1,00	0	NA	NA	0
STOX2	storkhead box 2	0,82	-0,29	0,21	0,62	0	NA	NA	NA	NA	0	NA	NA	0
MTMR2	myotubularin related protein 2	0,82	-0,29	0,11	0,50	0	-1,02	-0,03	0,91	1,00	0	1,03	0,04	0
AKAP7	A kinase (PRKA) anchor protein 7	0,82	-0,29	0,16	0,56	0	-1,18	-0,24	0,19	1,00	0	-1,23	-0,30	0
LOC100310756	hypothetical LOC100310756	0,82	-0,29	0,19	0,60	0	NA	NA	NA	NA	0	NA	NA	0
OPTN	optineurin	0,82	-0,29	0,21	0,62	0	-1,10	-0,13	0,33	1,00	0	-1,02	-0,02	0
GRINA	glutamate receptor, ionotropic, N-methyl D-aspartate-associated protein 1 (glutamate binding)	0,82	-0,29	0,13	0,53	0	-1,34	-0,42	0,06	1,00	0	2,35	1,23	0
TIA1	TIA1 cytotoxic granule-associated RNA binding protein	0,82	-0,29	0,12	0,50	0	-1,05	-0,07	0,79	1,00	0	NA	NA	0
NFATC3	nuclear factor of activated T-cells, cytoplasmic, calcineurin-dependent 3	0,82	-0,29	0,23	0,64	0	-1,26	-0,34	0,07	1,00	0	NA	NA	0
SFPQ	splicing factor proline/glutamine-rich	0,82	-0,29	0,39	0,75	0	-1,15	-0,20	0,54	1,00	0	-1,10	-0,14	0
DYNLT3	dynein, light chain, Tctex-type 3	0,82	-0,29	0,07	0,40	0	1,08	0,11	0,46	1,00	0	-1,32	-0,40	0
C18orf54	chromosome 18 open reading frame 54	0,82	-0,29	0,21	0,61	0	NA	NA	NA	NA	0	NA	NA	0
CEP57	centrosomal protein 57kDa	0,82	-0,30	0,31	0,70	0	1,00	0,01	0,99	1,00	0	-1,27	-0,33	0
ELMO2	engulfment and cell motility 2	0,81	-0,30	0,13	0,53	0	-1,10	-0,14	0,38	1,00	0	1,08	0,12	0
WNK1	WNK lysine deficient protein kinase 1	0,81	-0,30	0,03	0,30	0	-1,23	-0,29	0,24	1,00	0	NA	NA	0
NGDN	neuroguidin, EIF4E binding protein	0,81	-0,30	0,08	0,43	0	-1,01	-0,02	NA	NA	0	NA	NA	0
MNS1	meiosis-specific nuclear structural 1	0,81	-0,30	0,05	0,37	0	1,14	0,19	0,44	1,00	0	NA	NA	0
NQO1	NAD(P)H dehydrogenase, quinone 1	0,81	-0,30	0,25	0,65	0	-1,27	-0,34	0,20	1,00	0	NA	NA	0
PCBP2	poly(rC) binding protein 2	0,81	-0,30	0,45	0,79	0	-1,03	-0,04	0,81	1,00	0	-1,19	-0,25	0
FAM172A	family with sequence similarity 172, member A	0,81	-0,30	0,11	0,50	0	-1,01	-0,01	0,94	1,00	0	-1,54	-0,63	0
FN1	fibronectin 1	0,81	-0,30	0,10	0,48	0	-1,49	-0,58	0,23	1,00	0	NA	NA	0
NAA40	N(alpha)-acetyltransferase 40, NatD catalytic subunit, homolog (S. cerevisiae)	0,81	-0,30	0,20	0,60	0	-1,03	-0,04	0,83	1,00	0	NA	NA	0
SLCO3A1	solute carrier organic anion transporter family, member 3A1	0,81	-0,30	0,34	0,72	0	-1,04	-0,06	0,79	1,00	0	NA	NA	0
MAN1A1	mannosidase, alpha, class 1A, member 1	0,81	-0,30	0,39	0,76	0	-1,02	-0,03	0,85	1,00	0	NA	NA	0
C5orf28	chromosome 5 open reading frame 28	0,81	-0,30	0,38	0,75	0	1,08	0,12	0,33	1,00	0	-1,19	-0,26	0
LEPREL4	leprecan-like 4	0,81	-0,30	0,11	0,49	0	1,19	0,25	0,50	1,00	0	-1,06	-0,09	0
ALDH6A1	aldehyde dehydrogenase 6 family, member A1	0,81	-0,30	0,17	0,57	0	-1,16	-0,21	0,23	1,00	0	NA	NA	0
TSTA3	tissue specific transplantation antigen P35B	0,81	-0,30	0,23	0,63	0	1,05	0,07	0,58	1,00	0	NA	NA	0
VPS13C	vacuolar protein sorting 13 homolog C (S. cerevisiae)	0,81	-0,30	0,15	0,54	0	-1,09	-0,13	0,44	1,00	0	-1,16	-0,22	0
ZNF395	zinc finger protein 395	0,81	-0,30	0,17	0,57	0	1,02	0,03	0,82	1,00	0	-1,35	-0,44	0
OSGEPL1	O-sialoglycoprotein endopeptidase-like 1	0,81	-0,30	0,14	0,53	0	-1,29	-0,37	0,36	1,00	0	NA	NA	0
POP5	processing of precursor 5, ribonuclease P/MRP subunit (S. cerevisiae)	0,81	-0,30	0,16	0,56	0	-1,05	-0,07	0,70	1,00	0	NA	NA	0
FAP	fibroblast activation protein, alpha	0,81	-0,30	0,29	0,68	0	-1,39	-0,48	0,29	1,00	0	-1,09	-0,11	0
OBFC1	oligonucleotide/oligosaccharide-binding fold containing 1	0,81	-0,30	0,21	0,62	0	-1,08	-0,11	0,43	1,00	0	-1,37	-0,45	0
PSMB8	proteasome (prosome, macropain) subunit, beta type, 8 (large multifunctional peptidase 7)	0,81	-0,30	0,18	0,59	0	-1,01	-0,02	0,93	1,00	0	-1,25	-0,33	0
IL10RB	interleukin 10 receptor, beta	0,81	-0,30	0,07	0,41	0	-1,20	-0,26	0,25	1,00	0	NA	NA	0
SSX2IP	synovial sarcoma, X breakpoint 2 interacting protein	0,81	-0,30	0,14	0,53	0	-1,05	-0,07	0,69	1,00	0	-1,35	-0,43	0
TRIM69	tripartite motif-containing 69	0,81	-0,30	0,22	0,63	0	NA	NA	NA	NA	0	NA	NA	0
IFIT5	interferon-induced protein with tetratricopeptide repeats 5	0,81	-0,30	0,15	0,55	0	-1,65	-0,73	0,12	1,00	0	-1,28	-0,36	0
PLIN2	perilipin 2	0,81	-0,30	0,35	0,73	0	1,02	0,02	0,94	1,00	0	NA	NA	0
LRRC37A2	leucine rich repeat containing 37, member A2	0,81	-0,30	0,19	0,60	0	-1,00	-0,01	0,98	1,00	0	1,08	0,11	0
HAPLN3	hyaluronan and proteoglycan link protein 3	0,81	-0,30	0,42	0,77	0	NA	NA	NA	NA	0	NA	NA	0
RALA	v-ral simian leukemia viral oncogene homolog A (ras related)	0,81	-0,30	0,21	0,62	0	1,07	0,10	0,45	1,00	0	NA	NA	0
NA	NA	0,81	-0,30	0,04	0,34	0	-1,09	-0,12	0,22	1,00	0	NA	NA	0
LAMA2	laminin, alpha 2	0,81	-0,30	0,41	0,77	0	-1,29	-0,37	0,22	1,00	0	-1,37	-0,46	0
GLT8D1	glycosyltransferase 8 domain containing 1	0,81	-0,30	0,10	0,47	0	-1,05	-0,07	0,57	1,00	0	NA	NA	0
PRKRA	protein kinase, interferon-inducible double stranded RNA dependent activator	0,81	-0,30	0,12	0,51	0	1,02	0,03	0,74	1,00	0	-1,12	-0,17	0
PKD1	polycystic kidney disease 1 (autosomal dominant)	0,81	-0,30	0,11	0,50	0	-1,10	-0,14	0,58	1,00	0	NA	NA	0
OXCT1	3-oxoacid CoA transferase 1	0,81	-0,30	0,16	0,57	0	-1,09	-0,12	0,35	1,00	0	NA	NA	0
MICALL2	MICAL-like 2	0,81	-0,30	0,12	0,50	0	-1,07	-0,10	0,64	1,00	0	NA	NA	0
SNRPN	small nuclear ribonucleoprotein polypeptide N	0,81	-0,30	0,18	0,58	0	NA	NA	NA	NA	0	NA	NA	0
PIAS2	protein inhibitor of activated STAT, 2	0,81	-0,30	0,15	0,55	0	NA	NA	NA	NA	0	NA	NA	0
CEP57	centrosomal protein 57kDa	0,81	-0,30	0,11	0,49	0	-1,14	-0,19	0,23	1,00	0	NA	NA	0
PLS1	plastin 1	0,81	-0,30	0,58	0,86	0	1,07	0,10	0,56	1,00	0	NA	NA	0
C5orf13	chromosome 5 open reading frame 13	0,81	-0,30	0,31	0,70	0	-1,02	-0,03	0,83	1,00	0	1,03	0,05	0
RHOC	ras homolog gene family, member C	0,81	-0,31	0,28	0,68	0	NA	NA	NA	NA	0	NA	NA	0
C16orf5	chromosome 16 open reading frame 5	0,81	-0,31	0,15	0,55	0	1,18	0,23	0,35	1,00	0	NA	NA	0
SPAST	spastin	0,81	-0,31	0,14	0,54	0	-1,14	-0,19	0,81	1,00	0	-1,52	-0,59	0
SIN3B	SIN3 homolog B, transcription regulator (yeast)	0,81	-0,31	0,10	0,47	0	1,22	0,29	0,40	1,00	0	NA	NA	0
SLC12A7	solute carrier family 12 (potassium/chloride transporters), member 7	0,81	-0,31	0,30	0,69	0	-1,02	-0,03	0,92	1,00	0	1,09	0,12	0
C1GALT1C1	C1GALT1-specific chaperone 1	0,81	-0,31	0,10	0,47	0	NA	NA	NA	NA	0	NA	NA	0
PGRMC2	progesterone receptor membrane component 2	0,81	-0,31	0,10	0,47	0	-1,07	-0,09	0,45	1,00	0	-1,19	-0,26	0
LOC100129361	hypothetical LOC100129361	0,81	-0,31	0,17	0,57	0	1,03	0,04	0,80	1,00	0	NA	NA	0
PRKCA	protein kinase C, alpha	0,81	-0,31	0,30	0,69	0	-1,25	-0,33	0,54	1,00	0	1,01	0,02	0
CCDC41	coiled-coil domain containing 41	0,81	-0,31	0,36	0,73	0	-1,28	-0,36	0,26	1,00	0	-1,52	-0,61	0
GGA2	golgi-associated, gamma adaptin ear containing, ARF binding protein 2	0,81	-0,31	0,06	0,39	0	1,06	0,08	0,53	1,00	0	NA	NA	0
EML1	echinoderm microtubule associated protein like 1	0,81	-0,31	0,19	0,59	0	1,53	0,62	0,04	1,00	0	1,39	0,48	0
CASP4	caspase 4, apoptosis-related cysteine peptidase	0,81	-0,31	0,40	0,76	0	1,03	0,05	0,87	1,00	0	NA	NA	0
NA	NA	0,81	-0,31	0,32	0,71	0	1,38	0,47	0,17	1,00	0	-1,41	-0,49	0

SRSF7	serine/arginine-rich splicing factor 7	0,81	-0,31	0,33	0,71	0	1,28	0,36	0,32	1,00	0	-1,32	-0,39	0
CEP192	centrosomal protein 192kDa	0,81	-0,31	0,25	0,66	0	-1,08	-0,11	0,70	1,00	0	NA	NA	0
GSTT1	glutathione S-transferase theta 1	0,81	-0,31	0,55	0,85	0	1,09	0,13	0,92	1,00	0	-1,14	-0,19	0
ITGB5	integrin, beta 5	0,81	-0,31	0,07	0,42	0	-1,18	-0,24	0,16	1,00	0	NA	NA	0
GLT8D1	glycosyltransferase 8 domain containing 1	0,81	-0,31	0,11	0,50	0	-1,19	-0,25	0,16	1,00	0	NA	NA	0
CDS2	CDP-diacylglycerol synthase (phosphatidate cytidylyltransferase) 2	0,81	-0,31	0,34	0,72	0	1,01	0,02	0,92	1,00	0	NA	NA	0
SMCHD1	structural maintenance of chromosomes flexible hinge domain containing 1	0,81	-0,31	0,15	0,55	0	-1,21	-0,27	0,41	1,00	0	-1,16	-0,23	0
SYNE2	spectrin repeat containing, nuclear envelope 2	0,81	-0,31	0,23	0,64	0	-1,47	-0,56	0,04	1,00	0	-1,27	-0,33	0
TAP2	transporter 2, ATP-binding cassette, sub-family B (MDR/TAP)	0,81	-0,31	0,42	0,77	0	NA	NA	NA	NA	0	NA	NA	0
CROCCP2	ciliary rootlet coiled-coil, rootletin pseudogene 2	0,81	-0,31	0,13	0,52	0	-1,09	-0,13	0,50	1,00	0	NA	NA	0
FAM199X	family with sequence similarity 199, X-linked	0,81	-0,31	0,11	0,48	0	NA	NA	NA	NA	0	NA	NA	0
NDUFA5	NADH dehydrogenase (ubiquinone) 1 alpha subcomplex, 5, 13kDa	0,81	-0,31	0,26	0,66	0	-1,04	-0,05	0,65	1,00	0	NA	NA	0
NA	NA	0,81	-0,31	0,14	0,54	0	-1,08	-0,11	0,66	1,00	0	NA	NA	0
ARID4A	AT rich interactive domain 4A (RBP1-like)	0,81	-0,31	0,09	0,44	0	-1,26	-0,33	0,05	1,00	0	-1,04	-0,06	0
NA	NA	0,81	-0,31	0,08	0,43	0	NA	NA	NA	NA	0	NA	NA	0
UCP2	uncoupling protein 2 (mitochondrial, proton carrier)	0,80	-0,31	0,53	0,84	0	1,02	0,02	0,87	1,00	0	1,59	0,67	0
GMNN	geminin, DNA replication inhibitor	0,80	-0,31	0,30	0,70	0	-1,05	-0,07	0,68	1,00	0	NA	NA	0
C1orf109	chromosome 1 open reading frame 109	0,80	-0,31	0,32	0,71	0	-1,05	-0,07	0,56	1,00	0	NA	NA	0
NUPR1	nuclear protein, transcriptional regulator, 1	0,80	-0,31	0,09	0,44	0	-1,22	-0,29	0,26	1,00	0	NA	NA	0
MAP1B	microtubule-associated protein 1B	0,80	-0,31	0,16	0,56	0	1,05	0,07	0,83	1,00	0	1,10	0,13	0
CEP76	centrosomal protein 76kDa	0,80	-0,31	0,36	0,74	0	1,15	0,20	0,26	1,00	0	NA	NA	0
BLVRA	biliverdin reductase A	0,80	-0,31	0,25	0,65	0	-1,09	-0,13	0,44	1,00	0	-1,22	-0,28	0
ADM	adrenomedullin	0,80	-0,31	0,04	0,34	0	1,27	0,34	0,12	1,00	0	NA	NA	0
ZBED5	zinc finger, BED-type containing 5	0,80	-0,31	0,05	0,37	0	-1,24	-0,31	0,02	0,94	0	NA	NA	0
RNF14	ring finger protein 14	0,80	-0,31	0,32	0,71	0	-1,03	-0,04	0,68	1,00	0	NA	NA	0
TRPC1	transient receptor potential cation channel, subfamily C, member 1	0,80	-0,31	0,32	0,71	0	1,17	0,22	0,27	1,00	0	-1,32	-0,39	0
GYPC	glycophorin C (Gerbich blood group)	0,80	-0,31	0,36	0,74	0	-1,01	-0,02	NA	NA	0	1,21	0,27	0
C5orf30	chromosome 5 open reading frame 30	0,80	-0,31	0,09	0,45	0	1,01	0,02	0,91	1,00	0	NA	NA	0
ZBTB6	zinc finger and BTB domain containing 6	0,80	-0,32	0,20	0,61	0	1,30	0,38	0,55	1,00	0	-1,43	-0,51	0
NR3C1	nuclear receptor subfamily 3, group C, member 1 (glucocorticoid receptor)	0,80	-0,32	0,07	0,40	0	-1,19	-0,25	0,03	1,00	0	-1,20	-0,28	0
LYST	lysosomal trafficking regulator	0,80	-0,32	0,11	0,49	0	-1,11	-0,15	0,45	1,00	0	1,21	0,28	0
PCMTD1	protein-L-isoaspartate (D-aspartate) O-methyltransferase domain containing 1	0,80	-0,32	0,14	0,53	0	NA	NA	NA	NA	0	NA	NA	0
SFRS18	splicing factor, arginine/serine-rich 18	0,80	-0,32	0,08	0,43	0	-1,15	-0,20	0,48	1,00	0	-1,35	-0,44	0
CES2	carboxylesterase 2 (intestine, liver)	0,80	-0,32	0,50	0,82	0	-1,21	-0,28	0,27	1,00	0	NA	NA	0
NA	NA	0,80	-0,32	0,21	0,61	0	-1,10	-0,13	0,21	1,00	0	NA	NA	0
GCFC1	GC-rich sequence DNA-binding factor 1	0,80	-0,32	0,07	0,41	0	1,08	0,11	0,64	1,00	0	-1,28	-0,36	0
ACE2	angiotensin I converting enzyme (peptidyl-dipeptidase A) 2	0,80	-0,32	0,68	0,90	0	-1,01	-0,01	0,99	1,00	0	NA	NA	0
PHKA2	phosphorylase kinase, alpha 2 (liver)	0,80	-0,32	0,26	0,66	0	-1,09	-0,13	0,39	1,00	0	1,02	0,03	0
LOC728855	hypothetical LOC728855	0,80	-0,32	0,02	0,28	0	-1,03	-0,05	0,79	1,00	0	-1,16	-0,22	0
TMEM5	transmembrane protein 5	0,80	-0,32	0,16	0,56	0	-1,03	-0,05	0,72	1,00	0	NA	NA	0
SRSF11	serine/arginine-rich splicing factor 11	0,80	-0,32	0,14	0,53	0	-1,06	-0,09	0,62	1,00	0	-1,28	-0,35	0
IFI27	interferon, alpha-inducible protein 27	0,80	-0,32	0,40	0,76	0	-1,13	-0,18	0,78	1,00	0	-1,01	-0,01	0
SF3A2	splicing factor 3a, subunit 2, 66kDa	0,80	-0,32	0,18	0,59	0	-1,01	-0,02	0,97	1,00	0	NA	NA	0
PDE4DIP	phosphodiesterase 4D interacting protein	0,80	-0,32	0,41	0,77	0	-1,40	-0,48	0,20	1,00	0	NA	NA	0
FAM55C	family with sequence similarity 55, member C	0,80	-0,32	0,14	0,53	0	-1,22	-0,29	0,23	1,00	0	-1,22	-0,29	0
TRIM38	tripartite motif-containing 38	0,80	-0,32	0,38	0,75	0	-1,09	-0,12	0,39	1,00	0	-1,20	-0,27	0
NNMT	nicotinamide N-methyltransferase	0,80	-0,32	0,38	0,75	0	-1,00	0,00	1,00	1,00	0	-1,28	-0,36	0
C4orf27	chromosome 4 open reading frame 27	0,80	-0,32	0,06	0,38	0	-1,20	-0,27	0,06	1,00	0	NA	NA	0
PHIP	pleckstrin homology domain interacting protein	0,80	-0,32	0,10	0,47	0	-1,08	-0,11	0,55	1,00	0	NA	NA	0
VCAN	versican	0,80	-0,32	0,47	0,80	0	-1,02	-0,03	NA	NA	0	-1,54	-0,63	0
CSPG4	chondroitin sulfate proteoglycan 4	0,80	-0,32	0,15	0,55	0	-1,03	-0,05	NA	NA	0	1,40	0,49	0
FAM149B1	family with sequence similarity 149, member B1	0,80	-0,32	0,06	0,38	0	-1,03	-0,04	0,80	1,00	0	-1,14	-0,18	0
NA	NA	0,80	-0,32	0,18	0,58	0	-1,09	-0,12	0,53	1,00	0	NA	NA	0
C7orf23	chromosome 7 open reading frame 23	0,80	-0,32	0,13	0,52	0	-1,05	-0,08	0,62	1,00	0	NA	NA	0
TRIT1	tRNA isopentenyltransferase 1	0,80	-0,32	0,10	0,48	0	-1,09	-0,12	0,29	1,00	0	NA	NA	0
TAF1D	TATA box binding protein (TBP)-associated factor, RNA polymerase I, D, 41kDa	0,80	-0,32	0,36	0,73	0	1,04	0,05	0,83	1,00	0	-1,49	-0,57	0
NA	NA	0,80	-0,32	0,14	0,54	0	-1,00	-0,01	NA	NA	0	-1,23	-0,31	0
TBC1D15	TBC1 domain family, member 15	0,80	-0,32	0,10	0,48	0	-1,18	-0,24	0,39	1,00	0	NA	NA	0
XPC	xeroderma pigmentosum, complementation group C	0,80	-0,32	0,04	0,34	0	-1,23	-0,30	0,03	1,00	0	NA	NA	0
TRIAP1	TP53 regulated inhibitor of apoptosis 1	0,80	-0,32	0,15	0,54	0	-1,09	-0,13	0,41	1,00	0	NA	NA	0
FAM117A	family with sequence similarity 117, member A	0,80	-0,32	0,17	0,57	0	-1,09	-0,12	0,52	1,00	0	NA	NA	0
TGFB1I1	transforming growth factor beta 1 induced transcript 1	0,80	-0,32	0,09	0,45	0	-1,33	-0,41	0,03	0,99	0	NA	NA	0
CEP290	centrosomal protein 290kDa	0,80	-0,32	0,23	0,64	0	-1,07	-0,10	0,63	1,00	0	-1,43	-0,52	0
PRODH	proline dehydrogenase (oxidase) 1	0,80	-0,32	0,07	0,42	0	-1,90	-0,92	0,01	0,53	0	NA	NA	0
CTDSPL	CTD (carboxy-terminal domain, RNA polymerase II, polypeptide A) small phosphatase-like	0,80	-0,32	0,20	0,60	0	-1,31	-0,39	0,10	1,00	0	NA	NA	0
NA	NA	0,80	-0,33	0,13	0,52	0	NA	NA	NA	NA	0	NA	NA	0
DNAJC6	DnaJ (Hsp40) homolog, subfamily C, member 6	0,80	-0,33	0,39	0,75	0	1,11	0,15	0,51	1,00	0	0,00	0,01	0
DYRK3	dual-specificity tyrosine-(Y)-phosphorylation regulated kinase 3	0,80	-0,33	0,42	0,78	0	-1,07	-0,10	0,60	1,00	0	NA	NA	0
HLA-G	major histocompatibility complex, class I, G	0,80	-0,33	0,26	0,66	0	-1,08	-0,11	0,70	1,00	0	1,03	0,04	0
ATG12	ATG12 autophagy related 12 homolog (S. cerevisiae)	0,80	-0,33	0,07	0,41	0	-1,06	-0,08	0,63	1,00	0	NA	NA	0
SRR	serine racemase	0,80	-0,33	0,12	0,50	0	1,06	0,09	0,66	1,00	0	NA	NA	0
TSPAN6	tetraspanin 6	0,80	-0,33	0,08	0,44	0	1,04	0,05	0,67	1,00	0	NA	NA	0
TFCP2L1	transcription factor CP2-like 1	0,80	-0,33	0,41	0,77	0	-1,03	-0,04	0,79	1,00	0	NA	NA	0
CNDP2	CNDP dipeptidase 2 (metallopeptidase M20 family)	0,80	-0,33	0,18	0,59	0	-1,06	-0,09	0,41	1,00	0	-1,19	-0,26	0
CD46	CD46 molecule, complement regulatory protein	0,80	-0,33	0,25	0,65	0	1,08	0,12	0,84	1,00	0	1,02	0,03	0
MLLT11	myeloid/lymphoid or mixed-lineage leukemia (trithorax homolog, Drosophila); translocated to, 11	0,80	-0,33	0,08	0,42	0	1,00	0,01	0,97	1,00	0	-1,11	-0,15	0
ASF1A	ASF1 anti-silencing function 1 homolog A (S. cerevisiae)	0,80	-0,33	0,04	0,32	0	1,01	0,02	0,89	1,00	0	NA	NA	0
LRCH3	leucine-rich repeats and calponin homology (CH) domain containing 3	0,80	-0,33	0,22	0,63	0	-1,13	-0,17	0,54	1,00	0	-1,11	-0,16	0
SMC2	structural maintenance of chromosomes 2	0,80	-0,33	0,19	0,60	0	-1,14	-0,19	0,52	1,00	0	-1,27	-0,34	0
GTDC1	glycosyltransferase-like domain containing 1	0,80	-0,33	0,08	0,43	0	-1,18	-0,24	0,11	1,00	0	-1,12	-0,17	0
SAC3D1	SAC3 domain containing 1	0,80	-0,33	0,09	0,45	0	-1,05	-0,08	0,66	1,00	0	NA	NA	0
SWAP70	SWAP switching B-cell complex 70kDa subunit	0,80	-0,33	0,09	0,45	0	-1,03	-0,04	0,64	1,00	0	1,02	0,03	0
C1orf156	chromosome 1 open reading frame 156	0,80	-0,33	0,12	0,51	0	-1,15	-0,20	0,28	1,00	0	NA	NA	0
TGFBI	transforming growth factor, beta-induced, 68kDa	0,80	-0,33	0,17	0,58	0	1,03	0,04	0,83	1,00	0	NA	NA	0
DTX4	deltex homolog 4 (Drosophila)	0,80	-0,33	0,50	0,82	0	-1,00	0,00	0,99	1,00	0	NA	NA	0
DPY19L2	dpy-19-like 2 (C. elegans)	0,80	-0,33	0,23	0,64	0	NA	NA	NA	NA	0	NA	NA	0
NPRL2	nitrogen permease regulator-like 2 (S. cerevisiae)	0,80	-0,33	0,11	0,49	0	-1,09	-0,12	0,43	1,00	0	NA	NA	0
HAUS4	HAUS augmin-like complex, subunit 4	0,80	-0,33	0,03	0,30	0	-1,17	-0,23	0,05	1,00	0	NA	NA	0
CTNNAL1	catenin (cadherin-associated protein), alpha-like 1	0,80	-0,33	0,18	0,58	0	-1,07	-0,09	0,49	1,00	0	NA	NA	0
THBS1	thrombospondin 1	0,80	-0,33	0,19	0,60	0	-1,13	-0,18	0,62	1,00	0	-1,10	-0,13	0
RMND1	required for meiotic nuclear division 1 homolog (S. cerevisiae)	0,80	-0,33	0,03	0,29	0	-1,18	-0,24	0,20	1,00	0	NA	NA	0
C12orf52	chromosome 12 open reading frame 52	0,80	-0,33	0,22	0,63	0	1,22	0,29	0,15	1,00	0	NA	NA	0
THBD	thrombomodulin	0,80	-0,33	0,51	0,83	0	1,05	0,07	0,74	1,00	0	NA	NA	0
HS2ST1	heparan sulfate 2-O-sulfotransferase 1	0,80	-0,33	0,09	0,44	0	-1,13	-0,18	0,42	1,00	0	1,02	0,02	0
TMUB2	transmembrane and ubiquitin-like domain containing 2	0,80	-0,33	0,06	0,40	0	-1,12	-0,17	0,18	1,00	0	NA	NA	0
MEGF9	multiple EGF-like-domains 9	0,80	-0,33	0,07	0,42	0	1,00	0,01	0,97	1,00	0	-1,20	-0,27	0
PDE4DIP	phosphodiesterase 4D interacting protein	0,79	-0,33	0,13	0,52	0	-1,39	-0,47	0,04	1,00	0	NA	NA	0
TCF12	transcription factor 12	0,79	-0,33	0,09	0,46	0	-1,09	-0,13	0,36	1,00	0	NA	NA	0
MVP	major vault protein	0,79	-0,33	0,34	0,72	0	-1,07	-0,10	0,53	1,00	0	1,10	0,14	0
TCF7L2	transcription factor 7-like 2 (T-cell specific, HMG-box)	0,79	-0,33	0,10	0,47	0	-1,05	-0,07	0,66	1,00	0	NA	NA	0
DNA2	DNA replication helicase 2 homolog (yeast)	0,79	-0,33	0,31	0,70	0	1,02	0,03	0,95	1,00	0	-1,52	-0,60	0
MBOAT2	membrane bound O-acyltransferase domain containing 2	0,79	-0,33	0,12	0,51	0	-1,11	-0,15	0,40	1,00	0	NA	NA	0
NR2F2	nuclear receptor subfamily 2, group F, member 2	0,79	-0,33	0,05	0,36	0	-1,19	-0,25	0,01	0,74	0	NA	NA	0
KIAA0368	KIAA0368	0,79	-0,33	0,07	0,41	0	NA	NA	NA	NA	0	NA	NA	0
CTTN	cortactin	0,79	-0,33	0,32	0,71	0	1,08	0,10	0,68	1,00	0	NA	NA	0
ARL3	ADP-ribosylation factor-like 3	0,79	-0,34	0,14	0,54	0	-1,09	-0,13	0,46	1,00	0	NA	NA	0
FAHD2A	fumarylacetoacetate hydrolase domain containing 2A	0,79	-0,34	0,05	0,36	0	-1,11	-0,14	0,43	1,00	0	NA	NA	0
ZNF280C	zinc finger protein 280C	0,79	-0,34	0,09	0,46	0	NA	NA	NA	NA	0	NA	NA	0
DLEU2	deleted in lymphocytic leukemia 2 (non-protein coding)	0,79	-0,34	0,15	0,55	0	-1,04	-0,05	0,75	1,00	0	-1,41	-0,49	0
PDGFC	platelet derived growth factor C	0,79	-0,34	0,07	0,42	0	-1,21	-0,27	0,14	1,00	0	-1,05	-0,08	0
IQCB1	IQ motif containing B1	0,79	-0,34	0,18	0,59	0	-1,04	-0,05	0,70	1,00	0	NA	NA	0
RHOBTB3	Rho-related BTB domain containing 3	0,79	-0,34	0,28	0,68	0	-1,13	-0,18	0,63	1,00	0	NA	NA	0
HS2ST1	heparan sulfate 2-O-sulfotransferase 1	0,79	-0,34	0,22	0,63	0	-1,20	-0,26	0,24	1,00	0	-1,11	-0,16	0
ELMO2	engulfment and cell motility 2	0,79	-0,34	0,04	0,33	0	-1,01	-0,02	0,91	1,00	0	0,00	-0,01	0
ZNF302	zinc finger protein 302	0,79	-0,34	0,08	0,42	0	-1,25	-0,32	0,05	1,00	0	NA	NA	0
AASDHPPT	aminoadipate-semialdehyde dehydrogenase-phosphopantetheinyl transferase	0,79	-0,34	0,16	0,56	0	-1,20	-0,26	0,36	1,00	0	NA	NA	0
SNAPC5	small nuclear RNA activating complex, polypeptide 5, 19kDa	0,79	-0,34	0,21	0,62	0	-1,17	-0,22	0,41	1,00	0	NA	NA	0
WDR19	WD repeat domain 19	0,79	-0,34	0,11	0,48	0	-1,05	-0,08	0,83	1,00	0	NA	NA	0
HSF2	heat shock transcription factor 2	0,79	-0,34	0,12	0,50	0	-1,29	-0,36	0,01	0,82	0	NA	NA	0
PEG10	paternally expressed 10	0,79	-0,34	0,19	0,59	0	1,01	0,01	0,98	1,00	0	-1,11	-0,14	0
TCEAL4	transcription elongation factor A (SII)-like 4	0,79	-0,34	0,09	0,45	0	-1,14	-0,19	0,05	1,00	0	-1,08	-0,10	0
DYRK2	dual-specificity tyrosine-(Y)-phosphorylation regulated kinase 2	0,79	-0,34	0,30	0,69	0	-1,06	-0,08	0,74	1,00	0	-1,30	-0,38	0
DKFZp434H141	hypothetical protein DKFZp434H1419	0,79	-0,34	0,46	0,80	0	1,02	0,02	0,90	1,00	0	1,30	0,38	0
TBC1D4	TBC1 domain family, member 4	0,79	-0,34	0,08	0,43	0	-1,07	-0,10	0,57	1,00	0	NA	NA	0
SP140L	SP140 nuclear body protein-like	0,79	-0,34	0,08	0,43	0	-1,13	-0,18	0,49	1,00	0	-1,28	-0,35	0
RNF114	ring finger protein 114	0,79	-0,34	0,19	0,60	0	1,03	0,05	0,61	1,00	0	-1,14	-0,18	0
DHX58	DEXH (Asp-Glu-X-His) box polypeptide 58	0,79	-0,35	0,13	0,52	0	-1,00	0,00	0,99	1,00	0	0,00	0,00	0
ITPKB	inositol 1,4,5-trisphosphate 3-kinase B	0,79	-0,35	0,12	0,50	0	1,03	0,04	0,83	1,00	0	NA	NA	0
NSMAF	neutral sphingomyelinase (N-SMase) activation associated factor	0,79	-0,35	0,21	0,62	0	1,01	0,01	0,94	1,00	0	NA	NA	0
DYRK2	dual-specificity tyrosine-(Y)-phosphorylation regulated kinase 2	0,79	-0,35	0,45	0,79	0	-1,18	-0,24	0,44	1,00	0	-2,00	-0,98	0
EXTL2	exostoses (multiple)-like 2	0,79	-0,35	0,25	0,65	0	-1,43	-0,51	0,02	0,94	0	NA	NA	0
SWAP70	SWAP switching B-cell complex 70kDa subunit	0,79	-0,35	0,06	0,38	0	1,04	0,06	0,82	1,00	0	-1,04	-0,05	0
XPA	xeroderma pigmentosum, complementation group A	0,79	-0,35	0,08	0,42	0	-1,08	-0,11	0,43	1,00	0	NA	NA	0
SLC35E3	solute carrier family 35, member E3	0,79	-0,35	0,14	0,54	0	1,06	0,09	0,63	1,00	0	-1,04	-0,06	0
BRP44L	brain protein 44-like	0,79	-0,35	0,05	0,36	0	1,24	0,31	0,06	1,00	0	NA	NA	0
ZBTB3	zinc finger and BTB domain containing 3	0,79	-0,35	0,06	0,38	0	-1,42	-0,50	0,31	1,00	0	NA	NA	0
IRF1	interferon regulatory factor 1	0,79	-0,35	0,53	0,84	0	NA	NA	NA	NA	0	NA	NA	0

NA	NA	0,79	-0,35	0,16	0,56	0	1,01	0,02	0,95	1,00	0	NA	NA	0
TRHDE	thyrotropin-releasing hormone degrading enzyme	0,79	-0,35	0,39	0,76	0	-1,01	-0,02	NA	NA	0	-1,16	-0,21	0
RPA2	replication protein A2, 32kDa	0,79	-0,35	0,17	0,57	0	1,12	0,16	0,27	1,00	0	NA	NA	0
TCFL5	transcription factor-like 5 (basic helix-loop-helix)	0,79	-0,35	0,03	0,30	0	-1,13	-0,17	0,23	1,00	0	-1,27	-0,33	0
IL11RA	interleukin 11 receptor, alpha	0,79	-0,35	0,10	0,48	0	-1,01	-0,02	NA	NA	0	1,03	0,04	0
CEP57	centrosomal protein 57kDa	0,78	-0,35	0,06	0,39	0	-1,11	-0,15	0,42	1,00	0	NA	NA	0
HHLA3	HERV-H LTR-associating 3	0,78	-0,35	0,06	0,38	0	-1,28	-0,36	0,29	1,00	0	1,33	0,41	0
ZMYM3	zinc finger, MYM-type 3	0,78	-0,35	0,04	0,33	0	-1,03	-0,04	0,83	1,00	0	-1,19	-0,26	0
CCRL1	chemokine (C-C motif) receptor-like 1	0,78	-0,35	0,35	0,73	0	-1,02	-0,03	0,84	1,00	0	NA	NA	0
TMEFF1	transmembrane protein with EGF-like and two follistatin-like domains 1	0,78	-0,35	0,14	0,54	0	1,48	0,57	0,16	1,00	0	-1,16	-0,22	0
KANK2	KN motif and ankyrin repeat domains 2	0,78	-0,35	0,16	0,56	0	-1,03	-0,04	0,63	1,00	0	NA	NA	0
KLHDC2	kelch domain containing 2	0,78	-0,35	0,04	0,32	0	-1,10	-0,14	0,28	1,00	0	NA	NA	0
LETMD1	LETM1 domain containing 1	0,78	-0,35	0,05	0,36	0	-1,11	-0,16	0,23	1,00	0	NA	NA	0
ANKRD26	ankyrin repeat domain 26	0,78	-0,35	0,28	0,68	0	-1,13	-0,17	0,46	1,00	0	NA	NA	0
STAT1	signal transducer and activator of transcription 1, 91kDa	0,78	-0,35	0,13	0,52	0	-1,23	-0,30	0,14	1,00	0	-1,32	-0,40	0
PRKACB	protein kinase, cAMP-dependent, catalytic, beta	0,78	-0,35	0,19	0,60	0	-1,12	-0,17	0,56	1,00	0	-1,16	-0,22	0
SDC2	syndecan 2	0,78	-0,35	0,31	0,70	0	-1,01	-0,02	NA	NA	0	-1,20	-0,27	0
TRPC1	transient receptor potential cation channel, subfamily C, member 1	0,78	-0,35	0,23	0,64	0	-1,05	-0,08	0,77	1,00	0	-1,22	-0,29	0
ZFX	zinc finger protein, X-linked	0,78	-0,35	0,09	0,46	0	-1,01	-0,01	0,97	1,00	0	-1,12	-0,16	0
DPYD	dihydropyrimidine dehydrogenase	0,78	-0,35	0,15	0,55	0	-1,01	-0,02	0,94	1,00	0	-1,37	-0,45	0
C9orf125	chromosome 9 open reading frame 125	0,78	-0,36	0,08	0,43	0	-1,20	-0,27	0,56	1,00	0	NA	NA	0
CSPG4	chondroitin sulfate proteoglycan 4	0,78	-0,36	0,05	0,38	0	-1,02	-0,03	0,76	1,00	0	1,54	0,62	0
NR2F2	nuclear receptor subfamily 2, group F, member 2	0,78	-0,36	0,09	0,45	0	-1,32	-0,40	0,17	1,00	0	NA	NA	0
IMPACT	Impact homolog (mouse)	0,78	-0,36	0,07	0,41	0	-1,15	-0,20	0,34	1,00	0	NA	NA	0
FAN1	FANCD2/FANCI-associated nuclease 1	0,78	-0,36	0,04	0,35	0	1,02	0,02	0,91	1,00	0	NA	NA	0
PHF21A	PHD finger protein 21A	0,78	-0,36	0,06	0,39	0	1,00	0,01	0,97	1,00	0	-1,49	-0,58	0
CAPN7	calpain 7	0,78	-0,36	0,17	0,57	0	1,01	0,01	0,95	1,00	0	NA	NA	0
CAMLG	calcium modulating ligand	0,78	-0,36	0,06	0,39	0	1,01	0,01	0,95	1,00	0	NA	NA	0
C18orf10	chromosome 18 open reading frame 10	0,78	-0,36	0,17	0,57	0	-1,11	-0,15	0,17	1,00	0	-1,11	-0,14	0
LRRC40	leucine rich repeat containing 40	0,78	-0,36	0,10	0,47	0	1,02	0,03	0,79	1,00	0	NA	NA	0
ATF3	activating transcription factor 3	0,78	-0,36	0,31	0,70	0	1,47	0,55	0,27	1,00	0	1,58	0,66	0
CTBS	chitobiase, di-N-acetyl-	0,78	-0,36	0,08	0,43	0	-1,24	-0,31	0,15	1,00	0	-1,67	-0,74	0
C7orf44	chromosome 7 open reading frame 44	0,78	-0,36	0,19	0,59	0	1,22	0,29	0,34	1,00	0	-1,19	-0,26	0
PHF3	PHD finger protein 3	0,78	-0,36	0,13	0,52	0	-1,07	-0,10	0,24	1,00	0	NA	NA	0
ARHGEF10	Rho guanine nucleotide exchange factor (GEF) 10	0,78	-0,36	0,08	0,42	0	-1,05	-0,06	0,74	1,00	0	-1,15	-0,20	0
P2RX5	purinergic receptor P2X, ligand-gated ion channel, 5	0,78	-0,36	0,17	0,57	0	-1,09	-0,12	NA	NA	0	NA	NA	0
MFAP5	microfibrillar associated protein 5	0,78	-0,36	0,22	0,63	0	-1,01	-0,02	0,88	1,00	0	-1,41	-0,50	0
08-mar	membrane-associated ring finger (C3HC4) 8	0,78	-0,36	0,09	0,45	0	-1,02	-0,03	0,83	1,00	0	NA	NA	0
DCXR	dicarbonyl/L-xylulose reductase	0,78	-0,36	0,06	0,40	0	-1,13	-0,18	0,27	1,00	0	NA	NA	0
PTCH1	patched 1	0,78	-0,36	0,12	0,50	0	-1,08	-0,11	0,60	1,00	0	-1,19	-0,24	0
NUDT3	nudix (nucleoside diphosphate linked moiety X)-type motif 3	0,78	-0,36	0,09	0,45	0	-1,17	-0,22	0,20	1,00	0	NA	NA	0
HSDL2	hydroxysteroid dehydrogenase like 2	0,78	-0,36	0,25	0,65	0	-1,03	-0,05	0,80	1,00	0	-1,20	-0,27	0
H2AFV	H2A histone family, member V	0,78	-0,36	0,04	0,32	0	NA	NA	NA	NA	0	NA	NA	0
NA	NA	0,78	-0,36	0,34	0,72	0	NA	NA	NA	NA	0	NA	NA	0
DPP4	dipeptidyl-peptidase 4	0,78	-0,36	0,23	0,63	0	-1,01	-0,02	NA	NA	0	1,42	0,50	0
NQO1	NAD(P)H dehydrogenase, quinone 1	0,78	-0,36	0,20	0,60	0	-1,21	-0,28	0,32	1,00	0	NA	NA	0
CCPG1	cell cycle progression 1	0,78	-0,36	0,12	0,51	0	-1,04	-0,06	0,81	1,00	0	NA	NA	0
ANKRD10	ankyrin repeat domain 10	0,78	-0,36	0,15	0,55	0	-1,11	-0,15	0,62	1,00	0	NA	NA	0
BBS1	Bardet-Biedl syndrome 1	0,78	-0,37	0,11	0,49	0	-1,20	-0,27	0,53	1,00	0	NA	NA	0
OXSM	3-oxoacyl-ACP synthase, mitochondrial	0,78	-0,37	0,17	0,57	0	1,02	0,02	0,91	1,00	0	NA	NA	0
PSMB9	proteasome (prosome, macropain) subunit, beta type, 9 (large multifunctional peptidase 2)	0,78	-0,37	0,20	0,61	0	NA	NA	NA	NA	0	NA	NA	0
HLA-DPA1	major histocompatibility complex, class II, DP alpha 1	0,78	-0,37	0,34	0,72	0	-1,01	-0,01	0,90	1,00	0	-1,18	-0,23	0
NFYB	nuclear transcription factor Y, beta	0,78	-0,37	0,03	0,31	0	1,22	0,28	0,44	1,00	0	-1,32	-0,39	0
ANKRD28	ankyrin repeat domain 28	0,78	-0,37	0,15	0,55	0	-1,15	-0,20	0,39	1,00	0	-1,32	-0,40	0
DYNC2LI1	dynein, cytoplasmic 2, light intermediate chain 1	0,77	-0,37	0,06	0,39	0	-1,01	-0,02	0,89	1,00	0	NA	NA	0
ACE2	angiotensin I converting enzyme (peptidyl-dipeptidase A) 2	0,77	-0,37	0,57	0,86	0	-1,01	-0,01	0,96	1,00	0	NA	NA	0
DPH5	DPH5 homolog (S. cerevisiae)	0,77	-0,37	0,11	0,48	0	-1,10	-0,13	0,25	1,00	0	NA	NA	0
RBM9	RNA binding motif protein 9	0,77	-0,37	0,42	0,78	0	-1,01	-0,02	0,95	1,00	0	NA	NA	0
PBX1	pre-B-cell leukemia homeobox 1	0,77	-0,37	0,18	0,59	0	-1,03	-0,04	0,82	1,00	0	-1,32	-0,40	0
PIBF1	progesterone immunomodulatory binding factor 1	0,77	-0,37	0,01	0,21	0	-1,22	-0,29	0,40	1,00	0	NA	NA	0
RCOR3	REST corepressor 3	0,77	-0,37	0,21	0,61	0	-1,00	0,00	1,00	1,00	0	-1,35	-0,44	0
SLC29A1	solute carrier family 29 (nucleoside transporters), member 1	0,77	-0,37	0,05	0,37	0	-1,16	-0,22	0,34	1,00	0	NA	NA	0
LANCL1	LanC lantibiotic synthetase component C-like 1 (bacterial)	0,77	-0,37	0,06	0,39	0	-1,07	-0,10	0,32	1,00	0	NA	NA	0
HCP5	HLA complex P5	0,77	-0,37	0,21	0,62	0	1,00	0,00	0,98	1,00	0	0,00	0,01	0
MUC1	mucin 1, cell surface associated	0,77	-0,37	0,60	0,87	0	-1,42	-0,51	0,49	1,00	0	-1,49	-0,58	0
HLA-DOB	major histocompatibility complex, class II, DO beta	0,77	-0,37	0,28	0,68	0	-1,01	-0,01	0,97	1,00	0	0,00	0,00	0
PTBP2	polypyrimidine tract binding protein 2	0,77	-0,37	0,13	0,52	0	-1,07	-0,10	0,65	1,00	0	NA	NA	0
KIAA1109	KIAA1109	0,77	-0,38	0,02	0,27	0	-1,01	-0,02	0,93	1,00	0	NA	NA	0
MLF1	myeloid leukemia factor 1	0,77	-0,38	0,11	0,49	0	-1,03	-0,05	0,80	1,00	0	-1,23	-0,30	0
PCOLCE2	procollagen C-endopeptidase enhancer 2	0,77	-0,38	0,08	0,43	0	1,04	0,05	0,71	1,00	0	NA	NA	0
A2M	alpha-2-macroglobulin	0,77	-0,38	0,50	0,82	0	-1,01	-0,01	NA	NA	0	-1,45	-0,54	0
TBC1D19	TBC1 domain family, member 19	0,77	-0,38	0,05	0,36	0	-1,01	-0,01	NA	NA	0	-1,82	-0,87	0
HIST1H2BH	histone cluster 1, H2bh	0,77	-0,38	0,07	0,41	0	1,13	0,18	0,42	1,00	0	NA	NA	0
CBX7	chromobox homolog 7	0,77	-0,38	0,13	0,52	0	-1,00	0,00	0,99	1,00	0	0,00	0,00	0
CEP57	centrosomal protein 57kDa	0,77	-0,38	0,10	0,47	0	-1,02	-0,04	0,90	1,00	0	NA	NA	0
HMGN4	high mobility group nucleosomal binding domain 4	0,77	-0,38	0,02	0,28	0	-1,14	-0,19	0,21	1,00	0	NA	NA	0
C21orf91	chromosome 21 open reading frame 91	0,77	-0,38	0,18	0,58	0	1,05	0,08	0,72	1,00	0	0,00	0,00	0
C19orf66	chromosome 19 open reading frame 66	0,77	-0,38	0,09	0,46	0	-1,03	-0,05	0,82	1,00	0	-1,23	-0,30	0
ARL4C	ADP-ribosylation factor-like 4C	0,77	-0,38	0,13	0,52	0	1,18	0,24	0,15	1,00	0	NA	NA	0
ZNF512B	zinc finger protein 512B	0,77	-0,38	0,20	0,61	0	1,04	0,06	0,71	1,00	0	1,11	0,15	0
NA	NA	0,77	-0,38	0,05	0,36	0	NA	NA	NA	NA	0	NA	NA	0
MAF	v-maf musculoaponeurotic fibrosarcoma oncogene homolog (avian)	0,77	-0,38	0,06	0,39	0	-1,36	-0,44	0,06	1,00	0	NA	NA	0
NA	NA	0,77	-0,38	0,11	0,50	0	NA	NA	NA	NA	0	NA	NA	0
IL15RA	interleukin 15 receptor, alpha	0,77	-0,38	0,23	0,63	0	1,00	0,01	0,95	1,00	0	-1,06	-0,09	0
ATF2	activating transcription factor 2	0,77	-0,38	0,10	0,47	0	-1,04	-0,05	0,78	1,00	0	NA	NA	0
NA	NA	0,77	-0,38	0,14	0,54	0	NA	NA	NA	NA	0	NA	NA	0
NEK1	NIMA (never in mitosis gene a)-related kinase 1	0,77	-0,38	0,09	0,45	0	1,03	0,05	0,79	1,00	0	NA	NA	0
C5orf13	chromosome 5 open reading frame 13	0,77	-0,39	0,15	0,55	0	-1,12	-0,16	0,74	1,00	0	-1,19	-0,26	0
PPP2R5A	protein phosphatase 2, regulatory subunit B', alpha	0,77	-0,39	0,01	0,18	0	1,02	0,03	0,74	1,00	0	-1,09	-0,11	0
GSTK1	glutathione S-transferase kappa 1	0,77	-0,39	0,04	0,34	0	-1,17	-0,23	0,06	1,00	0	-1,16	-0,22	0
ZNF226	zinc finger protein 226	0,77	-0,39	0,18	0,59	0	-1,07	-0,09	0,66	1,00	0	NA	NA	0
TDRD3	tudor domain containing 3	0,77	-0,39	0,07	0,40	0	1,06	0,08	0,56	1,00	0	NA	NA	0
FXYD3	FXYD domain containing ion transport regulator 3	0,76	-0,39	0,14	0,54	0	1,08	0,11	0,53	1,00	0	NA	NA	0
PPOX	protoporphyrinogen oxidase	0,76	-0,39	0,09	0,45	0	-1,14	-0,19	0,37	1,00	0	NA	NA	0
TACC2	transforming, acidic coiled-coil containing protein 2	0,76	-0,39	0,07	0,41	0	-1,16	-0,21	0,23	1,00	0	-1,22	-0,28	0
TTC26	tetratricopeptide repeat domain 26	0,76	-0,39	0,06	0,40	0	-1,17	-0,22	0,24	1,00	0	NA	NA	0
FAM178A	family with sequence similarity 178, member A	0,76	-0,39	0,03	0,31	0	-1,14	-0,19	0,26	1,00	0	NA	NA	0
C2orf3	chromosome 2 open reading frame 3	0,76	-0,39	0,16	0,56	0	-1,08	-0,12	0,53	1,00	0	-1,41	-0,49	0
CYB5A	cytochrome b5 type A (microsomal)	0,76	-0,39	0,11	0,50	0	-1,03	-0,04	0,80	1,00	0	-1,09	-0,13	0
IRF1	interferon regulatory factor 1	0,76	-0,39	0,48	0,81	0	-1,01	-0,01	0,94	1,00	0	-1,33	-0,41	0
ITGB5	integrin, beta 5	0,76	-0,39	0,06	0,40	0	-1,03	-0,04	0,78	1,00	0	-1,04	-0,06	0
ZNF43	zinc finger protein 43	0,76	-0,39	0,20	0,61	0	-1,25	-0,33	0,15	1,00	0	NA	NA	0
MTF2	metal response element binding transcription factor 2	0,76	-0,39	0,02	0,28	0	-1,24	-0,31	0,17	1,00	0	NA	NA	0
GCSH	glycine cleavage system protein H (aminomethyl carrier)	0,76	-0,39	0,09	0,46	0	-1,01	-0,02	0,88	1,00	0	-1,11	-0,15	0
PHYH	phytanoyl-CoA 2-hydroxylase	0,76	-0,39	0,06	0,38	0	-1,04	-0,05	0,63	1,00	0	NA	NA	0
PDGFRA	platelet-derived growth factor receptor, alpha polypeptide	0,76	-0,39	0,25	0,65	0	-1,01	-0,01	0,95	1,00	0	NA	NA	0
CSNK2A2	casein kinase 2, alpha prime polypeptide	0,76	-0,39	0,16	0,56	0	-1,20	-0,27	0,05	1,00	0	NA	NA	0
GCSH	glycine cleavage system protein H (aminomethyl carrier)	0,76	-0,39	0,11	0,48	0	1,00	0,01	0,96	1,00	0	-1,10	-0,13	0
MTCP1NB	mature T-cell proliferation 1 neighbor	0,76	-0,39	0,05	0,37	0	-1,18	-0,23	0,20	1,00	0	NA	NA	0
MAGED2	melanoma antigen family D, 2	0,76	-0,39	0,07	0,41	0	-1,06	-0,08	0,57	1,00	0	NA	NA	0
NA	NA	0,76	-0,39	0,28	0,68	0	-1,27	-0,34	0,44	1,00	0	NA	NA	0
CHEK1	CHK1 checkpoint homolog (S. pombe)	0,76	-0,39	0,22	0,63	0	-1,03	-0,05	0,81	1,00	0	NA	NA	0
KIF3A	kinesin family member 3A	0,76	-0,39	0,10	0,47	0	NA	NA	NA	NA	0	NA	NA	0
CD58	CD58 molecule	0,76	-0,39	0,19	0,59	0	-1,01	-0,02	NA	NA	0	NA	NA	0
AOX1	aldehyde oxidase 1	0,76	-0,39	0,36	0,73	0	-1,06	-0,09	0,72	1,00	0	NA	NA	0
SP100	SP100 nuclear antigen	0,76	-0,39	0,26	0,66	0	-1,22	-0,28	0,20	1,00	0	-1,25	-0,31	0
PLCB4	phospholipase C, beta 4	0,76	-0,39	0,24	0,65	0	-1,00	-0,01	NA	NA	0	-1,08	-0,11	0
C1S	complement component 1, s subcomponent	0,76	-0,40	0,13	0,53	0	-1,01	-0,01	0,94	1,00	0	-1,12	-0,17	0
DDAH2	dimethylarginine dimethylaminohydrolase 2	0,76	-0,40	0,07	0,42	0	-1,01	-0,02	0,96	1,00	0	-1,25	-0,32	0
UBA7	ubiquitin-like modifier activating enzyme 7	0,76	-0,40	0,28	0,68	0	-1,01	-0,01	0,97	1,00	0	-1,16	-0,21	0
TMEM164	transmembrane protein 164	0,76	-0,40	0,13	0,53	0	-1,09	-0,12	0,37	1,00	0	NA	NA	0
EML4	echinoderm microtubule associated protein like 4	0,76	-0,40	0,15	0,54	0	-1,60	-0,68	0,23	1,00	0	-1,33	-0,41	0
NA	NA	0,76	-0,40	0,23	0,63	0	-1,03	-0,04	0,85	1,00	0	NA	NA	0
TMEM47	transmembrane protein 47	0,76	-0,40	0,39	0,76	0	-1,01	-0,01	NA	NA	0	-1,16	-0,23	0
EVL	Enah/Vasp-like	0,76	-0,40	0,02	0,27	0	-1,03	-0,04	0,86	1,00	0	NA	NA	0
KDELR3	KDEL (Lys-Asp-Glu-Leu) endoplasmic reticulum protein retention receptor 3	0,76	-0,40	0,06	0,38	0	-1,01	-0,01	NA	NA	0	-1,47	-0,56	0
DUSP10	dual specificity phosphatase 10	0,76	-0,40	0,32	0,71	0	-1,09	-0,13	0,79	1,00	0	-1,41	-0,50	0
LPCAT4	lysophosphatidylcholine acyltransferase 4	0,76	-0,40	0,67	0,90	0	-1,09	-0,13	0,50	1,00	0	NA	NA	0
APPL1	adaptor protein, phosphotyrosine interaction, PH domain and leucine zipper containing 1	0,76	-0,40	0,03	0,31	0	1,05	0,08	0,65	1,00	0	-1,54	-0,62	0
TRADD	TNFRSF1A-associated via death domain	0,76	-0,40	0,05	0,36	0	-1,13	-0,18	0,13	1,00	0	NA	NA	0
ZNF623	zinc finger protein 623	0,76	-0,40	0,05	0,37	0	-1,18	-0,24	0,51	1,00	0	-1,28	-0,35	0
DCUN1D4	DCN1, defective in cullin neddylation 1, domain containing 4 (S. cerevisiae)	0,76	-0,40	0,04	0,33	0	-1,04	-0,06	0,75	1,00	0	NA	NA	0
APOL2	apolipoprotein L, 2	0,76	-0,40	0,14	0,53	0	-1,03	-0,04	0,69	1,00	0	-1,06	-0,09	0
FPGT	fucose-1-phosphate guanylyltransferase	0,76	-0,40	0,02	0,23	0	1,03	0,04	0,85	1,00	0	NA	NA	0
MTMR11	myotubularin related protein 11	0,76	-0,40	0,10	0,46	0	1,01	0,01	0,95	1,00	0	-1,18	-0,23	0
PSIP1	PC4 and SFRS1 interacting protein 1	0,76	-0,40	0,06	0,39	0	-1,15	-0,21	0,31	1,00	0	NA	NA	0
SAP30L	SAP30-like	0,76	-0,41	0,05	0,35	0	-1,13	-0,17	0,29	1,00	0	1,14	0,19	0

ALDH1A1	aldehyde dehydrogenase 1 family, member A1	0,75	-0,41	0,23	0,64	0	-1,01	-0,01	NA	NA	0	-1,30	-0,37	0
DERA	deoxyribose-phosphate aldolase (putative)	0,75	-0,41	0,12	0,51	0	-1,05	-0,08	0,47	1,00	0	NA	NA	0
PHF3	PHD finger protein 3	0,75	-0,41	0,03	0,28	0	-1,14	-0,19	0,18	1,00	0	NA	NA	0
NENF	neuron derived neurotrophic factor	0,75	-0,41	0,02	0,26	0	-1,11	-0,15	0,37	1,00	0	NA	NA	0
KIAA1598	KIAA1598	0,75	-0,41	0,16	0,56	0	-1,21	-0,28	0,09	1,00	0	-1,37	-0,45	0
NA	NA	0,75	-0,41	0,70	0,91	0	-1,81	-0,85	0,00	0,43	0	NA	NA	0
ZMAT3	zinc finger, matrin type 3	0,75	-0,41	0,07	0,41	0	-1,32	-0,40	0,06	1,00	0	-1,19	-0,26	0
LSM2	LSM2 homolog, U6 small nuclear RNA associated (S. cerevisiae)	0,75	-0,41	0,19	0,60	0	-1,13	-0,18	0,42	1,00	0	NA	NA	0
BBS10	Bardet-Biedl syndrome 10	0,75	-0,41	0,15	0,55	0	-1,01	-0,01	0,94	1,00	0	-1,10	-0,14	0
CABC1	chaperone, ABC1 activity of bc1 complex homolog (S. pombe)	0,75	-0,41	0,02	0,25	0	-1,21	-0,27	0,21	1,00	0	NA	NA	0
MCAM	melanoma cell adhesion molecule	0,75	-0,41	0,04	0,34	0	-1,10	-0,14	0,29	1,00	0	NA	NA	0
ZC3H6	zinc finger CCCH-type containing 6	0,75	-0,41	0,03	0,31	0	NA	NA	NA	NA	0	NA	NA	0
NA	NA	0,75	-0,41	0,08	0,43	0	NA	NA	NA	NA	0	NA	NA	0
PPA1	pyrophosphatase (inorganic) 1	0,75	-0,41	0,06	0,38	0	-1,02	-0,03	0,72	1,00	0	-1,08	-0,10	0
CLDN23	claudin 23	0,75	-0,41	0,02	0,24	0	NA	NA	NA	NA	0	NA	NA	0
C6orf130	chromosome 6 open reading frame 130	0,75	-0,41	0,05	0,36	0	-1,02	-0,03	0,87	1,00	0	NA	NA	0
RP2	retinitis pigmentosa 2 (X-linked recessive)	0,75	-0,41	0,31	0,70	0	1,07	0,10	0,45	1,00	0	NA	NA	0
DYNLT1	dynein, light chain, Tctex-type 1	0,75	-0,42	0,03	0,28	0	1,09	0,13	0,39	1,00	0	NA	NA	0
PDCD4	programmed cell death 4 (neoplastic transformation inhibitor)	0,75	-0,42	0,07	0,41	0	-1,52	-0,60	0,00	0,26	0	NA	NA	0
FZD7	frizzled homolog 7 (Drosophila)	0,75	-0,42	0,10	0,48	0	-1,03	-0,05	0,89	1,00	0	-1,16	-0,21	0
ERAP2	endoplasmic reticulum aminopeptidase 2	0,75	-0,42	0,07	0,41	0	NA	NA	NA	NA	0	NA	NA	0
PBX1	pre-B-cell leukemia homeobox 1	0,75	-0,42	0,11	0,48	0	-1,26	-0,34	0,03	0,97	0	-1,28	-0,36	0
ORAI3	ORAI calcium release-activated calcium modulator 3	0,75	-0,42	0,03	0,31	0	-1,29	-0,37	0,36	1,00	0	NA	NA	0
APOL6	apolipoprotein L, 6	0,75	-0,42	0,36	0,74	0	NA	NA	NA	NA	0	NA	NA	0
SPAST	spastin	0,75	-0,42	0,14	0,53	0	-1,00	0,00	0,99	1,00	0	NA	NA	0
SNRPN	small nuclear ribonucleoprotein polypeptide N	0,75	-0,42	0,06	0,38	0	NA	NA	NA	NA	0	NA	NA	0
PHF16	PHD finger protein 16	0,75	-0,42	0,07	0,40	0	1,04	0,06	0,80	1,00	0	-1,23	-0,31	0
MKRN1	makorin ring finger protein 1	0,75	-0,42	0,04	0,34	0	-1,06	-0,09	0,62	1,00	0	-1,15	-0,20	0
THAP10	THAP domain containing 10	0,75	-0,42	0,08	0,44	0	1,15	0,20	0,64	1,00	0	NA	NA	0
ENPP2	ectonucleotide pyrophosphatase/phosphodiesterase 2	0,75	-0,42	0,18	0,59	0	-1,00	-0,01	NA	NA	0	1,02	0,02	0
TRIM21	tripartite motif-containing 21	0,75	-0,42	0,19	0,60	0	-1,15	-0,20	0,45	1,00	0	-1,37	-0,45	0
C19orf66	chromosome 19 open reading frame 66	0,75	-0,42	0,12	0,51	0	-1,07	-0,10	0,56	1,00	0	-1,22	-0,29	0
MORC4	MORC family CW-type zinc finger 4	0,75	-0,42	0,07	0,42	0	-1,07	-0,09	0,58	1,00	0	-1,19	-0,26	0
HIST1H2BE	histone cluster 1, H2be	0,75	-0,42	0,06	0,40	0	1,26	0,33	0,17	1,00	0	NA	NA	0
C4orf43	chromosome 4 open reading frame 43	0,75	-0,42	0,19	0,60	0	-1,01	-0,01	0,97	1,00	0	NA	NA	0
AKR7A2	aldo-keto reductase family 7, member A2 (aflatoxin aldehyde reductase)	0,75	-0,42	0,02	0,27	0	-1,01	-0,01	0,92	1,00	0	NA	NA	0
NA	NA	0,75	-0,42	0,31	0,70	0	-1,20	-0,26	0,66	1,00	0	NA	NA	0
PRKCA	protein kinase C, alpha	0,75	-0,42	0,11	0,49	0	-1,04	-0,06	NA	NA	0	-1,25	-0,31	0
ITGB3BP	integrin beta 3 binding protein (beta3-endonexin)	0,75	-0,42	0,24	0,65	0	-1,01	-0,01	0,96	1,00	0	NA	NA	0
TRIM14	tripartite motif-containing 14	0,75	-0,42	0,42	0,78	0	1,23	0,30	0,34	1,00	0	-1,49	-0,57	0
RFX7	regulatory factor X, 7	0,75	-0,42	0,17	0,57	0	-1,26	-0,33	0,47	1,00	0	-1,28	-0,37	0
ASF1A	ASF1 anti-silencing function 1 homolog A (S. cerevisiae)	0,74	-0,43	0,08	0,44	0	1,03	0,04	0,81	1,00	0	NA	NA	0
HOXC10	homeobox C10	0,74	-0,43	0,07	0,42	0	-1,17	-0,22	0,24	1,00	0	NA	NA	0
NA	NA	0,74	-0,43	0,07	0,40	0	NA	NA	NA	NA	0	NA	NA	0
IQCB1	IQ motif containing B1	0,74	-0,43	0,18	0,59	0	1,05	0,07	0,71	1,00	0	NA	NA	0
ARL4D	ADP-ribosylation factor-like 4D	0,74	-0,43	0,07	0,42	0	1,05	0,07	0,72	1,00	0	NA	NA	0
TCEAL1	transcription elongation factor A (SII)-like 1	0,74	-0,43	0,05	0,35	0	-1,06	-0,08	0,63	1,00	0	-1,19	-0,24	0
TFB1M	transcription factor B1, mitochondrial	0,74	-0,43	0,05	0,35	0	-1,12	-0,16	0,56	1,00	0	NA	NA	0
PLAGL1	pleiomorphic adenoma gene-like 1	0,74	-0,43	0,10	0,48	0	1,18	0,24	0,15	1,00	0	NA	NA	0
FAS	Fas (TNF receptor superfamily, member 6)	0,74	-0,43	0,01	0,22	0	-1,01	-0,01	0,90	1,00	0	1,28	0,35	0
ATG2B	ATG2 autophagy related 2 homolog B (S. cerevisiae)	0,74	-0,43	0,07	0,41	0	-1,28	-0,36	0,05	1,00	0	-1,35	-0,44	0
ZNF184	zinc finger protein 184	0,74	-0,43	0,14	0,53	0	-1,16	-0,21	0,47	1,00	0	NA	NA	0
HOXA10	homeobox A10	0,74	-0,43	0,06	0,38	0	1,03	0,04	0,88	1,00	0	NA	NA	0
ATG14	ATG14 autophagy related 14 homolog (S. cerevisiae)	0,74	-0,43	0,01	0,15	0	-1,00	0,00	0,99	1,00	0	NA	NA	0
C1D	C1D nuclear receptor corepressor	0,74	-0,43	0,28	0,68	0	-1,16	-0,22	0,11	1,00	0	NA	NA	0
FBLN1	fibulin 1	0,74	-0,43	0,12	0,51	0	-1,18	-0,24	0,47	1,00	0	NA	NA	0
TAPBPL	TAP binding protein-like	0,74	-0,43	0,15	0,55	0	1,05	0,08	0,75	1,00	0	-1,27	-0,34	0
PLAGL1	pleiomorphic adenoma gene-like 1	0,74	-0,43	0,01	0,20	0	-1,06	-0,08	0,60	1,00	0	NA	NA	0
BTN3A1	butyrophilin, subfamily 3, member A1	0,74	-0,43	0,09	0,44	0	-1,30	-0,38	0,28	1,00	0	-1,23	-0,31	0
DCK	deoxycytidine kinase	0,74	-0,43	0,16	0,56	0	-1,05	-0,07	0,73	1,00	0	NA	NA	0
CDH2	cadherin 2, type 1, N-cadherin (neuronal)	0,74	-0,43	0,33	0,72	0	-1,01	-0,01	NA	NA	0	-1,08	-0,11	0
ZCCHC11	zinc finger, CCHC domain containing 11	0,74	-0,43	0,10	0,48	0	-1,22	-0,28	0,31	1,00	0	-1,43	-0,50	0
BTN3A2	butyrophilin, subfamily 3, member A2	0,74	-0,43	0,06	0,38	0	-1,22	-0,28	0,29	1,00	0	-1,11	-0,16	0
BBS4	Bardet-Biedl syndrome 4	0,74	-0,44	0,09	0,45	0	-1,31	-0,39	0,16	1,00	0	NA	NA	0
ORC5L	origin recognition complex, subunit 5-like (yeast)	0,74	-0,44	0,03	0,28	0	1,09	0,13	0,39	1,00	0	NA	NA	0
NA	NA	0,74	-0,44	0,20	0,60	0	NA	NA	NA	NA	0	NA	NA	0
CENPM	centromere protein M	0,74	-0,44	0,14	0,54	0	-1,04	-0,05	0,76	1,00	0	NA	NA	0
SRPX	sushi-repeat-containing protein, X-linked	0,74	-0,44	0,06	0,38	0	1,07	0,10	0,67	1,00	0	-1,23	-0,30	0
TRAFD1	TRAF-type zinc finger domain containing 1	0,74	-0,44	0,14	0,53	0	-1,03	-0,04	0,79	1,00	0	0,00	-0,01	0
TTC33	tetratricopeptide repeat domain 33	0,74	-0,44	0,08	0,42	0	-1,13	-0,18	0,30	1,00	0	-1,43	-0,52	0
GLMN	glomulin, FKBP associated protein	0,74	-0,44	0,08	0,43	0	NA	NA	NA	NA	0	NA	NA	0
DAAM2	dishevelled associated activator of morphogenesis 2	0,74	-0,44	0,33	0,72	0	-1,01	-0,02	NA	NA	0	-1,09	-0,12	0
RAD51C	RAD51 homolog C (S. cerevisiae)	0,74	-0,44	0,03	0,30	0	1,04	0,06	0,87	1,00	0	NA	NA	0
DDHD2	DDHD domain containing 2	0,74	-0,44	0,04	0,33	0	-1,04	-0,06	0,63	1,00	0	NA	NA	0
CRIPT	cysteine-rich PDZ-binding protein	0,74	-0,44	0,25	0,66	0	-1,02	-0,02	0,93	1,00	0	-1,28	-0,36	0
SERPINE1	serpin peptidase inhibitor, clade E (nexin, plasminogen activator inhibitor type 1), member 1	0,74	-0,44	0,32	0,71	0	-1,01	-0,02	0,91	1,00	0	NA	NA	0
GABARAPL1	GABA(A) receptor-associated protein like 1	0,74	-0,44	0,04	0,34	0	-1,16	-0,21	0,47	1,00	0	-1,10	-0,13	0
NNT	nicotinamide nucleotide transhydrogenase	0,74	-0,44	0,01	0,21	0	-1,21	-0,27	0,16	1,00	0	NA	NA	0
VLDLR	very low density lipoprotein receptor	0,74	-0,44	0,09	0,45	0	-1,09	-0,12	0,27	1,00	0	-1,23	-0,31	0
NA	NA	0,74	-0,44	0,25	0,65	0	NA	NA	NA	NA	0	NA	NA	0
KIAA0564	KIAA0564	0,74	-0,44	0,01	0,20	0	-1,03	-0,04	0,75	1,00	0	NA	NA	0
BAMBI	BMP and activin membrane-bound inhibitor homolog (Xenopus laevis)	0,74	-0,44	0,02	0,25	0	-1,15	-0,21	0,02	0,94	0	-1,25	-0,32	0
METTL7A	methyltransferase like 7A	0,74	-0,44	0,20	0,60	0	-1,90	-0,93	0,20	1,00	0	-1,47	-0,57	0
ABHD10	abhydrolase domain containing 10	0,74	-0,44	0,02	0,25	0	-1,03	-0,04	0,84	1,00	0	-1,32	-0,39	0
USP9Y	ubiquitin specific peptidase 9, Y-linked	0,74	-0,44	0,04	0,32	0	NA	NA	NA	NA	0	NA	NA	0
NA	NA	0,74	-0,44	0,10	0,47	0	-1,23	-0,30	0,10	1,00	0	NA	NA	0
MPHOSPH9	M-phase phosphoprotein 9	0,74	-0,44	0,07	0,41	0	-1,18	-0,24	0,40	1,00	0	NA	NA	0
TXNIP	thioredoxin interacting protein	0,74	-0,44	0,72	0,91	0	-1,85	-0,89	0,19	1,00	0	-1,67	-0,74	0
IPW	imprinted in Prader-Willi syndrome (non-protein coding)	0,73	-0,44	0,10	0,47	0	-1,18	-0,24	0,28	1,00	0	-1,39	-0,48	0
C18orf10	chromosome 18 open reading frame 10	0,73	-0,44	0,04	0,34	0	-1,18	-0,23	0,09	1,00	0	-1,16	-0,21	0
CELF2	CUGBP, Elav-like family member 2	0,73	-0,45	0,13	0,53	0	-1,22	-0,28	0,24	1,00	0	NA	NA	0
NA	NA	0,73	-0,45	0,06	0,39	0	-1,00	-0,01	0,98	1,00	0	-1,27	-0,33	0
NA	NA	0,73	-0,45	0,01	0,21	0	-1,06	-0,08	0,74	1,00	0	-1,11	-0,15	0
DEGS1	degenerative spermatocyte homolog 1, lipid desaturase (Drosophila)	0,73	-0,45	0,02	0,23	0	-1,17	-0,23	0,05	1,00	0	NA	NA	0
EFNB2	ephrin-B2	0,73	-0,45	0,40	0,76	0	1,02	0,02	0,91	1,00	0	-1,33	-0,41	0
CPT1A	carnitine palmitoyltransferase 1A (liver)	0,73	-0,45	0,03	0,29	0	-1,22	-0,29	0,49	1,00	0	NA	NA	0
CBR4	carbonyl reductase 4	0,73	-0,45	0,01	0,15	0	1,08	0,12	0,57	1,00	0	-1,33	-0,42	0
LASS6	LAG1 homolog, ceramide synthase 6	0,73	-0,45	0,05	0,36	0	-1,08	-0,11	0,62	1,00	0	NA	NA	0
VCAN	versican	0,73	-0,45	0,26	0,66	0	-1,01	-0,02	NA	NA	0	-1,43	-0,51	0
ITGAE	integrin, alpha E (antigen CD103, human mucosal lymphocyte antigen 1; alpha polypeptide)	0,73	-0,45	0,15	0,55	0	1,16	0,22	0,16	1,00	0	1,01	0,02	0
DDAH2	dimethylarginine dimethylaminohydrolase 2	0,73	-0,45	0,03	0,29	0	-1,04	-0,06	0,76	1,00	0	-1,09	-0,12	0
EFS	embryonal Fyn-associated substrate	0,73	-0,45	0,03	0,30	0	-1,08	-0,11	0,57	1,00	0	NA	NA	0
MLF1IP	MLF1 interacting protein	0,73	-0,45	0,14	0,53	0	-1,01	-0,01	0,96	1,00	0	NA	NA	0
FAM50B	family with sequence similarity 50, member B	0,73	-0,45	0,05	0,36	0	-1,05	-0,07	0,75	1,00	0	NA	NA	0
CLU	clusterin	0,73	-0,45	0,11	0,50	0	-1,27	-0,34	0,23	1,00	0	NA	NA	0
SATB1	SATB homeobox 1	0,73	-0,45	0,12	0,51	0	-1,27	-0,34	0,24	1,00	0	NA	NA	0
ENPP2	ectonucleotide pyrophosphatase/phosphodiesterase 2	0,73	-0,45	0,14	0,54	0	-1,01	-0,01	NA	NA	0	1,03	0,04	0
CDC14B	CDC14 cell division cycle 14 homolog B (S. cerevisiae)	0,73	-0,45	0,06	0,38	0	1,00	0,01	NA	NA	0	-1,23	-0,30	0
HOXA9	homeobox A9	0,73	-0,45	0,02	0,27	0	1,01	0,01	0,96	1,00	0	NA	NA	0
NUPL2	nucleoporin like 2	0,73	-0,45	0,02	0,28	0	1,06	0,09	0,67	1,00	0	-1,20	-0,27	0
FUBP1	far upstream element (FUSE) binding protein 1	0,73	-0,46	0,13	0,52	0	-1,01	-0,01	0,98	1,00	0	-1,28	-0,37	0
MCAM	melanoma cell adhesion molecule	0,73	-0,46	0,19	0,60	0	1,23	0,30	0,35	1,00	0	-1,02	-0,02	0
CLU	clusterin	0,73	-0,46	0,15	0,55	0	-1,14	-0,18	0,51	1,00	0	NA	NA	0
NUFIP1	nuclear fragile X mental retardation protein interacting protein 1	0,73	-0,46	0,12	0,51	0	-1,03	-0,04	0,85	1,00	0	NA	NA	0
NA	NA	0,73	-0,46	0,03	0,30	0	NA	NA	NA	NA	0	NA	NA	0
COX11	COX11 cytochrome c oxidase assembly homolog (yeast)	0,73	-0,46	0,04	0,33	0	-1,00	0,00	0,99	1,00	0	NA	NA	0
GBP1	guanylate binding protein 1, interferon-inducible, 67kDa	0,73	-0,46	0,14	0,54	0	1,13	0,18	0,34	1,00	0	-1,39	-0,47	0
RAB8B	RAB8B, member RAS oncogene family	0,73	-0,46	0,03	0,29	0	-1,11	-0,15	0,33	1,00	0	-1,19	-0,25	0
C1RL	complement component 1, r subcomponent-like	0,73	-0,46	0,06	0,39	0	-1,05	-0,07	0,69	1,00	0	-1,23	-0,31	0
NR2F2	nuclear receptor subfamily 2, group F, member 2	0,73	-0,46	0,05	0,37	0	-1,17	-0,23	0,19	1,00	0	NA	NA	0
ATP8B4	ATPase, class I, type 8B, member 4	0,73	-0,46	0,25	0,66	0	-1,01	-0,01	NA	NA	0	1,01	0,01	0
SDC2	syndecan 2	0,73	-0,46	0,52	0,83	0	1,00	0,00	1,00	1,00	0	-1,09	-0,13	0
JAK2	Janus kinase 2	0,73	-0,46	0,22	0,62	0	-1,02	-0,04	0,86	1,00	0	-1,03	-0,05	0
KAT2A	K(lysine) acetyltransferase 2A	0,73	-0,46	0,05	0,35	0	-1,09	-0,12	0,45	1,00	0	NA	NA	0
HCFC1R1	host cell factor C1 regulator 1 (XPO1 dependent)	0,73	-0,46	0,04	0,34	0	1,02	0,04	0,82	1,00	0	1,14	0,19	0
NEDD9	neural precursor cell expressed, developmentally down-regulated 9	0,73	-0,46	0,20	0,60	0	1,17	0,23	0,44	1,00	0	NA	NA	0
IRX5	iroquois homeobox 5	0,73	-0,46	0,06	0,39	0	1,20	0,26	0,55	1,00	0	NA	NA	0
ARMCX5	armadillo repeat containing, X-linked 5	0,73	-0,46	0,01	0,15	0	-1,04	-0,05	0,60	1,00	0	-1,27	-0,34	0
MTCP1NB	mature T-cell proliferation 1 neighbor	0,72	-0,46	0,04	0,34	0	-1,18	-0,24	0,06	1,00	0	NA	NA	0
SLC37A4	solute carrier family 37 (glucose-6-phosphate transporter), member 4	0,72	-0,47	0,01	0,16	0	1,05	0,07	0,76	1,00	0	NA	NA	0
SFMBT2	Scm-like with four mbt domains 2	0,72	-0,47	0,10	0,48	0	NA	NA	NA	NA	0	NA	NA	0
SPSB1	splA/ryanodine receptor domain and SOCS box containing 1	0,72	-0,47	0,02	0,26	0	1,05	0,07	0,73	1,00	0	NA	NA	0
LY96	lymphocyte antigen 96	0,72	-0,47	0,31	0,70	0	1,01	0,02	0,97	1,00	0	NA	NA	0
USPL1	ubiquitin specific peptidase like 1	0,72	-0,47	0,08	0,43	0	NA	NA	NA	NA	0	NA	NA	0
CDC14B	CDC14 cell division cycle 14 homolog B (S. cerevisiae)	0,72	-0,47	0,02	0,24	0	-1,01	-0,01	NA	NA	0	-1,02	-0,02	0
DCAF17	DDB1 and CUL4 associated factor 17	0,72	-0,47	0,07	0,40	0	-1,32	-0,40	0,21	1,00	0	-1,67	-0,74	0
ST6GALNAC2	ST6 (alpha-N-acetyl-neuraminyl-2,3-beta-galactosyl-1,3)-N-acetylgalactosaminide alpha-2,6-sialyltransferase 2	0,72	-0,47	0,12	0,51	0	-1,39	-0,47	0,00	0,43	0	NA	NA	0

PEX12	peroxisomal biogenesis factor 12	0,72	-0,47	0,02	0,23	0	1,01	0,02	0,93	1,00	0	-1,18	-0,24	0
SLC35D2	solute carrier family 35, member D2	0,72	-0,47	0,01	0,21	0	-1,11	-0,15	0,20	1,00	0	NA	NA	0
HNRNPA1	heterogeneous nuclear ribonucleoprotein A1	0,72	-0,47	0,18	0,58	0	1,10	0,14	0,57	1,00	0	-1,19	-0,25	0
WRB	tryptophan rich basic protein	0,72	-0,48	0,07	0,42	0	-1,01	-0,02	0,91	1,00	0	-1,15	-0,20	0
HIBCH	3-hydroxyisobutyryl-CoA hydrolase	0,72	-0,48	0,05	0,37	0	-1,06	-0,08	0,53	1,00	0	-1,35	-0,44	0
POLI	polymerase (DNA directed) iota	0,72	-0,48	0,10	0,46	0	-1,51	-0,59	0,03	1,00	0	NA	NA	0
DPH5	DPH5 homolog (S. cerevisiae)	0,72	-0,48	0,07	0,42	0	-1,25	-0,32	0,23	1,00	0	NA	NA	0
BHLHE41	basic helix-loop-helix family, member e41	0,72	-0,48	0,22	0,62	0	-1,00	0,00	NA	NA	0	-1,22	-0,28	0
SOCS2	suppressor of cytokine signaling 2	0,72	-0,48	0,09	0,44	0	-1,19	-0,25	0,36	1,00	0	NA	NA	0
NA	NA	0,72	-0,48	0,16	0,56	0	1,18	0,24	0,24	1,00	0	1,08	0,11	0
CYB5A	cytochrome b5 type A (microsomal)	0,72	-0,48	0,06	0,38	0	1,08	0,11	0,44	1,00	0	-1,20	-0,26	0
PRKACB	protein kinase, cAMP-dependent, catalytic, beta	0,72	-0,48	0,01	0,19	0	-1,08	-0,11	0,51	1,00	0	NA	NA	0
HSD17B11	hydroxysteroid (17-beta) dehydrogenase 11	0,72	-0,48	0,07	0,42	0	-1,17	-0,22	0,32	1,00	0	NA	NA	0
PCYOX1L	prenylcysteine oxidase 1 like	0,71	-0,48	0,02	0,24	0	1,03	0,04	0,90	1,00	0	-1,04	-0,06	0
GABARAPL1	GABA(A) receptor-associated protein like 1	0,71	-0,48	0,01	0,20	0	-1,06	-0,08	0,71	1,00	0	-1,09	-0,11	0
RAB20	RAB20, member RAS oncogene family	0,71	-0,49	0,46	0,80	0	1,05	0,07	0,67	1,00	0	-1,01	-0,01	0
CLEC2B	C-type lectin domain family 2, member B	0,71	-0,49	0,16	0,56	0	1,55	0,63	0,20	1,00	0	-1,32	-0,39	0
CNTN1	contactin 1	0,71	-0,49	0,28	0,68	0	-1,40	-0,49	0,36	1,00	0	NA	NA	0
SOCS2	suppressor of cytokine signaling 2	0,71	-0,49	0,37	0,75	0	-1,03	-0,05	0,90	1,00	0	NA	NA	0
C14orf132	chromosome 14 open reading frame 132	0,71	-0,49	0,11	0,49	0	-1,01	-0,01	NA	NA	0	-1,11	-0,16	0
HOXA9	homeobox A9	0,71	-0,49	0,01	0,16	0	-1,06	-0,08	0,44	1,00	0	NA	NA	0
GBP3	guanylate binding protein 3	0,71	-0,49	0,03	0,28	0	NA	NA	NA	NA	0	NA	NA	0
PLSCR4	phospholipid scramblase 4	0,71	-0,49	0,15	0,55	0	-1,02	-0,03	0,94	1,00	0	-1,49	-0,58	0
NISCH	nischarin	0,71	-0,49	0,01	0,21	0	-1,14	-0,19	0,12	1,00	0	NA	NA	0
RFX5	regulatory factor X, 5 (influences HLA class II expression)	0,71	-0,49	0,13	0,53	0	-1,19	-0,25	0,16	1,00	0	1,08	0,12	0
TMEM62	transmembrane protein 62	0,71	-0,49	0,14	0,53	0	1,27	0,35	0,12	1,00	0	1,11	0,16	0
FBXL3	F-box and leucine-rich repeat protein 3	0,71	-0,49	0,01	0,18	0	NA	NA	NA	NA	0	NA	NA	0
TMC6	transmembrane channel-like 6	0,71	-0,49	0,17	0,57	0	-1,10	-0,14	0,48	1,00	0	NA	NA	0
SP100	SP100 nuclear antigen	0,71	-0,49	0,04	0,34	0	-1,14	-0,19	0,32	1,00	0	-1,33	-0,42	0
SP100	SP100 nuclear antigen	0,71	-0,49	0,13	0,53	0	-1,00	-0,01	0,98	1,00	0	-1,22	-0,29	0
ZDBF2	zinc finger, DBF-type containing 2	0,71	-0,49	0,12	0,51	0	NA	NA	NA	NA	0	NA	NA	0
TRIM2	tripartite motif-containing 2	0,71	-0,49	0,02	0,27	0	-1,40	-0,49	0,10	1,00	0	-1,14	-0,18	0
ACSL1	acyl-CoA synthetase long-chain family member 1	0,71	-0,49	0,04	0,32	0	-1,16	-0,21	0,39	1,00	0	-1,33	-0,42	0
CYP2J2	cytochrome P450, family 2, subfamily J, polypeptide 2	0,71	-0,49	0,11	0,48	0	1,02	0,03	0,94	1,00	0	NA	NA	0
NA	NA	0,71	-0,49	0,07	0,41	0	-1,15	-0,20	0,20	1,00	0	-1,32	-0,39	0
MYCL1	v-myc myelocytomatosis viral oncogene homolog 1, lung carcinoma derived (avian)	0,71	-0,50	0,17	0,57	0	-1,00	-0,01	0,95	1,00	0	NA	NA	0
VAMP1	vesicle-associated membrane protein 1 (synaptobrevin 1)	0,71	-0,50	0,05	0,37	0	1,02	0,03	0,90	1,00	0	-1,32	-0,39	0
AMZ2	archaelysin family metallopeptidase 2	0,71	-0,50	0,01	0,22	0	-1,13	-0,17	0,17	1,00	0	NA	NA	0
TACC2	transforming, acidic coiled-coil containing protein 2	0,71	-0,50	0,00	0,10	0	-1,07	-0,10	0,47	1,00	0	1,19	0,26	0
NA	NA	0,71	-0,50	0,03	0,29	0	-1,20	-0,27	0,42	1,00	0	NA	NA	0
LEPR	leptin receptor	0,71	-0,50	0,07	0,40	0	-1,00	-0,01	NA	NA	0	-1,19	-0,25	0
ENPEP	glutamyl aminopeptidase (aminopeptidase A)	0,71	-0,50	0,18	0,59	0	-1,01	-0,01	NA	NA	0	0,00	0,00	0
DCLRE1A	DNA cross-link repair 1A	0,71	-0,50	0,01	0,18	0	-1,02	-0,03	0,88	1,00	0	NA	NA	0
EFS	embryonal Fyn-associated substrate	0,71	-0,50	0,02	0,26	0	-1,32	-0,40	0,41	1,00	0	-1,43	-0,52	0
NNMT	nicotinamide N-methyltransferase	0,71	-0,50	0,07	0,42	0	-1,35	-0,43	0,47	1,00	0	NA	NA	0
ZC3H6	zinc finger CCCH-type containing 6	0,71	-0,50	0,01	0,15	0	NA	NA	NA	NA	0	NA	NA	0
LIPT1	lipoyltransferase 1	0,71	-0,50	0,06	0,40	0	1,02	0,03	0,90	1,00	0	-1,35	-0,43	0
NQO1	NAD(P)H dehydrogenase, quinone 1	0,71	-0,50	0,15	0,55	0	-1,26	-0,33	0,32	1,00	0	NA	NA	0
KIAA0528	KIAA0528	0,71	-0,50	0,01	0,21	0	-1,12	-0,17	0,31	1,00	0	-1,33	-0,42	0
SMARCA2	SWI/SNF related, matrix associated, actin dependent regulator of chromatin, subfamily a, member 2	0,71	-0,50	0,01	0,16	0	-1,28	-0,35	0,01	0,65	0	NA	NA	0
POT1	protection of telomeres 1 homolog (S. pombe)	0,71	-0,50	0,01	0,19	0	-1,03	-0,04	0,83	1,00	0	NA	NA	0
DPYSL3	dihydropyrimidinase-like 3	0,70	-0,51	0,26	0,66	0	-1,01	-0,01	NA	NA	0	0,00	0,00	0
SLC27A3	solute carrier family 27 (fatty acid transporter), member 3	0,70	-0,51	0,19	0,59	0	-1,32	-0,40	0,07	1,00	0	NA	NA	0
DIP2C	DIP2 disco-interacting protein 2 homolog C (Drosophila)	0,70	-0,51	0,01	0,19	0	-1,11	-0,15	0,68	1,00	0	-1,10	-0,13	0
SKP2	S-phase kinase-associated protein 2 (p45)	0,70	-0,51	0,02	0,25	0	-1,07	-0,10	0,50	1,00	0	NA	NA	0
CKLF	chemokine-like factor	0,70	-0,51	0,06	0,40	0	1,16	0,21	0,42	1,00	0	NA	NA	0
TNFSF13B	tumor necrosis factor (ligand) superfamily, member 13b	0,70	-0,51	0,05	0,37	0	NA	NA	NA	NA	0	NA	NA	0
NMI	N-myc (and STAT) interactor	0,70	-0,51	0,03	0,30	0	-1,10	-0,14	0,42	1,00	0	-1,47	-0,57	0
C8orf83	chromosome 8 open reading frame 83	0,70	-0,51	0,05	0,37	0	NA	NA	NA	NA	0	NA	NA	0
PDCD4	programmed cell death 4 (neoplastic transformation inhibitor)	0,70	-0,51	0,01	0,20	0	-1,14	-0,18	0,70	1,00	0	NA	NA	0
LY75	lymphocyte antigen 75	0,70	-0,51	0,23	0,63	0	-1,00	0,00	1,00	1,00	0	1,01	0,02	0
NA	NA	0,70	-0,51	0,10	0,48	0	1,02	0,03	0,89	1,00	0	NA	NA	0
TBC1D4	TBC1 domain family, member 4	0,70	-0,52	0,09	0,45	0	-1,09	-0,12	0,55	1,00	0	NA	NA	0
C12orf47	chromosome 12 open reading frame 47	0,70	-0,52	0,01	0,21	0	-1,15	-0,21	0,61	1,00	0	NA	NA	0
MTUS1	microtubule associated tumor suppressor 1	0,70	-0,52	0,45	0,79	0	-1,26	-0,33	0,46	1,00	0	NA	NA	0
NA	NA	0,70	-0,52	0,05	0,35	0	-1,04	-0,05	0,83	1,00	0	NA	NA	0
RBPMS	RNA binding protein with multiple splicing	0,70	-0,52	0,06	0,40	0	1,19	0,25	0,10	1,00	0	NA	NA	0
NBEA	neurobeachin	0,70	-0,52	0,12	0,51	0	1,00	0,00	NA	NA	0	-1,32	-0,40	0
PPAP2A	phosphatidic acid phosphatase type 2A	0,70	-0,52	0,03	0,31	0	1,08	0,11	0,57	1,00	0	1,01	0,02	0
ANKRA2	ankyrin repeat, family A (RFXANK-like), 2	0,70	-0,52	0,04	0,34	0	-1,15	-0,20	0,23	1,00	0	NA	NA	0
NA	NA	0,70	-0,52	0,04	0,33	0	-1,14	-0,18	0,21	1,00	0	-1,22	-0,29	0
ARHGDIB	Rho GDP dissociation inhibitor (GDI) beta	0,70	-0,52	0,19	0,60	0	-1,14	-0,19	0,25	1,00	0	-1,16	-0,21	0
C14orf139	chromosome 14 open reading frame 139	0,70	-0,52	0,09	0,44	0	-1,80	-0,85	0,01	0,53	0	NA	NA	0
EVI2A	ecotropic viral integration site 2A	0,70	-0,52	0,35	0,73	0	-1,01	-0,01	NA	NA	0	-1,52	-0,59	0
CSAD	cysteine sulfinic acid decarboxylase	0,70	-0,52	0,02	0,23	0	-1,00	-0,01	0,97	1,00	0	NA	NA	0
SAP30	Sin3A-associated protein, 30kDa	0,69	-0,53	0,04	0,32	0	1,13	0,18	0,25	1,00	0	NA	NA	0
CASP1	caspase 1, apoptosis-related cysteine peptidase (interleukin 1, beta, convertase)	0,69	-0,53	0,00	0,14	0	-1,09	-0,12	0,54	1,00	0	-1,14	-0,18	0
BST2	bone marrow stromal cell antigen 2	0,69	-0,53	0,39	0,76	0	-1,01	-0,01	0,88	1,00	0	0,00	0,00	0
NA	NA	0,69	-0,53	0,01	0,17	0	-1,10	-0,14	0,19	1,00	0	NA	NA	0
IRX4	iroquois homeobox 4	0,69	-0,53	0,15	0,55	0	-1,10	-0,14	0,33	1,00	0	NA	NA	0
UBE2L6	ubiquitin-conjugating enzyme E2L 6	0,69	-0,53	0,08	0,43	0	-1,23	-0,30	0,19	1,00	0	-1,43	-0,52	0
SETMAR	SET domain and mariner transposase fusion gene	0,69	-0,53	0,03	0,31	0	-1,07	-0,10	0,74	1,00	0	NA	NA	0
HOXC6	homeobox C6	0,69	-0,53	0,04	0,33	0	-1,17	-0,23	0,03	0,96	0	-1,14	-0,18	0
TUBB2B	tubulin, beta 2B	0,69	-0,53	0,23	0,64	0	-1,00	0,00	NA	NA	0	-1,18	-0,24	0
LACTB2	lactamase, beta 2	0,69	-0,53	0,25	0,66	0	-1,24	-0,31	0,33	1,00	0	-1,32	-0,40	0
CYBRD1	cytochrome b reductase 1	0,69	-0,53	0,07	0,41	0	-2,31	-1,21	0,00	0,45	0	NA	NA	0
RSAD2	radical S-adenosyl methionine domain containing 2	0,69	-0,53	0,41	0,77	0	1,00	0,01	0,98	1,00	0	0,00	0,00	0
HTATIP2	HIV-1 Tat interactive protein 2, 30kDa	0,69	-0,54	0,01	0,17	0	-1,09	-0,13	0,14	1,00	0	-1,06	-0,09	0
EAF2	ELL associated factor 2	0,69	-0,54	0,15	0,54	0	1,23	0,30	0,56	1,00	0	NA	NA	0
PRPF39	PRP39 pre-mRNA processing factor 39 homolog (S. cerevisiae)	0,69	-0,54	0,13	0,52	0	-1,09	-0,12	0,48	1,00	0	-1,56	-0,64	0
ZFP36L1	zinc finger protein 36, C3H type-like 1	0,69	-0,54	0,27	0,67	0	-2,13	-1,09	0,01	0,74	0	1,33	0,41	0
SDC2	syndecan 2	0,69	-0,54	0,18	0,59	0	-1,02	-0,02	0,80	1,00	0	-1,11	-0,15	0
SERPING1	serpin peptidase inhibitor, clade G (C1 inhibitor), member 1	0,69	-0,54	0,17	0,57	0	-1,13	-0,17	0,46	1,00	0	-1,33	-0,42	0
CYB5A	cytochrome b5 type A (microsomal)	0,69	-0,54	0,05	0,37	0	1,13	0,18	0,24	1,00	0	-1,14	-0,18	0
C9orf40	chromosome 9 open reading frame 40	0,69	-0,54	0,01	0,21	0	1,03	0,04	0,89	1,00	0	NA	NA	0
PDCD4	programmed cell death 4 (neoplastic transformation inhibitor)	0,69	-0,54	0,02	0,27	0	-1,09	-0,12	0,80	1,00	0	NA	NA	0
CHKA	choline kinase alpha	0,69	-0,54	0,03	0,29	0	1,04	0,05	0,78	1,00	0	NA	NA	0
IFI35	interferon-induced protein 35	0,69	-0,54	0,20	0,61	0	-1,17	-0,22	0,67	1,00	0	-1,23	-0,31	0
PPP1R3C	protein phosphatase 1, regulatory (inhibitor) subunit 3C	0,69	-0,54	0,16	0,56	0	1,18	0,23	0,66	1,00	0	NA	NA	0
PPIP5K2	diphosphoinositol pentakisphosphate kinase 2	0,69	-0,54	0,01	0,21	0	-1,08	-0,11	0,59	1,00	0	NA	NA	0
NA	NA	0,69	-0,54	0,03	0,29	0	-1,27	-0,35	0,25	1,00	0	-1,09	-0,12	0
IFI6	interferon, alpha-inducible protein 6	0,68	-0,55	0,39	0,75	0	-1,33	-0,42	0,26	1,00	0	-1,61	-0,70	0
NA	NA	0,68	-0,55	0,05	0,37	0	NA	NA	NA	NA	0	NA	NA	0
FSD1L	fibronectin type III and SPRY domain containing 1-like	0,68	-0,55	0,13	0,51	0	NA	NA	NA	NA	0	NA	NA	0
NA	NA	0,68	-0,55	0,01	0,21	0	-1,05	-0,07	0,88	1,00	0	NA	NA	0
NA	NA	0,68	-0,55	0,13	0,51	0	1,25	0,32	0,11	1,00	0	1,18	0,24	0
IFITM1	interferon induced transmembrane protein 1 (9-27)	0,68	-0,55	0,11	0,49	0	-1,22	-0,28	0,54	1,00	0	-1,45	-0,54	0
UBA7	ubiquitin-like modifier activating enzyme 7	0,68	-0,55	0,15	0,55	0	-1,00	-0,01	0,93	1,00	0	1,05	0,07	0
USP9Y	ubiquitin specific peptidase 9, Y-linked	0,68	-0,55	0,05	0,36	0	-1,36	-0,44	0,24	1,00	0	-1,33	-0,41	0
NAV2	neuron navigator 2	0,68	-0,55	0,01	0,20	0	-1,07	-0,09	0,64	1,00	0	NA	NA	0
TRIM22	tripartite motif-containing 22	0,68	-0,55	0,06	0,39	0	-1,02	-0,03	0,90	1,00	0	-1,16	-0,21	0
ZNF32	zinc finger protein 32	0,68	-0,55	0,01	0,22	0	-1,18	-0,24	0,25	1,00	0	NA	NA	0
KRIT1	KRIT1, ankyrin repeat containing	0,68	-0,55	0,04	0,34	0	-1,15	-0,20	0,31	1,00	0	NA	NA	0
NA	NA	0,68	-0,55	0,01	0,21	0	-1,24	-0,31	0,42	1,00	0	NA	NA	0
C6orf204	chromosome 6 open reading frame 204	0,68	-0,55	0,05	0,35	0	NA	NA	NA	NA	0	NA	NA	0
CROT	carnitine O-octanoyltransferase	0,68	-0,55	0,01	0,18	0	1,02	0,03	0,86	1,00	0	NA	NA	0
C1orf38	chromosome 1 open reading frame 38	0,68	-0,56	0,06	0,39	0	1,01	0,02	NA	NA	0	-1,03	-0,04	0
DST	dystonin	0,68	-0,56	0,11	0,49	0	-1,32	-0,40	0,11	1,00	0	1,03	0,05	0
OAZ3	ornithine decarboxylase antizyme 3	0,68	-0,56	0,11	0,48	0	-1,01	-0,02	0,96	1,00	0	NA	NA	0
ERCC6	excision repair cross-complementing rodent repair deficiency, complementation group 6	0,68	-0,56	0,17	0,57	0	-1,13	-0,18	0,55	1,00	0	-1,45	-0,53	0
NA	NA	0,68	-0,56	0,01	0,21	0	-1,21	-0,27	0,27	1,00	0	-1,35	-0,43	0
FECH	ferrochelatase	0,68	-0,56	0,01	0,22	0	-1,09	-0,13	0,18	1,00	0	-1,23	-0,30	0
C11orf61	chromosome 11 open reading frame 61	0,68	-0,56	0,01	0,21	0	-1,08	-0,12	0,69	1,00	0	NA	NA	0
NA	NA	0,68	-0,56	0,02	0,23	0	NA	NA	NA	NA	0	NA	NA	0
FAM164A	family with sequence similarity 164, member A	0,68	-0,56	0,00	0,14	0	-1,02	-0,03	0,86	1,00	0	NA	NA	0
RNASE4	ribonuclease, RNase A family, 4	0,68	-0,56	0,01	0,20	0	1,09	0,12	0,73	1,00	0	-1,15	-0,20	0
PTPRZ1	protein tyrosine phosphatase, receptor-type, Z polypeptide 1	0,68	-0,56	0,07	0,40	0	-1,11	-0,16	0,17	1,00	0	NA	NA	0
SLC38A6	solute carrier family 38, member 6	0,68	-0,56	0,09	0,46	0	-1,01	-0,02	0,94	1,00	0	NA	NA	0
SLC7A11	solute carrier family 7, (cationic amino acid transporter, y+ system) member 11	0,68	-0,56	0,12	0,51	0	-1,35	-0,43	0,03	1,00	0	-1,02	-0,03	0
PTGIS	prostaglandin I2 (prostacyclin) synthase	0,68	-0,57	0,05	0,37	0	-1,00	0,00	NA	NA	0	-1,45	-0,54	0
SH3YL1	SH3 domain containing, Ysc84-like 1 (S. cerevisiae)	0,68	-0,57	0,01	0,15	0	-1,03	-0,05	0,60	1,00	0	-1,23	-0,30	0
EFHC1	EF-hand domain (C-terminal) containing 1	0,68	-0,57	0,03	0,30	0	-1,33	-0,41	0,02	0,94	0	NA	NA	0
DNAJB4	DnaJ (Hsp40) homolog, subfamily B, member 4	0,68	-0,57	0,04	0,33	0	-1,36	-0,45	0,48	1,00	0	-1,23	-0,30	0
TREX1	three prime repair exonuclease 1	0,67	-0,57	0,01	0,22	0	-1,01	-0,01	0,93	1,00	0	1,10	0,14	0
HIST1H2BC	histone cluster 1, H2bc	0,67	-0,57	0,08	0,43	0	1,41	0,49	0,18	1,00	0	NA	NA	0
HIF3A	hypoxia inducible factor 3, alpha subunit	0,67	-0,57	0,07	0,41	0	NA	NA	NA	NA	0	NA	NA	0
MOCOS	molybdenum cofactor sulfurase	0,67	-0,57	0,01	0,21	0	-1,09	-0,12	0,78	1,00	0	-1,47	-0,56	0

DCUN1D4	DCN1, defective in cullin neddylation 1, domain containing 4 (S. cerevisiae)	0,67	-0,57	0,05	0,35	0	-1,10	-0,13	0,68	1,00	0	-1,41	-0,49	0
TUBA1A	tubulin, alpha 1a	0,67	-0,57	0,03	0,29	0	1,05	0,07	0,63	1,00	0	NA	NA	0
TNFRSF14	tumor necrosis factor receptor superfamily, member 14 (herpesvirus entry mediator)	0,67	-0,57	0,01	0,22	0	-1,10	-0,13	0,42	1,00	0	0,00	0,00	0
TBC1D8	TBC1 domain family, member 8 (with GRAM domain)	0,67	-0,57	0,03	0,31	0	-1,15	-0,21	0,05	1,00	0	NA	NA	0
RAD54B	RAD54 homolog B (S. cerevisiae)	0,67	-0,57	0,05	0,36	0	1,25	0,32	0,06	1,00	0	-1,25	-0,32	0
FZD1	frizzled homolog 1 (Drosophila)	0,67	-0,57	0,02	0,28	0	-1,01	-0,02	0,92	1,00	0	NA	NA	0
NSL1	NSL1, MIND kinetochore complex component, homolog (S. cerevisiae)	0,67	-0,57	0,05	0,36	0	-1,03	-0,04	NA	NA	0	-1,02	-0,02	0
MXRA5	matrix-remodelling associated 5	0,67	-0,57	0,08	0,42	0	-1,47	-0,56	0,14	1,00	0	NA	NA	0
ABCA5	ATP-binding cassette, sub-family A (ABC1), member 5	0,67	-0,58	0,00	0,13	0	-1,17	-0,22	0,27	1,00	0	-1,01	-0,02	0
LEPR	leptin receptor	0,67	-0,58	0,02	0,26	0	-1,06	-0,08	NA	NA	0	-1,45	-0,53	0
OPTN	optineurin	0,67	-0,58	0,02	0,23	0	-1,10	-0,13	0,39	1,00	0	-1,16	-0,21	0
ITM2A	integral membrane protein 2A	0,67	-0,58	0,21	0,61	0	-1,21	-0,28	0,34	1,00	0	NA	NA	0
ALDH3A2	aldehyde dehydrogenase 3 family, member A2	0,67	-0,58	0,03	0,28	0	-1,21	-0,27	0,06	1,00	0	NA	NA	0
CKLF	chemokine-like factor	0,67	-0,58	0,06	0,39	0	-1,08	-0,11	0,77	1,00	0	NA	NA	0
ARL4C	ADP-ribosylation factor-like 4C	0,67	-0,58	0,02	0,25	0	1,07	0,10	0,56	1,00	0	NA	NA	0
TK2	thymidine kinase 2, mitochondrial	0,67	-0,58	0,01	0,22	0	-1,07	-0,10	0,73	1,00	0	NA	NA	0
NA	NA	0,67	-0,58	0,13	0,51	0	-1,00	0,00	NA	NA	0	-1,19	-0,26	0
MBNL2	muscleblind-like 2 (Drosophila)	0,67	-0,58	0,00	0,11	0	-1,23	-0,30	0,10	1,00	0	-1,23	-0,31	0
GLRB	glycine receptor, beta	0,67	-0,58	0,03	0,29	0	-1,10	-0,14	NA	NA	0	1,14	0,18	0
HEG1	HEG homolog 1 (zebrafish)	0,67	-0,58	0,02	0,27	0	1,03	0,04	0,85	1,00	0	NA	NA	0
C3orf14	chromosome 3 open reading frame 14	0,67	-0,58	0,04	0,32	0	-1,15	-0,20	0,29	1,00	0	-1,09	-0,12	0
DLEU1	deleted in lymphocytic leukemia 1 (non-protein coding)	0,67	-0,58	0,25	0,65	0	1,12	0,17	0,54	1,00	0	-1,22	-0,28	0
NOD1	nucleotide-binding oligomerization domain containing 1	0,67	-0,58	0,05	0,36	0	-1,00	0,00	1,00	1,00	0	NA	NA	0
PLAGL1	pleiomorphic adenoma gene-like 1	0,67	-0,59	0,01	0,22	0	1,03	0,05	0,81	1,00	0	NA	NA	0
ITGB5	integrin, beta 5	0,66	-0,59	0,02	0,23	0	-1,17	-0,22	0,55	1,00	0	-1,37	-0,45	0
PRR5L	proline rich 5 like	0,66	-0,59	0,02	0,26	0	-1,22	-0,29	0,06	1,00	0	NA	NA	0
NA	NA	0,66	-0,59	0,33	0,71	0	NA	NA	NA	NA	0	NA	NA	0
ZNF432	zinc finger protein 432	0,66	-0,59	0,01	0,16	0	1,47	0,56	0,07	1,00	0	NA	NA	0
ESR1	estrogen receptor 1	0,66	-0,59	0,20	0,60	0	-1,34	-0,42	0,20	1,00	0	NA	NA	0
LGALS9	lectin, galactoside-binding, soluble, 9	0,66	-0,59	0,42	0,77	0	-1,03	-0,04	0,71	1,00	0	0,00	0,01	0
PLK1S1	polo-like kinase 1 substrate 1	0,66	-0,59	0,09	0,45	0	1,06	0,08	0,82	1,00	0	-1,16	-0,22	0
GATAD1	GATA zinc finger domain containing 1	0,66	-0,59	0,01	0,18	0	1,05	0,07	0,76	1,00	0	-1,25	-0,31	0
PITX2	paired-like homeodomain 2	0,66	-0,60	0,02	0,23	0	1,20	0,26	0,03	0,96	0	NA	NA	0
OLFML1	olfactomedin-like 1	0,66	-0,60	0,20	0,60	0	-1,01	-0,01	NA	NA	0	-1,01	-0,01	0
ARHGAP6	Rho GTPase activating protein 6	0,66	-0,60	0,10	0,48	0	-1,01	-0,01	NA	NA	0	-1,35	-0,43	0
FAM179B	family with sequence similarity 179, member B	0,66	-0,60	0,02	0,25	0	-1,06	-0,09	0,72	1,00	0	NA	NA	0
SKP2	S-phase kinase-associated protein 2 (p45)	0,66	-0,60	0,16	0,56	0	-1,29	-0,37	0,43	1,00	0	-1,30	-0,37	0
DYNC2LI1	dynein, cytoplasmic 2, light intermediate chain 1	0,66	-0,60	0,01	0,20	0	-1,11	-0,15	0,40	1,00	0	NA	NA	0
BTN3A3	butyrophilin, subfamily 3, member A3	0,66	-0,60	0,03	0,29	0	-1,30	-0,38	0,42	1,00	0	-1,32	-0,40	0
SEPX1	selenoprotein X, 1	0,66	-0,61	0,10	0,48	0	1,00	0,00	0,98	1,00	0	1,19	0,25	0
LPCAT4	lysophosphatidylcholine acyltransferase 4	0,66	-0,61	0,50	0,82	0	-1,03	-0,05	0,84	1,00	0	NA	NA	0
PTPLA	protein tyrosine phosphatase-like (proline instead of catalytic arginine), member A	0,66	-0,61	0,05	0,36	0	-1,10	-0,14	0,36	1,00	0	NA	NA	0
ALDH3A2	aldehyde dehydrogenase 3 family, member A2	0,66	-0,61	0,03	0,29	0	-1,58	-0,66	0,33	1,00	0	NA	NA	0
GSDMB	gasdermin B	0,66	-0,61	0,12	0,50	0	-1,01	-0,01	0,92	1,00	0	NA	NA	0
MTUS1	microtubule associated tumor suppressor 1	0,65	-0,61	0,17	0,57	0	1,07	0,10	0,58	1,00	0	NA	NA	0
COMMD8	COMM domain containing 8	0,65	-0,61	0,03	0,31	0	-1,15	-0,20	0,15	1,00	0	NA	NA	0
L1CAM	L1 cell adhesion molecule	0,65	-0,61	0,01	0,22	0	-1,05	-0,07	0,88	1,00	0	NA	NA	0
NA	NA	0,65	-0,62	0,02	0,28	0	NA	NA	NA	NA	0	NA	NA	0
ALDH3A2	aldehyde dehydrogenase 3 family, member A2	0,65	-0,62	0,00	0,13	0	-1,16	-0,21	0,18	1,00	0	NA	NA	0
HOXA5	homeobox A5	0,65	-0,62	0,12	0,50	0	-1,17	-0,23	0,45	1,00	0	NA	NA	0
GBP2	guanylate binding protein 2, interferon-inducible	0,65	-0,62	0,26	0,66	0	1,00	0,01	0,95	1,00	0	1,04	0,06	0
MEIS2	Meis homeobox 2	0,65	-0,62	0,01	0,19	0	-1,17	-0,23	0,16	1,00	0	NA	NA	0
KLHL23	kelch-like 23 (Drosophila)	0,65	-0,62	0,06	0,39	0	-1,12	-0,16	0,62	1,00	0	1,01	0,01	0
SEMA4D	sema domain, immunoglobulin domain (Ig), transmembrane domain (TM) and short cytoplasmic domain, (semaphorin) 4D	0,65	-0,62	0,00	0,14	0	NA	NA	NA	NA	0	NA	NA	0
ACN9	ACN9 homolog (S. cerevisiae)	0,65	-0,62	0,14	0,53	0	-1,12	-0,17	0,45	1,00	0	NA	NA	0
C14orf143	chromosome 14 open reading frame 143	0,65	-0,62	0,01	0,17	0	-1,27	-0,34	0,22	1,00	0	NA	NA	0
BTN3A3	butyrophilin, subfamily 3, member A3	0,65	-0,62	0,03	0,29	0	-1,17	-0,22	0,32	1,00	0	-1,35	-0,42	0
STBD1	starch binding domain 1	0,65	-0,62	0,01	0,20	0	-1,03	-0,04	0,61	1,00	0	1,35	0,44	0
D4S234E	DNA segment on chromosome 4 (unique) 234 expressed sequence	0,65	-0,62	0,05	0,35	0	-1,07	-0,10	0,56	1,00	0	NA	NA	0
CENPC1	centromere protein C 1	0,65	-0,62	0,02	0,27	0	1,18	0,24	0,21	1,00	0	-1,56	-0,66	0
MDFIC	MyoD family inhibitor domain containing	0,65	-0,62	0,04	0,34	0	-1,33	-0,41	0,24	1,00	0	-1,14	-0,18	0
TDRD7	tudor domain containing 7	0,65	-0,62	0,18	0,59	0	-1,28	-0,36	0,09	1,00	0	-1,22	-0,29	0
PRKY	protein kinase, Y-linked	0,65	-0,62	0,01	0,19	0	1,11	0,15	0,63	1,00	0	NA	NA	0
FNBP1L	formin binding protein 1-like	0,65	-0,62	0,03	0,31	0	1,00	0,00	0,98	1,00	0	NA	NA	0
DCUN1D4	DCN1, defective in cullin neddylation 1, domain containing 4 (S. cerevisiae)	0,65	-0,62	0,16	0,56	0	-1,08	-0,11	0,59	1,00	0	-1,47	-0,55	0
NA	NA	0,65	-0,63	0,10	0,47	0	NA	NA	NA	NA	0	NA	NA	0
CLEC7A	C-type lectin domain family 7, member A	0,65	-0,63	0,21	0,62	0	-1,01	-0,01	0,98	1,00	0	NA	NA	0
THYN1	thymocyte nuclear protein 1	0,65	-0,63	0,00	0,14	0	-1,08	-0,11	0,64	1,00	0	NA	NA	0
NA	NA	0,65	-0,63	0,00	0,11	0	NA	NA	NA	NA	0	NA	NA	0
C12orf47	chromosome 12 open reading frame 47	0,65	-0,63	0,06	0,38	0	-1,27	-0,34	0,31	1,00	0	NA	NA	0
POT1	protection of telomeres 1 homolog (S. pombe)	0,65	-0,63	0,04	0,32	0	-1,19	-0,25	0,48	1,00	0	NA	NA	0
SLC6A16	solute carrier family 6, member 16	0,64	-0,63	0,00	0,13	0	NA	NA	NA	NA	0	NA	NA	0
AIM2	absent in melanoma 2	0,64	-0,63	0,52	0,83	0	-1,00	-0,01	0,93	1,00	0	0,00	0,00	0
CCPG1	cell cycle progression 1	0,64	-0,63	0,07	0,42	0	-1,02	-0,02	0,88	1,00	0	-1,08	-0,10	0
PHF17	PHD finger protein 17	0,64	-0,64	0,02	0,27	0	-1,01	-0,01	0,98	1,00	0	-1,30	-0,37	0
SPAG16	sperm associated antigen 16	0,64	-0,64	0,05	0,35	0	-1,22	-0,29	0,11	1,00	0	-1,25	-0,31	0
CLK4	CDC-like kinase 4	0,64	-0,65	0,04	0,32	0	-1,11	-0,16	0,68	1,00	0	-1,43	-0,51	0
PLXDC1	plexin domain containing 1	0,64	-0,65	0,11	0,49	0	-1,02	-0,02	0,85	1,00	0	NA	NA	0
NA	NA	0,64	-0,65	0,05	0,36	0	NA	NA	NA	NA	0	NA	NA	0
SAMHD1	SAM domain and HD domain 1	0,64	-0,65	0,22	0,62	0	1,11	0,15	0,70	1,00	0	-1,67	-0,74	0
APOL6	apolipoprotein L, 6	0,64	-0,65	0,29	0,69	0	-1,00	-0,01	0,94	1,00	0	0,00	0,00	0
PLEKHA4	pleckstrin homology domain containing, family A (phosphoinositide binding specific) member 4	0,64	-0,65	0,04	0,32	0	-1,01	-0,01	NA	NA	0	0,00	0,00	0
PDE4DIP	phosphodiesterase 4D interacting protein	0,64	-0,65	0,05	0,37	0	-1,13	-0,17	0,68	1,00	0	NA	NA	0
NA	NA	0,64	-0,65	0,01	0,22	0	-1,23	-0,30	0,28	1,00	0	-1,08	-0,10	0
D4S234E	DNA segment on chromosome 4 (unique) 234 expressed sequence	0,64	-0,65	0,04	0,34	0	-1,22	-0,29	0,06	1,00	0	NA	NA	0
C14orf45	chromosome 14 open reading frame 45	0,64	-0,65	0,02	0,26	0	1,01	0,02	NA	NA	0	-1,09	-0,12	0
ACSF2	acyl-CoA synthetase family member 2	0,64	-0,65	0,06	0,38	0	-1,04	-0,05	0,82	1,00	0	NA	NA	0
PRKAR2B	protein kinase, cAMP-dependent, regulatory, type II, beta	0,63	-0,66	0,24	0,65	0	-1,01	-0,01	NA	NA	0	-1,39	-0,48	0
SECTM1	secreted and transmembrane 1	0,63	-0,66	0,28	0,68	0	1,03	0,05	0,85	1,00	0	1,02	0,03	0
VGLL3	vestigial like 3 (Drosophila)	0,63	-0,66	0,11	0,48	0	-1,03	-0,04	0,76	1,00	0	-1,12	-0,17	0
PLLP	plasmolipin	0,63	-0,67	0,14	0,54	0	-1,23	-0,30	0,20	1,00	0	NA	NA	0
DNAJB4	DnaJ (Hsp40) homolog, subfamily B, member 4	0,62	-0,68	0,01	0,20	0	-1,30	-0,37	0,06	1,00	0	NA	NA	0
TAP1	transporter 1, ATP-binding cassette, sub-family B (MDR/TAP)	0,62	-0,68	0,04	0,35	0	-1,30	-0,38	0,01	0,74	0	-1,28	-0,36	0
C1orf63	chromosome 1 open reading frame 63	0,62	-0,68	0,16	0,56	0	-1,07	-0,10	0,87	1,00	0	-1,37	-0,46	0
ALDH2	aldehyde dehydrogenase 2 family (mitochondrial)	0,62	-0,68	0,01	0,19	0	-1,10	-0,13	0,41	1,00	0	-1,19	-0,26	0
D4S234E	DNA segment on chromosome 4 (unique) 234 expressed sequence	0,62	-0,68	0,01	0,17	0	-1,28	-0,36	0,13	1,00	0	NA	NA	0
HEG1	HEG homolog 1 (zebrafish)	0,62	-0,68	0,17	0,58	0	-1,01	-0,01	0,93	1,00	0	NA	NA	0
NA	NA	0,62	-0,69	0,00	0,13	0	1,02	0,03	0,84	1,00	0	1,04	0,06	0
FAM13A	family with sequence similarity 13, member A	0,62	-0,69	0,06	0,38	0	1,03	0,04	0,80	1,00	0	NA	NA	0
ACP6	acid phosphatase 6, lysophosphatidic	0,62	-0,69	0,04	0,32	0	-1,04	-0,05	0,66	1,00	0	-1,39	-0,48	0
ARL4A	ADP-ribosylation factor-like 4A	0,62	-0,69	0,06	0,38	0	-1,05	-0,08	0,48	1,00	0	NA	NA	0
NA	NA	0,62	-0,69	0,01	0,16	0	NA	NA	NA	NA	0	NA	NA	0
SSBP2	single-stranded DNA binding protein 2	0,62	-0,69	0,00	0,14	0	-1,10	-0,13	0,44	1,00	0	-1,39	-0,48	0
APOBEC3G	apolipoprotein B mRNA editing enzyme, catalytic polypeptide-like 3G	0,62	-0,69	0,19	0,59	0	1,02	0,03	0,89	1,00	0	-1,19	-0,26	0
NA	NA	0,62	-0,69	0,02	0,23	0	-1,13	-0,18	0,46	1,00	0	NA	NA	0
ITM2A	integral membrane protein 2A	0,62	-0,69	0,17	0,57	0	-1,19	-0,26	0,11	1,00	0	NA	NA	0
HERC5	hect domain and RLD 5	0,62	-0,69	0,03	0,29	0	-1,02	-0,03	0,95	1,00	0	-1,03	-0,05	0
NA	NA	0,62	-0,70	0,00	0,13	0	1,18	0,23	0,32	1,00	0	NA	NA	0
MARCKSL1	MARCKS-like 1	0,62	-0,70	0,26	0,67	0	-1,04	-0,06	0,80	1,00	0	NA	NA	0
FGFR2	fibroblast growth factor receptor 2	0,62	-0,70	0,12	0,51	0	1,18	0,24	0,28	1,00	0	NA	NA	0
CDK18	cyclin-dependent kinase 18	0,62	-0,70	0,20	0,61	0	1,00	0,00	1,00	1,00	0	NA	NA	0
NA	NA	0,62	-0,70	0,02	0,23	0	NA	NA	NA	NA	0	NA	NA	0
SMYD3	SET and MYND domain containing 3	0,62	-0,70	0,05	0,35	0	-1,06	-0,09	0,61	1,00	0	NA	NA	0
IFI30	interferon, gamma-inducible protein 30	0,62	-0,70	0,05	0,36	0	-1,13	-0,18	0,42	1,00	0	-1,41	-0,49	0
SESN1	sestrin 1	0,61	-0,71	0,01	0,17	0	-1,03	-0,04	0,86	1,00	0	NA	NA	0
MEF2C	myocyte enhancer factor 2C	0,61	-0,71	0,01	0,22	0	-1,00	-0,01	NA	NA	0	-1,05	-0,08	0
TNIK	TRAF2 and NCK interacting kinase	0,61	-0,71	0,01	0,22	0	-1,17	-0,22	0,50	1,00	0	NA	NA	0
SLC7A11	solute carrier family 7, (cationic amino acid transporter, y+ system) member 11	0,61	-0,71	0,02	0,25	0	-1,24	-0,31	0,13	1,00	0	-1,05	-0,07	0
NA	NA	0,61	-0,72	0,17	0,57	0	-1,00	0,00	0,99	1,00	0	-1,01	-0,01	0
NRN1	neuritin 1	0,61	-0,72	0,15	0,55	0	-1,01	-0,01	NA	NA	0	-1,25	-0,32	0
FAM63A	family with sequence similarity 63, member A	0,61	-0,72	0,01	0,22	0	-1,09	-0,12	0,74	1,00	0	NA	NA	0
C1orf38	chromosome 1 open reading frame 38	0,61	-0,72	0,02	0,27	0	-1,00	0,00	NA	NA	0	-1,04	-0,06	0
COBL	cordon-bleu homolog (mouse)	0,61	-0,72	0,02	0,23	0	-1,03	-0,04	0,94	1,00	0	NA	NA	0
PRKX	protein kinase, X-linked	0,61	-0,72	0,01	0,21	0	1,08	0,10	0,62	1,00	0	-1,11	-0,16	0
DIO2	deiodinase, iodothyronine, type II	0,61	-0,72	0,36	0,74	0	1,02	0,03	NA	NA	0	1,19	0,25	0
CCDC68	coiled-coil domain containing 68	0,60	-0,73	0,02	0,26	0	-1,07	-0,10	0,44	1,00	0	-1,27	-0,33	0
IGFBP2	insulin-like growth factor binding protein 2, 36kDa	0,60	-0,73	0,35	0,73	0	-1,29	-0,37	0,12	1,00	0	NA	NA	0
BCL2L11	BCL2-like 11 (apoptosis facilitator)	0,60	-0,73	0,21	0,62	0	-1,04	-0,06	0,85	1,00	0	NA	NA	0
CCPG1	cell cycle progression 1	0,60	-0,73	0,04	0,33	0	-1,07	-0,09	0,40	1,00	0	-1,14	-0,19	0
SGK494	uncharacterized serine/threonine-protein kinase SgK494	0,60	-0,73	0,03	0,29	0	NA	NA	NA	NA	0	NA	NA	0
KRT13	keratin 13	0,60	-0,74	0,61	0,88	0	-1,54	-0,63	0,06	1,00	0	NA	NA	0
SMARCA2	SWI/SNF related, matrix associated, actin dependent regulator of chromatin, subfamily a, member 2	0,60	-0,74	0,11	0,49	0	-1,29	-0,37	0,09	1,00	0	NA	NA	0
SMARCA2	SWI/SNF related, matrix associated, actin dependent regulator of chromatin, subfamily a, member 2	0,60	-0,74	0,02	0,27	0	-1,67	-0,74	0,15	1,00	0	NA	NA	0
SERPINI1	serpin peptidase inhibitor, clade I (neuroserpin), member 1	0,60	-0,74	0,01	0,16	0	-1,18	-0,24	0,38	1,00	0	NA	NA	0
MPP1	membrane protein, palmitoylated 1, 55kDa	0,60	-0,75	0,03	0,31	0	-1,06	-0,08	NA	NA	0	-1,27	-0,35	0
RNF128	ring finger protein 128	0,60	-0,75	0,05	0,37	0	-1,18	-0,24	0,28	1,00	0	NA	NA	0
CEP68	centrosomal protein 68kDa	0,60	-0,75	0,01	0,20	0	-1,11	-0,15	0,62	1,00	0	NA	NA	0

PARP8	poly (ADP-ribose) polymerase family, member 8	0,59	-0,75	0,07	0,41	0	-1,02	-0,03	0,89	1,00	0	0,00	0,00	0
GLRB	glycine receptor, beta	0,59	-0,75	0,03	0,31	0	1,23	0,30	0,11	1,00	0	-1,16	-0,22	0
TP53AIP1	tumor protein p53 regulated apoptosis inducing protein 1	0,59	-0,76	0,07	0,40	0	-1,12	-0,16	0,73	1,00	0	NA	NA	0
CA2	carbonic anhydrase II	0,59	-0,76	0,00	0,10	0	1,06	0,08	0,73	1,00	0	NA	NA	0
GRIK2	glutamate receptor, ionotropic, kainate 2	0,59	-0,76	0,13	0,52	0	-1,01	-0,01	NA	NA	0	-1,04	-0,07	0
NA	NA	0,59	-0,76	0,01	0,17	0	NA	NA	NA	NA	0	NA	NA	0
RABGAP1L	RAB GTPase activating protein 1-like	0,59	-0,76	0,03	0,30	0	-1,13	-0,17	0,63	1,00	0	-1,11	-0,15	0
NA	NA	0,59	-0,77	0,24	0,64	0	-1,03	-0,04	0,73	1,00	0	NA	NA	0
ZNF107	zinc finger protein 107	0,59	-0,77	0,02	0,25	0	1,03	0,04	0,85	1,00	0	-1,54	-0,62	0
FGFR2	fibroblast growth factor receptor 2	0,59	-0,77	0,15	0,55	0	1,04	0,06	0,78	1,00	0	NA	NA	0
NLRC5	NLR family, CARD domain containing 5	0,59	-0,77	0,01	0,21	0	NA	NA	NA	NA	0	NA	NA	0
LEPR	leptin receptor	0,58	-0,78	0,00	0,12	0	-1,01	-0,01	NA	NA	0	-1,54	-0,62	0
ARID5A	AT rich interactive domain 5A (MRF1-like)	0,58	-0,78	0,05	0,38	0	-1,03	-0,04	NA	NA	0	1,15	0,20	0
MGST2	microsomal glutathione S-transferase 2	0,58	-0,79	0,02	0,23	0	-1,03	-0,04	0,77	1,00	0	-1,12	-0,17	0
VSIG10	V-set and immunoglobulin domain containing 10	0,58	-0,79	0,01	0,19	0	-1,10	-0,13	0,60	1,00	0	NA	NA	0
LOX	lysyl oxidase	0,58	-0,79	0,20	0,61	0	-1,01	-0,01	NA	NA	0	-1,19	-0,25	0
DYRK2	dual-specificity tyrosine-(Y)-phosphorylation regulated kinase 2	0,58	-0,79	0,06	0,39	0	-1,11	-0,15	0,56	1,00	0	NA	NA	0
HOMER2	homer homolog 2 (Drosophila)	0,58	-0,80	0,04	0,34	0	1,03	0,04	0,78	1,00	0	NA	NA	0
RHOBTB1	Rho-related BTB domain containing 1	0,58	-0,80	0,02	0,23	0	-1,21	-0,28	0,47	1,00	0	NA	NA	0
NA	NA	0,58	-0,80	0,01	0,20	0	NA	NA	NA	NA	0	NA	NA	0
KCTD7	potassium channel tetramerisation domain containing 7	0,58	-0,80	0,02	0,27	0	-1,01	-0,01	0,95	1,00	0	-1,10	-0,13	0
MTUS1	microtubule associated tumor suppressor 1	0,57	-0,80	0,16	0,56	0	1,33	0,41	0,57	1,00	0	NA	NA	0
REV3L	REV3-like, catalytic subunit of DNA polymerase zeta (yeast)	0,57	-0,80	0,01	0,22	0	-1,28	-0,35	0,23	1,00	0	NA	NA	0
GBP1	guanylate binding protein 1, interferon-inducible, 67kDa	0,57	-0,81	0,16	0,57	0	1,11	0,15	0,55	1,00	0	-1,33	-0,42	0
TMSB15B	thymosin beta 15B	0,57	-0,81	0,06	0,40	0	-1,12	-0,17	0,39	1,00	0	-1,47	-0,56	0
DDX60	DEAD (Asp-Glu-Ala-Asp) box polypeptide 60	0,57	-0,81	0,14	0,53	0	-1,60	-0,68	0,11	1,00	0	-1,28	-0,35	0
LOC100128416	RAB28, member RAS oncogene family pseudogene	0,57	-0,82	0,00	0,14	0	NA	NA	NA	NA	0	NA	NA	0
NFE2	nuclear factor (erythroid-derived 2), 45kDa	0,57	-0,82	0,01	0,20	0	-1,01	-0,01	NA	NA	0	NA	NA	0
NA	NA	0,57	-0,82	0,09	0,46	0	NA	NA	NA	NA	0	NA	NA	0
APOL2	apolipoprotein L, 2	0,56	-0,83	0,03	0,30	0	-1,00	0,00	0,97	1,00	0	0,00	0,00	0
BCHE	butyrylcholinesterase	0,56	-0,84	0,17	0,58	0	-1,32	-0,40	0,44	1,00	0	NA	NA	0
MATN2	matrilin 2	0,55	-0,85	0,00	0,14	0	-1,25	-0,33	0,35	1,00	0	-1,43	-0,51	0
TSGA10	testis specific, 10	0,55	-0,85	0,00	0,13	0	NA	NA	NA	NA	0	NA	NA	0
SPATA7	spermatogenesis associated 7	0,55	-0,86	0,03	0,28	0	1,03	0,04	0,85	1,00	0	NA	NA	0
NA	NA	0,55	-0,86	0,01	0,17	0	NA	NA	NA	NA	0	NA	NA	0
HRASLS2	HRAS-like suppressor 2	0,55	-0,87	0,38	0,75	0	-1,15	-0,21	0,73	1,00	0	NA	NA	0
FLT3LG	fms-related tyrosine kinase 3 ligand	0,55	-0,87	0,03	0,28	0	-1,03	-0,04	0,69	1,00	0	-1,01	-0,01	0
GALNT12	UDP-N-acetyl-alpha-D-galactosamine:polypeptide N-acetylgalactosaminyltransferase 12 (GalNAc-T12)	0,54	-0,88	0,39	0,76	0	-1,02	-0,02	0,79	1,00	0	-1,02	-0,03	0
GHR	growth hormone receptor	0,54	-0,88	0,06	0,38	0	1,01	0,01	0,97	1,00	0	NA	NA	0
ADAMTS1	ADAM metallopeptidase with thrombospondin type 1 motif, 1	0,53	-0,91	0,05	0,37	0	-1,05	-0,07	0,77	1,00	0	-1,37	-0,46	0
MDM1	Mdm1 nuclear protein homolog (mouse)	0,53	-0,91	0,01	0,17	0	-1,07	-0,10	0,75	1,00	0	NA	NA	0
GPC1	glypican 1	0,53	-0,92	0,02	0,24	0	-1,15	-0,20	0,43	1,00	0	1,04	0,06	0
IL15	interleukin 15	0,53	-0,92	0,04	0,32	0	-1,04	-0,05	0,69	1,00	0	-1,19	-0,25	0
VPS41	vacuolar protein sorting 41 homolog (S. cerevisiae)	0,53	-0,93	0,01	0,19	0	1,03	0,05	0,64	1,00	0	NA	NA	0
NA	NA	0,52	-0,93	0,02	0,28	0	-1,00	-0,01	0,93	1,00	0	-1,11	-0,16	0
TRIM38	tripartite motif-containing 38	0,52	-0,93	0,01	0,22	0	-1,34	-0,42	0,04	1,00	0	-1,30	-0,38	0
PXMP4	peroxisomal membrane protein 4, 24kDa	0,52	-0,94	0,01	0,17	0	-1,09	-0,12	0,60	1,00	0	NA	NA	0
C10orf116	chromosome 10 open reading frame 116	0,52	-0,95	0,20	0,60	0	1,02	0,04	0,90	1,00	0	NA	NA	0
MDK	midkine (neurite growth-promoting factor 2)	0,51	-0,96	0,31	0,70	0	-1,09	-0,12	0,41	1,00	0	1,13	0,17	0
LPCAT4	lysophosphatidylcholine acyltransferase 4	0,51	-0,98	0,19	0,60	0	-1,20	-0,26	0,39	1,00	0	NA	NA	0
PRSS12	protease, serine, 12 (neurotrypsin, motopsin)	0,51	-0,98	0,03	0,30	0	1,25	0,32	0,46	1,00	0	-1,18	-0,23	0
NA	NA	0,51	-0,98	0,04	0,33	0	1,00	0,00	1,00	1,00	0	0,00	0,01	0
LOC100272216	hypothetical LOC100272216	0,50	-1,01	0,07	0,42	0	-1,30	-0,38	0,28	1,00	0	NA	NA	0
BBOX1	butyrobetaine (gamma), 2-oxoglutarate dioxygenase (gamma-butyrobetaine hydroxylase) 1	0,49	-1,02	0,01	0,20	0	-1,59	-0,67	0,04	1,00	0	NA	NA	0
OAS1	2',5'-oligoadenylate synthetase 1, 40/46kDa	0,49	-1,02	0,08	0,44	0	-1,35	-0,44	0,28	1,00	0	-1,05	-0,07	0
HLA-F	major histocompatibility complex, class I, F	0,49	-1,04	0,08	0,44	0	1,00	0,01	0,95	1,00	0	0,00	0,00	0
SLC2A5	solute carrier family 2 (facilitated glucose/fructose transporter), member 5	0,49	-1,04	0,06	0,39	0	-1,00	0,00	NA	NA	0	0,00	0,00	0
IGFBP5	insulin-like growth factor binding protein 5	0,48	-1,05	0,35	0,73	0	-1,01	-0,02	NA	NA	0	1,71	0,78	0
RARRES3	retinoic acid receptor responder (tazarotene induced) 3	0,48	-1,06	0,37	0,75	0	-1,01	-0,02	0,87	1,00	0	-1,16	-0,22	0
LAMP3	lysosomal-associated membrane protein 3	0,47	-1,09	0,04	0,32	0	-1,00	0,00	1,00	1,00	0	NA	NA	0
GCNT1	glucosaminyl (N-acetyl) transferase 1, core 2	0,47	-1,09	0,05	0,36	0	-1,03	-0,04	0,63	1,00	0	-1,15	-0,20	0
TMEM140	transmembrane protein 140	0,47	-1,10	0,09	0,45	0	-1,01	-0,01	0,99	1,00	0	-1,12	-0,16	0
EHD3	EH-domain containing 3	0,46	-1,11	0,01	0,21	0	-1,14	-0,18	0,41	1,00	0	0,00	0,01	0
ROR1	receptor tyrosine kinase-like orphan receptor 1	0,46	-1,13	0,02	0,27	0	-1,08	-0,12	0,65	1,00	0	-1,41	-0,49	0
CLMN	calmin (calponin-like, transmembrane)	0,44	-1,18	0,19	0,60	0	-1,25	-0,32	0,14	1,00	0	NA	NA	0
ROR1	receptor tyrosine kinase-like orphan receptor 1	0,44	-1,18	0,03	0,29	0	-1,10	-0,14	0,40	1,00	0	-1,49	-0,58	0
KRT15	keratin 15	0,44	-1,19	0,17	0,57	0	-1,61	-0,69	0,01	0,51	0	NA	NA	0
DKK1	dickkopf homolog 1 (Xenopus laevis)	0,43	-1,20	0,01	0,20	0	-1,14	-0,18	0,34	1,00	0	NA	NA	0
LIMCH1	LIM and calponin homology domains 1	0,42	-1,26	0,02	0,27	0	-1,24	-0,31	0,01	0,51	0	-1,19	-0,26	0
PLA2G16	phospholipase A2, group XVI	0,42	-1,26	0,08	0,44	0	-1,01	-0,01	NA	NA	0	1,04	0,06	0
APOL6	apolipoprotein L, 6	0,41	-1,28	0,03	0,29	0	NA	NA	NA	NA	0	NA	NA	0
TNNI2	troponin I type 2 (skeletal, fast)	0,39	-1,38	0,01	0,15	0	-1,55	-0,64	0,00	0,22	0	NA	NA	0
NA	NA	0,38	-1,40	0,00	0,13	0	NA	NA	NA	NA	0	NA	NA	0
CYP1B1	cytochrome P450, family 1, subfamily B, polypeptide 1	0,38	-1,40	0,04	0,32	0	-1,14	-0,19	0,66	1,00	0	NA	NA	0
CALML3	calmodulin-like 3	0,37	-1,43	0,01	0,17	0	-1,15	-0,20	0,62	1,00	0	NA	NA	0
IFI44	interferon-induced protein 44	0,37	-1,44	0,01	0,22	0	-1,02	-0,03	0,91	1,00	0	0,00	0,00	0
PLAC1	placenta-specific 1	0,36	-1,46	0,04	0,33	0	-1,07	-0,09	0,79	1,00	0	NA	NA	0
TLR3	toll-like receptor 3	0,36	-1,48	0,16	0,56	0	-1,03	-0,05	0,51	1,00	0	-1,03	-0,05	0
CYP1B1	cytochrome P450, family 1, subfamily B, polypeptide 1	0,35	-1,50	0,03	0,29	0	-1,63	-0,70	0,07	1,00	0	NA	NA	0
IL15	interleukin 15	0,35	-1,51	0,00	0,13	0	-1,01	-0,01	0,93	1,00	0	-1,09	-0,12	0
GPRC5B	G protein-coupled receptor, family C, group 5, member B	0,27	-1,87	0,01	0,16	0	-1,01	-0,01	0,94	1,00	0	-1,09	-0,13	0
PTX3	pentraxin 3, long	0,27	-1,87	0,01	0,15	0	-1,27	-0,35	0,54	1,00	0	1,15	0,20	0
B3GALNT1	beta-1,3-N-acetylgalactosaminyltransferase 1 (globoside blood group)	NA	NA	NA	NA	0	-1,12	-0,17	0,54	1,00	0	-1,61	-0,70	-1
IFIT2	interferon-induced protein with tetratricopeptide repeats 2	NA	NA	NA	NA	0	-1,01	-0,01	0,93	1,00	0	-3,03	-1,61	-1
KIF15	kinesin family member 15	NA	NA	NA	NA	0	-1,17	-0,22	0,58	1,00	0	-1,52	-0,59	-1
SLC4A4	solute carrier family 4, sodium bicarbonate cotransporter, member 4	NA	NA	NA	NA	0	-1,01	-0,01	NA	NA	0	-2,17	-1,12	-1
SH3GL2	SH3-domain GRB2-like 2	NA	NA	NA	NA	0	-1,01	-0,01	NA	NA	0	-1,72	-0,77	-1
FGF5	fibroblast growth factor 5	NA	NA	NA	NA	0	-1,00	0,00	NA	NA	0	-1,52	-0,61	-1
HFE	hemochromatosis	NA	NA	NA	NA	0	-1,05	-0,07	0,55	1,00	0	-1,79	-0,84	-1
SLC8A1	solute carrier family 8 (sodium/calcium exchanger), member 1	NA	NA	NA	NA	0	-1,01	-0,01	NA	NA	0	-1,79	-0,84	-1
RBMS3	RNA binding motif, single stranded interacting protein 3	NA	NA	NA	NA	0	-1,01	-0,02	NA	NA	0	-2,00	-1,00	-1
ESM1	endothelial cell-specific molecule 1	NA	NA	NA	NA	0	-1,01	-0,01	NA	NA	0	-1,72	-0,78	-1
PDE5A	phosphodiesterase 5A, cGMP-specific	NA	NA	NA	NA	0	-1,05	-0,07	NA	NA	0	-1,79	-0,84	-1
GMPR	guanosine monophosphate reductase	NA	NA	NA	NA	0	-1,01	-0,01	NA	NA	0	-1,56	-0,65	-1
DBT	dihydrolipoamide branched chain transacylase E2	NA	NA	NA	NA	0	-1,03	-0,04	0,95	1,00	0	-1,85	-0,90	-1
TRPC1	transient receptor potential cation channel, subfamily C, member 1	NA	NA	NA	NA	0	-1,02	-0,03	NA	NA	0	-2,04	-1,03	-1
NOX4	NADPH oxidase 4	NA	NA	NA	NA	0	-1,01	-0,01	NA	NA	0	-2,08	-1,04	-1
TOX	thymocyte selection-associated high mobility group box	NA	NA	NA	NA	0	-1,01	-0,01	NA	NA	0	-2,22	-1,14	-1
RGS2	regulator of G-protein signaling 2, 24kDa	2,04	1,03	0,01	0,20	0	-1,23	-0,30	0,29	1,00	0	-2,33	-1,21	-1
RGS4	regulator of G-protein signaling 4	1,84	0,88	0,12	0,51	0	-1,00	-0,01	NA	NA	0	-1,72	-0,77	-1
ARFIP1	ADP-ribosylation factor interacting protein 1	1,71	0,77	0,10	0,47	0	1,04	0,05	0,96	1,00	0	-1,79	-0,84	-1
SLC12A2	solute carrier family 12 (sodium/potassium/chloride transporters), member 2	1,61	0,69	0,05	0,35	0	1,21	0,28	0,25	1,00	0	-2,00	-1,01	-1
GRAMD1C	GRAM domain containing 1C	1,57	0,65	0,18	0,58	0	1,14	0,19	0,66	1,00	0	-1,75	-0,82	-1
NFE2L3	nuclear factor (erythroid-derived 2)-like 3	1,54	0,63	0,01	0,20	0	-1,16	-0,21	0,47	1,00	0	-2,08	-1,05	-1
RGS4	regulator of G-protein signaling 4	1,53	0,61	0,46	0,80	0	-1,00	-0,01	NA	NA	0	-1,52	-0,59	-1
GK	glycerol kinase	1,44	0,52	0,08	0,43	0	1,01	0,01	0,93	1,00	0	-1,52	-0,59	-1
PLAC8	placenta-specific 8	1,41	0,49	0,60	0,87	0	-1,01	-0,01	NA	NA	0	-1,52	-0,60	-1
HGF	hepatocyte growth factor (hepapoietin A; scatter factor)	1,33	0,41	0,27	0,67	0	-1,04	-0,06	0,63	1,00	0	-1,79	-0,84	-1
ITGA6	integrin, alpha 6	1,31	0,39	0,46	0,80	0	-1,39	-0,47	0,35	1,00	0	-1,52	-0,60	-1
PRKAG2	protein kinase, AMP-activated, gamma 2 non-catalytic subunit	1,28	0,35	0,23	0,64	0	1,77	0,82	0,34	1,00	0	-1,75	-0,81	-1
FLRT3	fibronectin leucine rich transmembrane protein 3	1,20	0,26	0,62	0,88	0	-1,32	-0,41	0,10	1,00	0	-2,08	-1,07	-1
TJP2	tight junction protein 2 (zona occludens 2)	1,17	0,23	0,31	0,70	0	1,03	0,04	0,80	1,00	0	-1,56	-0,64	-1
DLX2	distal-less homeobox 2	1,16	0,22	0,31	0,70	0	1,04	0,06	0,80	1,00	0	-1,72	-0,78	-1
ID4	inhibitor of DNA binding 4, dominant negative helix-loop-helix protein	1,16	0,22	0,59	0,87	0	-1,02	-0,03	NA	NA	0	-2,27	-1,20	-1
GK	glycerol kinase	1,16	0,21	0,53	0,83	0	-1,01	-0,02	0,96	1,00	0	-1,64	-0,71	-1
MALL	mal, T-cell differentiation protein-like	1,14	0,19	0,55	0,85	0	-1,06	-0,08	0,66	1,00	0	-2,44	-1,27	-1
IPO8	importin 8	1,12	0,16	0,52	0,83	0	-1,29	-0,37	0,33	1,00	0	-1,72	-0,78	-1
ETV1	ets variant 1	1,10	0,13	0,64	0,89	0	-1,03	-0,04	NA	NA	0	-1,67	-0,73	-1
XRCC4	X-ray repair complementing defective repair in Chinese hamster cells 4	1,10	0,13	0,52	0,83	0	1,22	0,28	0,53	1,00	0	-1,92	-0,95	-1
RNF121	ring finger protein 121	1,08	0,11	0,41	0,77	0	-1,04	-0,06	0,77	1,00	0	-1,89	-0,92	-1
BCLAF1	BCL2-associated transcription factor 1	1,08	0,11	0,66	0,89	0	-1,29	-0,36	0,72	1,00	0	-1,52	-0,59	-1
BHLHE40	basic helix-loop-helix family, member e40	1,08	0,11	0,79	0,94	0	-1,00	0,00	1,00	1,00	0	-1,72	-0,78	-1
MRPS31	mitochondrial ribosomal protein S31	1,08	0,11	0,65	0,89	0	-1,03	-0,05	0,80	1,00	0	-1,61	-0,69	-1
EXOG	endo/exonuclease (5'-3'), endonuclease G-like	1,06	0,09	0,68	0,90	0	1,15	0,20	0,53	1,00	0	-1,54	-0,62	-1
SP100	SP100 nuclear antigen	1,06	0,09	0,74	0,92	0	1,06	0,09	0,82	1,00	0	-1,92	-0,95	-1
COBLL1	COBL-like 1	1,04	0,06	0,77	0,94	0	1,25	0,32	0,64	1,00	0	-2,08	-1,07	-1
ENPP1	ectonucleotide pyrophosphatase/phosphodiesterase 1	1,04	0,06	0,89	0,97	0	-1,01	-0,02	NA	NA	0	-1,56	-0,65	-1
PPAP2B	phosphatidic acid phosphatase type 2B	1,04	0,06	0,87	0,97	0	-1,01	-0,01	NA	NA	0	-3,03	-1,60	-1
CREBL2	cAMP responsive element binding protein-like 2	1,03	0,05	0,83	0,95	0	1,08	0,11	0,55	1,00	0	-1,61	-0,69	-1
NFKBIE	nuclear factor of kappa light polypeptide gene enhancer in B-cells inhibitor, epsilon	1,03	0,04	0,80	0,94	0	-1,15	-0,21	0,18	1,00	0	-1,64	-0,71	-1
MEST	mesoderm specific transcript homolog (mouse)	1,03	0,04	0,91	0,98	0	-1,14	-0,18	0,28	1,00	0	-2,50	-1,34	-1
PIK3R1	phosphoinositide-3-kinase, regulatory subunit 1 (alpha)	1,02	0,03	0,86	0,96	0	-1,19	-0,25	0,34	1,00	0	-2,38	-1,25	-1
TRIB2	tribbles homolog 2 (Drosophila)	1,00	0,00	0,99	1,00	0	-1,62	-0,70	0,21	1,00	0	-2,08	-1,05	-1
RAD51C	RAD51 homolog C (S. cerevisiae)	0,99	-0,01	0,95	0,99	0	-1,07	-0,09	0,78	1,00	0	-1,59	-0,66	-1
IFI16	interferon, gamma-inducible protein 16	0,99	-0,01	0,95	0,99	0	-1,02	-0,03	0,81	1,00	0	-1,54	-0,61	-1
PUS3	pseudouridylate synthase 3	0,98	-0,02	0,86	0,96	0	1,22	0,28	0,20	1,00	0	-2,08	-1,06	-1
ALKBH4	alkB, alkylation repair homolog 4 (E. coli)	0,98	-0,03	0,88	0,97	0	1,11	0,15	0,25	1,00	0	-1,59	-0,66	-1

OSBPL10	oxysterol binding protein-like 10	0,98	-0,03	0,91	0,98	0	-1,12	-0,17	0,09	1,00	0	-1,52	-0,61	-1
SEMA3A	sema domain, immunoglobulin domain (Ig), short basic domain, secreted, (semaphorin) 3A	0,97	-0,04	0,95	0,99	0	-1,01	-0,01	NA	NA	0	-1,89	-0,91	-1
NUDT11	nudix (nucleoside diphosphate linked moiety X)-type motif 11	0,97	-0,04	0,86	0,97	0	-1,34	-0,42	0,10	1,00	0	-1,52	-0,59	-1
PSD3	pleckstrin and Sec7 domain containing 3	0,96	-0,06	0,71	0,91	0	-1,16	-0,22	0,40	1,00	0	-1,64	-0,72	-1
F2RL1	coagulation factor II (thrombin) receptor-like 1	0,96	-0,06	0,74	0,92	0	-1,09	-0,13	0,46	1,00	0	-1,92	-0,94	-1
EPB41L3	erythrocyte membrane protein band 4.1-like 3	0,95	-0,08	0,82	0,95	0	-1,04	-0,06	0,55	1,00	0	-1,52	-0,61	-1
GPR137B	G protein-coupled receptor 137B	0,94	-0,08	0,58	0,86	0	-1,17	-0,23	0,36	1,00	0	-1,52	-0,60	-1
DCN	decorin	0,93	-0,10	0,66	0,89	0	-1,01	-0,02	NA	NA	0	-1,52	-0,61	-1
LBH	limb bud and heart development homolog (mouse)	0,93	-0,11	0,62	0,88	0	1,63	0,71	0,13	1,00	0	-3,03	-1,59	-1
SEL1L3	sel-1 suppressor of lin-12-like 3 (C. elegans)	0,92	-0,11	0,58	0,86	0	-1,19	-0,25	0,06	1,00	0	-1,59	-0,66	-1
PKIA	protein kinase (cAMP-dependent, catalytic) inhibitor alpha	0,92	-0,12	0,62	0,88	0	1,11	0,15	0,68	1,00	0	-1,92	-0,95	-1
SAMD9	sterile alpha motif domain containing 9	0,90	-0,15	0,79	0,94	0	1,00	0,01	0,98	1,00	0	-1,72	-0,79	-1
ALDH7A1	aldehyde dehydrogenase 7 family, member A1	0,90	-0,15	0,52	0,83	0	-1,21	-0,27	0,13	1,00	0	-1,59	-0,66	-1
C2orf3	chromosome 2 open reading frame 3	0,90	-0,15	0,48	0,81	0	-1,04	-0,05	0,79	1,00	0	-1,64	-0,70	-1
NR2F1	nuclear receptor subfamily 2, group F, member 1	0,90	-0,16	0,39	0,76	0	-1,00	0,00	NA	NA	0	-2,04	-1,03	-1
OLFML2B	olfactomedin-like 2B	0,89	-0,16	0,39	0,76	0	1,00	0,01	NA	NA	0	-2,04	-1,03	-1
STK17B	serine/threonine kinase 17b	0,89	-0,17	0,66	0,89	0	-1,17	-0,23	0,60	1,00	0	-1,79	-0,84	-1
CNN3	calponin 3, acidic	0,88	-0,19	0,44	0,78	0	1,35	0,43	0,41	1,00	0	-1,75	-0,81	-1
GEM	GTP binding protein overexpressed in skeletal muscle	0,87	-0,20	0,65	0,89	0	1,24	0,31	0,22	1,00	0	-1,82	-0,85	-1
SSX2IP	synovial sarcoma, X breakpoint 2 interacting protein	0,87	-0,20	0,32	0,71	0	-1,25	-0,32	0,08	1,00	0	-1,52	-0,59	-1
MITF	microphthalmia-associated transcription factor	0,86	-0,21	0,24	0,65	0	-1,18	-0,24	0,14	1,00	0	-1,69	-0,76	-1
RARRES1	retinoic acid receptor responder (tazarotene induced) 1	0,86	-0,21	0,68	0,90	0	-1,01	-0,01	0,99	1,00	0	-1,56	-0,65	-1
FAM65B	family with sequence similarity 65, member B	0,86	-0,21	0,64	0,89	0	-1,01	-0,01	NA	NA	0	-1,85	-0,89	-1
OAS3	2'-5'-oligoadenylate synthetase 3, 100kDa	0,86	-0,21	0,66	0,89	0	-1,23	-0,30	0,10	1,00	0	-2,22	-1,14	-1
PTN	pleiotrophin	0,86	-0,22	0,45	0,79	0	-1,00	0,00	NA	NA	0	-1,67	-0,74	-1
TLE1	transducin-like enhancer of split 1 (E(sp1) homolog, Drosophila)	0,86	-0,22	0,19	0,60	0	1,01	0,01	0,94	1,00	0	-1,56	-0,64	-1
PTN	pleiotrophin	0,86	-0,22	0,49	0,81	0	-1,02	-0,03	NA	NA	0	-1,92	-0,93	-1
NA	NA	0,85	-0,23	0,39	0,75	0	1,03	0,04	0,81	1,00	0	-1,79	-0,85	-1
SRI	sorcin	0,84	-0,26	0,35	0,73	0	1,07	0,10	0,51	1,00	0	-1,59	-0,66	-1
MR1	major histocompatibility complex, class I-related	0,82	-0,29	0,23	0,64	0	-1,09	-0,12	0,48	1,00	0	-1,67	-0,75	-1
LOC81691	exonuclease NEF-sp	0,82	-0,29	0,08	0,43	0	-1,16	-0,22	0,22	1,00	0	-1,89	-0,90	-1
TRIM14	tripartite motif-containing 14	0,81	-0,30	0,52	0,83	0	1,05	0,07	0,67	1,00	0	-1,56	-0,64	-1
EIF2AK2	eukaryotic translation initiation factor 2-alpha kinase 2	0,81	-0,30	0,25	0,65	0	1,01	0,02	0,93	1,00	0	-1,75	-0,81	-1
TARDBP	TAR DNA binding protein	0,81	-0,31	0,33	0,71	0	-1,03	-0,05	0,93	1,00	0	-2,13	-1,09	-1
TCP11L1	t-complex 11 (mouse)-like 1	0,80	-0,32	0,04	0,34	0	-1,04	-0,06	0,85	1,00	0	-1,59	-0,67	-1
DUS4L	dihydrouridine synthase 4-like (S. cerevisiae)	0,80	-0,33	0,03	0,30	0	-1,06	-0,09	0,61	1,00	0	-1,61	-0,68	-1
OSBPL1A	oxysterol binding protein-like 1A	0,79	-0,34	0,07	0,41	0	1,15	0,20	0,72	1,00	0	-1,64	-0,72	-1
FAM115A	family with sequence similarity 115, member A	0,78	-0,35	0,17	0,58	0	-1,17	-0,22	0,20	1,00	0	-1,49	-0,58	-1
PTN	pleiotrophin	0,78	-0,37	0,29	0,69	0	-1,01	-0,01	NA	NA	0	-2,00	-1,00	-1
BCKDHB	branched chain keto acid dehydrogenase E1, beta polypeptide	0,78	-0,37	0,08	0,43	0	-1,54	-0,62	0,15	1,00	0	-2,27	-1,20	-1
SSX2IP	synovial sarcoma, X breakpoint 2 interacting protein	0,77	-0,37	0,05	0,37	0	1,03	0,04	NA	NA	0	-1,85	-0,88	-1
STAT1	signal transducer and activator of transcription 1, 91kDa	0,77	-0,37	0,23	0,63	0	-1,13	-0,18	0,59	1,00	0	-1,61	-0,70	-1
PLSCR1	phospholipid scramblase 1	0,77	-0,37	0,12	0,51	0	-1,06	-0,09	0,61	1,00	0	-1,75	-0,81	-1
SCARB2	scavenger receptor class B, member 2	0,77	-0,37	0,06	0,39	0	-1,02	-0,02	0,95	1,00	0	-1,64	-0,71	-1
LPAR6	lysophosphatidic acid receptor 6	0,77	-0,37	0,23	0,63	0	-1,12	-0,17	0,38	1,00	0	-2,27	-1,19	-1
ATP7A	ATPase, Cu++ transporting, alpha polypeptide	0,77	-0,38	0,07	0,40	0	-1,02	-0,03	0,90	1,00	0	-1,61	-0,70	-1
AGTPBP1	ATP/GTP binding protein 1	0,77	-0,38	0,06	0,39	0	-1,14	-0,19	0,43	1,00	0	-1,79	-0,82	-1
TRIM38	tripartite motif-containing 38	0,76	-0,39	0,12	0,51	0	-1,21	-0,27	0,10	1,00	0	-1,54	-0,62	-1
SFRP1	secreted frizzled-related protein 1	0,75	-0,42	0,29	0,69	0	-1,31	-0,39	0,19	1,00	0	-2,78	-1,48	-1
FAM115A	family with sequence similarity 115, member A	0,75	-0,42	0,29	0,69	0	-1,31	-0,39	0,18	1,00	0	-3,70	-1,88	-1
EFEMP1	EGF-containing fibulin-like extracellular matrix protein 1	0,75	-0,42	0,20	0,61	0	-1,32	-0,40	0,26	1,00	0	-1,52	-0,60	-1
CFH	complement factor H	0,75	-0,42	0,19	0,59	0	-1,01	-0,01	0,97	1,00	0	-1,52	-0,61	-1
C13orf15	chromosome 13 open reading frame 15	0,74	-0,44	0,35	0,73	0	-1,02	-0,03	NA	NA	0	-1,75	-0,81	-1
GPD1L	glycerol-3-phosphate dehydrogenase 1-like	0,73	-0,44	0,03	0,29	0	1,09	0,13	0,68	1,00	0	-1,82	-0,85	-1
IFITM1	interferon induced transmembrane protein 1 (9-27)	0,73	-0,45	0,09	0,44	0	-1,38	-0,47	0,28	1,00	0	-1,64	-0,70	-1
LOC730101	hypothetical LOC730101	0,73	-0,45	0,20	0,61	0	-1,01	-0,01	NA	NA	0	-1,64	-0,72	-1
RGS4	regulator of G-protein signaling 4	0,73	-0,45	0,69	0,90	0	-1,01	-0,01	NA	NA	0	-1,54	-0,62	-1
PROS1	protein S (alpha)	0,72	-0,47	0,03	0,30	0	1,01	0,01	0,97	1,00	0	-1,52	-0,60	-1
SFRP1	secreted frizzled-related protein 1	0,72	-0,48	0,06	0,39	0	-1,16	-0,21	0,13	1,00	0	-1,56	-0,64	-1
ATP10A	ATPase, class V, type 10A	0,72	-0,48	0,19	0,60	0	-1,01	-0,01	NA	NA	0	-1,54	-0,62	-1
TP53TG1	TP53 target 1 (non-protein coding)	0,72	-0,48	0,03	0,29	0	-1,24	-0,31	0,14	1,00	0	-1,72	-0,79	-1
PBX3	pre-B-cell leukemia homeobox 3	0,70	-0,52	0,02	0,27	0	1,02	0,03	0,75	1,00	0	-1,52	-0,60	-1
LRRC17	leucine rich repeat containing 17	0,69	-0,53	0,08	0,43	0	-1,01	-0,01	NA	NA	0	-2,38	-1,26	-1
ZC3HAV1	zinc finger CCCH-type, antiviral 1	0,69	-0,54	0,16	0,56	0	1,04	0,05	0,86	1,00	0	-1,64	-0,70	-1
SLC24A1	solute carrier family 24 (sodium/potassium/calcium exchanger), member 1	0,69	-0,54	0,03	0,28	0	1,12	0,17	0,55	1,00	0	-2,00	-1,01	-1
PLSCR1	phospholipid scramblase 1	0,68	-0,55	0,06	0,38	0	-1,04	-0,05	0,78	1,00	0	-1,59	-0,67	-1
SP110	SP110 nuclear body protein	0,67	-0,59	0,22	0,63	0	-1,00	0,00	1,00	1,00	0	-1,56	-0,66	-1
DDX58	DEAD (Asp-Glu-Ala-Asp) box polypeptide 58	0,66	-0,59	0,15	0,55	0	-1,09	-0,12	0,74	1,00	0	-2,04	-1,04	-1
XAF1	XIAP associated factor 1	0,66	-0,60	0,33	0,71	0	-1,00	-0,01	0,99	1,00	0	-2,56	-1,37	-1
FAM65B	family with sequence similarity 65, member B	0,65	-0,61	0,18	0,58	0	-1,01	-0,01	NA	NA	0	-1,92	-0,94	-1
EFEMP1	EGF-containing fibulin-like extracellular matrix protein 1	0,65	-0,62	0,12	0,50	0	-1,51	-0,59	0,32	1,00	0	-1,69	-0,77	-1
IFIT3	interferon-induced protein with tetratricopeptide repeats 3	0,65	-0,63	0,22	0,63	0	-1,43	-0,52	0,02	0,94	0	-2,00	-1,00	-1
LDB2	LIM domain binding 2	0,63	-0,66	0,12	0,50	0	-1,02	-0,02	NA	NA	0	-1,64	-0,72	-1
SNCAIP	synuclein, alpha interacting protein	0,63	-0,67	0,08	0,43	0	-1,01	-0,01	NA	NA	0	-1,82	-0,86	-1
MBNL2	muscleblind-like 2 (Drosophila)	0,62	-0,68	0,09	0,45	0	-1,90	-0,92	0,21	1,00	0	-1,79	-0,82	-1
PSMB9	proteasome (prosome, macropain) subunit, beta type, 9 (large multifunctional peptidase 2)	0,62	-0,69	0,04	0,32	0	-1,36	-0,44	0,22	1,00	0	-1,59	-0,68	-1
SNCA	synuclein, alpha (non A4 component of amyloid precursor)	0,61	-0,71	0,04	0,32	0	-1,00	0,00	1,00	1,00	0	-2,33	-1,22	-1
SP110	SP110 nuclear body protein	0,60	-0,75	0,19	0,59	0	-1,15	-0,20	0,49	1,00	0	-1,61	-0,70	-1
COLEC12	collectin sub-family member 12	0,59	-0,76	0,05	0,36	0	-1,01	-0,01	NA	NA	0	-3,70	-1,87	-1
IFIH1	interferon induced with helicase C domain 1	0,59	-0,76	0,03	0,30	0	-1,36	-0,44	0,07	1,00	0	-2,27	-1,18	-1
EPHA4	EPH receptor A4	0,59	-0,77	0,09	0,45	0	-1,00	0,00	NA	NA	0	-2,17	-1,12	-1
USP18	ubiquitin specific peptidase 18	0,58	-0,77	0,07	0,42	0	-1,02	-0,03	0,96	1,00	0	-2,27	-1,19	-1
COL11A1	collagen, type XI, alpha 1	0,58	-0,78	0,03	0,29	0	-1,01	-0,01	NA	NA	0	-1,64	-0,71	-1
COL11A1	collagen, type XI, alpha 1	0,57	-0,82	0,08	0,43	0	-1,01	-0,01	NA	NA	0	-1,72	-0,78	-1
C5orf23	chromosome 5 open reading frame 23	0,56	-0,84	0,32	0,71	0	-1,01	-0,01	NA	NA	0	-1,79	-0,84	-1
CASP6	caspase 6, apoptosis-related cysteine peptidase	0,56	-0,84	0,21	0,62	0	-1,06	-0,08	0,79	1,00	0	-1,67	-0,75	-1
OAS1	2',5'-oligoadenylate synthetase 1, 40/46kDa	0,55	-0,85	0,13	0,53	0	-1,14	-0,18	0,51	1,00	0	-2,33	-1,22	-1
TXNIP	thioredoxin interacting protein	0,55	-0,86	0,51	0,83	0	-1,75	-0,80	0,02	0,94	0	-1,82	-0,87	-1
IFI44	interferon-induced protein 44	0,54	-0,88	0,04	0,32	0	-1,31	-0,39	0,51	1,00	0	-2,38	-1,26	-1
HERC6	hect domain and RLD 6	0,54	-0,89	0,24	0,64	0	-1,10	-0,14	0,57	1,00	0	-2,56	-1,36	-1
OAS2	2'-5'-oligoadenylate synthetase 2, 69/71kDa	0,54	-0,89	0,17	0,57	0	-1,78	-0,83	0,08	1,00	0	-2,86	-1,53	-1
ISG15	ISG15 ubiquitin-like modifier	0,53	-0,92	0,11	0,49	0	-1,13	-0,18	0,66	1,00	0	-1,59	-0,66	-1
SP110	SP110 nuclear body protein	0,52	-0,93	0,09	0,44	0	1,02	0,03	0,95	1,00	0	-2,17	-1,12	-1
TXNIP	thioredoxin interacting protein	0,51	-0,96	0,39	0,76	0	-1,59	-0,67	0,00	0,35	0	-2,17	-1,12	-1
TPD52L1	tumor protein D52-like 1	0,50	-1,00	0,03	0,29	0	1,07	0,09	0,71	1,00	0	-1,52	-0,60	-1
SEPP1	selenoprotein P, plasma, 1	0,48	-1,04	0,02	0,22	0	-1,22	-0,29	0,67	1,00	0	-3,33	-1,72	-1
LXN	latexin	0,48	-1,05	0,03	0,29	0	-1,46	-0,54	0,01	0,70	0	-1,56	-0,64	-1
MX1	myxovirus (influenza virus) resistance 1, interferon-inducible protein p78 (mouse)	0,48	-1,05	0,16	0,56	0	-1,17	-0,22	0,54	1,00	0	-2,17	-1,13	-1
SCN9A	sodium channel, voltage-gated, type IX, alpha subunit	0,46	-1,12	0,06	0,39	0	-1,01	-0,02	NA	NA	0	-1,79	-0,85	-1
GATA6	GATA binding protein 6	0,43	-1,21	0,01	0,17	0	-1,01	-0,02	NA	NA	0	-2,17	-1,10	-1
MX2	myxovirus (influenza virus) resistance 2 (mouse)	0,41	-1,29	0,06	0,40	0	-1,00	0,00	0,99	1,00	0	-2,44	-1,29	-1
SLC38A4	solute carrier family 38, member 4	0,40	-1,33	0,01	0,21	0	-1,02	-0,02	NA	NA	0	-3,03	-1,60	-1
IFI44L	interferon-induced protein 44-like	0,37	-1,43	0,06	0,39	0	-1,56	-0,64	0,51	1,00	0	-5,26	-2,39	-1
IFIT1	interferon-induced protein with tetratricopeptide repeats 1	0,28	-1,85	0,07	0,41	0	-1,62	-0,70	0,19	1,00	0	-2,04	-1,03	-1
CXCL12	chemokine (C-X-C motif) ligand 12	0,22	-2,19	0,02	0,23	0	-1,01	-0,01	NA	NA	0	-2,33	-1,22	-1
MMP9	matrix metallopeptidase 9 (gelatinase B, 92kDa gelatinase, 92kDa type IV collagenase)	0,79	-0,34	0,54	0,84	0	-10,43	-3,38	0,00	0,04	-1	NA	NA	0
FLOT1	flotillin 1	0,78	-0,36	0,14	0,54	0	-1,59	-0,67	0,00	0,07	-1	NA	NA	0
FLOT1	flotillin 1	0,76	-0,39	0,07	0,41	0	-1,52	-0,60	0,00	0,16	-1	NA	NA	0
ACTA2	actin, alpha 2, smooth muscle, aorta	0,73	-0,45	0,09	0,45	0	-1,67	-0,74	0,00	0,11	-1	1,04	0,05	0
FUBP1	far upstream element (FUSE) binding protein 1	0,61	-0,72	0,04	0,34	0	-1,77	-0,82	0,00	0,11	-1	-1,03	-0,04	0
LOR	loricrin	0,33	-1,60	0,02	0,26	0	-6,14	-2,62	0,00	0,09	-1	NA	NA	0
GKAP1	G kinase anchoring protein 1	0,65	-0,62	0,00	0,04	-1	NA	NA	NA	NA	0	NA	NA	0
NOL3	nucleolar protein 3 (apoptosis repressor with CARD domain)	0,65	-0,63	0,00	0,09	-1	-1,11	-0,15	0,49	1,00	0	NA	NA	0
RBPMS	RNA binding protein with multiple splicing	0,64	-0,63	0,00	0,06	-1	1,12	0,16	0,11	1,00	0	NA	NA	0
C8orf48	chromosome 8 open reading frame 48	0,64	-0,63	0,00	0,07	-1	NA	NA	NA	NA	0	NA	NA	0
GSTM1	glutathione S-transferase mu 1	0,64	-0,64	0,00	0,09	-1	-1,19	-0,25	0,13	1,00	0	NA	NA	0
LYPLAL1	lysophospholipase-like 1	0,64	-0,65	0,00	0,09	-1	NA	NA	NA	NA	0	NA	NA	0
UPK1A	uroplakin 1A	0,64	-0,65	0,00	0,03	-1	-1,00	0,00	NA	NA	0	NA	NA	0
SRGAP2P1	SLIT-ROBO Rho GTPase activating protein 2 pseudogene 1	0,63	-0,66	0,00	0,08	-1	NA	NA	NA	NA	0	NA	NA	0
NA	NA	0,63	-0,66	0,00	0,06	-1	1,01	0,02	NA	NA	0	NA	NA	0
NA	NA	0,63	-0,67	0,00	0,05	-1	-1,27	-0,34	0,13	1,00	0	NA	NA	0
LOC439938	hypothetical LOC439938	0,63	-0,68	0,00	0,10	-1	NA	NA	NA	NA	0	NA	NA	0
SRGAP2P1	SLIT-ROBO Rho GTPase activating protein 2 pseudogene 1	0,63	-0,68	0,00	0,06	-1	NA	NA	NA	NA	0	NA	NA	0
ZMYM3	zinc finger, MYM-type 3	0,62	-0,68	0,00	0,10	-1	NA	NA	NA	NA	0	NA	NA	0
NOL3	nucleolar protein 3 (apoptosis repressor with CARD domain)	0,62	-0,68	0,00	0,05	-1	-1,00	0,00	0,99	1,00	0	NA	NA	0
NPHP3	nephronophthisis 3 (adolescent)	0,62	-0,69	0,00	0,06	-1	NA	NA	NA	NA	0	NA	NA	0
SGK3	serum/glucocorticoid regulated kinase family, member 3	0,62	-0,70	0,00	0,03	-1	NA	NA	NA	NA	0	NA	NA	0
PRKAB2	protein kinase, AMP-activated, beta 2 non-catalytic subunit	0,62	-0,70	0,00	0,05	-1	NA	NA	NA	NA	0	NA	NA	0
NA	NA	0,61	-0,71	0,00	0,06	-1	NA	NA	NA	NA	0	NA	NA	0
RBPMS	RNA binding protein with multiple splicing	0,61	-0,71	0,00	0,09	-1	-1,09	-0,13	0,27	1,00	0	NA	NA	0
PRKAB2	protein kinase, AMP-activated, beta 2 non-catalytic subunit	0,61	-0,71	0,00	0,04	-1	NA	NA	NA	NA	0	NA	NA	0
ARHGAP33	Rho GTPase activating protein 33	0,61	-0,72	0,00	0,07	-1	-1,04	-0,06	NA	NA	0	NA	NA	0
NA	NA	0,60	-0,73	0,00	0,07	-1	NA	NA	NA	NA	0	NA	NA	0
NA	NA	0,60	-0,73	0,00	0,07	-1	-1,01	-0,01	NA	NA	0	NA	NA	0
KRT10	keratin 10	0,60	-0,73	0,00	0,06	-1	-1,47	-0,56	0,00	0,17	0	-1,04	-0,06	0
NA	NA	0,60	-0,73	0,00	0,10	-1	NA	NA	NA	NA	0	NA	NA	0
IL20RA	interleukin 20 receptor, alpha	0,60	-0,73	0,00	0,06	-1	-1,65	-0,72	0,14	1,00	0	NA	NA	0

DDB2	damage-specific DNA binding protein 2, 48kDa	0,60	-0,74	0,00	0,09	-1	-1,33	-0,41	0,00	0,46	0	NA	NA	0
SENP7	SUMO1/sentrin specific peptidase 7	0,60	-0,74	0,00	0,05	-1	NA	NA	NA	NA	0	NA	NA	0
C1orf21	chromosome 1 open reading frame 21	0,60	-0,74	0,00	0,08	-1	NA	NA	NA	NA	0	NA	NA	0
NAT1	N-acetyltransferase 1 (arylamine N-acetyltransferase)	0,60	-0,74	0,00	0,06	-1	1,04	0,05	0,72	1,00	0	NA	NA	0
NA	NA	0,60	-0,75	0,00	0,08	-1	NA	NA	NA	NA	0	NA	NA	0
TRADD	TNFRSF1A-associated via death domain	0,59	-0,75	0,00	0,08	-1	-1,18	-0,23	0,15	1,00	0	NA	NA	0
TP53TG1	TP53 target 1 (non-protein coding)	0,59	-0,76	0,00	0,04	-1	-1,31	-0,39	0,03	1,00	0	NA	NA	0
LIAS	lipoic acid synthetase	0,59	-0,76	0,00	0,09	-1	1,08	0,11	0,86	1,00	0	NA	NA	0
SSX2IP	synovial sarcoma, X breakpoint 2 interacting protein	0,59	-0,76	0,00	0,02	-1	-1,10	-0,14	NA	NA	0	-2,00	-0,99	0
PIR	pirin (iron-binding nuclear protein)	0,59	-0,77	0,00	0,06	-1	-1,19	-0,25	0,21	1,00	0	NA	NA	0
NA	NA	0,59	-0,77	0,00	0,09	-1	NA	NA	NA	NA	0	NA	NA	0
PRSS23	protease, serine, 23	0,58	-0,78	0,00	0,09	-1	NA	NA	NA	NA	0	NA	NA	0
IQCH	IQ motif containing H	0,58	-0,78	0,00	0,10	-1	NA	NA	NA	NA	0	NA	NA	0
ARHGAP26	Rho GTPase activating protein 26	0,58	-0,79	0,00	0,04	-1	-1,00	-0,01	NA	NA	0	NA	NA	0
PGAP3	post-GPI attachment to proteins 3	0,58	-0,79	0,00	0,07	-1	1,09	0,13	0,21	1,00	0	NA	NA	0
CSRNP3	cysteine-serine-rich nuclear protein 3	0,58	-0,79	0,00	0,09	-1	NA	NA	NA	NA	0	NA	NA	0
HSPA2	heat shock 70kDa protein 2	0,58	-0,80	0,00	0,04	-1	-1,16	-0,21	0,08	1,00	0	-1,19	-0,26	0
ZNF404	zinc finger protein 404	0,58	-0,80	0,00	0,06	-1	NA	NA	NA	NA	0	NA	NA	0
NA	NA	0,57	-0,80	0,00	0,01	-1	NA	NA	NA	NA	0	NA	NA	0
SLC25A27	solute carrier family 25, member 27	0,57	-0,80	0,00	0,05	-1	NA	NA	NA	NA	0	NA	NA	0
NA	NA	0,57	-0,80	0,00	0,04	-1	NA	NA	NA	NA	0	NA	NA	0
ZNF280D	zinc finger protein 280D	0,57	-0,80	0,00	0,04	-1	-1,18	-0,24	0,27	1,00	0	NA	NA	0
RAB3IP	RAB3A interacting protein (rabin3)	0,57	-0,81	0,00	0,04	-1	NA	NA	NA	NA	0	NA	NA	0
NHLRC3	NHL repeat containing 3	0,57	-0,81	0,00	0,06	-1	NA	NA	NA	NA	0	NA	NA	0
PRKAB2	protein kinase, AMP-activated, beta 2 non-catalytic subunit	0,57	-0,81	0,00	0,05	-1	-1,13	-0,18	0,09	1,00	0	NA	NA	0
ELMOD2	ELMO/CED-12 domain containing 2	0,57	-0,82	0,00	0,10	-1	NA	NA	NA	NA	0	NA	NA	0
KRT10	keratin 10	0,57	-0,82	0,00	0,05	-1	-1,29	-0,37	0,00	0,29	0	NA	NA	0
NA	NA	0,57	-0,82	0,00	0,04	-1	NA	NA	NA	NA	0	NA	NA	0
RAB40B	RAB40B, member RAS oncogene family	0,57	-0,82	0,00	0,09	-1	-1,22	-0,29	0,11	1,00	0	-1,27	-0,34	0
TPD52L1	tumor protein D52-like 1	0,57	-0,82	0,00	0,06	-1	-1,00	0,00	0,99	1,00	0	-1,25	-0,33	0
ZFP112	zinc finger protein 112 homolog (mouse)	0,56	-0,83	0,00	0,10	-1	-1,22	-0,29	0,40	1,00	0	NA	NA	0
ZNF193	zinc finger protein 193	0,56	-0,83	0,00	0,07	-1	-1,03	-0,04	0,70	1,00	0	NA	NA	0
NA	NA	0,56	-0,83	0,00	0,08	-1	NA	NA	NA	NA	0	NA	NA	0
FAM83C	family with sequence similarity 83, member C	0,56	-0,83	0,00	0,01	-1	NA	NA	NA	NA	0	NA	NA	0
TMEM63A	transmembrane protein 63A	0,56	-0,83	0,00	0,08	-1	NA	NA	NA	NA	0	NA	NA	0
FLJ45244	hypothetical locus FLJ45244	0,56	-0,84	0,00	0,05	-1	NA	NA	NA	NA	0	NA	NA	0
SCAI	suppressor of cancer cell invasion	0,56	-0,84	0,00	0,09	-1	NA	NA	NA	NA	0	NA	NA	0
ASAP3	ArfGAP with SH3 domain, ankyrin repeat and PH domain 3	0,55	-0,86	0,00	0,07	-1	-1,36	-0,44	0,01	0,76	0	1,03	0,05	0
LOC285147	hypothetical protein LOC285147	0,55	-0,86	0,00	0,09	-1	NA	NA	NA	NA	0	NA	NA	0
GCA	grancalcin, EF-hand calcium binding protein	0,55	-0,86	0,00	0,07	-1	-1,06	-0,09	0,78	1,00	0	-1,16	-0,21	0
LOC100287896	similar to cag	0,55	-0,86	0,00	0,09	-1	NA	NA	NA	NA	0	NA	NA	0
KLHL24	kelch-like 24 (Drosophila)	0,55	-0,86	0,00	0,09	-1	1,18	0,23	0,27	1,00	0	NA	NA	0
NA	NA	0,55	-0,87	0,00	0,03	-1	NA	NA	NA	NA	0	NA	NA	0
TRIM69	tripartite motif-containing 69	0,54	-0,88	0,00	0,07	-1	NA	NA	NA	NA	0	NA	NA	0
ZNF846	zinc finger protein 846	0,54	-0,88	0,00	0,02	-1	NA	NA	NA	NA	0	NA	NA	0
PRKAA2	protein kinase, AMP-activated, alpha 2 catalytic subunit	0,54	-0,88	0,00	0,09	-1	NA	NA	NA	NA	0	NA	NA	0
LOC100289187	hypothetical protein LOC100289187	0,54	-0,90	0,00	0,04	-1	NA	NA	NA	NA	0	NA	NA	0
NA	NA	0,53	-0,91	0,00	0,10	-1	NA	NA	NA	NA	0	NA	NA	0
MANEAL	mannosidase, endo-alpha-like	0,53	-0,91	0,00	0,04	-1	NA	NA	NA	NA	0	NA	NA	0
NHLRC3	NHL repeat containing 3	0,53	-0,91	0,00	0,08	-1	NA	NA	NA	NA	0	NA	NA	0
NUDT7	nudix (nucleoside diphosphate linked moiety X)-type motif 7	0,53	-0,92	0,00	0,03	-1	NA	NA	NA	NA	0	NA	NA	0
MIAT	myocardial infarction associated transcript (non-protein coding)	0,53	-0,93	0,00	0,10	-1	NA	NA	NA	NA	0	NA	NA	0
NA	NA	0,53	-0,93	0,00	0,10	-1	NA	NA	NA	NA	0	NA	NA	0
LOC284440	hypothetical LOC284440	0,53	-0,93	0,00	0,04	-1	NA	NA	NA	NA	0	NA	NA	0
TP53INP1	tumor protein p53 inducible nuclear protein 1	0,52	-0,93	0,00	0,08	-1	NA	NA	NA	NA	0	NA	NA	0
DZIP3	DAZ interacting protein 3, zinc finger	0,52	-0,94	0,00	0,04	-1	-1,05	-0,06	0,71	1,00	0	NA	NA	0
TSHZ2	teashirt zinc finger homeobox 2	0,52	-0,94	0,00	0,07	-1	-1,00	-0,01	NA	NA	0	NA	NA	0
KRT1	keratin 1	0,52	-0,95	0,00	0,03	-1	-1,22	-0,28	0,32	1,00	0	NA	NA	0
FAM82B	family with sequence similarity 82, member B	0,52	-0,95	0,00	0,07	-1	NA	NA	NA	NA	0	NA	NA	0
TMEM116	transmembrane protein 116	0,52	-0,95	0,00	0,04	-1	NA	NA	NA	NA	0	NA	NA	0
PSMB2	proteasome (prosome, macropain) subunit, beta type, 2	0,52	-0,95	0,00	0,04	-1	NA	NA	NA	NA	0	NA	NA	0
TMEM91	transmembrane protein 91	0,52	-0,95	0,00	0,03	-1	NA	NA	NA	NA	0	NA	NA	0
NA	NA	0,52	-0,95	0,00	0,10	-1	NA	NA	NA	NA	0	NA	NA	0
RAB7B	RAB7B, member RAS oncogene family	0,52	-0,96	0,00	0,06	-1	NA	NA	NA	NA	0	NA	NA	0
LOC253039	hypothetical LOC253039	0,51	-0,97	0,00	0,03	-1	NA	NA	NA	NA	0	NA	NA	0
NA	NA	0,51	-0,97	0,00	0,01	-1	NA	NA	NA	NA	0	NA	NA	0
PRSS23	protease, serine, 23	0,51	-0,98	0,00	0,05	-1	-1,24	-0,30	0,08	1,00	0	NA	NA	0
MBD5	methyl-CpG binding domain protein 5	0,51	-0,98	0,00	0,05	-1	NA	NA	NA	NA	0	NA	NA	0
GPC6	glypican 6	0,51	-0,98	0,00	0,06	-1	NA	NA	NA	NA	0	NA	NA	0
NA	NA	0,51	-0,98	0,00	0,10	-1	NA	NA	NA	NA	0	NA	NA	0
MANEAL	mannosidase, endo-alpha-like	0,50	-0,99	0,00	0,03	-1	NA	NA	NA	NA	0	NA	NA	0
PHF17	PHD finger protein 17	0,50	-0,99	0,00	0,06	-1	NA	NA	NA	NA	0	NA	NA	0
NA	NA	0,49	-1,03	0,00	0,07	-1	NA	NA	NA	NA	0	NA	NA	0
FAM83C	family with sequence similarity 83, member C	0,49	-1,03	0,00	0,06	-1	NA	NA	NA	NA	0	NA	NA	0
NA	NA	0,49	-1,04	0,00	0,06	-1	NA	NA	NA	NA	0	NA	NA	0
COX7A1	cytochrome c oxidase subunit VIIa polypeptide 1 (muscle)	0,49	-1,04	0,00	0,04	-1	-1,05	-0,07	0,76	1,00	0	NA	NA	0
E2F2	E2F transcription factor 2	0,48	-1,05	0,00	0,08	-1	NA	NA	NA	NA	0	NA	NA	0
ATP6AP1L	ATPase, H+ transporting, lysosomal accessory protein 1-like	0,48	-1,05	0,00	0,04	-1	NA	NA	NA	NA	0	NA	NA	0
VAV3	vav 3 guanine nucleotide exchange factor	0,48	-1,05	0,00	0,05	-1	-1,36	-0,44	0,13	1,00	0	NA	NA	0
C1orf97	chromosome 1 open reading frame 97	0,48	-1,05	0,00	0,08	-1	NA	NA	NA	NA	0	NA	NA	0
ADHFE1	alcohol dehydrogenase, iron containing, 1	0,48	-1,05	0,00	0,09	-1	NA	NA	NA	NA	0	NA	NA	0
CNTN3	contactin 3 (plasmacytoma associated)	0,47	-1,08	0,00	0,05	-1	NA	NA	NA	NA	0	NA	NA	0
EPHX2	epoxide hydrolase 2, cytoplasmic	0,47	-1,08	0,00	0,04	-1	-1,02	-0,03	0,88	1,00	0	NA	NA	0
NA	NA	0,47	-1,08	0,00	0,01	-1	NA	NA	NA	NA	0	NA	NA	0
ARHGEF26	Rho guanine nucleotide exchange factor (GEF) 26	0,47	-1,08	0,00	0,08	-1	NA	NA	NA	NA	0	NA	NA	0
ZNF677	zinc finger protein 677	0,47	-1,09	0,00	0,07	-1	NA	NA	NA	NA	0	NA	NA	0
PYCARD	PYD and CARD domain containing	0,47	-1,09	0,00	0,06	-1	-1,44	-0,52	0,00	0,44	0	-1,18	-0,23	0
IL18	interleukin 18 (interferon-gamma-inducing factor)	0,47	-1,10	0,00	0,08	-1	-1,25	-0,32	0,05	1,00	0	NA	NA	0
LOC147670	hypothetical protein LOC147670	0,46	-1,11	0,00	0,09	-1	NA	NA	NA	NA	0	NA	NA	0
NA	NA	0,46	-1,11	0,00	0,05	-1	-1,09	-0,13	0,73	1,00	0	NA	NA	0
PPARG	peroxisome proliferator-activated receptor gamma	0,46	-1,12	0,00	0,07	-1	-1,01	-0,01	NA	NA	0	NA	NA	0
CCDC138	coiled-coil domain containing 138	0,46	-1,13	0,00	0,04	-1	NA	NA	NA	NA	0	NA	NA	0
RASL11B	RAS-like, family 11, member B	0,45	-1,15	0,00	0,04	-1	1,10	0,14	0,45	1,00	0	NA	NA	0
ESPN	espin	0,45	-1,15	0,00	0,08	-1	NA	NA	NA	NA	0	NA	NA	0
LOC100129858	hypothetical LOC100129858	0,45	-1,16	0,00	0,03	-1	NA	NA	NA	NA	0	NA	NA	0
NA	NA	0,45	-1,16	0,00	0,04	-1	NA	NA	NA	NA	0	NA	NA	0
KBTBD3	kelch repeat and BTB (POZ) domain containing 3	0,45	-1,16	0,00	0,06	-1	NA	NA	NA	NA	0	NA	NA	0
TCEA3	transcription elongation factor A (SII), 3	0,45	-1,16	0,00	0,07	-1	NA	NA	NA	NA	0	NA	NA	0
VSIG10	V-set and immunoglobulin domain containing 10	0,45	-1,17	0,00	0,09	-1	NA	NA	NA	NA	0	NA	NA	0
KITLG	KIT ligand	0,45	-1,17	0,00	0,05	-1	NA	NA	NA	NA	0	NA	NA	0
NA	NA	0,44	-1,17	0,00	0,06	-1	NA	NA	NA	NA	0	NA	NA	0
NA	NA	0,44	-1,17	0,00	0,01	-1	NA	NA	NA	NA	0	NA	NA	0
NA	NA	0,44	-1,17	0,00	0,05	-1	NA	NA	NA	NA	0	NA	NA	0
C9orf3	chromosome 9 open reading frame 3	0,44	-1,18	0,00	0,05	-1	-1,04	-0,06	0,61	1,00	0	NA	NA	0
NPL	N-acetylneuraminate pyruvate lyase (dihydrodipicolinate synthase)	0,44	-1,18	0,00	0,08	-1	1,03	0,05	NA	NA	0	NA	NA	0
FLJ27352	hypothetical LOC145788	0,44	-1,19	0,00	0,02	-1	NA	NA	NA	NA	0	NA	NA	0
CAT	catalase	0,44	-1,19	0,00	0,00	-1	-1,12	-0,17	0,53	1,00	0	NA	NA	0
NA	NA	0,44	-1,19	0,00	0,04	-1	NA	NA	NA	NA	0	NA	NA	0
PPWD1	peptidylprolyl isomerase domain and WD repeat containing 1	0,44	-1,20	0,00	0,03	-1	NA	NA	NA	NA	0	NA	NA	0
CAT	catalase	0,42	-1,24	0,00	0,01	-1	-1,17	-0,22	0,65	1,00	0	-1,37	-0,45	0
CARD16	caspase recruitment domain family, member 16	0,42	-1,25	0,00	0,02	-1	NA	NA	NA	NA	0	NA	NA	0
TTC18	tetratricopeptide repeat domain 18	0,42	-1,25	0,00	0,10	-1	NA	NA	NA	NA	0	NA	NA	0
EML6	echinoderm microtubule associated protein like 6	0,42	-1,26	0,00	0,08	-1	NA	NA	NA	NA	0	NA	NA	0
PCLO	piccolo (presynaptic cytomatrix protein)	0,41	-1,30	0,00	0,05	-1	-1,03	-0,05	0,67	1,00	0	-1,02	-0,03	0
04-set	septin 4	0,40	-1,32	0,00	0,07	-1	1,00	0,01	NA	NA	0	NA	NA	0
SLC40A1	solute carrier family 40 (iron-regulated transporter), member 1	0,39	-1,35	0,00	0,06	-1	NA	NA	NA	NA	0	NA	NA	0
LRRC6	leucine rich repeat containing 6	0,39	-1,35	0,00	0,05	-1	-1,04	-0,06	NA	NA	0	NA	NA	0
IL1F7	interleukin 1 family, member 7 (zeta)	0,39	-1,36	0,00	0,08	-1	-1,00	-0,01	NA	NA	0	NA	NA	0
NA	NA	0,39	-1,37	0,00	0,06	-1	1,00	0,00	NA	NA	0	NA	NA	0
LOC653602	hypothetical LOC653602	0,38	-1,38	0,00	0,06	-1	NA	NA	NA	NA	0	NA	NA	0
N4BP2L1	NEDD4 binding protein 2-like 1	0,38	-1,40	0,00	0,06	-1	-1,01	-0,02	NA	NA	0	NA	NA	0
NA	NA	0,38	-1,40	0,00	0,05	-1	NA	NA	NA	NA	0	NA	NA	0
GPRIN2	G protein regulated inducer of neurite outgrowth 2	0,37	-1,42	0,00	0,03	-1	-1,01	-0,01	NA	NA	0	NA	NA	0
ZBTB7C	zinc finger and BTB domain containing 7C	0,37	-1,43	0,00	0,07	-1	NA	NA	NA	NA	0	NA	NA	0
EPSTI1	epithelial stromal interaction 1 (breast)	0,37	-1,43	0,00	0,10	-1	NA	NA	NA	NA	0	NA	NA	0
FAM171B	family with sequence similarity 171, member B	0,37	-1,44	0,00	0,07	-1	NA	NA	NA	NA	0	NA	NA	0
MAF	v-maf musculoaponeurotic fibrosarcoma oncogene homolog (avian)	0,37	-1,44	0,00	0,10	-1	1,07	0,10	0,34	1,00	0	NA	NA	0
AK5	adenylate kinase 5	0,37	-1,45	0,00	0,05	-1	-1,01	-0,02	NA	NA	0	-1,56	-0,65	0
NA	NA	0,36	-1,48	0,00	0,10	-1	NA	NA	NA	NA	0	NA	NA	0
CYP39A1	cytochrome P450, family 39, subfamily A, polypeptide 1	0,35	-1,50	0,00	0,04	-1	-1,05	-0,07	NA	NA	0	NA	NA	0
GOSR2	golgi SNAP receptor complex member 2	0,35	-1,50	0,00	0,05	-1	NA	NA	NA	NA	0	NA	NA	0
NA	NA	0,34	-1,54	0,00	0,03	-1	NA	NA	NA	NA	0	NA	NA	0
C9orf9	chromosome 9 open reading frame 9	0,34	-1,55	0,00	0,04	-1	-1,01	-0,02	NA	NA	0	NA	NA	0
TSC22D3	TSC22 domain family, member 3	0,34	-1,56	0,00	0,06	-1	-1,24	-0,31	0,06	1,00	0	-1,28	-0,36	0
NA	NA	0,34	-1,57	0,00	0,02	-1	-1,09	-0,13	0,66	1,00	0	NA	NA	0
SEMA4G	sema domain, immunoglobulin domain (Ig), transmembrane domain (TM) and short cytoplasmic domain, (semaphorin) 4G	0,34	-1,57	0,00	0,04	-1	-1,00	0,00	NA	NA	0	NA	NA	0
SLC45A4	solute carrier family 45, member 4	0,33	-1,59	0,00	0,06	-1	NA	NA	NA	NA	0	NA	NA	0

NA	NA	0,33	-1,62	0,00	0,01	-1	-1,12	-0,16	0,65	1,00	0	NA	NA	0
ZNF323	zinc finger protein 323	0,32	-1,62	0,00	0,01	-1	1,19	0,26	0,27	1,00	0	NA	NA	0
GPR37	G protein-coupled receptor 37 (endothelin receptor type B-like)	0,32	-1,63	0,00	0,01	-1	-1,03	-0,05	0,80	1,00	0	NA	NA	0
NHLH2	nescient helix loop helix 2	0,32	-1,65	0,00	0,05	-1	-1,33	-0,41	0,14	1,00	0	NA	NA	0
NA	NA	0,32	-1,67	0,00	0,04	-1	NA	NA	NA	NA	0	NA	NA	0
FAM70A	family with sequence similarity 70, member A	0,31	-1,68	0,00	0,06	-1	-1,02	-0,03	NA	NA	0	NA	NA	0
EPHB6	EPH receptor B6	0,31	-1,70	0,00	0,00	-1	-1,18	-0,24	0,12	1,00	0	NA	NA	0
ITGAM	integrin, alpha M (complement component 3 receptor 3 subunit)	0,31	-1,70	0,00	0,10	-1	-1,01	-0,01	NA	NA	0	NA	NA	0
ITGBL1	integrin, beta-like 1 (with EGF-like repeat domains)	0,31	-1,71	0,00	0,07	-1	-1,04	-0,06	NA	NA	0	-2,13	-1,09	0
LOC100289632	hypothetical protein LOC100289632	0,30	-1,72	0,00	0,03	-1	NA	NA	NA	NA	0	NA	NA	0
PYDC1	PYD (pyrin domain) containing 1	0,30	-1,75	0,00	0,04	-1	NA	NA	NA	NA	0	NA	NA	0
NA	NA	0,29	-1,77	0,00	0,01	-1	-1,12	-0,17	0,59	1,00	0	NA	NA	0
NCRNA00086	non-protein coding RNA 86	0,28	-1,82	0,00	0,01	-1	NA	NA	NA	NA	0	NA	NA	0
NA	NA	0,28	-1,85	0,00	0,00	-1	-1,18	-0,24	0,45	1,00	0	NA	NA	0
ROR1	receptor tyrosine kinase-like orphan receptor 1	0,27	-1,89	0,00	0,06	-1	NA	NA	NA	NA	0	NA	NA	0
TNNT2	troponin T type 2 (cardiac)	0,27	-1,90	0,00	0,02	-1	-1,01	-0,02	NA	NA	0	NA	NA	0
NA	NA	0,26	-1,95	0,00	0,02	-1	NA	NA	NA	NA	0	NA	NA	0
CRNN	cornulin	0,26	-1,95	0,00	0,00	-1	-1,00	-0,01	NA	NA	0	NA	NA	0
MYB	v-myb myeloblastosis viral oncogene homolog (avian)	0,26	-1,97	0,00	0,05	-1	1,00	0,00	NA	NA	0	NA	NA	0
PDE4C	phosphodiesterase 4C, cAMP-specific	0,25	-1,99	0,00	0,06	-1	NA	NA	NA	NA	0	NA	NA	0
BHLHE22	basic helix-loop-helix family, member e22	0,24	-2,06	0,00	0,10	-1	NA	NA	NA	NA	0	NA	NA	0
NA	NA	0,24	-2,07	0,00	0,09	-1	NA	NA	NA	NA	0	NA	NA	0
NA	NA	0,21	-2,26	0,00	0,01	-1	NA	NA	NA	NA	0	NA	NA	0
STMN2	stathmin-like 2	0,20	-2,31	0,00	0,04	-1	-1,01	-0,01	NA	NA	0	NA	NA	0
NA	NA	0,19	-2,36	0,00	0,01	-1	-1,04	-0,06	0,85	1,00	0	NA	NA	0
NA	NA	0,19	-2,36	0,00	0,01	-1	NA	NA	NA	NA	0	NA	NA	0
BTC	betacellulin	0,19	-2,37	0,00	0,07	-1	NA	NA	NA	NA	0	NA	NA	0
ERP27	endoplasmic reticulum protein 27	0,19	-2,41	0,00	0,00	-1	NA	NA	NA	NA	0	NA	NA	0
STMN2	stathmin-like 2	0,18	-2,47	0,00	0,02	-1	-1,00	-0,01	NA	NA	0	NA	NA	0
GPX2	glutathione peroxidase 2 (gastrointestinal)	0,18	-2,49	0,00	0,09	-1	1,07	0,10	0,32	1,00	0	NA	NA	0
SLC46A2	solute carrier family 46, member 2	0,17	-2,59	0,00	0,01	-1	NA	NA	NA	NA	0	NA	NA	0
IGFL2	IGF-like family member 2	0,17	-2,59	0,00	0,01	-1	NA	NA	NA	NA	0	NA	NA	0
NA	NA	0,16	-2,64	0,00	0,01	-1	NA	NA	NA	NA	0	NA	NA	0
ALDH3A1	aldehyde dehydrogenase 3 family, member A1	0,15	-2,69	0,00	0,00	-1	-1,02	-0,02	NA	NA	0	NA	NA	0
MYCT1	myc target 1	0,15	-2,71	0,00	0,01	-1	NA	NA	NA	NA	0	NA	NA	0
SLC47A2	solute carrier family 47, member 2	0,15	-2,74	0,00	0,00	-1	NA	NA	NA	NA	0	NA	NA	0
CXCL14	chemokine (C-X-C motif) ligand 14	0,14	-2,86	0,00	0,01	-1	NA	NA	NA	NA	0	NA	NA	0
KRT77	keratin 77	0,14	-2,87	0,00	0,01	-1	NA	NA	NA	NA	0	NA	NA	0
AKR1B10	aldo-keto reductase family 1, member B10 (aldose reductase)	0,13	-2,90	0,00	0,01	-1	1,20	0,27	0,48	1,00	0	NA	NA	0
NA	NA	0,13	-2,92	0,00	0,02	-1	NA	NA	NA	NA	0	NA	NA	0
FAIM2	Fas apoptotic inhibitory molecule 2	0,13	-2,93	0,00	0,02	-1	-1,01	-0,01	NA	NA	0	NA	NA	0
BTBD16	BTB (POZ) domain containing 16	0,13	-2,99	0,00	0,03	-1	NA	NA	NA	NA	0	NA	NA	0
CXCL14	chemokine (C-X-C motif) ligand 14	0,11	-3,19	0,00	0,02	-1	-2,03	-1,02	0,24	1,00	0	NA	NA	0
SPINK7	serine peptidase inhibitor, Kazal type 7 (putative)	0,10	-3,26	0,00	0,07	-1	NA	NA	NA	NA	0	NA	NA	0
MTTP	microsomal triglyceride transfer protein	0,10	-3,30	0,00	0,04	-1	-1,01	-0,01	NA	NA	0	NA	NA	0
APOB	apolipoprotein B (including Ag(x) antigen)	0,09	-3,40	0,00	0,01	-1	-1,01	-0,01	NA	NA	0	NA	NA	0
ADH1B	alcohol dehydrogenase 1B (class I), beta polypeptide	0,09	-3,42	0,00	0,10	-1	-1,01	-0,02	NA	NA	0	NA	NA	0
TCHH	trichohyalin	0,09	-3,51	0,00	0,01	-1	-1,01	-0,01	NA	NA	0	NA	NA	0
AQP5	aquaporin 5	0,09	-3,54	0,00	0,01	-1	-1,00	-0,01	NA	NA	0	NA	NA	0
DAPL1	death associated protein-like 1	0,08	-3,64	0,00	0,00	-1	NA	NA	NA	NA	0	NA	NA	0
OSR2	odd-skipped related 2 (Drosophila)	0,35	-1,51	0,00	0,07	-1	-1,13	-0,18	0,65	1,00	0	-1,56	-0,64	-1
KRT10	keratin 10	0,61	-0,70	0,00	0,07	-1	-1,53	-0,61	0,00	0,10	-1	-1,02	-0,03	0
CYP4B1	cytochrome P450, family 4, subfamily B, polypeptide 1	0,20	-2,29	0,00	0,10	-1	-4,56	-2,19	0,00	0,07	-1	NA	NA	0

FCH, fold change; FDR, false discovery rate; lg, log
