# Supplementary material for: IL-17 Induces an Expanded Range of Downstream Genes in Reconstituted Human Epidermis Model
Source: PLoS One. 2014 Feb 28;9(2):e90284. doi: 10.1371/journal.pone.0090284 (PMC3938679; doi:10.1371/journal.pone.0090284)
Supplement: Table S2 — Antibodies used for immunohistochemistry and immunofluorescence. (DOCX) [file pone.0090284.s008.docx]

| Antigen | Manufacturer | Isotype | Clone | Amplification/  detection | Catalog number |
| --- | --- | --- | --- | --- | --- |
| CEBPβ | Abcam, MA, USA | IgG1 | A16 | purified mouse anti-human monoclonal antibody | [ab18336] |
| LCN2 | Abcam, MA, USA | IgG1 | 5G5 | purified mouse anti-human monoclonal antibody | [ab23477] |
| HBD2 | PeproTech, NJ, USA | IgG | - | purified goat anti-human polyclonal antibody | [500-P161G] |
| STAT1 | BDbiosciences, NJ, USA | IgG1 | A431 | purified mouse anti-human monoclonal antibody | [610115] |
| RFX5 | Santa Cruz Biotechnology, Inc., CA, USA | IgG | - | purified goat anti-human polyclonal antibody | [sc-10667] |

**Table S2. Antibodies used for immunohistochemistry and immunofluorescence**
